# Impact of constitutional copy number variants on biological pathway evolution

**DOI:** 10.1186/1471-2148-13-19

**Published:** 2013-01-23

**Authors:** Maria Poptsova, Samprit Banerjee, Omer Gokcumen, Mark A Rubin, Francesca Demichelis

**Affiliations:** 1 Department of Pathology and Laboratory Medicine, Weill Cornell Medical College, New York, NY, USA; 2 Department of Public Health, Weill Cornell Medical College, New York, NY, USA; 3 Department of Pathology, Brigham and Women’s Hospital and Harvard Medical School, Boston, Massachusetts, USA; 4 Centre for Integrative Biology, University of Trento, Trento, Italy; 5 Institute for Computational Biomedicine, Weill Cornell Medical College, New York, NY, USA

**Keywords:** CNVs, Pathways, Pathway evolution, Population genetics, eQTL

## Abstract

**Background:**

Inherited Copy Number Variants (CNVs) can modulate the expression levels of individual genes. However, little is known about how CNVs alter biological pathways and how this varies across different populations. To trace potential evolutionary changes of well-described biological pathways, we jointly queried the genomes and the transcriptomes of a collection of individuals with Caucasian, Asian or Yoruban descent combining high-resolution array and sequencing data.

**Results:**

We implemented an enrichment analysis of pathways accounting for CNVs and genes sizes and detected significant enrichment not only in signal transduction and extracellular biological processes, but also in metabolism pathways. Upon the estimation of CNV population differentiation (CNVs with different polymorphism frequencies across populations), we evaluated that 22% of the pathways contain at least one gene that is proximal to a CNV (CNV-gene pair) that shows significant population differentiation. The majority of these CNV-gene pairs belong to signal transduction pathways and 6% of the CNV-gene pairs show statistical association between the copy number states and the transcript levels.

**Conclusions:**

The analysis suggested possible examples of positive selection within individual populations including NF-kB, MAPK signaling pathways, and Alu/L1 retrotransposition factors. Altogether, our results suggest that constitutional CNVs may modulate subtle pathway changes through specific pathway enzymes, which may become fixed in some populations.

## Background

The study of human genome variation over the last several years has expanded from single nucleotide polymorphisms (SNPs) to structural variants (SVs) of which copy number variants (CNVs) constitute a distinct class [1,2]. CNVs are unbalanced SVs that are present in polymorphic copy number states, and include deletions, duplications, or combinations thereof. Recently, a comprehensive population-wide CNV map of the human genome was published [3]. The authors reported on a large set of common CNVs (Minor Allele Frequency (MAF) > 5%) greater than 1 kilobase (kb) in length and genotyped 4,978 of them on 450 individuals with ancestry in Europe, Africa or Asia (the HapMap collection, http://www.hapmap.org). In addition, a recent report from the 1000 Genome Project [4] included about 20,000 deletion polymorphisms (MAF > 1%) in the same HapMap populations from European, African and Asian ancestry, and genotypes were inferred for most of the variants. Further Mills et al. characterized the majority of these deletions at sequence level resolution [5], and proposed a detailed map of SV hotspots formed by common mechanisms.

CNVs have now been implicated with multiple common human diseases (see [6,7] for comprehensive reviews) including Crohn’s disease (20-kb deletion upstream IRGM) [8], osteoporosis (117-kb deletion of UGT2B17) [9], body mass index (45-kb deletion upstream of NEGR1)[10], and decreased susceptibility to HIV (higher copy number of CCL3L1) [11]. CNVs have also been shown to be associated with high risk of autism [12,13], schizophrenia [14,15] and cancer [16,17].

The effect of SNPs on gene expression was recently described through extensive expression Quantitative Trait Locus (eQTL) analysis in European and African populations [18,19]. In 2007, Stranger et al. [20] performed transcripts association analysis both with SNPs and with CNVs, and highlighted similarities and differences for the two types of variation. Recently, two independent studies focused on the characterization of the potential impact of CNVs on gene transcripts by systematically querying CNV copy number states and paired gene expression levels obtained by RNA sequencing on a common set of 129 individuals. Our group [21] queried more than 5,000 CNVs and showed that short CNVs (< 1 kb) and gains are more likely to have functional impact with respect to larger CNVs and deletions, respectively. Schlattl et al. [22] observed that CNVs exhibit a stronger correlation with expression than nearby SNPs and suggested a frequent causal role of CNVs in expression quantitative trait loci.

Several human polymorphism studies, mainly based on SNPs, have been undertaken to identify human genome regions that are under selection (a summary of genome-wide scans for positive selection can be found in Akey et al. [23]; identification of areas of balancing selection and of classic selective sweeps in Andres et al. [24] and Hernandez et al. [25], respectively). The integrated map of positive selection from Akey et al. [23] suggested that positive selection targets encompass ~14% of the human genome and ~23% of all UCSC RefSeq genes. In the context of SNPs, the signature of positive selection includes a high proportion of function-altering mutations, site frequency spectrum with high frequency of the derived allele and low genetic diversity (as a signature of complete or partial sweep), different allele frequency between populations, and long haplotypes [26]. In the context of CNVs, positive selection could be suggested by allele frequencies that significantly differ between populations and by linkage disequilibrium with SNPs under positive selection [27,28]. Alternatively, population differentiation could be a result of genetic drift, however the latter is more significant for small populations [29]. For instance, the salivary amylase gene (*AMY1*) copy number varies considerably across populations and correlates with dietary starch prevalence, which supports the hypothesis of positive selection acting on *AMY1* copy number in high-starch diet populations [30]. Another example is the complex evolutionary history of the polymorphic *UGT2B17* gene that shows high population differentiation [31] (see Additional file 1). Recently, *RHOXF2* has been reported as fast-evolving homeobox gene in primates with rapid evolution and copy number changes driven by Darwinian positive selection acting on the male reproductive system [32].

Based on the observation of close genetic distances at the nucleotide level between human and chimpanzee, the hypothesis that regulatory mutations account for the majority of biological differences was proposed as early as 1975 [33]. More recent work suggests that gene expression levels can also serve as targets of selection [34]. In the past, few studies focused on individual genes’ substitution rates (non-uniform across the genome) suggested that different types of selection act on genes depending on their position in the pathway [35,36]. Here, we posit more broadly that pathways can be subjects for selection.

Existing models of pathway evolution, such as the Horowitz retrograde model [37], the chemistry-driven patchwork model [38], and others (see [39] for a review on pathway evolution theories) consider major modifications to a pathway chain, such as recruitment of a new enzyme (new node) or a whole pathway duplication, that eventually lead to creation of a new pathway. In addition to these major changes, we argue that more subtle ones might play an important role. Specifically, we hypothesize that fixed changes in gene product concentration levels - resulting from the changes in gene transcription levels - act as *small* adjustments while we assume that the number of pathway nodes and the pathway structure remain unchanged.

In this study, we performed an analysis of 491 well-characterized biological pathways seeking to determine how CNVs mapping to pathway genes impact transcript levels and how this effect differs across populations. Our results suggest that CNVs may modulate subtle changes in pathways at specific nodes, which may become fixed in certain populations. We propose a model of pathway evolution where population differences are tuned at finite nodal points. We discuss here the role of CNVs as potential modulators of biological pathways in human genome evolution.

## Methods

### CNVs and HapMap genotype data

The complete set of 11,700 CNV coordinates from [3] was considered in the pathways enrichment/depletion analysis. Based upon the availability of high-resolution data [3] at single sample level, we considered genotype calls for 4,978 CNVs and then used this annotated set for population differentiation analysis. The sample set included 180 CEU (Utah residents with ancestry from Northern and Western Europe), 180 YRI (Yoruba from Ibadan, Nigeria), 45 JPT (individuals from Tokyo, Japan), and 45 CHB (individuals from Beijing, China) from the International HapMap Consortium (http://www.hapmap.org). Throughout this study, Japanese and Chinese individuals are collectively referred to as Asian (ASN). All the data were downloaded from Conrad et al. [3]. Classification of CNVs as gains and losses was taken exactly as inferred in [3].

### Gene annotation and pathway information

RefSeq Gene annotation information was downloaded from the University of California-Santa Cruz (UCSC) Web browser [40] as NCBI build 36 (hg18). The complete list of pathways and corresponding genes was compiled from Kyoto Encyclopedia of Genes and Genomes (KEGG) [41] and Biocarta (http://www.biocarta.com).

### F-statistics and CNV frequencies

Population differentiation was evaluated using the F-statistics [42] that ranges between 0 (completely undifferentiated) and 1 (highly differentiated). F-statistics was calculated for each population pair by considering each CN genotype as an allele for diallelic CNVs as in McCarroll et al. [43]. The F-statistics (F_st_) was evaluated as follows: F_st_ = (H_t_-H_s_) / H_t_ ; H_t_ = 1 - ∑ t_i_^2^; t_i_ =( (x_i_ · N_x_) + (y_i_ · N_y_) ) / (N_x_+N_y_); Hs =( (1 - ∑ x_i_^2^) · N_x_ + (1 - ∑ y_i_^2^) · N_y_ ) / (N_x_+N_y_), where x_i_ and y_i_ are the population frequencies of allelic copy number *i* (*i* = A0, A1, A2, A3, A4 or >A4) in population X and Y, respectively, N_x_ and N_y_ denote the number of individuals in population X and Y, and t_i_ is a weighted average of x_i_ and y_i_. This approach ignores the phase of the haplotype.

F-statistics cutoffs corresponding to the 5% and 1% tails of the distributions built for each population pair were considered. Specifically, the following values were calculated and applied: Fst=0.19 (P<0.05) and Fst=0.32 (P<0.01) for CEU-YRI; Fst=0.2 (P<0.05) and Fst=0.36 for (P<0.01) for YRI-ASN; Fst=0.14 (P<0.05) and Fst=0.24 (P<0.01) for CEU-ASN.

Beside the formal assessment of population differentiation by means of the F-statistics, we considered the frequency of polymorphisms, referred to as CNV frequency, as the sum of the frequencies of all CN states that differ from the major CN state. CNV frequencies are utilized in the generation of frequency heatmaps. For consistency, we selected the CEU major CN state for each CNV. This choice does not affect the results as we focus on differences rather than absolute values.

### Size-dependent CNV enrichment/depletion analysis

**Figure 1:**
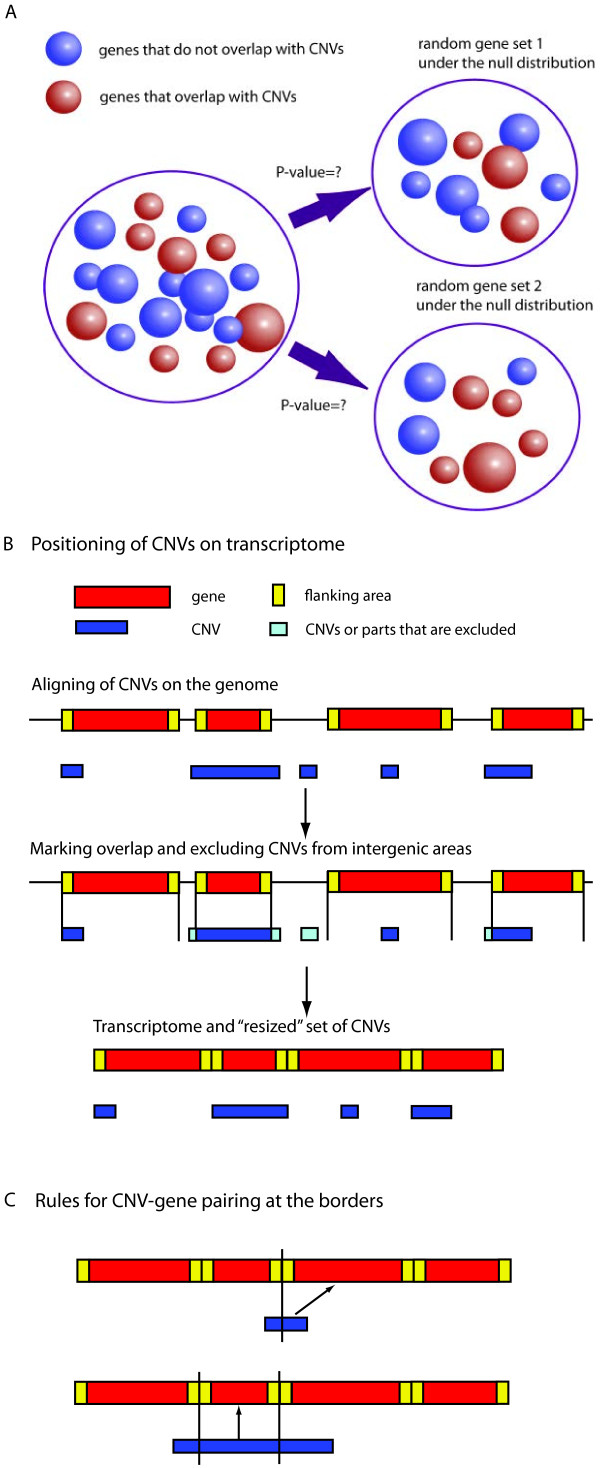
**Size-dependent enrichment analysis. *A****. Incorporation of sizes into enrichment analysis.* The size of the objects corresponds to the probability of being drawn. ***B****. Positioning of CNVs on transcriptome.* First, CNVs are aligned on the genome. Second, CNVs or part of CNVs that fell outside of the gene areas are excluded or truncated. Third, transcriptome and newly “resized” set of CNVs are used for permutations. ***C****. Rules for CNV-gene pairing at the borders.* If CNV falls on the boundary between two gene areas then we count overlap for the gene that contain more than 50% of CNV length. Second, we count overlap with the gene if it is entirely inside a CNV.

Given a set of CNVs and a set of genes (e.g., gene families, pathways, Gene Ontology (GO) categories), the approach estimates if the number of observed CNV-gene overlaps is significantly higher (enriched) or lower (depleted) than what it would be expected by chance.To evaluate enrichment or depletion of pathways for the presence of overlapping CNVs, we developed a size-dependent enrichment method that takes into account the sizes of genes and CNVs and we empirically simulated the null distribution through permutations. The approach overcomes the limitation of commonly applied statistical tests that do not consider either CNV or gene size information. In reality, gene families widely differ in terms of gene sizes (e.g., the ETS family of transcription factors and the keratin gene families have average gene sizes of 55 kb and 3 megabases (Mb), respectively). A schematic representation of this problem is presented in Figure 1A where the blue (red) circles represent genes overlapping (not overlapping) with CNVs.

The following steps describe the method: First, for each chromosome, we compose a “pseudo transcriptome” made of concatenated genes (regions from transcription start to transcription end positions) with flanking regions (Figure 1B). For simplicity, for genes with multiple isoforms coordinates corresponding to the widest size are considered. We then define a ‘gene area’ as the area that spans the gene and upstream and downstream flanks. Second, we refine the CNV set by retaining only CNVs that overlap with gene areas. In case of partial overlap, the CNV segment inside the gene area is retained (Figure 1B). Third, the null distribution of enrichment is constructed by permuting this new set of truncated CNVs on the transcriptome by maintaining the number and size of CNVs per chromosome. Last, we define simple rules to count CNV-gene overlap as illustrated in Figure 1C; the gene area is entirely within a CNV or the gene spans at least 50% of a CNV. Finally, for each pathway, the p-value is calculated by comparing the observed number of CNV-gene overlaps with the empirical null distribution constructed by the permutations.

It is important to mention few aspects that were not considered in the implementation of the null distribution. First, the utilized CNVs genomic distribution information is limited by the use of array-based platforms. Second, paralogous genes, likely relevant in the context of segmental duplications where CNVs are significantly enriched, were not considered. More refined approaches should eventually address these limitations.

The Size Dependent Enrichment Test executable and source code is downloadable at: http://demichelislab.unitn.it/tools/SizeEnrichmentTest.tar.gz.

### Graphical representation of pathway related to CNV frequencies across populations

For each pathway, we considered the subset of genes overlapping with CNVs based on the gene areas (defined above) and represented it with the corresponding set of CNV-gene pairs. Based upon the systematic analyses of short- and long-range effect of CNVs on gene transcript levels [3,20,21], we considered 10 kb and 1 Mb flanks and composed two CNV-gene frequency maps. The 10 kb flanks map will capture the majority of the significant associations between CNVs and genes (Stranger et al. 2007) and the 1 Mb flanks map will include longer distance effects. We used 10 kB flanks for size-dependent enrichment/depletion analysis, and 1 Mb flanks for CNV association analysis with gene expression levels. For population differentiation analysis at the level of individual genes and of pathways we used 10 kb flanks to link genes and CNVs. For population differentiation analysis of genes and pathways linked to “functional” CNVs we used 1 Mb flanks. One CNV can overlap multiple genes and vice-versa one gene can encompass (‘hit by’) several CNVs. Therefore the number of CNV-gene pairs is not necessarily bound by the number of CNVs. Using 10 kb flanks we counted 5,282 genes paired with CNVs and using 1 Mb flanks we counted 21,378 genes paired with CNVs.

Heatmaps were used to graphically represent the CNV polymorphism frequency in each population, with CNV-gene pairs in rows, and populations in columns (YRI, CEU, and ASN). Hierarchical clustering (complete linkage with Euclidean distances) was applied to identify groups of similar CNV-gene frequencies across the three populations.

### Gene expression data and association analysis between CNVs and gene expression levels

RNA-seq data for CEU and 69 YRI HapMap individuals were downloaded from [18] and [19]. Sequencing data were originally generated using the Illumina Analyzer II with 36-base and 35 or 46-base pairs, respectively. YRI individual data were downloaded from http://eqtl.uchicago.edu, CEU individual raw data were obtained from ArrayExpress under accession numbers E-MTAB-197 and preprocessed applying RSEQtools [44]. Association analysis between gene expression data and CNVs was performed independently for CEU and YRI individuals by *cis* analysis applying 1 Mb flanks to each variant accordingly to [20]. Dosage effect (linear model) and allelic effect of transcript levels versus the copy number states were tested [21]. Multiple hypothesis testing correction was evaluated by calculating the False Discovery Rate (Benjamini 1995) on the p-values; 10% and 5% thresholds were applied. We will refer to CNV-gene pair with significant association between CNV states and gene expression levels as functional CNV-gene pair.

### Mechanisms of CNV formation

Mechanisms of CNV formation were inferred as described in [21] and are divided in four major classes: variable number of tandem repeats (VNTR), non-allelic homologous recombination (NAHR), transposable elements insertion (TEI) and non-homologous recombination (NHR). RepeatMasker annotation tracks were downloaded from UCSC browser to infer VNTR and TEI mechanisms. Two-sample test of proportions was applied to assess the significant differences in proportions of mechanism formation classes for CNVs differentiated in populations.

## Results

### CNVs enrichment/depletion analysis at the level of pathways

**Figure 2:**
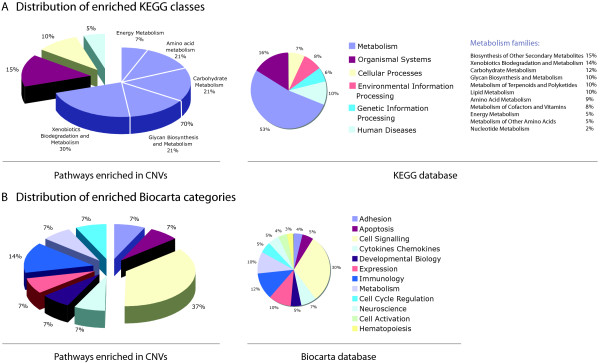
**Distribution of enriched KEGG and Biocarta pathway classes*. A****. Distribution of enriched KEGG classes.* Original distribution of pathway classes in KEGG database is given on the left. Distribution of the KEGG classes enriched (p-value < 0.05) in CNVs that were obtained with the size-dependent enrichment analysis is given on the right. ***B****. Distribution of enriched Biocarta categories.* Original distribution of pathway categories in Biocarta database is given on the left. Distribution of the Biocarta categories enriched (p-value < 0.05) in CNVs that were obtained with the size-dependent enrichment analysis is given on the right.

We analyzed all annotated pathways from KEGG [41] and Biocarta (http://www.biocarta.com) (N=491) for CNV enrichment. For each pathway, we identified a set of CNV-gene pairs based on genomic co-location and applied size-dependent enrichment/depletion analysis (see Methods). The list of significantly enriched pathways is given in Table 1 (Additional file 2: Table S1 shows the complete list) and the distribution of enriched KEGG functional classes and Biocarta categories are presented in Figure 2. Despite the general belief that CNVs are enriched mostly in extracellular and signaling pathways [3,45], our results indicate significant enrichment in key metabolic pathway classes, such as carbohydrate (Pentose and glucuronate interconversions and Inositol metabolism), xenobiotic (Carbazole degradation, Fluorene degradation and Metabolism of xenobiotics by cytochrome P450) and glycan (Peptidoglycan biosynthesis) metabolisms (see Table 1). Among the metabolic pathways that are enriched in CNVs are the pathways from amino-acid (Urea cycle and metabolism of amino groups, Cyanoamino acid metabolism and Glutathione metabolism) and energy (Sulfur metabolism) metabolisms.

**Table 1:** List of pathways enriched for CNV-gene pairs through size-dependent analysis (10 kb flanks)

	**Pathway**	**Source**	**Gene CNV**	**Gene total**	**P-value**	**Fst**
1	Antigen processing and presentation	KEGG	29	79	<0.001	
2	Metabolism of xenobiotics by cytochrome P450	KEGG	22	60	<0.001	0.31(CEU|YRI),0.23(YRI|CHB),0.16(CEU|CHB),
3	Type I diabetes mellitus	KEGG	17	41	<0.001	
4	Keratinocyte Differentiation	BIOCARTA	16	18	<0.001	0.53(CEU|YRI), 0.57(YRI|CHB)
5	Pentose and glucuronate interconversions	KEGG	9	14	<0.001	0.16(CEU|CHB), 0.28(YRI|CHB)
6	The role of FYVE-finger proteins in vesicle transport	BIOCARTA	6	7	<0.001	0.12(CEU|YRI), 0.37(CEU|CHB),0.47(YRI|CHB),
7	Phospholipase C d1 in phospholipid associated cell signaling	BIOCARTA	4	5	<0.001	0.53(CEU|YRI), 0.67(YRI|CHB),
8	Activation of cAMP-dependent protein kinase, PKA	BIOCARTA	2	3	<0.001	
9	B Cell Receptor Complex	BIOCARTA	1	2	<0.001	
10	Carbazole degradation	KEGG	1	1	<0.001	
11	Fluorene degradation	KEGG	1	2	<0.001	
12	Inositol metabolism	KEGG	1	2	<0.001	
13	Peptidoglycan biosynthesis	KEGG	1	2	<0.001	
14	Segmentation Clock	BIOCARTA	7	12	0.001	

* Column Fst lists the highest Fst value for each population pair. For the full list of pathways with CNV-gene pair annotation and corresponding Fst values, see Additional file 2: Table S1.

Enriched signaling pathways include Keratinocyte differentiation (16 genes out of 18, size-dependent enrichment test) and Phospholipase C d1 in phospholipid associated cell signaling (4 genes out of 5, size-dependent enrichment test) (see Table 1). The list of depleted pathways and distribution of KEGG and Biocarta functional classes is given in Additional file 2: Table S1 and Additional file 3: Figure S1. Distribution of the depleted pathway classes is similar with that of all pathway classes in both databases.

Focusing on the pathways enriched for the presence of CNVs, we compared the overall proportions of four CNV formation mechanisms and found evidence for enrichment of NHR (1.12 fold, P-value= 0.00014) and depletion of TEI (0.63 fold, P-value= 0.0019) formation classes. With respect to CNV types, gains or losses, depletion was observed for losses (0.89 fold, P-value= 0.0055) and enrichment for gains (1.19 fold, P-value= 0.0050).

### Population differentiation at the level of individual genes and of pathways

Population differentiation analysis was performed for each CNV and each population pair-wise, by evaluating the F-statistics (0, completely undifferentiated and 1, highly differentiated) [42] (see Additional file 4: Table S2). Overall 290 differentiated CNV-gene pairs were detected at 5% significance level and 57 differentiated CNV-gene pairs were detected at 1% significance level, including previously reported genes [3,27,42,45,46]. The CNV-gene pairs with the highest differentiation at 1% significance level are presented in Table 2. The distribution of CNV frequency differences for each pair of populations is presented in Additional file 3: Figure S2. The data show that frequency differences are higher between Yoruban and the two other populations than between Europeans and Asians, supporting the observation in [28]. The same trend is observed in the distribution of allele frequency differences for SNPs [47].

**Table 2:** List of top ranked CNVs (gene overlap using 10 kb flanks) for population differentiation (at 1% significance level, see Methods for cutoff values in three population pairs)

**CNV**	**Overlapping genes**	**F-stat**	**Pairs**
CNVR2664.1	ADRA1B	0.53; 0.67	CEU-YRI; YRI_ASN
CNVR3865.1	IKBKB^1^,POLB	0.53; 0.57	CEU-YRI; YRI-ASN
CNVR1543.1	CPNE4	0.52; 0.37	YRI-ASN; CEU-YRI
CNVR1708.1	TFRC	0.47; 0.37	YRI-ASN; CEU-ASN
CNVR371.1	YY1AP1,DAP3	0.47; 0.37	CEU-YRI; YRI-ASN
CNVR2217.1	PDLIM3^1^	0.47; 0.32	CEU-YRI; YRI-ASN
CNVR544.1	SLC35F3^2^	0.46; 0.72	CEU-ASN; YRI-ASN
CNVR1648.3	KCNMB2	0.41; 0.77	CEU-YRI; YRI-ASN
CNVR6782.1	CNTNAP4	0.41; 0.31	YRI-ASN; CEU-YRI
CNVR7114.8	KIAA1267	0.4; 0.3	CEU-YRI; CEU-ASN
CNVR995.1	TUBA3D	0.39; 0.37	CEU-YRI; YRI-ASN
CNVR8147.1	HMGXB4	0.39; 0.36	CEU-YRI; YRI-ASN
CNVR1373.1	PRSS45	0.37; 0.31	CEU-YRI; YRI-ASN
CNVR3347.1	BBS9	0.35; 0.35	CEU-ASN; YRI-ASN
CNVR2152.1	TLL1	0.32; 0.34	CEU-YRI; YRI-ASN
CNVR4440.1	TXN	0.54	YRI-ASN
CNVR2807.2	FLJ22536	0.53	CEU-ASN
CNVR3563.1	GCC1	0.49	YRI-ASN
CNVR3398.2	PSPH	0.47	CEU-YRI
CNVR1841.1	SLIT2	0.46	YRI-ASN
CNVR95.2	PADI4	0.43	YRI-ASN
CNVR2906.1	C6orf142	0.42	CEU-YRI
CNVR7722.1	LILRA3 LILRA5	0.42	YRI-ASN
CNVR6372.1	SORD	0.42	CEU-YRI
CNVR5162.1	C11orf49	0.41	CEU-YRI
CNVR6117.1	KIAA0391	0.41	YRI-ASN
CNVR203.1	L1TD1	0.4	YRI-ASN
CNVR1009.1	ZRANB3	0.4	CEU-YRI

* The index refers to the original study where the gene has already been reported for population differentiation.

^1^[3].

^2^[45].

**Figure 3:**
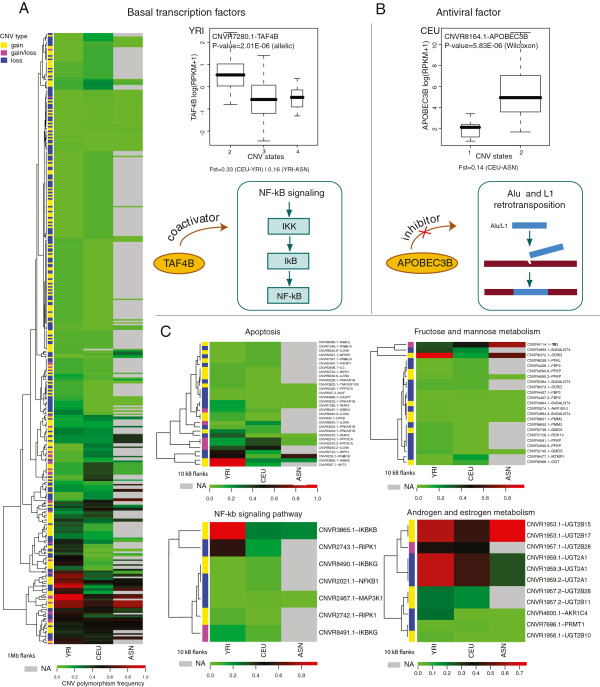
**CNV-gene frequency heatmaps *A.**** CNV-gene frequency heatmap for Basal transcription factors pathway and example of the significant gene variant association for TAF4B gene from this pathway taken from YRI RNA-seq data.* In the heatmap, rows correspond to CNV-gene pairs and columns correspond to three Hapmap populations: YRI, CEU and ASN. The values of the heatmap are CNV polymorphism frequency (see Methods). Evidence for population differentiation is given below the box plots. The schematic diagram shows connection of the gene TFA4B with NF-kB signaling pathway (see text for explanations). ***B****. Example of the significant gene variant association for APOBEC3B gene taken from CEU RNA-seq data.* Evidence for population differentiation is given below the box plots. Schematic diagram shows connection of the gene APOBEC3B with Alu/L1 retrotransposition (see text for explanations). ***C****. Examples of CNV-gene frequency heatmaps for four pathways*: *Apoptosis, Fructose and mannose metabolism, NF-kb signaling pathway and Androgen and estrogen metabolism.* Here flanking areas for CNV-gene pairs are 10 kb.

Gene expression levels are also subject to selection in the human genome [48]. At the level of individual genes, the selection acting on a CNV could be linked to transcription levels associated with the CNV states. At the level of pathways, signatures of selection can be traced through the selection acting on individual genes constituting the pathway. To investigate the extent of population differentiation on a pathway basis, we constructed pathway frequency maps (see Methods) that combine polymorphism frequencies of each CNV-gene pair in each population and are visualized using a heatmap (see Figure 3 and Additional file 3: Figure S3). Examples of pathway frequency maps are depicted in Figure 3A and 3C. Focusing on the Apoptosis pathway, we observed that AKT3 and IKBKB related CNVs exhibit higher frequency of polymorphisms in the Yoruban population compared to the European and Asian populations. TSTA3, B4GALNT4 and SORD CNV-gene pairs show significant population differentiation in the map for Fructose and Mannose metabolism, and UGT2B15, UGT2B17, UGT2A1 CNV-gene pairs are seen as highly differentiated in the map for Androgen and Estrogen metabolism (Figure 3)C.

The analysis of all pathway CNV-gene frequency maps (see Additional file 3: Figure S3) suggested a characteristic pattern. Generally, a small proportion (6% on average) of CNV-gene per pathway show strong population differentiation (at least one CNV-gene pair in the pathway has significant population differentiation) while the remaining CNV-gene pairs are weakly differentiated or undifferentiated as we can observe for Apoptosis, Fructose and Mannose metabolism and NF-kb signaling pathway (Figure 3C). A total of 107 pathways exhibited population differentiation in the sense that at least one CNV-gene pair is differentiated (P<0.05), among them 41 pathways showed the highest population differentiation (P<0.01). This list includes the Purine metabolism (P<0.01), the Starch and sucrose metabolism (P<0.01), the Pentose and glucuronate interconversions (P<0.01), and other highly differentiated metabolic pathways (see the full list in Additional file 5: Table S3).

**Figure 4:**
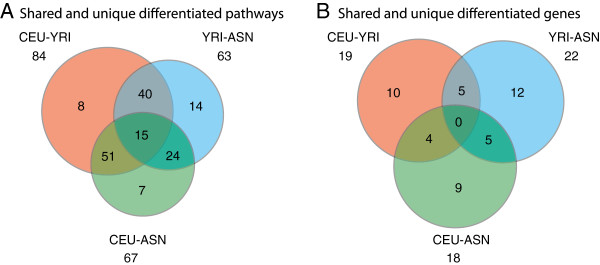
**Venn diagrams of the number of pathways and genes that show population differentiation. *A****. Venn diagram of the number of pathways that have at least one gene that shows population differentiation* (via CNV in 10 kb flank) (see the full list in the Additional file 6: Table S4). ***B***. *Venn diagram of the number of genes that show population differentiation* (via CNV in 10 kb flank. (see the full list in the Additional file 7: Table S5).

Figure 4A presents a Venn diagram of the differentiated pathways for each population pair. The corresponding lists are given in Additional file 6: Table S4. Among the metabolic class pathways, the Valine, leucine and isoleucine degradation (P<0.05), the Glycerolipid metabolism (P<0.05) and the Purine metabolism (P<0.01) show significant differentiation between only European and Yoruban populations; pathways such as Arginine and proline metabolism (P<0.05), Pyrimidine metabolism (P<0.05), ER-associated degradation (ERAD) pathway (P<0.05), Alkaloid biosynthesis (P<0.05), Glycan structure biosynthesis (P<0.05) and Glycosylphosphatidylinositol(GPI)-anchor biosynthesis (P<0.05) show differentiation between only Yoruban and Asian populations; the Pentose and glucuronate interconversions (P<0.01), the Starch and sucrose metabolism (P<0.01), the Androgen and estrogen metabolism (P<0.01), the Porphyrin and chlorophyll metabolism (P<0.01), the Metabolism of xenobiotics by cytochrome P450 (P<0.01) were detected as significantly differentiated between only European and Asian populations. The Metabolism of xenobiotics by cytochrome P450 is a metabolic pathway that is also significantly (P<0.05) differentiated between all three population pairs. Similarly, a Venn diagram for the differentiated CNV-genes from the above mentioned pathways is depicted in Figure 4B and the corresponding list of unique and shared genes for each population pair is given in Additional file 7: Table S5. 45 genes were detected as population differentiated with P<0.05, among them 9 genes are highly differentiated (P<0.01): ADCY8, ADRA1B, GRID2, IKBKB, POLB, SLIT2, TFRC, UGT2B15, UGT2B17. The gene that showed polymorphic copy number states in all three populations and significant population differentiation (P<0.05) in two population pairs (CEU-YRI and CEU-ASN) is SORD. SORD encodes the sorbitol dehydrogenase enzyme and participates in the Fructose and mannose metabolism pathway (see heatmap in Figure 3C). SORD converts sorbitol to fructose, and the latter can then be metabolized via the glycolytic pathway to produce adenosine triphosphate (ATP). The polymorphism frequency of the SORD CNV-gene pair is 97% in the YRI population, 16% in the CEU population, and 66% in ASN population. The CNV is located in the first gene intron that often contains regulatory elements, however, we did not find evidence for regulatory elements based on the ENCODE histone marks data. We tested for significant differences between proportions of CNV formation mechanisms for population differentiated CNVs and found that the proportion of TEI class was significantly higher (1.85-fold, P-value=2.2e-16) while that of VNTR and NHR was significantly lower (0.25-fold, P-value= 1.773e-06 and 0.88 fold, P-value= 8.496e-07). Proportion of NAHR class was not significantly different in the three population pairs.

### Association of CNVs with gene expression at the level of genes and pathways

The proximity between a CNV and a gene can be considered an indicator for transcription regulation effect. However, this only occurs for a fraction of variants. To better assess the potential regulator effect of CNVs on gene transcripts, we considered RNA sequencing gene expression data from 60 CEU [18] and 69 YRI [19] individuals (see Methods) as in [21]. Overall, we detected significant CNV gene expression association (FDR < 5%) involving 54 functional CNV-gene pairs in CEU of which 16 could be mapped to 24 pathways, and 37 functional CNV-gene pairs in YRI of which 8 genes could be mapped to 14 pathways. Focusing on the set of functional CNV-gene pairs, we performed CNV enrichment/depletion analysis (using Hypergeometric test) for the set of 24 pathways in CEU and 14 pathways in YRI (Additional file 8: Table S7) and found a significant enrichment of functional CNV-gene pairs for one pathway, the Atrazine degradation pathway, in CEU and for 14, mostly metabolic, pathways in YRI. The latter includes Glutathione metabolism, Starch and sucrose metabolism, Cyanoamino acid metabolism, Androgen and estrogen metabolism and others (see the full list in Additional file 8: Table S7).

Then we focused on functional CNV-gene pairs, which were characterized as significantly differentiated among populations. Out of 81 (in CEU and YRI combined) functional CNV-gene pairs, 13 (16%) showed significant population differentiation (P<0.05), and thus, are potential candidates for positive selection. Of the genes linked to functional CNVs that showed population differentiation (P<0.05), 4 genes (UGT2B17, KRT39, APOBEC3B, and TAF4B) are involved in 8 known pathways (Additional file 9: Table S6): the Androgen and estrogen metabolism, the Metabolism of xenobiotics by cytochrome P450, the Pentose and glucuronate interconversions, the Porphyrin and chlorophyll metabolism, the Starch and sucrose metabolism, the Cell Communication, the Basal transcription factors, and the Atrazine degradation. The highest population differentiation (P<0.01) among functional CNVs was detected for 2 CNV-gene pairs: one is for the gene FAM128A, and the other for the gene TAF4B from the Basal transcription factors pathway. Illustrations of significant associations between CNV and gene expression are presented for the gene TAF4B belonging to the Basal transcription factors pathway from YRI data (Figure 3A) and for the gene APOBEC3B belonging to the Atrazine degradation pathway from CEU data (Figure 3B). TAF4B encodes a subunit of transcription initiation factor that has been shown to function as co-activator of genes from NF-kB signaling pathway [49]. Functional CNV for TAF4B is 4 kb large and located 58 kb upstream the gene of 165 kb. Observed CN states are 2, 3, and 4, and the expression is the highest for CN equal to 2. CNV population frequencies are 89% in YRI, 15% in CEU, and 42% in ASN. The gene APOBEC3B is a gene from cytidine deaminase family performing C to U RNA-editing. It has been known as an antiviral factor that can act against retroviruses, such as HIV [50], and it has been also found to act as an inhibitor of L1 and Alu retrotranspositon [51]. Functional CNVs for APOBEC3B is 36 kb large and completely removes the gene which is 10 kb in size. Population frequencies are 13% for CEU, 7% for YRI, and 54% for ASN.

Illustrations of associations, though not statistically significant, between CNV and gene expression for genes from the Mitogen-activated protein kinase (MAPK) signaling pathway are presented in Additional file 3: Figure S5. Associations were detected for two genes located consecutively in the pathway chain: first gene is from the CACNG family of gamma subunits important for regulating calcium channels [52], and the second belongs to RASGRP family that activates MAP kinase cascade.

## Discussion

In this study, we performed a comprehensive size-dependent analysis of the impact of common CNVs on 491 biological pathways across human populations, with the idea that abundance of genetic variation in pathways can be indicative of ongoing evolution. We first showed that CNV enriched pathways include not only signaling pathways and pathways involved in extracellular biological processes [3], but also metabolic pathways such as amino-acid, carbohydrate, energy and glycan metabolisms. The Glycan biosynthesis pathway is annotated as originated in vertebrates in a recent phylogenetic study of metabolic pathways in the context of evolution [53] and in the orangutan genome paper [54]. Some of the CNV-gene pairs we report are potential candidates for individual studies intended to investigate pathway-related dysfunctions and metabolic diseases [55] (see Additional file 1).

In order to characterize the extent of human variation at the level of each pathway, we compiled a comprehensive list of population differentiated CNV-gene pairs. As expected, the majority of pathways exhibiting population differentiation, belong to the signaling class, including Calcium, MAPK, Toll-like receptor signaling, and others. Pathways from the metabolic class include Purine metabolism, Arginine and proline metabolism, and Starch and sucrose metabolism with the well documented example of AMY1 polymorphism and its relation to starch diet [30]. These findings provide evidence that signaling pathways and pathways involved in extracellular activities are less conserved and are potentially under the influence of positive or adaptive selection [56]. Comprehensive characterization of the influence of negative selection is mandatory in future studies. Highly differentiated CNVs overlapping or in proximity of genes indicate recent evolutionary events, and emphasize the importance to improve our understanding of selection forces that shape the observed population differentiation [57]. Alternatively genetic drift events [29] might be responsible for the observed population differentiation, however their effect is considered to be significant in small populations. Pathway based analysis revealed CNV-gene pairs with intermediate Fst in addition to highly differentiated CNVs. We reasoned that this might correspond to ‘CNVs genetic hitchhiking’ similar to what was suggested by Barreiro [58] in the context of SNPs.

Recent studies also indicated the pervasiveness of negative, or purifying selection, acting on CNVs. Conrad et al. [3] reported on purifying selection acting on exonic and intronic deletions. Other studies reported on variants under negative selection [58-60]. The methods used for detection of purifying selection were based on the nucleotide level resolution, and restricted solely to the genes. Where our study focuses on the existence of significantly differentiated pattern, it is relevant to highlight that appropriate distinction between positive and purifying selection acting on CNVs is an important challenge that requires extensive future work.

We then investigated pathway enrichment considering the set of CNVs deemed functional through the association between the corresponding transcript levels and the copy number states and focused on those which also show differentiation across populations. Despite the fact that the transcript analysis is limited due to the sample size, the differences in the sequencing depth, and the sample type (i.e., lymphoblast cells), we reasoned that our integrated analysis would benefit from this additional layer of information. Making a simplifying assumption, one can consider that selection acting on transcript levels ultimately influences the gene product leading to population specific selective advantage, as it was shown for UGT2B17 and AMY1 in Asians and Europeans, respectively. Similarly, the variant associated with APOBEC3B (Figure 3B) and present at different frequencies across populations may suggest a population specific antiviral effect, and in particular, population specific effect on Alu-L1 retrotranspositional activity. However, one has to consider that the detected potential “functional” CNVs are not necessarily direct causal variants and might simply be linked to the causal variants.

Throughout the study some well-known cancer-related pathways were detected as enriched in population differentiated CNVs (see Additional file 1). Even though most of the detected genes have been extensively studied in relation to cancer, their differential effect across populations, including differential disease susceptibility, is still to be investigated. The gene TAF4B, whose expression levels are significantly associated with a population differentiated CNV (Figure 3A) is a known co-activator of NF-kB genes further stimulating NF-kB transcriptional activity [61]. It was shown that functional consequences of NF-kB signaling pathway is determined by NF-kB oscillation dynamics [62] and the number, period and amplitude of NF-kB oscillations are regulated, via a negative feedback loop, by transcription levels of IkBα. In a similar way, transcription levels of TAF4B can regulate dynamics of NF-kB genes, providing different functional outcomes for the pathway. MAPK signaling pathway is another example of how the tuning of enzyme concentration can affect signaling pathway. We found population differentiated functional CNVs for CANCG and RASGRP4 located upstream of the MAPK pathway chain (Additional file 1 and Additional file 3: Figure S5). The analysis of the model for this pathway identified existence of two different dynamical regimes, and depending on parameters, the system can switch from single-state bi-stability to oscillations [63]. This means that the MAPK signaling network can act as a switch and as a clock, and the altered (tuned) element concentration levels can initiate such transition or prevent cycling due to a shift in the threshold positions.

The effect of gene concentration level changes on the cellular phenotype and the concept of genetic balance has been addressed in [64], together with the study of dosage-sensitive genes documented in genomes of various species, including yeast and human, and often encoding transcription regulators, signal transduction elements and binding factors [65]. However, we recognized that the complexity of possible downstream changes relies on the non-linear dynamics of biochemical reactions possibly leading to non-proportional change in the concentration of the final component [66].

Here we provide insights into human pathways enriched for population differentiated functionally active CNVs. Under the assumption that a pathway chain remains intact as a whole (i.e., no new enzymes are added), we hypothesize that evolutionary selected changes in transcription level of some genes constituting the pathway “tune” the pathway into a more favorable state for homeostasis, a process we refer to as the ‘tuning effect’. Last, we suggest that new pathways can stem as long-term potential outcome of the proposed tuning effect (Additional file 1 and Additional file 3: Figure S6).

## Conclusion

Upon the characterization of functional CNVs and the concomitant population differentiation of the same variants suggestive of positive selection in different populations, it is challenging to discover the real effect of these changes on a pathway chain and to study the regulatory mechanisms in the cell that control the changes in gene concentration levels. The picture becomes more complicated by acknowledging the multiplicity due to the existence of co-factors concurring to gene regulation, to the presence of other sources of variations, like epigenetic events, and by gene regulation compensatory effects. Our analysis may help to reveal pathway nodes, which have undergone changes (positively, neutrally or negatively) in gene concentrations, or, in other words, pathways that have been tuned. Further studies are required to understand the impact of these and other changes on pathway structure and human diversity.

## Competing interests

The authors declare that they have no competing interests.

## Authors’ contributions

MP carried out computational analyses, implemented the size-dependent enrichment analysis and drafted the manuscript. SB carried out computational and statistical analysis and drafted the manuscript. OG participated in the interpretation of the results. MAR participated in the design of the study and interpretation of the results. FD conceived the study, participated in its design, coordination and implementation and drafted the manuscript. All authors read and approved the final manuscript.

## Supplementary Material

Additional file 1 Supplementary Material. Click here for file

Additional file 2 **Table S1.** Size-dependent enrichment analysis results for all the pathways considered in the study. Click here for file

Additional file 3 **Figure S1.** Distribution of depleted KEGG and Biocarta pathway classes*. ****A****. Distribution of depleted KEGG classes.* Original distribution of pathway classes in KEGG database is given on the left. Distribution of the KEGG classes depleted in CNVs that were obtained with the size-dependent enrichment analysis is given on the right. ***B****. Distribution of the depleted Biocarta categories.* Original distribution of pathway categories in Biocarta database is given on the left. Distribution of the Biocarta categories depleted in CNVs that were obtained with the size-dependent enrichment analysis is given on the right. **Figure S2.** Histogram of CNV frequency differences in three population pairs: CEU-YRI, CEU-ASN and ASN_YRI. CNV frequency is a frequency of polymorphism and calculated as described in the Methods. Frequency differences are given in absolute values. **Figure S3.** CNV-gene frequency heatmaps for 368 pathways. Heatmaps are constructed for CNV-gene pairs with 10 kb flanks. **Figure S4.** Rearrangements around SORD gene area in human and chimpanzee. Figure shows Mauve block alignment of four homologous regions in human and chimpanzee. First two regions are extracted from human reference genome (build hg18): chr15:43,080,000-43,163,000 and chr15:42,917,000-43,079,000, and the second two regions are taken from chimpanzee genomes (build panTro2): chr15:42,173,000-42,250,000 and chr15:41,950,000-42,030,000. The region of ~ 80 kb that includes the gene SORD underwent inverse duplication before the split of human and chimpanzee. The active copy of the gene SORD is encoded on the plus strand and is shown in the orange color. The copy of the gene SORD on the minus strand, that most likely became a pseudogene, is shown in the light orange. CNV resulted from a loss of a region in human genome from the inverted copy of the gene SORD (see empty box at the second alignment row). Analysis of the RepeatMasker annotation revealed that in the chimpanzee, the L1 element (L1PA3) is located right next to the CNV boundary. In the corresponding region in human, we see that the same L1PA3 element was truncated from 50% of length to 15%, and the Alu element (AluJb) was inserted just at the location of CNV. However, only 62% of Alu length remained in the sequence. The transposable elements activity can also be seen in the promoter area of the gene SORD. The remnants of retrovirus (HERV9, 50% of length) are present in the promoter region of three copies of SORD except the active human copy (first alignment row). Also, full length L1 element (L1PA6) that was most likely inserted in the retrovirus, is observed upstream of the active copy of the gene SORD, and the truncated copy of this element (65% of length) remained upstream of the SORD pseudogene copy. **Figure S5.** MAPK signaling pathway. *A. CNV-gene frequency heatmap for MAPK signaling pathway.* Rows correspond to CNV-gene pairs and columns correspond to three Hapmap populations: YRI, CEU and ASN. The values of the heatmap are CNV polymorphism frequency (see Methods). *B. Schematic representation of a fragment of MAPK signaling pathway (adopted from KEGG).* Highlighted in orange are the gene families, CACN and RASGRP, whose genes have CNVs with significant gene expression associations (P-value<=0.01) and evidence for population differentiation. Examples of gene variant associations for three genes from CACN family, CACNG2, CACNG6 and CACNG7, are given in separate boxes. Example of gene variant association for RASGRP family is given for RASGRP4 gene. **Figure S6.** Tuning Effect of Pathway Evolution. Different color and shape correspond to different enzymes. Increase in the concentration level of one enzyme (here green) can induce changes in the concentration levels of the linked enzymes (here blue and red). In the process of evolution, it can lead to the recruitment of enzymes that perform better functions, and as a result, create a new pathway. Click here for file

Additional file 4 **Table S2.** Complete list of 4978 CNVs with Fst calculated for each population pair. Click here for file

Additional file 5 **Table S3.** List of pathways enriched for population differentiated CNV-gene pairs. Click here for file

Additional file 6 **Table S4.** List of unique and shared pathways for Venn Diagram. Click here for file

Additional file 7 **Table S5.** List of unique and shared genes for Venn Diagram of Figure 4B. Click here for file

Additional file 8 **Table S7.** Enrichment analysis for the functional CNVs for CEU and YRI. Click here for file

Additional file 9 **Table S6.** List of CNVs that showed significant association with gene expression levels annotated with population differentiation statistics, pathways information and annotation on overlap with enhancers. Click here for file

## References

[B1] Iafrate AJ Feuk L Rivera MN Listewnik ML Donahoe PK Qi Y Scherer SW Lee C Detection of large-scale variation in the human genome Nat Genet 2004 36 9 949 951 10.1038/ng1416 15286789

[B2] Sebat J Lakshmi B Troge J Alexander J Young J Lundin P Maner S Massa H Walker M Chi M Large-scale copy number polymorphism in the human genome Science 2004 305 5683 525 528 10.1126/science.1098918 15273396

[B3] Conrad DF Pinto D Redon R Feuk L Gokcumen O Zhang Y Aerts J Andrews TD Barnes C Campbell P Origins and functional impact of copy number variation in the human genome Nature 2010 464 7289 704 712 10.1038/nature08516 19812545 PMC3330748

[B4] Durbin RM Abecasis GR Altshuler DL Auton A Brooks LD Gibbs RA Hurles ME McVean GA A map of human genome variation from population-scale sequencing Nature 2010 467 7319 1061 1073 10.1038/nature09534 20981092 PMC3042601

[B5] Mills RE Walter K Stewart C Handsaker RE Chen K Alkan C Abyzov A Yoon SC Ye K Cheetham RK Mapping copy number variation by population-scale genome sequencing Nature 2011 470 7332 59 65 10.1038/nature09708 21293372 PMC3077050

[B6] Fanciulli M Petretto E Aitman TJ Gene copy number variation and common human disease Clin Genet 2010 77 3 201 213 10.1111/j.1399-0004.2009.01342.x 20002459

[B7] Voight BF Kudaravalli S Wen X Pritchard JK A map of recent positive selection in the human genome PLoS Biol 2006 4 3 e72 10.1371/journal.pbio.0040072 16494531 PMC1382018

[B8] McCarroll SA Huett A Kuballa P Chilewski SD Landry A Goyette P Zody MC Hall JL Brant SR Cho JH Deletion polymorphism upstream of IRGM associated with altered IRGM expression and Crohn’s disease Nat Genet 2008 40 9 1107 1112 10.1038/ng.215 19165925 PMC2731799

[B9] Yang TL Chen XD Guo Y Lei SF Wang JT Zhou Q Pan F Chen Y Zhang ZX Dong SS Genome-wide copy-number-variation study identified a susceptibility gene, UGT2B17, for osteoporosis Am J Hum Genet 2008 83 6 663 674 10.1016/j.ajhg.2008.10.006 18992858 PMC2667994

[B10] Willer CJ Speliotes EK Loos RJ Li S Lindgren CM Heid IM Berndt SI Elliott AL Jackson AU Lamina C Six new loci associated with body mass index highlight a neuronal influence on body weight regulation Nat Genet 2009 41 1 25 34 10.1038/ng.287 19079261 PMC2695662

[B11] Gonzalez E Kulkarni H Bolivar H Mangano A Sanchez R Catano G Nibbs RJ Freedman BI Quinones MP Bamshad MJ The influence of CCL3L1 gene-containing segmental duplications on HIV-1/AIDS susceptibility Science 2005 307 5714 1434 1440 10.1126/science.1101160 15637236

[B12] Pinto D Pagnamenta AT Klei L Anney R Merico D Regan R Conroy J Magalhaes TR Correia C Abrahams BS Functional impact of global rare copy number variation in autism spectrum disorders Nature 2010 466 7304 368 372 10.1038/nature09146 20531469 PMC3021798

[B13] Sebat J Lakshmi B Malhotra D Troge J Lese-Martin C Walsh T Yamrom B Yoon S Krasnitz A Kendall J Strong association of de novo copy number mutations with autism Science 2007 316 5823 445 449 10.1126/science.1138659 17363630 PMC2993504

[B14] McCarthy SE Makarov V Kirov G Addington AM McClellan J Yoon S Perkins DO Dickel DE Kusenda M Krastoshevsky O Microduplications of 16p11.2 are associated with schizophrenia Nat Genet 2009 41 11 1223 1227 10.1038/ng.474 19855392 PMC2951180

[B15] Stefansson H Rujescu D Cichon S Pietilainen OP Ingason A Steinberg S Fossdal R Sigurdsson E Sigmundsson T Buizer-Voskamp JE Large recurrent microdeletions associated with schizophrenia Nature 2008 455 7210 232 236 10.1038/nature07229 18668039 PMC2687075

[B16] Demichelis F Setlur SR Banerjee S Chakravarty D Chen JY Chen CX Huang J Beltran H Oldridge DA Kitabayashi N Identification of functionally active, low frequency copy number variants at 15q21.3 and 12q21.31 associated with prostate cancer risk Proc Natl Acad Sci USA 2012 109 17 6686 6691 10.1073/pnas.1117405109 22496589 PMC3340033

[B17] Diskin SJ Hou C Glessner JT Attiyeh EF Laudenslager M Bosse K Cole K Mosse YP Wood A Lynch JE Copy number variation at 1q21.1 associated with neuroblastoma Nature 2009 459 7249 987 991 10.1038/nature08035 19536264 PMC2755253

[B18] Montgomery SB Sammeth M Gutierrez-Arcelus M Lach RP Ingle C Nisbett J Guigo R Dermitzakis ET Transcriptome genetics using second generation sequencing in a Caucasian population Nature 2010 464 7289 773 777 10.1038/nature08903 20220756 PMC3836232

[B19] Pickrell JK Marioni JC Pai AA Degner JF Engelhardt BE Nkadori E Veyrieras JB Stephens M Gilad Y Pritchard JK Understanding mechanisms underlying human gene expression variation with RNA sequencing Nature 2010 464 7289 768 772 10.1038/nature08872 20220758 PMC3089435

[B20] Stranger BE Forrest MS Dunning M Ingle CE Beazley C Thorne N Redon R Bird CP de Grassi A Lee C Relative impact of nucleotide and copy number variation on gene expression phenotypes Science 2007 315 5813 848 853 10.1126/science.1136678 17289997 PMC2665772

[B21] Banerjee S Oldridge D Poptsova M Hussain W Chakravarty D Demichelis F A computational framework discovers New copy number variants with functional importance PLoS One 2011 6 3 e17539 10.1371/journal.pone.0017539 21479260 PMC3066184

[B22] Schlattl A Anders S Waszak SM Huber W Korbel JO Relating CNVs to transcriptome data at fine-resolution: Assessment of the effect of variant size, type, and overlap with functional regions Genome Res 2011 21 12 2004 2013 10.1101/gr.122614.111 21862627 PMC3227091

[B23] Akey JM Constructing genomic maps of positive selection in humans: where do we go from here? Genome Res 2009 19 5 711 722 10.1101/gr.086652.108 19411596 PMC3647533

[B24] Andres AM Hubisz MJ Indap A Torgerson DG Degenhardt JD Boyko AR Gutenkunst RN White TJ Green ED Bustamante CD Targets of balancing selection in the human genome Mol Biol Evol 2009 26 12 2755 2764 10.1093/molbev/msp190 19713326 PMC2782326

[B25] Hernandez RD Kelley JL Elyashiv E Melton SC Auton A McVean G Sella G Przeworski M Classic selective sweeps were rare in recent human evolution Science 2011 331 6019 920 924 10.1126/science.1198878 21330547 PMC3669691

[B26] Kelley JL Swanson WJ Positive selection in the human genome: from genome scans to biological significance Annu Rev Genomics Hum Genet 2008 9 143 160 10.1146/annurev.genom.9.081307.164411 18505377

[B27] Kato M Kawaguchi T Ishikawa S Umeda T Nakamichi R Shapero MH Jones KW Nakamura Y Aburatani H Tsunoda T Population-genetic nature of copy number variations in the human genome Hum Mol Genet 2010 19 5 761 773 10.1093/hmg/ddp541 19966329 PMC2816609

[B28] Jakobsson M Scholz SW Scheet P Gibbs JR VanLiere JM Fung HC Szpiech ZA Degnan JH Wang K Guerreiro R Genotype, haplotype and copy-number variation in worldwide human populations Nature 2008 451 7181 998 1003 10.1038/nature06742 18288195

[B29] Cavalli-Sforza LL Menozzi P Piazza A The history and geography of human genes 1996 Princeton, N.J.: Princeton University Press

[B30] Perry GH Dominy NJ Claw KG Lee AS Fiegler H Redon R Werner J Villanea FA Mountain JL Misra R Diet and the evolution of human amylase gene copy number variation Nat Genet 2007 39 10 1256 1260 10.1038/ng2123 17828263 PMC2377015

[B31] Xue Y Sun D Daly A Yang F Zhou X Zhao M Huang N Zerjal T Lee C Carter NP Adaptive evolution of UGT2B17 copy-number variation Am J Hum Genet 2008 83 3 337 346 10.1016/j.ajhg.2008.08.004 18760392 PMC2556428

[B32] Niu AL Wang YQ Zhang H Liao CH Wang JK Zhang R Che J Su B Rapid evolution and copy number variation of primate RHOXF2, an X-linked homeobox gene involved in male reproduction and possibly brain function BMC Evol Biol 2011 11 298 10.1186/1471-2148-11-298 21988730 PMC3214919

[B33] King MC Wilson AC Evolution at two levels in humans and chimpanzees Science 1975 188 4184 107 116 10.1126/science.1090005 1090005

[B34] Blekhman R Marioni JC Zumbo P Stephens M Gilad Y Sex-specific and lineage-specific alternative splicing in primates Genome Res 2010 20 2 180 189 10.1101/gr.099226.109 20009012 PMC2813474

[B35] Flowers JM Sezgin E Kumagai S Duvernell DD Matzkin LM Schmidt PS Eanes WF Adaptive evolution of metabolic pathways in Drosophila Mol Biol Evol 2007 24 6 1347 1354 10.1093/molbev/msm057 17379620

[B36] Eanes WF Molecular population genetics and selection in the glycolytic pathway J Exp Biol 2011 214 Pt 2 165 171 21177937 10.1242/jeb.046458 PMC3183487

[B37] Horowitz NH On the evolution of biochemical synthesis Proc Natl Acad Sci USA 1945 31 153 157 10.1073/pnas.31.6.153 16578152 PMC1078786

[B38] Ycas M On earlier states of the biochemical system J Theor Biol 1974 44 1 145 160 10.1016/S0022-5193(74)80035-4 4207200

[B39] Lazcano A Miller SL On the origin of metabolic pathways J Mol Evol 1999 49 4 424 431 10.1007/PL00006565 10486000

[B40] Kuhn RM Karolchik D Zweig AS Wang T Smith KE Rosenbloom KR Rhead B Raney BJ Pohl A Pheasant M The UCSC genome browser database: update 2009 Nucleic Acids Res 2009 37 Database issue D755 761 18996895 10.1093/nar/gkn875 PMC2686463

[B41] Kanehisa M Goto S KEGG: kyoto encyclopedia of genes and genomes Nucleic Acids Res 2000 28 1 27 30 10.1093/nar/28.1.27 10592173 PMC102409

[B42] Weir BS Hill WG Estimating F-statistics Annu Rev Genet 2002 36 721 750 10.1146/annurev.genet.36.050802.093940 12359738

[B43] McCarroll SA Kuruvilla FG Korn JM Cawley S Nemesh J Wysoker A Shapero MH de Bakker PI Maller JB Kirby A Integrated detection and population-genetic analysis of SNPs and copy number variation Nat Genet 2008 40 10 1166 1174 10.1038/ng.238 18776908

[B44] Habegger L Sboner A Gianoulis TA Rozowsky J Agarwal A Snyder M Gerstein M RSEQtools: a modular framework to analyze RNA-Seq data using compact, anonymized data summaries Bioinformatics 2011 27 2 281 283 10.1093/bioinformatics/btq643 21134889 PMC3018817

[B45] Redon R Ishikawa S Fitch KR Feuk L Perry GH Andrews TD Fiegler H Shapero MH Carson AR Chen W Global variation in copy number in the human genome Nature 2006 444 7118 444 454 10.1038/nature05329 17122850 PMC2669898

[B46] Holsinger KE Weir BS Genetics in geographically structured populations: defining, estimating and interpreting F(ST) Nat Rev Genet 2009 10 9 639 650 10.1038/nrg2611 19687804 PMC4687486

[B47] Baye TM Wilke RA Olivier M Genomic and geographic distribution of private SNPs and pathways in human populations Per Med 2009 6 6 623 641 10.2217/pme.09.54 20352079 PMC2843937

[B48] Kudaravalli S Veyrieras JB Stranger BE Dermitzakis ET Pritchard JK Gene expression levels are a target of recent natural selection in the human genome Mol Biol Evol 2009 26 3 649 658 19091723 10.1093/molbev/msn289 PMC2767089

[B49] Yamit-Hezi A Nir S Wolstein O Dikstein R Interaction of TAFII105 with selected p65/RelA dimers is associated with activation of subset of NF-kappa B genes J Biol Chem 2000 275 24 18180 18187 10.1074/jbc.275.24.18180 10849440

[B50] Romani B Engelbrecht S Glashoff RH Antiviral roles of APOBEC proteins against HIV-1 and suppression by Vif Arch Virol 2009 154 10 1579 1588 10.1007/s00705-009-0481-y 19669862

[B51] Bogerd HP Wiegand HL Hulme AE Garcia-Perez JL O’Shea KS Moran JV Cullen BR Cellular inhibitors of long interspersed element 1 and Alu retrotransposition Proc Natl Acad Sci USA 2006 103 23 8780 8785 10.1073/pnas.0603313103 16728505 PMC1482655

[B52] Burgess DL Gefrides LA Foreman PJ Noebels JL A cluster of three novel Ca2+ channel gamma subunit genes on chromosome 19q13.4: evolution and expression profile of the gamma subunit gene family Genomics 2001 71 3 339 350 10.1006/geno.2000.6440 11170751

[B53] Freilich S Goldovsky L Ouzounis CA Thornton JM Metabolic innovations towards the human lineage BMC Evol Biol 2008 8 247 10.1186/1471-2148-8-247 18782449 PMC2553087

[B54] Locke DP Hillier LW Warren WC Worley KC Nazareth LV Muzny DM Yang SP Wang Z Chinwalla AT Minx P Comparative and demographic analysis of orang-utan genomes Nature 2011 469 7331 529 533 10.1038/nature09687 21270892 PMC3060778

[B55] Lanktree M Hegele RA Copy number variation in metabolic phenotypes Cytogenet Genome Res 2008 123 1–4 169 175 19287152 10.1159/000184705

[B56] Kim PM Korbel JO Gerstein MB Positive selection at the protein network periphery: evaluation in terms of structural constraints and cellular context Proc Natl Acad Sci USA 2007 104 51 20274 20279 10.1073/pnas.0710183104 18077332 PMC2154421

[B57] Sabeti PC Schaffner SF Fry B Lohmueller J Varilly P Shamovsky O Palma A Mikkelsen TS Altshuler D Lander ES Positive natural selection in the human lineage Science 2006 312 5780 1614 1620 10.1126/science.1124309 16778047

[B58] Barreiro LB Laval G Quach H Patin E Quintana-Murci L Natural selection has driven population differentiation in modern humans Nat Genet 2008 40 3 340 345 10.1038/ng.78 18246066

[B59] Nguyen DQ Webber C Hehir-Kwa J Pfundt R Veltman J Ponting CP Reduced purifying selection prevails over positive selection in human copy number variant evolution Genome Res 2008 18 11 1711 1723 10.1101/gr.077289.108 18687881 PMC2577867

[B60] Schuster-Bockler B Conrad D Bateman A Dosage sensitivity shapes the evolution of copy-number varied regions PLoS One 2010 5 3 e9474 10.1371/journal.pone.0009474 20224824 PMC2835737

[B61] Yamit-Hezi A Dikstein R TAFII105 mediates activation of anti-apoptotic genes by NF-kappaB EMBO J 1998 17 17 5161 5169 10.1093/emboj/17.17.5161 9724652 PMC1170844

[B62] Nelson DE Ihekwaba AE Elliott M Johnson JR Gibney CA Foreman BE Nelson G See V Horton CA Spiller DG Oscillations in NF-kappaB signaling control the dynamics of gene expression Science 2004 306 5696 704 708 10.1126/science.1099962 15499023

[B63] Qiao L Nachbar RB Kevrekidis IG Shvartsman SY Bistability and oscillations in the Huang-Ferrell model of MAPK signaling PLoS Comput Biol 2007 3 9 1819 1826 17907797 10.1371/journal.pcbi.0030184 PMC1994985

[B64] Veitia RA Gene dosage balance in cellular pathways: implications for dominance and gene duplicability Genetics 2004 168 1 569 574 10.1534/genetics.104.029785 15454568 PMC1448121

[B65] Veitia RA Gene dosage balance: deletions, duplications and dominance Trends Genet 2005 21 1 33 35 10.1016/j.tig.2004.11.002 15680512

[B66] Veitia RA Nonlinear effects in macromolecular assembly and dosage sensitivity J Theor Biol 2003 220 1 19 25 10.1006/jtbi.2003.3105 12453447

