# Supplementary material for: Impact of constitutional copy number variants on biological pathway evolution
Source: BMC Evol Biol. 2013 Jan 23;13:19. doi: 10.1186/1471-2148-13-19 (PMC3563492; doi:10.1186/1471-2148-13-19)
Supplement: Additional file 1 — Supplementary Material. [file 1471-2148-13-19-S1.doc]

**Supplementary Material**

**Impact of Constitutional Copy Number Variants on Biological Pathway Evolution**

Maria Poptsova, Samprit Banerjee, Omer Gokcumen, Mark A Rubin, Francesca Demichelis

**Evolutionary history of *UGT2B17***………………………………………………………………1

**Ancestral analysis of the area around gene *SORD***…………………………………………….2

**Enrichment of CNVs in cancer-related gene families**…………………………………………2

**Examples of the enriched pathways of metabolic class (Sulfur metabolism and**

**Glutathion metabolism)** ……………………………………………………………………..….3

**MAPK signaling pathway** ..…………………………………………………………………….4

**Pathway evolution models** ..……………………………………………………………………..5

**References**…………………………………………………………………………...……………6

**Evolutionary history of *UGT2B17***

*UGT2B17* gene exists in different copies and shows high population differentiation**.** Copy number variation of *UGT2B17* is the result of a one-copy deletion within a region that underwent segmental duplications. The deletion allele is highly predominant in the Asian population, the non-deleted allele is preserved in the majority of the African population, while both alleles are present in the European population at similar frequencies. This data demonstrates a strong positive selection of the deleted allele in Asians with only moderate selection in Europeans. However, the existence of both alleles in the African population for more than 2.4 million years is indicative of long-term balancing selection .

**Ancestral analysis of the area around gene SORD**

The chimpanzee genome (panTro2) was downloaded from UCSC Genome Browser. The corresponding coordinates for the SORD gene (differentiated in all three populations) in chimpanzee genome were obtained with the liftOver tool. Homology search of the CNV and SORD regions was performed with BLAT. Alignment and visualization was done using Mauve . Set of chimpanzee CNVs was retrieved from .

Though this CNV is reported as a gain, ancestral state analysis showed that it is actually a loss. The detailed analysis of the corresponding regions in human reference and chimpanzee genomes (see **Supplementary** **Figure 4**) revealed that a whole region (approximately 80 kb) including the gene SORD underwent an inverted duplication and the second copy of the gene SORD most likely became a pseudogene. The observed CNV resulted from a loss of ~ 5kB DNA fragment from the second copy of the gene SORD as a result of Alu insertion at the site of L1 element (see **Supplementary** **Figure 4**). The deletion allele was favored in the European population (frequency of 84% for CN=2, and 16% for CN=3), while the Yorubans maintain the undeleted allele as a majority (frequency of 65% for CN=4; frequency of 32% for CN=3 and frequency of 3% for CN=2); and the Asians have intermediate frequency distribution (frequency of 34% for CN=2, frequency of 50% for CN=3 and frequency of 16% for CN=4). The duplication-deletion scenario of this CNV inside gene SORD is similar to UGTB17 CNV formation with the difference that for UGT2B17 the deletion allele was favored in Asian population while the deletion allele for CNV in SORD was favored by the Europeans.

**Enrichment of CNVs in cancer-related gene families.**

Among the 32 pathways enriched for differentiated CNV-gene pairs, we found the Role of ERBB2 in signal transduction and oncology pathway among the top ranked. The impact of germline CNVs on cancerogenesis is not understood, but it has been shown that 40% of cancer-related genes overlap with common CNVs . Our results indicate significant enrichment of the proportions of overlapping CNV-gene pairs in cancer-related families. We evaluated that 30% of the genes reported in the Mitelman translocation database (1.94 fold, P-value=2.2E-16), 30% of all tyrosine kinases known to be mutated in cancer (1.88 fold, P-value= 0.000277), and around 25% of keratins known to have increased production in cancer (1.46 fold, P-value= 0.01853) overlap with common CNVs queried in this study. In the Role of ERBB2 in signal transduction and oncology pathway, three genes showed population differentiation: ERBB4 (receptor tyrosine-protein kinase erbB-4), STAT3 (Signal transducer and activator of transcription 3) and EGFR (epidermal growth factor receptor). Even though all three genes have been extensively studied in relation to cancer, their differential effect across populations, including differential disease susceptibility, is still to be investigated. Another example of pathway enriched for significantly differentiated CNV-gene pairs is the Androgen and estrogen metabolism with the genes AKR1C4 (Aldo-keto reductase family 1 member C4), UGT2B15 and UDP-glucuronosyltransferase 2B15) and UGT2B17 (UDP-glucuronosyltransferase 2B17). UGT2B17 has been studied in the context of population differentiation , association with osteoporosis , endometrial cancer , and prostate cancer . From the signaling pathways, the NF-kB signaling pathway contains the IKBKB gene (inhibitor of kappa light polypeptide gene enhancer in B-cells, kinase beta) that, as we showed, is highly differentiated both between CEU and YRI and YRI and ASN. This gene is an upstream activator of NFkB, nuclear factor kB, which has an increase activation in many cancers . Even though all these genes have been extensively studied in relation to cancer, their differential effect across populations, including differential disease susceptibility, is still to be investigated.

**Examples of the enriched pathways of metabolic class (Sulfur metabolism and Glutathion metabolism)**

Within the enriched Sulfur metabolism pathway, PAPSS2 (3'-phosphoadenosine 5'-phosphosulfate synthase 2) gene was reported in a 4-gene panel that predicts survival of patients with resected adenocarcinoma of the esophagus, junction, and gastric cardia , and the deficiency in this gene resulted in osteochondrodysplasia . From the same pathway, SULT1A1, which encodes sulfotransferase isoform 1A1, is surrounded by several variants in the coding and promoter regions significantly associated with enzymatic activity . The Glutathion metabolism is another example of enriched metabolic class pathway. Among the genes overlapping with CNVs, there are members of the GST (glutathione S-transferase) family. These well-studied genes have shown polymorphism and association with various cancers and schizophrenia .

**MAPK signaling pathway**

Data plots showing the associations between CNV states and gene expression for genes from the Mitogen-activated protein kinase (MAPK) signaling pathway are presented in **Supplementary Figure 5**. The MAPK signaling pathway is a canonical pathway that controls cell response to chemical and physical stresses and controls cell survival and adaptation . A portion of the MAPK pathway (adapted from KEGG) is presented in **Supplementary Figure 5B**. Highlighted in orange are the gene families RASGRP (includes genes RASGRP1-4) and CACN (includes genes CACNG1-8, CACNA1A-H, CACNA1S, CACNA2D1-4, CACNB1-4) whose members were detected as altered. CACN is a gene family involved in calcium channel formation, where CACNG is a family of gamma subunits, composing calcium channels that are known to modulate their biophysical properties . Three members of the gamma subunit family, CACNG2, CACNG6 and CACNG7, showed CNV associated transcript levels and revealed differentiation in population. Elevated expression levels of CACNG2 were observed in individuals with CN=2 and that of CACNG6 and CACNG7 in individuals with CN=4. The population differentiation is higher for the CNV affecting CACNG2, compare to other gamma subunits, with frequencies of 42%, 99% and 20% in CEU, YRI, and ASN populations, respectively. Association was also detected between a deletion CNV, observed only in CEU population (22% frequency), and the RASGRP4 gene that belongs to the RAS guanyl nucleotide-releasing protein (RASGRP) gene family that activates MAP kinase cascade. A boxplot showing the inverse relationship between the copy number states and RASGRP4 transcript levels is presented in **Supplementary Figure 5B**. These results indicate the recent European specific changes in MAPK signaling pathway, and also suggest hitchhiking effect, where changes in CACNG2 expression potentially influence changes in RASGRP4 expression. All 8 gamma subunits have been shown to co-express in adult and fetal brains, and are considered to be important for regulating calcium channels . This is an example of CNVs affecting expression of the genes that are located at the beginning of the pathway chain, CNVs affecting expression of downstream gene, and at the same time, showing population differentiation.

**Pathway Evolution Models**

Pathway is a set of reactions that are grouped into causal chains. Existing models of pathway evolution, such as Horowitz retrograde model , chemistry-driven patchwork model , and some others (see for reviews on pathway evolution theories), consider major modifications to a pathway chain, such as recruitment of a new enzyme or a whole pathway duplication, that eventually lead to creation of a new pathway. We argue that smaller changes can also play an important role, through gene product concentration changes that might adjust one or more pathway nodes while the pathway structure remains unchanged.

We investigated evolutionary patterns of biological pathways by tracing evolutionary signatures of individual genes constituting the pathway. The latter are being inferred both from the distribution of CNVs in the individual genome (location in the gene areas) and from the distribution of CNV frequencies across populations (signatures of selection). Our results suggest an evolutionary importance of small changes in the pathway structure, such as perturbation of a singular pathway node by adjusting gene expression levels that later become fixed in different populations. It is the fixation of different gene expression levels for the same pathway that we called here as the “tuning effect” of pathway evolution. It essentially states that though a pathway chain remains intact as a whole (no new enzymes or metabolites are added), the evolutionary selected changes in transcription level of some genes constituting the pathway “tune” the pathway into more favorable state.

This “Tuning effect” of pathway evolution is schematically presented in **Supplementary** **Figure 6**. Enzyme recruitment was suggested to be the main driving force for the evolution of new pathway , and here we hypothesize that a long-term potential outcome of the proposed tuning effect could be a creation of a new pathway.

**References**

1. Xue Y, Sun D, Daly A, Yang F, Zhou X, Zhao M, Huang N, Zerjal T, Lee C, Carter NP *et al*: **Adaptive evolution of UGT2B17 copy-number variation**. *Am J Hum Genet* 2008, **83**(3):337-346.

2. Darling AC, Mau B, Blattner FR, Perna NT: **Mauve: multiple alignment of conserved genomic sequence with rearrangements**. *Genome Res* 2004, **14**(7):1394-1403.

3. Perry GH, Yang F, Marques-Bonet T, Murphy C, Fitzgerald T, Lee AS, Hyland C, Stone AC, Hurles ME, Tyler-Smith C *et al*: **Copy number variation and evolution in humans and chimpanzees**. *Genome Res* 2008, **18**(11):1698-1710.

4. Shlien A, Malkin D: **Copy number variations and cancer**. *Genome Med* 2009, **1**(6):62.

5. **Mitelman Database of Chromosome Aberrations and Gene Fusions in Cancer**

6. Yang TL, Chen XD, Guo Y, Lei SF, Wang JT, Zhou Q, Pan F, Chen Y, Zhang ZX, Dong SS *et al*: **Genome-wide copy-number-variation study identified a susceptibility gene, UGT2B17, for osteoporosis**. *Am J Hum Genet* 2008, **83**(6):663-674.

7. Hirata H, Hinoda Y, Zaman MS, Chen Y, Ueno K, Majid S, Tripsas C, Rubin M, Chen LM, Dahiya R: **Function of UDP-glucuronosyltransferase 2B17 (UGT2B17) is involved in endometrial cancer**. *Carcinogenesis*, **31**(9):1620-1626.

8. Demichelis F, Setlur SR, Banerjee S, Chakravarty D, Chen JY, Chen CX, Huang J, Beltran H, Oldridge DA, Kitabayashi N *et al*: **Identification of functionally active, low frequency copy number variants at 15q21.3 and 12q21.31 associated with prostate cancer risk**. *Proc Natl Acad Sci U S A* 2012, **109**(17):6686-6691.

9. Gyrd-Hansen M, Meier P: **IAPs: from caspase inhibitors to modulators of NF-kappaB, inflammation and cancer**. *Nat Rev Cancer* 2010, **10**(8):561-574.

10. Peters CJ, Rees JR, Hardwick RH, Hardwick JS, Vowler SL, Ong CA, Zhang C, Save V, O'Donovan M, Rassl D *et al*: **A 4-gene signature predicts survival of patients with resected adenocarcinoma of the esophagus, junction, and gastric cardia**. *Gastroenterology*, **139**(6):1995-2004 e1915.

11. Venkatachalam KV: **Human 3'-phosphoadenosine 5'-phosphosulfate (PAPS) synthase: biochemistry, molecular biology and genetic deficiency**. *IUBMB Life* 2003, **55**(1):1-11.

12. Yu X, Dhakal IB, Beggs M, Edavana VK, Williams S, Zhang X, Mercer K, Ning B, Lang NP, Kadlubar FF *et al*: **Functional genetic variants in the 3'-untranslated region of sulfotransferase isoform 1A1 (SULT1A1) and their effect on enzymatic activity**. *Toxicol Sci*, **118**(2):391-403.

13. Ueda M, Toji E, Nunobiki O, Sato N, Izuma S, Torii K, Okamoto Y, Noda S: **Germline polymorphisms of glutathione-S-transferase GSTM1, GSTT1 and p53 codon 72 in cervical carcinogenesis**. *Hum Cell* 2010, **23**(4):119-125.

14. Watanabe Y, Nunokawa A, Kaneko N, Someya T: **A case-control study and meta-analysis of association between a common copy number variation of the glutathione S-transferase mu 1 (GSTM1) gene and schizophrenia**. *Schizophr Res*, **124**(1-3):236-237.

15. Chang L, Karin M: **Mammalian MAP kinase signalling cascades**. *Nature* 2001, **410**(6824):37-40.

16. Burgess DL, Gefrides LA, Foreman PJ, Noebels JL: **A cluster of three novel Ca2+ channel gamma subunit genes on chromosome 19q13.4: evolution and expression profile of the gamma subunit gene family**. *Genomics* 2001, **71**(3):339-350.

17. Horowitz NH: **On the evolution of biochemical synthesis**. *Proc Natl Acad Sci U S A* 1945, **31**:153-157.

18. Ycas M: **On earlier states of the biochemical system**. *J Theor Biol* 1974, **44**(1):145-160.

19. Lazcano A, Miller SL: **On the origin of metabolic pathways**. *J Mol Evol* 1999, **49**(4):424-431.

20. Schmidt S, Sunyaev S, Bork P, Dandekar T: **Metabolites: a helping hand for pathway evolution?** *Trends Biochem Sci* 2003, **28**(6):336-341.
