# Supplementary material for: Impact of constitutional copy number variants on biological pathway evolution
Source: BMC Evol Biol. 2013 Jan 23;13:19. doi: 10.1186/1471-2148-13-19 (PMC3563492; doi:10.1186/1471-2148-13-19)
Supplement: Additional file 3 — Figure S1. Distribution of depleted KEGG and Biocarta pathway classes. A. Distribution of depleted KEGG classes. Original distribution of pathway classes in KEGG database is given on the left. Distribution of the KEGG classes depleted in CNVs that were obtained with the size-dependent enrichment analysis is given on the right. B. Distribution of the depleted Biocarta categories. Original distribution of pathway categories in Biocarta database is given on the left. Distribution of the Biocarta categories depleted in CNVs that were obtained with the size-dependent enrichment analysis is given on the right. Figure S2. Histogram of CNV frequency differences in three population pairs: CEU-YRI, CEU-ASN and ASN_YRI. CNV frequency is a frequency of polymorphism and calculated as described in the Methods. Frequency differences are given in absolute values. Figure S3. CNV-gene frequency heatmaps for 368 pathways. Heatmaps are constructed for CNV-gene pairs with 10 kb flanks. Figure S4. Rearrangements around SORD gene area in human and chimpanzee. Figure shows Mauve block alignment of four homologous regions in human and chimpanzee. First two regions are extracted from human reference genome (build hg18): chr15:43,080,000-43,163,000 and chr15:42,917,000-43,079,000, and the second two regions are taken from chimpanzee genomes (build panTro2): chr15:42,173,000-42,250,000 and chr15:41,950,000-42,030,000. The region of ~ 80 kb that includes the gene SORD underwent inverse duplication before the split of human and chimpanzee. The active copy of the gene SORD is encoded on the plus strand and is shown in the orange color. The copy of the gene SORD on the minus strand, that most likely became a pseudogene, is shown in the light orange. CNV resulted from a loss of a region in human genome from the inverted copy of the gene SORD (see empty box at the second alignment row). Analysis of the RepeatMasker annotation revealed that in the chimpanzee, the L1 element (L1PA3) is located right n [file 1471-2148-13-19-S3.pdf]

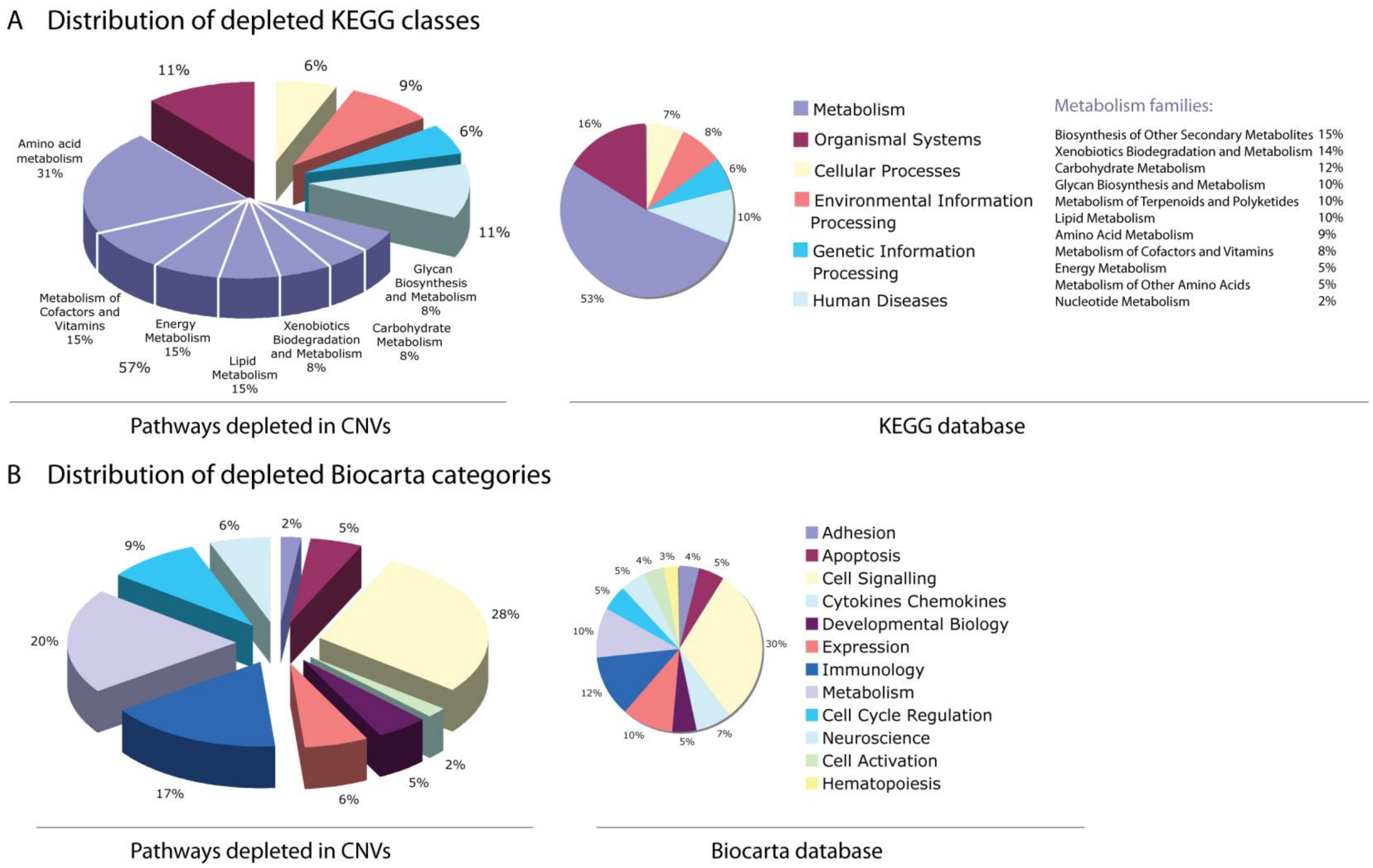


**Supplementary Figure 1. Distribution of depleted KEGG and Biocarta pathway classes.** *A. Distribution of depleted KEGG classes.* Original distribution of pathway classes in KEGG database is given on the left. Distribution of the KEGG classes depleted in CNVs that were obtained with the size-dependent enrichment analysis is given on the right. *B. Distribution of the depleted Biocarta categories.* Original distribution of pathway categories in Biocarta database is given on the left. Distribution of the Biocarta categories depleted in CNVs that were obtained with the size-dependent enrichment analysis is given on the right.

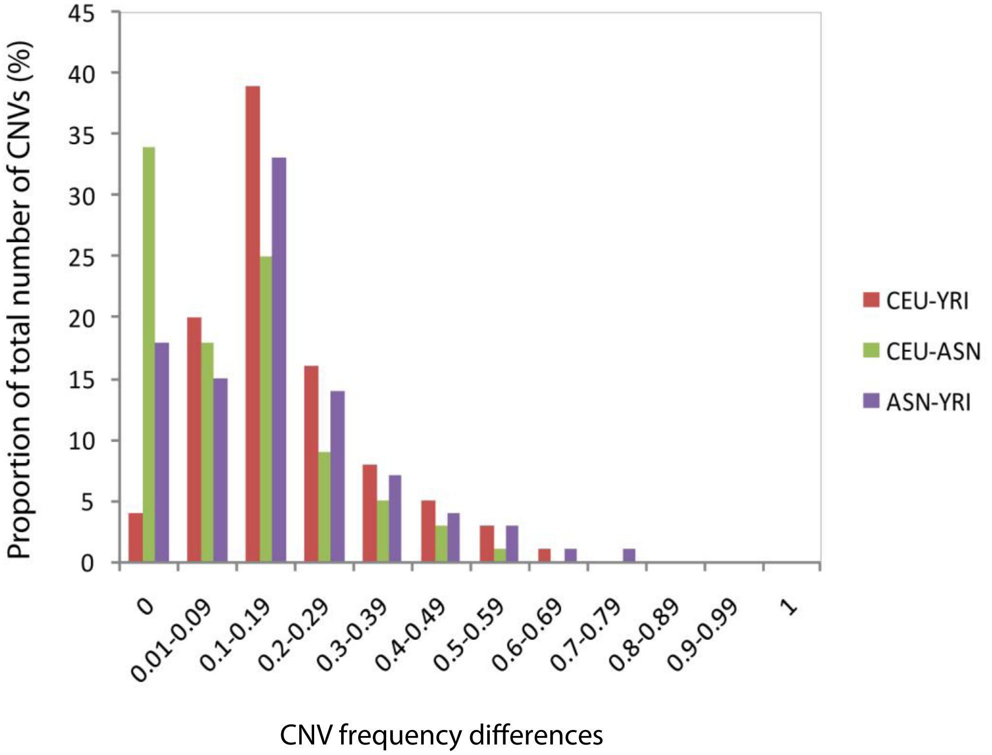


Supplementary Figure 2. Histogram of CNV frequency differences in three population pairs: CEU-YRI, CEU-ASN and ASN-YRI. CNV frequency is a frequency of polymorphism and calculated as described in the Methods. Frequency differences are given in absolute values.

**Supplementary Figure 3. CNV-gene frequency heatmaps for 368 pathways.** Heatmaps are constructed for CNV-gene pairs with 10kB flanks.

(next 368 pages)

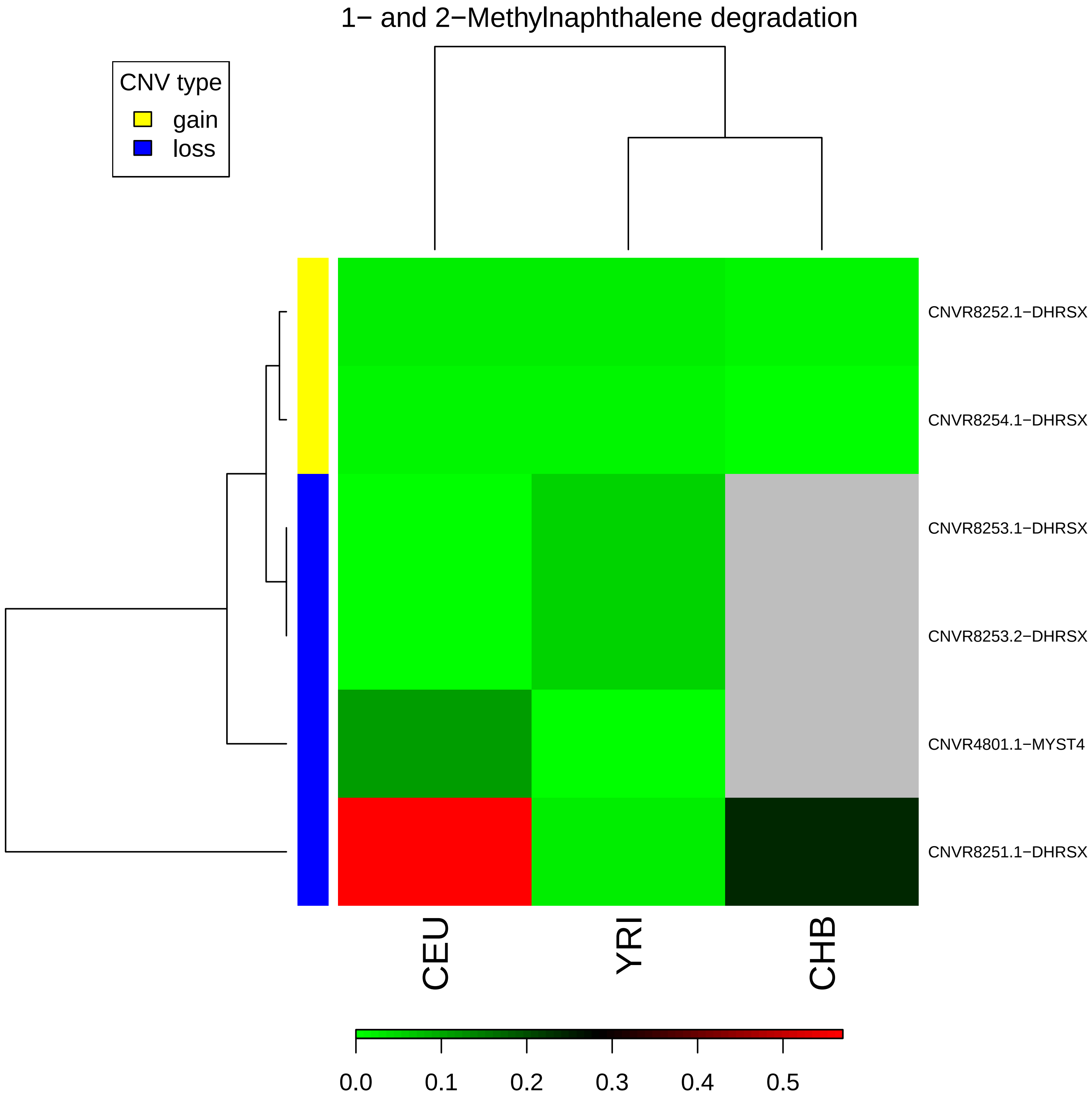

1− and 2−Methylnaphthalene degradation
CNV type
gain
loss
CNVR8252.1−DHRSX
CNVR8254.1−DHRSX
CNVR8253.1−DHRSX
CNVR8253.2−DHRSX
CNVR4801.1−MYST4
CNVR8251.1−DHRSX
CEU
YRI
CHB
0.0
0.1
0.2
0.3
0.4
0.5

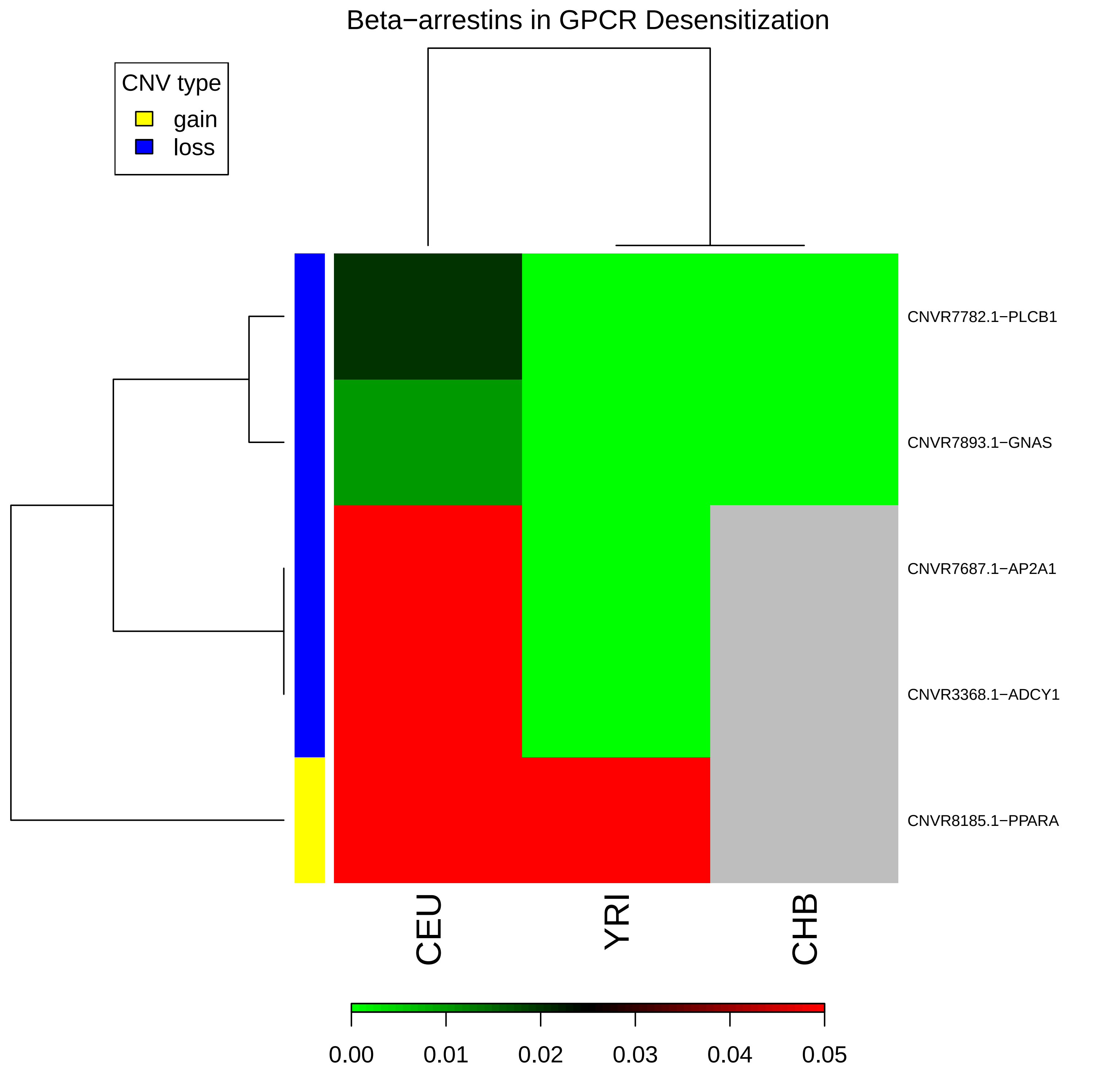

Beta−arrestins in GPCR Desensitization
CNV type
gain
loss
CNVR7782.1−PLCB1
CNVR7893.1−GNAS
CNVR7687.1−AP2A1
CNVR3368.1−ADCY1
CNVR8185.1−PPARA
CEU
YRI
CHB
0.00
0.01
0.02
0.03
0.04
0.05

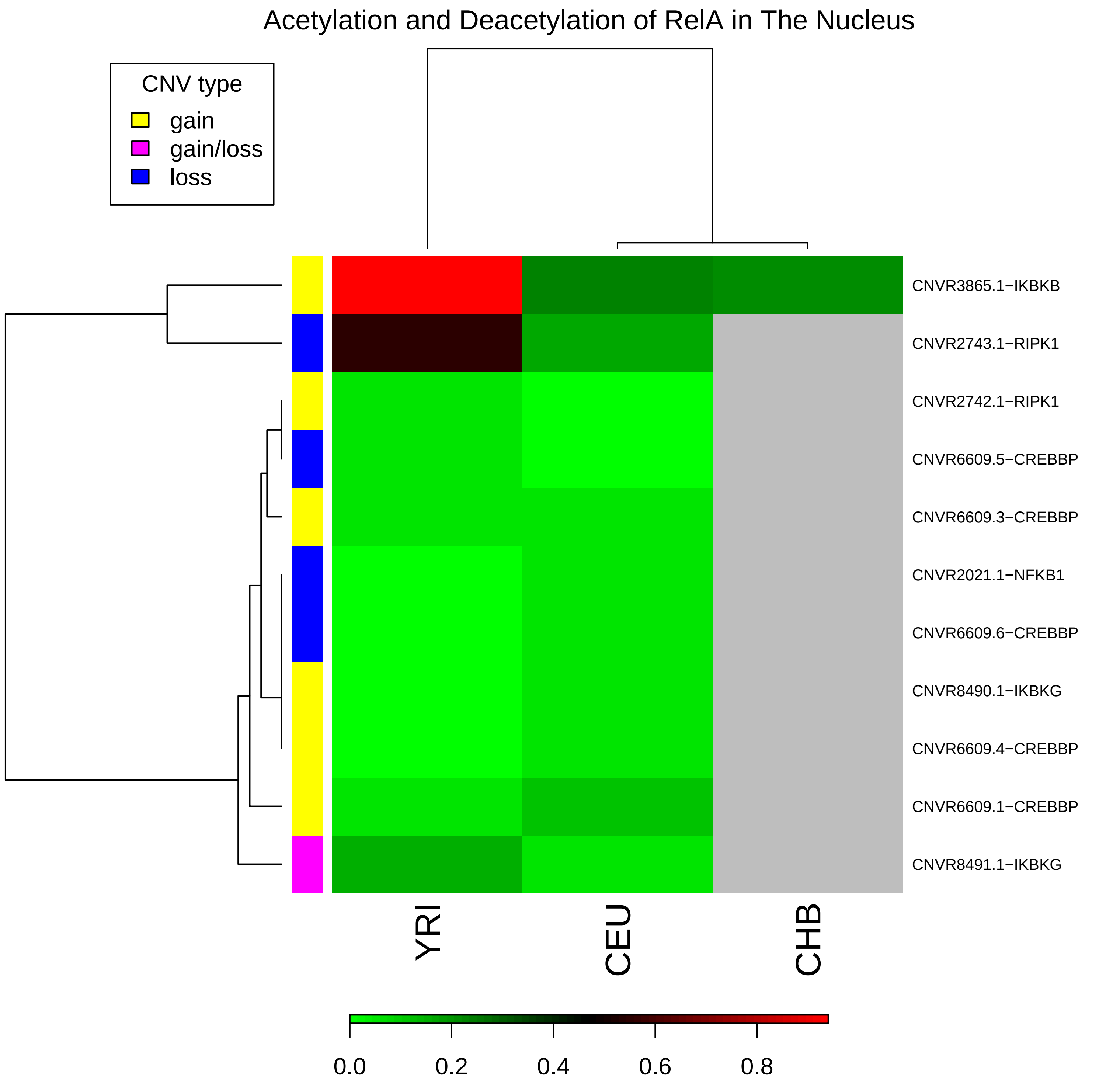
Acetylation and Deacetylation of RelA in The Nucleus
CNV type
gain
gain/loss
loss
CNVR3865.1−IKBKB
CNVR2743.1−RIPK1
CNVR2742.1−RIPK1
CNVR6609.5−CREBBP
CNVR6609.3−CREBBP
CNVR2021.1−NFKB1
CNVR6609.6−CREBBP
CNVR8490.1−IKBKG
CNVR6609.4−CREBBP
CNVR6609.1−CREBBP
CNVR8491.1−IKBKG
YRI
CEU
CHB
0.0
0.2
0.4
0.6
0.8

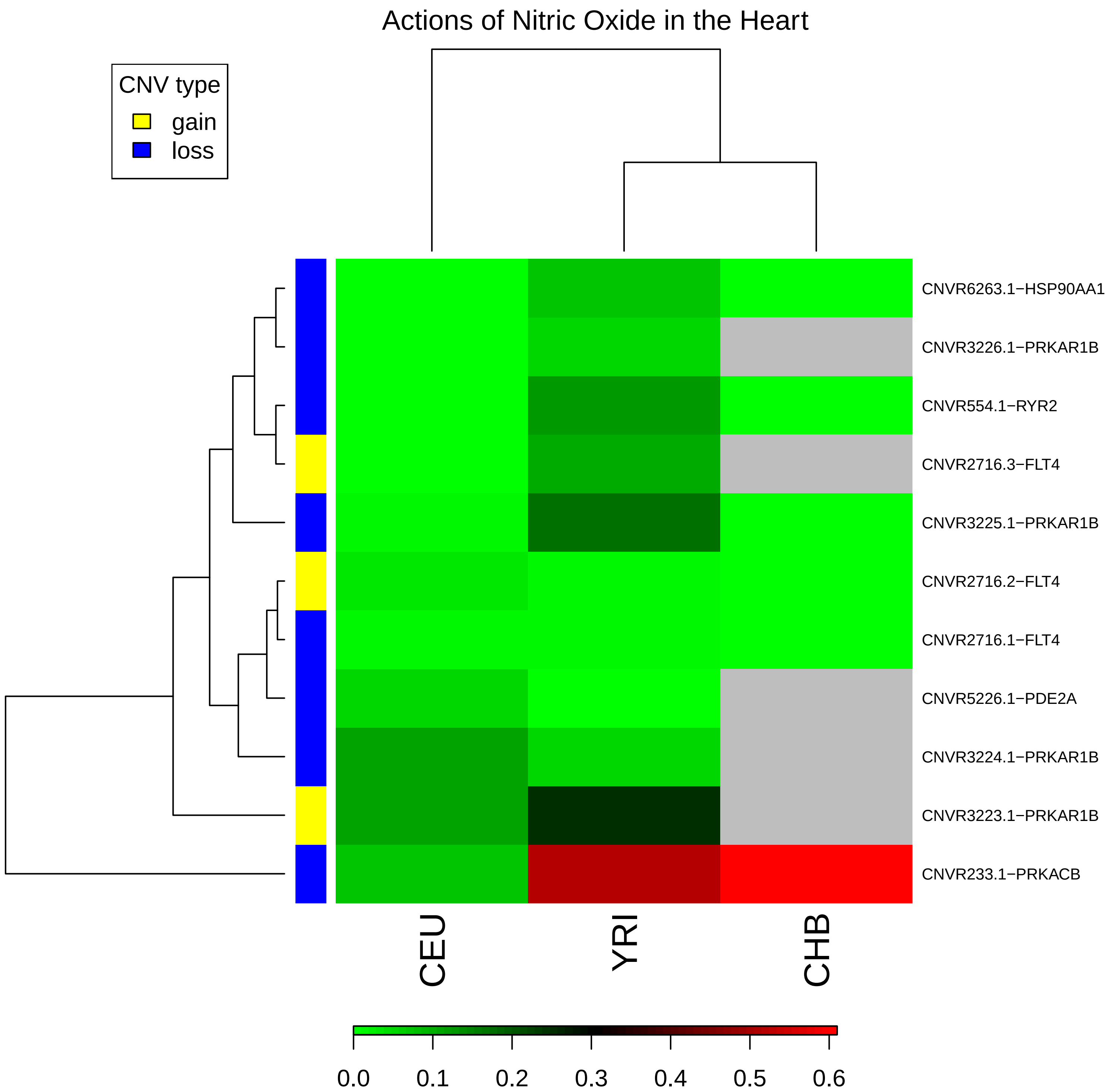
Actions of Nitric Oxide in the Heart
CNV type
gain
loss
CNVR6263.1−HSP90AA1
CNVR3226.1−PRKAR1B
CNVR554.1−RYR2
CNVR2716.3−FLT4
CNVR3225.1−PRKAR1B
CNVR2716.2−FLT4
CNVR2716.1−FLT4
CNVR5226.1−PDE2A
CNVR3224.1−PRKAR1B
CNVR3223.1−PRKAR1B
CNVR233.1−PRKACB
CEU
YRI
CHB
0.0
0.1
0.2
0.3
0.4
0.5
0.6

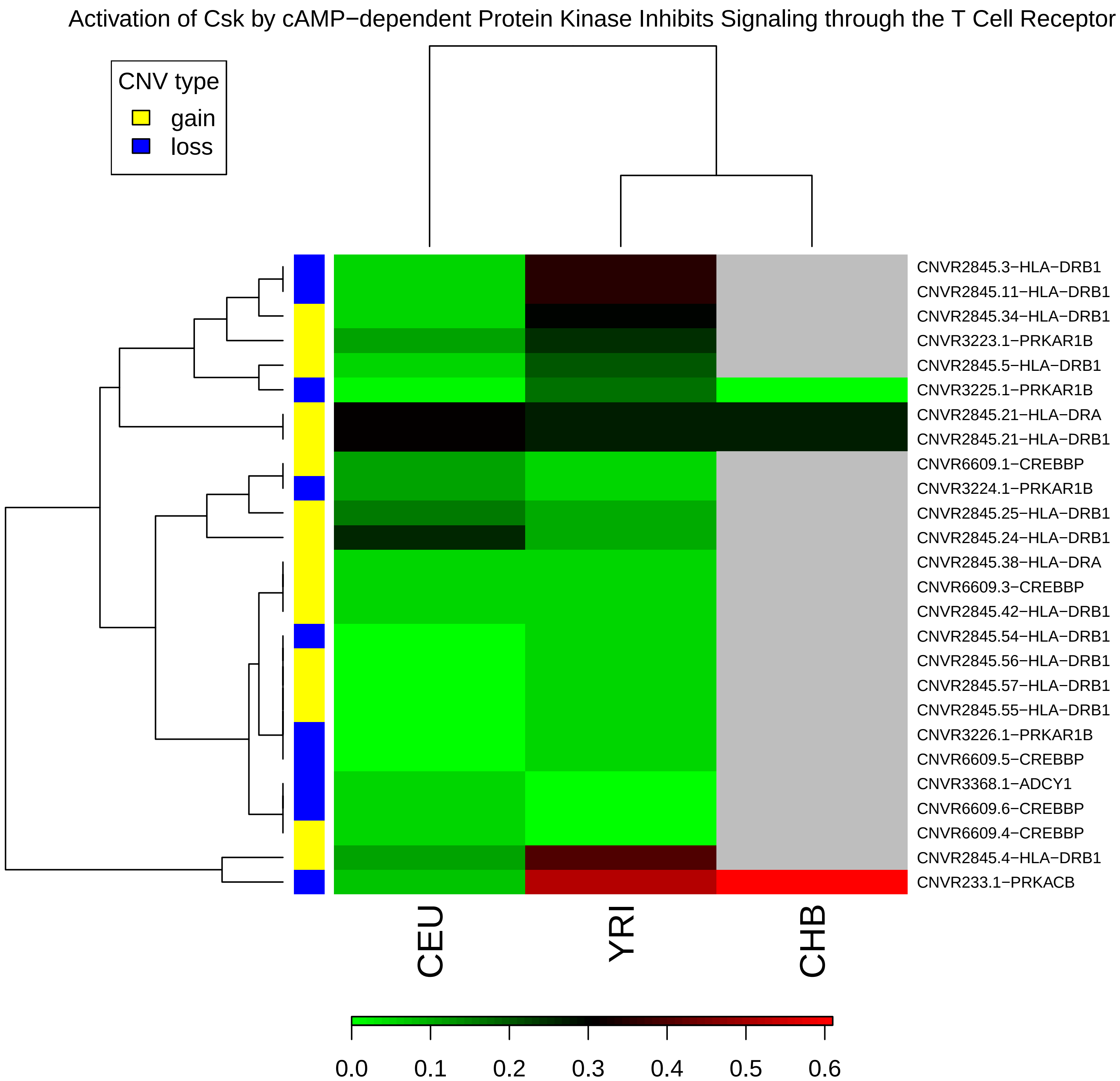

Activation of Csk by cAMP−dependent Protein Kinase Inhibits Signaling through the T Cell Receptor
CNV type
gain
loss
CNVR2845.3−HLA−DRB1
CNVR2845.11−HLA−DRB1
CNVR2845.34−HLA−DRB1
CNVR3223.1−PRKAR1B
CNVR2845.5−HLA−DRB1
CNVR3225.1−PRKAR1B
CNVR2845.21−HLA−DRA
CNVR2845.21−HLA−DRB1
CNVR6609.1−CREBBP
CNVR3224.1−PRKAR1B
CNVR2845.25−HLA−DRB1
CNVR2845.24−HLA−DRB1
CNVR2845.38−HLA−DRA
CNVR6609.3−CREBBP
CNVR2845.42−HLA−DRB1
CNVR2845.54−HLA−DRB1
CNVR2845.56−HLA−DRB1
CNVR2845.57−HLA−DRB1
CNVR2845.55−HLA−DRB1
CNVR3226.1−PRKAR1B
CNVR6609.5−CREBBP
CNVR3368.1−ADCY1
CNVR6609.6−CREBBP
CNVR6609.4−CREBBP
CNVR2845.4−HLA−DRB1
CNVR233.1−PRKACB
CEU
YRI
CHB
0.0
0.1
0.2
0.3
0.4
0.5
0.6

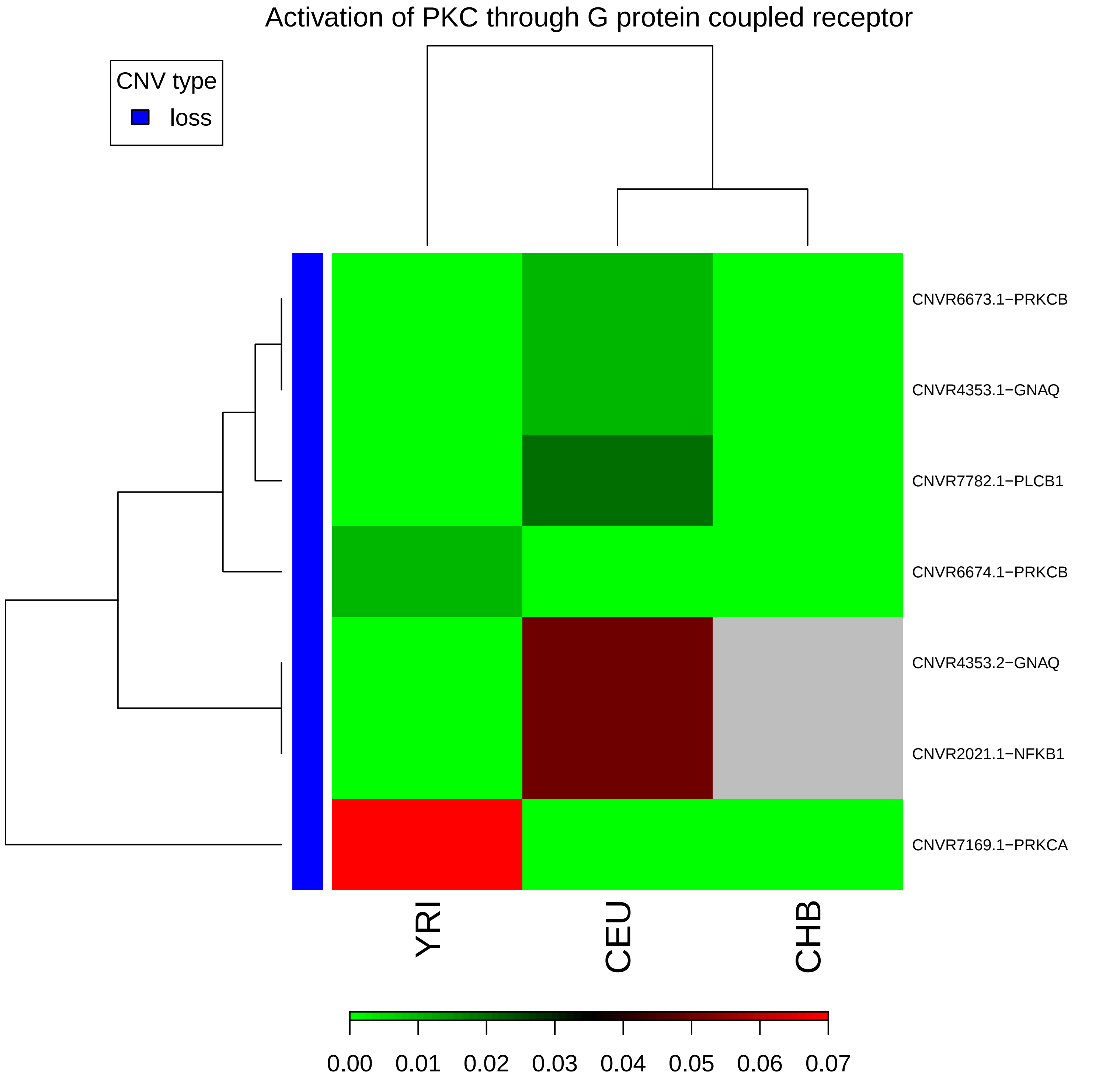
Activation of PKC through G protein coupled receptor
CNV type
loss
CNVR6673.1−PRKCB
CNVR4353.1−GNAQ
CNVR7782.1−PLCB1
CNVR6674.1−PRKCB
CNVR4353.2−GNAQ
CNVR2021.1−NFKB1
CNVR7169.1−PRKCA
YRI
CEU
CHB
0.00
0.01
0.02
0.03
0.04
0.05
0.06
0.07

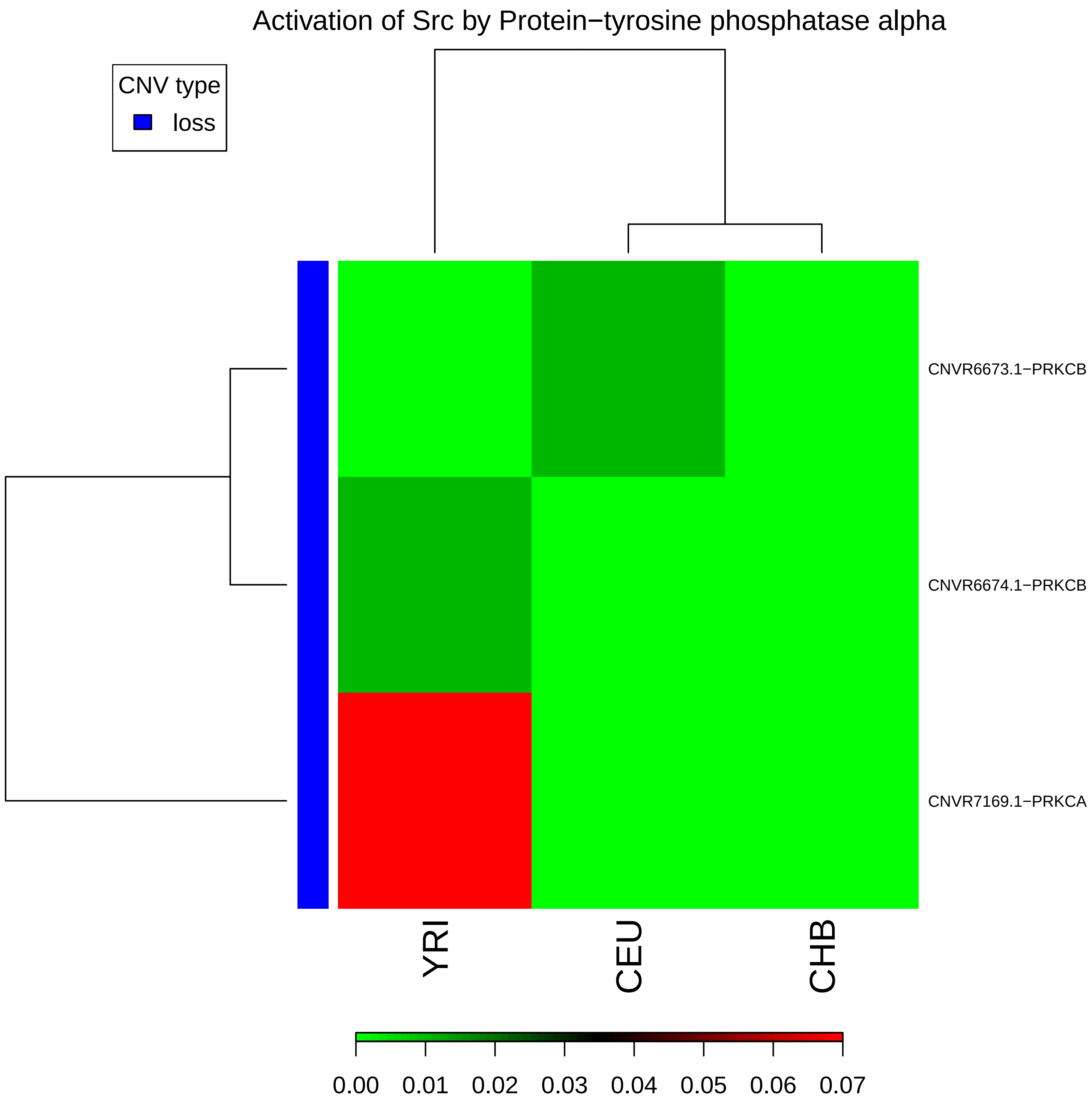

Activation of Src by Protein−tyrosine phosphatase alpha
CNV type
loss
CNVR6673.1−PRKCB
CNVR6674.1−PRKCB
CNVR7169.1−PRKCA
YRI
CEU
CHB
0.00
0.01
0.02
0.03
0.04
0.05
0.06
0.07

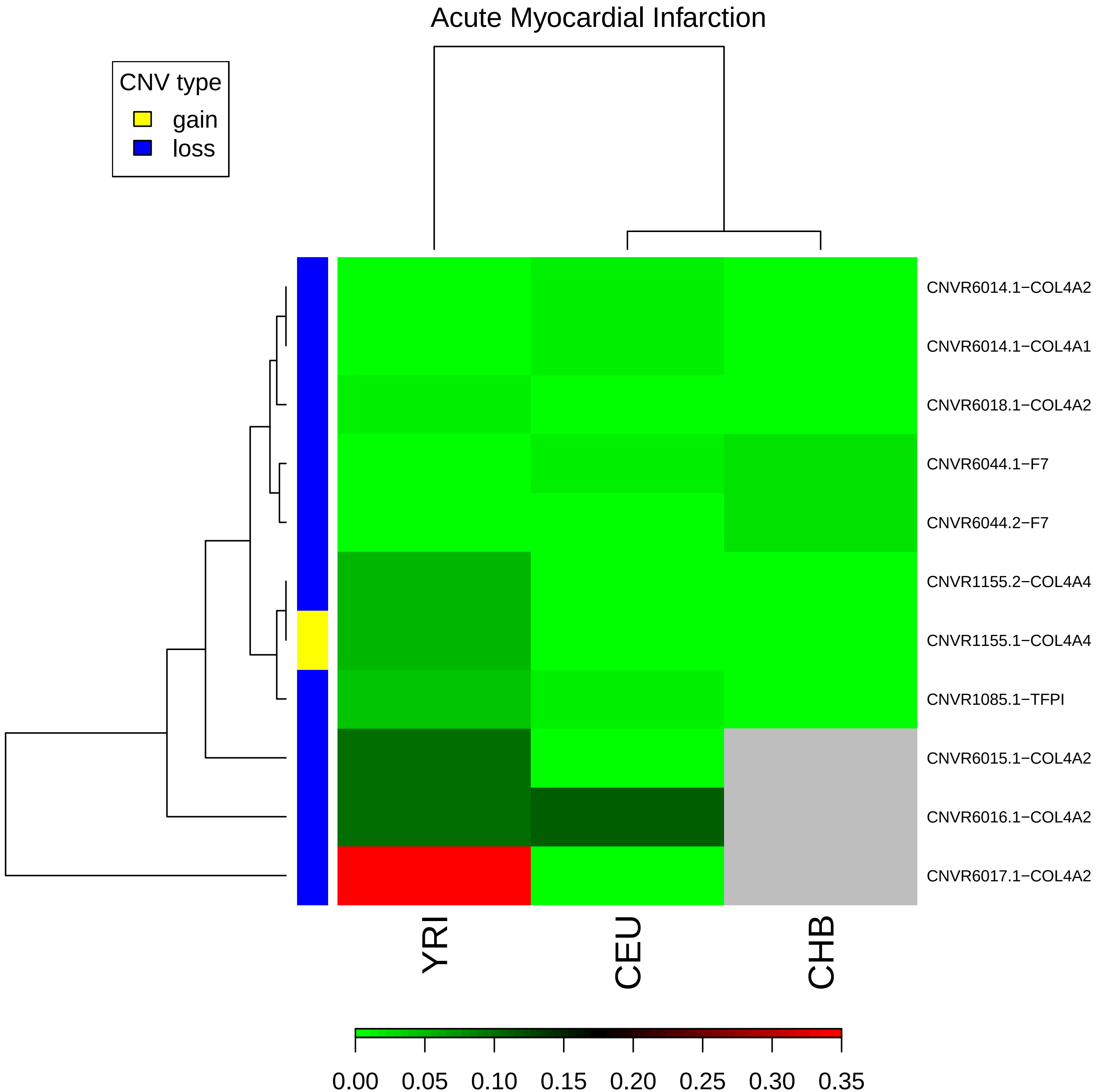

Acute Myocardial Infarction
CNV type
gain
loss
CNVR6014.1−COL4A2
CNVR6014.1−COL4A1
CNVR6018.1−COL4A2
CNVR6044.1−F7
CNVR6044.2−F7
CNVR1155.2−COL4A4
CNVR1155.1−COL4A4
CNVR1085.1−TFPI
CNVR6015.1−COL4A2
CNVR6016.1−COL4A2
CNVR6017.1−COL4A2
YRI
CEU
CHB
0.00
0.05
0.10
0.15
0.20
0.25
0.30
0.35

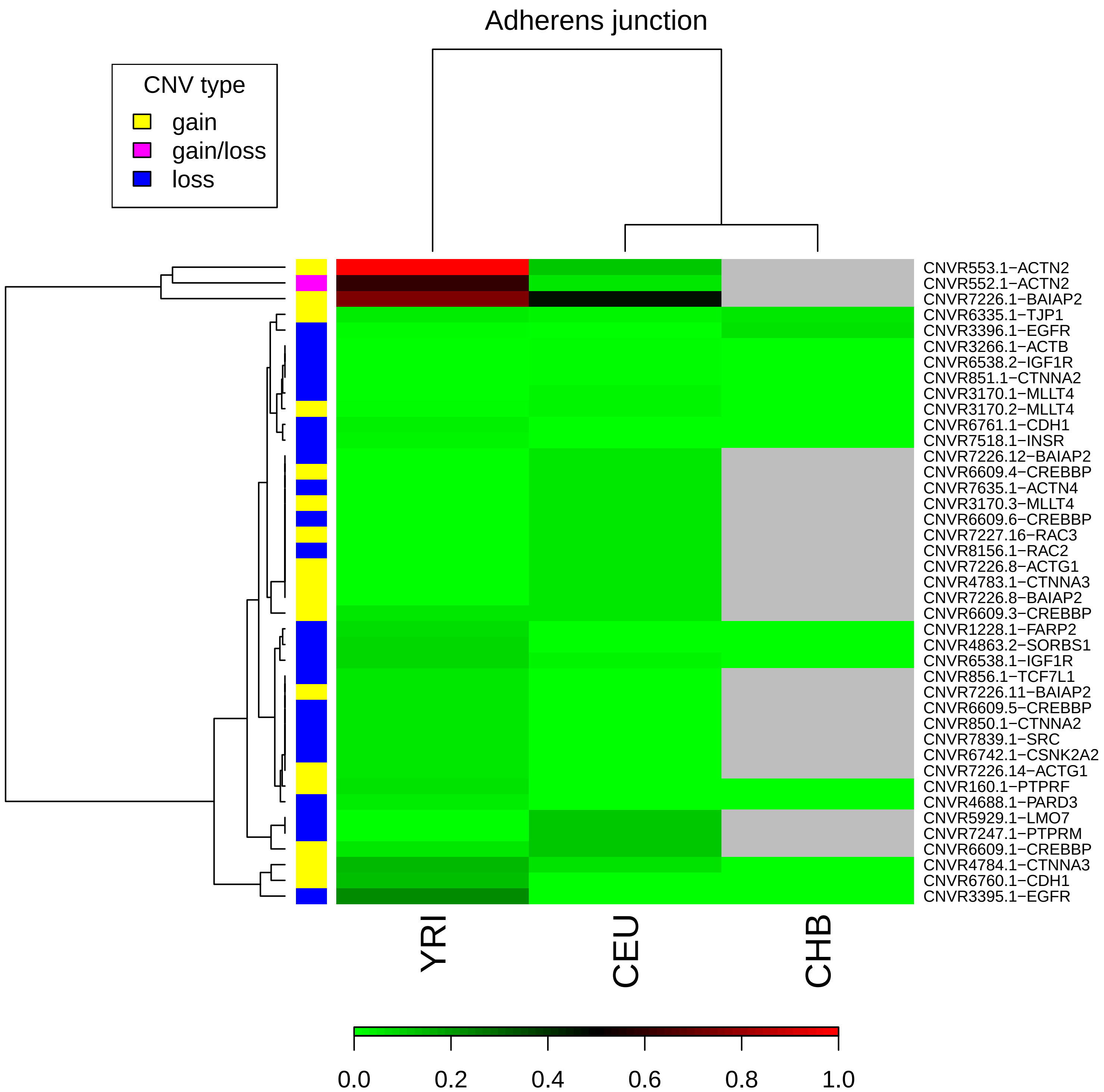
Adherens junction
CNV type
gain
gain/loss
loss
CNVR553.1−ACTN2
CNVR552.1−ACTN2
CNVR7226.1−BAIAP2
CNVR6335.1−TJP1
CNVR3396.1−EGFR
CNVR3266.1−ACTB
CNVR6538.2−IGF1R
CNVR851.1−CTNNA2
CNVR3170.1−MLLT4
CNVR3170.2−MLLT4
CNVR6761.1−CDH1
CNVR7518.1−INSR
CNVR7226.12−BAIAP2
CNVR6609.4−CREBBP
CNVR7635.1−ACTN4
CNVR3170.3−MLLT4
CNVR6609.6−CREBBP
CNVR7227.16−RAC3
CNVR8156.1−RAC2
CNVR7226.8−ACTG1
CNVR4783.1−CTNNA3
CNVR7226.8−BAIAP2
CNVR6609.3−CREBBP
CNVR1228.1−FARP2
CNVR4863.2−SORBS1
CNVR6538.1−IGF1R
CNVR856.1−TCF7L1
CNVR7226.11−BAIAP2
CNVR6609.5−CREBBP
CNVR850.1−CTNNA2
CNVR7839.1−SRC
CNVR6742.1−CSNK2A2
CNVR7226.14−ACTG1
CNVR160.1−PTPRF
CNVR4688.1−PARD3
CNVR5929.1−LMO7
CNVR7247.1−PTPRM
CNVR6609.1−CREBBP
CNVR4784.1−CTNNA3
CNVR6760.1−CDH1
CNVR3395.1−EGFR
YRI
CEU
CHB
0.0
0.2
0.4
0.6
0.8
1.0

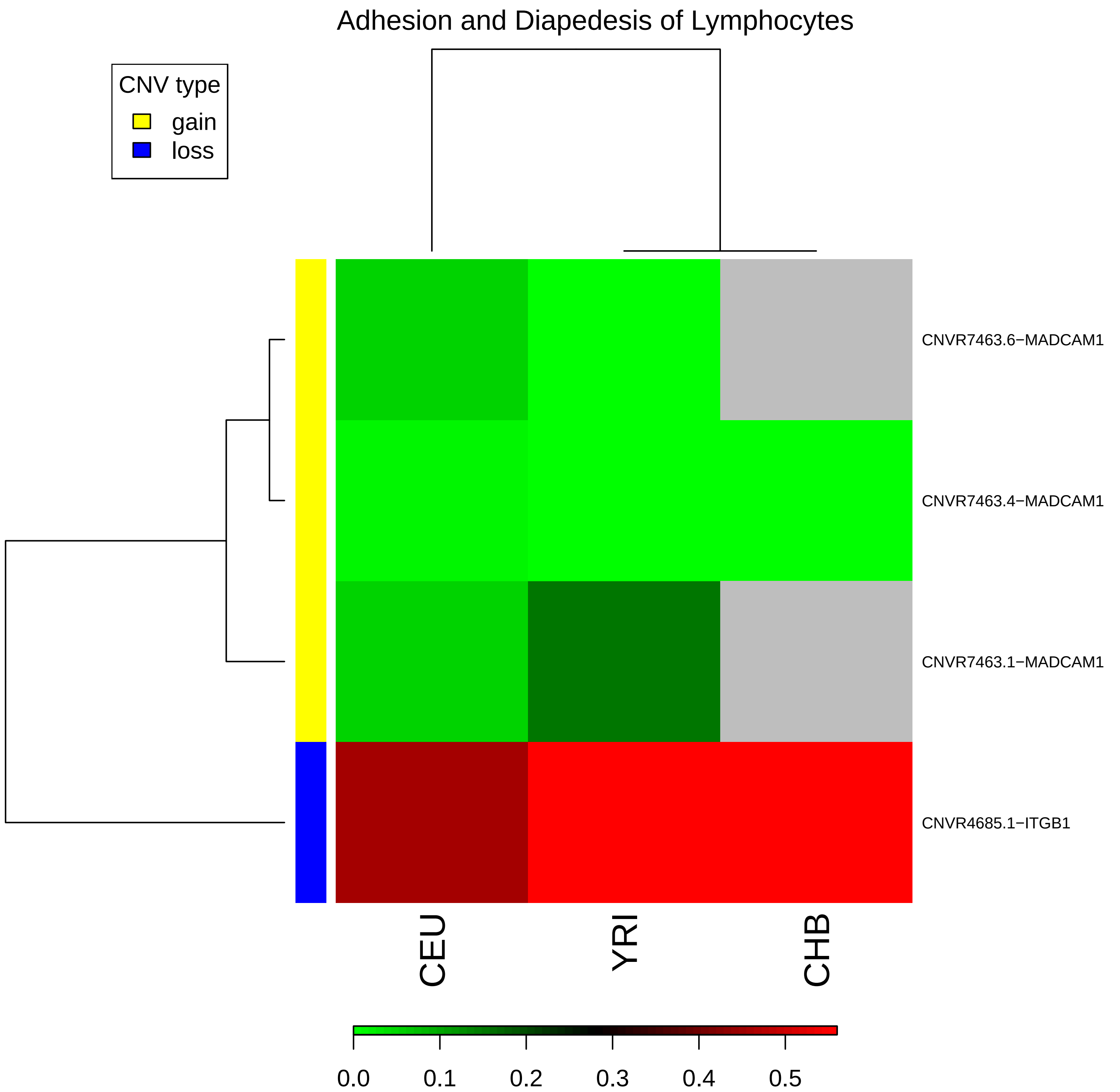
Adhesion and Diapedesis of Lymphocytes
CNV type
gain
loss
CNVR7463.6−MADCAM1
CNVR7463.4−MADCAM1
CNVR7463.1−MADCAM1
CNVR4685.1−ITGB1
CEU
YRI
CHB
0.0
0.1
0.2
0.3
0.4
0.5

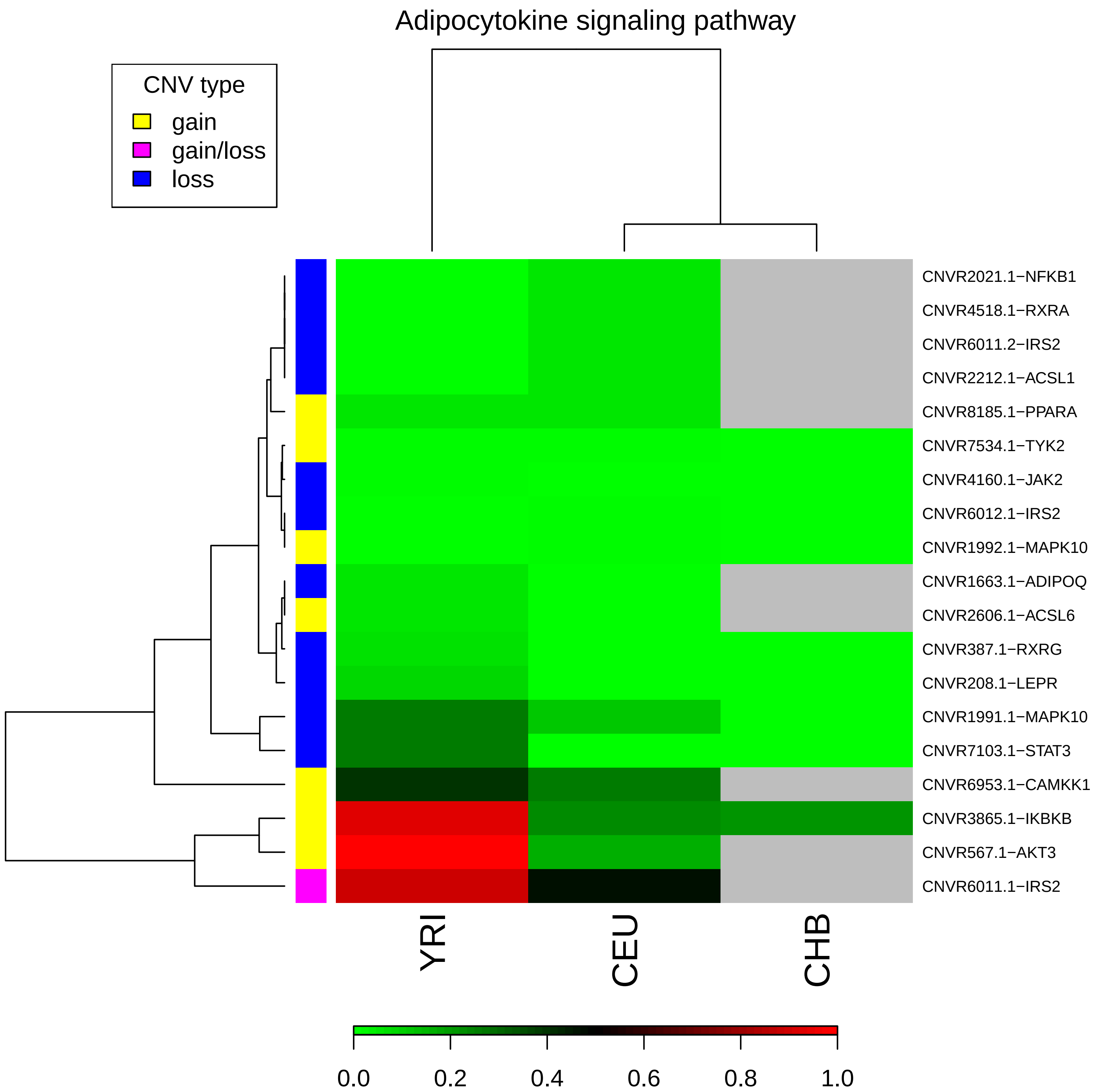

Adipocytokine signaling pathway
CNV type
gain
gain/loss
loss
CNVR2021.1−NFKB1
CNVR4518.1−RXRA
CNVR6011.2−IRS2
CNVR2212.1−ACSL1
CNVR8185.1−PPARA
CNVR7534.1−TYK2
CNVR4160.1−JAK2
CNVR6012.1−IRS2
CNVR1992.1−MAPK10
CNVR1663.1−ADIPOQ
CNVR2606.1−ACSL6
CNVR387.1−RXRG
CNVR208.1−LEPR
CNVR1991.1−MAPK10
CNVR7103.1−STAT3
CNVR6953.1−CAMKK1
CNVR3865.1−IKBKB
CNVR567.1−AKT3
CNVR6011.1−IRS2
YRI
CEU
CHB
0.0
0.2
0.4
0.6
0.8
1.0

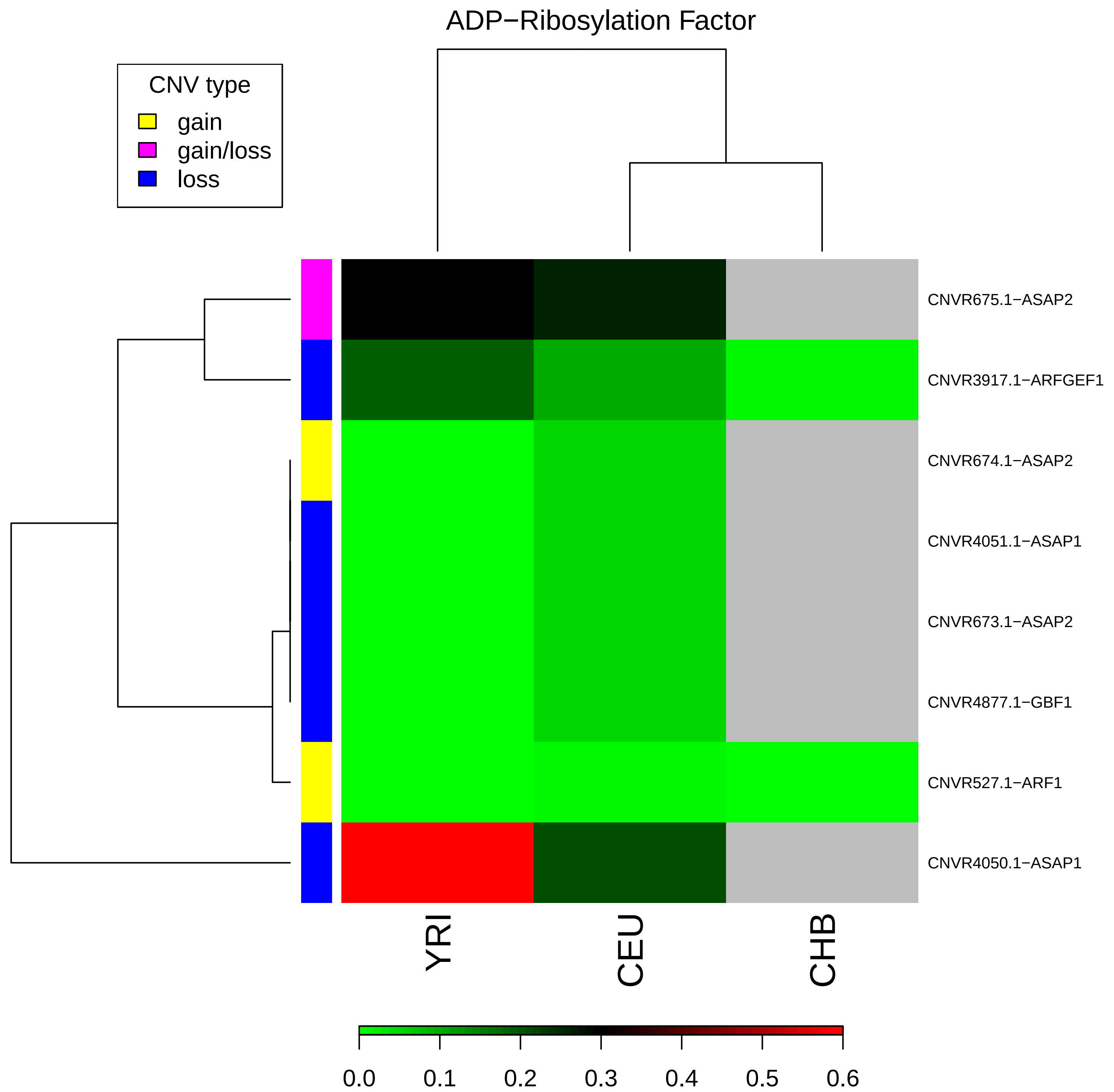
ADP−Ribosylation Factor
CNV type
gain
gain/loss
loss
CNVR675.1−ASAP2
CNVR3917.1−ARFGEF1
CNVR674.1−ASAP2
CNVR4051.1−ASAP1
CNVR673.1−ASAP2
CNVR4877.1−GBF1
CNVR527.1−ARF1
CNVR4050.1−ASAP1
YRI
CEU
CHB
0.0
0.1
0.2
0.3
0.4
0.5
0.6

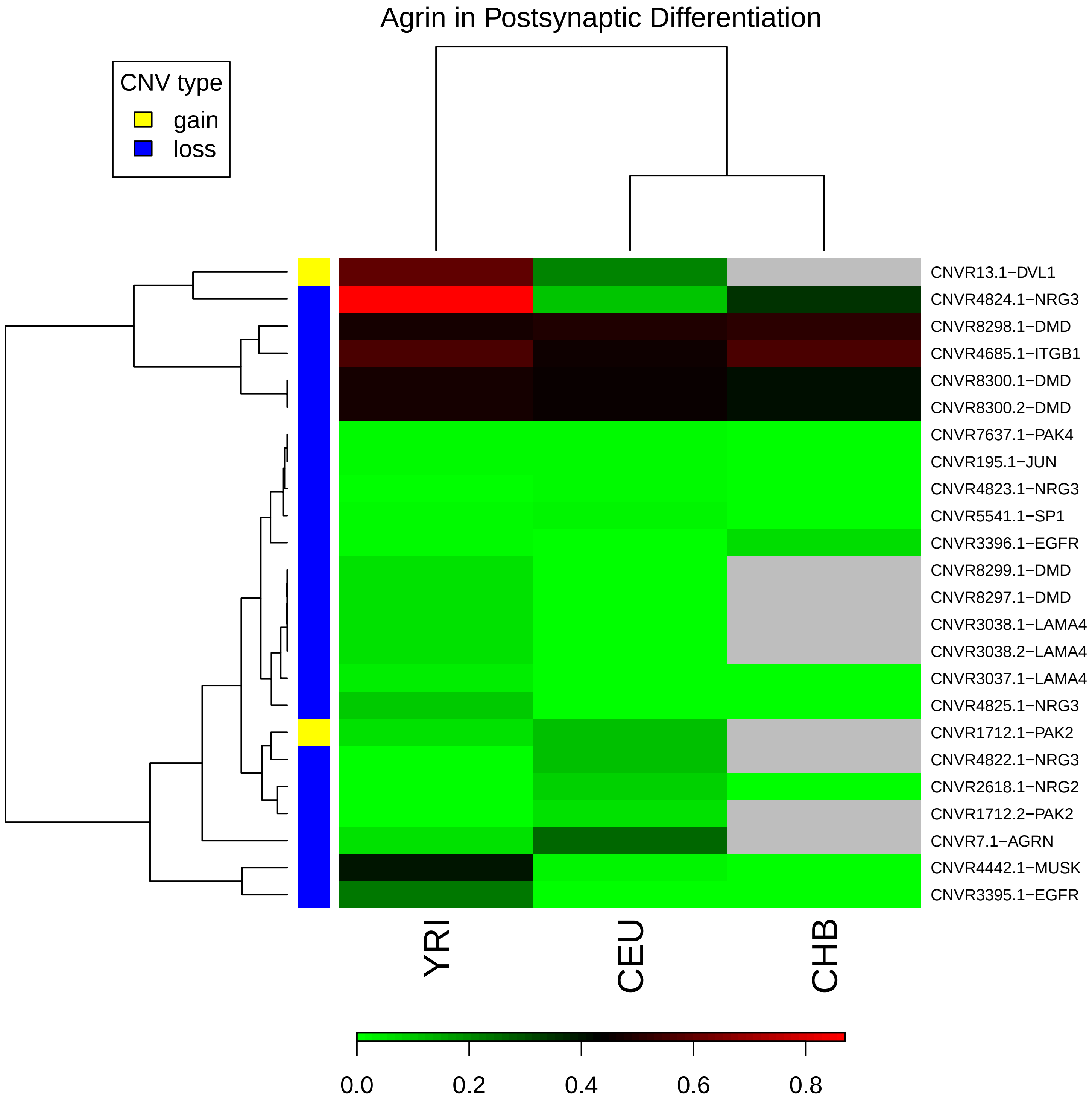

Agrin in Postsynaptic Differentiation
CNV type
gain
loss
CNVR13.1−DVL1
CNVR4824.1−NRG3
CNVR8298.1−DMD
CNVR4685.1−ITGB1
CNVR8300.1−DMD
CNVR8300.2−DMD
CNVR7637.1−PAK4
CNVR195.1−JUN
CNVR4823.1−NRG3
CNVR5541.1−SP1
CNVR3396.1−EGFR
CNVR8299.1−DMD
CNVR8297.1−DMD
CNVR3038.1−LAMA4
CNVR3038.2−LAMA4
CNVR3037.1−LAMA4
CNVR4825.1−NRG3
CNVR1712.1−PAK2
CNVR4822.1−NRG3
CNVR2618.1−NRG2
CNVR1712.2−PAK2
CNVR7.1−AGRN
CNVR4442.1−MUSK
CNVR3395.1−EGFR
YRI
CEU
CHB
0.0
0.2
0.4
0.6
0.8

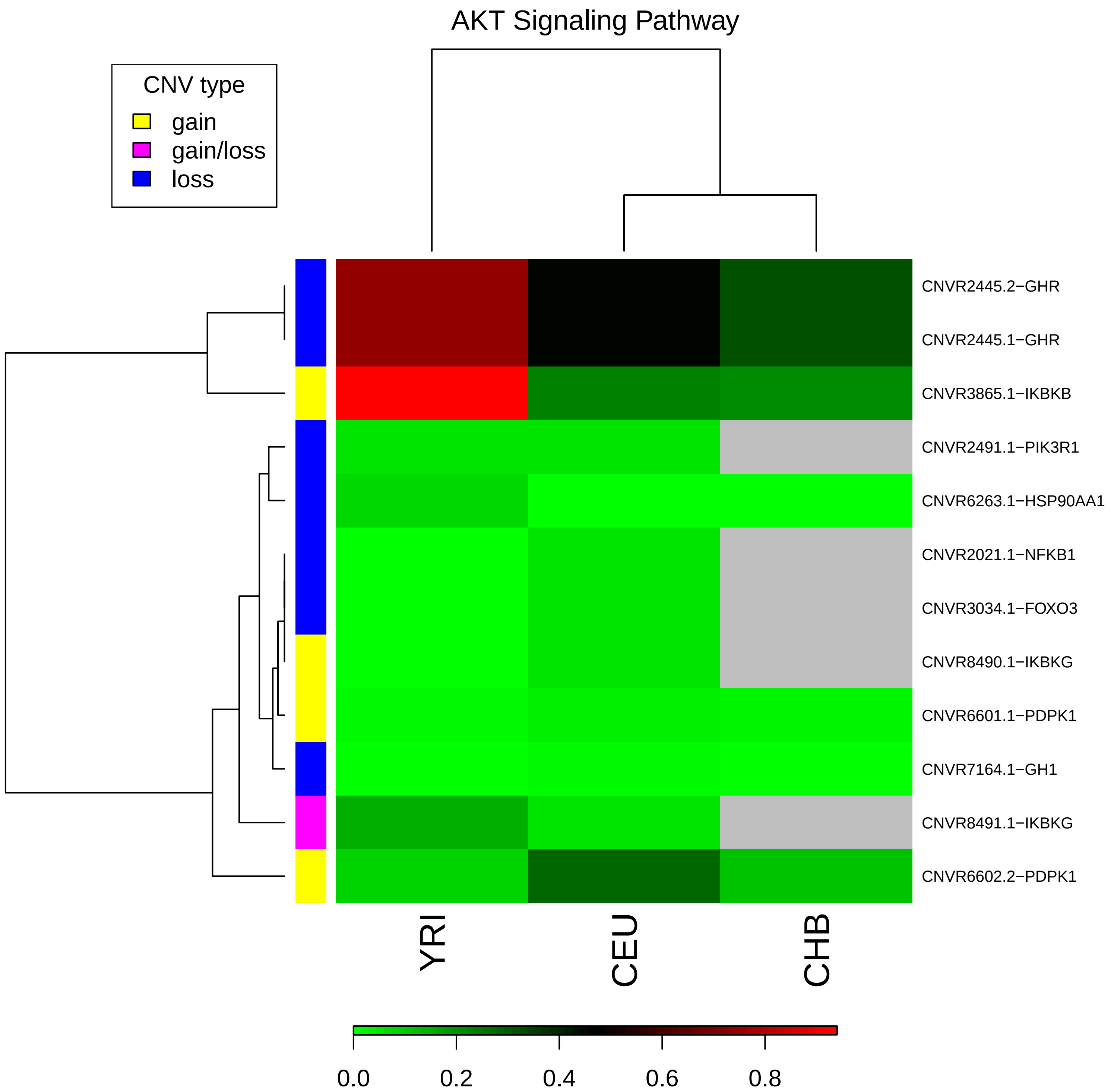
AKT Signaling Pathway
CNV type
gain
gain/loss
loss
CNVR2445.2−GHR
CNVR2445.1−GHR
CNVR3865.1−IKBKB
CNVR2491.1−PIK3R1
CNVR6263.1−HSP90AA1
CNVR2021.1−NFKB1
CNVR3034.1−FOXO3
CNVR8490.1−IKBKG
CNVR6601.1−PDPK1
CNVR7164.1−GH1
CNVR8491.1−IKBKG
CNVR6602.2−PDPK1
YRI
CEU
CHB
0.0
0.2
0.4
0.6
0.8

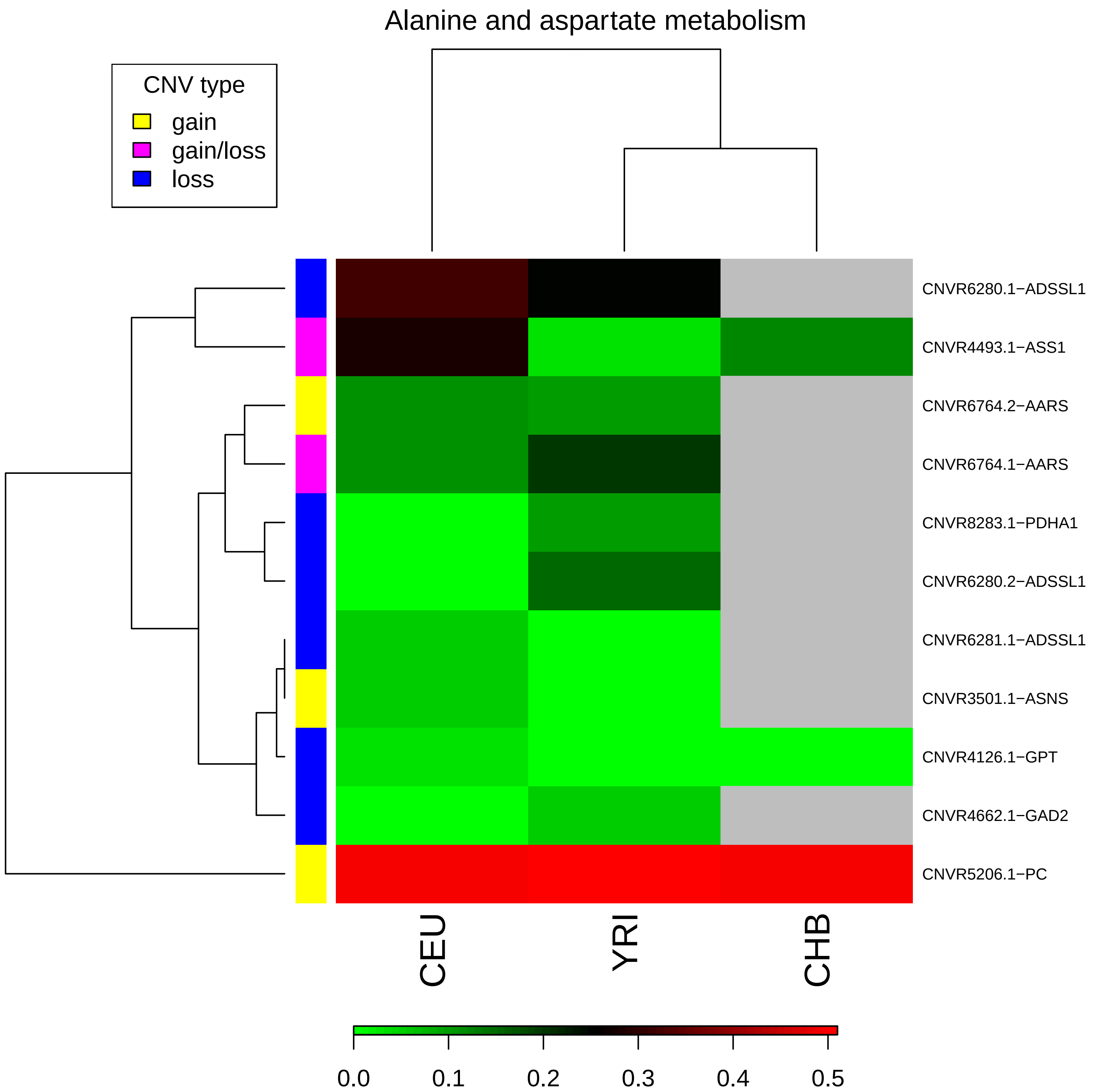

Alanine and aspartate metabolism
CNV type
gain
gain/loss
loss
CNVR6280.1−ADSSL1
CNVR4493.1−ASS1
CNVR6764.2−AARS
CNVR6764.1−AARS
CNVR8283.1−PDHA1
CNVR6280.2−ADSSL1
CNVR6281.1−ADSSL1
CNVR3501.1−ASNS
CNVR4126.1−GPT
CNVR4662.1−GAD2
CNVR5206.1−PC
CEU
YRI
CHB
0.0
0.1
0.2
0.3
0.4
0.5

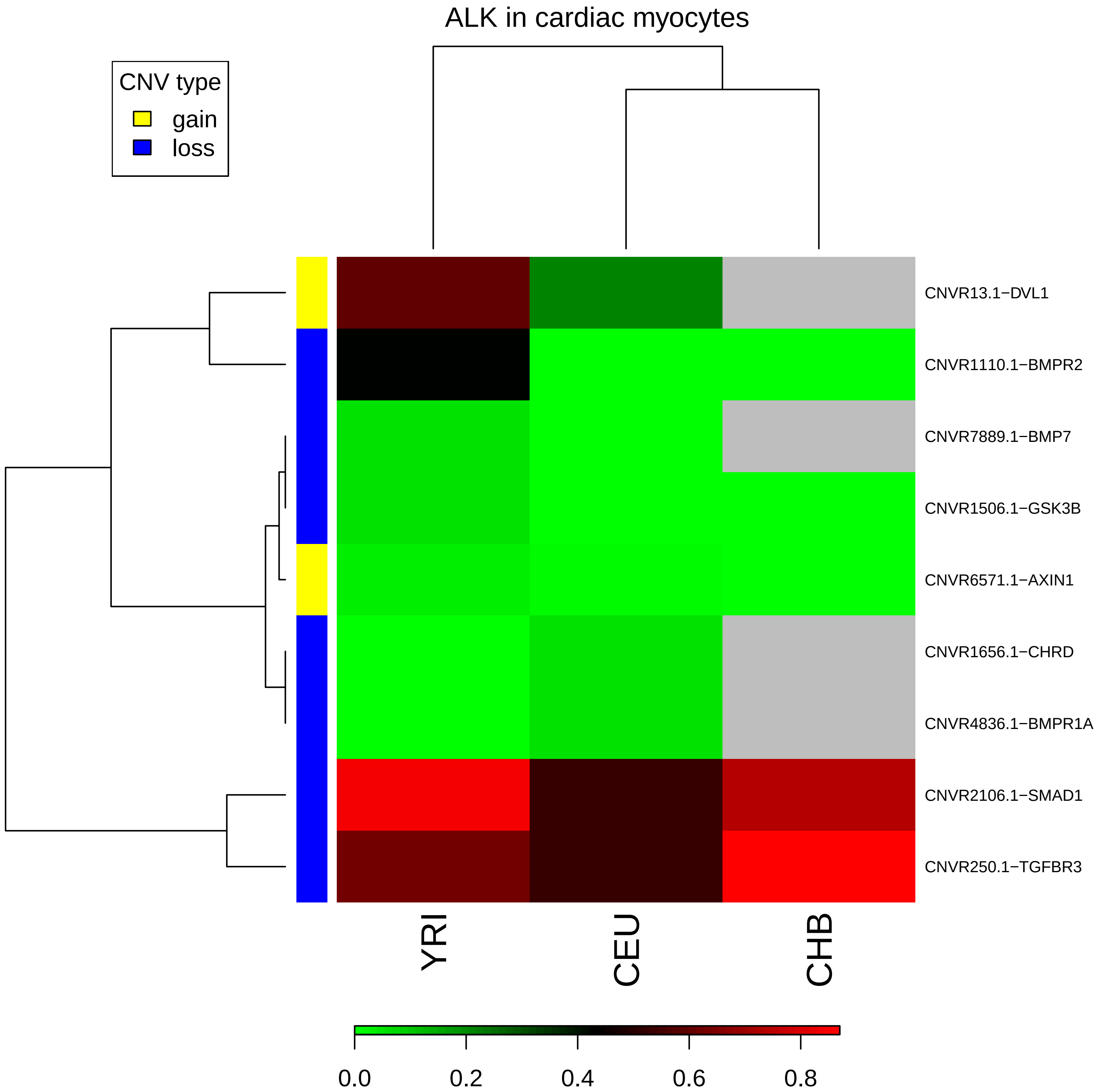
ALK in cardiac myocytes
CNV type
gain
loss
CNVR13.1−DVL1
CNVR1110.1−BMPR2
CNVR7889.1−BMP7
CNVR1506.1−GSK3B
CNVR6571.1−AXIN1
CNVR1656.1−CHRD
CNVR4836.1−BMPR1A
CNVR2106.1−SMAD1
CNVR250.1−TGFBR3
YRI
CEU
CHB
0.0
0.2
0.4
0.6
0.8

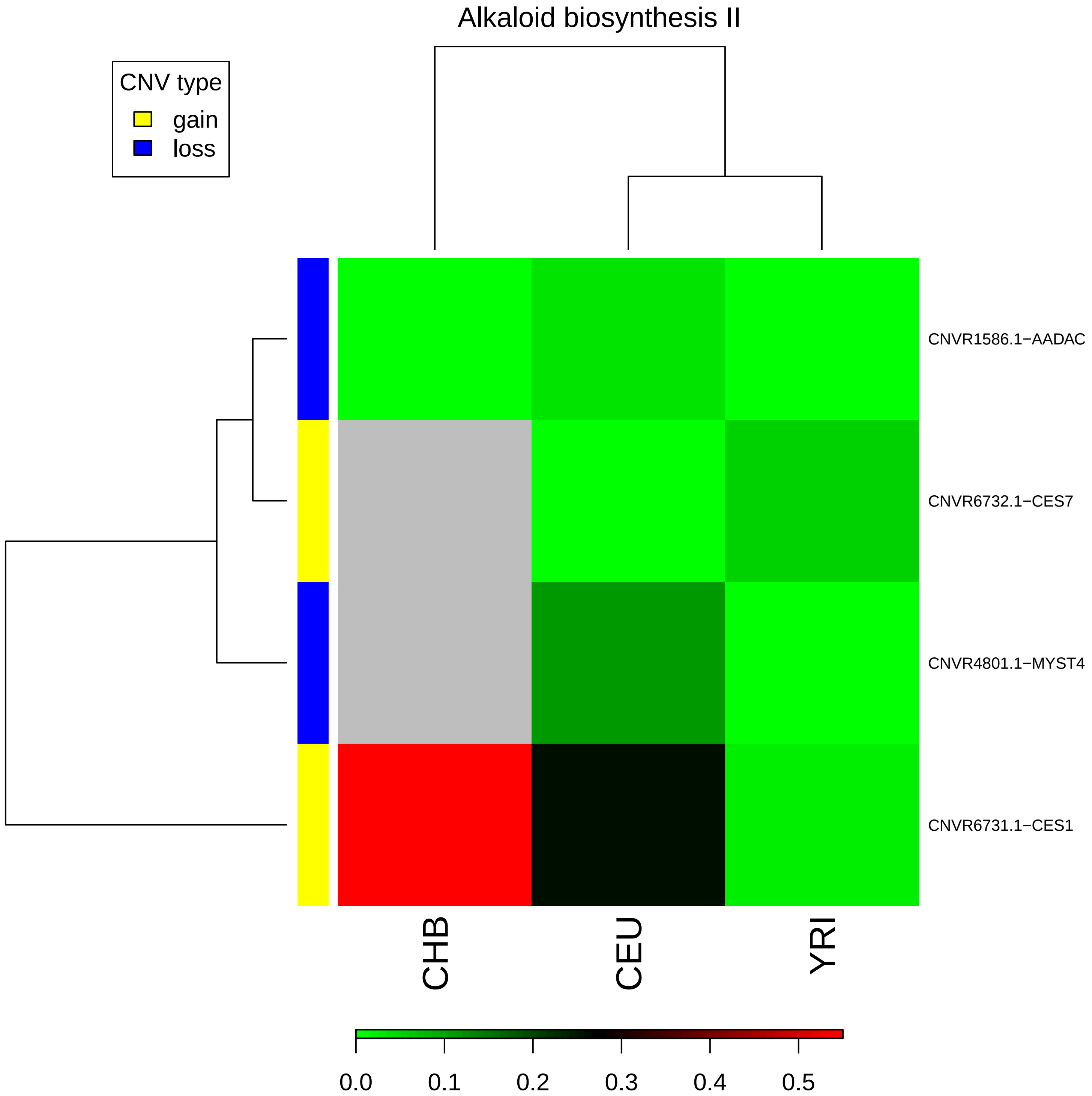
Alkaloid biosynthesis II
CNV type
gain
loss
CNVR1586.1−AADAC
CNVR6732.1−CES7
CNVR4801.1−MYST4
CNVR6731.1−CES1
CHB
CEU
YRI
0.0
0.1
0.2
0.3
0.4
0.5

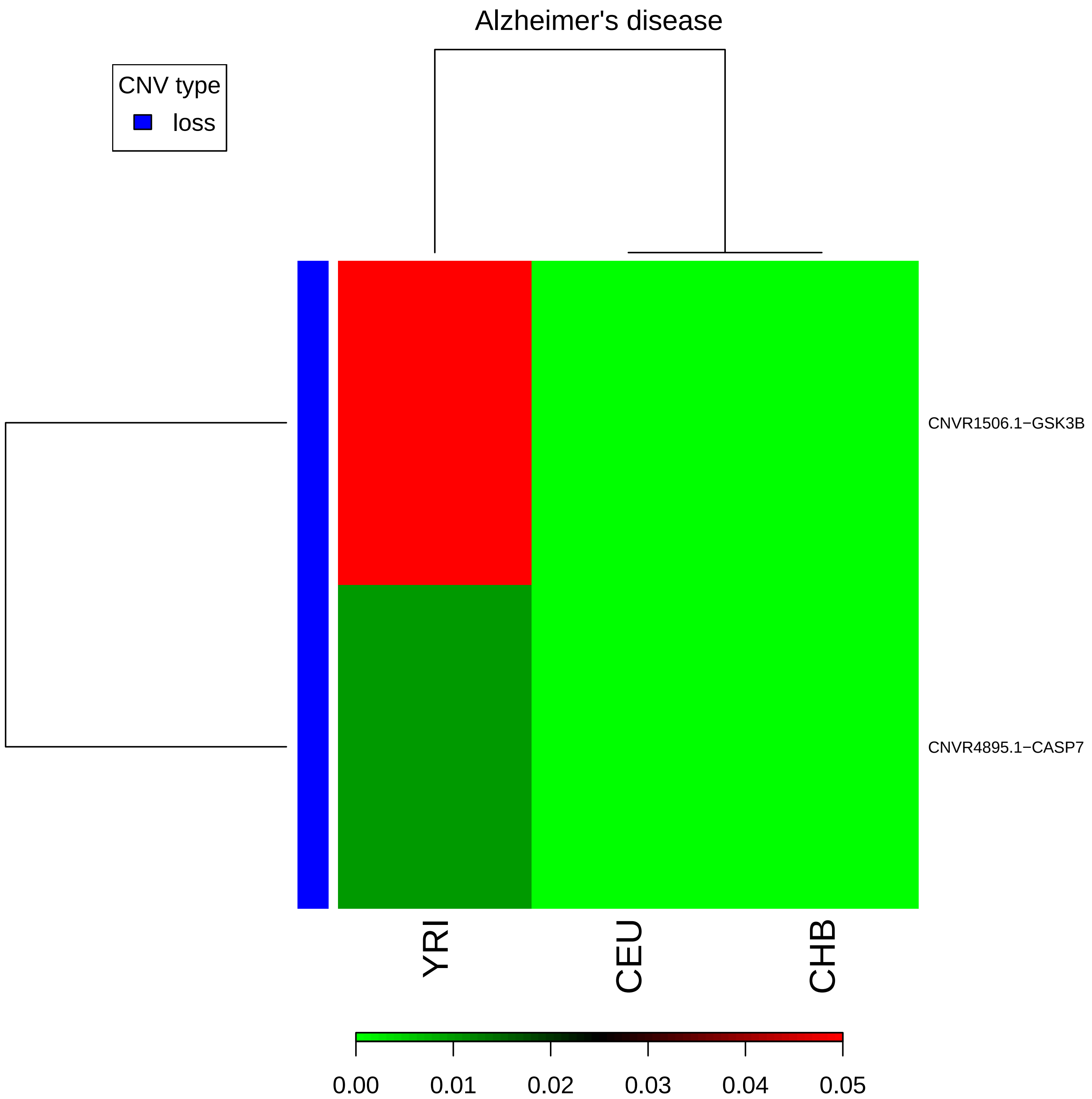
Alzheimer's disease
CNV type
loss
CNVR1506.1−GSK3B
CNVR4895.1−CASP7
YRI
CEU
CHB
0.00
0.01
0.02
0.03
0.04
0.05

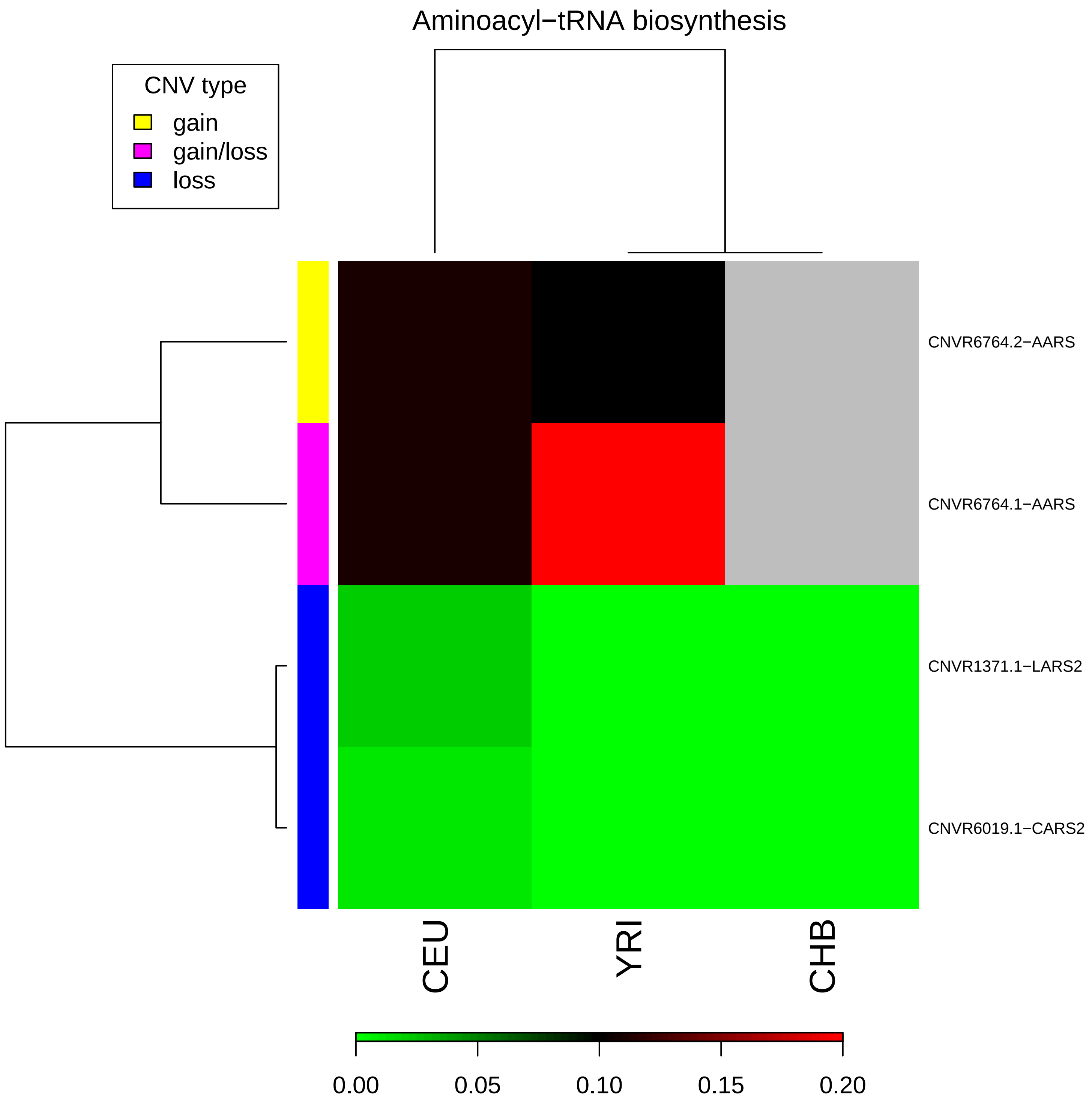
Aminoacyl−tRNA biosynthesis
CNV type
gain
gain/loss
loss
CNVR6764.2−AARS
CNVR6764.1−AARS
CNVR1371.1−LARS2
CNVR6019.1−CARS2
CEU
YRI
CHB
0.00
0.05
0.10
0.15
0.20

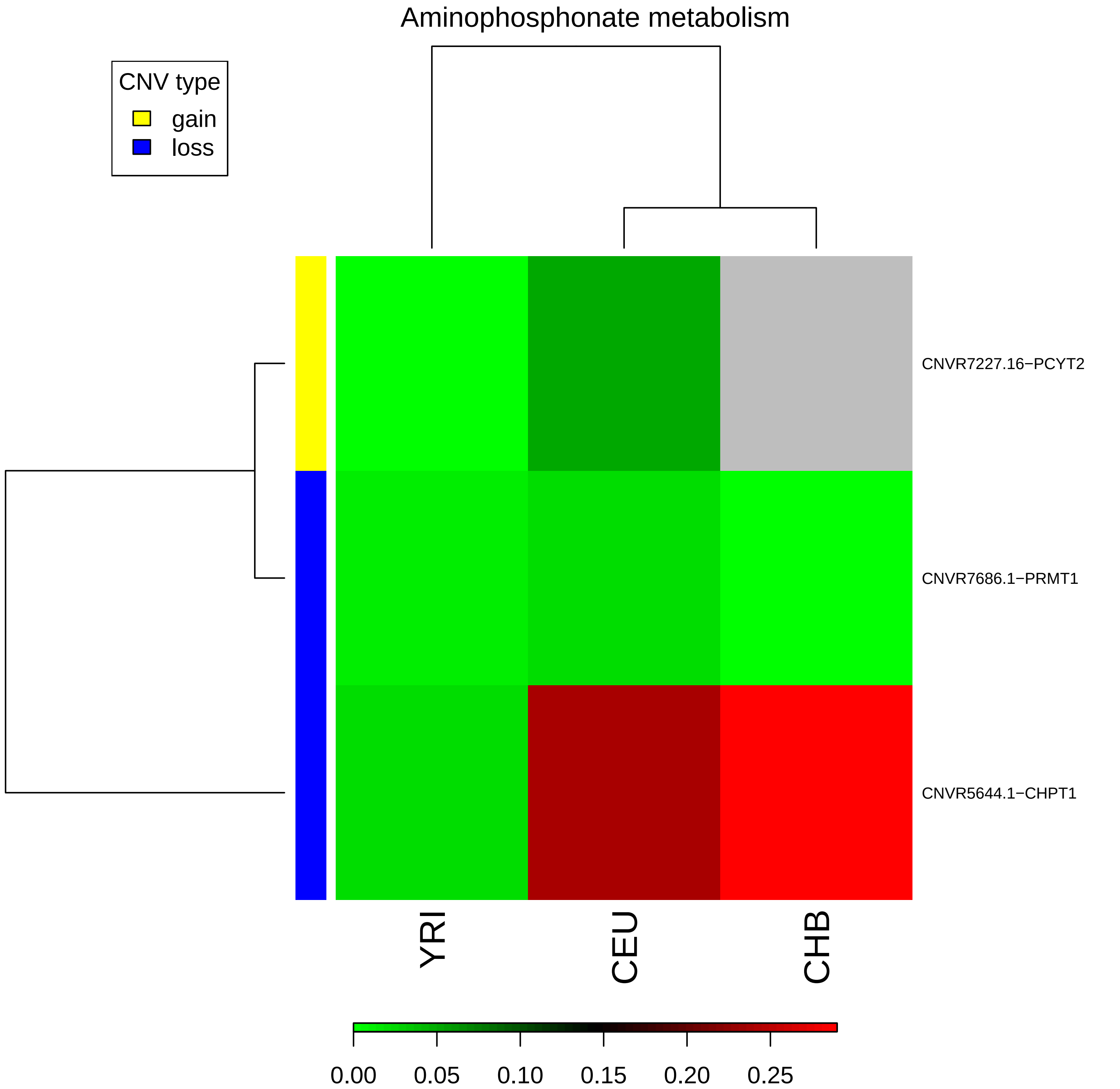

Aminophosphonate metabolism
CNV type
gain
loss
CNVR7227.16−PCYT2
CNVR7686.1−PRMT1
CNVR5644.1−CHPT1
YRI
CEU
CHB
0.00
0.05
0.10
0.15
0.20
0.25

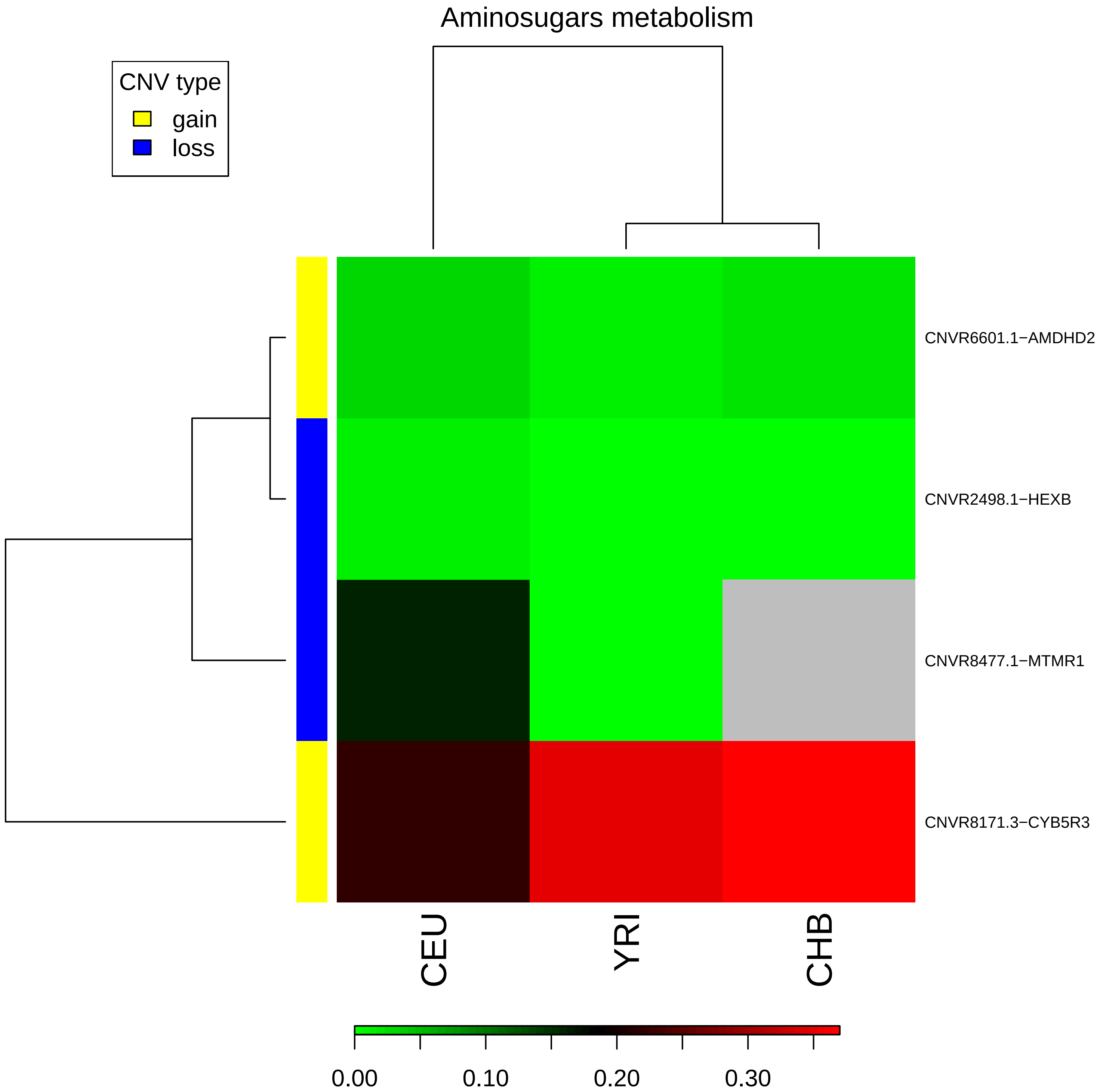

Aminosugars metabolism
CNV type
gain
loss
CNVR6601.1−AMDHD2
CNVR2498.1−HEXB
CNVR8477.1−MTMR1
CNVR8171.3−CYB5R3
CEU
YRI
CHB
0.00
0.10
0.20
0.30

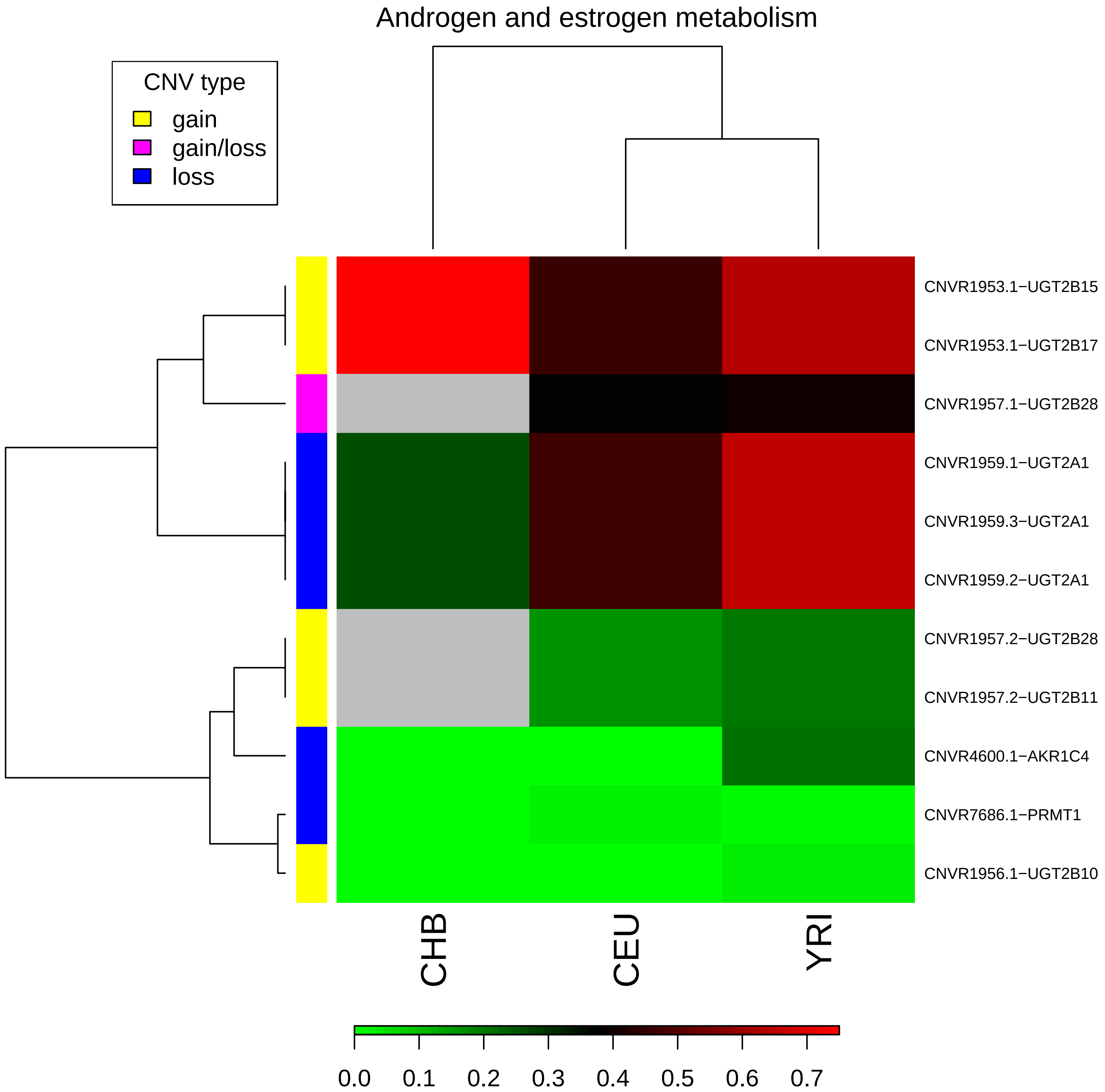
Androgen and estrogen metabolism
CNV type
gain
gain/loss
loss
CNVR1953.1−UGT2B15
CNVR1953.1−UGT2B17
CNVR1957.1−UGT2B28
CNVR1959.1−UGT2A1
CNVR1959.3−UGT2A1
CNVR1959.2−UGT2A1
CNVR1957.2−UGT2B28
CNVR1957.2−UGT2B11
CNVR4600.1−AKR1C4
CNVR7686.1−PRMT1
CNVR1956.1−UGT2B10
CHB
CEU
YRI
0.0
0.1
0.2
0.3
0.4
0.5
0.6
0.7

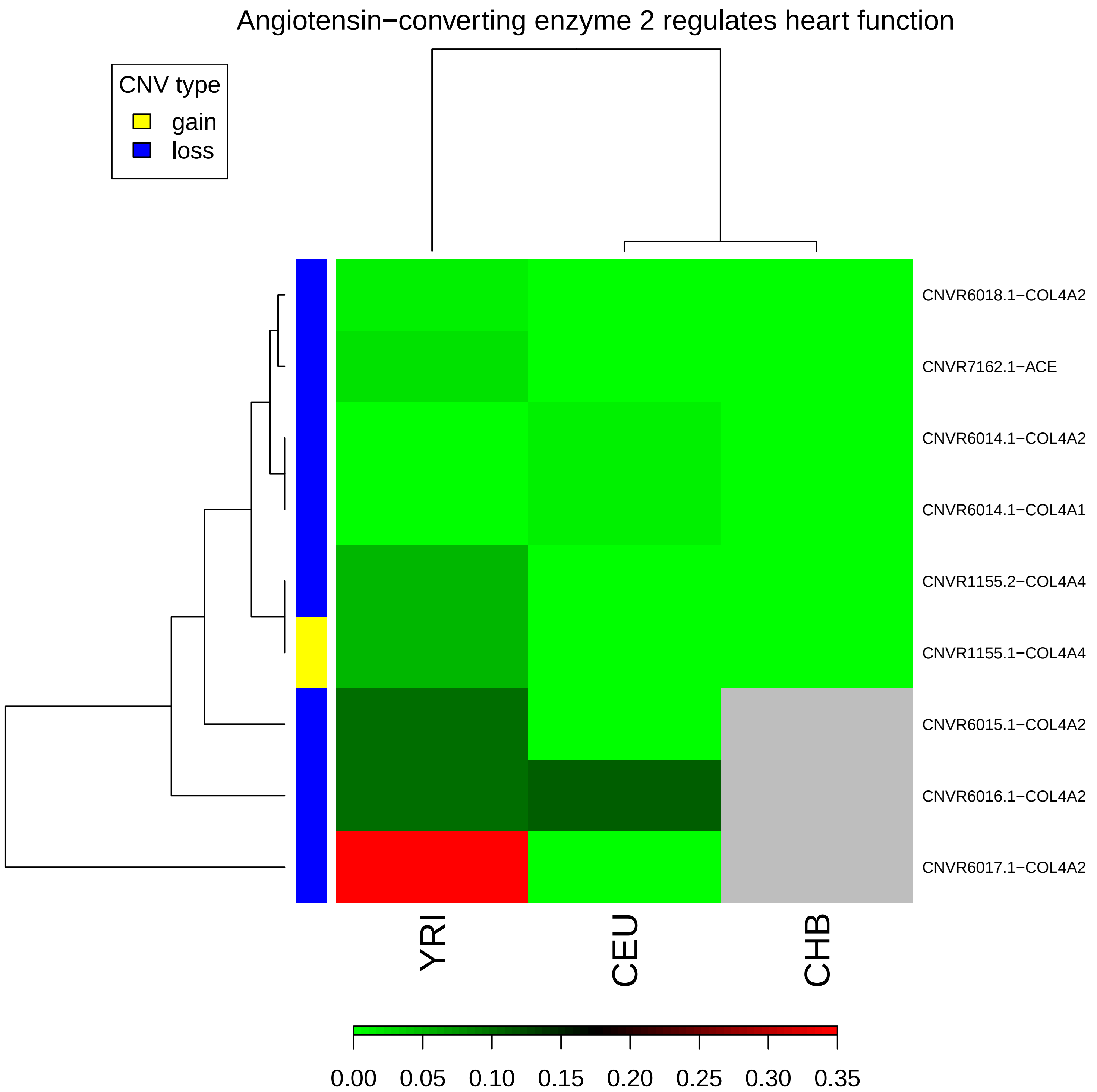

Angiotensin−converting enzyme 2 regulates heart function
CNV type
gain
loss
CNVR6018.1−COL4A2
CNVR7162.1−ACE
CNVR6014.1−COL4A2
CNVR6014.1−COL4A1
CNVR1155.2−COL4A4
CNVR1155.1−COL4A4
CNVR6015.1−COL4A2
CNVR6016.1−COL4A2
CNVR6017.1−COL4A2
YRI
CEU
CHB
0.00
0.05
0.10
0.15
0.20
0.25
0.30
0.35

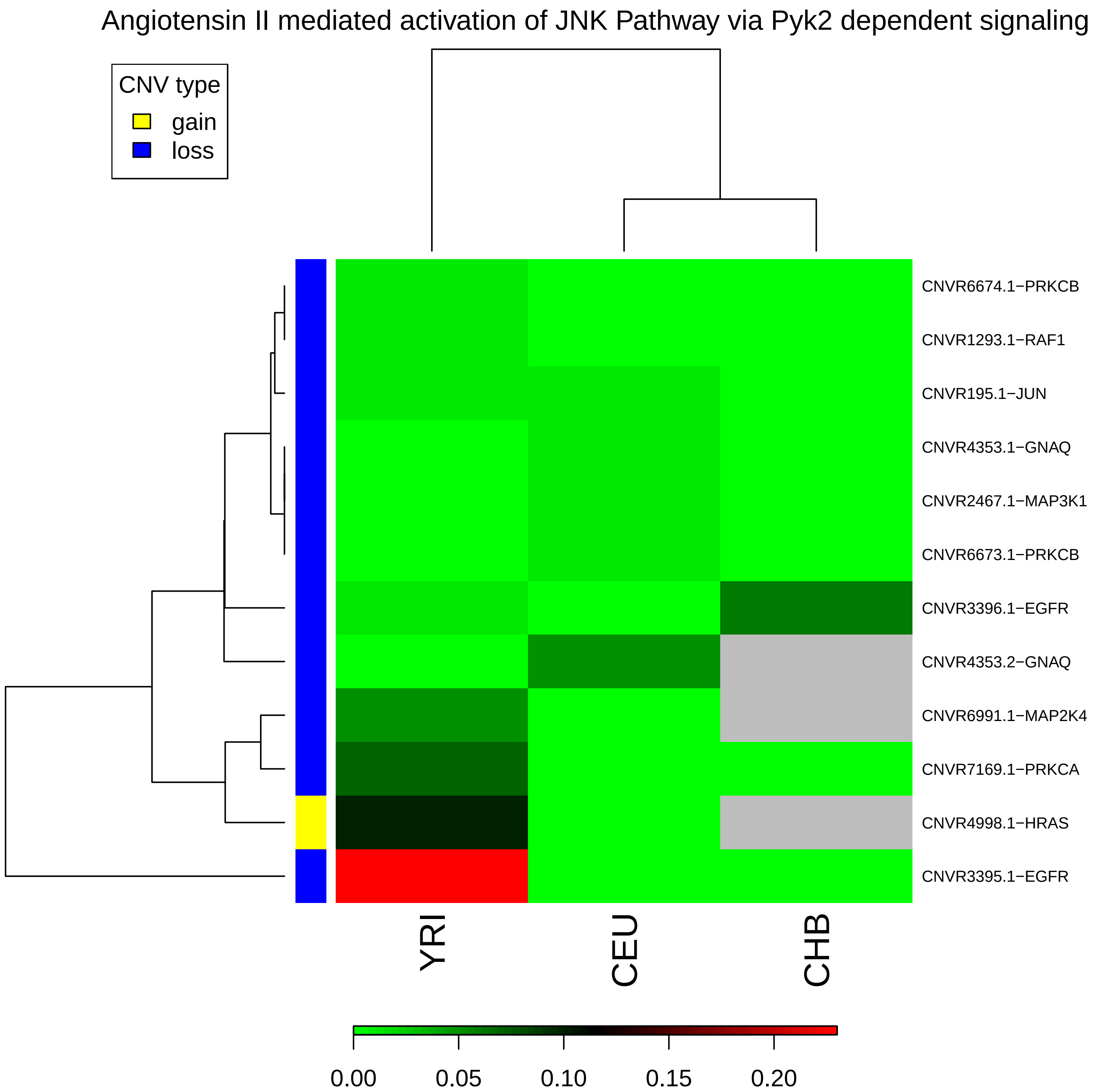

Angiotensin II mediated activation of JNK Pathway via Pyk2 dependent signaling
CNV type
gain
loss
CNVR6674.1−PRKCB
CNVR1293.1−RAF1
CNVR195.1−JUN
CNVR4353.1−GNAQ
CNVR2467.1−MAP3K1
CNVR6673.1−PRKCB
CNVR3396.1−EGFR
CNVR4353.2−GNAQ
CNVR6991.1−MAP2K4
CNVR7169.1−PRKCA
CNVR4998.1−HRAS
CNVR3395.1−EGFR
YRI
CEU
CHB
0.00
0.05
0.10
0.15
0.20

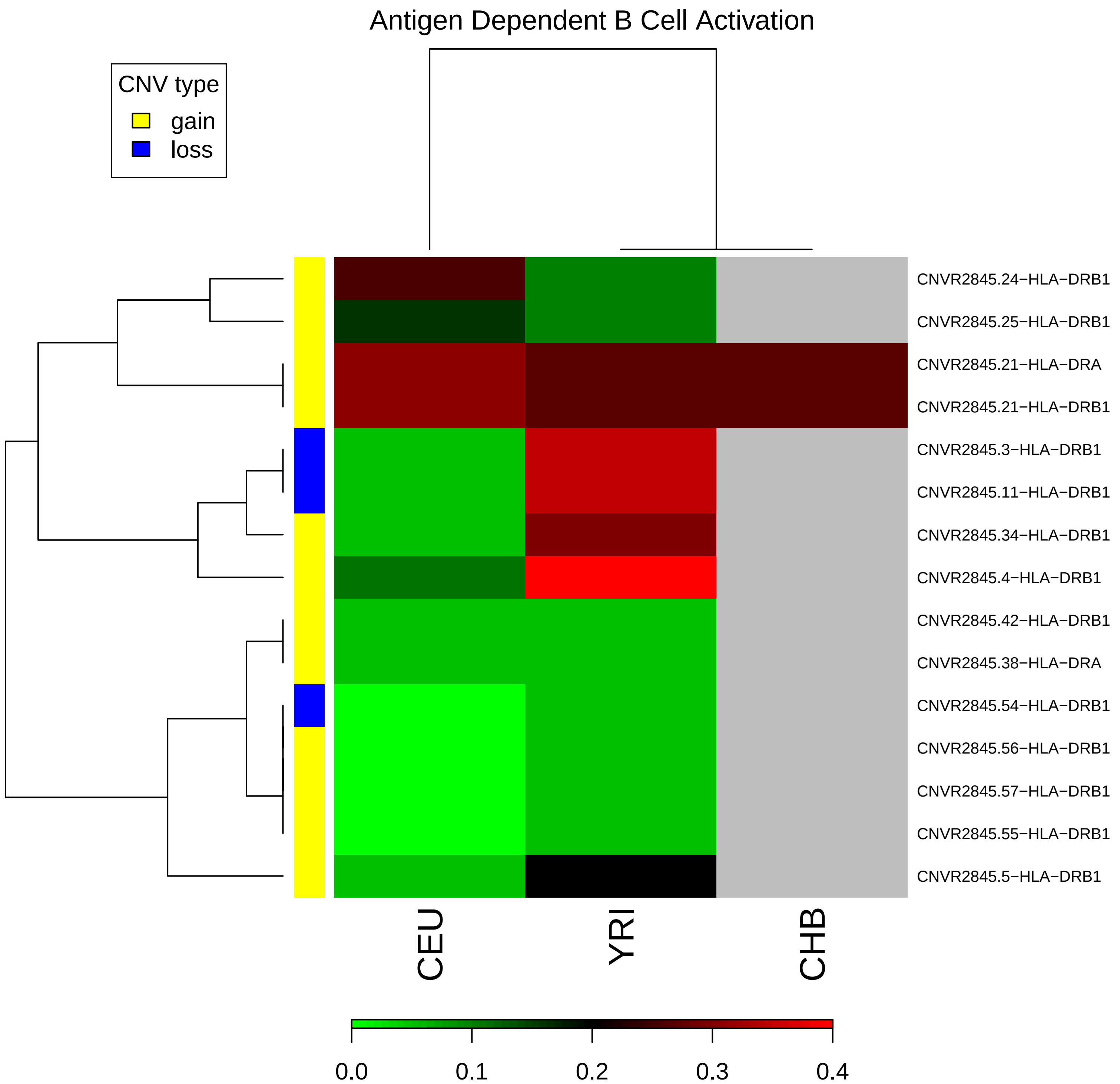
Antigen Dependent B Cell Activation
CNV type
gain
loss
CNVR2845.24−HLA−DRB1
CNVR2845.25−HLA−DRB1
CNVR2845.21−HLA−DRA
CNVR2845.21−HLA−DRB1
CNVR2845.3−HLA−DRB1
CNVR2845.11−HLA−DRB1
CNVR2845.34−HLA−DRB1
CNVR2845.4−HLA−DRB1
CNVR2845.42−HLA−DRB1
CNVR2845.38−HLA−DRA
CNVR2845.54−HLA−DRB1
CNVR2845.56−HLA−DRB1
CNVR2845.57−HLA−DRB1
CNVR2845.55−HLA−DRB1
CNVR2845.5−HLA−DRB1
CEU
YRI
CHB
0.0
0.1
0.2
0.3
0.4

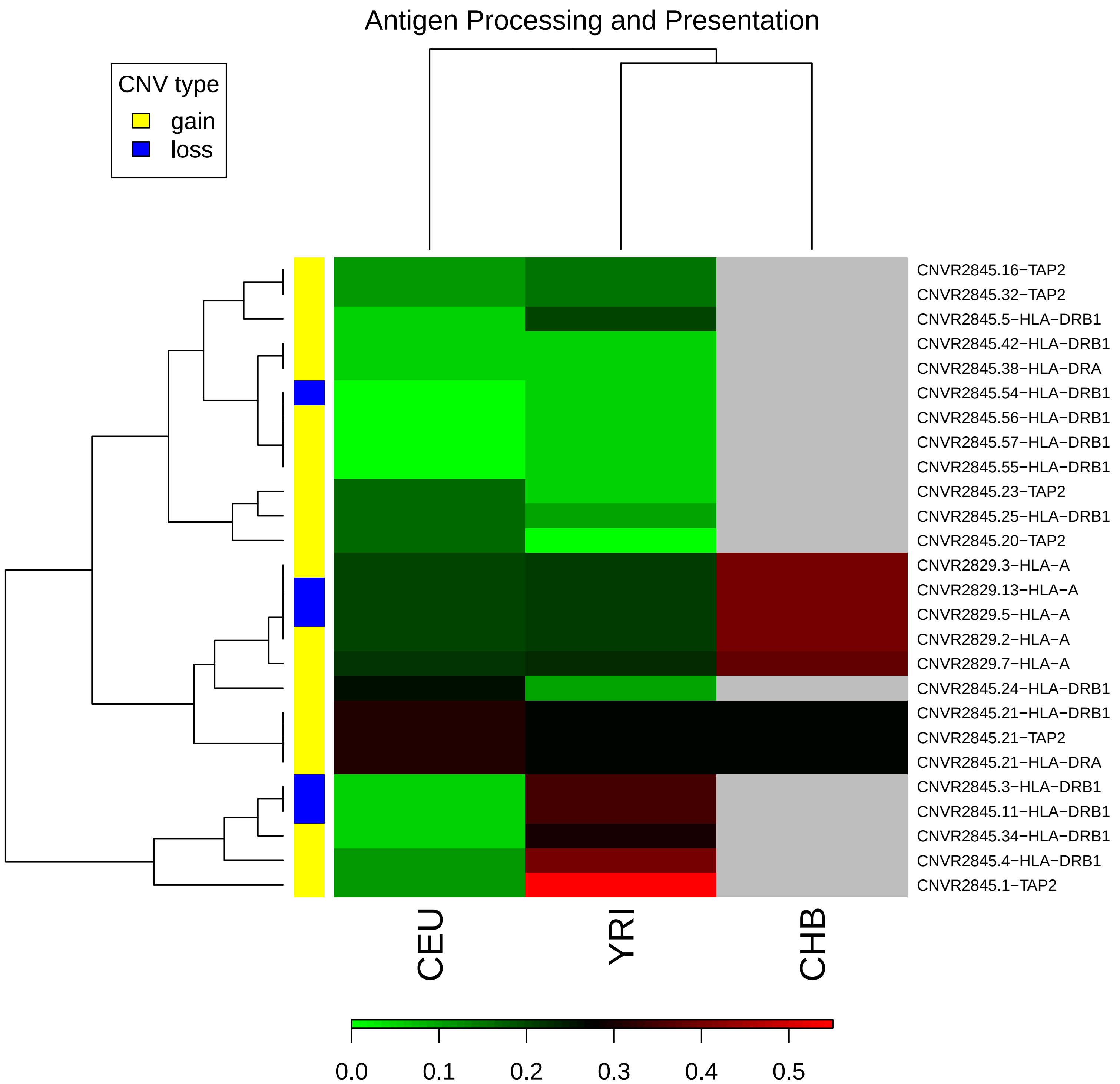
Antigen Processing and Presentation
CNV type
gain
loss
CNVR2845.16−TAP2
CNVR2845.32−TAP2
CNVR2845.5−HLA−DRB1
CNVR2845.42−HLA−DRB1
CNVR2845.38−HLA−DRA
CNVR2845.54−HLA−DRB1
CNVR2845.56−HLA−DRB1
CNVR2845.57−HLA−DRB1
CNVR2845.55−HLA−DRB1
CNVR2845.23−TAP2
CNVR2845.25−HLA−DRB1
CNVR2845.20−TAP2
CNVR2829.3−HLA−A
CNVR2829.13−HLA−A
CNVR2829.5−HLA−A
CNVR2829.2−HLA−A
CNVR2829.7−HLA−A
CNVR2845.24−HLA−DRB1
CNVR2845.21−HLA−DRB1
CNVR2845.21−TAP2
CNVR2845.21−HLA−DRA
CNVR2845.3−HLA−DRB1
CNVR2845.11−HLA−DRB1
CNVR2845.34−HLA−DRB1
CNVR2845.4−HLA−DRB1
CNVR2845.1−TAP2
CEU
YRI
CHB
0.0
0.1
0.2
0.3
0.4
0.5

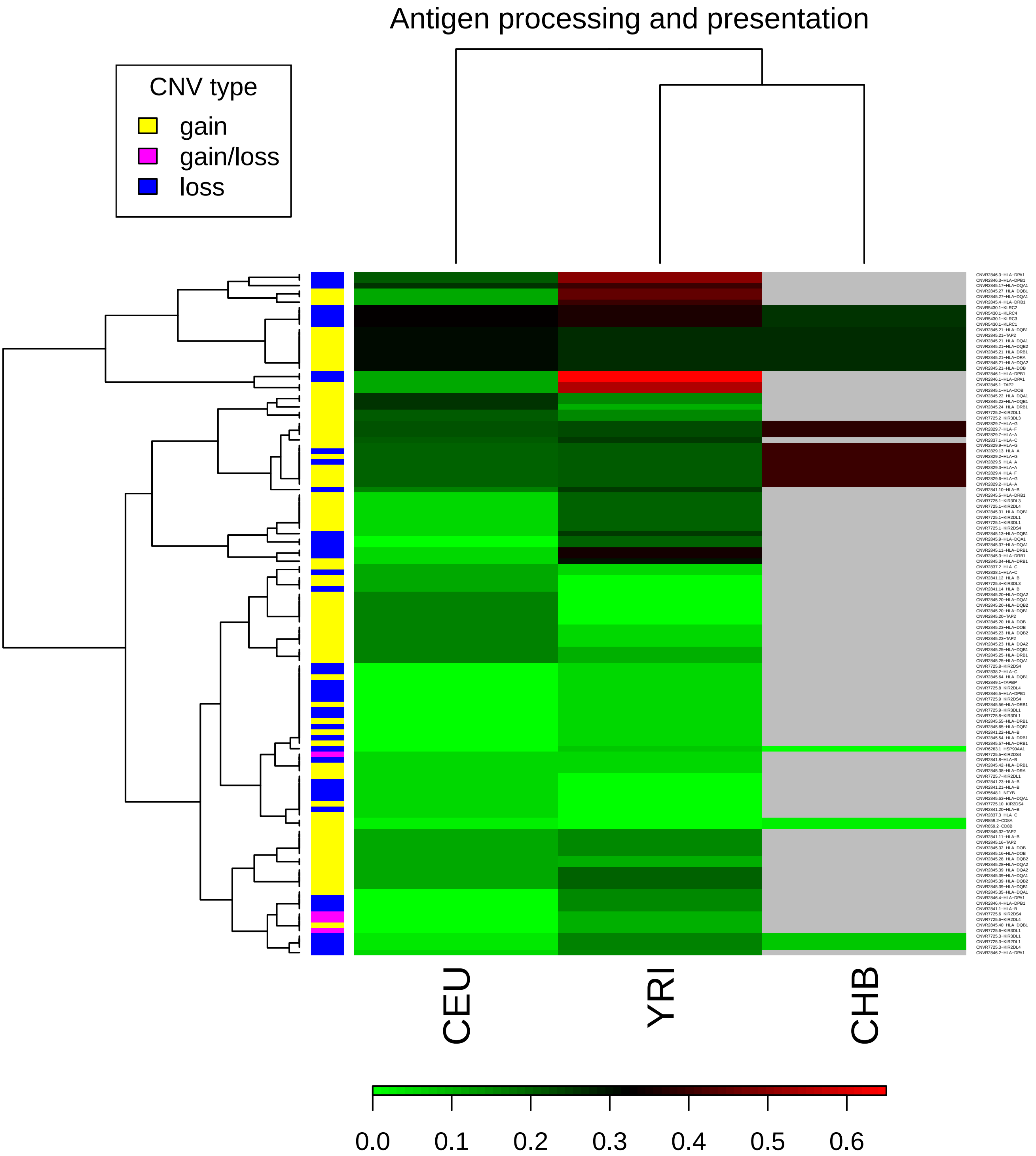

Antigen processing and presentation
CNV type
gain
gain/loss
loss
CEU
YRI
CHB
0.0
0.1
0.2
0.3
0.4
0.5
0.6

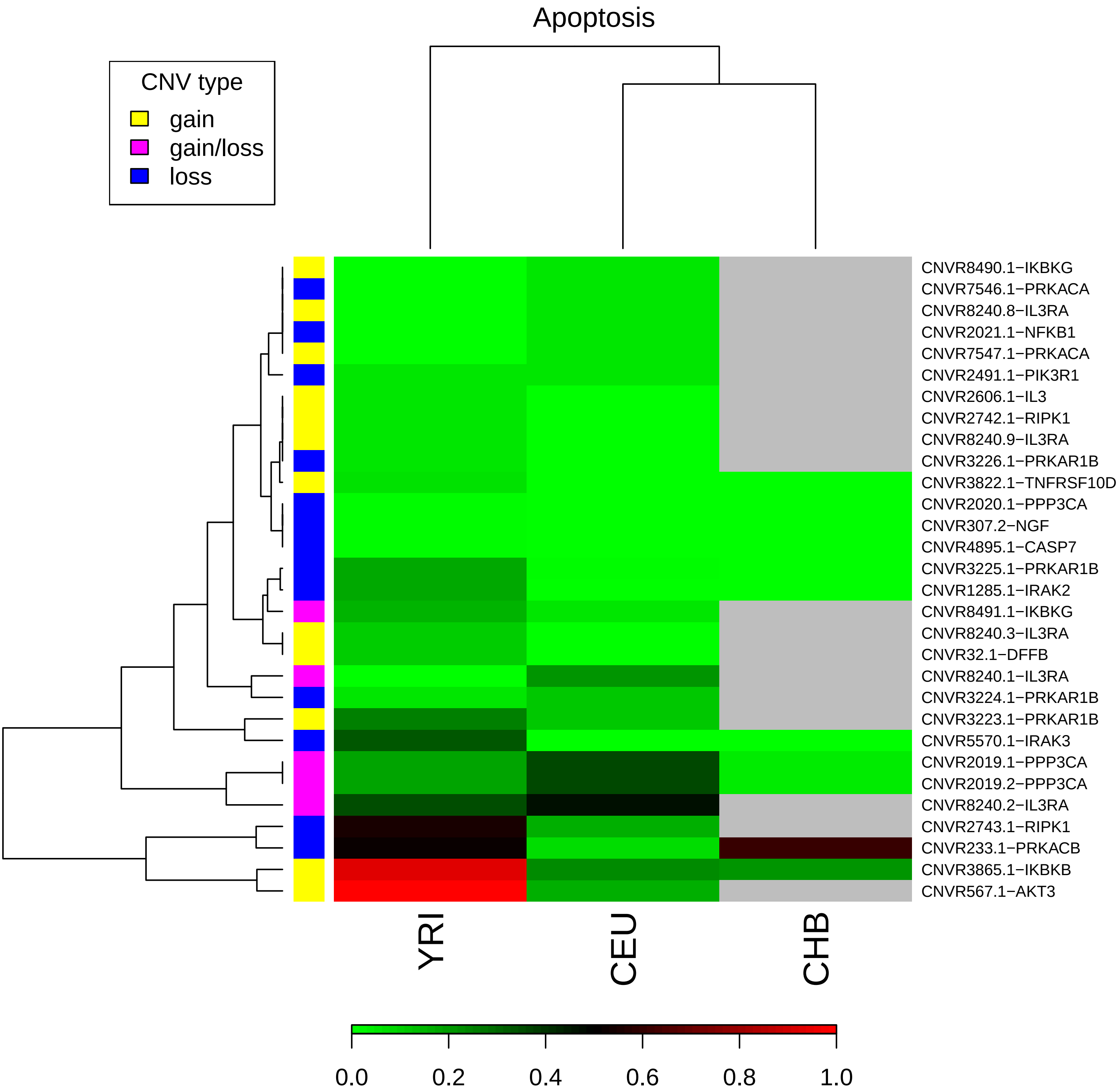
Apoptosis
CNV type
gain
gain/loss
loss
CNVR8490.1−IKBKG
CNVR7546.1−PRKACA
CNVR8240.8−IL3RA
CNVR2021.1−NFKB1
CNVR7547.1−PRKACA
CNVR2491.1−PIK3R1
CNVR2606.1−IL3
CNVR2742.1−RIPK1
CNVR8240.9−IL3RA
CNVR3226.1−PRKAR1B
CNVR3822.1−TNFRSF10D
CNVR2020.1−PPP3CA
CNVR307.2−NGF
CNVR4895.1−CASP7
CNVR3225.1−PRKAR1B
CNVR1285.1−IRAK2
CNVR8491.1−IKBKG
CNVR8240.3−IL3RA
CNVR32.1−DFFB
CNVR8240.1−IL3RA
CNVR3224.1−PRKAR1B
CNVR3223.1−PRKAR1B
CNVR5570.1−IRAK3
CNVR2019.1−PPP3CA
CNVR2019.2−PPP3CA
CNVR8240.2−IL3RA
CNVR2743.1−RIPK1
CNVR233.1−PRKACB
CNVR3865.1−IKBKB
CNVR567.1−AKT3
YRI
CEU
CHB
0.0
0.2
0.4
0.6
0.8
1.0

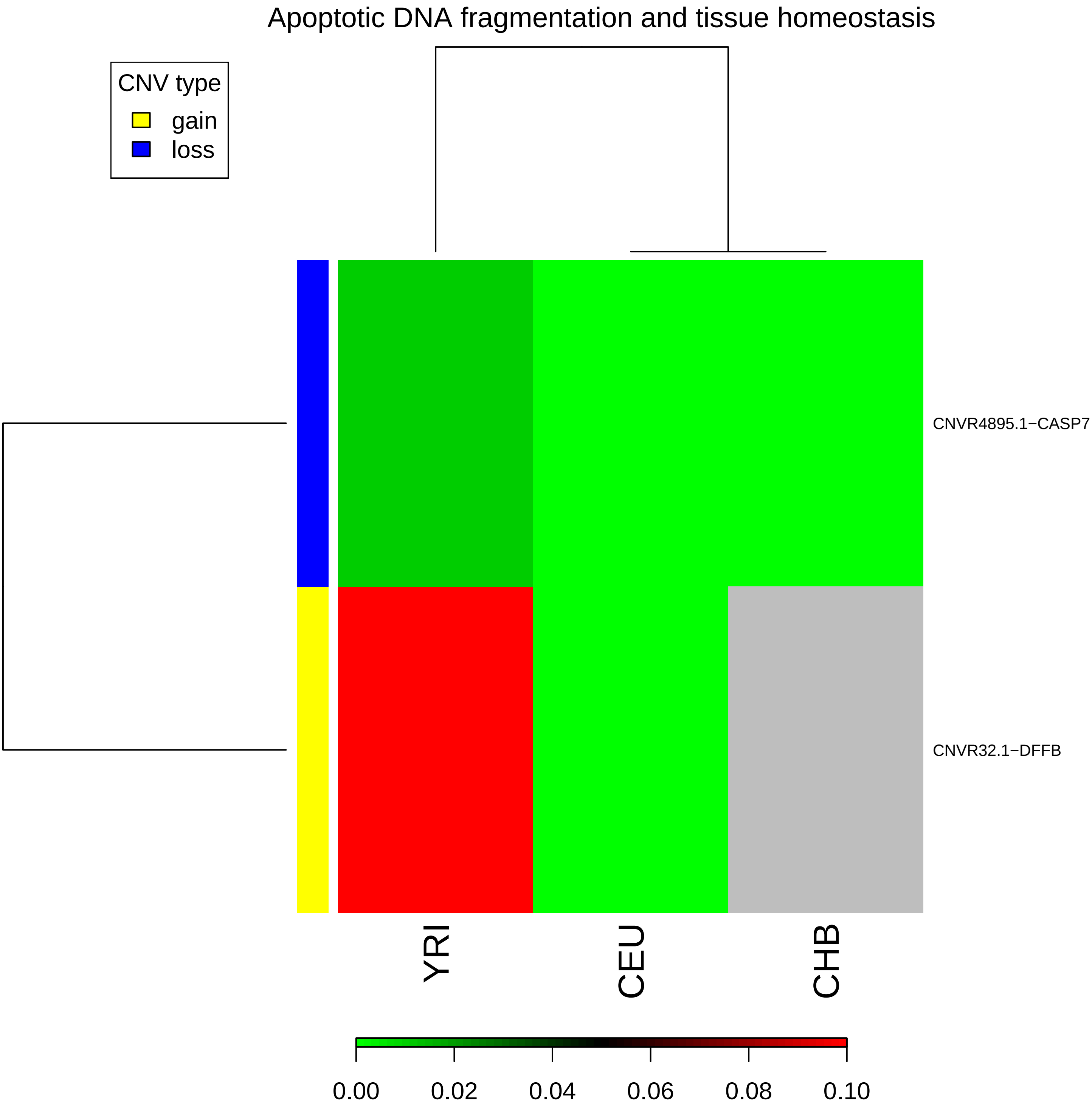
Apoptotic DNA fragmentation and tissue homeostasis
CNV type
gain
loss
CNVR4895.1−CASP7
CNVR32.1−DFFB
YRI
CEU
CHB
0.00
0.02
0.04
0.06
0.08
0.10

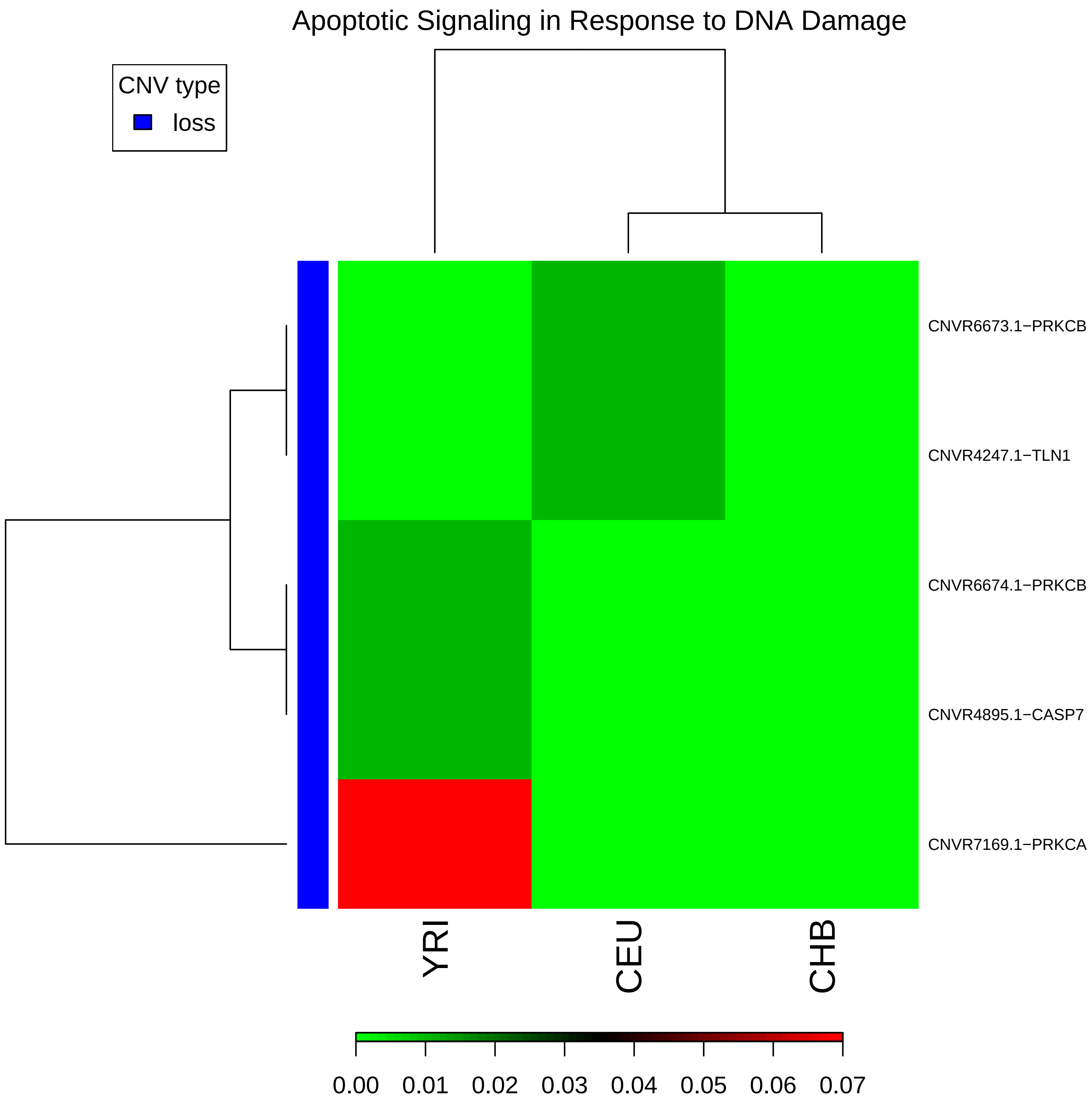

Apoptotic Signaling in Response to DNA Damage
CNV type
loss
CNVR6673.1−PRKCB
CNVR4247.1−TLN1
CNVR6674.1−PRKCB
CNVR4895.1−CASP7
CNVR7169.1−PRKCA
YRI
CEU
CHB
0.00
0.01
0.02
0.03
0.04
0.05
0.06
0.07

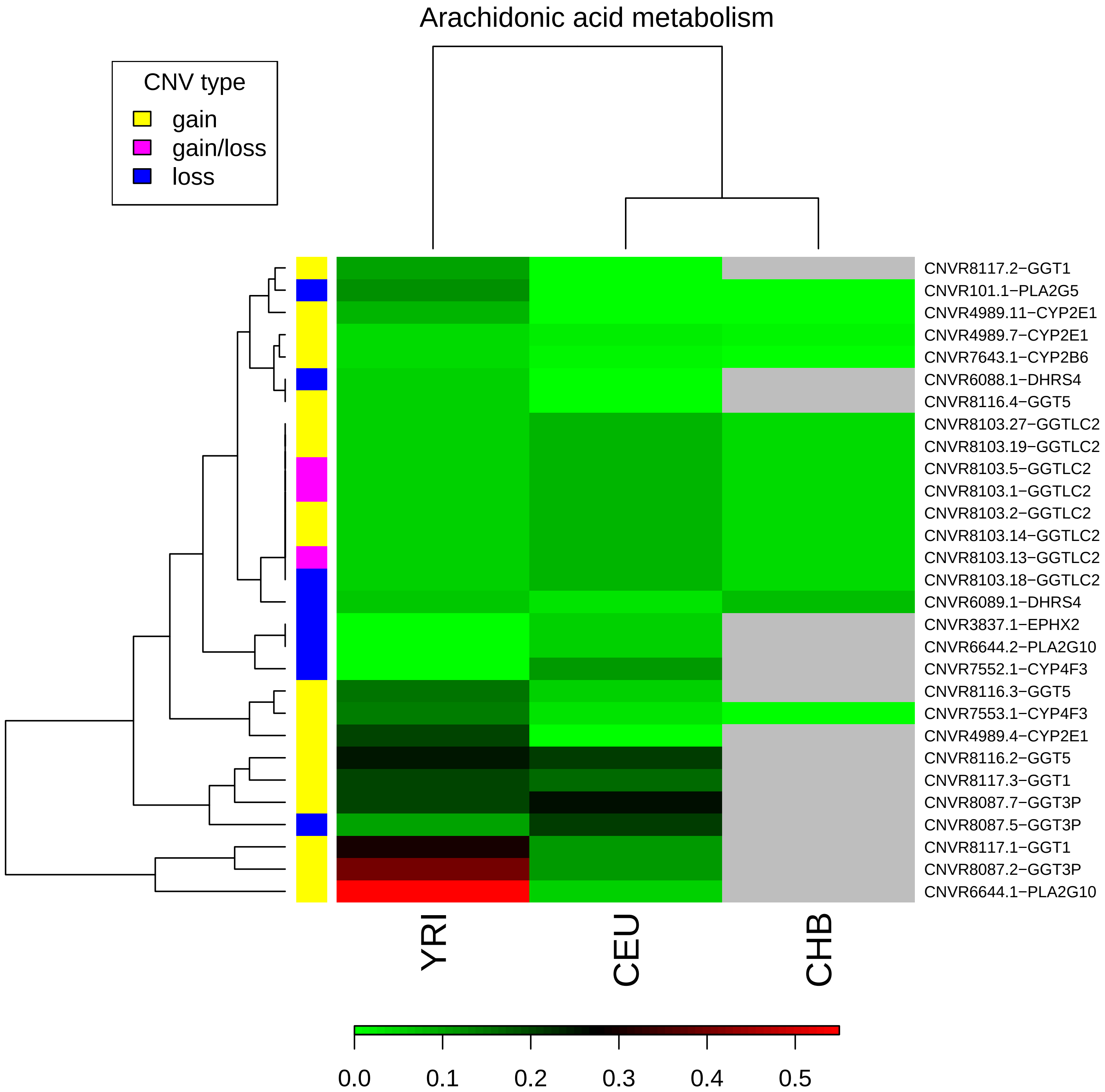

Arachidonic acid metabolism
CNV type
gain
gain/loss
loss
CNVR8117.2−GGT1
CNVR101.1−PLA2G5
CNVR4989.11−CYP2E1
CNVR4989.7−CYP2E1
CNVR7643.1−CYP2B6
CNVR6088.1−DHRS4
CNVR8116.4−GGT5
CNVR8103.27−GGTLC2
CNVR8103.19−GGTLC2
CNVR8103.5−GGTLC2
CNVR8103.1−GGTLC2
CNVR8103.2−GGTLC2
CNVR8103.14−GGTLC2
CNVR8103.13−GGTLC2
CNVR8103.18−GGTLC2
CNVR6089.1−DHRS4
CNVR3837.1−EPHX2
CNVR6644.2−PLA2G10
CNVR7552.1−CYP4F3
CNVR8116.3−GGT5
CNVR7553.1−CYP4F3
CNVR4989.4−CYP2E1
CNVR8116.2−GGT5
CNVR8117.3−GGT1
CNVR8087.7−GGT3P
CNVR8087.5−GGT3P
CNVR8117.1−GGT1
CNVR8087.2−GGT3P
CNVR6644.1−PLA2G10
YRI
CEU
CHB
0.0
0.1
0.2
0.3
0.4
0.5

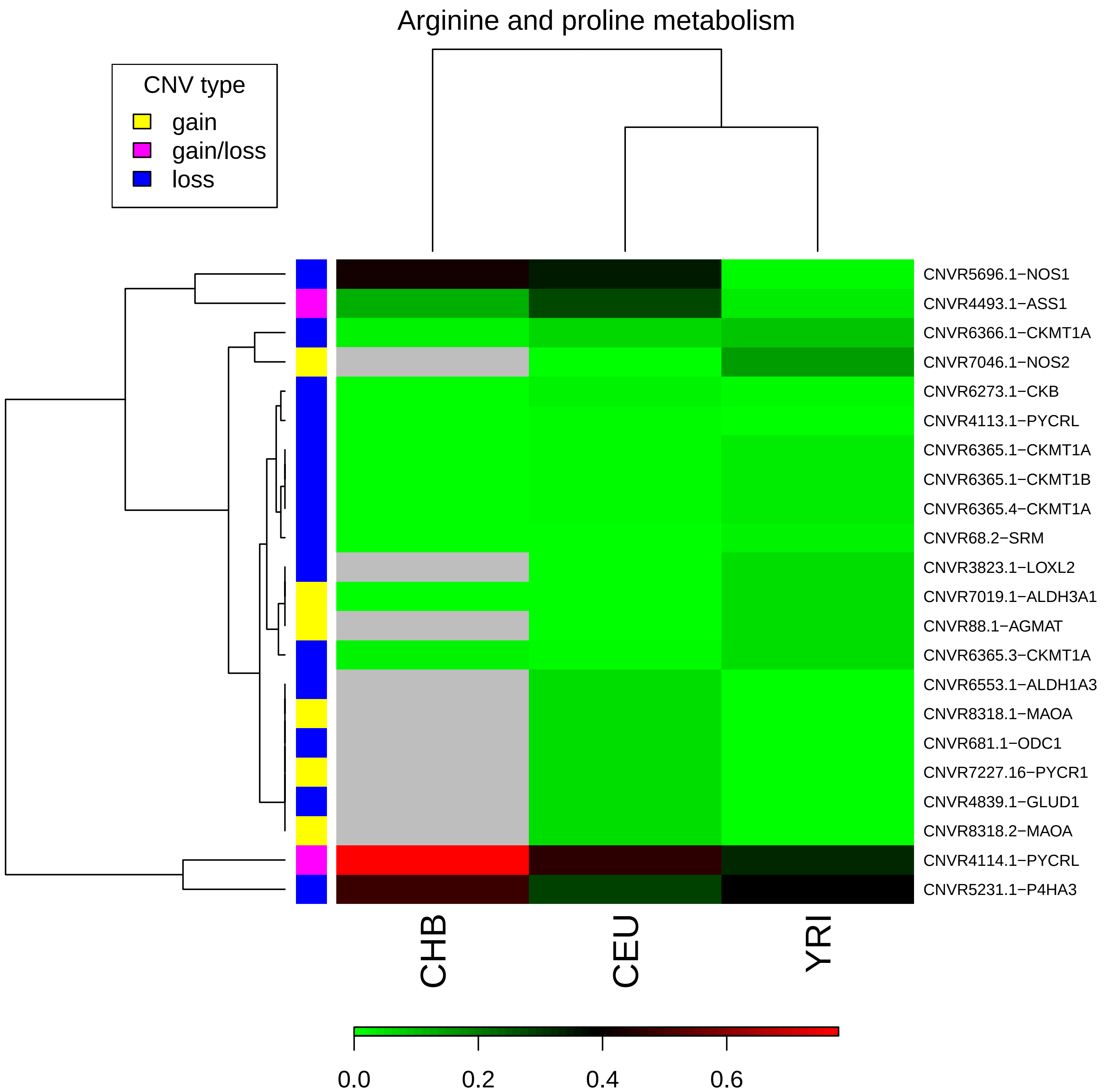
Arginine and proline metabolism
CNV type
gain
gain/loss
loss
CNVR5696.1−NOS1
CNVR4493.1−ASS1
CNVR6366.1−CKMT1A
CNVR7046.1−NOS2
CNVR6273.1−CKB
CNVR4113.1−PYCRL
CNVR6365.1−CKMT1A
CNVR6365.1−CKMT1B
CNVR6365.4−CKMT1A
CNVR68.2−SRM
CNVR3823.1−LOXL2
CNVR7019.1−ALDH3A1
CNVR88.1−AGMAT
CNVR6365.3−CKMT1A
CNVR6553.1−ALDH1A3
CNVR8318.1−MAOA
CNVR681.1−ODC1
CNVR7227.16−PYCR1
CNVR4839.1−GLUD1
CNVR8318.2−MAOA
CNVR4114.1−PYCRL
CNVR5231.1−P4HA3
CHB
CEU
YRI
0.0
0.2
0.4
0.6

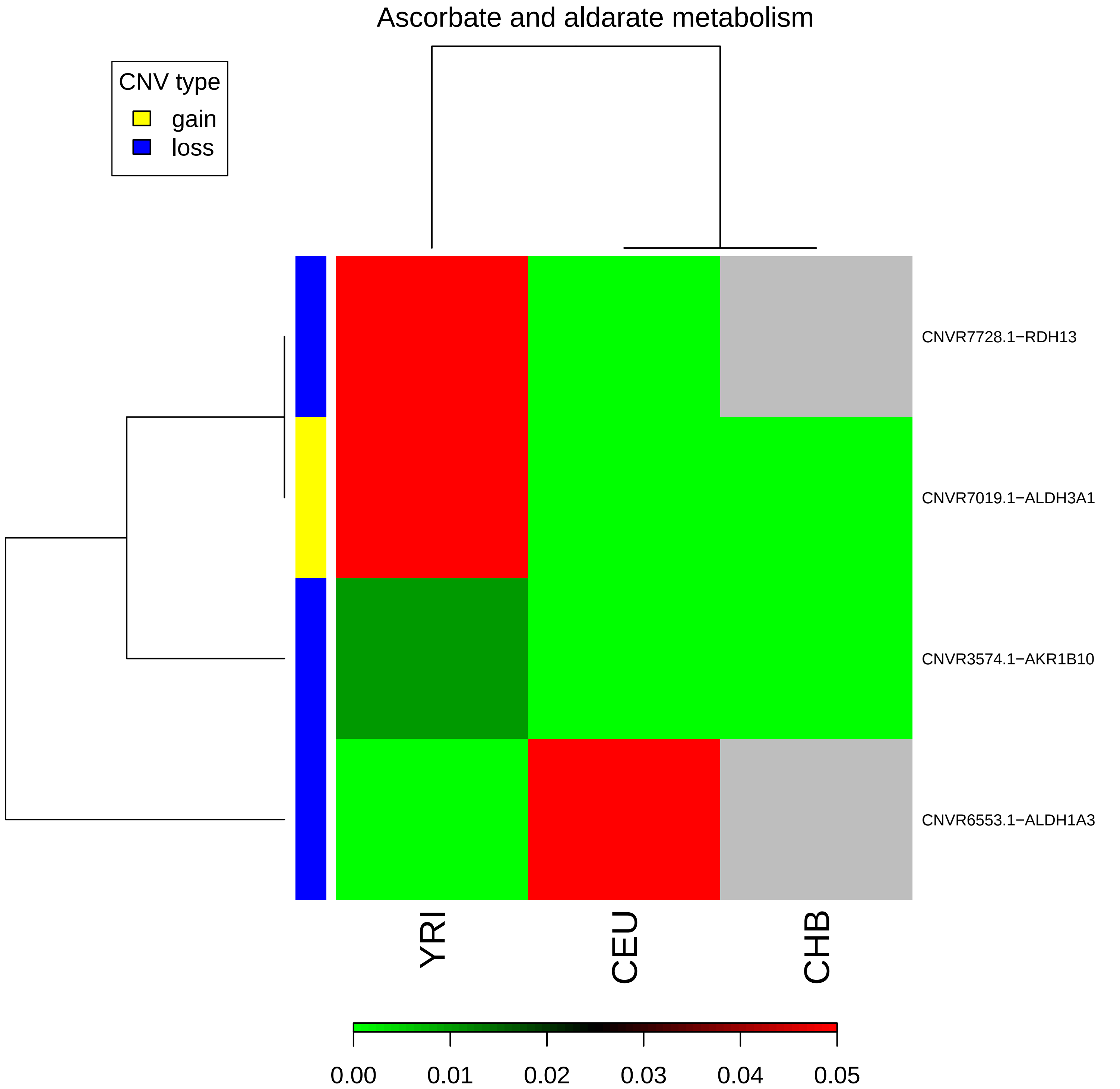
Ascorbate and aldarate metabolism
CNV type
gain
loss
CNVR7728.1−RDH13
CNVR7019.1−ALDH3A1
CNVR3574.1−AKR1B10
CNVR6553.1−ALDH1A3
YRI
CEU
CHB
0.00
0.01
0.02
0.03
0.04
0.05

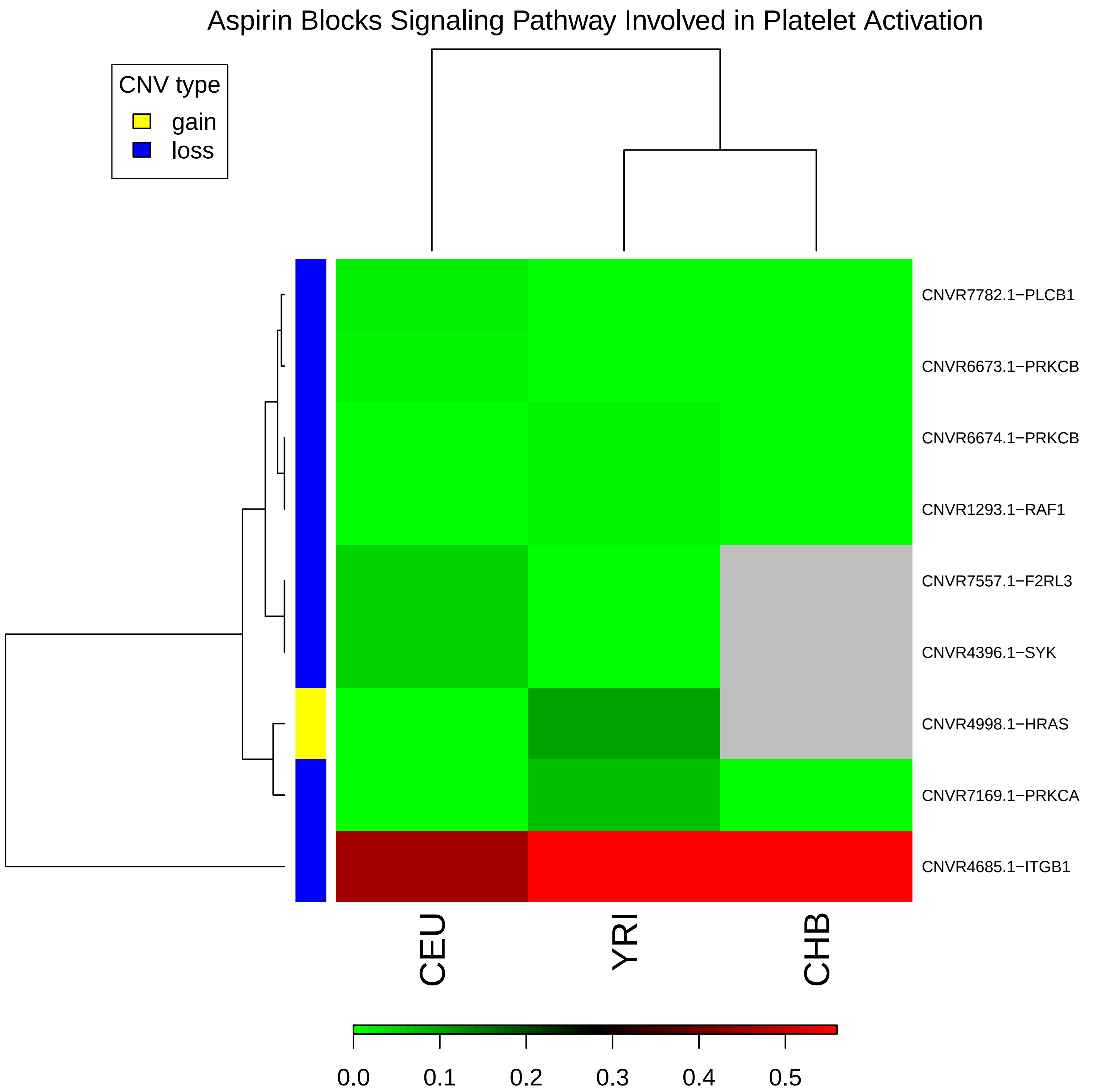
Aspirin Blocks Signaling Pathway Involved in Platelet Activation
CNV type
gain
loss
CNVR7782.1−PLCB1
CNVR6673.1−PRKCB
CNVR6674.1−PRKCB
CNVR1293.1−RAF1
CNVR7557.1−F2RL3
CNVR4396.1−SYK
CNVR4998.1−HRAS
CNVR7169.1−PRKCA
CNVR4685.1−ITGB1
CEU
YRI
CHB
0.0
0.1
0.2
0.3
0.4
0.5

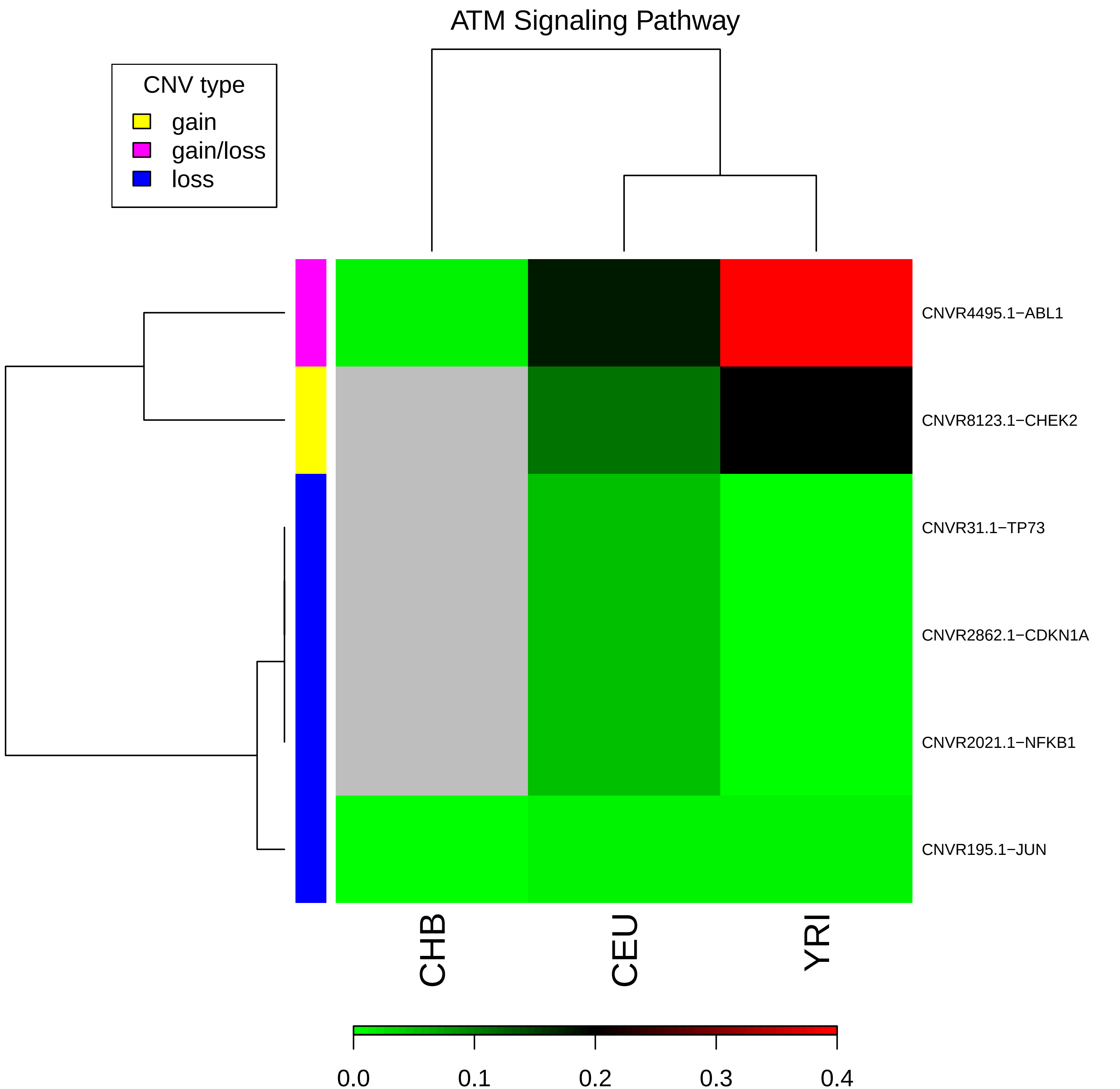
ATM Signaling Pathway
CNV type
gain
gain/loss
loss
CNVR4495.1−ABL1
CNVR8123.1−CHEK2
CNVR31.1−TP73
CNVR2862.1−CDKN1A
CNVR2021.1−NFKB1
CNVR195.1−JUN
CHB
CEU
YRI
0.0
0.1
0.2
0.3
0.4

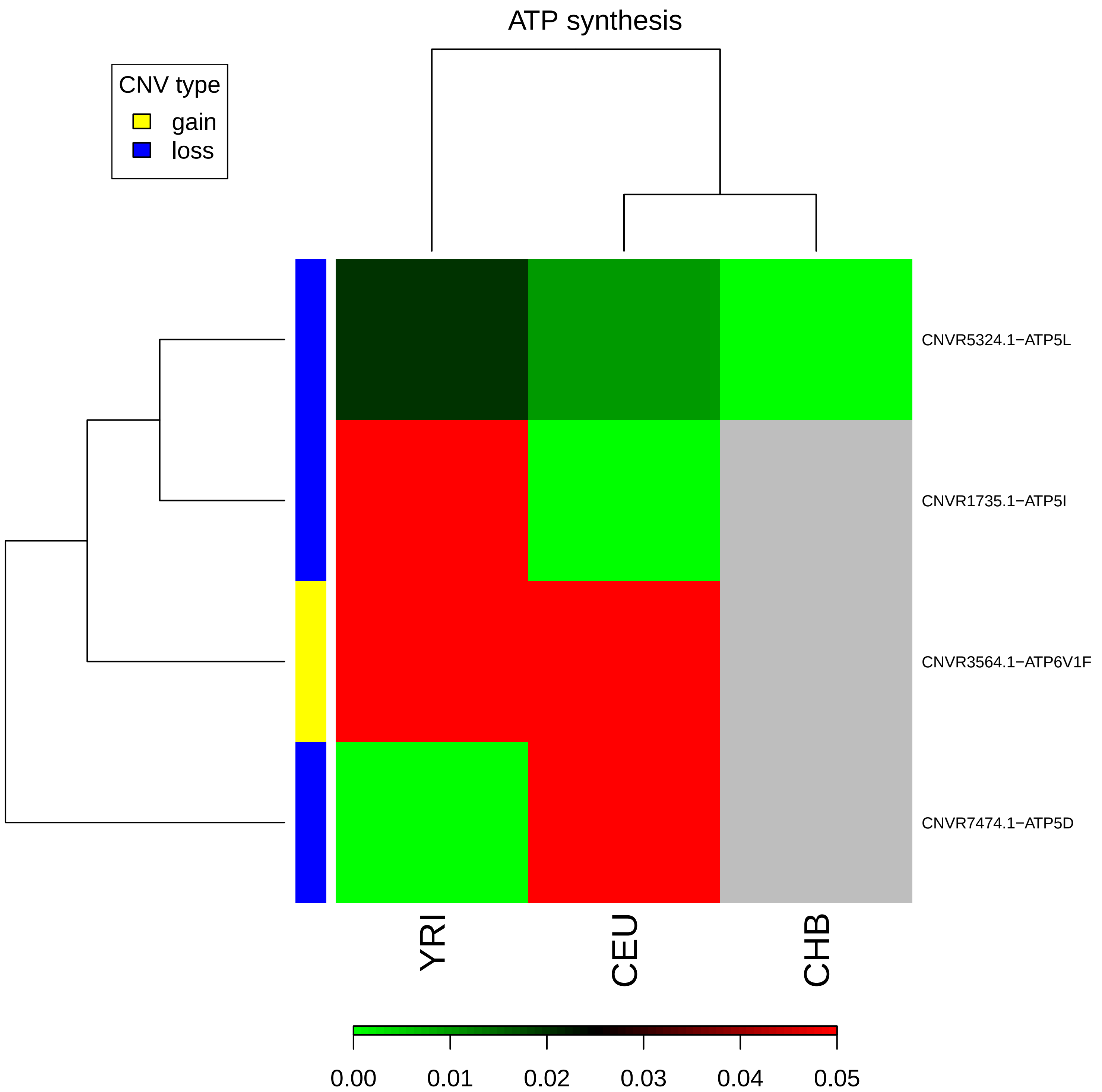
ATP synthesis
CNV type
gain
loss
CNVR5324.1−ATP5L
CNVR1735.1−ATP5I
CNVR3564.1−ATP6V1F
CNVR7474.1−ATP5D
YRI
CEU
CHB
0.00
0.01
0.02
0.03
0.04
0.05

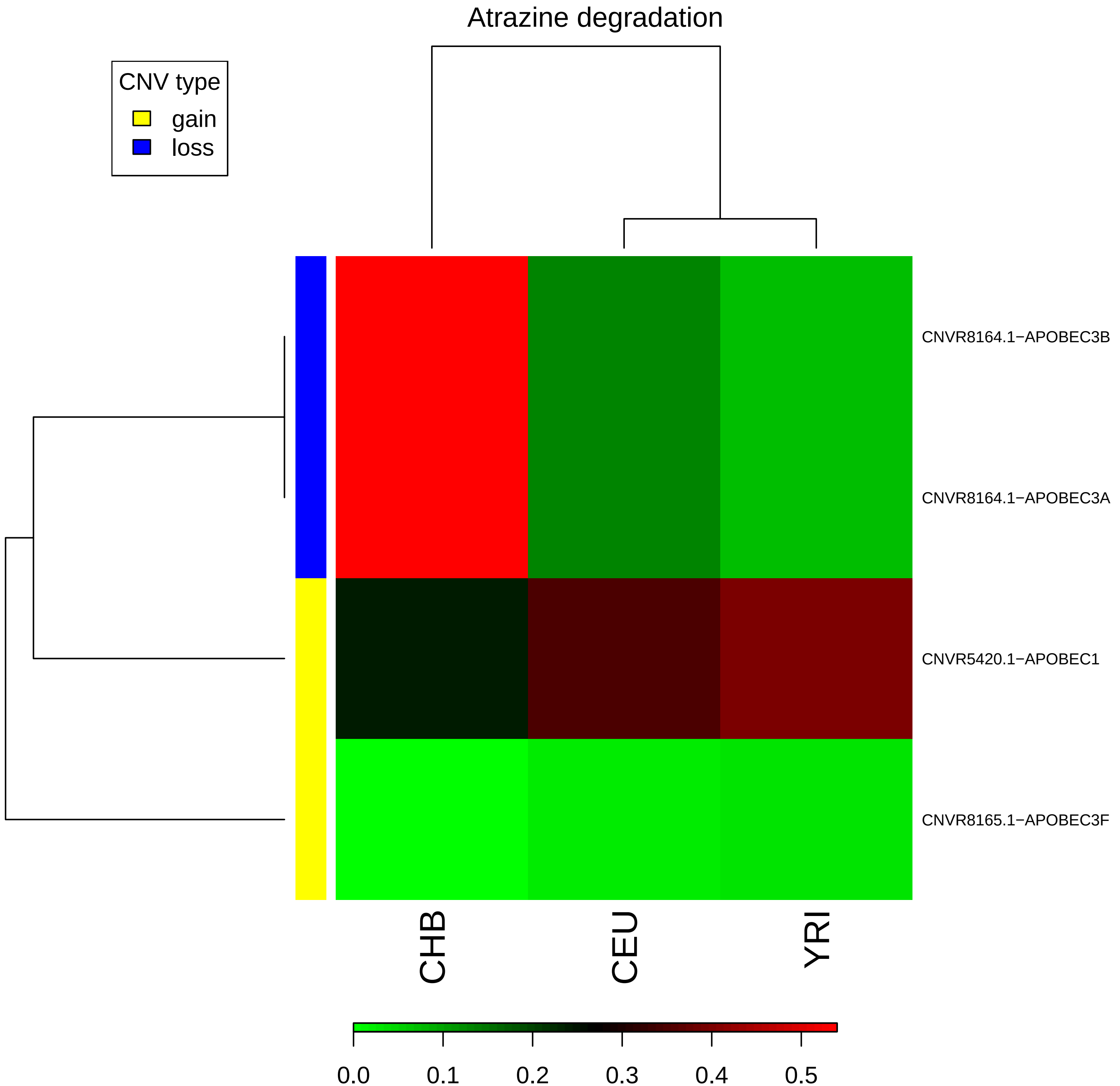

Atrazine degradation
CNV type
gain
loss
CNVR8164.1−APOBEC3B
CNVR8164.1−APOBEC3A
CNVR5420.1−APOBEC1
CNVR8165.1−APOBEC3F
CHB
CEU
YRI
0.0
0.1
0.2
0.3
0.4
0.5

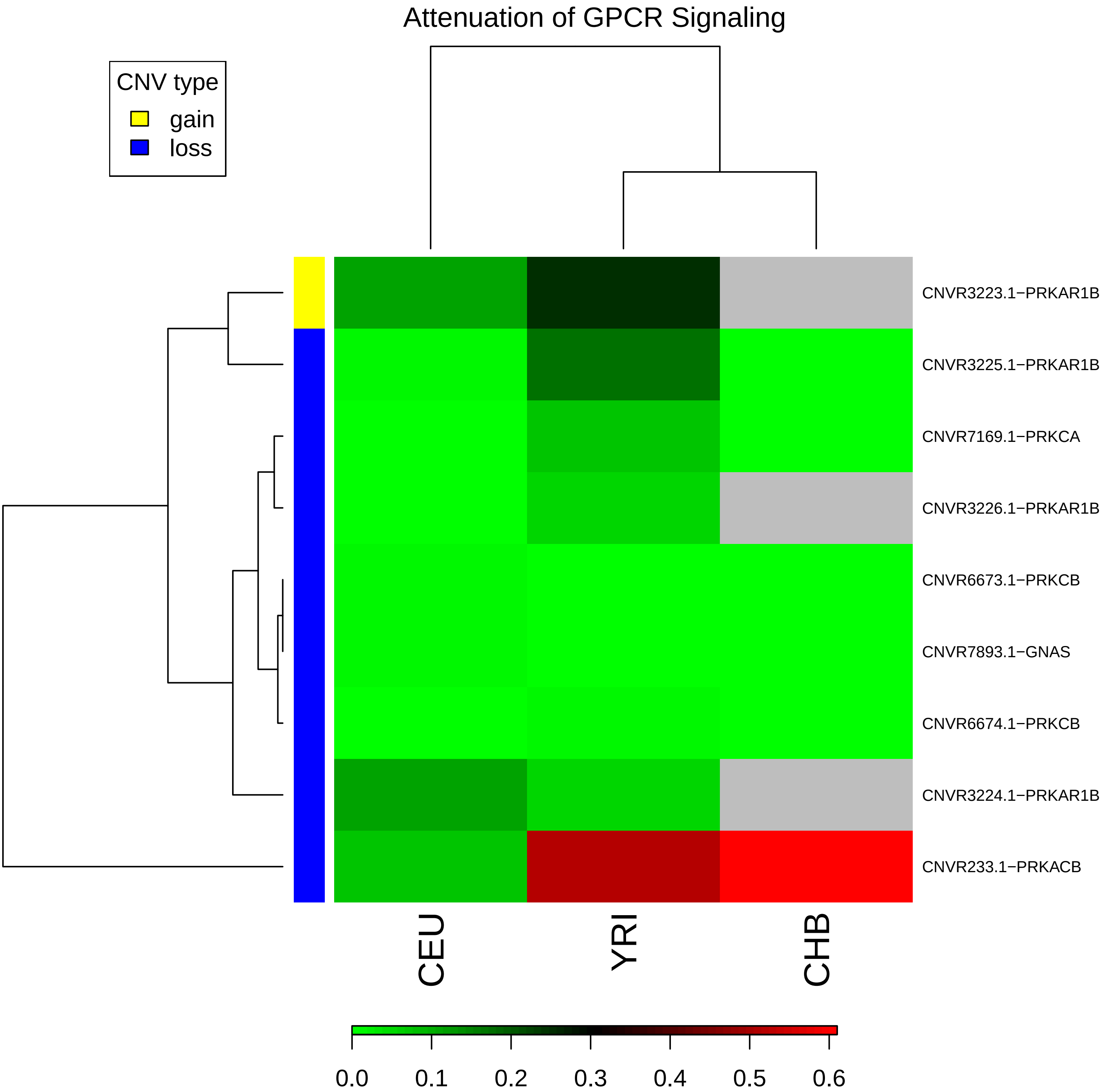
Attenuation of GPCR Signaling
CNV type
gain
loss
CNVR3223.1−PRKAR1B
CNVR3225.1−PRKAR1B
CNVR7169.1−PRKCA
CNVR3226.1−PRKAR1B
CNVR6673.1−PRKCB
CNVR7893.1−GNAS
CNVR6674.1−PRKCB
CNVR3224.1−PRKAR1B
CNVR233.1−PRKACB
CEU
YRI
CHB
0.0
0.1
0.2
0.3
0.4
0.5
0.6

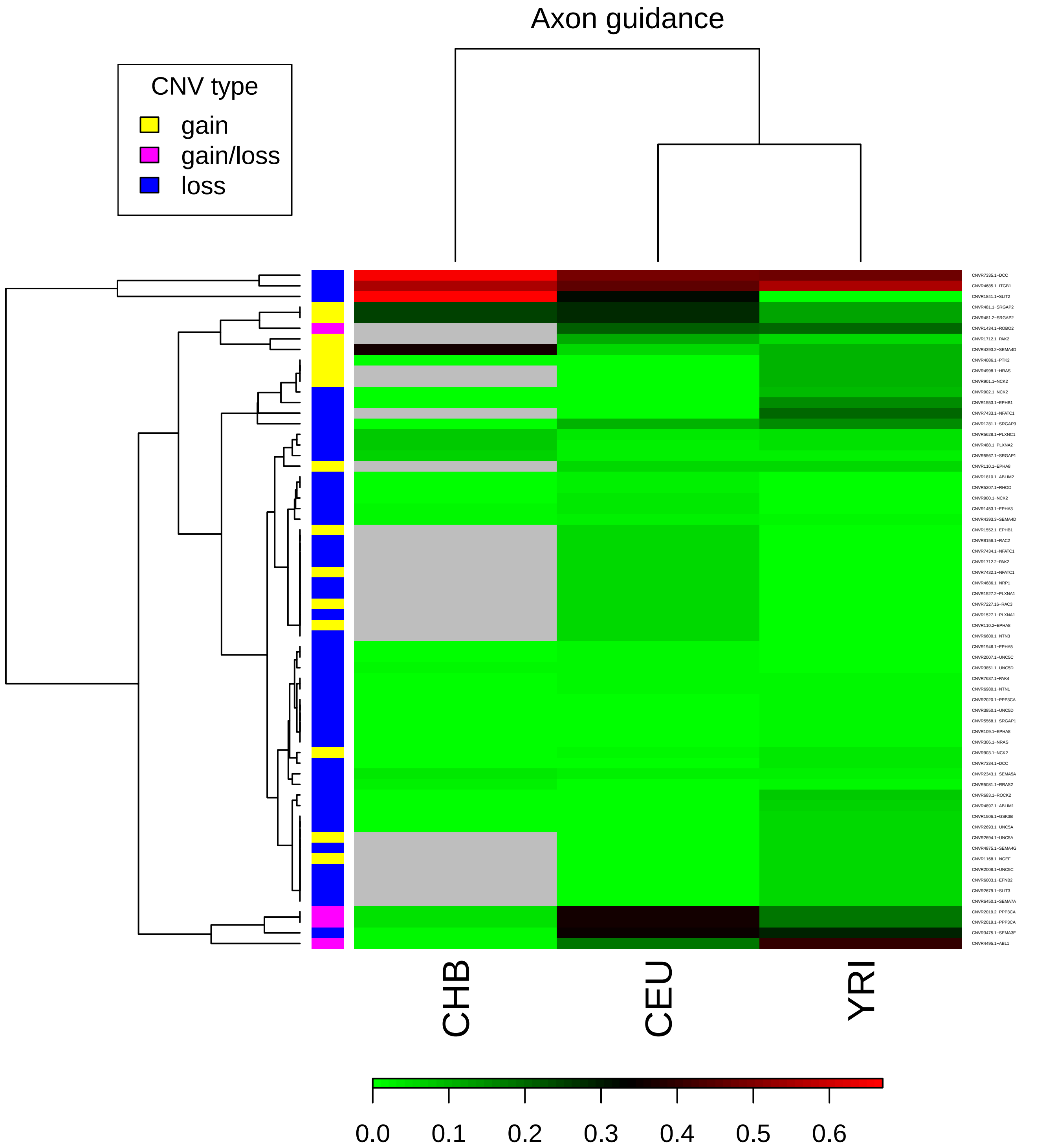
Axon guidance
CNV type
gain
gain/loss
loss
CNVR7335.1−DCC
CNVR4685.1−ITGB1
CNVR1841.1−SLIT2
CNVR481.1−SRGAP2
CNVR481.2−SRGAP2
CNVR1434.1−ROBO2
CNVR1712.1−PAK2
CNVR4393.2−SEMA4D
CNVR4086.1−PTK2
CNVR4998.1−HRAS
CNVR901.1−NCK2
CNVR902.1−NCK2
CNVR1553.1−EPHB1
CNVR7433.1−NFATC1
CNVR1281.1−SRGAP3
CNVR5628.1−PLXNC1
CNVR488.1−PLXNA2
CNVR5567.1−SRGAP1
CNVR110.1−EPHA8
CNVR1810.1−ABLIM2
CNVR5207.1−RHOD
CNVR900.1−NCK2
CNVR1453.1−EPHA3
CNVR4393.3−SEMA4D
CNVR1552.1−EPHB1
CNVR8156.1−RAC2
CNVR7434.1−NFATC1
CNVR1712.2−PAK2
CNVR7432.1−NFATC1
CNVR4686.1−NRP1
CNVR1527.2−PLXNA1
CNVR7227.16−RAC3
CNVR1527.1−PLXNA1
CNVR110.2−EPHA8
CNVR6600.1−NTN3
CNVR1946.1−EPHA5
CNVR2007.1−UNC5C
CNVR3851.1−UNC5D
CNVR7637.1−PAK4
CNVR6980.1−NTN1
CNVR2020.1−PPP3CA
CNVR3850.1−UNC5D
CNVR5568.1−SRGAP1
CNVR109.1−EPHA8
CNVR306.1−NRAS
CNVR903.1−NCK2
CNVR7334.1−DCC
CNVR2343.1−SEMA5A
CNVR5081.1−RRAS2
CNVR683.1−ROCK2
CNVR4897.1−ABLIM1
CNVR1506.1−GSK3B
CNVR2693.1−UNC5A
CNVR2694.1−UNC5A
CNVR4875.1−SEMA4G
CNVR1168.1−NGEF
CNVR2008.1−UNC5C
CNVR6003.1−EFNB2
CNVR2679.1−SLIT3
CNVR6450.1−SEMA7A
CNVR2019.2−PPP3CA
CNVR2019.1−PPP3CA
CNVR3475.1−SEMA3E
CNVR4495.1−ABL1
CHB
CEU
YRI
0.0
0.1
0.2
0.3
0.4
0.5
0.6

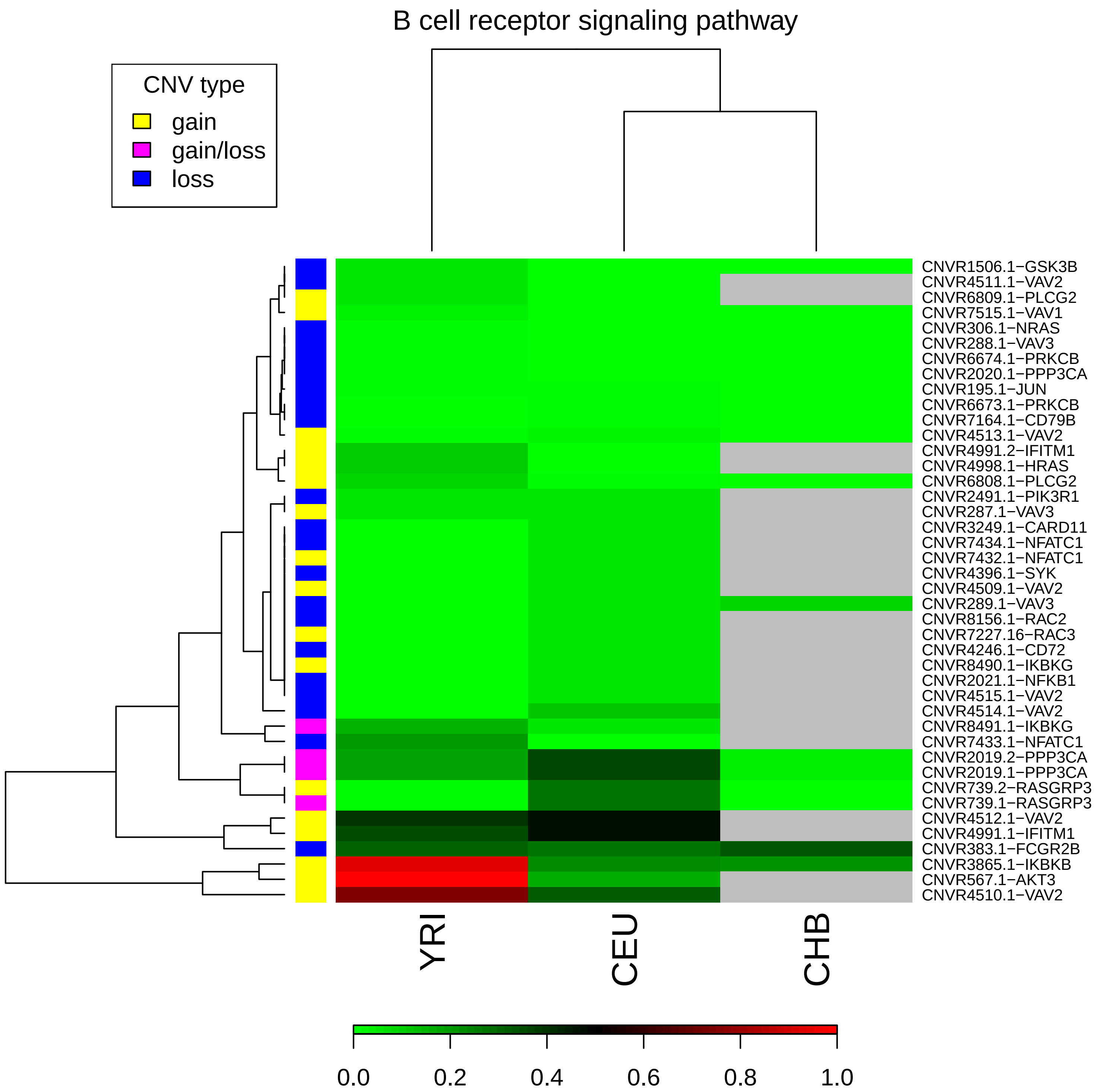
B cell receptor signaling pathway
CNV type
gain
gain/loss
loss
CNVR1506.1−GSK3B
CNVR4511.1−VAV2
CNVR6809.1−PLCG2
CNVR7515.1−VAV1
CNVR306.1−NRAS
CNVR288.1−VAV3
CNVR6674.1−PRKCB
CNVR2020.1−PPP3CA
CNVR195.1−JUN
CNVR6673.1−PRKCB
CNVR7164.1−CD79B
CNVR4513.1−VAV2
CNVR4991.2−IFITM1
CNVR4998.1−HRAS
CNVR6808.1−PLCG2
CNVR2491.1−PIK3R1
CNVR287.1−VAV3
CNVR3249.1−CARD11
CNVR7434.1−NFATC1
CNVR7432.1−NFATC1
CNVR4396.1−SYK
CNVR4509.1−VAV2
CNVR289.1−VAV3
CNVR8156.1−RAC2
CNVR7227.16−RAC3
CNVR4246.1−CD72
CNVR8490.1−IKBKG
CNVR2021.1−NFKB1
CNVR4515.1−VAV2
CNVR4514.1−VAV2
CNVR8491.1−IKBKG
CNVR7433.1−NFATC1
CNVR2019.2−PPP3CA
CNVR2019.1−PPP3CA
CNVR739.2−RASGRP3
CNVR739.1−RASGRP3
CNVR4512.1−VAV2
CNVR4991.1−IFITM1
CNVR383.1−FCGR2B
CNVR3865.1−IKBKB
CNVR567.1−AKT3
CNVR4510.1−VAV2
YRI
CEU
CHB
0.0
0.2
0.4
0.6
0.8
1.0

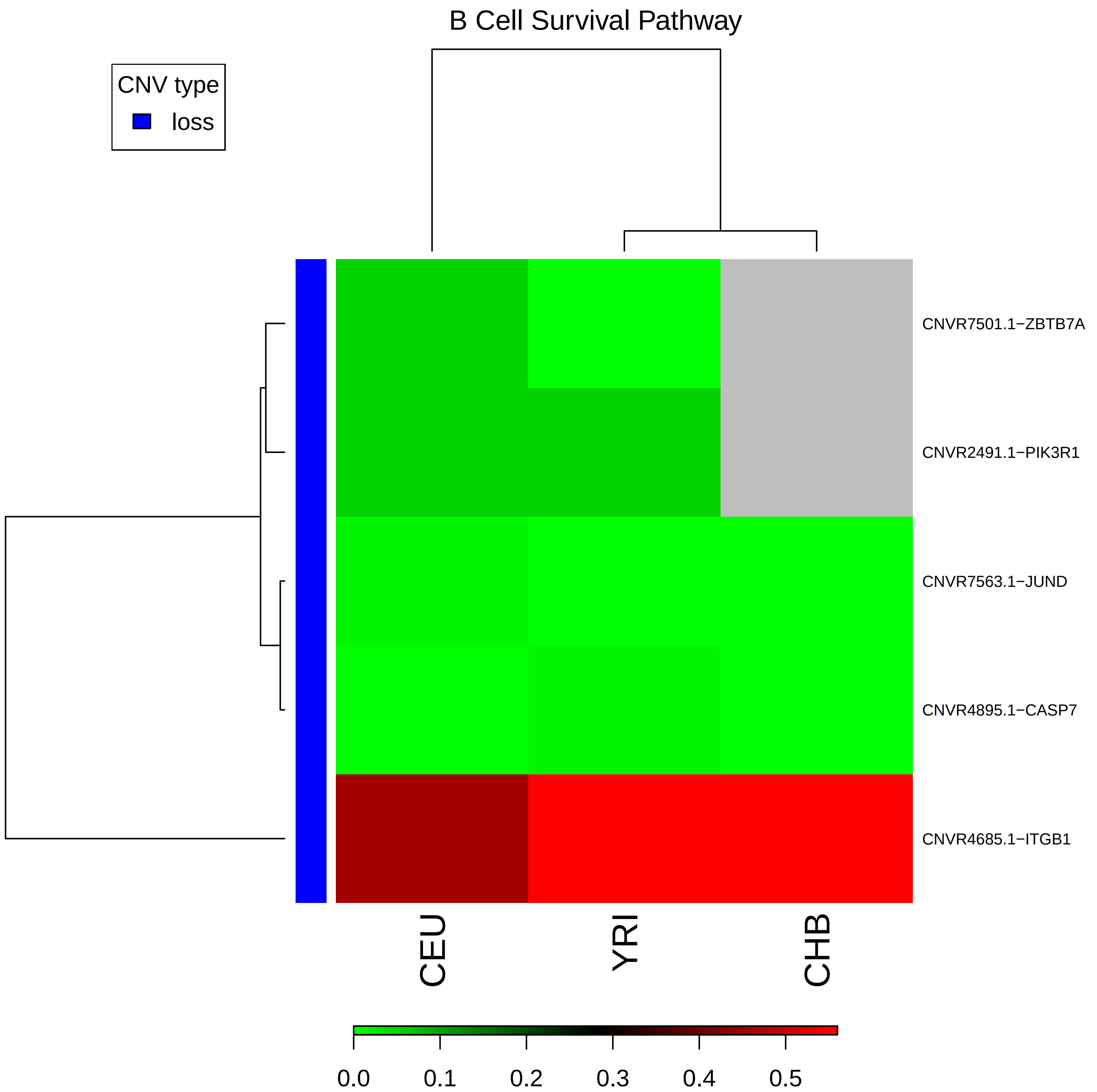
B Cell Survival Pathway
CNV type
loss
CNVR7501.1−ZBTB7A
CNVR2491.1−PIK3R1
CNVR7563.1−JUND
CNVR4895.1−CASP7
CNVR4685.1−ITGB1
CEU
YRI
CHB
0.0
0.1
0.2
0.3
0.4
0.5

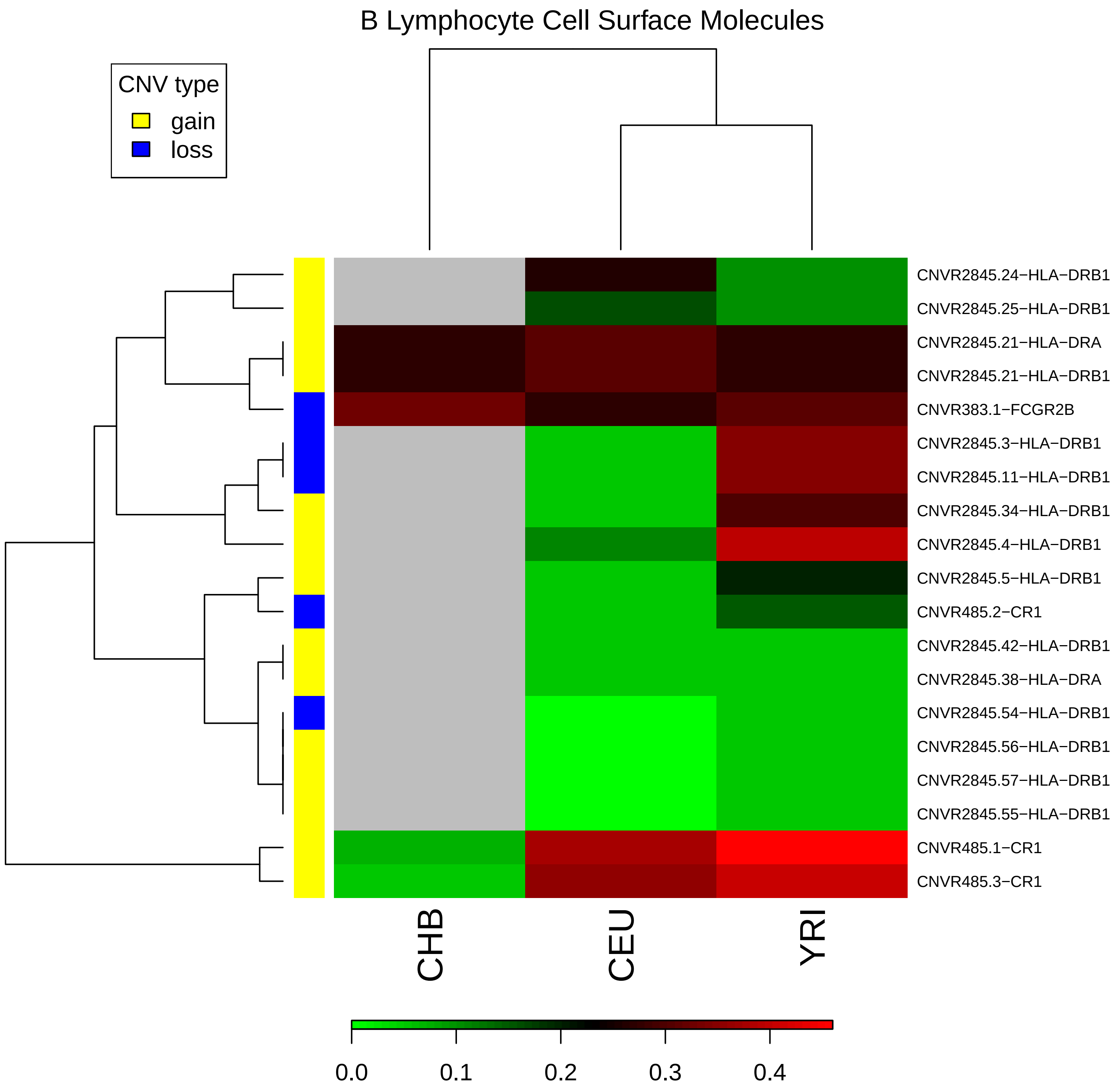

B Lymphocyte Cell Surface Molecules
CNV type
gain
loss
CNVR2845.24−HLA−DRB1
CNVR2845.25−HLA−DRB1
CNVR2845.21−HLA−DRA
CNVR2845.21−HLA−DRB1
CNVR383.1−FCGR2B
CNVR2845.3−HLA−DRB1
CNVR2845.11−HLA−DRB1
CNVR2845.34−HLA−DRB1
CNVR2845.4−HLA−DRB1
CNVR2845.5−HLA−DRB1
CNVR485.2−CR1
CNVR2845.42−HLA−DRB1
CNVR2845.38−HLA−DRA
CNVR2845.54−HLA−DRB1
CNVR2845.56−HLA−DRB1
CNVR2845.57−HLA−DRB1
CNVR2845.55−HLA−DRB1
CNVR485.1−CR1
CNVR485.3−CR1
CHB
CEU
YRI
0.0
0.1
0.2
0.3
0.4

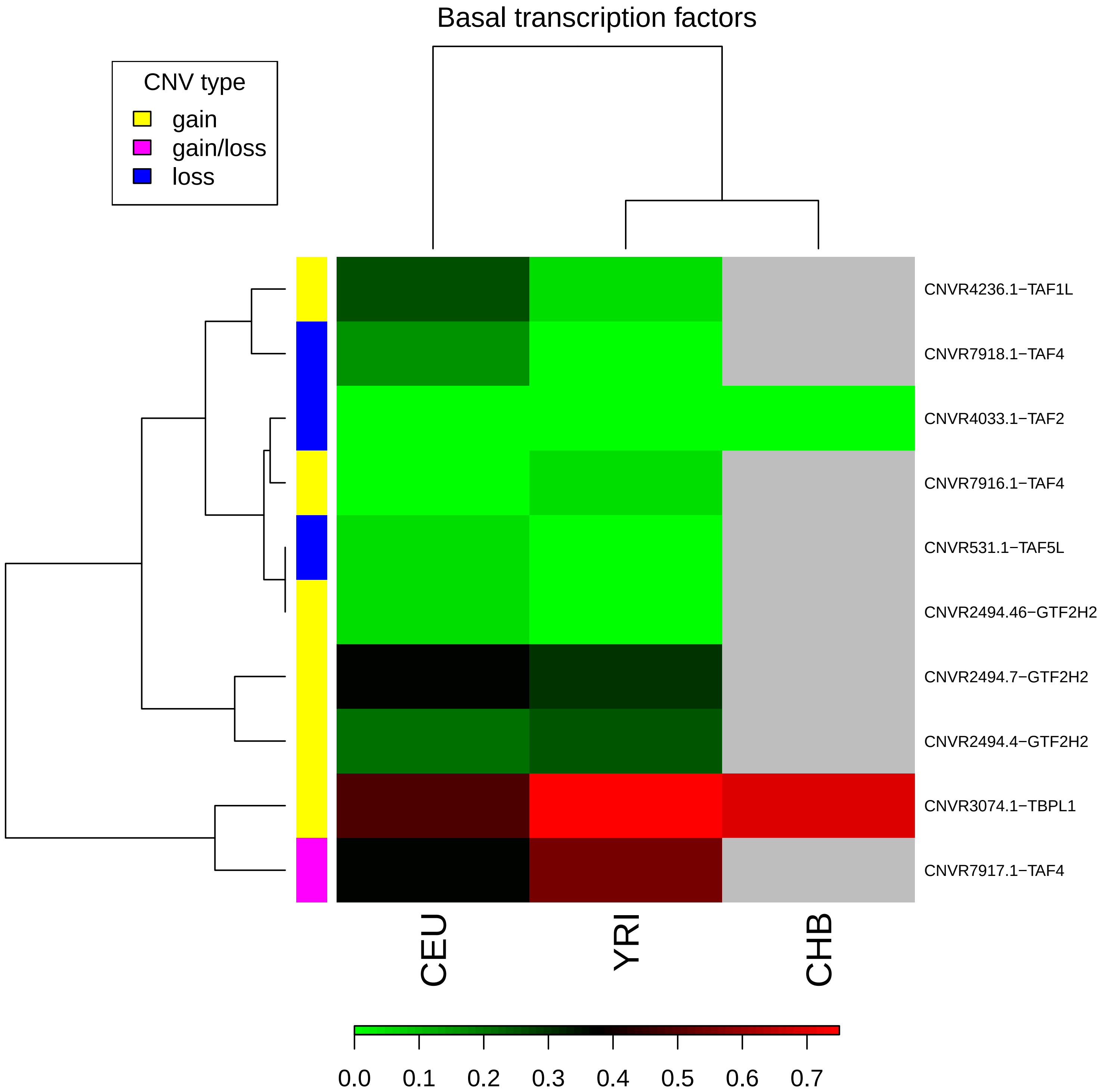

Basal transcription factors
CNV type
gain
gain/loss
loss
CNVR4236.1−TAF1L
CNVR7918.1−TAF4
CNVR4033.1−TAF2
CNVR7916.1−TAF4
CNVR531.1−TAF5L
CNVR2494.46−GTF2H2
CNVR2494.7−GTF2H2
CNVR2494.4−GTF2H2
CNVR3074.1−TBPL1
CNVR7917.1−TAF4
CEU
YRI
CHB
0.0
0.1
0.2
0.3
0.4
0.5
0.6
0.7

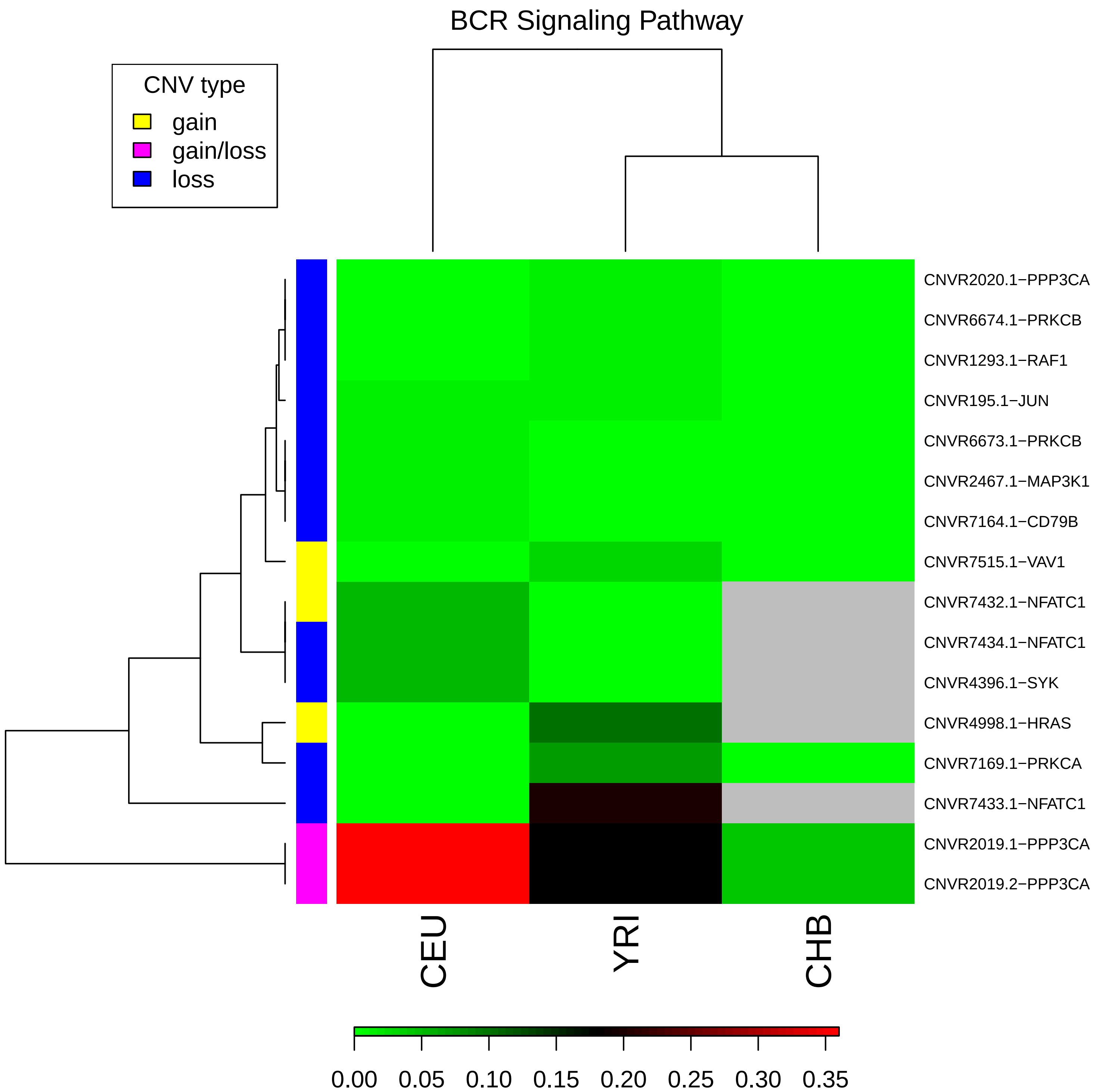

BCR Signaling Pathway
CNV type
gain
gain/loss
loss
CNVR2020.1−PPP3CA
CNVR6674.1−PRKCB
CNVR1293.1−RAF1
CNVR195.1−JUN
CNVR6673.1−PRKCB
CNVR2467.1−MAP3K1
CNVR7164.1−CD79B
CNVR7515.1−VAV1
CNVR7432.1−NFATC1
CNVR7434.1−NFATC1
CNVR4396.1−SYK
CNVR4998.1−HRAS
CNVR7169.1−PRKCA
CNVR7433.1−NFATC1
CNVR2019.1−PPP3CA
CNVR2019.2−PPP3CA
CEU
YRI
CHB
0.00
0.05
0.10
0.15
0.20
0.25
0.30
0.35

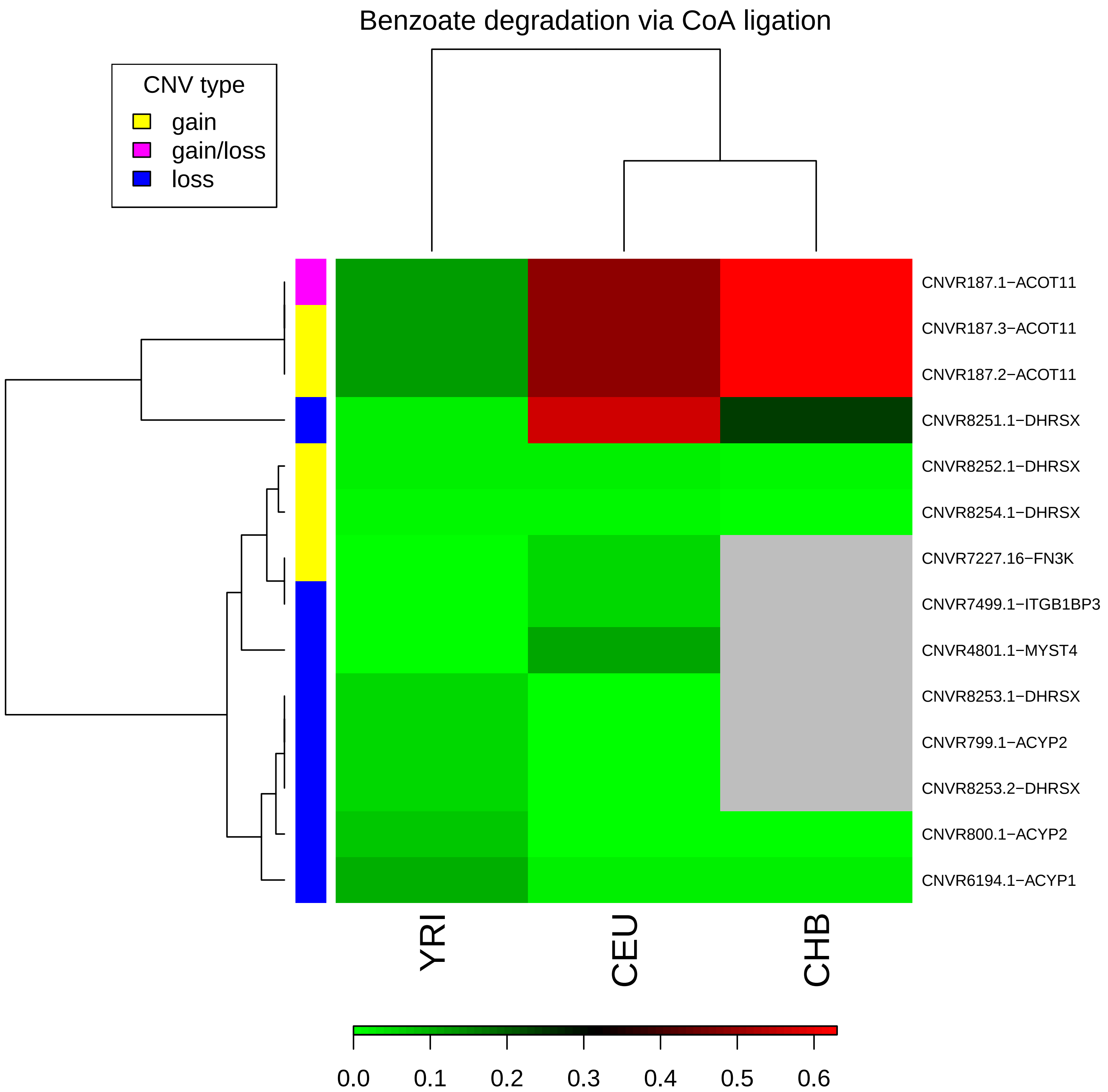

Benzoate degradation via CoA ligation
CNV type
gain
gain/loss
loss
CNVR187.1−ACOT11
CNVR187.3−ACOT11
CNVR187.2−ACOT11
CNVR8251.1−DHRSX
CNVR8252.1−DHRSX
CNVR8254.1−DHRSX
CNVR7227.16−FN3K
CNVR7499.1−ITGB1BP3
CNVR4801.1−MYST4
CNVR8253.1−DHRSX
CNVR799.1−ACYP2
CNVR8253.2−DHRSX
CNVR800.1−ACYP2
CNVR6194.1−ACYP1
YRI
CEU
CHB
0.0
0.1
0.2
0.3
0.4
0.5
0.6

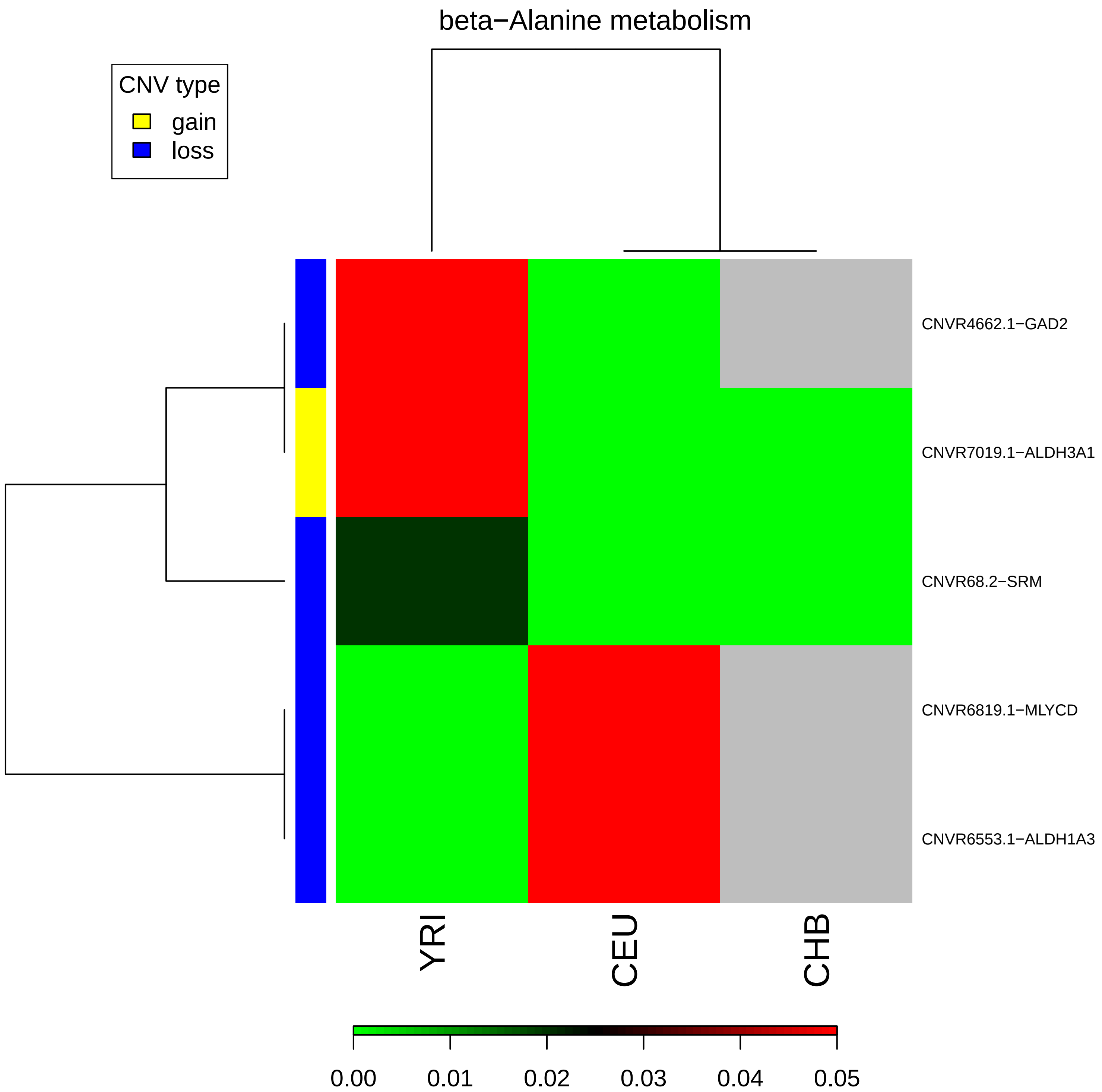
beta−Alanine metabolism
CNV type
gain
loss
CNVR4662.1−GAD2
CNVR7019.1−ALDH3A1
CNVR68.2−SRM
CNVR6819.1−MLYCD
CNVR6553.1−ALDH1A3
YRI
CEU
CHB
0.00
0.01
0.02
0.03
0.04
0.05

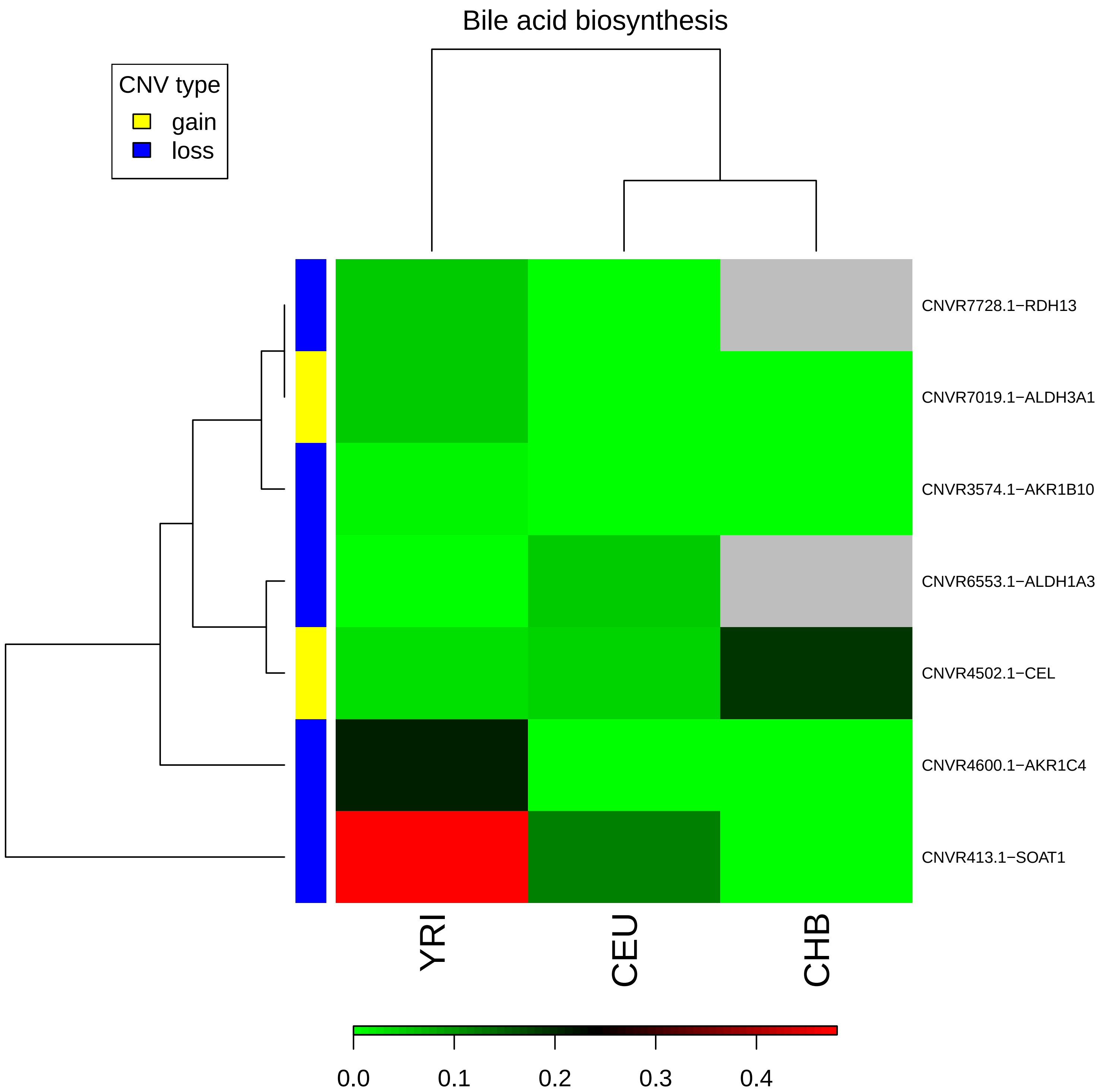

Bile acid biosynthesis
CNV type
gain
loss
CNVR7728.1−RDH13
CNVR7019.1−ALDH3A1
CNVR3574.1−AKR1B10
CNVR6553.1−ALDH1A3
CNVR4502.1−CEL
CNVR4600.1−AKR1C4
CNVR413.1−SOAT1
YRI
CEU
CHB
0.0
0.1
0.2
0.3
0.4

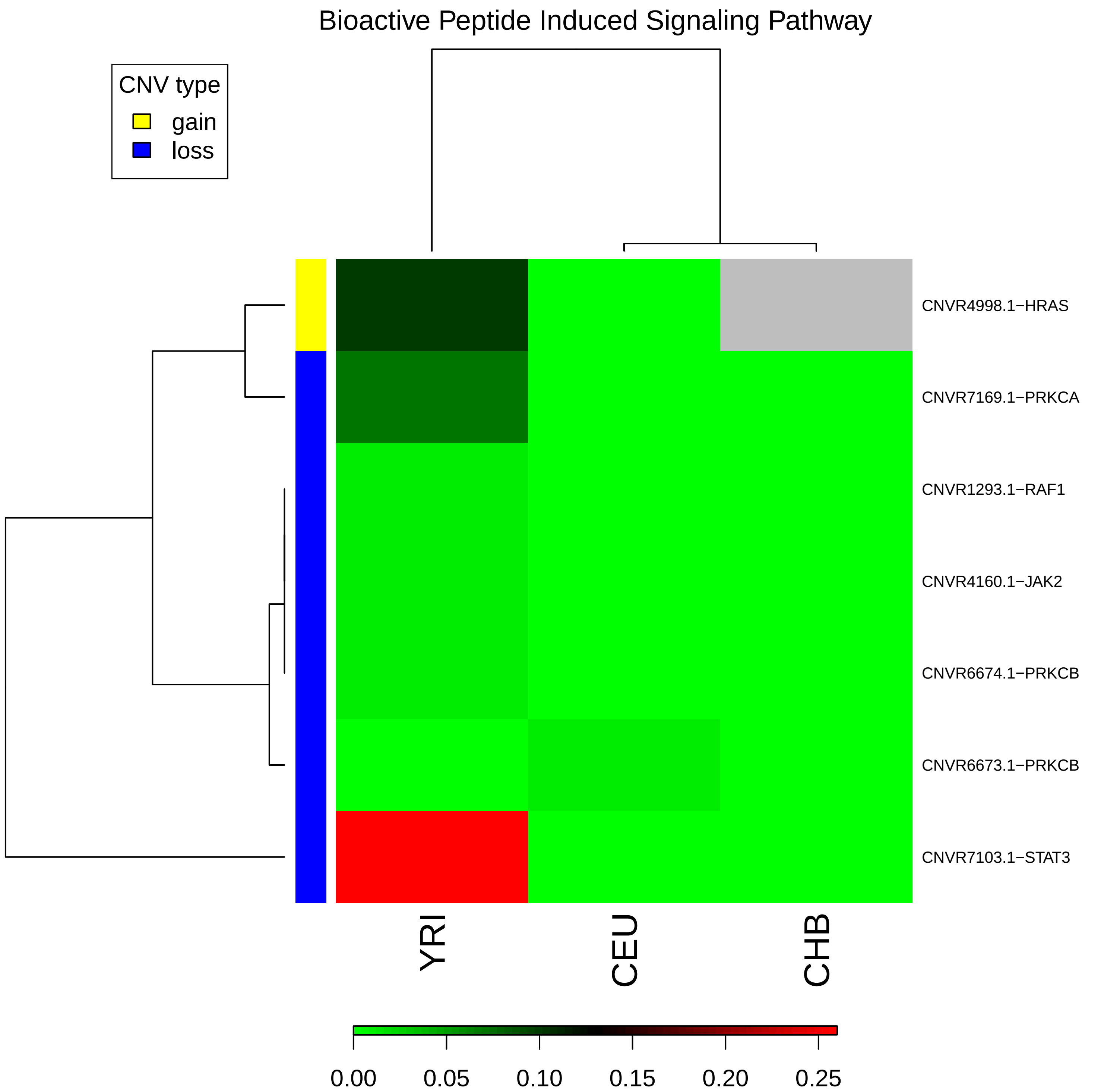

Bioactive Peptide Induced Signaling Pathway
CNV type
gain
loss
CNVR4998.1−HRAS
CNVR7169.1−PRKCA
CNVR1293.1−RAF1
CNVR4160.1−JAK2
CNVR6674.1−PRKCB
CNVR6673.1−PRKCB
CNVR7103.1−STAT3
YRI
CEU
CHB
0.00
0.05
0.10
0.15
0.20
0.25

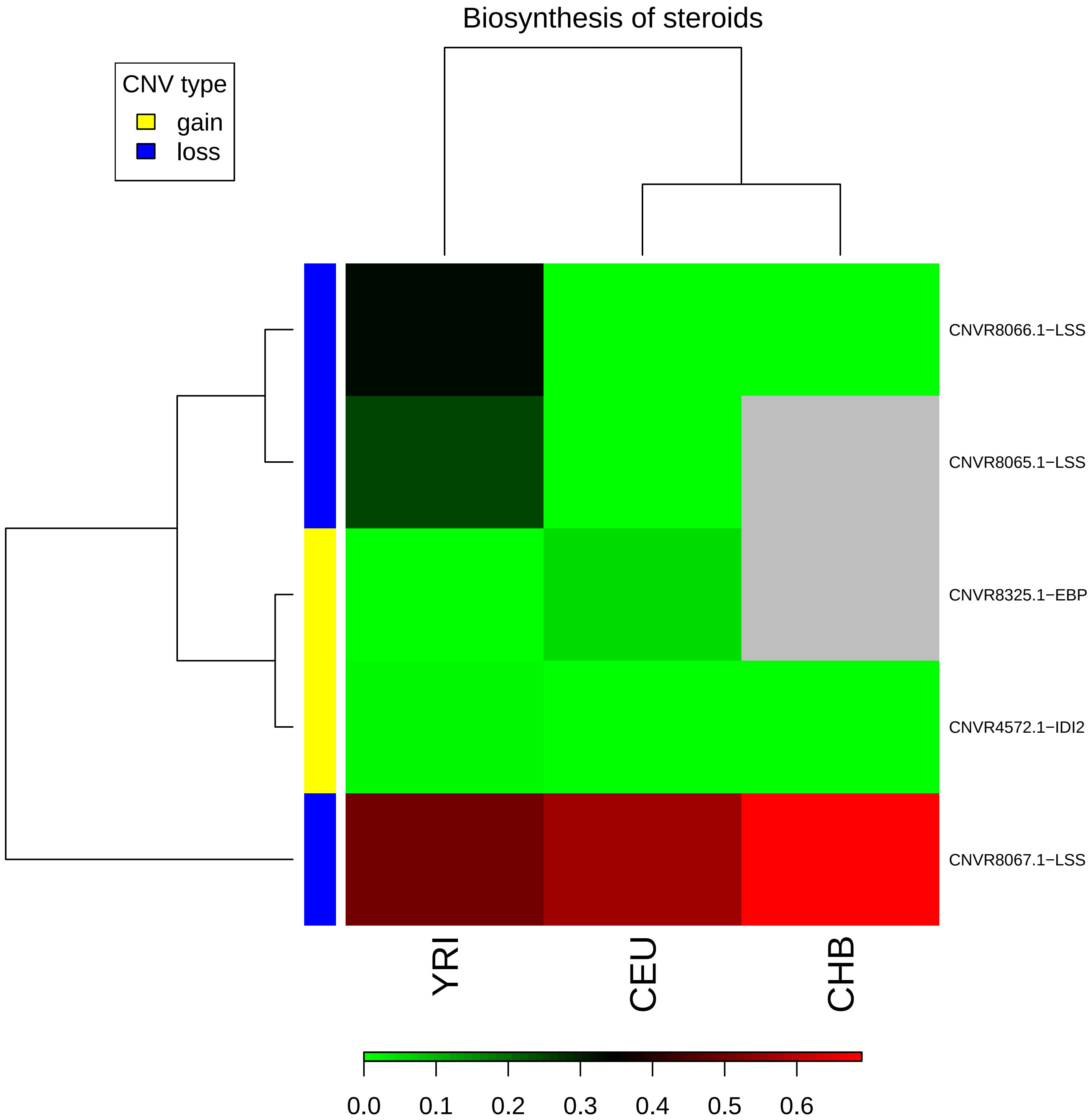

Biosynthesis of steroids
CNV type
gain
loss
CNVR8066.1−LSS
CNVR8065.1−LSS
CNVR8325.1−EBP
CNVR4572.1−IDI2
CNVR8067.1−LSS
YRI
CEU
CHB
0.0
0.1
0.2
0.3
0.4
0.5
0.6

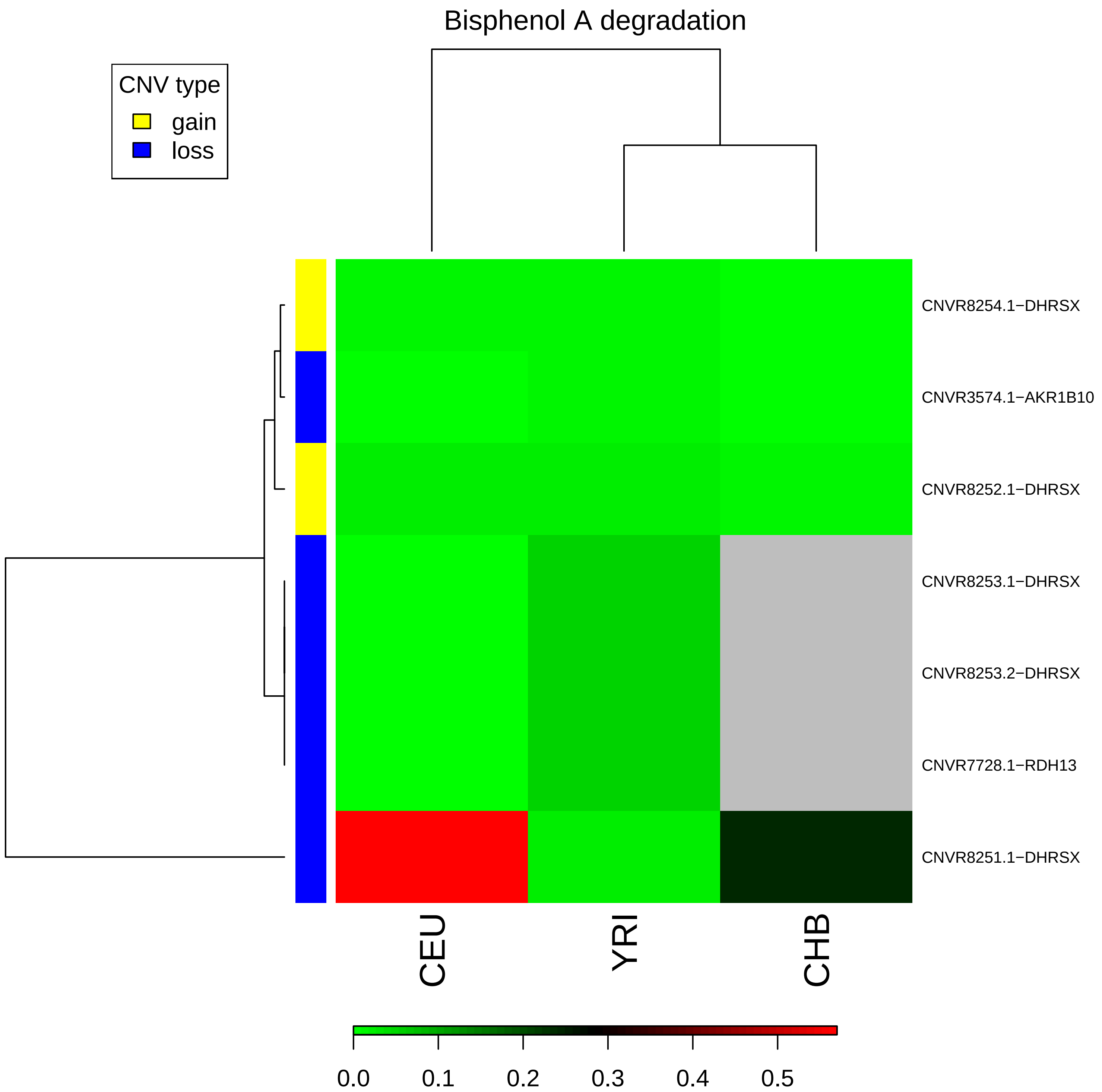

Bisphenol A degradation
CNV type
gain
loss
CNVR8254.1−DHRSX
CNVR3574.1−AKR1B10
CNVR8252.1−DHRSX
CNVR8253.1−DHRSX
CNVR8253.2−DHRSX
CNVR7728.1−RDH13
CNVR8251.1−DHRSX
CEU
YRI
CHB
0.0
0.1
0.2
0.3
0.4
0.5

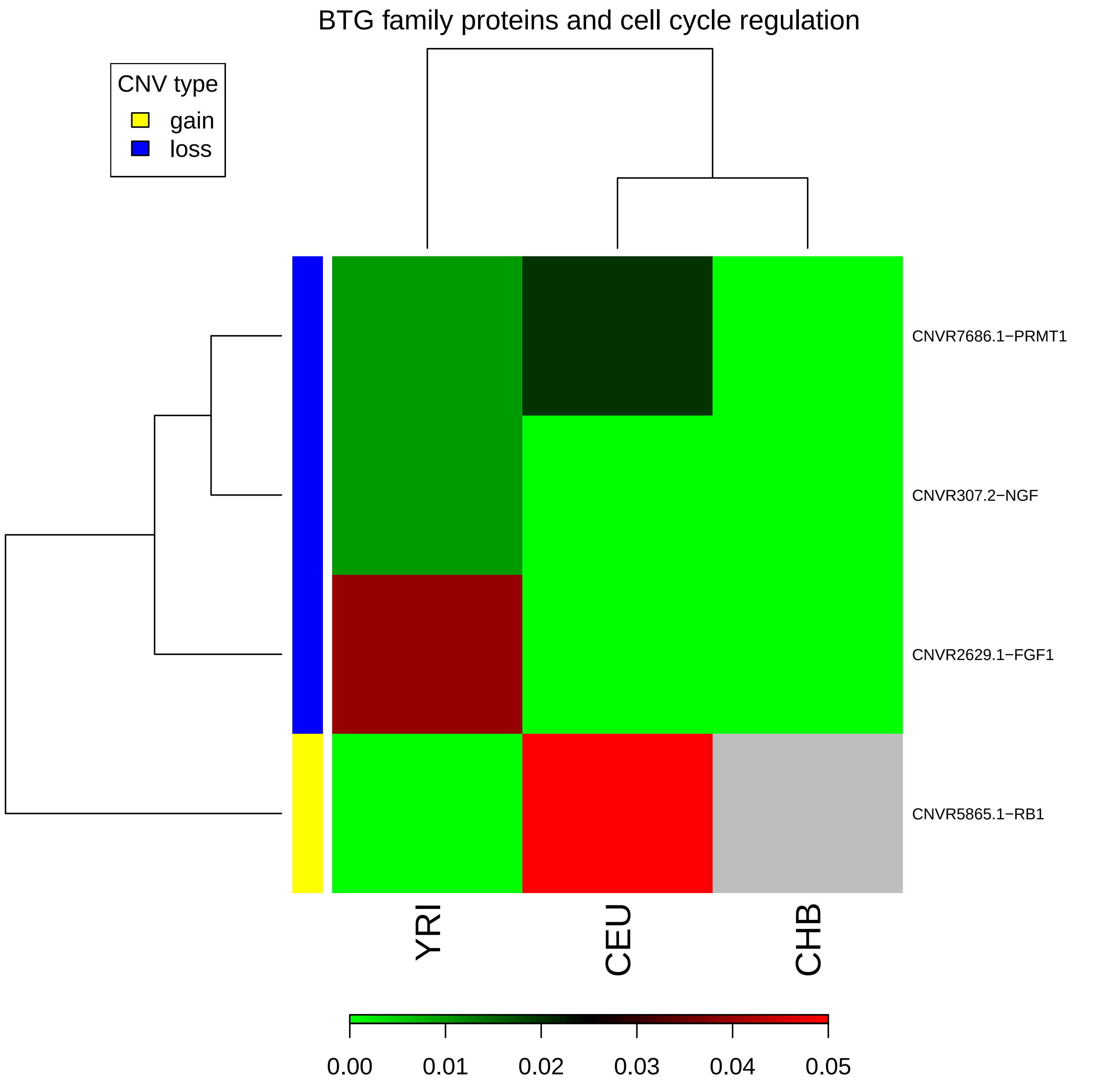
BTG family proteins and cell cycle regulation
CNV type
gain
loss
CNVR7686.1−PRMT1
CNVR307.2−NGF
CNVR2629.1−FGF1
CNVR5865.1−RB1
YRI
CEU
CHB
0.00
0.01
0.02
0.03
0.04
0.05

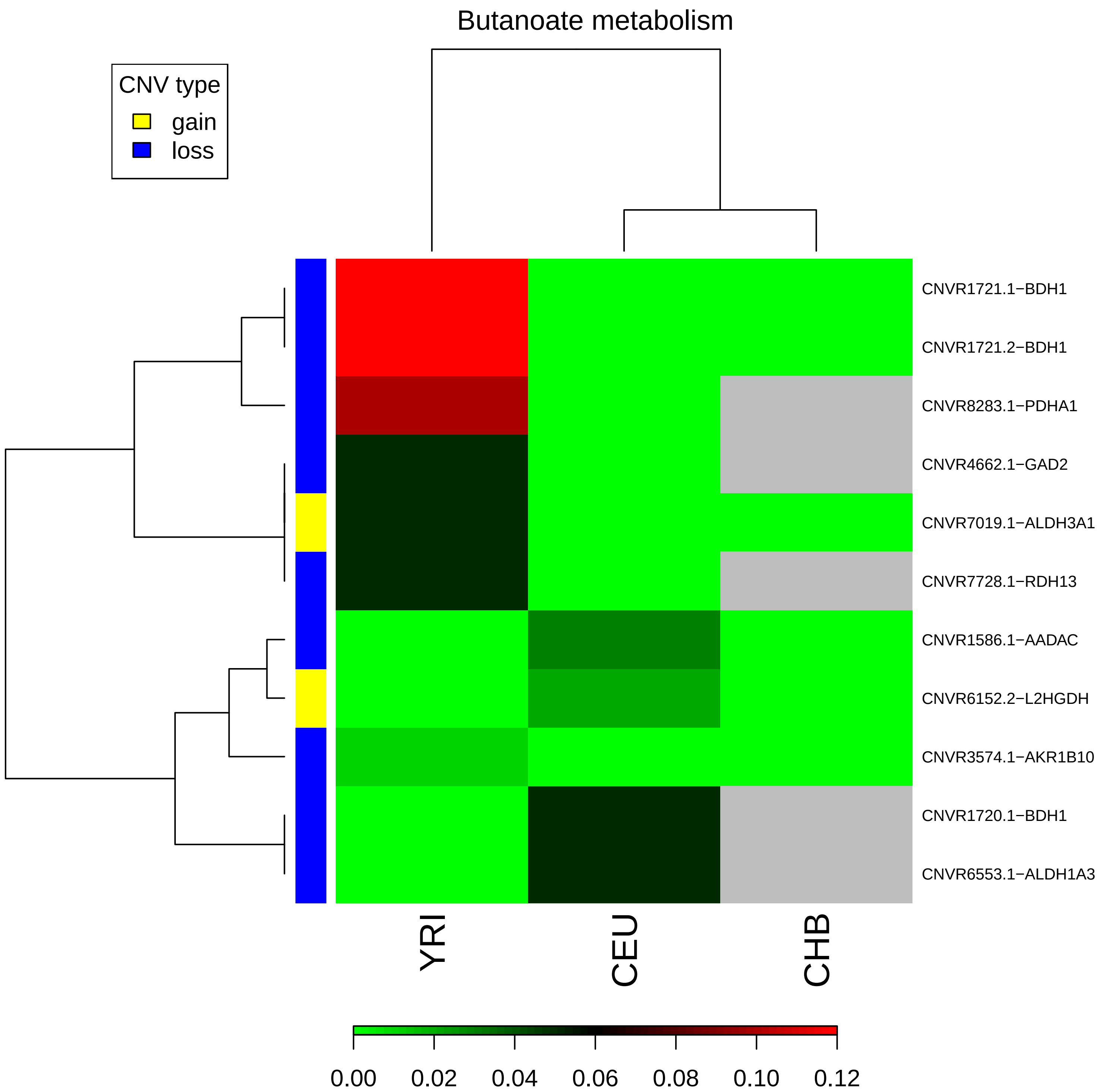

Butanoate metabolism
CNV type
gain
loss
CNVR1721.1−BDH1
CNVR1721.2−BDH1
CNVR8283.1−PDHA1
CNVR4662.1−GAD2
CNVR7019.1−ALDH3A1
CNVR7728.1−RDH13
CNVR1586.1−AADAC
CNVR6152.2−L2HGDH
CNVR3574.1−AKR1B10
CNVR1720.1−BDH1
CNVR6553.1−ALDH1A3
YRI
CEU
CHB
0.00
0.02
0.04
0.06
0.08
0.10
0.12

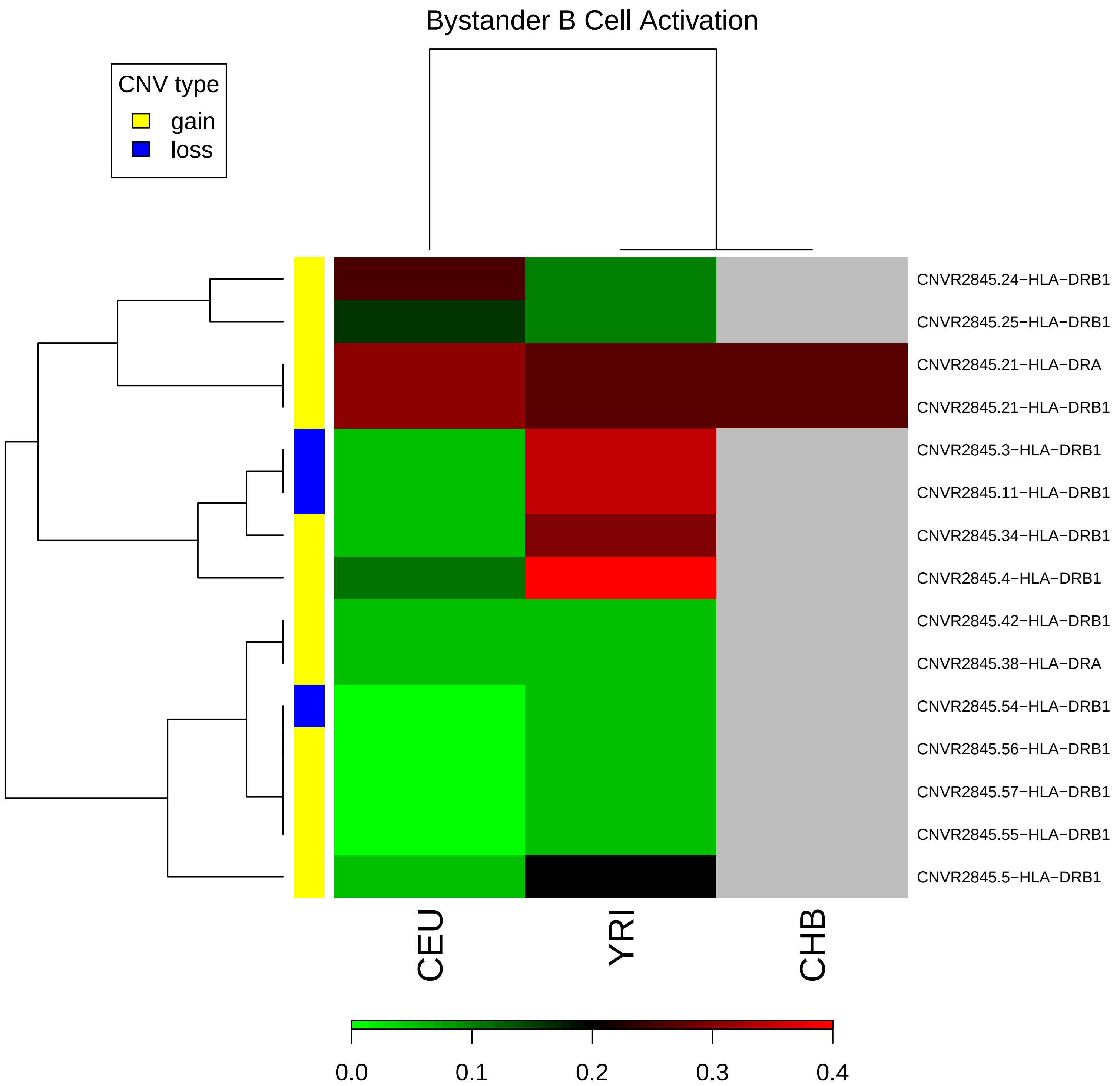
Bystander B Cell Activation
CNV type
gain
loss
CNVR2845.24−HLA−DRB1
CNVR2845.25−HLA−DRB1
CNVR2845.21−HLA−DRA
CNVR2845.21−HLA−DRB1
CNVR2845.3−HLA−DRB1
CNVR2845.11−HLA−DRB1
CNVR2845.34−HLA−DRB1
CNVR2845.4−HLA−DRB1
CNVR2845.42−HLA−DRB1
CNVR2845.38−HLA−DRA
CNVR2845.54−HLA−DRB1
CNVR2845.56−HLA−DRB1
CNVR2845.57−HLA−DRB1
CNVR2845.55−HLA−DRB1
CNVR2845.5−HLA−DRB1
CEU
YRI
CHB
0.0
0.1
0.2
0.3
0.4

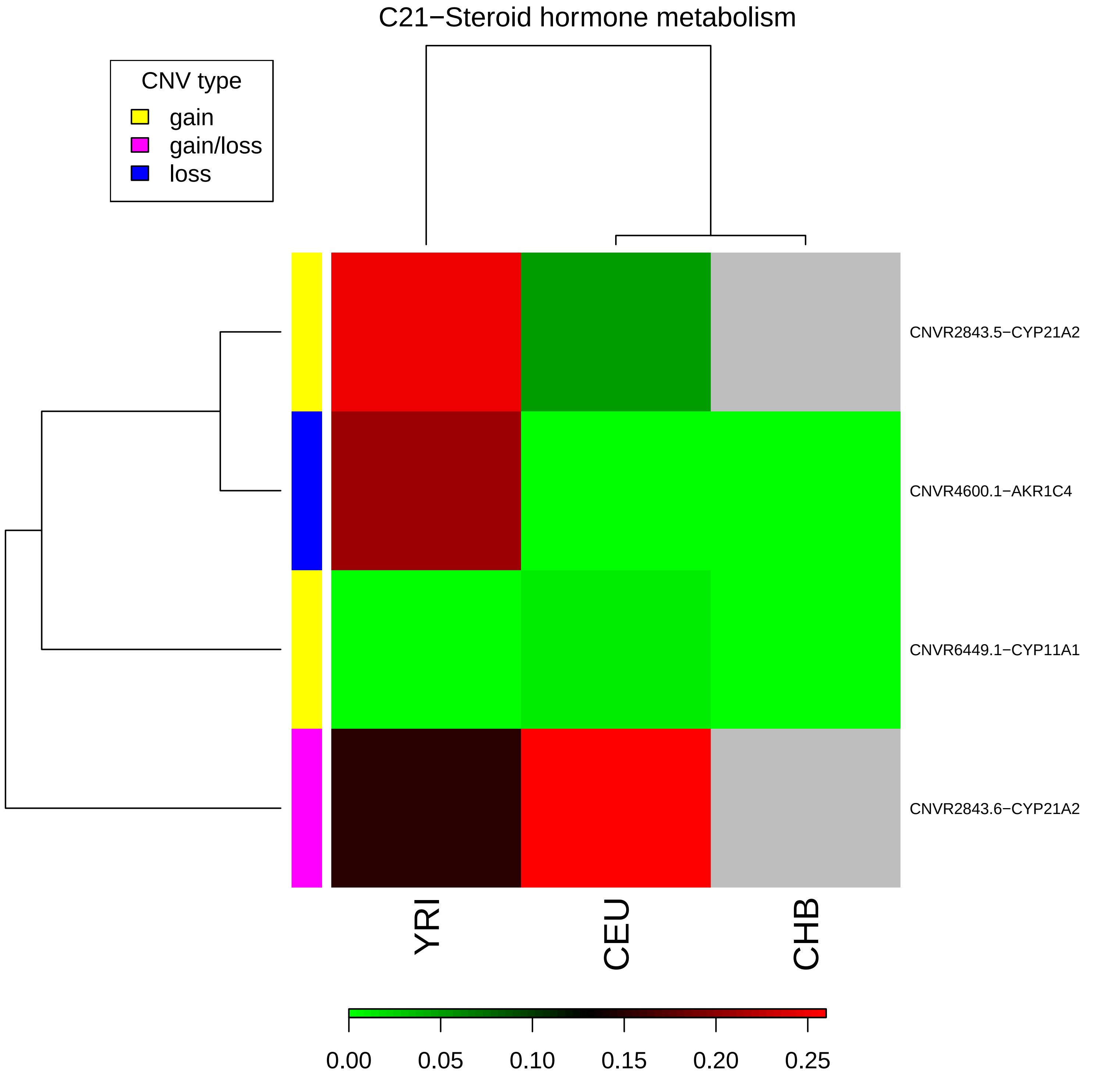

C21−Steroid hormone metabolism
CNV type
gain
gain/loss
loss
CNVR2843.5−CYP21A2
CNVR4600.1−AKR1C4
CNVR6449.1−CYP11A1
CNVR2843.6−CYP21A2
YRI
CEU
CHB
0.00
0.05
0.10
0.15
0.20
0.25

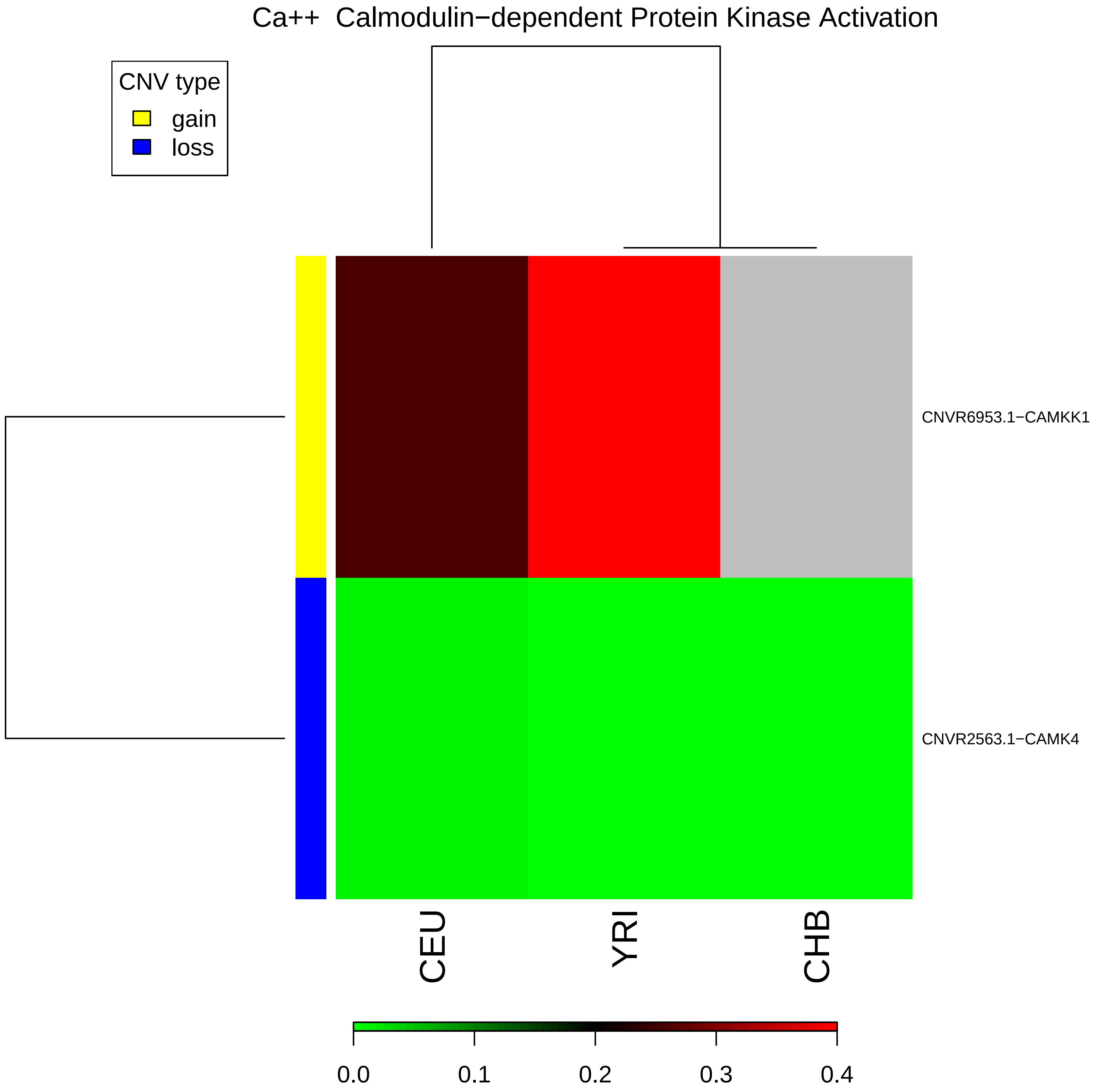
Ca++ Calmodulin−dependent Protein Kinase Activation
CNV type
gain
loss
CNVR6953.1−CAMKK1
CNVR2563.1−CAMK4
CEU
YRI
CHB
0.0
0.1
0.2
0.3
0.4

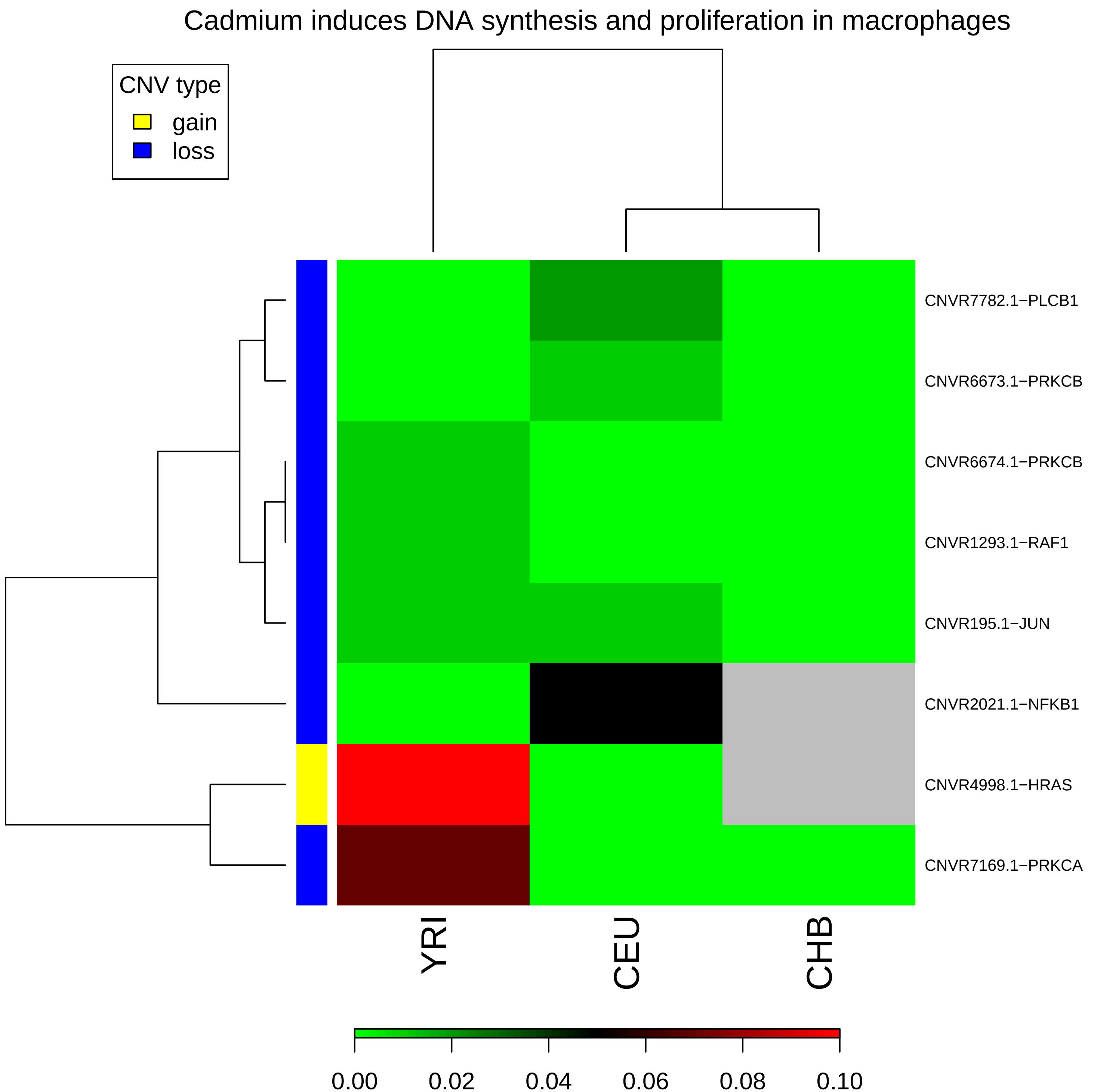

Cadmium induces DNA synthesis and proliferation in macrophages
CNV type
gain
loss
CNVR7782.1−PLCB1
CNVR6673.1−PRKCB
CNVR6674.1−PRKCB
CNVR1293.1−RAF1
CNVR195.1−JUN
CNVR2021.1−NFKB1
CNVR4998.1−HRAS
CNVR7169.1−PRKCA
YRI
CEU
CHB
0.00
0.02
0.04
0.06
0.08
0.10

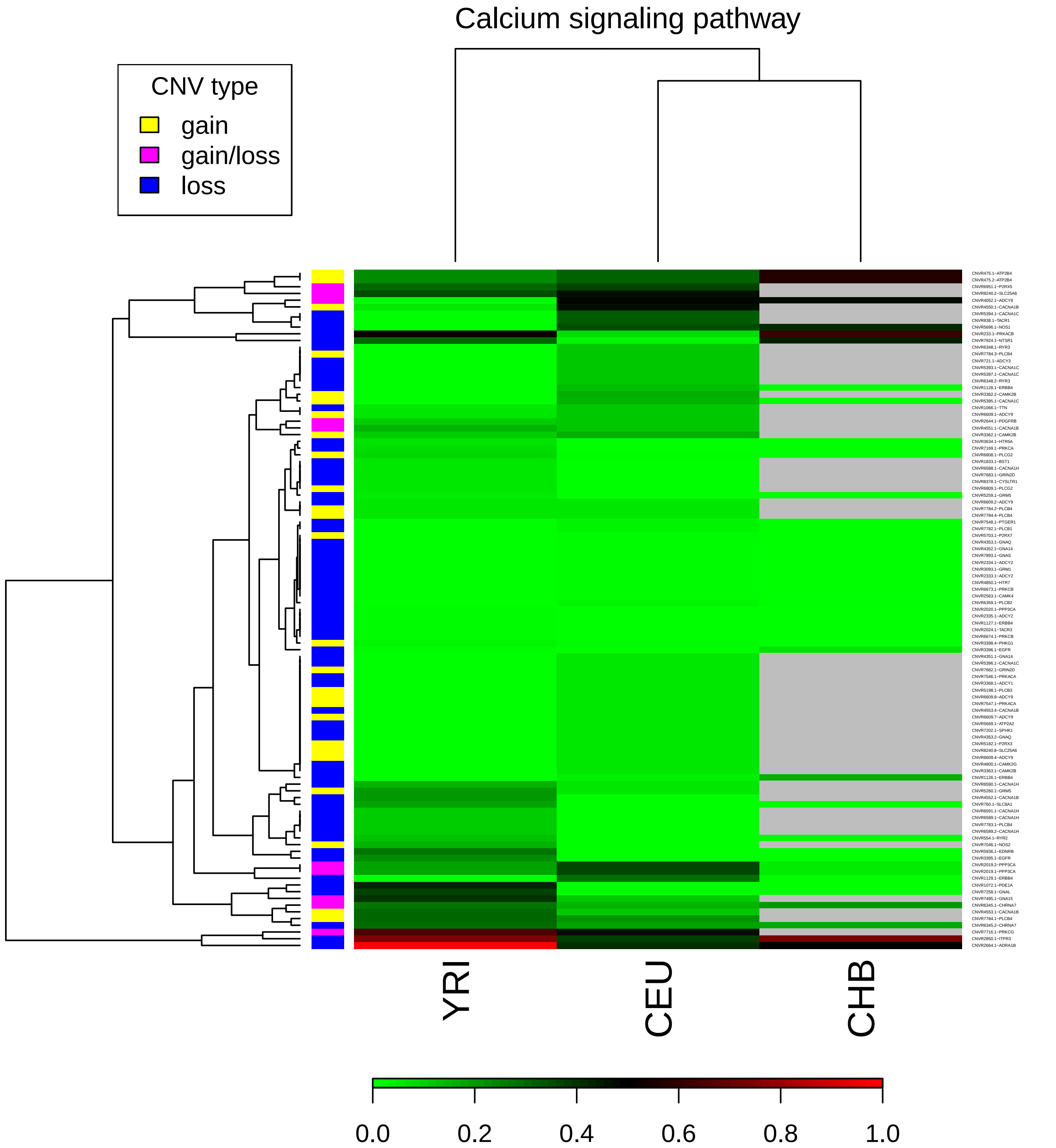

Calcium signaling pathway
CNV type
gain
gain/loss
loss
CNVR475.1−ATP2B4
CNVR475.2−ATP2B4
CNVR6951.1−P2RX5
CNVR8240.2−SLC25A6
CNVR4052.1−ADCY8
CNVR4550.1−CACNA1B
CNVR5394.1−CACNA1C
CNVR838.1−TACR1
CNVR5696.1−NOS1
CNVR233.1−PRKACB
CNVR7924.1−NTSR1
CNVR6348.1−RYR3
CNVR7784.3−PLCB4
CNVR721.1−ADCY3
CNVR5393.1−CACNA1C
CNVR5397.1−CACNA1C
CNVR6348.2−RYR3
CNVR1128.1−ERBB4
CNVR3362.2−CAMK2B
CNVR5395.1−CACNA1C
CNVR1066.1−TTN
CNVR6609.1−ADCY9
CNVR2644.1−PDGFRB
CNVR4551.1−CACNA1B
CNVR3362.1−CAMK2B
CNVR3634.1−HTR5A
CNVR7169.1−PRKCA
CNVR6808.1−PLCG2
CNVR1833.1−BST1
CNVR6588.1−CACNA1H
CNVR7683.1−GRIN2D
CNVR8378.1−CYSLTR1
CNVR6809.1−PLCG2
CNVR5259.1−GRM5
CNVR6609.2−ADCY9
CNVR7784.2−PLCB4
CNVR7784.4−PLCB4
CNVR7548.1−PTGER1
CNVR7782.1−PLCB1
CNVR5703.1−P2RX7
CNVR4353.1−GNAQ
CNVR4352.1−GNA14
CNVR7893.1−GNAS
CNVR2334.1−ADCY2
CNVR3093.1−GRM1
CNVR2333.1−ADCY2
CNVR4850.1−HTR7
CNVR6673.1−PRKCB
CNVR2563.1−CAMK4
CNVR6359.1−PLCB2
CNVR2020.1−PPP3CA
CNVR2335.1−ADCY2
CNVR1127.1−ERBB4
CNVR2024.1−TACR3
CNVR6674.1−PRKCB
CNVR3398.4−PHKG1
CNVR3396.1−EGFR
CNVR4351.1−GNA14
CNVR5396.1−CACNA1C
CNVR7682.1−GRIN2D
CNVR7546.1−PRKACA
CNVR3368.1−ADCY1
CNVR5198.1−PLCB3
CNVR6609.8−ADCY9
CNVR7547.1−PRKACA
CNVR4553.4−CACNA1B
CNVR6609.7−ADCY9
CNVR5669.1−ATP2A2
CNVR7202.1−SPHK1
CNVR4353.2−GNAQ
CNVR5182.1−P2RX3
CNVR8240.8−SLC25A6
CNVR6609.4−ADCY9
CNVR4800.1−CAMK2G
CNVR3363.1−CAMK2B
CNVR1126.1−ERBB4
CNVR6590.1−CACNA1H
CNVR5260.1−GRM5
CNVR4552.1−CACNA1B
CNVR760.1−SLC8A1
CNVR6591.1−CACNA1H
CNVR6589.1−CACNA1H
CNVR7783.1−PLCB4
CNVR6589.2−CACNA1H
CNVR554.1−RYR2
CNVR7046.1−NOS2
CNVR5936.1−EDNRB
CNVR3395.1−EGFR
CNVR2019.2−PPP3CA
CNVR2019.1−PPP3CA
CNVR1129.1−ERBB4
CNVR1072.1−PDE1A
CNVR7258.1−GNAL
CNVR7495.1−GNA15
CNVR6345.1−CHRNA7
CNVR4553.1−CACNA1B
CNVR7784.1−PLCB4
CNVR6345.2−CHRNA7
CNVR7716.1−PRKCG
CNVR2850.1−ITPR3
CNVR2664.1−ADRA1B
YRI
CEU
CHB
0.0
0.2
0.4
0.6
0.8
1.0

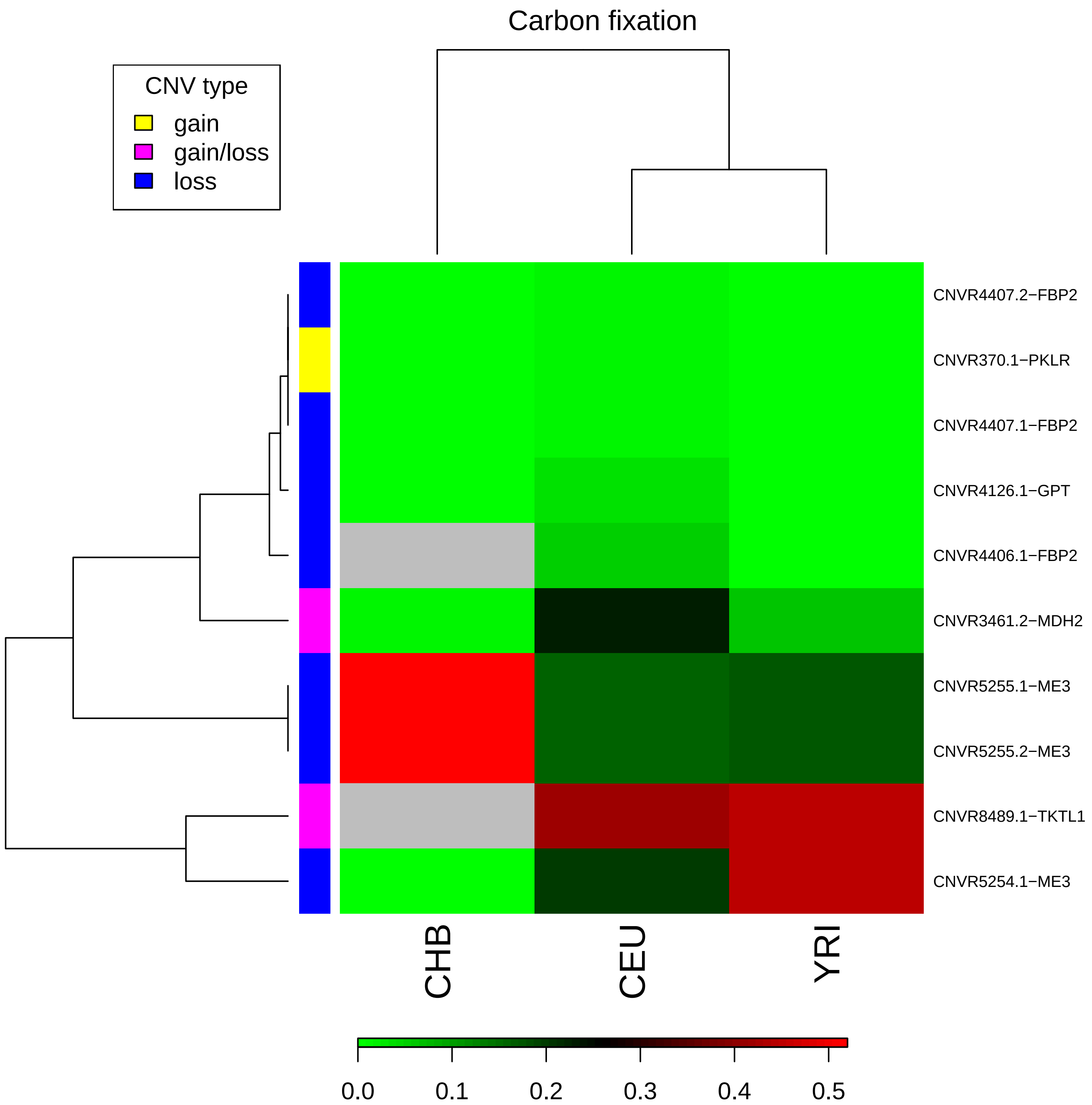

Carbon fixation
CNV type
gain
gain/loss
loss
CNVR4407.2−FBP2
CNVR370.1−PKLR
CNVR4407.1−FBP2
CNVR4126.1−GPT
CNVR4406.1−FBP2
CNVR3461.2−MDH2
CNVR5255.1−ME3
CNVR5255.2−ME3
CNVR8489.1−TKTL1
CNVR5254.1−ME3
CHB
CEU
YRI
0.0
0.1
0.2
0.3
0.4
0.5

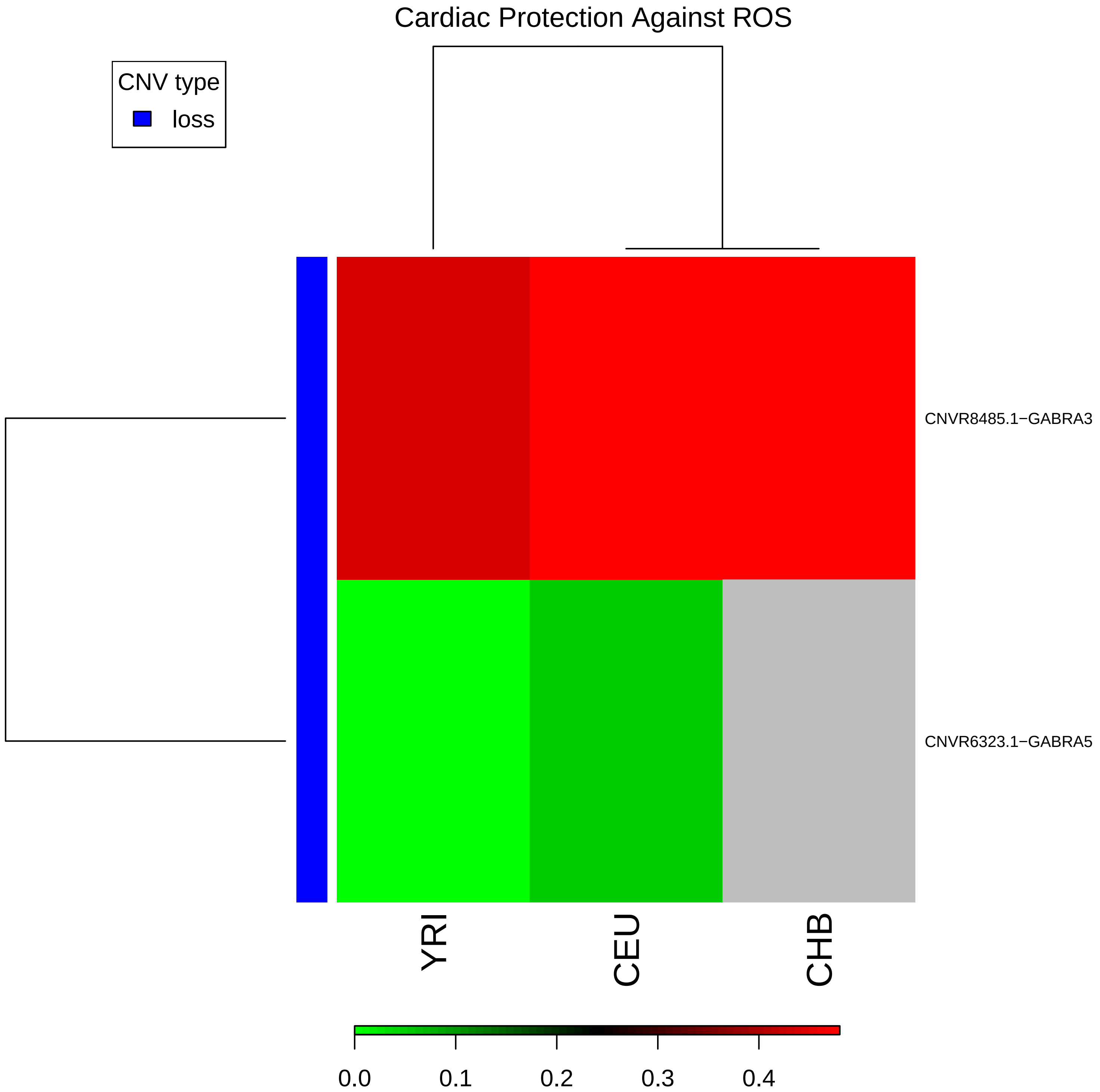
Cardiac Protection Against ROS
CNV type
loss
CNVR8485.1−GABRA3
CNVR6323.1−GABRA5
YRI
CEU
CHB
0.0
0.1
0.2
0.3
0.4

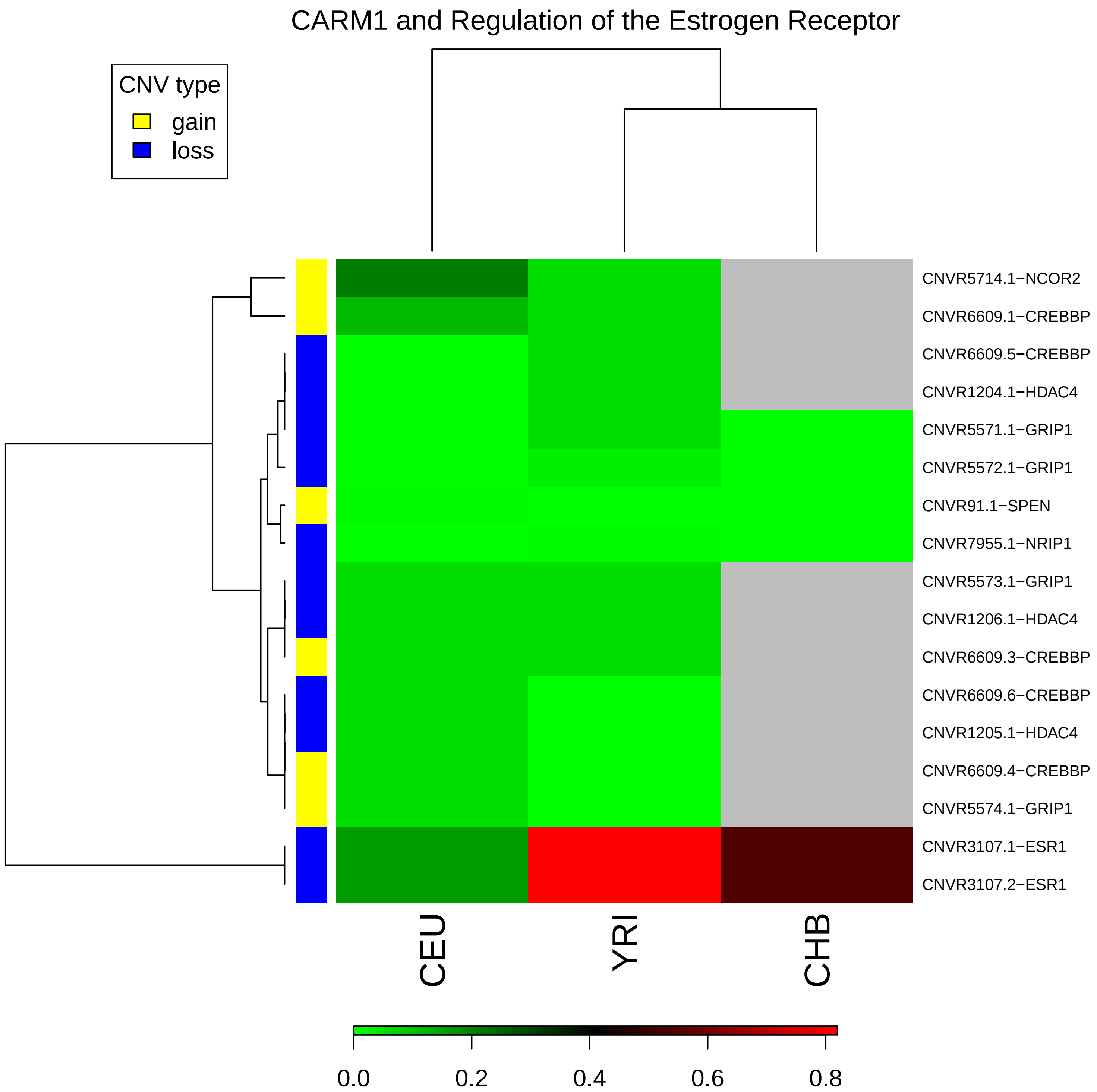

CARM1 and Regulation of the Estrogen Receptor
CNV type
gain
loss
CNVR5714.1−NCOR2
CNVR6609.1−CREBBP
CNVR6609.5−CREBBP
CNVR1204.1−HDAC4
CNVR5571.1−GRIP1
CNVR5572.1−GRIP1
CNVR91.1−SPEN
CNVR7955.1−NRIP1
CNVR5573.1−GRIP1
CNVR1206.1−HDAC4
CNVR6609.3−CREBBP
CNVR6609.6−CREBBP
CNVR1205.1−HDAC4
CNVR6609.4−CREBBP
CNVR5574.1−GRIP1
CNVR3107.1−ESR1
CNVR3107.2−ESR1
CEU
YRI
CHB
0.0
0.2
0.4
0.6
0.8

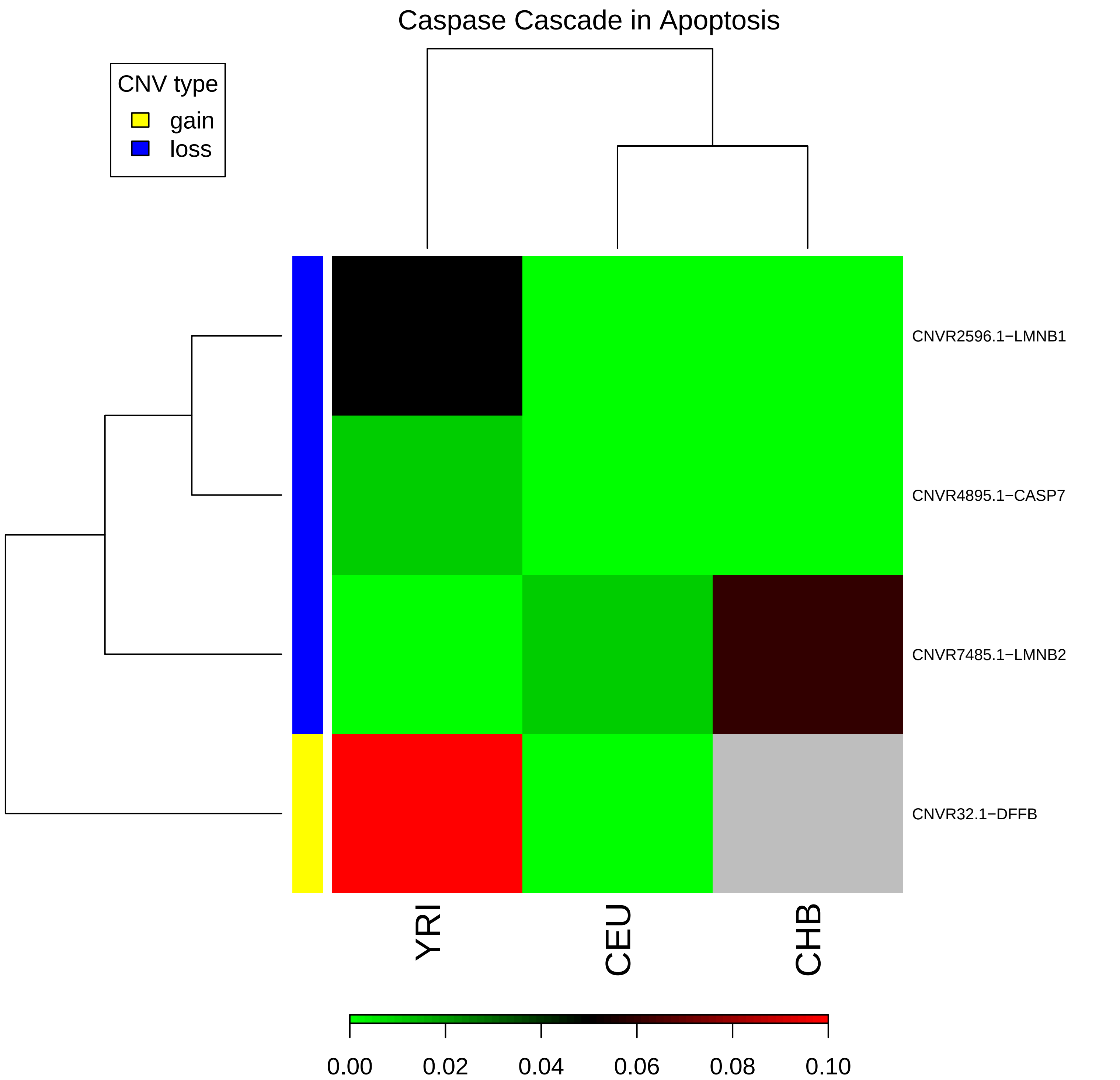
Caspase Cascade in Apoptosis
CNV type
gain
loss
CNVR2596.1−LMNB1
CNVR4895.1−CASP7
CNVR7485.1−LMNB2
CNVR32.1−DFFB
YRI
CEU
CHB
0.00
0.02
0.04
0.06
0.08
0.10

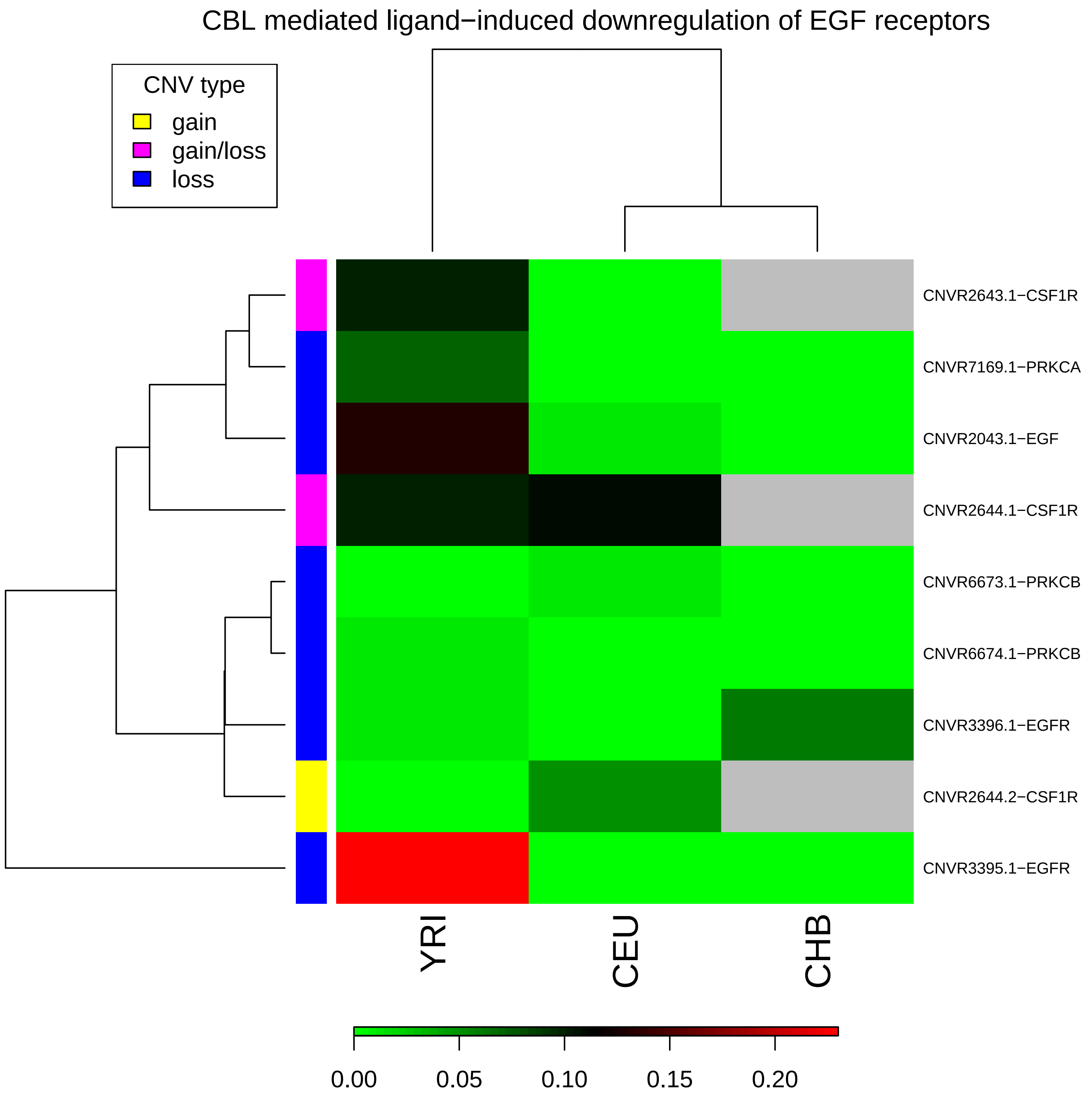
CBL mediated ligand−induced downregulation of EGF receptors
CNV type
gain
gain/loss
loss
CNVR2643.1−CSF1R
CNVR7169.1−PRKCA
CNVR2043.1−EGF
CNVR2644.1−CSF1R
CNVR6673.1−PRKCB
CNVR6674.1−PRKCB
CNVR3396.1−EGFR
CNVR2644.2−CSF1R
CNVR3395.1−EGFR
YRI
CEU
CHB
0.00
0.05
0.10
0.15
0.20

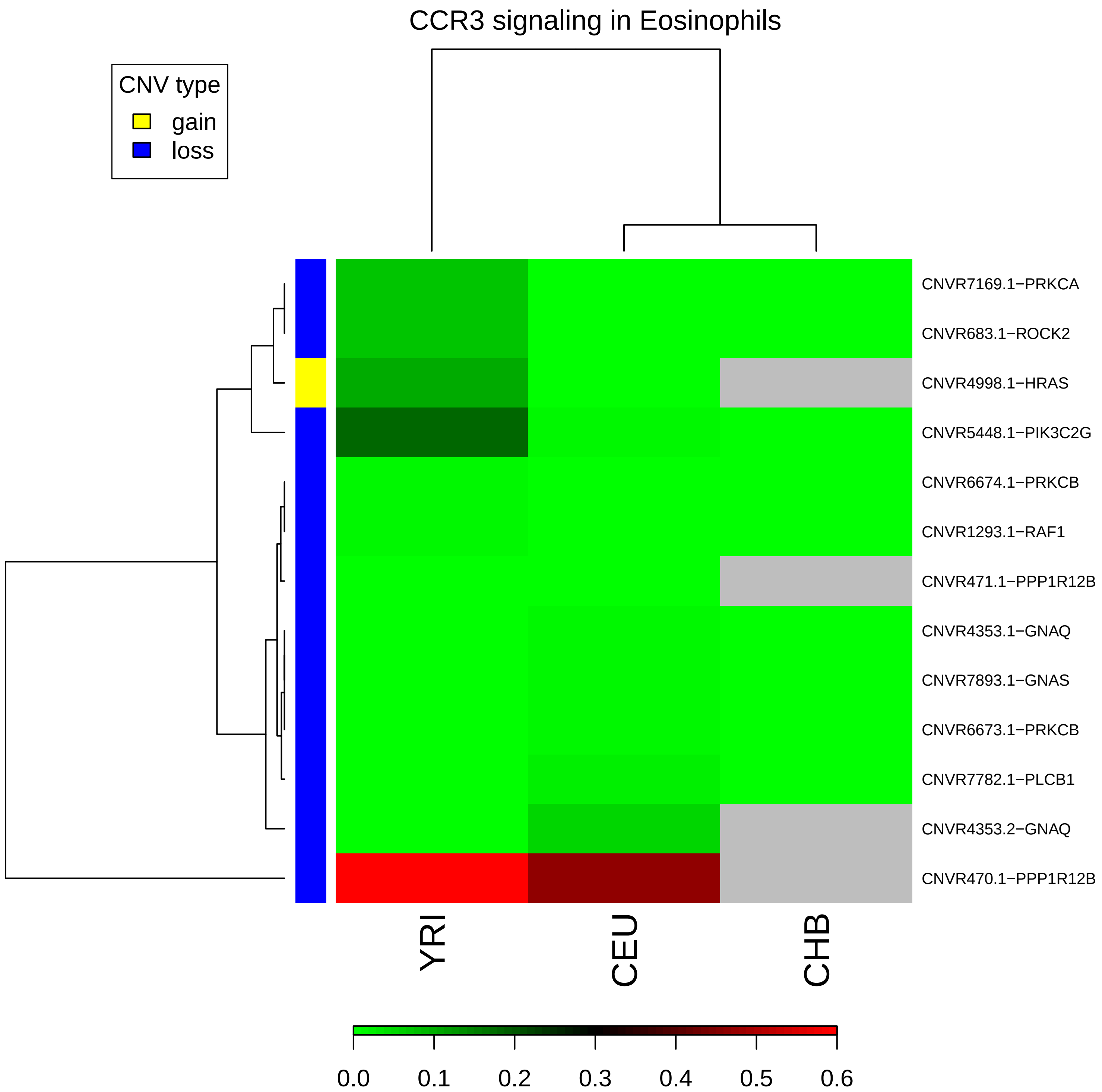
CCR3 signaling in Eosinophils
CNV type
gain
loss
CNVR7169.1−PRKCA
CNVR683.1−ROCK2
CNVR4998.1−HRAS
CNVR5448.1−PIK3C2G
CNVR6674.1−PRKCB
CNVR1293.1−RAF1
CNVR471.1−PPP1R12B
CNVR4353.1−GNAQ
CNVR7893.1−GNAS
CNVR6673.1−PRKCB
CNVR7782.1−PLCB1
CNVR4353.2−GNAQ
CNVR470.1−PPP1R12B
YRI
CEU
CHB
0.0
0.1
0.2
0.3
0.4
0.5
0.6

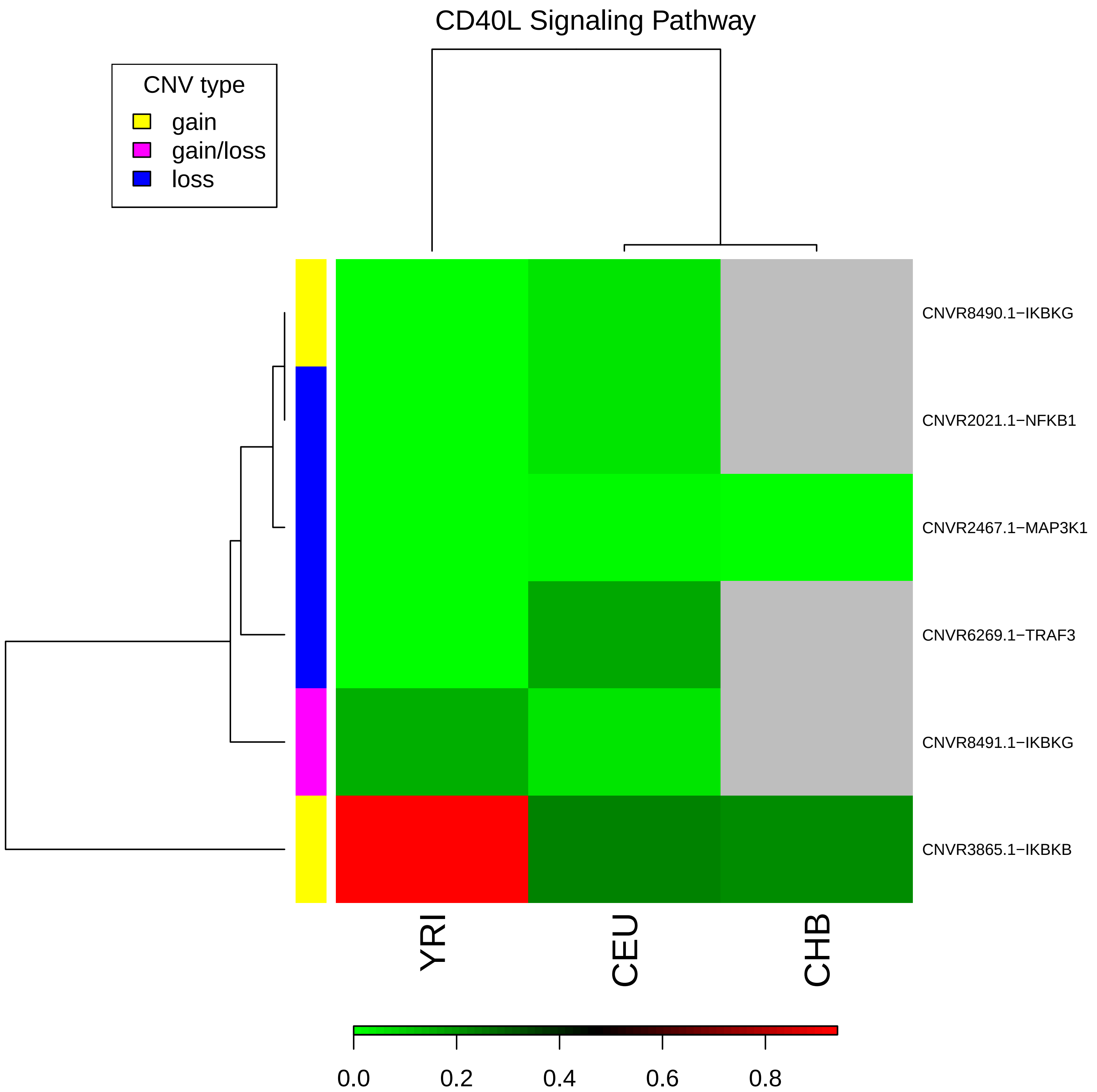
CD40L Signaling Pathway
CNV type
gain
gain/loss
loss
CNVR8490.1−IKBKG
CNVR2021.1−NFKB1
CNVR2467.1−MAP3K1
CNVR6269.1−TRAF3
CNVR8491.1−IKBKG
CNVR3865.1−IKBKB
YRI
CEU
CHB
0.0
0.2
0.4
0.6
0.8

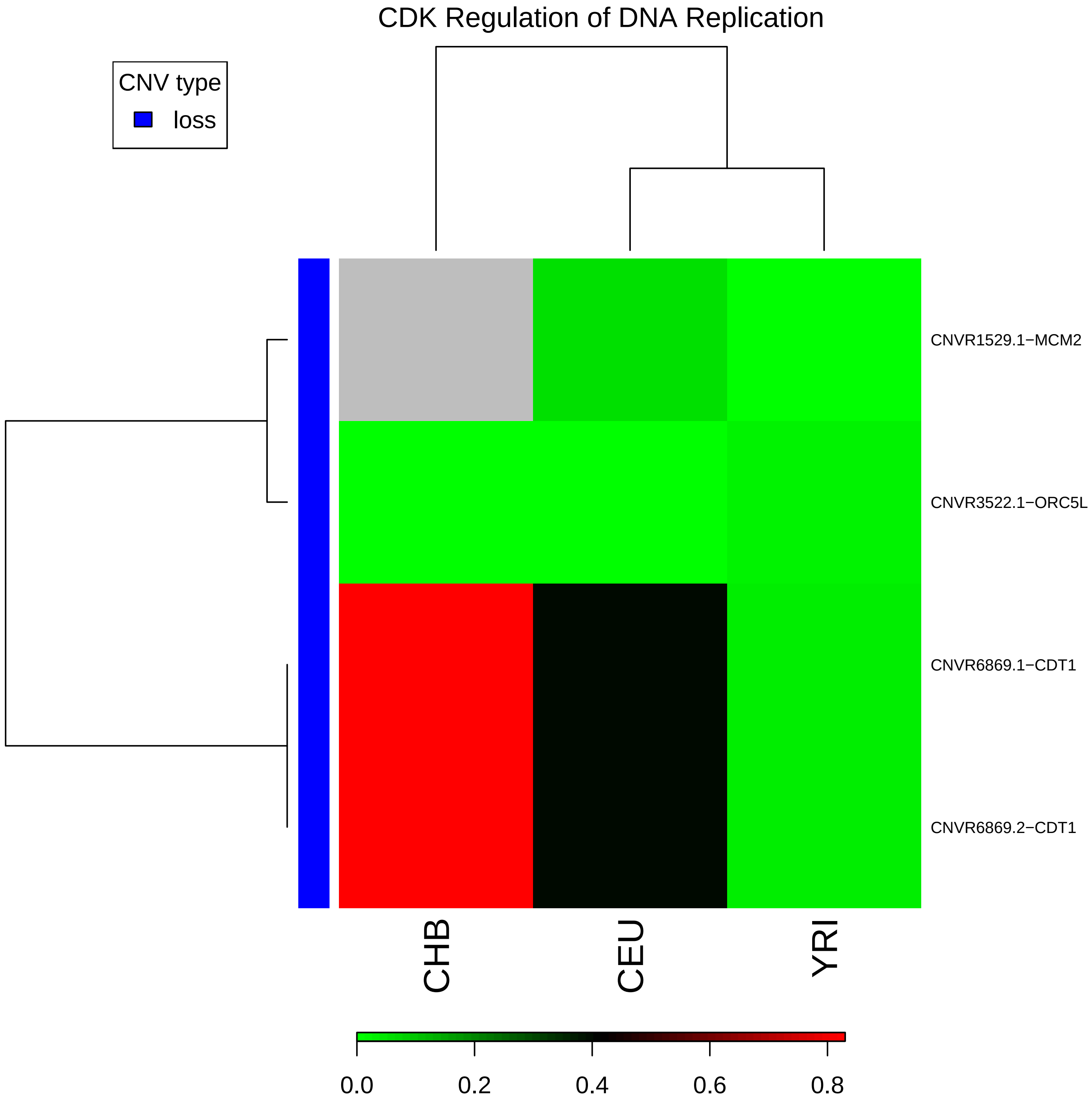

CDK Regulation of DNA Replication
CNV type
loss
CNVR1529.1−MCM2
CNVR3522.1−ORC5L
CNVR6869.1−CDT1
CNVR6869.2−CDT1
CHB
CEU
YRI
0.0
0.2
0.4
0.6
0.8

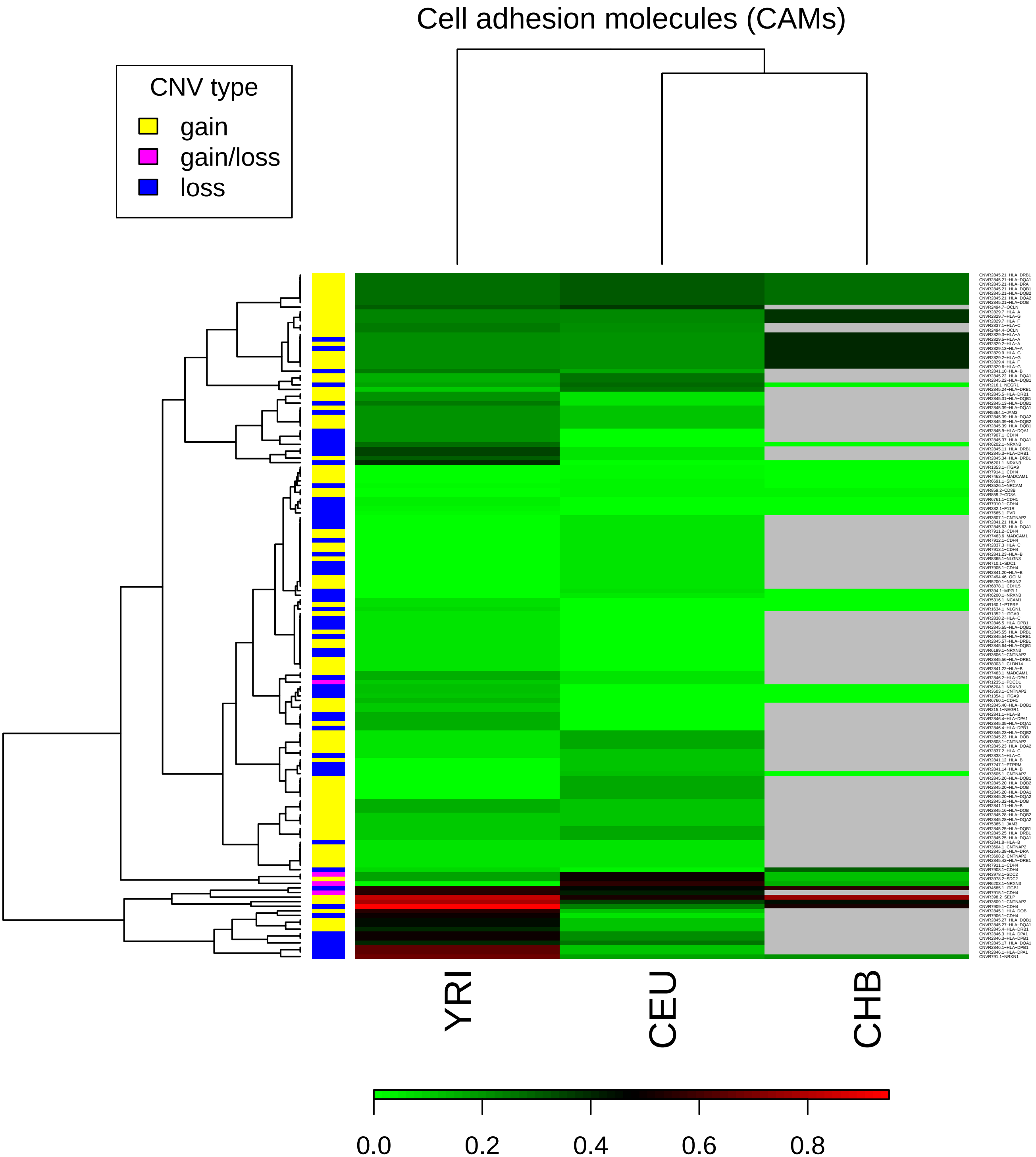

Cell adhesion molecules (CAMs)
CNV type
gain
gain/loss
loss
YRI
CEU
CHB
0.0
0.2
0.4
0.6
0.8

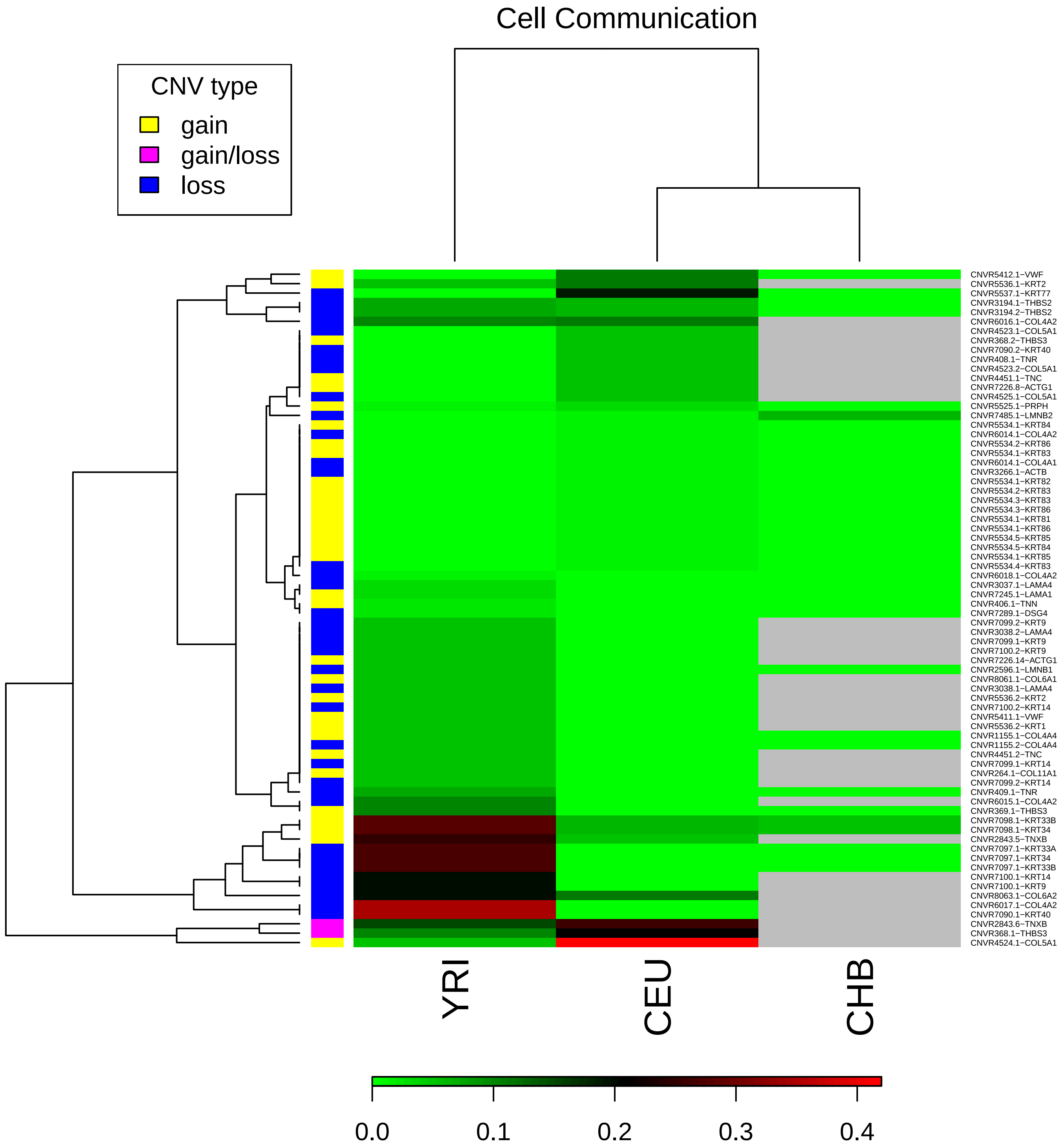
Cell Communication
CNV type
gain
gain/loss
loss
CNVR5412.1−VWF
CNVR5536.1−KRT2
CNVR5537.1−KRT77
CNVR3194.1−THBS2
CNVR3194.2−THBS2
CNVR6016.1−COL4A2
CNVR4523.1−COL5A1
CNVR368.2−THBS3
CNVR7090.2−KRT40
CNVR408.1−TNR
CNVR4523.2−COL5A1
CNVR4451.1−TNC
CNVR7226.8−ACTG1
CNVR4525.1−COL5A1
CNVR5525.1−PRPH
CNVR7485.1−LMNB2
CNVR5534.1−KRT84
CNVR6014.1−COL4A2
CNVR5534.2−KRT86
CNVR5534.1−KRT83
CNVR6014.1−COL4A1
CNVR3266.1−ACTB
CNVR5534.1−KRT82
CNVR5534.2−KRT83
CNVR5534.3−KRT83
CNVR5534.3−KRT86
CNVR5534.1−KRT81
CNVR5534.1−KRT86
CNVR5534.5−KRT85
CNVR5534.5−KRT84
CNVR5534.1−KRT85
CNVR5534.4−KRT83
CNVR6018.1−COL4A2
CNVR3037.1−LAMA4
CNVR7245.1−LAMA1
CNVR406.1−TNN
CNVR7289.1−DSG4
CNVR7099.2−KRT9
CNVR3038.2−LAMA4
CNVR7099.1−KRT9
CNVR7100.2−KRT9
CNVR7226.14−ACTG1
CNVR2596.1−LMNB1
CNVR8061.1−COL6A1
CNVR3038.1−LAMA4
CNVR5536.2−KRT2
CNVR7100.2−KRT14
CNVR5411.1−VWF
CNVR5536.2−KRT1
CNVR1155.1−COL4A4
CNVR1155.2−COL4A4
CNVR4451.2−TNC
CNVR7099.1−KRT14
CNVR264.1−COL11A1
CNVR7099.2−KRT14
CNVR409.1−TNR
CNVR6015.1−COL4A2
CNVR369.1−THBS3
CNVR7098.1−KRT33B
CNVR7098.1−KRT34
CNVR2843.5−TNXB
CNVR7097.1−KRT33A
CNVR7097.1−KRT34
CNVR7097.1−KRT33B
CNVR7100.1−KRT14
CNVR7100.1−KRT9
CNVR8063.1−COL6A2
CNVR6017.1−COL4A2
CNVR7090.1−KRT40
CNVR2843.6−TNXB
CNVR368.1−THBS3
CNVR4524.1−COL5A1
YRI
CEU
CHB
0.0
0.1
0.2
0.3
0.4

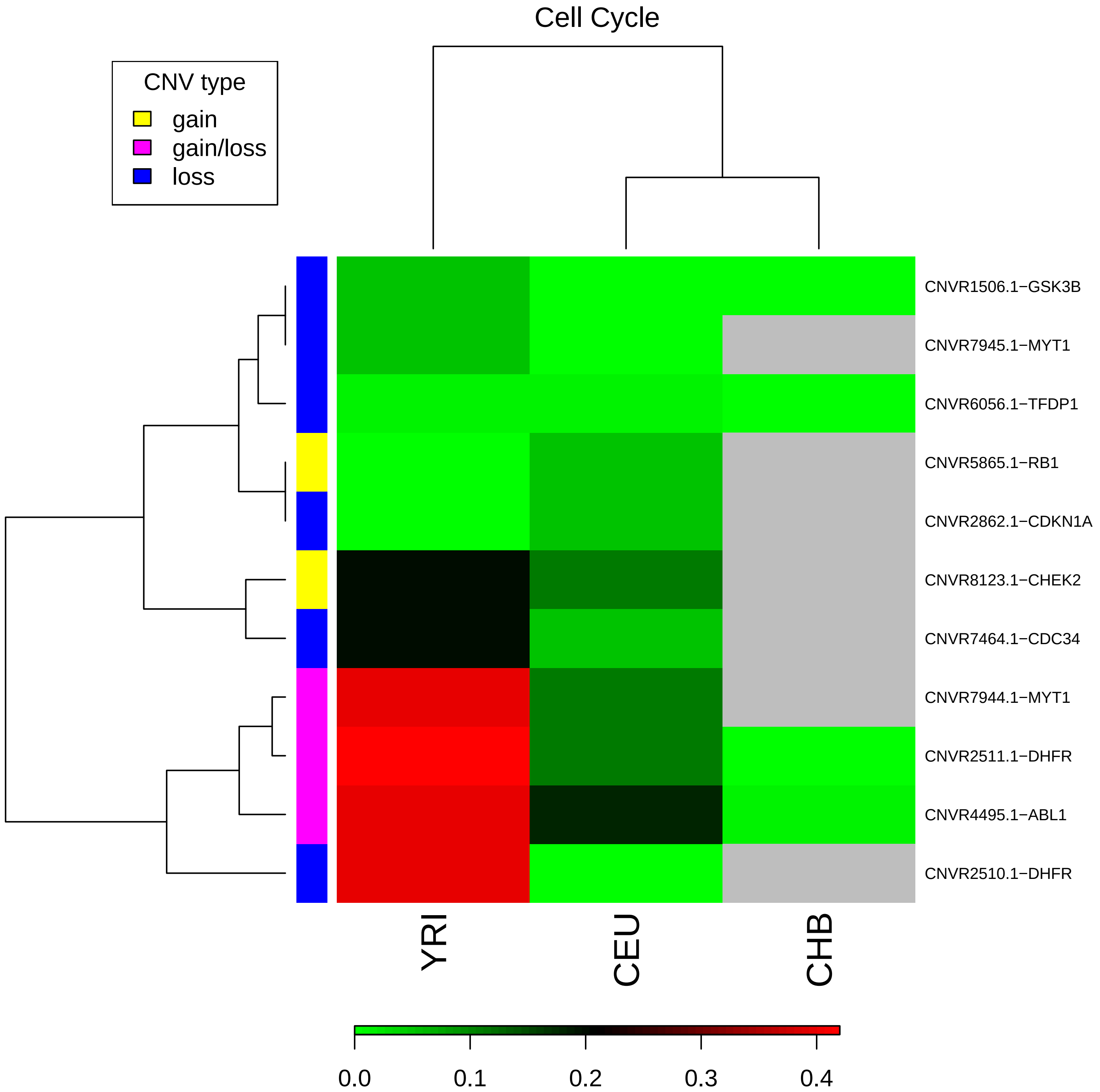

Cell Cycle
CNV type
gain
gain/loss
loss
CNVR1506.1−GSK3B
CNVR7945.1−MYT1
CNVR6056.1−TFDP1
CNVR5865.1−RB1
CNVR2862.1−CDKN1A
CNVR8123.1−CHEK2
CNVR7464.1−CDC34
CNVR7944.1−MYT1
CNVR2511.1−DHFR
CNVR4495.1−ABL1
CNVR2510.1−DHFR
YRI
CEU
CHB
0.0
0.1
0.2
0.3
0.4

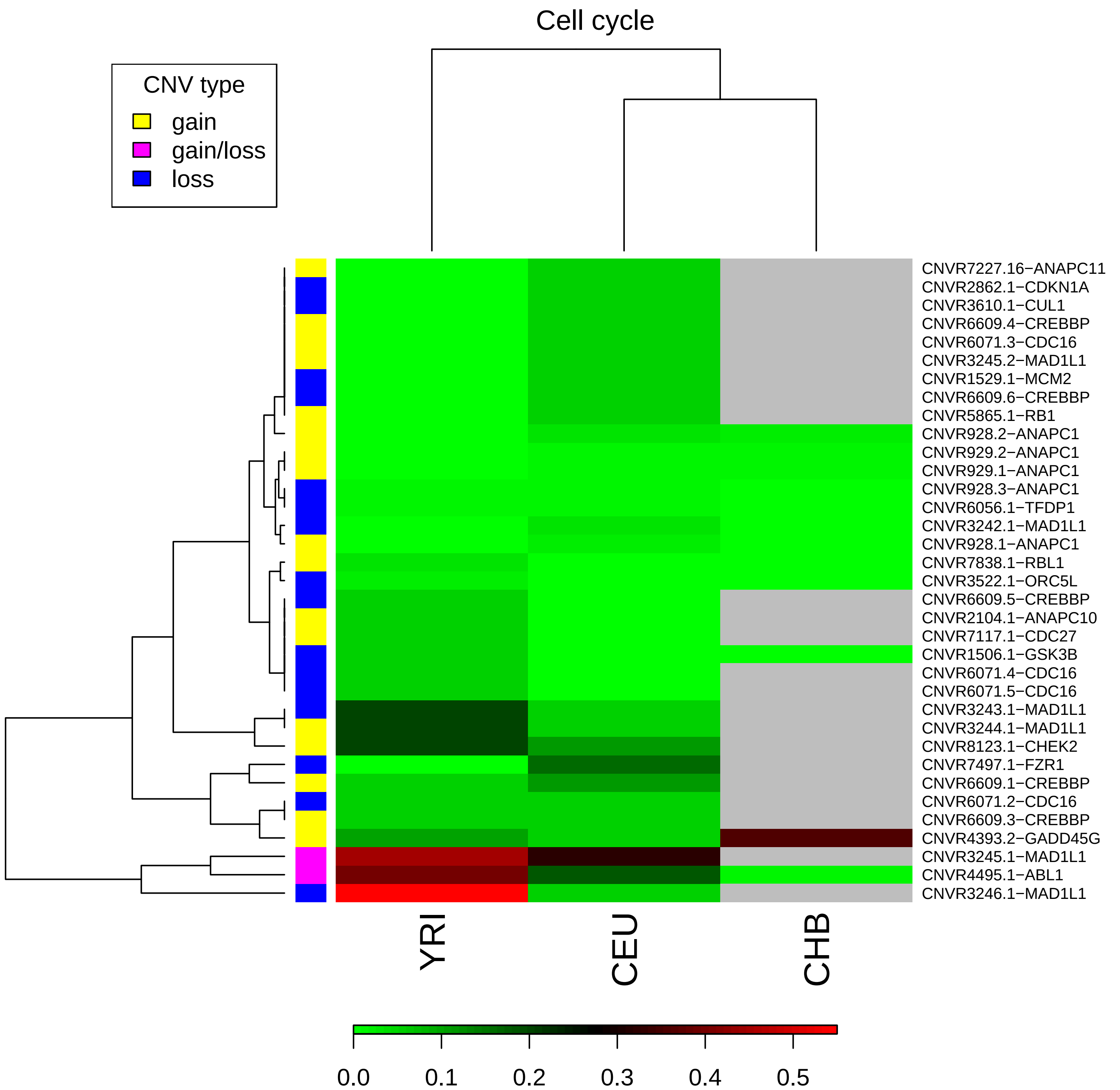
Cell cycle
CNV type
gain
gain/loss
loss
CNVR7227.16−ANAPC11
CNVR2862.1−CDKN1A
CNVR3610.1−CUL1
CNVR6609.4−CREBBP
CNVR6071.3−CDC16
CNVR3245.2−MAD1L1
CNVR1529.1−MCM2
CNVR6609.6−CREBBP
CNVR5865.1−RB1
CNVR928.2−ANAPC1
CNVR929.2−ANAPC1
CNVR929.1−ANAPC1
CNVR928.3−ANAPC1
CNVR6056.1−TFDP1
CNVR3242.1−MAD1L1
CNVR928.1−ANAPC1
CNVR7838.1−RBL1
CNVR3522.1−ORC5L
CNVR6609.5−CREBBP
CNVR2104.1−ANAPC10
CNVR7117.1−CDC27
CNVR1506.1−GSK3B
CNVR6071.4−CDC16
CNVR6071.5−CDC16
CNVR3243.1−MAD1L1
CNVR3244.1−MAD1L1
CNVR8123.1−CHEK2
CNVR7497.1−FZR1
CNVR6609.1−CREBBP
CNVR6071.2−CDC16
CNVR6609.3−CREBBP
CNVR4393.2−GADD45G
CNVR3245.1−MAD1L1
CNVR4495.1−ABL1
CNVR3246.1−MAD1L1
YRI
CEU
CHB
0.0
0.1
0.2
0.3
0.4
0.5

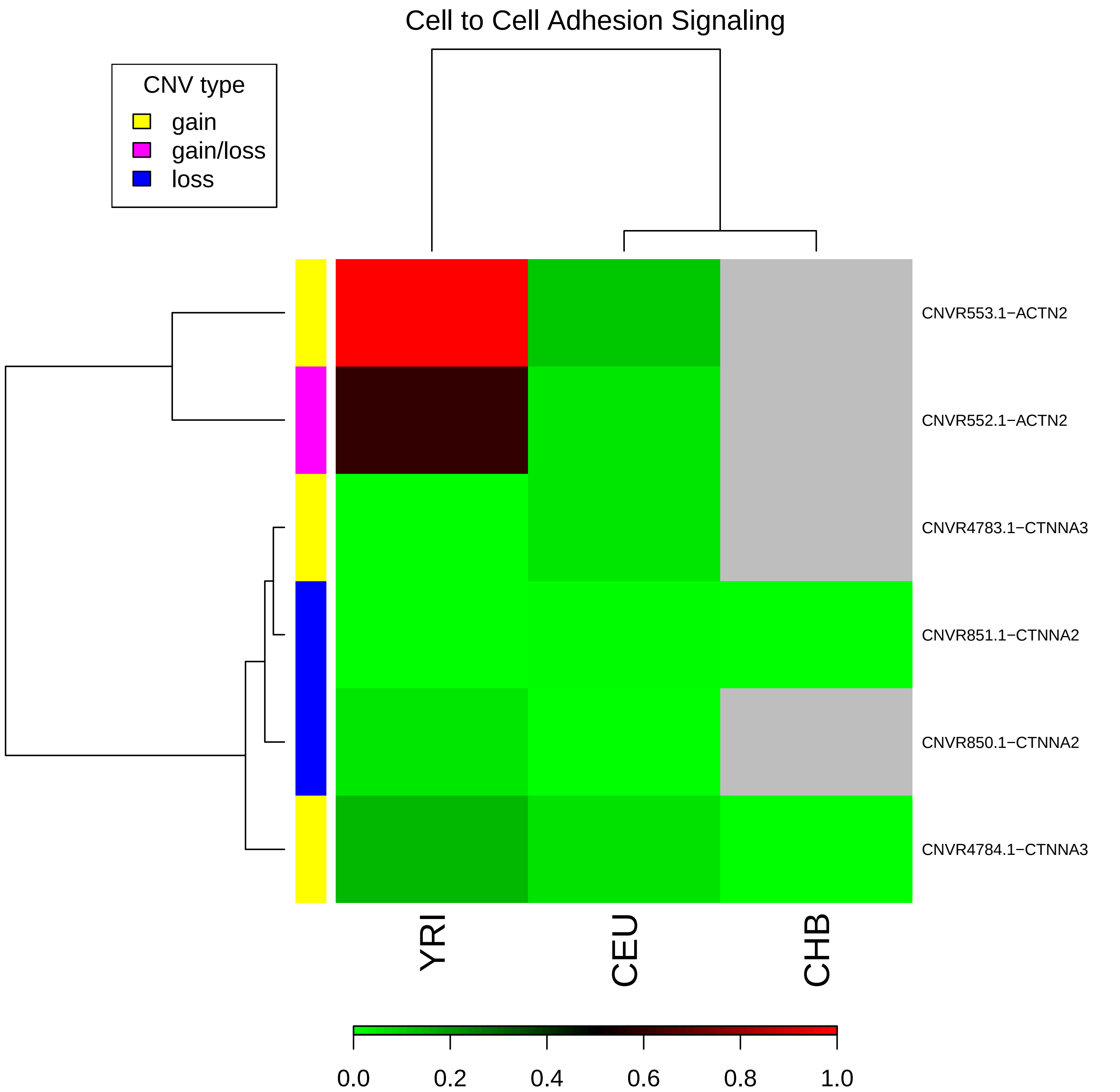
Cell to Cell Adhesion Signaling
CNV type
gain
gain/loss
loss
CNVR553.1−ACTN2
CNVR552.1−ACTN2
CNVR4783.1−CTNNA3
CNVR851.1−CTNNA2
CNVR850.1−CTNNA2
CNVR4784.1−CTNNA3
YRI
CEU
CHB
0.0
0.2
0.4
0.6
0.8
1.0

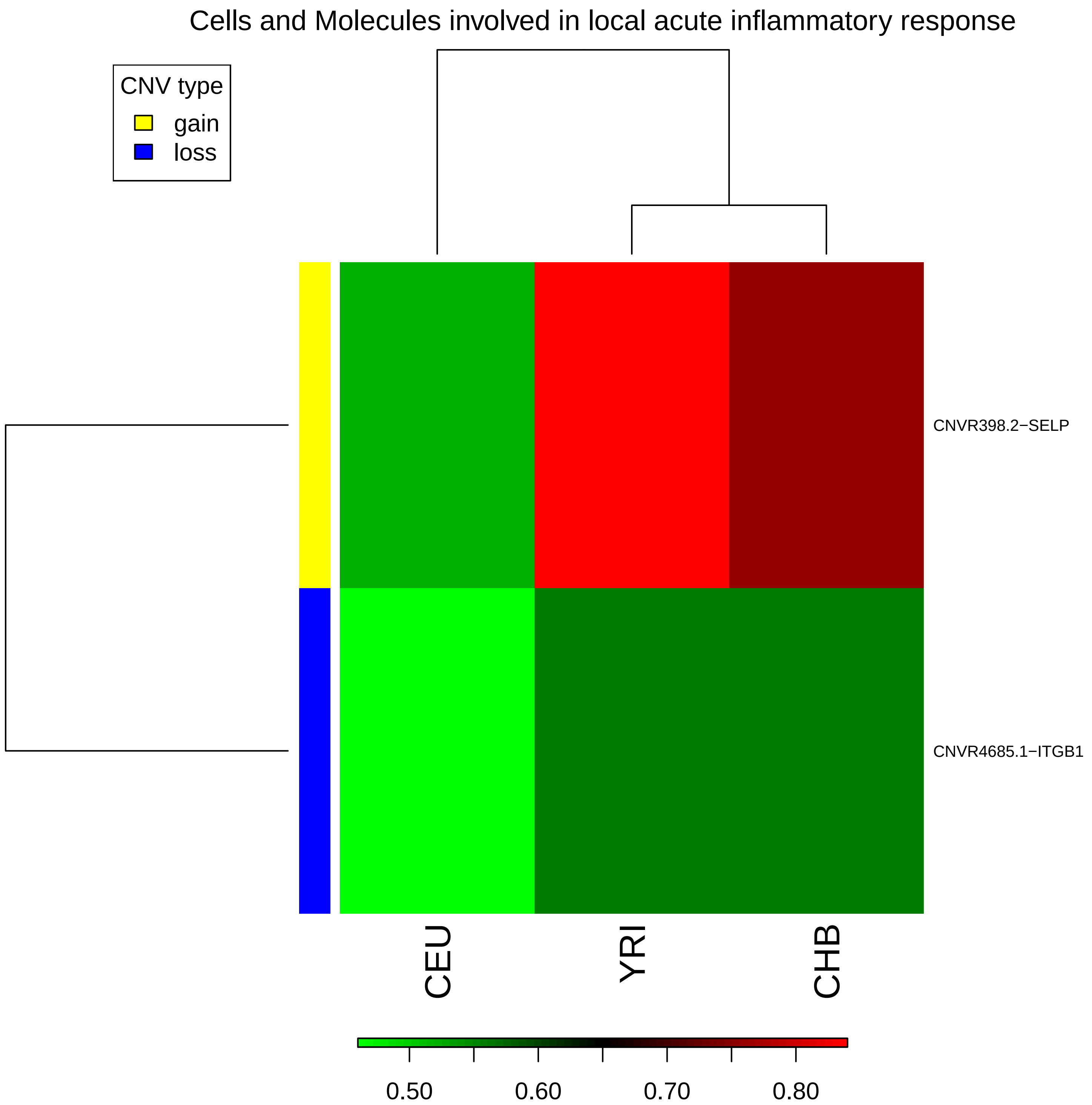
Cells and Molecules involved in local acute inflammatory response
CNV type
gain
loss
CNVR398.2−SELP
CNVR4685.1−ITGB1
CEU
YRI
CHB
0.50
0.60
0.70
0.80

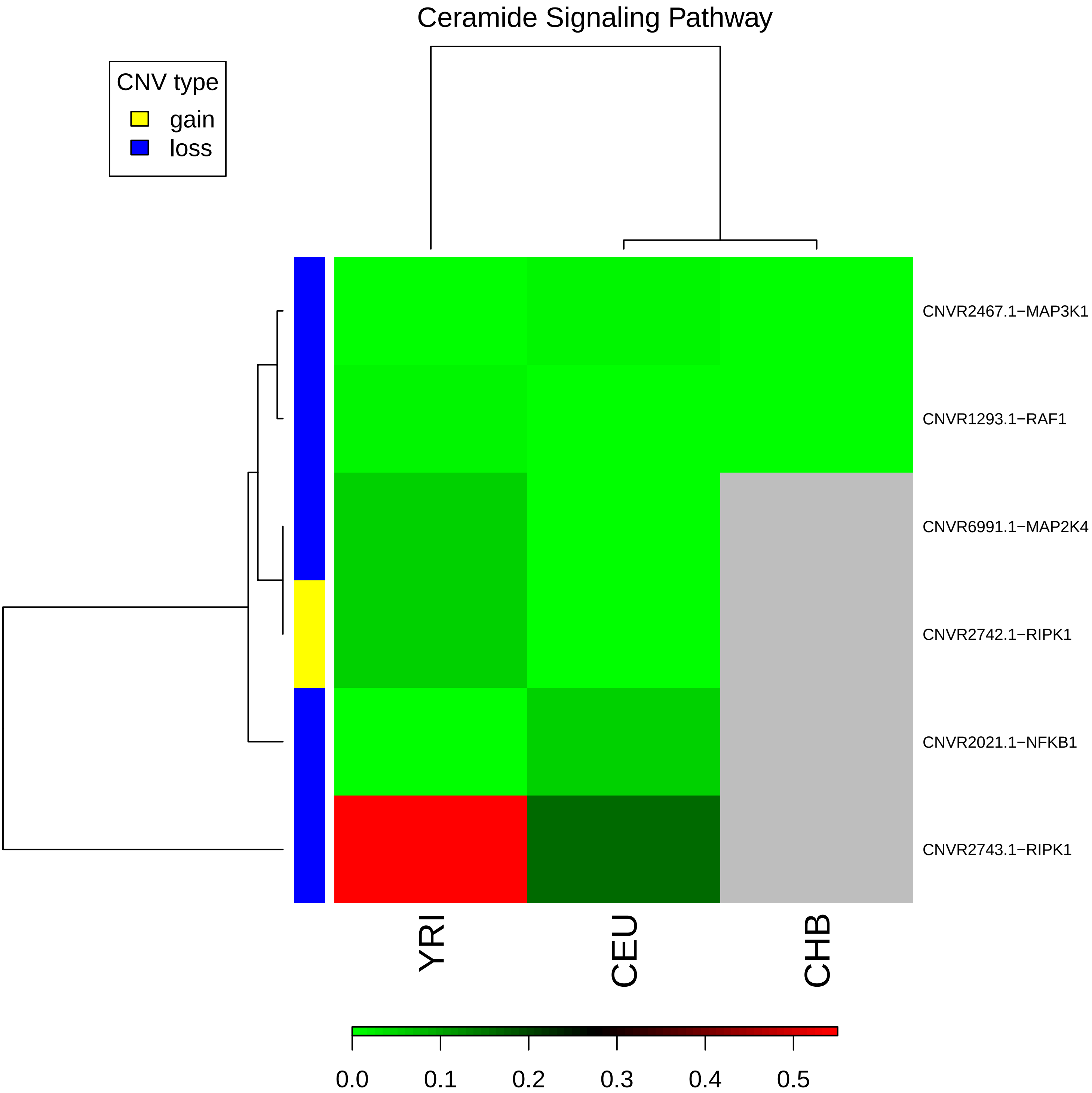

Ceramide Signaling Pathway
CNV type
gain
loss
CNVR2467.1−MAP3K1
CNVR1293.1−RAF1
CNVR6991.1−MAP2K4
CNVR2742.1−RIPK1
CNVR2021.1−NFKB1
CNVR2743.1−RIPK1
YRI
CEU
CHB
0.0
0.1
0.2
0.3
0.4
0.5

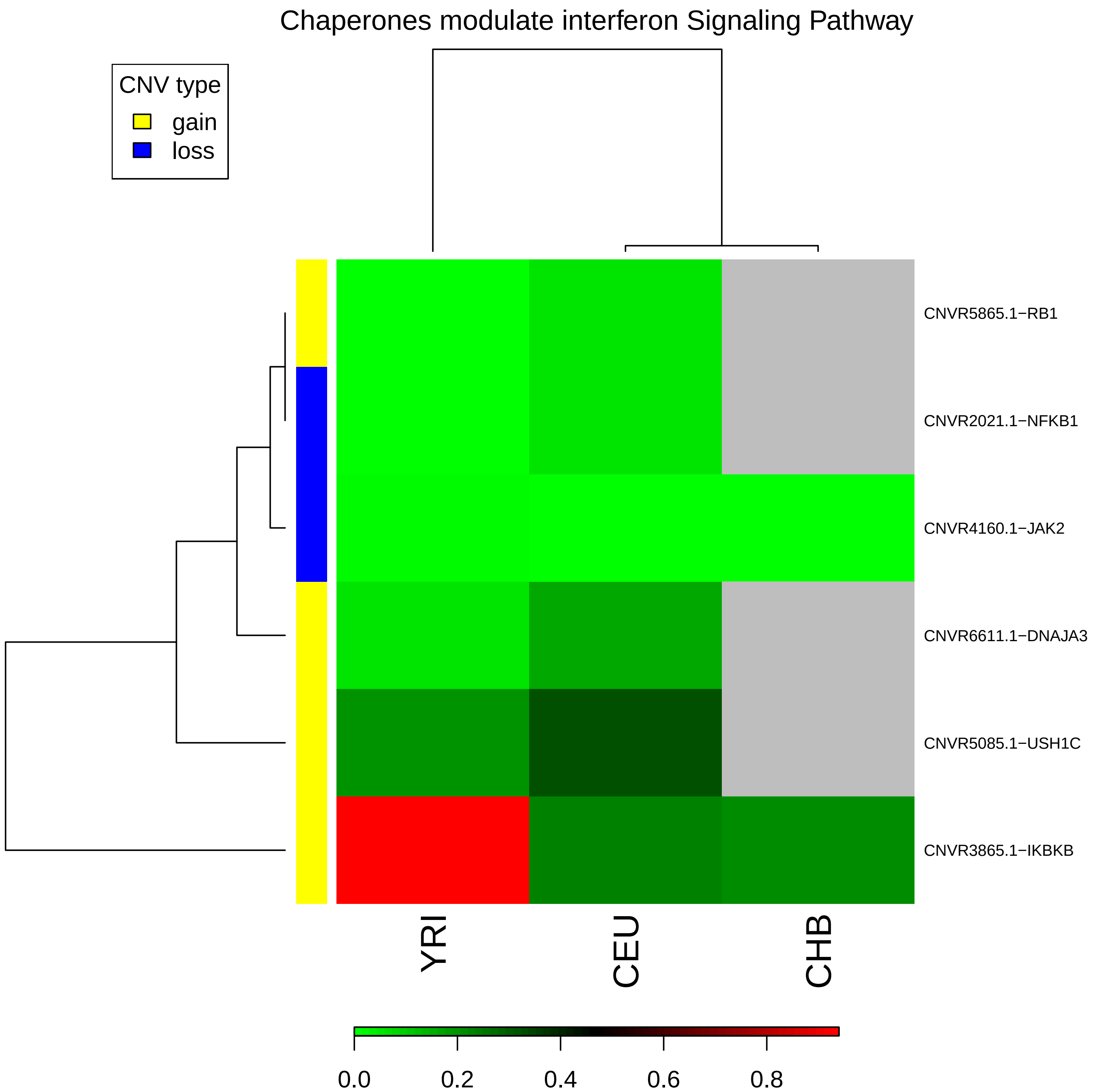

Chaperones modulate interferon Signaling Pathway
CNV type
gain
loss
CNVR5865.1−RB1
CNVR2021.1−NFKB1
CNVR4160.1−JAK2
CNVR6611.1−DNAJA3
CNVR5085.1−USH1C
CNVR3865.1−IKBKB
YRI
CEU
CHB
0.0
0.2
0.4
0.6
0.8

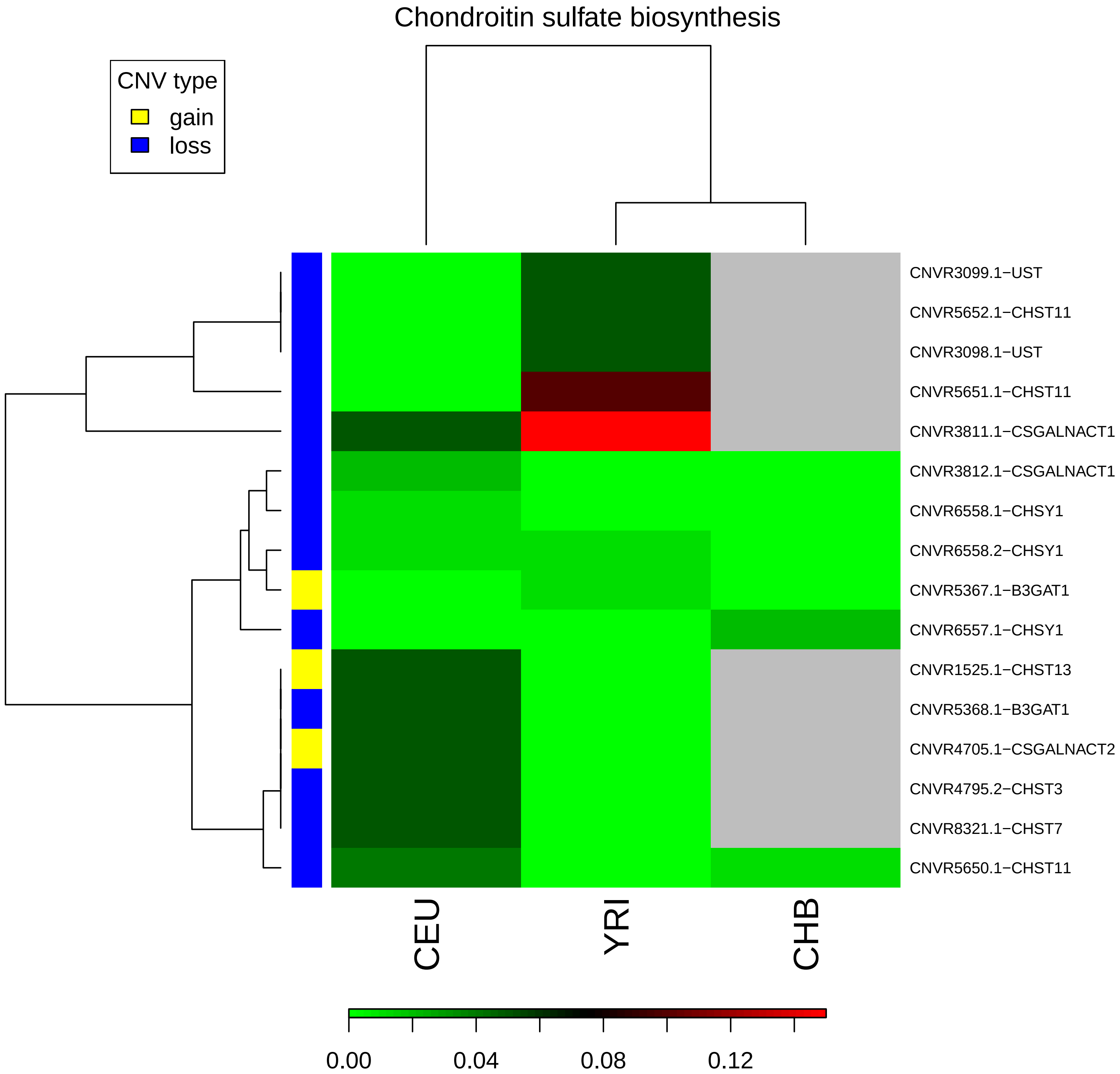

Chondroitin sulfate biosynthesis
CNV type
gain
loss
CNVR3099.1−UST
CNVR5652.1−CHST11
CNVR3098.1−UST
CNVR5651.1−CHST11
CNVR3811.1−CSGALNACT1
CNVR3812.1−CSGALNACT1
CNVR6558.1−CHSY1
CNVR6558.2−CHSY1
CNVR5367.1−B3GAT1
CNVR6557.1−CHSY1
CNVR1525.1−CHST13
CNVR5368.1−B3GAT1
CNVR4705.1−CSGALNACT2
CNVR4795.2−CHST3
CNVR8321.1−CHST7
CNVR5650.1−CHST11
CEU
YRI
CHB
0.00
0.04
0.08
0.12

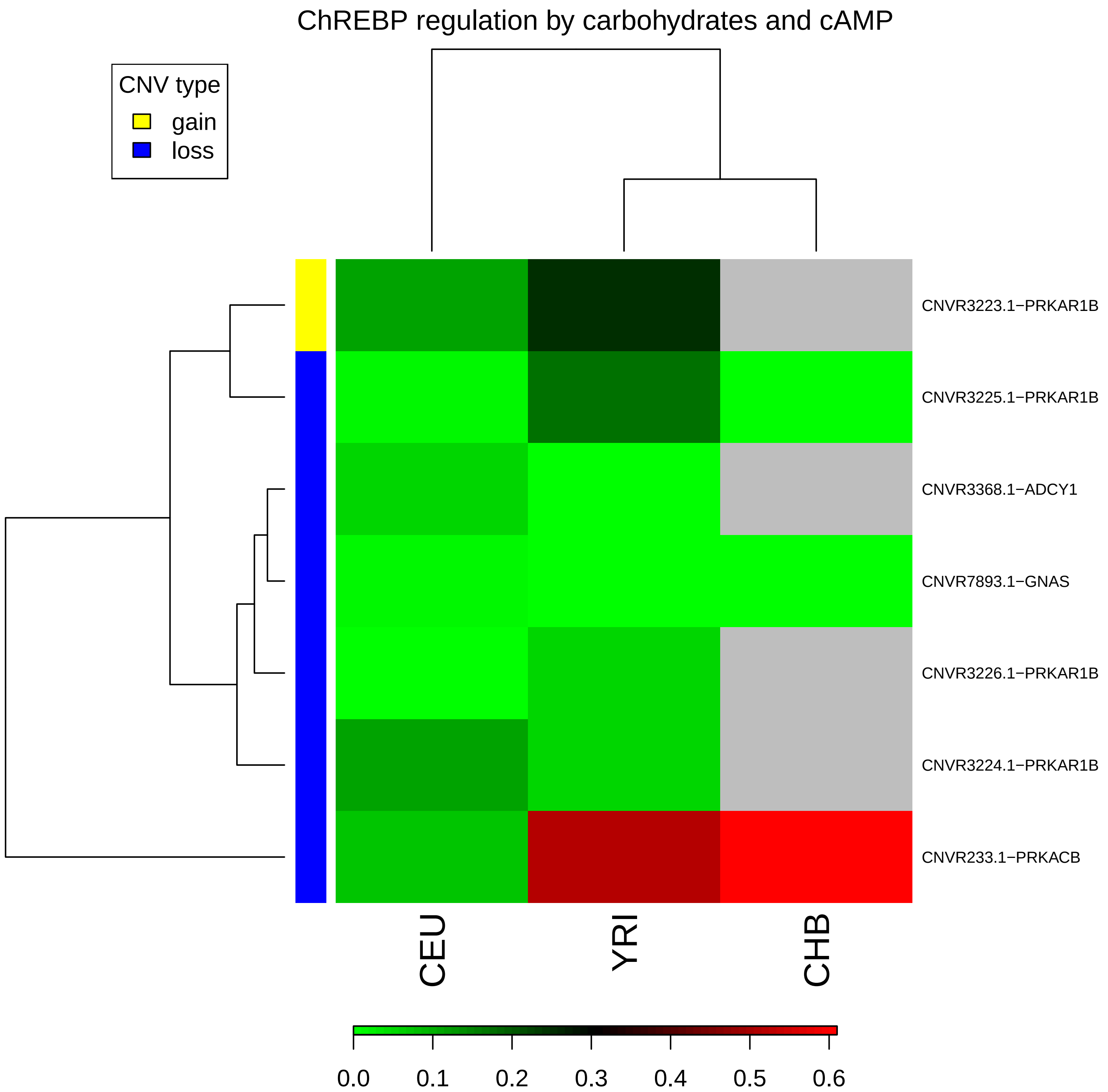
ChREBP regulation by carbohydrates and cAMP
CNV type
gain
loss
CNVR3223.1−PRKAR1B
CNVR3225.1−PRKAR1B
CNVR3368.1−ADCY1
CNVR7893.1−GNAS
CNVR3226.1−PRKAR1B
CNVR3224.1−PRKAR1B
CNVR233.1−PRKACB
CEU
YRI
CHB
0.0
0.1
0.2
0.3
0.4
0.5
0.6

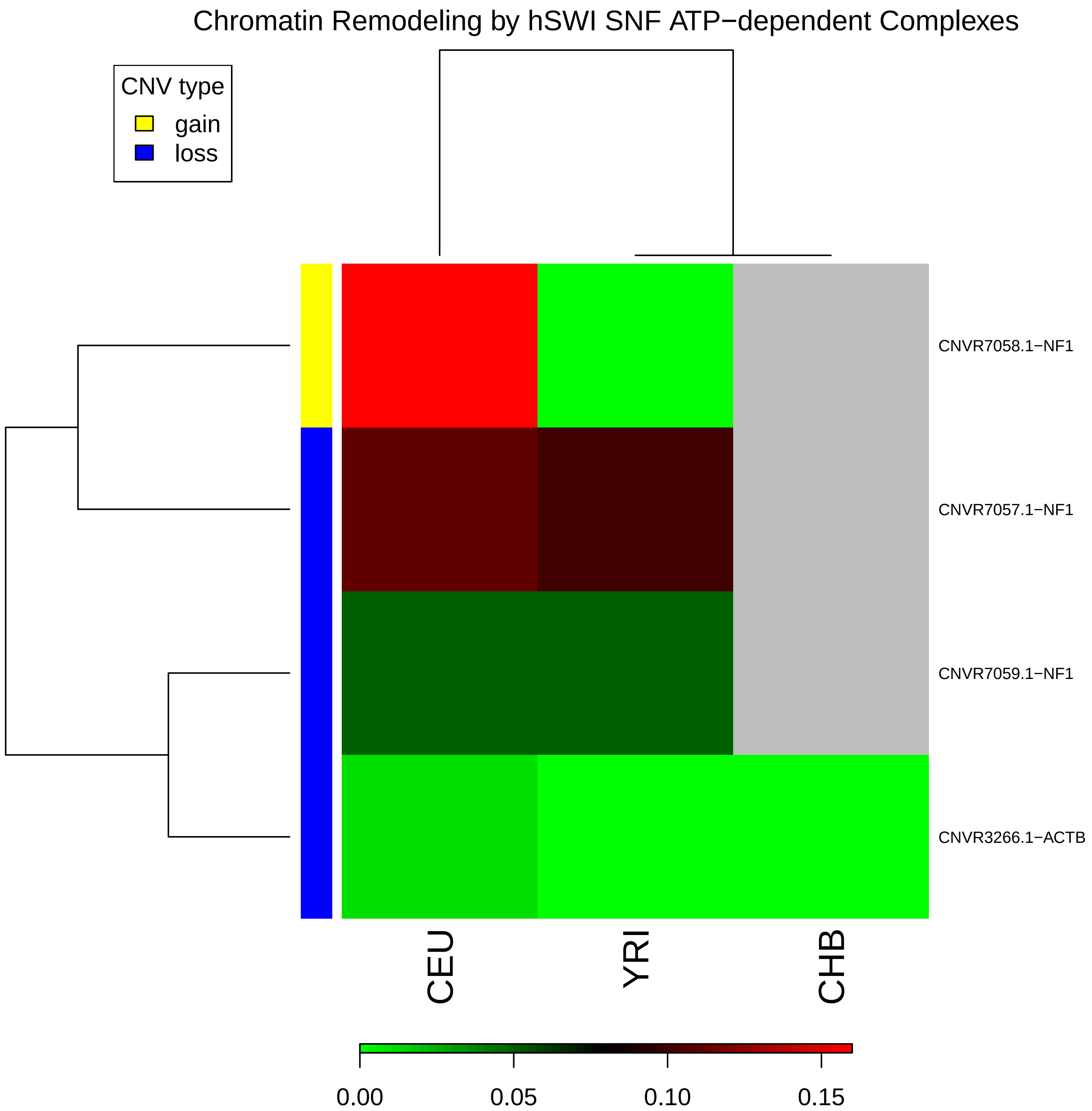

Chromatin Remodeling by hSWI SNF ATP−dependent Complexes
CNV type
gain
loss
CNVR7058.1−NF1
CNVR7057.1−NF1
CNVR7059.1−NF1
CNVR3266.1−ACTB
CEU
YRI
CHB
0.00
0.05
0.10
0.15

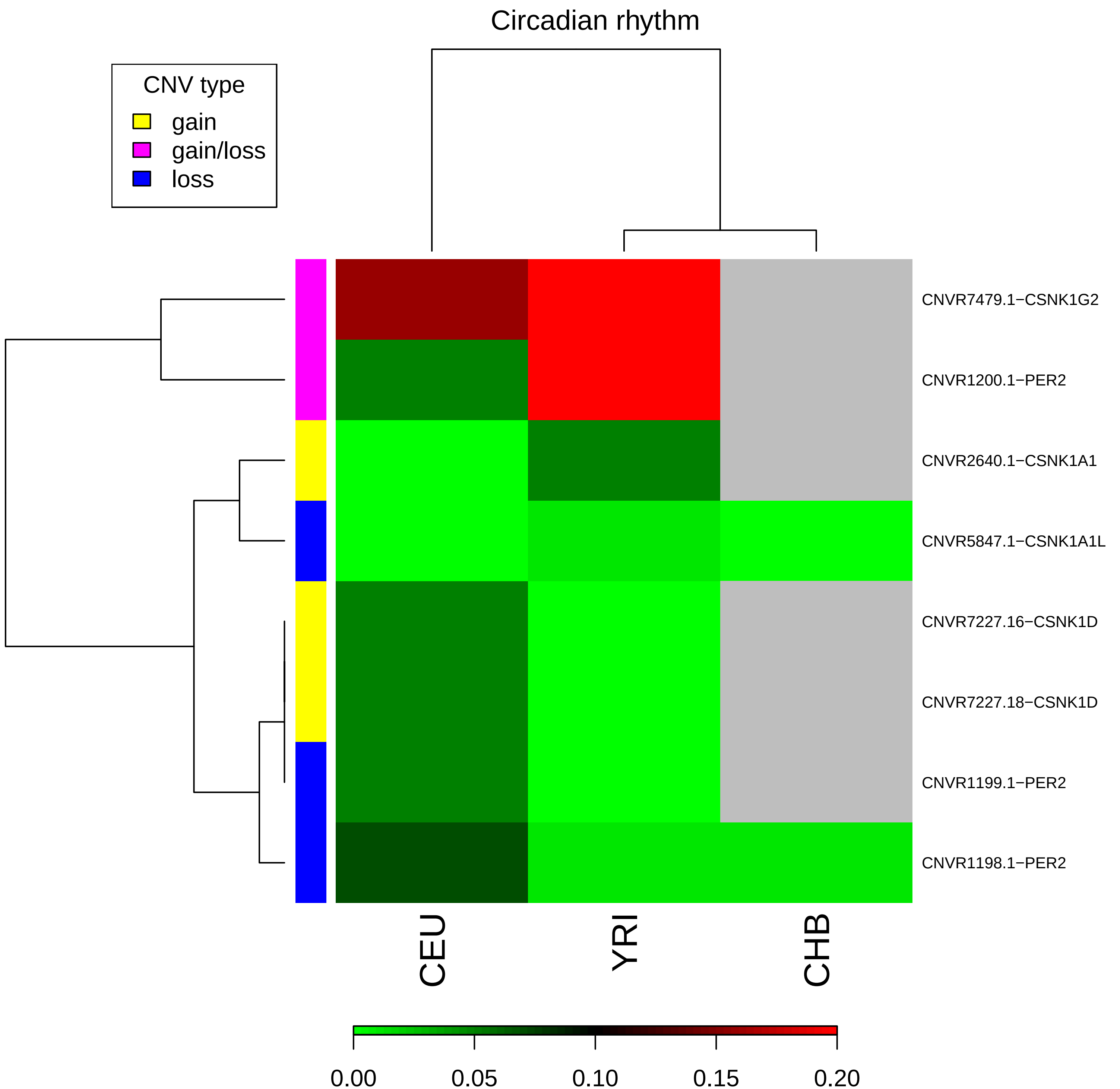

Circadian rhythm
CNV type
gain
gain/loss
loss
CNVR7479.1−CSNK1G2
CNVR1200.1−PER2
CNVR2640.1−CSNK1A1
CNVR5847.1−CSNK1A1L
CNVR7227.16−CSNK1D
CNVR7227.18−CSNK1D
CNVR1199.1−PER2
CNVR1198.1−PER2
CEU
YRI
CHB
0.00
0.05
0.10
0.15
0.20

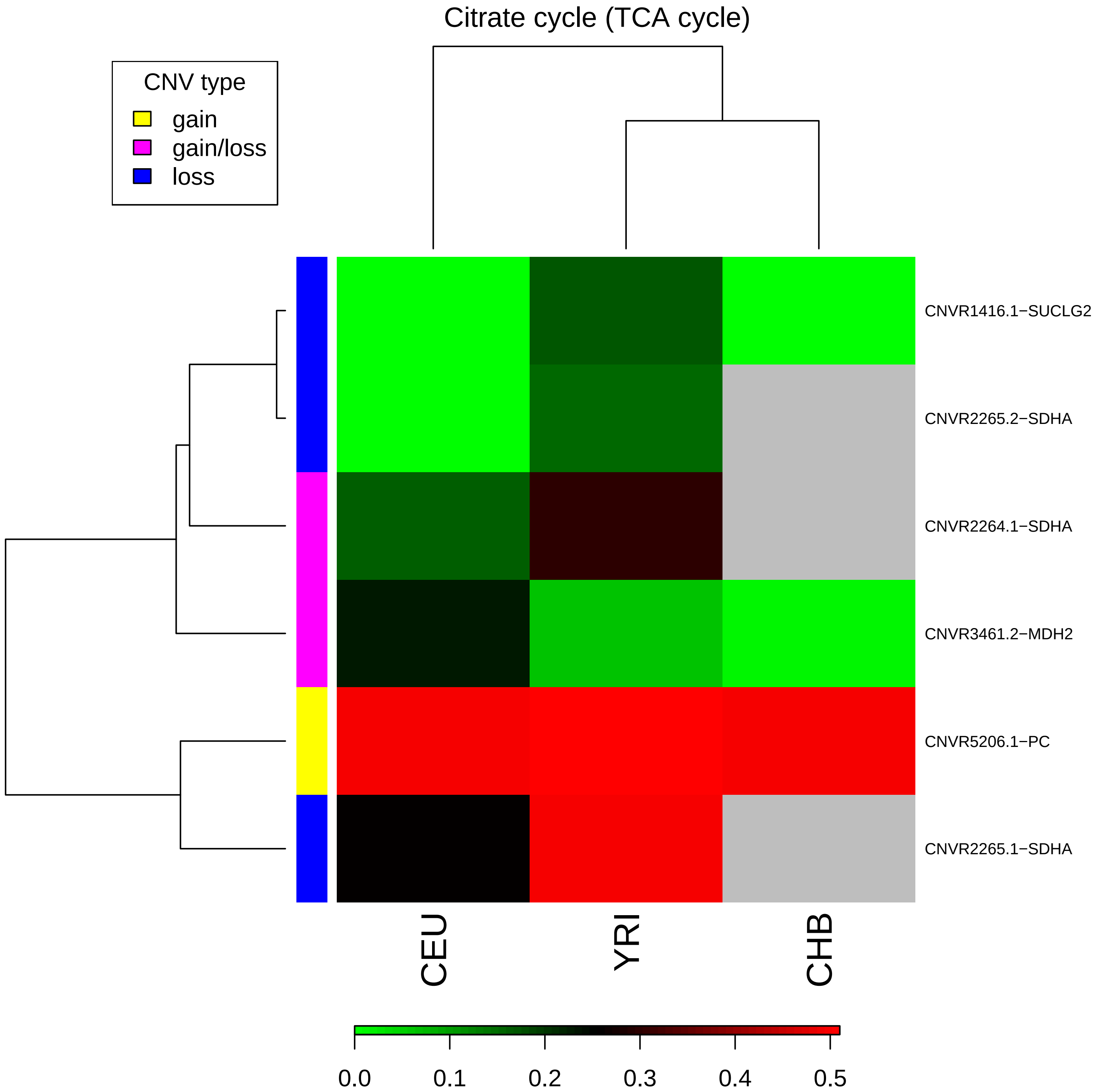

Citrate cycle (TCA cycle)
CNV type
gain
gain/loss
loss
CNVR1416.1−SUCLG2
CNVR2265.2−SDHA
CNVR2264.1−SDHA
CNVR3461.2−MDH2
CNVR5206.1−PC
CNVR2265.1−SDHA
CEU
YRI
CHB
0.0
0.1
0.2
0.3
0.4
0.5

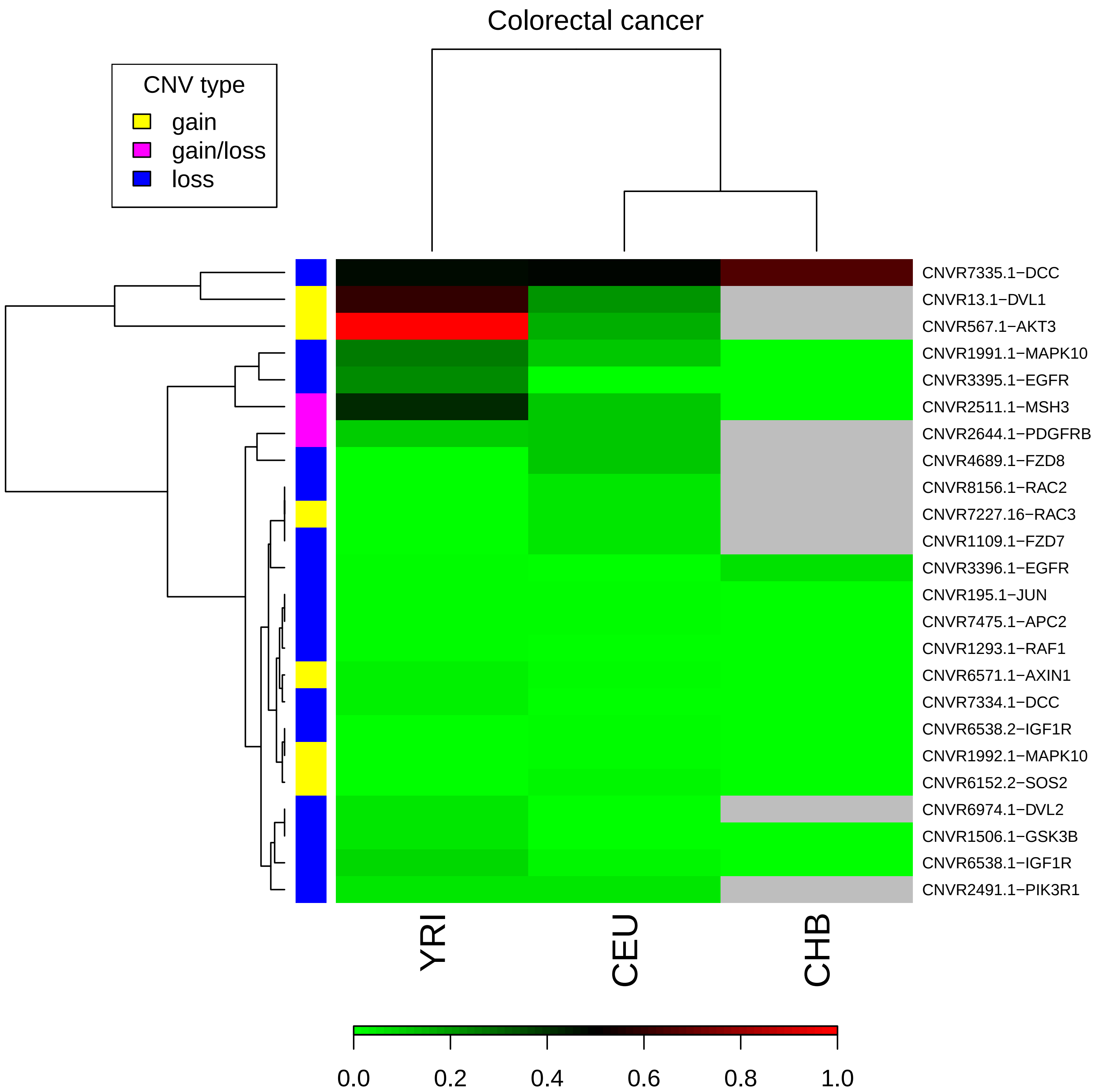

Colorectal cancer
CNV type
gain
gain/loss
loss
CNVR7335.1−DCC
CNVR13.1−DVL1
CNVR567.1−AKT3
CNVR1991.1−MAPK10
CNVR3395.1−EGFR
CNVR2511.1−MSH3
CNVR2644.1−PDGFRB
CNVR4689.1−FZD8
CNVR8156.1−RAC2
CNVR7227.16−RAC3
CNVR1109.1−FZD7
CNVR3396.1−EGFR
CNVR195.1−JUN
CNVR7475.1−APC2
CNVR1293.1−RAF1
CNVR6571.1−AXIN1
CNVR7334.1−DCC
CNVR6538.2−IGF1R
CNVR1992.1−MAPK10
CNVR6152.2−SOS2
CNVR6974.1−DVL2
CNVR1506.1−GSK3B
CNVR6538.1−IGF1R
CNVR2491.1−PIK3R1
YRI
CEU
CHB
0.0
0.2
0.4
0.6
0.8
1.0

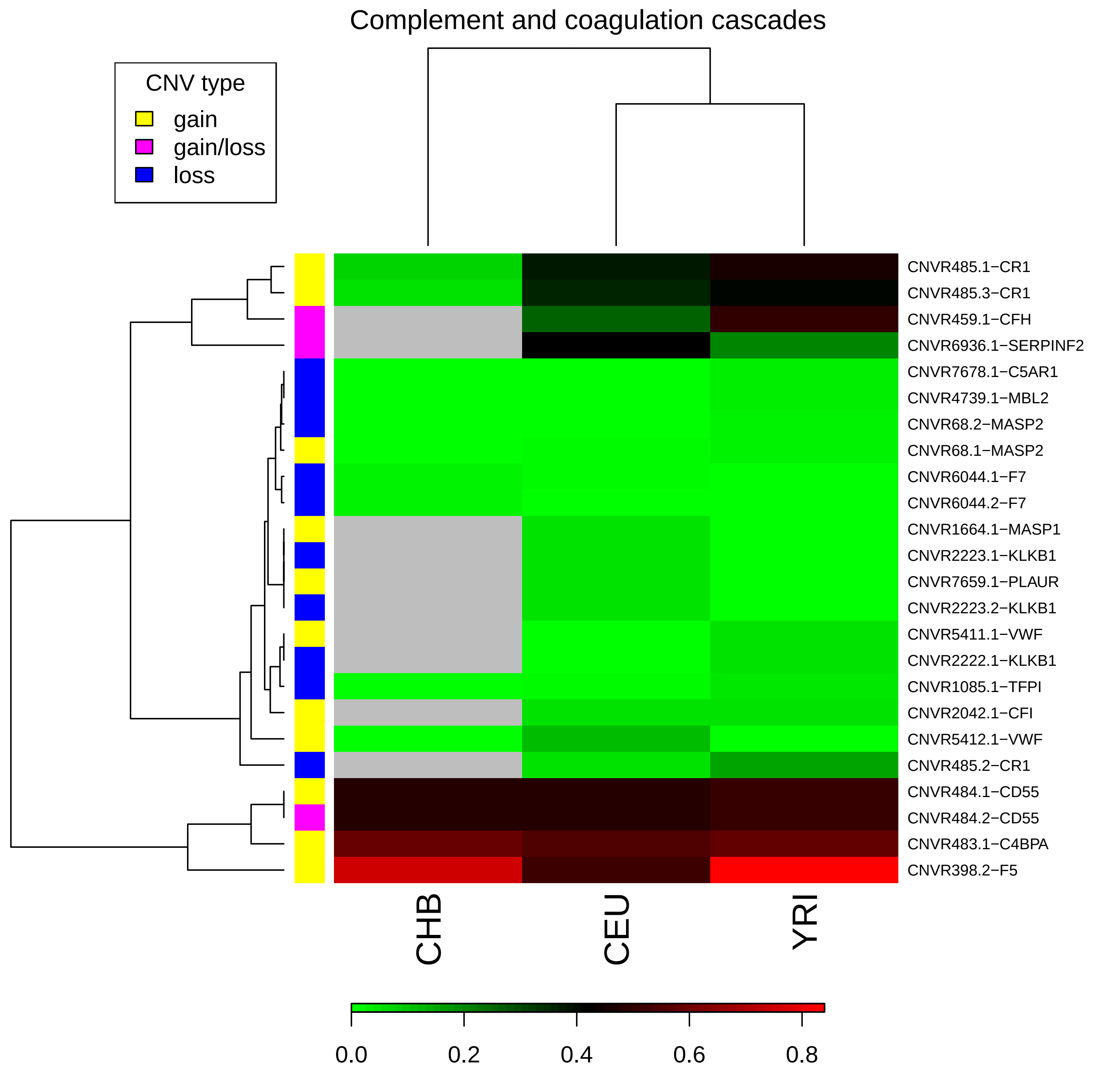

Complement and coagulation cascades
CNV type
gain
gain/loss
loss
CNVR485.1−CR1
CNVR485.3−CR1
CNVR459.1−CFH
CNVR6936.1−SERPINF2
CNVR7678.1−C5AR1
CNVR4739.1−MBL2
CNVR68.2−MASP2
CNVR68.1−MASP2
CNVR6044.1−F7
CNVR6044.2−F7
CNVR1664.1−MASP1
CNVR2223.1−KLKB1
CNVR7659.1−PLAUR
CNVR2223.2−KLKB1
CNVR5411.1−VWF
CNVR2222.1−KLKB1
CNVR1085.1−TFPI
CNVR2042.1−CFI
CNVR5412.1−VWF
CNVR485.2−CR1
CNVR484.1−CD55
CNVR484.2−CD55
CNVR483.1−C4BPA
CNVR398.2−F5
CHB
CEU
YRI
0.0
0.2
0.4
0.6
0.8

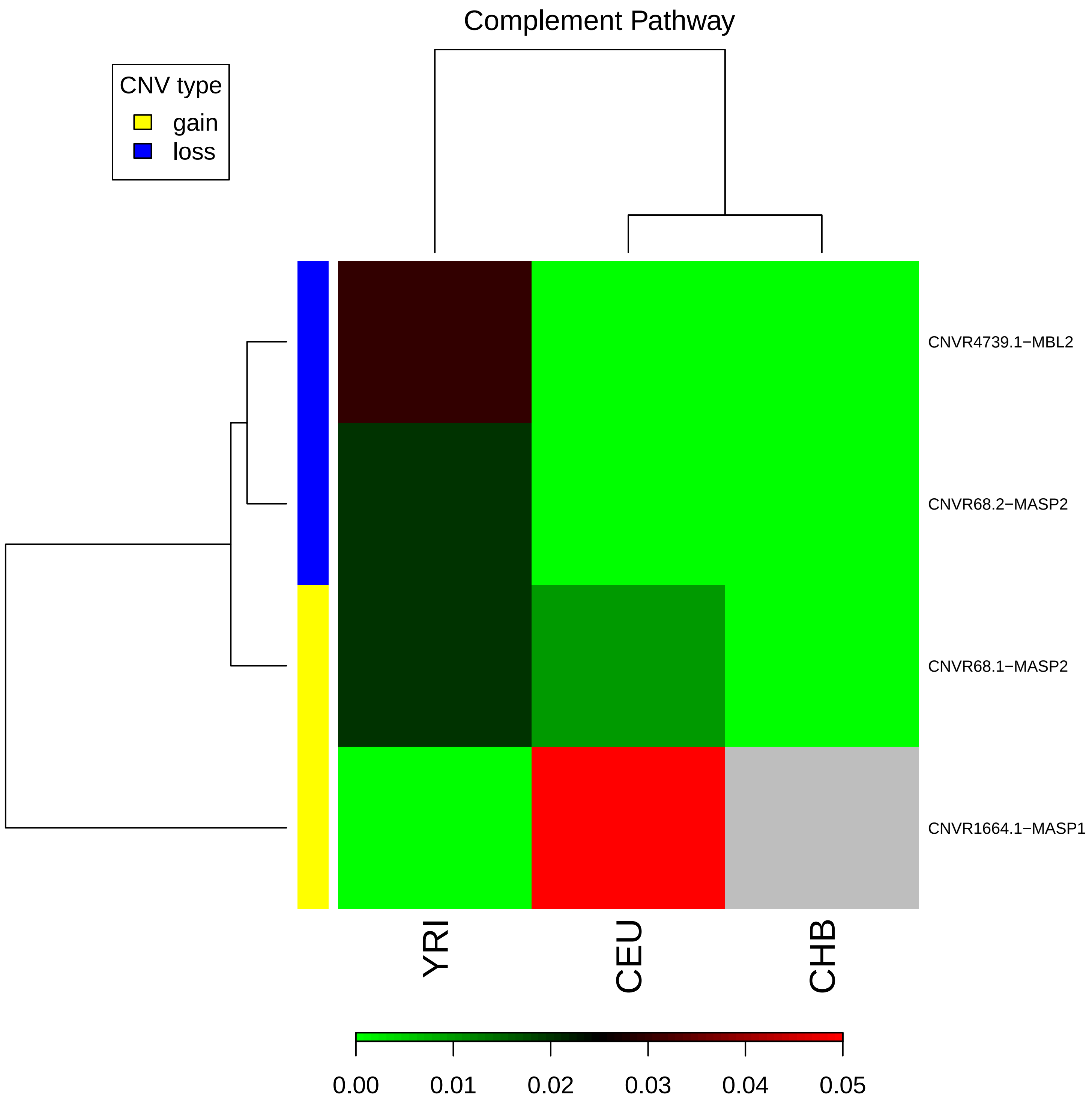
Complement Pathway
CNV type
gain
loss
CNVR4739.1−MBL2
CNVR68.2−MASP2
CNVR68.1−MASP2
CNVR1664.1−MASP1
YRI
CEU
CHB
0.00
0.01
0.02
0.03
0.04
0.05

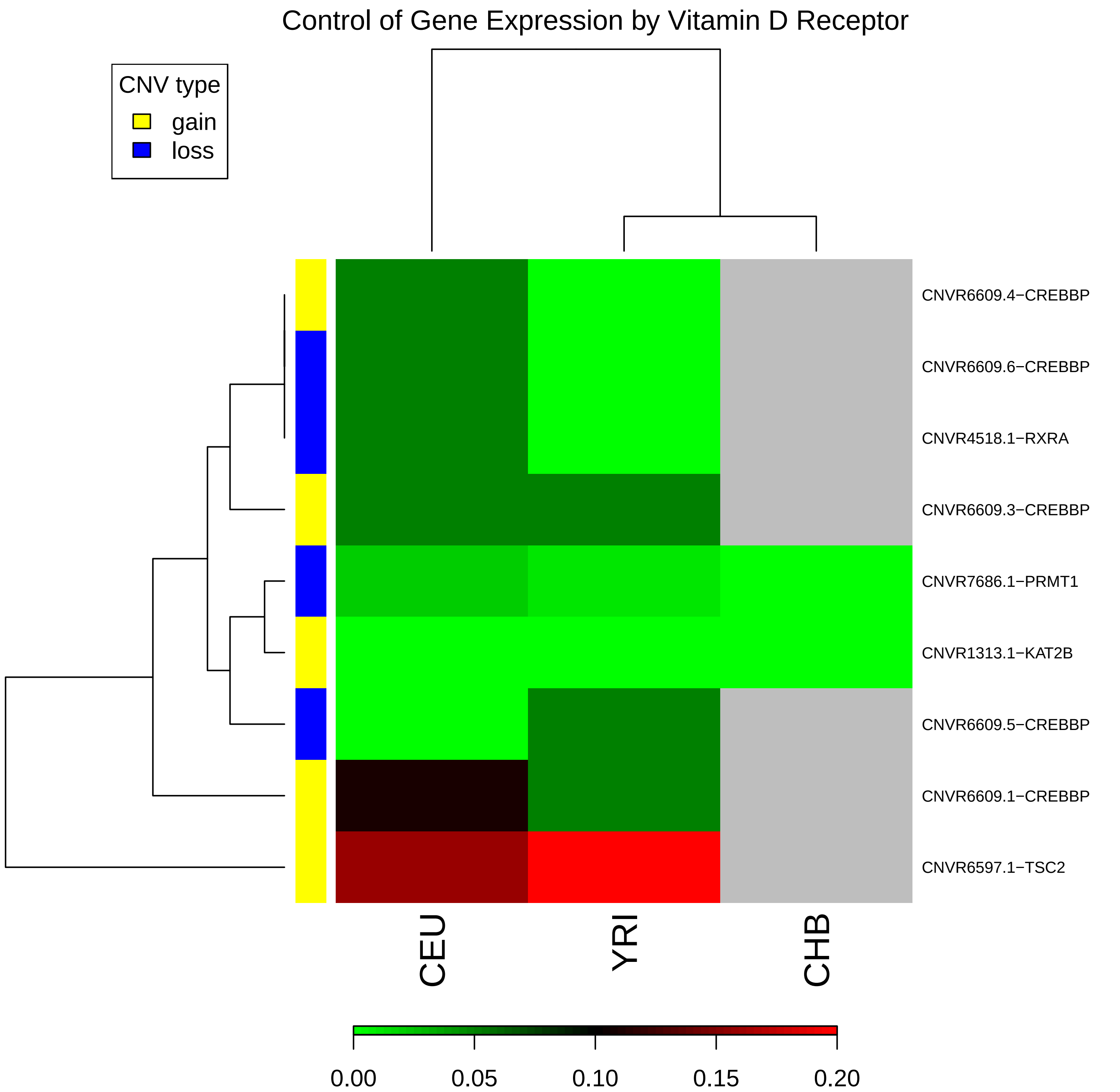
Control of Gene Expression by Vitamin D Receptor
CNV type
gain
loss
CNVR6609.4−CREBBP
CNVR6609.6−CREBBP
CNVR4518.1−RXRA
CNVR6609.3−CREBBP
CNVR7686.1−PRMT1
CNVR1313.1−KAT2B
CNVR6609.5−CREBBP
CNVR6609.1−CREBBP
CNVR6597.1−TSC2
CEU
YRI
CHB
0.00
0.05
0.10
0.15
0.20

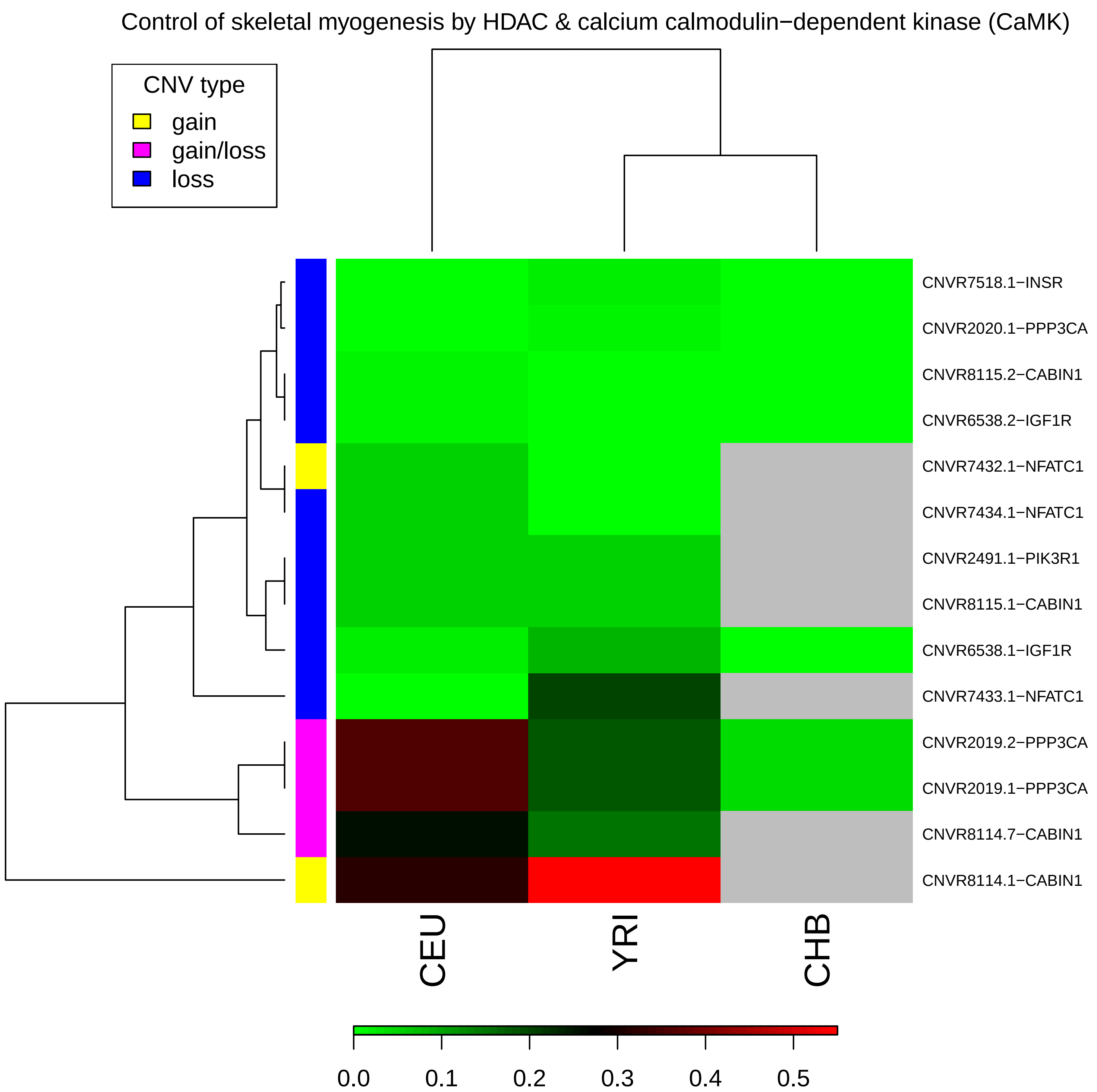

Control of skeletal myogenesis by HDAC & calcium calmodulin−dependent kinase (CaMK)
CNV type
gain
gain/loss
loss
CNVR7518.1−INSR
CNVR2020.1−PPP3CA
CNVR8115.2−CABIN1
CNVR6538.2−IGF1R
CNVR7432.1−NFATC1
CNVR7434.1−NFATC1
CNVR2491.1−PIK3R1
CNVR8115.1−CABIN1
CNVR6538.1−IGF1R
CNVR7433.1−NFATC1
CNVR2019.2−PPP3CA
CNVR2019.1−PPP3CA
CNVR8114.7−CABIN1
CNVR8114.1−CABIN1
CEU
YRI
CHB
0.0
0.1
0.2
0.3
0.4
0.5

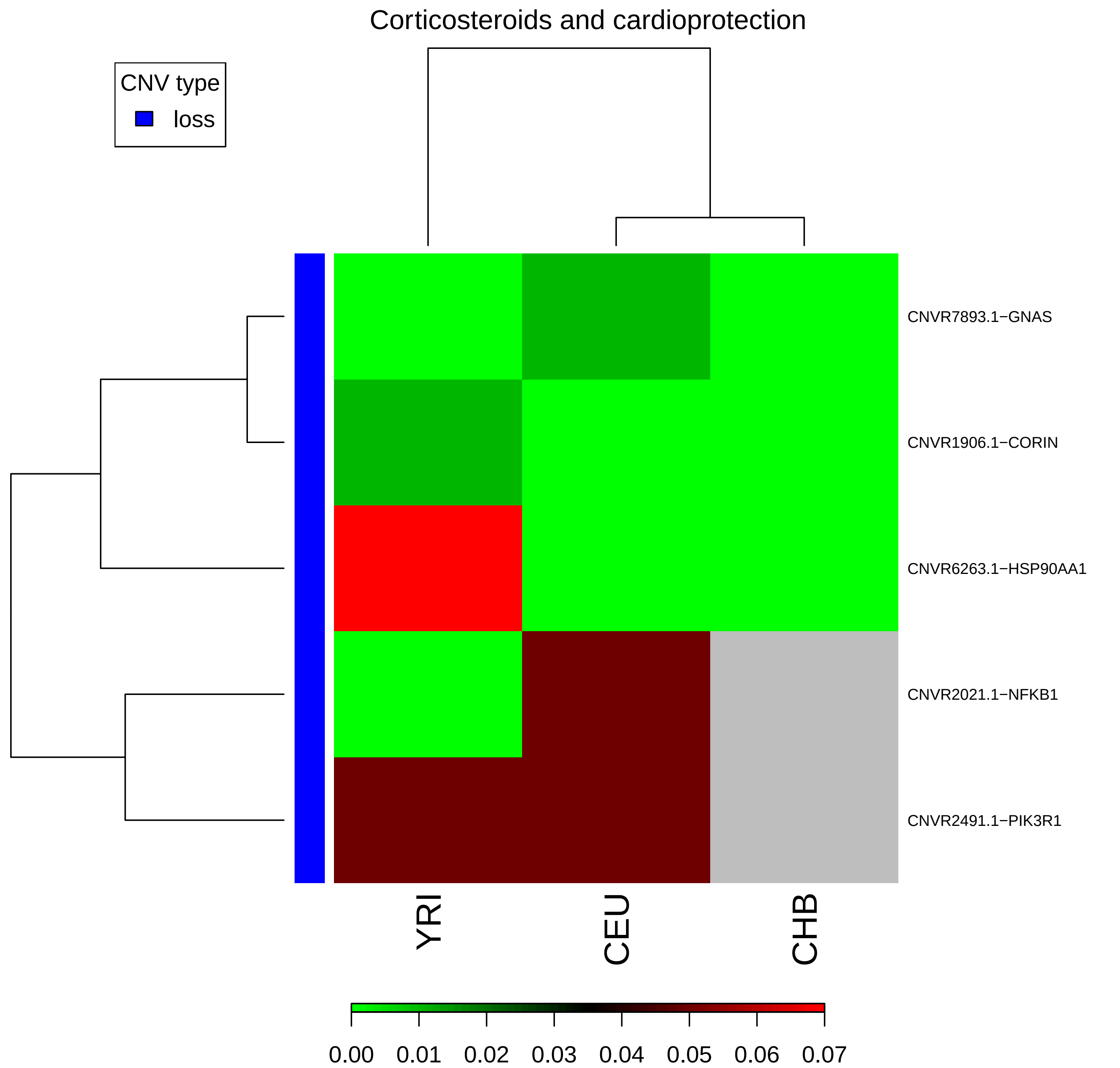
Corticosteroids and cardioprotection
CNV type
loss
CNVR7893.1−GNAS
CNVR1906.1−CORIN
CNVR6263.1−HSP90AA1
CNVR2021.1−NFKB1
CNVR2491.1−PIK3R1
YRI
CEU
CHB
0.00
0.01
0.02
0.03
0.04
0.05
0.06
0.07

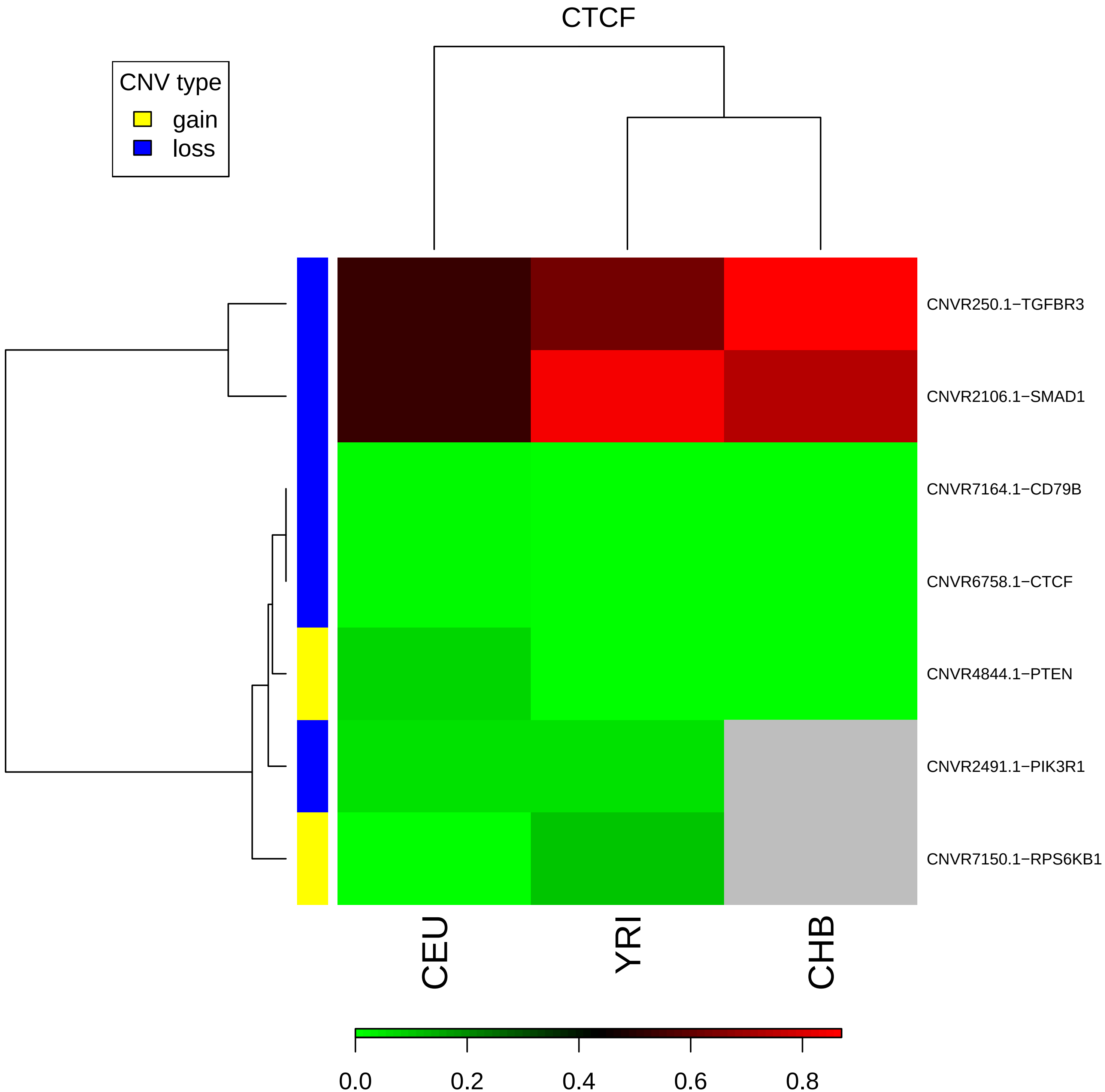
CTCF
CNV type
gain
loss
CNVR250.1−TGFBR3
CNVR2106.1−SMAD1
CNVR7164.1−CD79B
CNVR6758.1−CTCF
CNVR4844.1−PTEN
CNVR2491.1−PIK3R1
CNVR7150.1−RPS6KB1
CEU
YRI
CHB
0.0
0.2
0.4
0.6
0.8

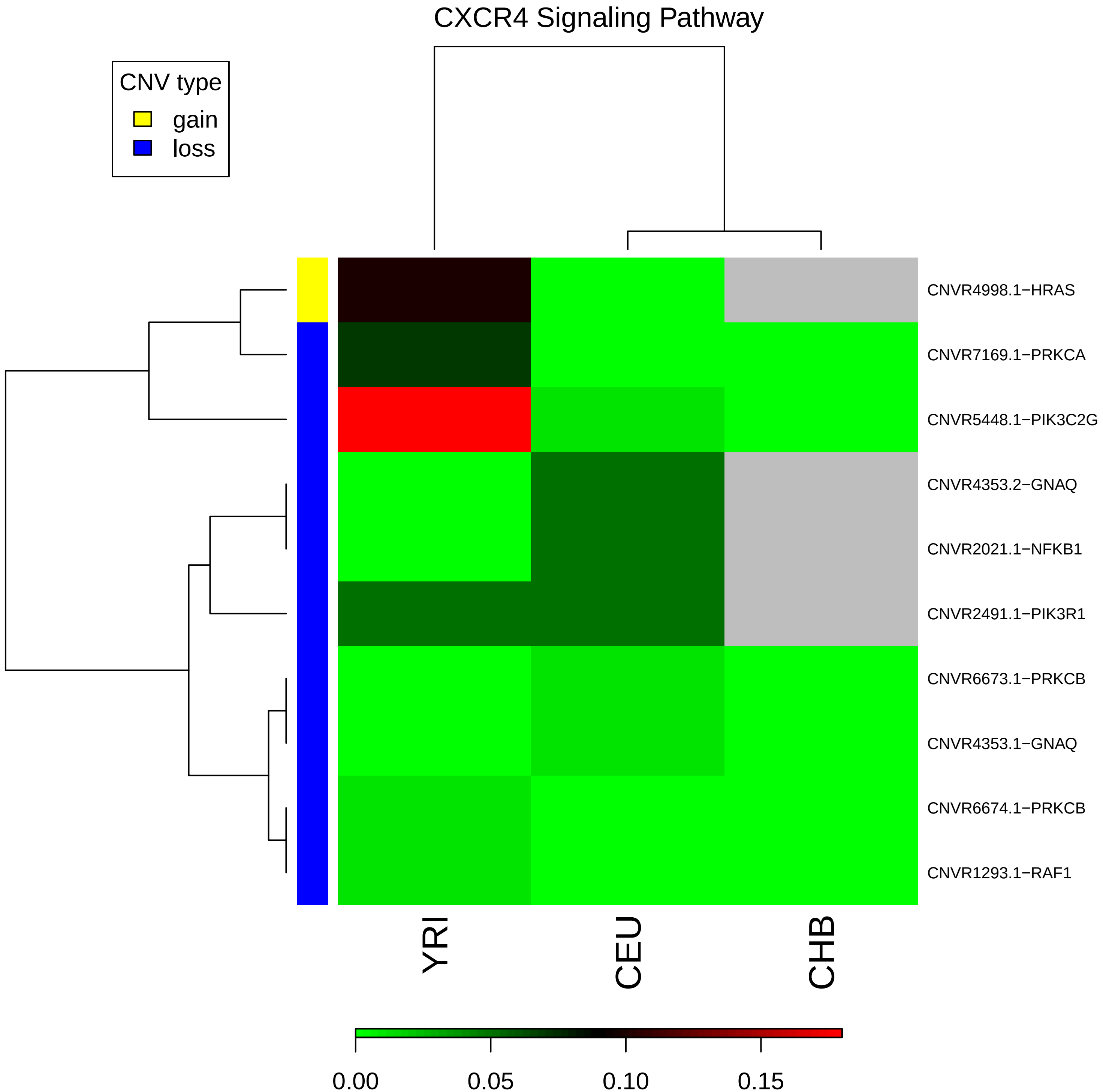
CXCR4 Signaling Pathway
CNV type
gain
loss
CNVR4998.1−HRAS
CNVR7169.1−PRKCA
CNVR5448.1−PIK3C2G
CNVR4353.2−GNAQ
CNVR2021.1−NFKB1
CNVR2491.1−PIK3R1
CNVR6673.1−PRKCB
CNVR4353.1−GNAQ
CNVR6674.1−PRKCB
CNVR1293.1−RAF1
YRI
CEU
CHB
0.00
0.05
0.10
0.15

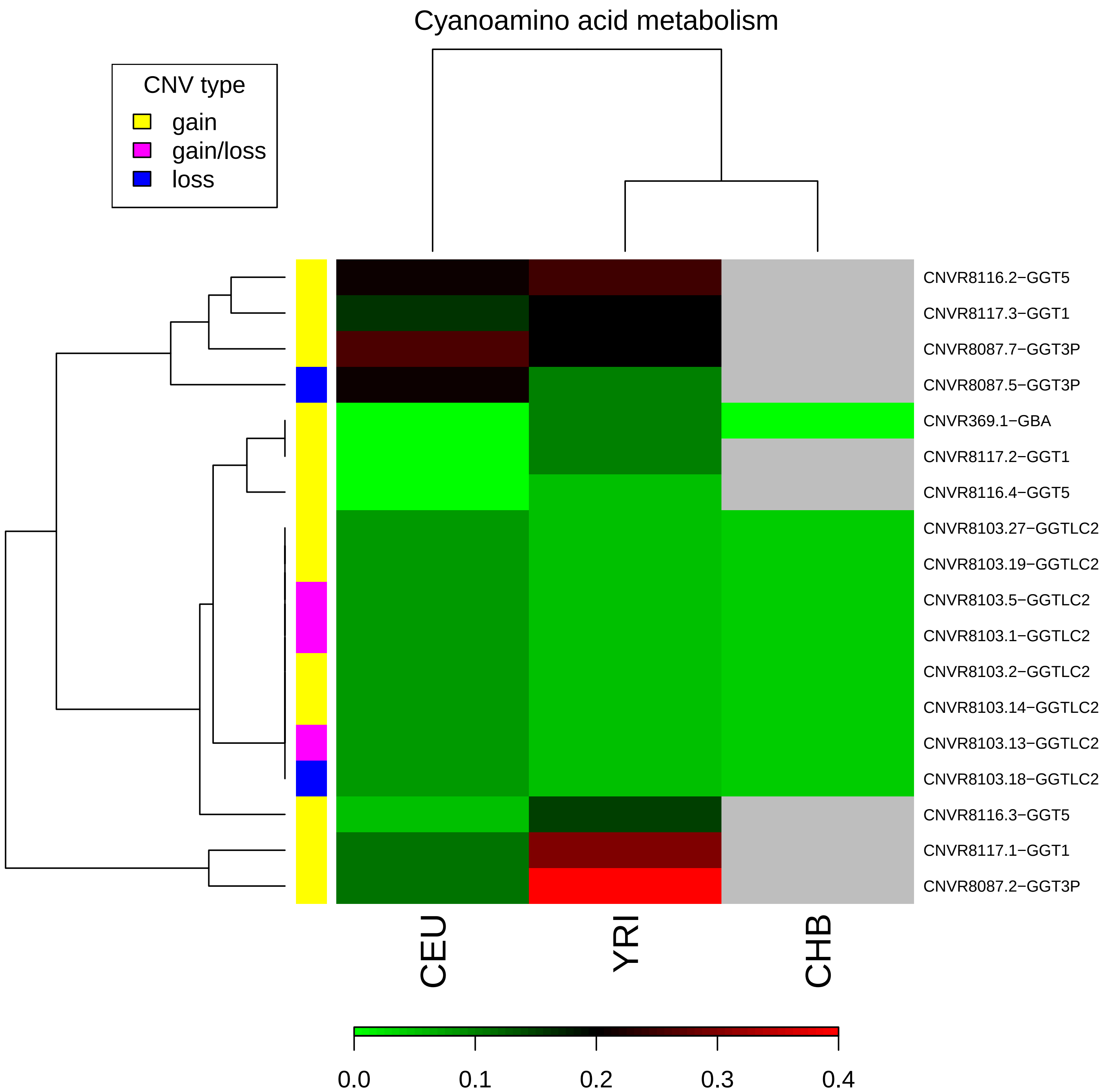
Cyanoamino acid metabolism
CNV type
gain
gain/loss
loss
CNVR8116.2−GGT5
CNVR8117.3−GGT1
CNVR8087.7−GGT3P
CNVR8087.5−GGT3P
CNVR369.1−GBA
CNVR8117.2−GGT1
CNVR8116.4−GGT5
CNVR8103.27−GGTLC2
CNVR8103.19−GGTLC2
CNVR8103.5−GGTLC2
CNVR8103.1−GGTLC2
CNVR8103.2−GGTLC2
CNVR8103.14−GGTLC2
CNVR8103.13−GGTLC2
CNVR8103.18−GGTLC2
CNVR8116.3−GGT5
CNVR8117.1−GGT1
CNVR8087.2−GGT3P
CEU
YRI
CHB
0.0
0.1
0.2
0.3
0.4

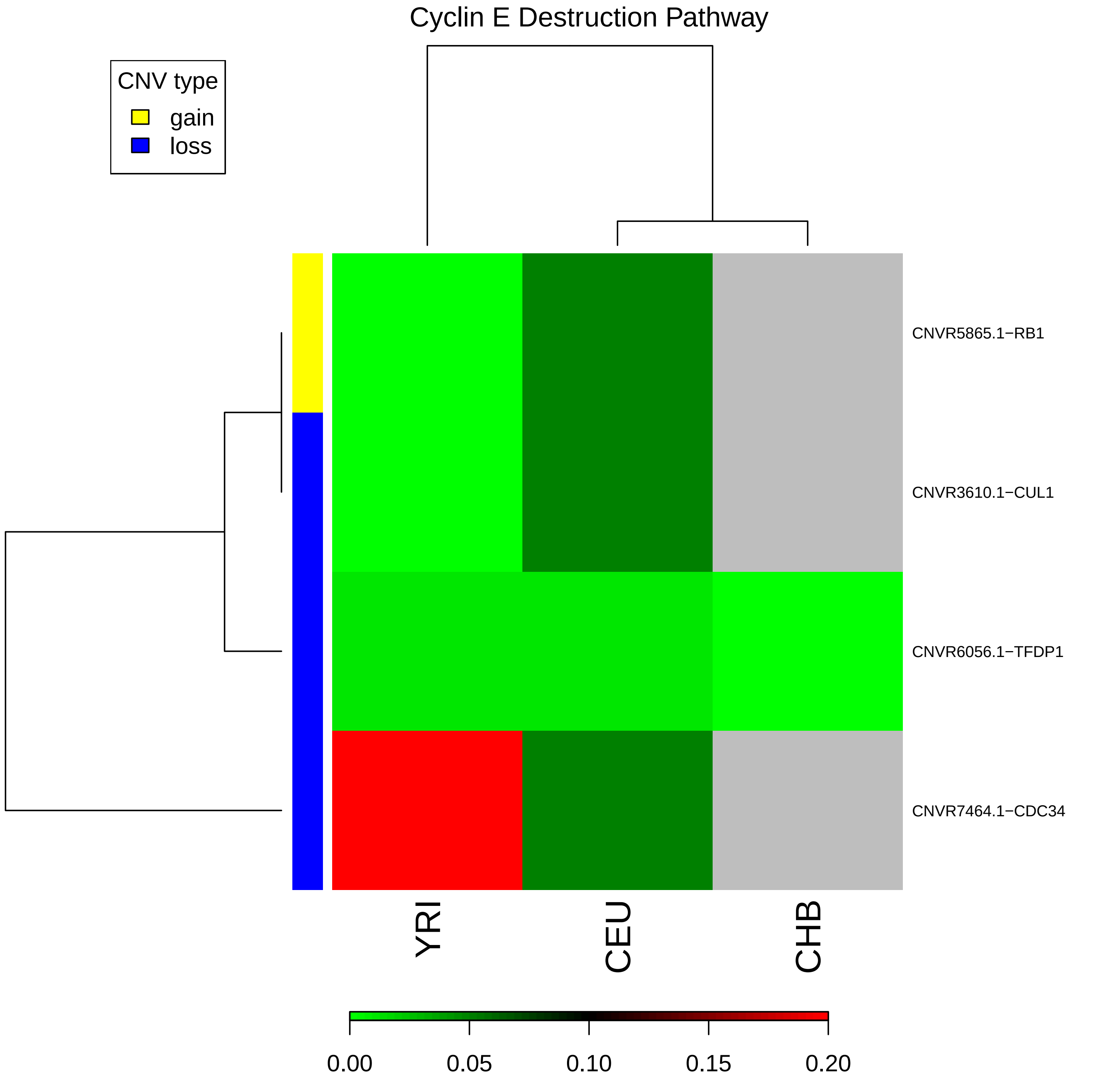

Cyclin E Destruction Pathway
CNV type
gain
loss
CNVR5865.1−RB1
CNVR3610.1−CUL1
CNVR6056.1−TFDP1
CNVR7464.1−CDC34
YRI
CEU
CHB
0.00
0.05
0.10
0.15
0.20

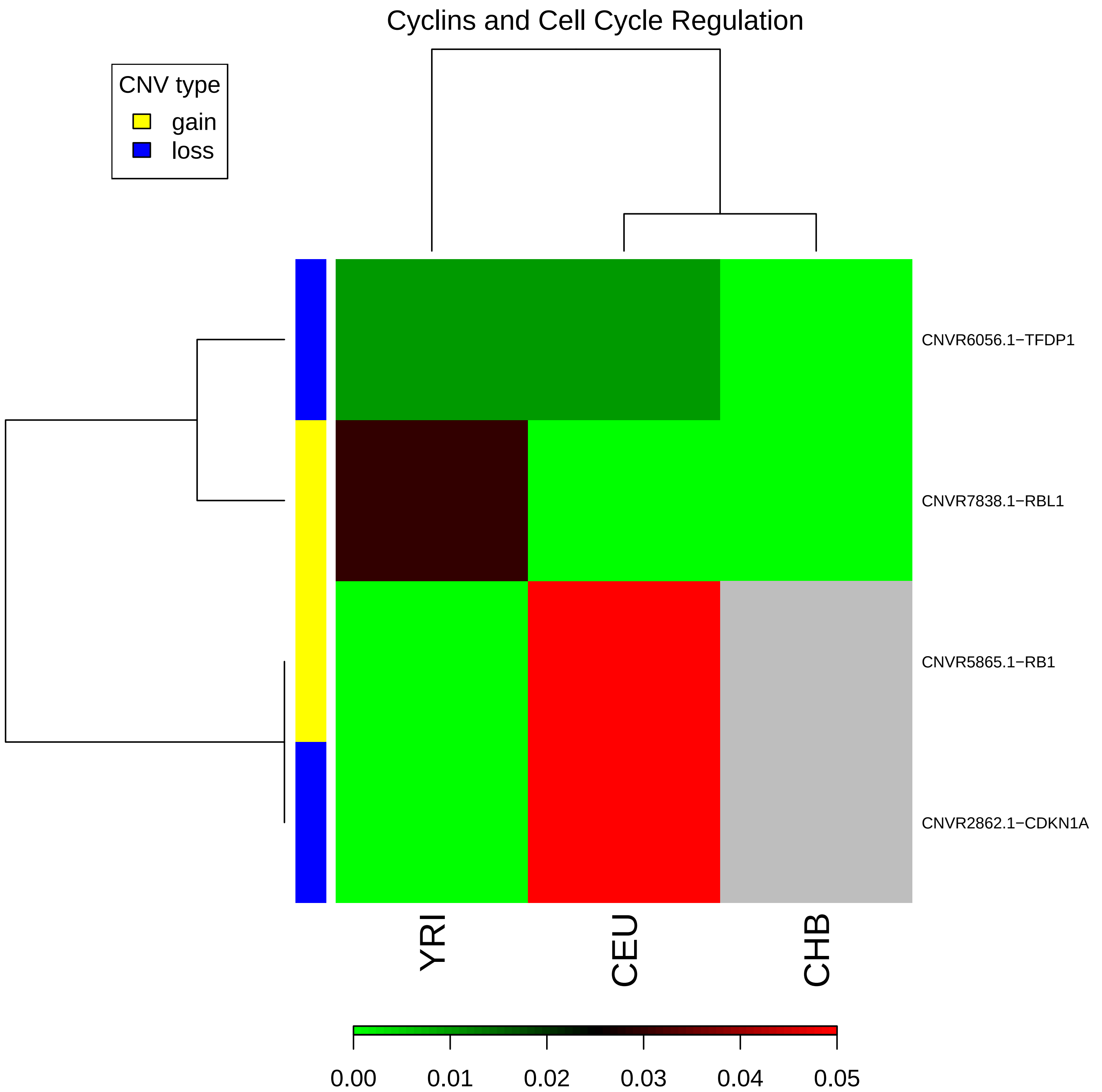

Cyclins and Cell Cycle Regulation
CNV type
gain
loss
CNVR6056.1−TFDP1
CNVR7838.1−RBL1
CNVR5865.1−RB1
CNVR2862.1−CDKN1A
YRI
CEU
CHB
0.00
0.01
0.02
0.03
0.04
0.05

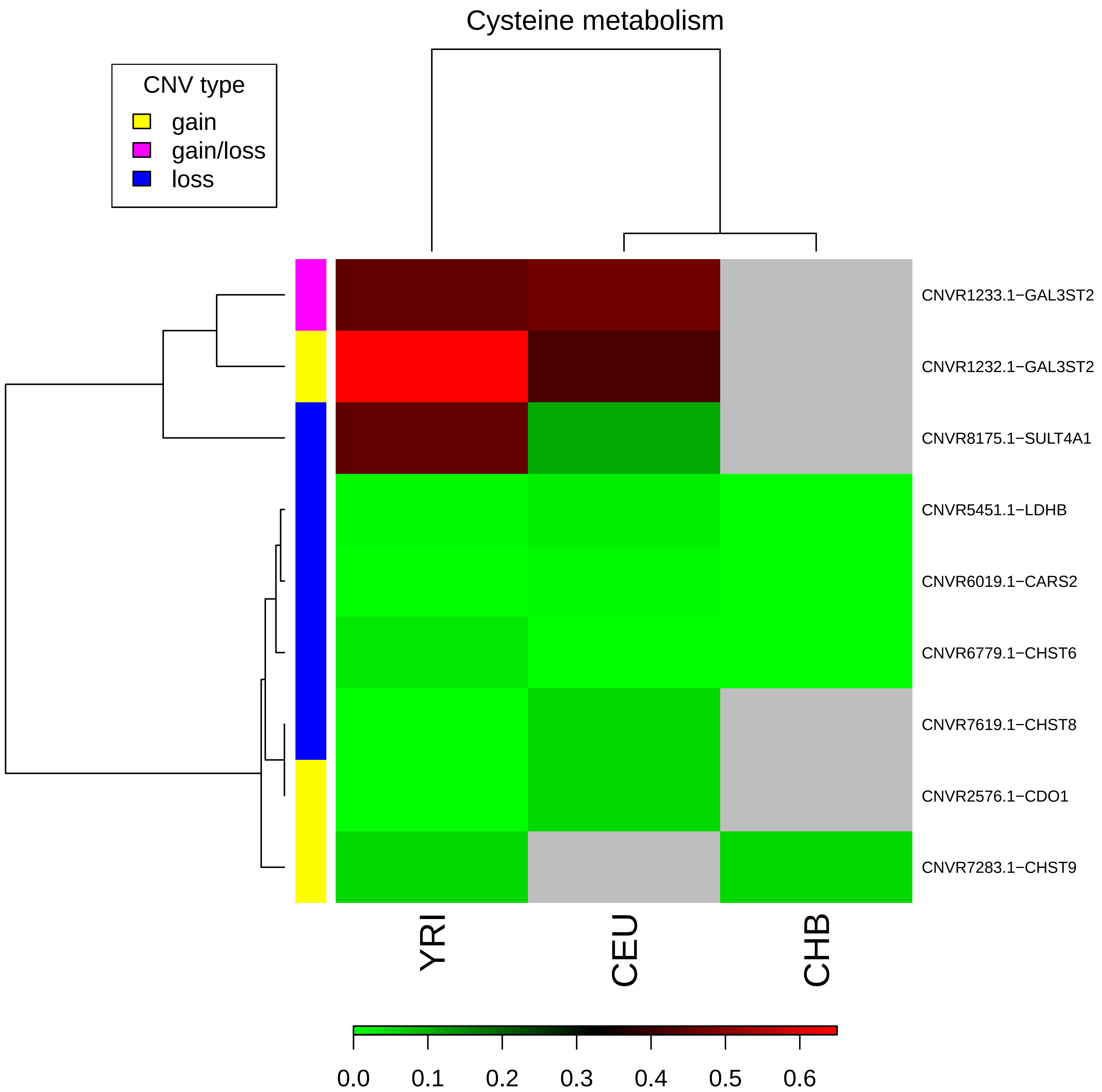
Cysteine metabolism
CNV type
gain
gain/loss
loss
CNVR1233.1−GAL3ST2
CNVR1232.1−GAL3ST2
CNVR8175.1−SULT4A1
CNVR5451.1−LDHB
CNVR6019.1−CARS2
CNVR6779.1−CHST6
CNVR7619.1−CHST8
CNVR2576.1−CDO1
CNVR7283.1−CHST9
YRI
CEU
CHB
0.0
0.1
0.2
0.3
0.4
0.5
0.6

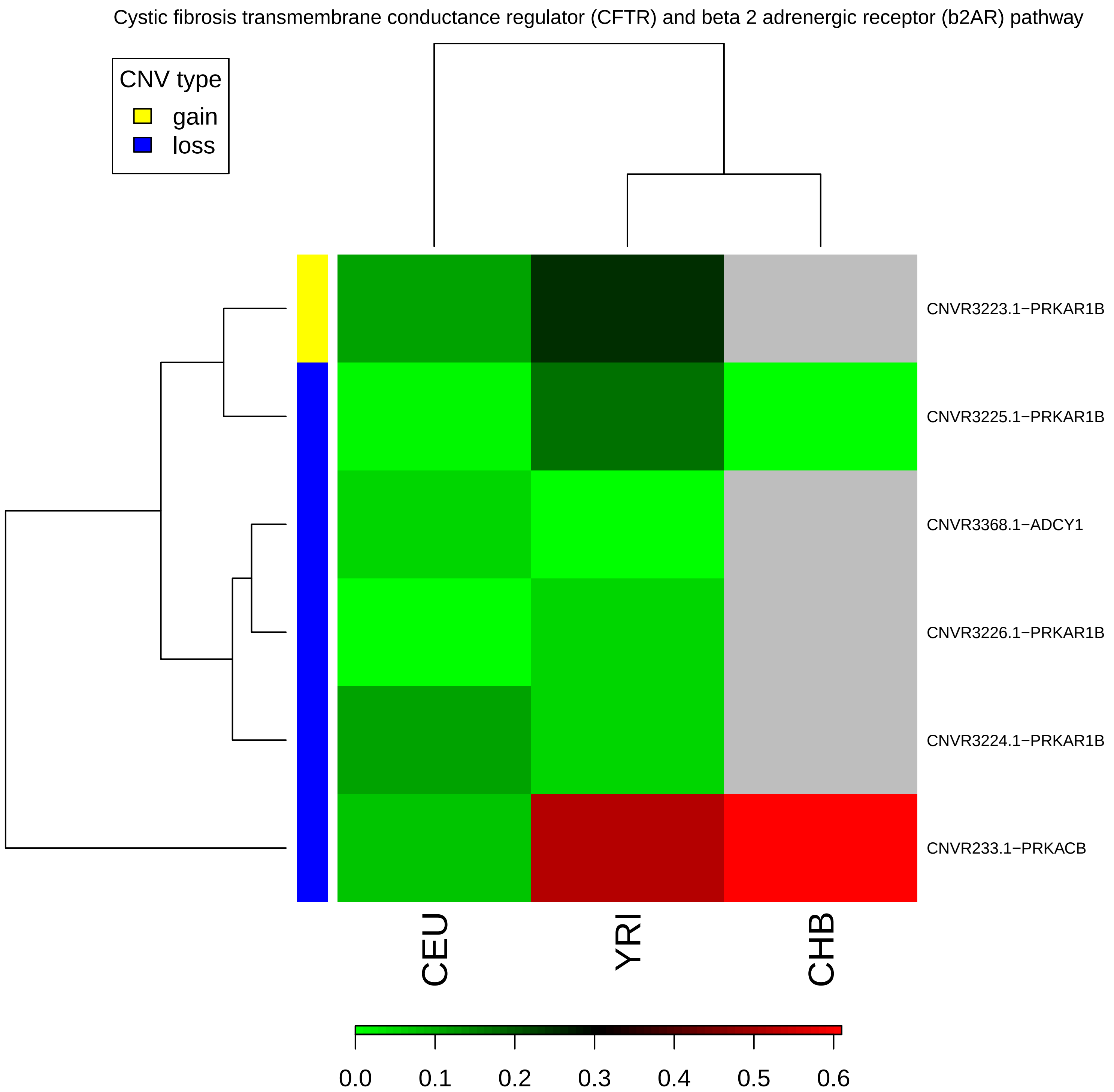

Cystic fibrosis transmembrane conductance regulator (CFTR) and beta 2 adrenergic receptor (b2AR) pathway
CNV type
gain
loss
CNVR3223.1−PRKAR1B
CNVR3225.1−PRKAR1B
CNVR3368.1−ADCY1
CNVR3226.1−PRKAR1B
CNVR3224.1−PRKAR1B
CNVR233.1−PRKACB
CEU
YRI
CHB
0.0
0.1
0.2
0.3
0.4
0.5
0.6

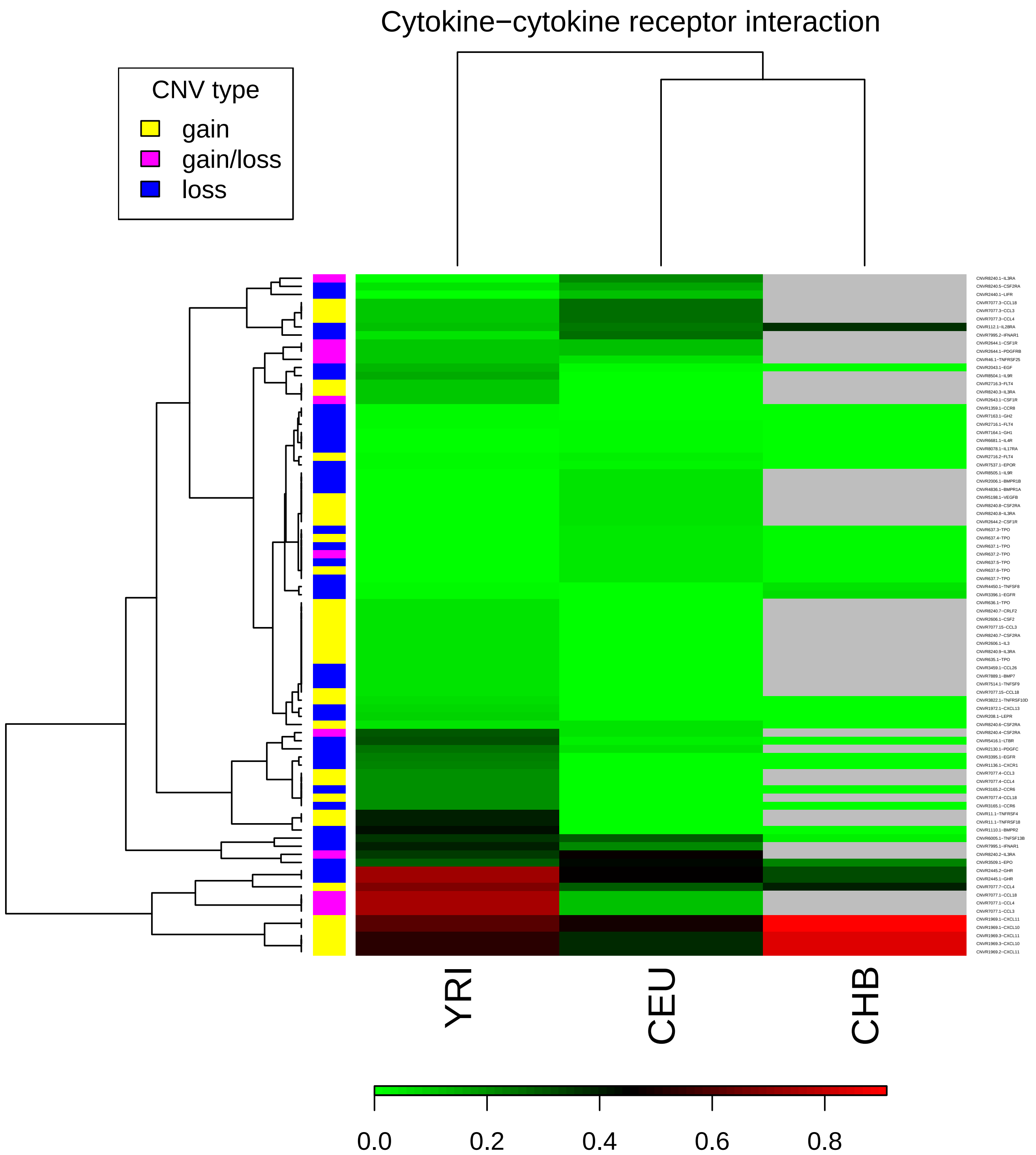
Cytokine−cytokine receptor interaction
CNV type
gain
gain/loss
loss
CNVR8240.1−IL3RA
CNVR8240.5−CSF2RA
CNVR2440.1−LIFR
CNVR7077.3−CCL18
CNVR7077.3−CCL3
CNVR7077.3−CCL4
CNVR112.1−IL28RA
CNVR7995.2−IFNAR1
CNVR2644.1−CSF1R
CNVR2644.1−PDGFRB
CNVR46.1−TNFRSF25
CNVR2043.1−EGF
CNVR8504.1−IL9R
CNVR2716.3−FLT4
CNVR8240.3−IL3RA
CNVR2643.1−CSF1R
CNVR1359.1−CCR8
CNVR7163.1−GH2
CNVR2716.1−FLT4
CNVR7164.1−GH1
CNVR6681.1−IL4R
CNVR8078.1−IL17RA
CNVR2716.2−FLT4
CNVR7537.1−EPOR
CNVR8505.1−IL9R
CNVR2006.1−BMPR1B
CNVR4836.1−BMPR1A
CNVR5198.1−VEGFB
CNVR8240.8−CSF2RA
CNVR8240.8−IL3RA
CNVR2644.2−CSF1R
CNVR637.3−TPO
CNVR637.4−TPO
CNVR637.1−TPO
CNVR637.2−TPO
CNVR637.5−TPO
CNVR637.6−TPO
CNVR637.7−TPO
CNVR4450.1−TNFSF8
CNVR3396.1−EGFR
CNVR636.1−TPO
CNVR8240.7−CRLF2
CNVR2606.1−CSF2
CNVR7077.15−CCL3
CNVR8240.7−CSF2RA
CNVR2606.1−IL3
CNVR8240.9−IL3RA
CNVR635.1−TPO
CNVR3459.1−CCL26
CNVR7889.1−BMP7
CNVR7514.1−TNFSF9
CNVR7077.15−CCL18
CNVR3822.1−TNFRSF10D
CNVR1972.1−CXCL13
CNVR208.1−LEPR
CNVR8240.6−CSF2RA
CNVR8240.4−CSF2RA
CNVR5416.1−LTBR
CNVR2130.1−PDGFC
CNVR3395.1−EGFR
CNVR1136.1−CXCR1
CNVR7077.4−CCL3
CNVR7077.4−CCL4
CNVR3165.2−CCR6
CNVR7077.4−CCL18
CNVR3165.1−CCR6
CNVR11.1−TNFRSF4
CNVR11.1−TNFRSF18
CNVR1110.1−BMPR2
CNVR6005.1−TNFSF13B
CNVR7995.1−IFNAR1
CNVR8240.2−IL3RA
CNVR3509.1−EPO
CNVR2445.2−GHR
CNVR2445.1−GHR
CNVR7077.7−CCL4
CNVR7077.1−CCL18
CNVR7077.1−CCL4
CNVR7077.1−CCL3
CNVR1969.1−CXCL11
CNVR1969.1−CXCL10
CNVR1969.3−CXCL11
CNVR1969.3−CXCL10
CNVR1969.2−CXCL11
YRI
CEU
CHB
0.0
0.2
0.4
0.6
0.8

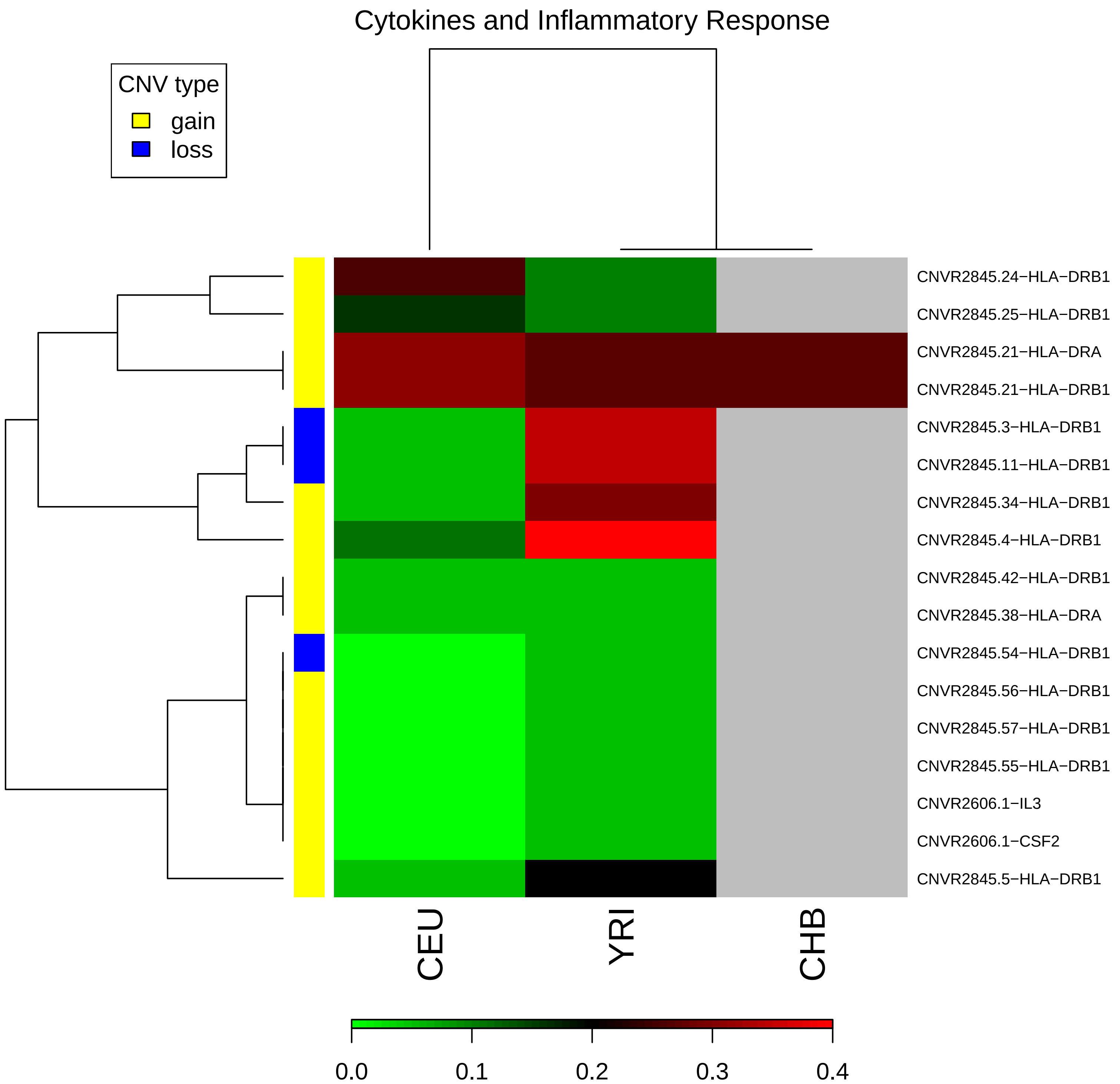

Cytokines and Inflammatory Response
CNV type
gain
loss
CNVR2845.24−HLA−DRB1
CNVR2845.25−HLA−DRB1
CNVR2845.21−HLA−DRA
CNVR2845.21−HLA−DRB1
CNVR2845.3−HLA−DRB1
CNVR2845.11−HLA−DRB1
CNVR2845.34−HLA−DRB1
CNVR2845.4−HLA−DRB1
CNVR2845.42−HLA−DRB1
CNVR2845.38−HLA−DRA
CNVR2845.54−HLA−DRB1
CNVR2845.56−HLA−DRB1
CNVR2845.57−HLA−DRB1
CNVR2845.55−HLA−DRB1
CNVR2606.1−IL3
CNVR2606.1−CSF2
CNVR2845.5−HLA−DRB1
CEU
YRI
CHB
0.0
0.1
0.2
0.3
0.4

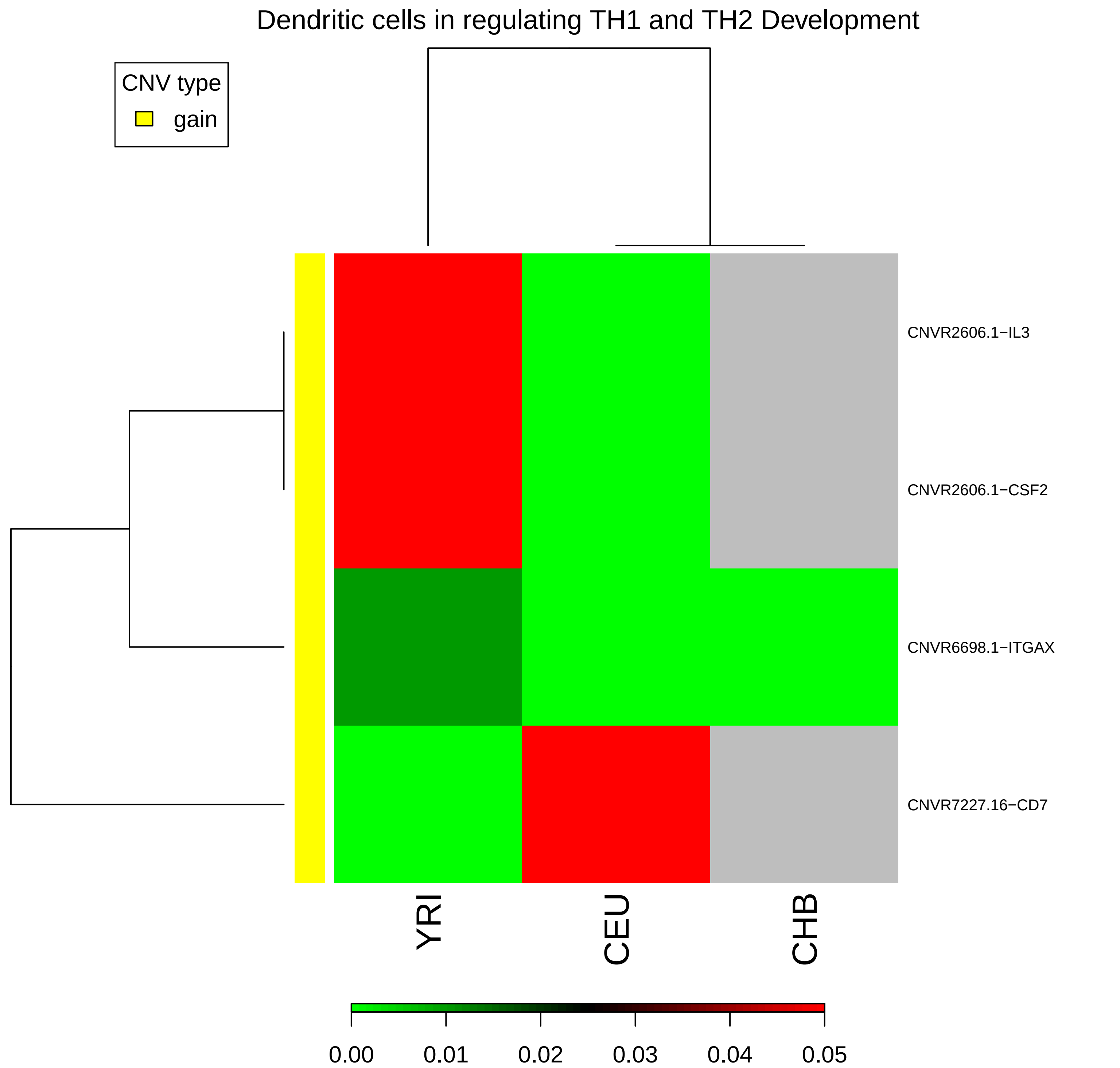

Dendritic cells in regulating TH1 and TH2 Development
CNV type
gain
CNVR2606.1−IL3
CNVR2606.1−CSF2
CNVR6698.1−ITGAX
CNVR7227.16−CD7
YRI
CEU
CHB
0.00
0.01
0.02
0.03
0.04
0.05

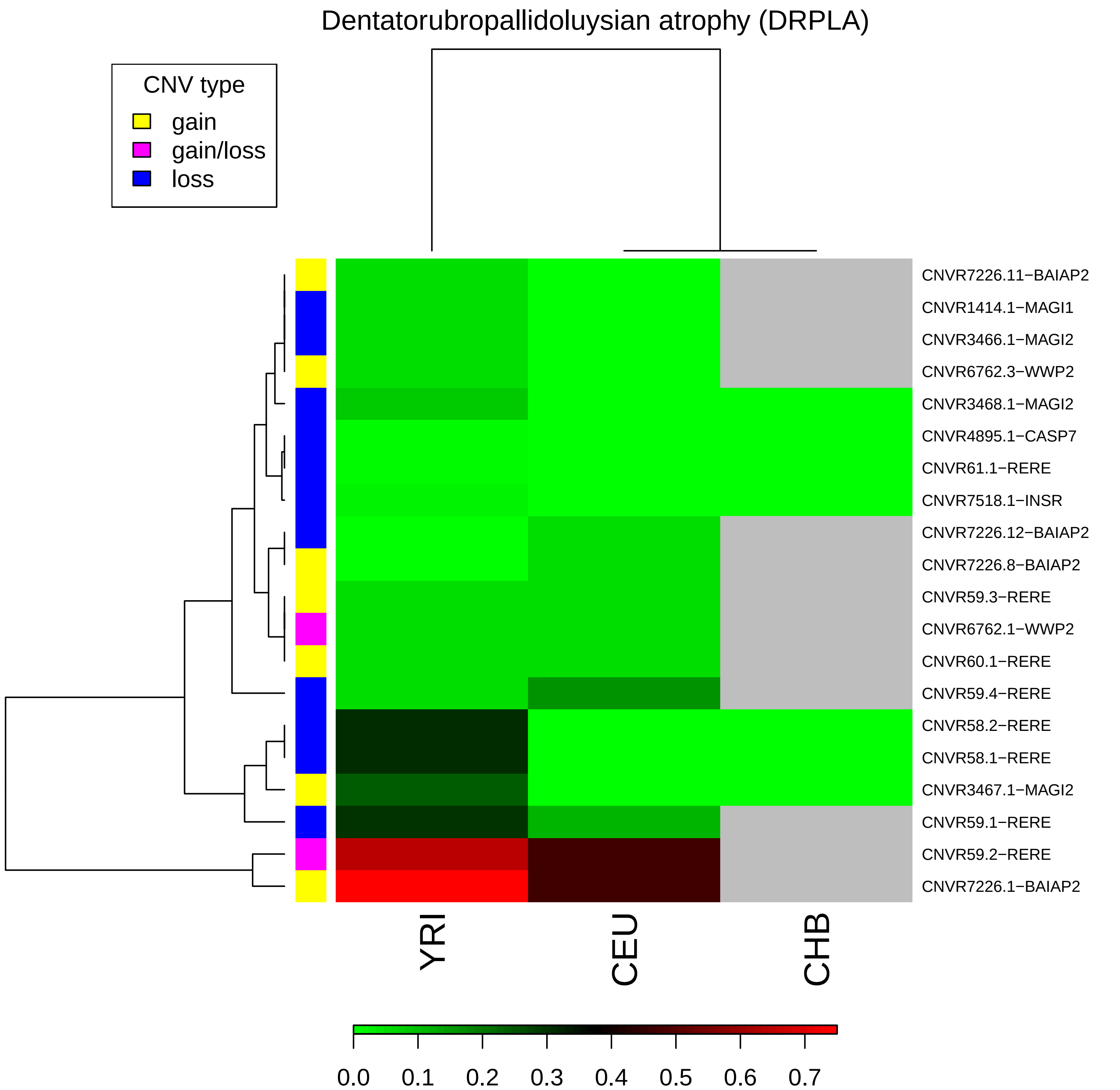
Dentatorubropallidoluysian atrophy (DRPLA)
CNV type
gain
gain/loss
loss
CNVR7226.11−BAIAP2
CNVR1414.1−MAGI1
CNVR3466.1−MAGI2
CNVR6762.3−WWP2
CNVR3468.1−MAGI2
CNVR4895.1−CASP7
CNVR61.1−RERE
CNVR7518.1−INSR
CNVR7226.12−BAIAP2
CNVR7226.8−BAIAP2
CNVR59.3−RERE
CNVR6762.1−WWP2
CNVR60.1−RERE
CNVR59.4−RERE
CNVR58.2−RERE
CNVR58.1−RERE
CNVR3467.1−MAGI2
CNVR59.1−RERE
CNVR59.2−RERE
CNVR7226.1−BAIAP2
YRI
CEU
CHB
0.0
0.1
0.2
0.3
0.4
0.5
0.6
0.7

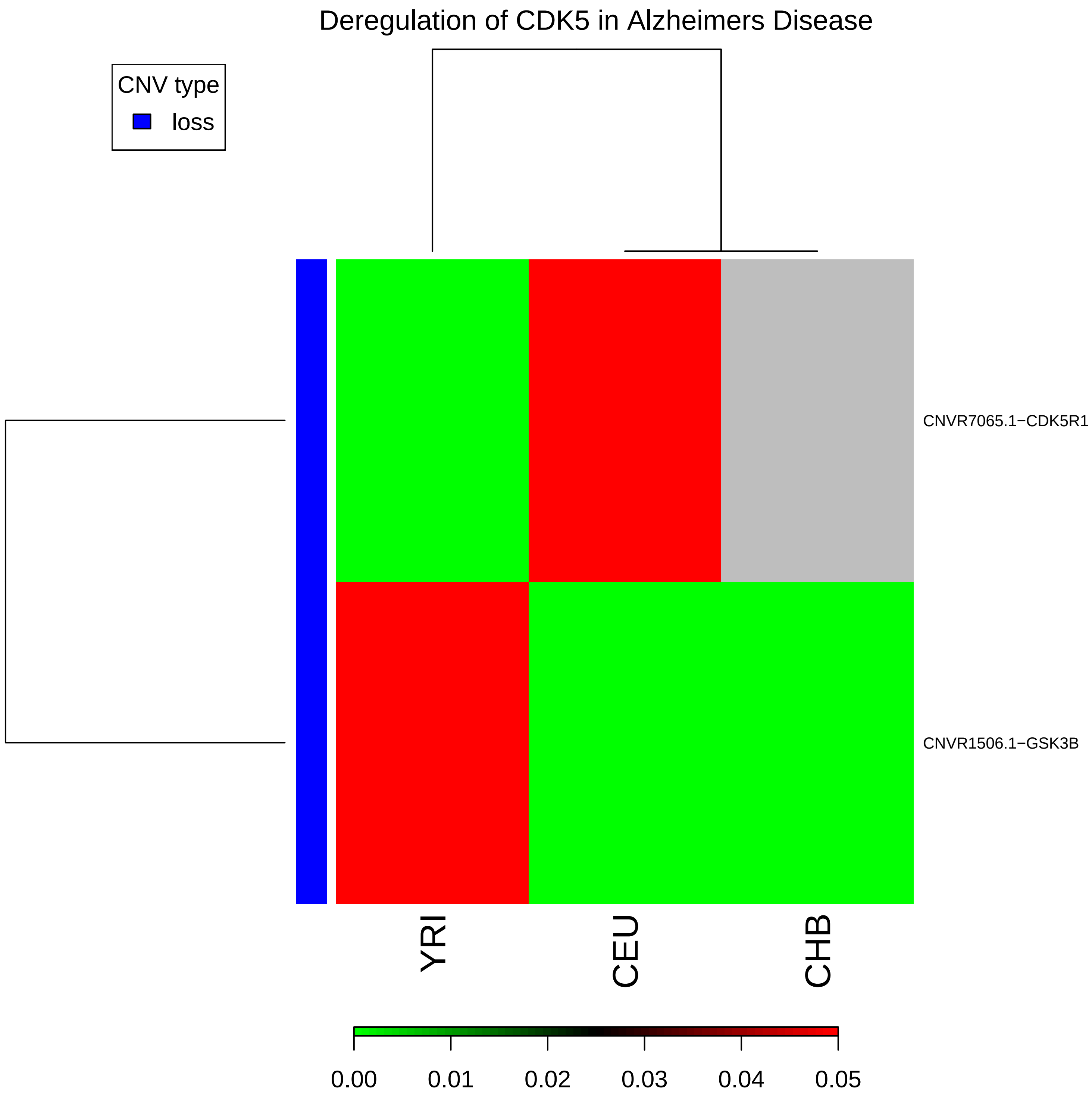
Deregulation of CDK5 in Alzheimers Disease
CNV type
loss
CNVR7065.1−CDK5R1
CNVR1506.1−GSK3B
YRI
CEU
CHB
0.00
0.01
0.02
0.03
0.04
0.05

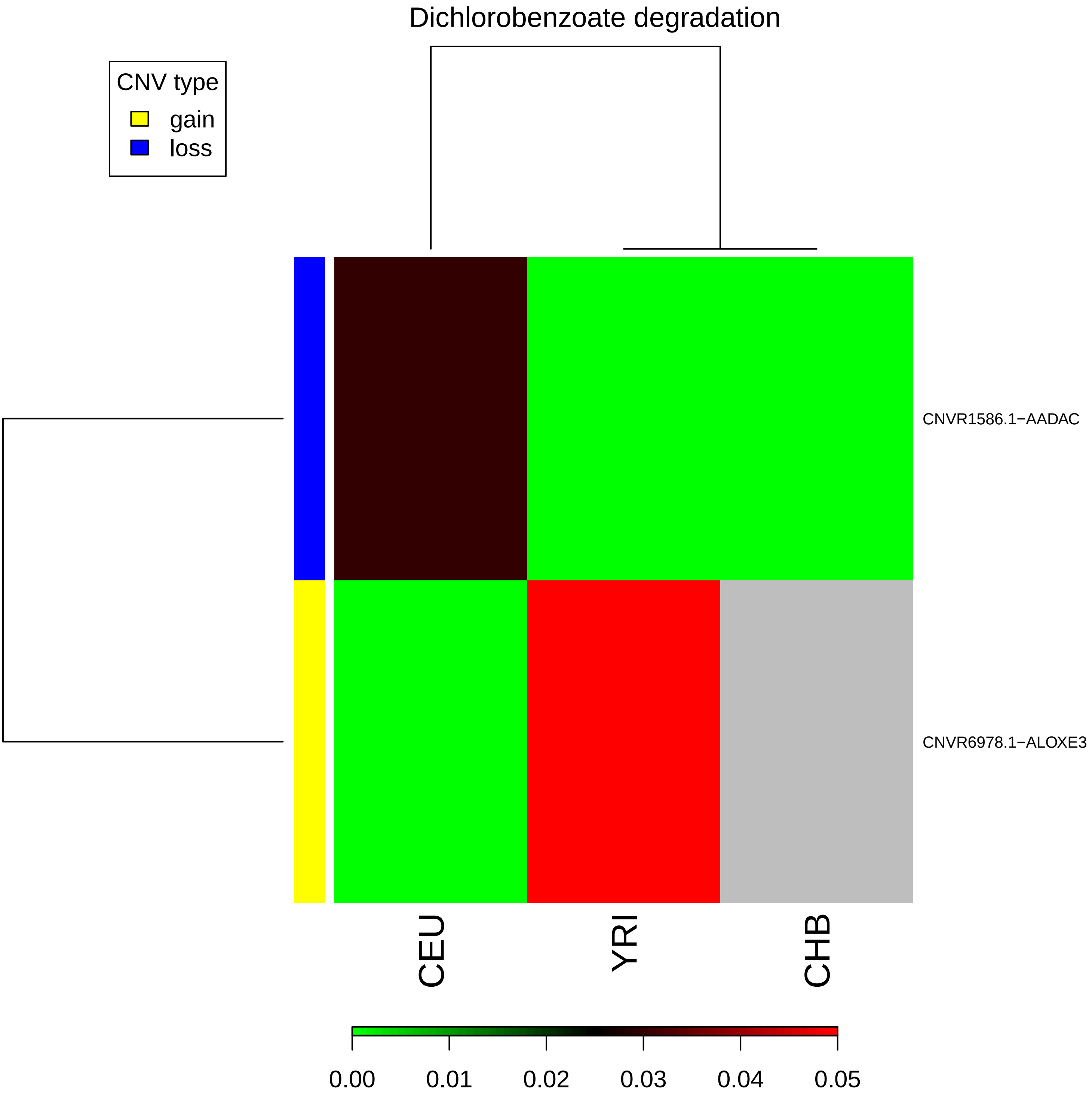
Dichlorobenzoate degradation
CNV type
gain
loss
CNVR1586.1−AADAC
CNVR6978.1−ALOXE3
CEU
YRI
CHB
0.00
0.01
0.02
0.03
0.04
0.05

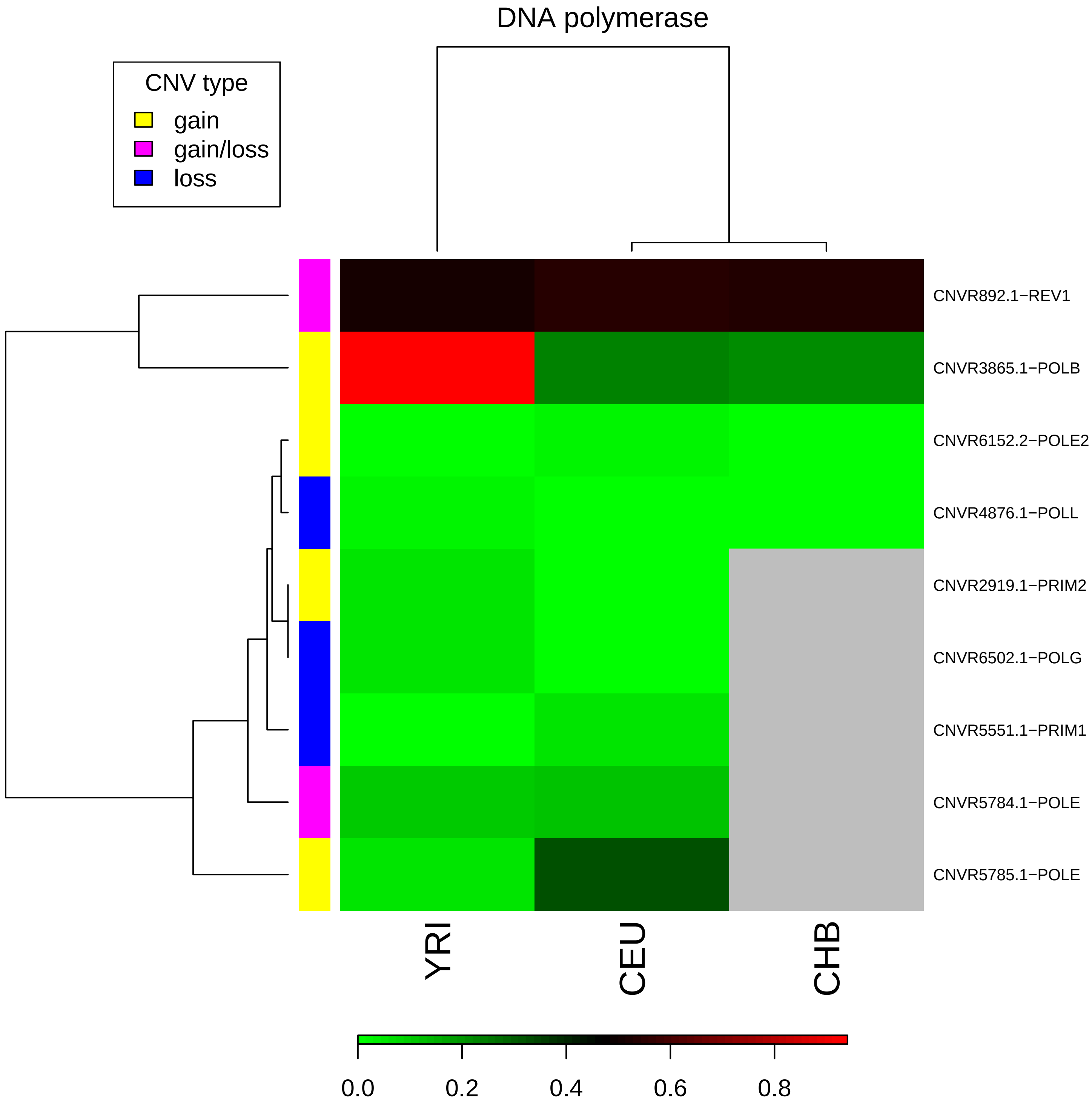

DNA polymerase
CNV type
gain
gain/loss
loss
CNVR892.1−REV1
CNVR3865.1−POLB
CNVR6152.2−POLE2
CNVR4876.1−POLL
CNVR2919.1−PRIM2
CNVR6502.1−POLG
CNVR5551.1−PRIM1
CNVR5784.1−POLE
CNVR5785.1−POLE
YRI
CEU
CHB
0.0
0.2
0.4
0.6
0.8

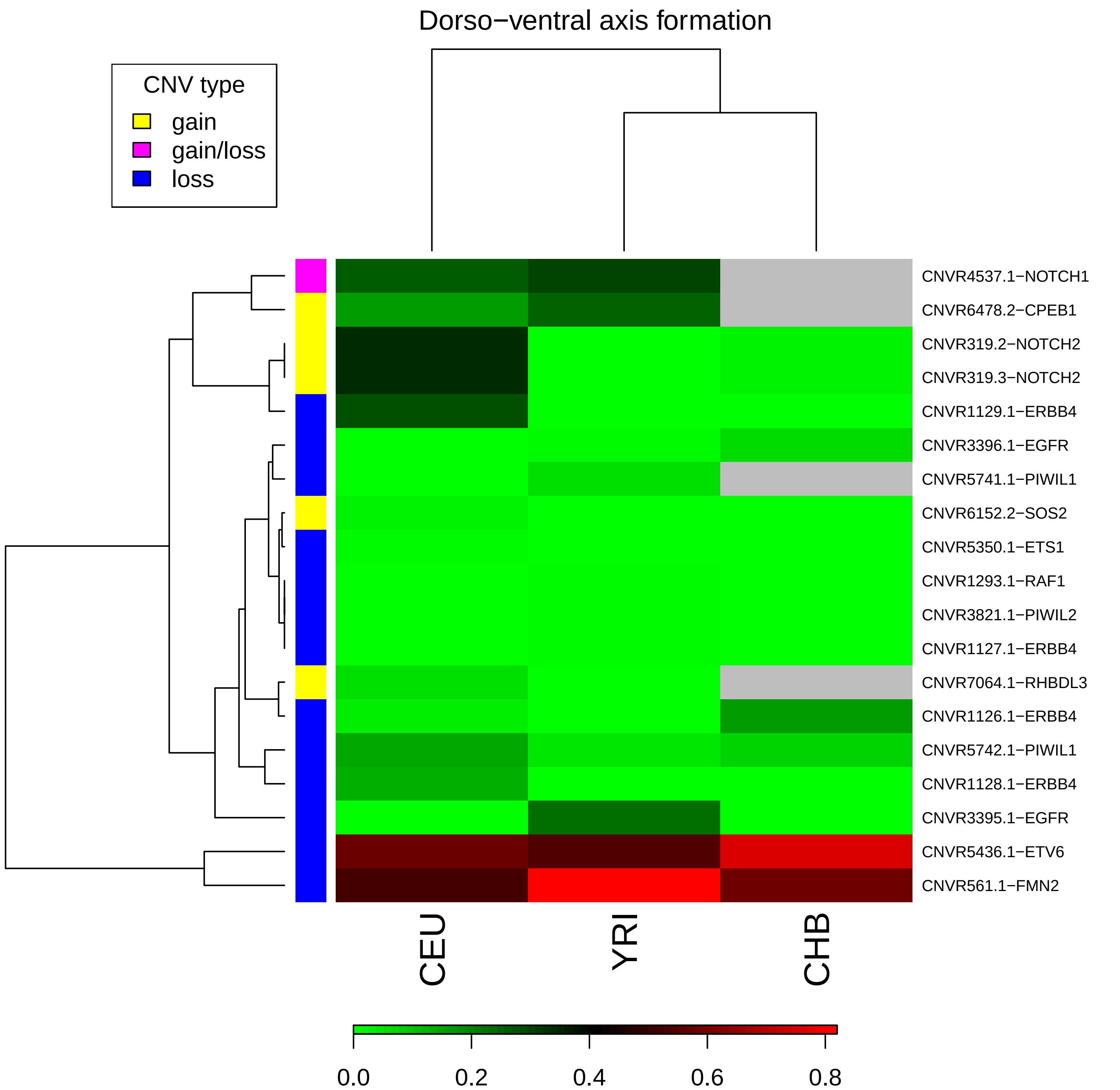

Dorso−ventral axis formation
CNV type
gain
gain/loss
loss
CNVR4537.1−NOTCH1
CNVR6478.2−CPEB1
CNVR319.2−NOTCH2
CNVR319.3−NOTCH2
CNVR1129.1−ERBB4
CNVR3396.1−EGFR
CNVR5741.1−PIWIL1
CNVR6152.2−SOS2
CNVR5350.1−ETS1
CNVR1293.1−RAF1
CNVR3821.1−PIWIL2
CNVR1127.1−ERBB4
CNVR7064.1−RHBDL3
CNVR1126.1−ERBB4
CNVR5742.1−PIWIL1
CNVR1128.1−ERBB4
CNVR3395.1−EGFR
CNVR5436.1−ETV6
CNVR561.1−FMN2
CEU
YRI
CHB
0.0
0.2
0.4
0.6
0.8

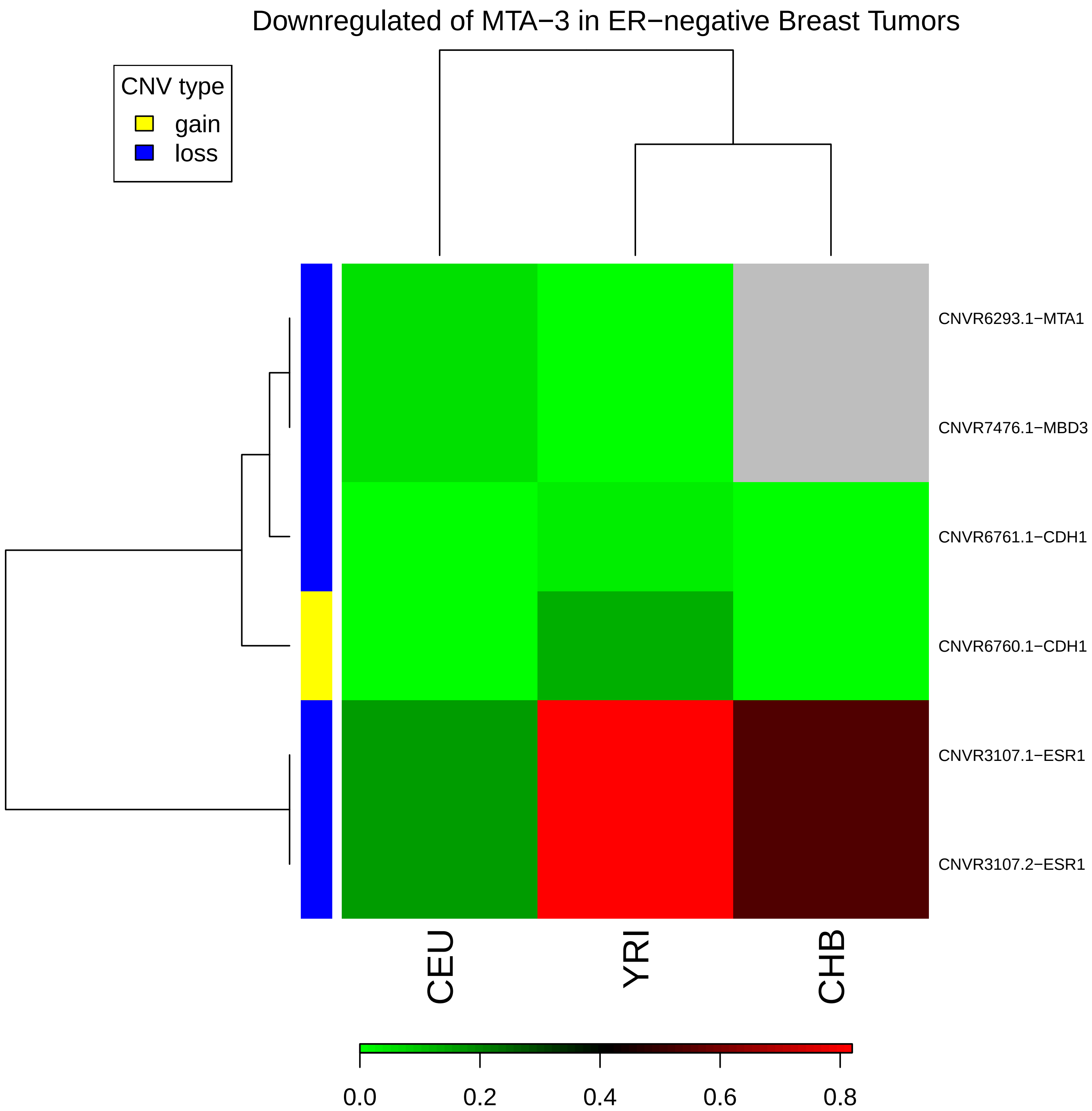

Downregulated of MTA−3 in ER−negative Breast Tumors
CNV type
gain
loss
CNVR6293.1−MTA1
CNVR7476.1−MBD3
CNVR6761.1−CDH1
CNVR6760.1−CDH1
CNVR3107.1−ESR1
CNVR3107.2−ESR1
CEU
YRI
CHB
0.0
0.2
0.4
0.6
0.8

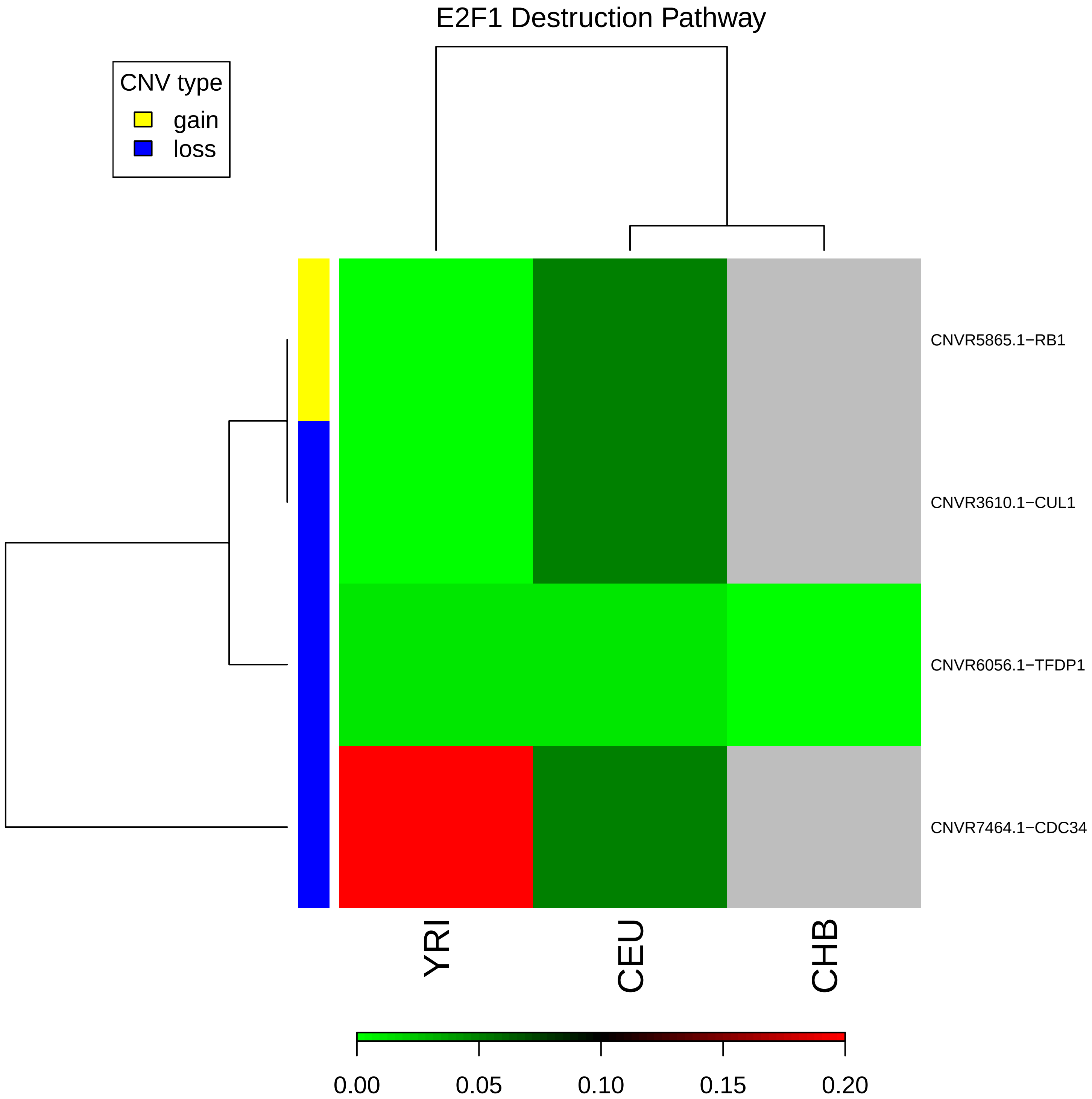
E2F1 Destruction Pathway
CNV type
gain
loss
CNVR5865.1−RB1
CNVR3610.1−CUL1
CNVR6056.1−TFDP1
CNVR7464.1−CDC34
YRI
CEU
CHB
0.00
0.05
0.10
0.15
0.20

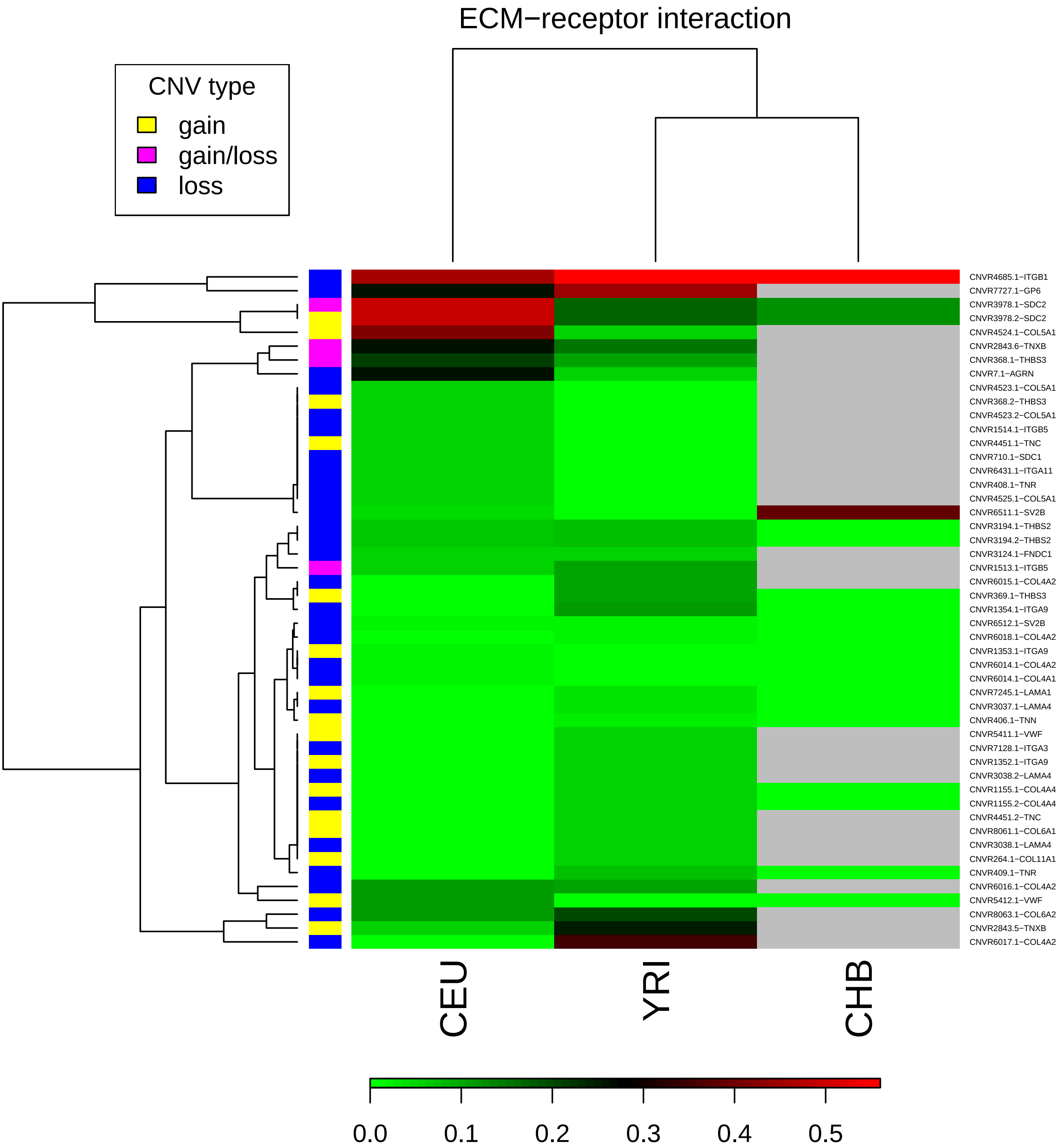
ECM−receptor interaction
CNV type
gain
gain/loss
loss
CNVR4685.1−ITGB1
CNVR7727.1−GP6
CNVR3978.1−SDC2
CNVR3978.2−SDC2
CNVR4524.1−COL5A1
CNVR2843.6−TNXB
CNVR368.1−THBS3
CNVR7.1−AGRN
CNVR4523.1−COL5A1
CNVR368.2−THBS3
CNVR4523.2−COL5A1
CNVR1514.1−ITGB5
CNVR4451.1−TNC
CNVR710.1−SDC1
CNVR6431.1−ITGA11
CNVR408.1−TNR
CNVR4525.1−COL5A1
CNVR6511.1−SV2B
CNVR3194.1−THBS2
CNVR3194.2−THBS2
CNVR3124.1−FNDC1
CNVR1513.1−ITGB5
CNVR6015.1−COL4A2
CNVR369.1−THBS3
CNVR1354.1−ITGA9
CNVR6512.1−SV2B
CNVR6018.1−COL4A2
CNVR1353.1−ITGA9
CNVR6014.1−COL4A2
CNVR6014.1−COL4A1
CNVR7245.1−LAMA1
CNVR3037.1−LAMA4
CNVR406.1−TNN
CNVR5411.1−VWF
CNVR7128.1−ITGA3
CNVR1352.1−ITGA9
CNVR3038.2−LAMA4
CNVR1155.1−COL4A4
CNVR1155.2−COL4A4
CNVR4451.2−TNC
CNVR8061.1−COL6A1
CNVR3038.1−LAMA4
CNVR264.1−COL11A1
CNVR409.1−TNR
CNVR6016.1−COL4A2
CNVR5412.1−VWF
CNVR8063.1−COL6A2
CNVR2843.5−TNXB
CNVR6017.1−COL4A2
CEU
YRI
CHB
0.0
0.1
0.2
0.3
0.4
0.5

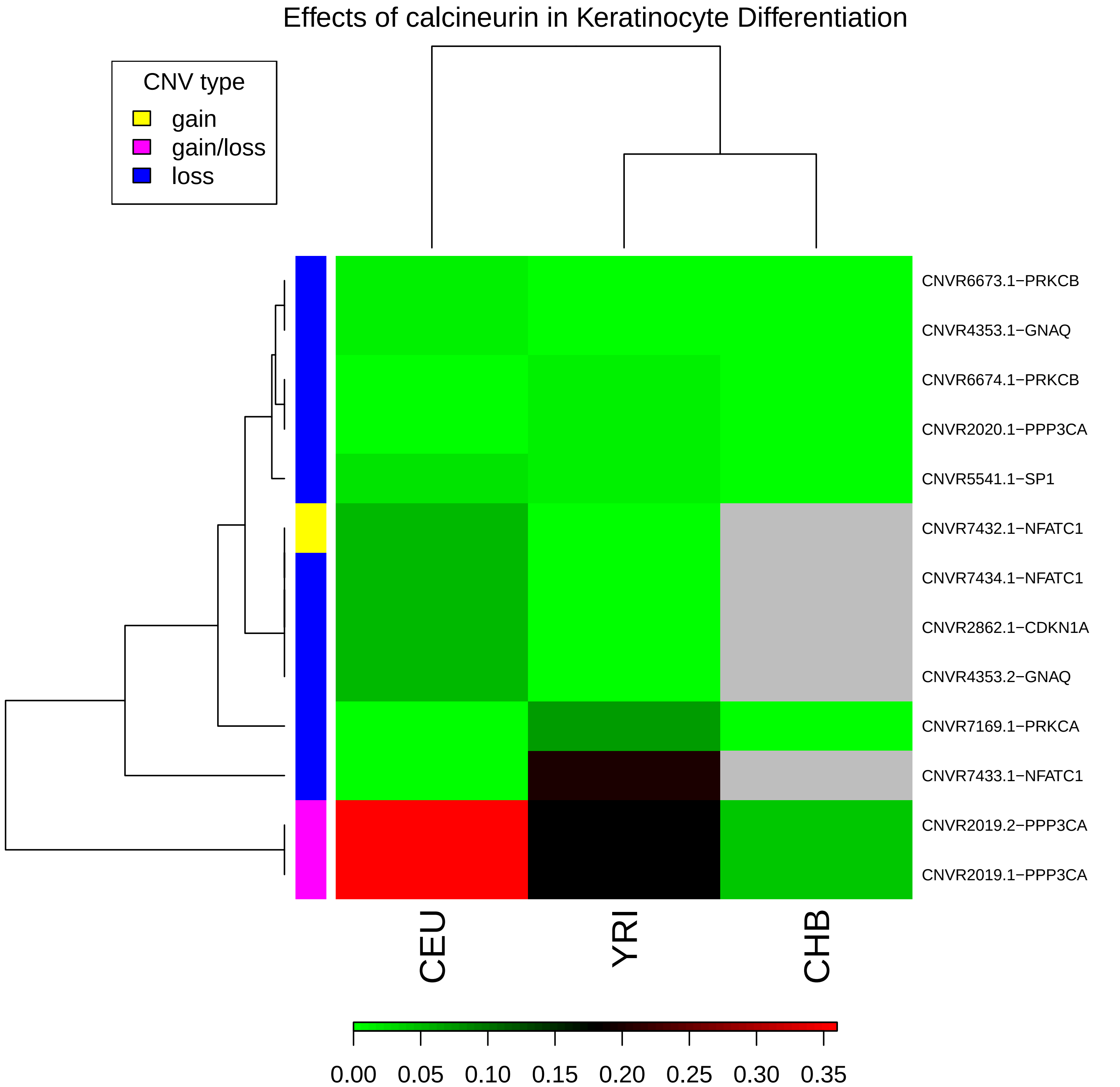
Effects of calcineurin in Keratinocyte Differentiation
CNV type
gain
gain/loss
loss
CNVR6673.1−PRKCB
CNVR4353.1−GNAQ
CNVR6674.1−PRKCB
CNVR2020.1−PPP3CA
CNVR5541.1−SP1
CNVR7432.1−NFATC1
CNVR7434.1−NFATC1
CNVR2862.1−CDKN1A
CNVR4353.2−GNAQ
CNVR7169.1−PRKCA
CNVR7433.1−NFATC1
CNVR2019.2−PPP3CA
CNVR2019.1−PPP3CA
CEU
YRI
CHB
0.00
0.05
0.10
0.15
0.20
0.25
0.30
0.35

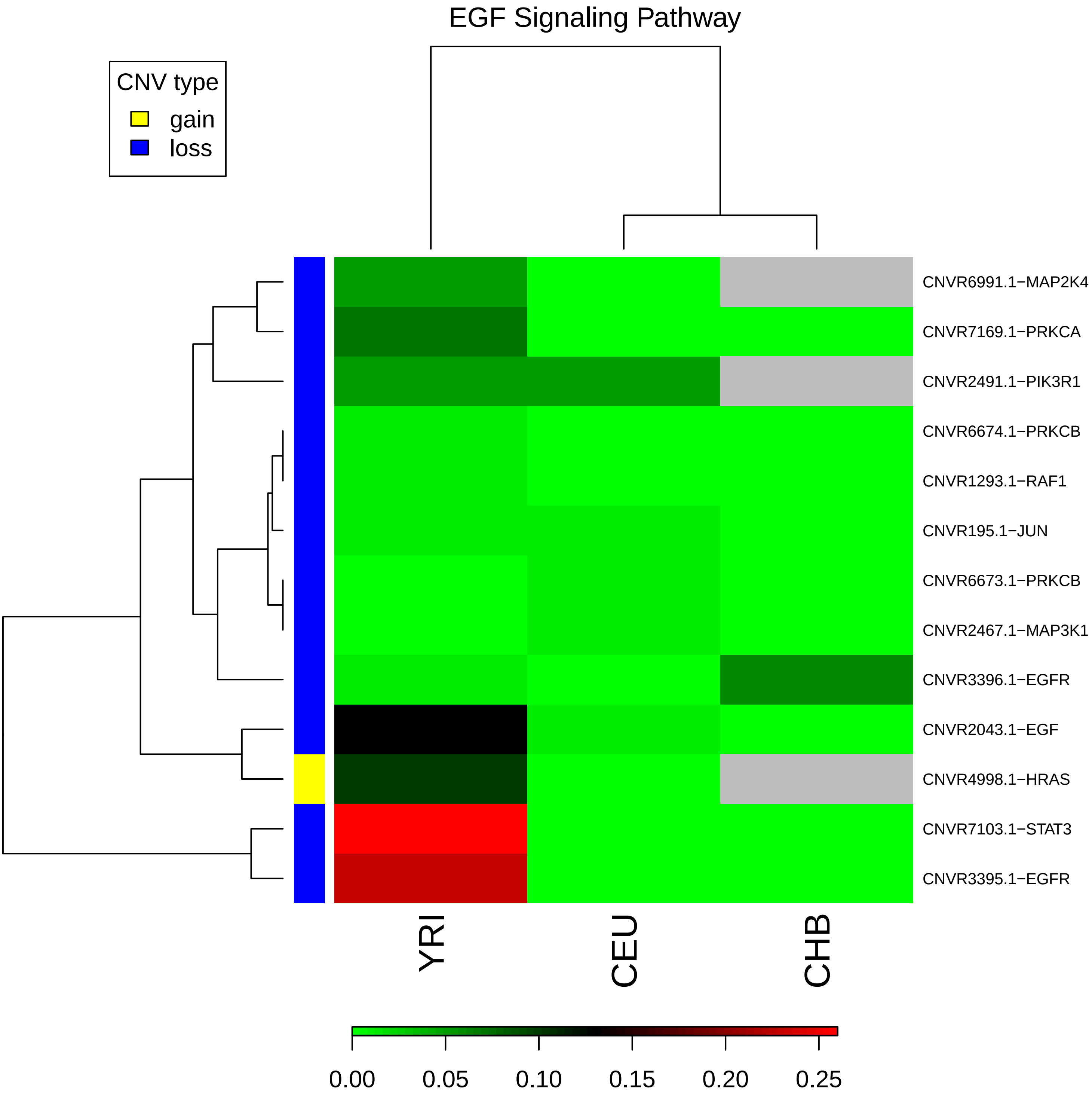
EGF Signaling Pathway
CNV type
gain
loss
CNVR6991.1−MAP2K4
CNVR7169.1−PRKCA
CNVR2491.1−PIK3R1
CNVR6674.1−PRKCB
CNVR1293.1−RAF1
CNVR195.1−JUN
CNVR6673.1−PRKCB
CNVR2467.1−MAP3K1
CNVR3396.1−EGFR
CNVR2043.1−EGF
CNVR4998.1−HRAS
CNVR7103.1−STAT3
CNVR3395.1−EGFR
YRI
CEU
CHB
0.00
0.05
0.10
0.15
0.20
0.25

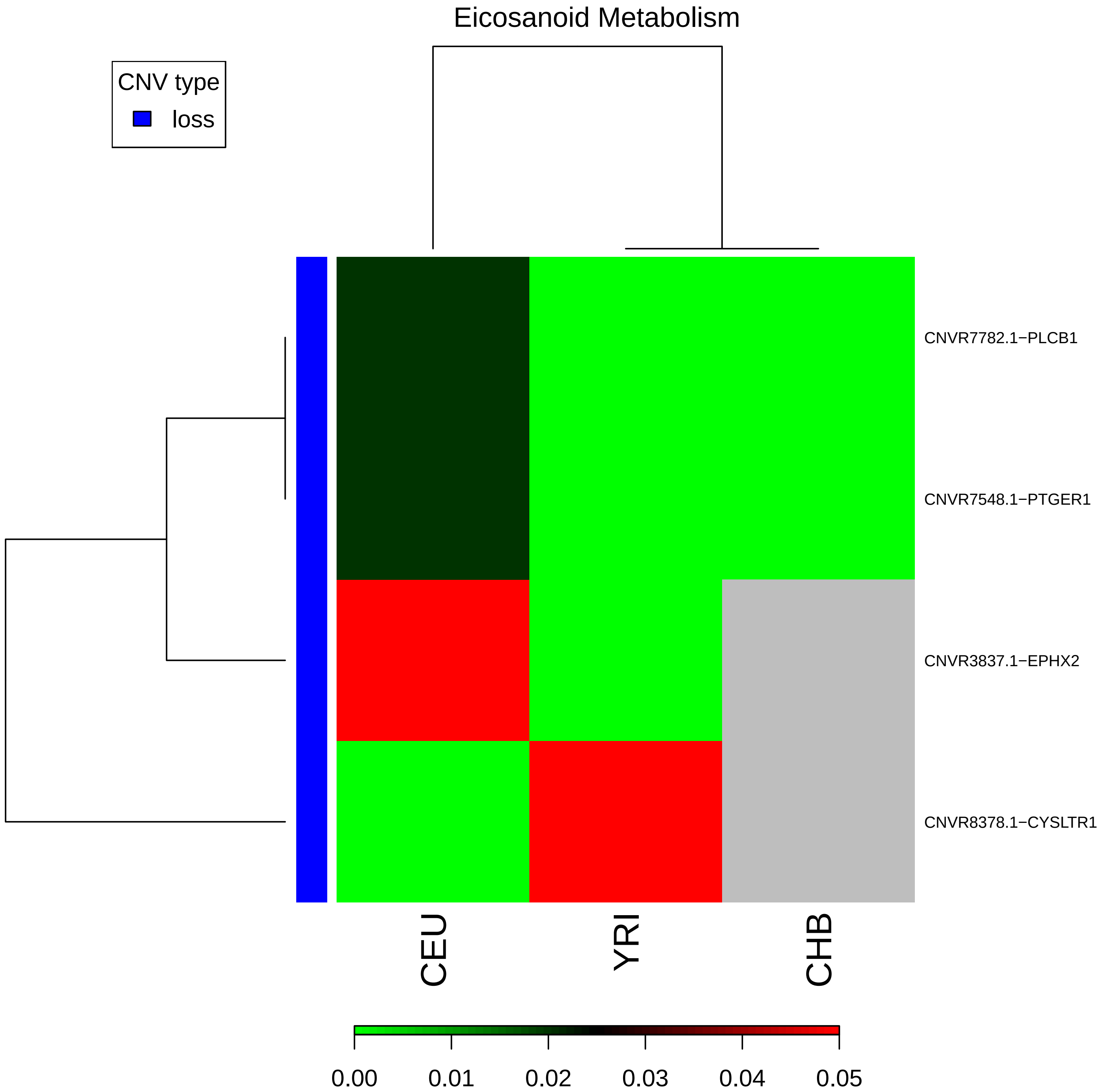
Eicosanoid Metabolism
CNV type
loss
CNVR7782.1−PLCB1
CNVR7548.1−PTGER1
CNVR3837.1−EPHX2
CNVR8378.1−CYSLTR1
CEU
YRI
CHB
0.00
0.01
0.02
0.03
0.04
0.05

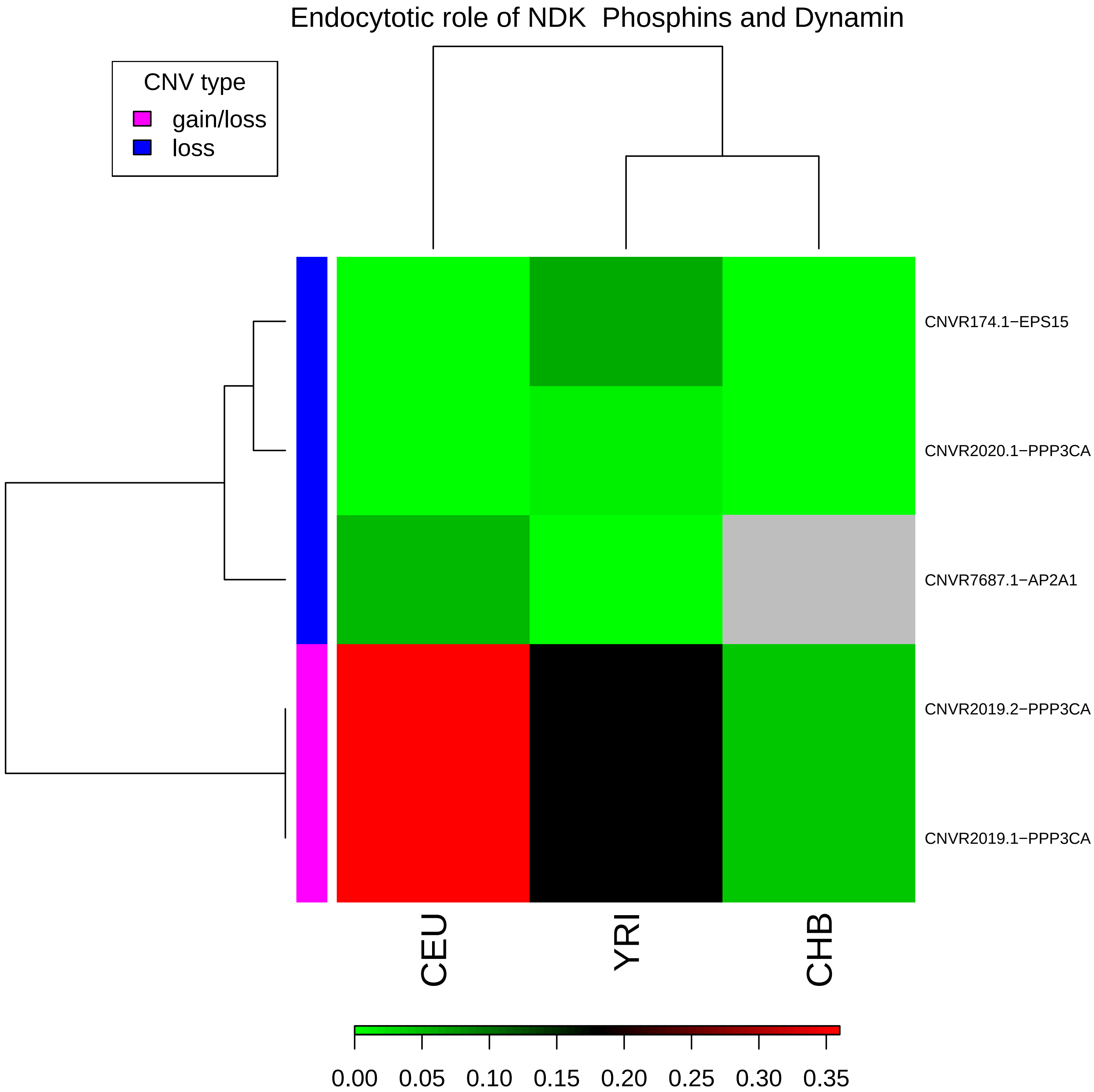
Endocytotic role of NDK  Phosphins and Dynamin
CNV type
gain/loss
loss
CNVR174.1−EPS15
CNVR2020.1−PPP3CA
CNVR7687.1−AP2A1
CNVR2019.2−PPP3CA
CNVR2019.1−PPP3CA
CEU
YRI
CHB
0.00
0.05
0.10
0.15
0.20
0.25
0.30
0.35

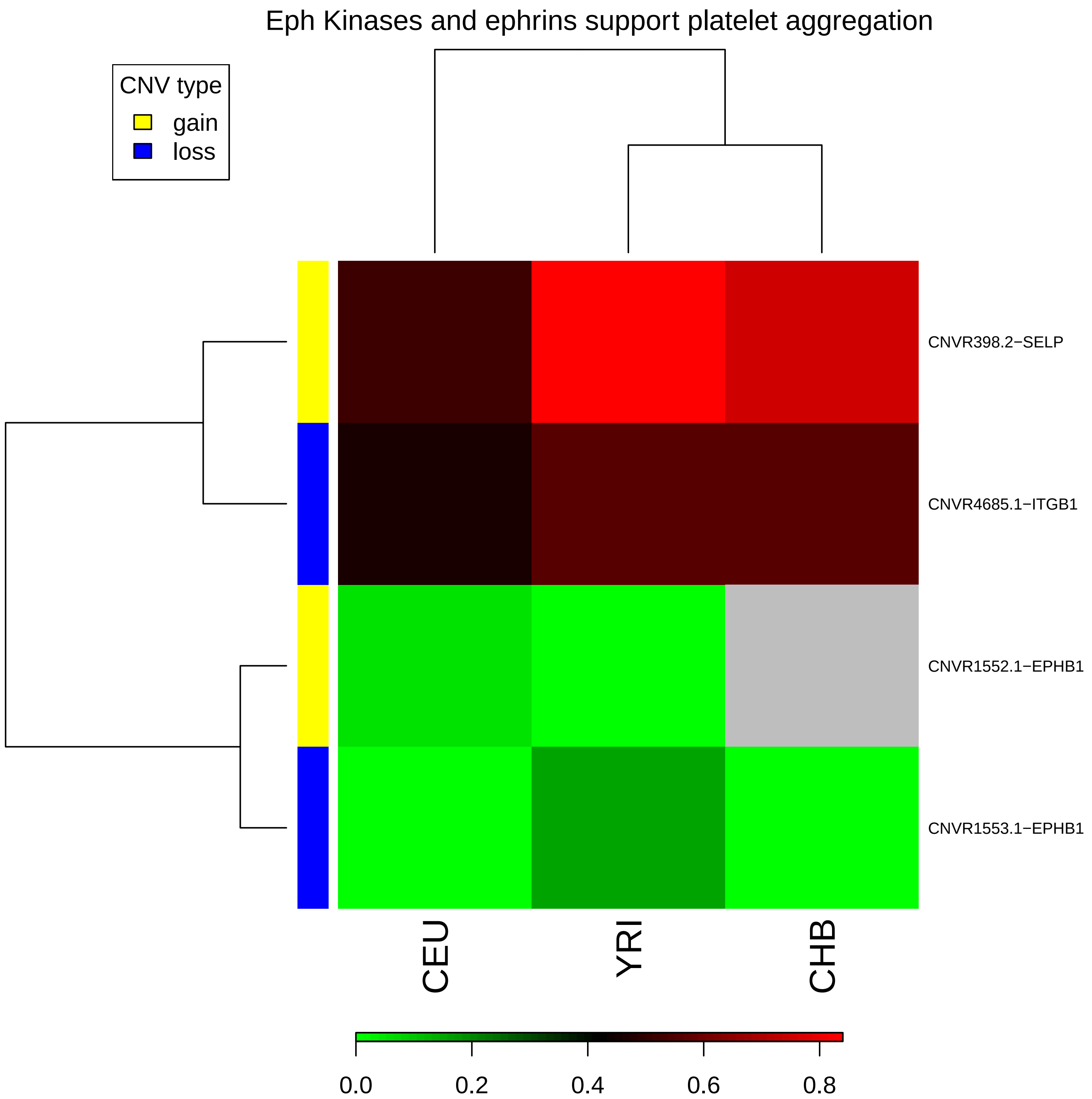
Eph Kinases and ephrins support platelet aggregation
CNV type
gain
loss
CNVR398.2−SELP
CNVR4685.1−ITGB1
CNVR1552.1−EPHB1
CNVR1553.1−EPHB1
CEU
YRI
CHB
0.0
0.2
0.4
0.6
0.8

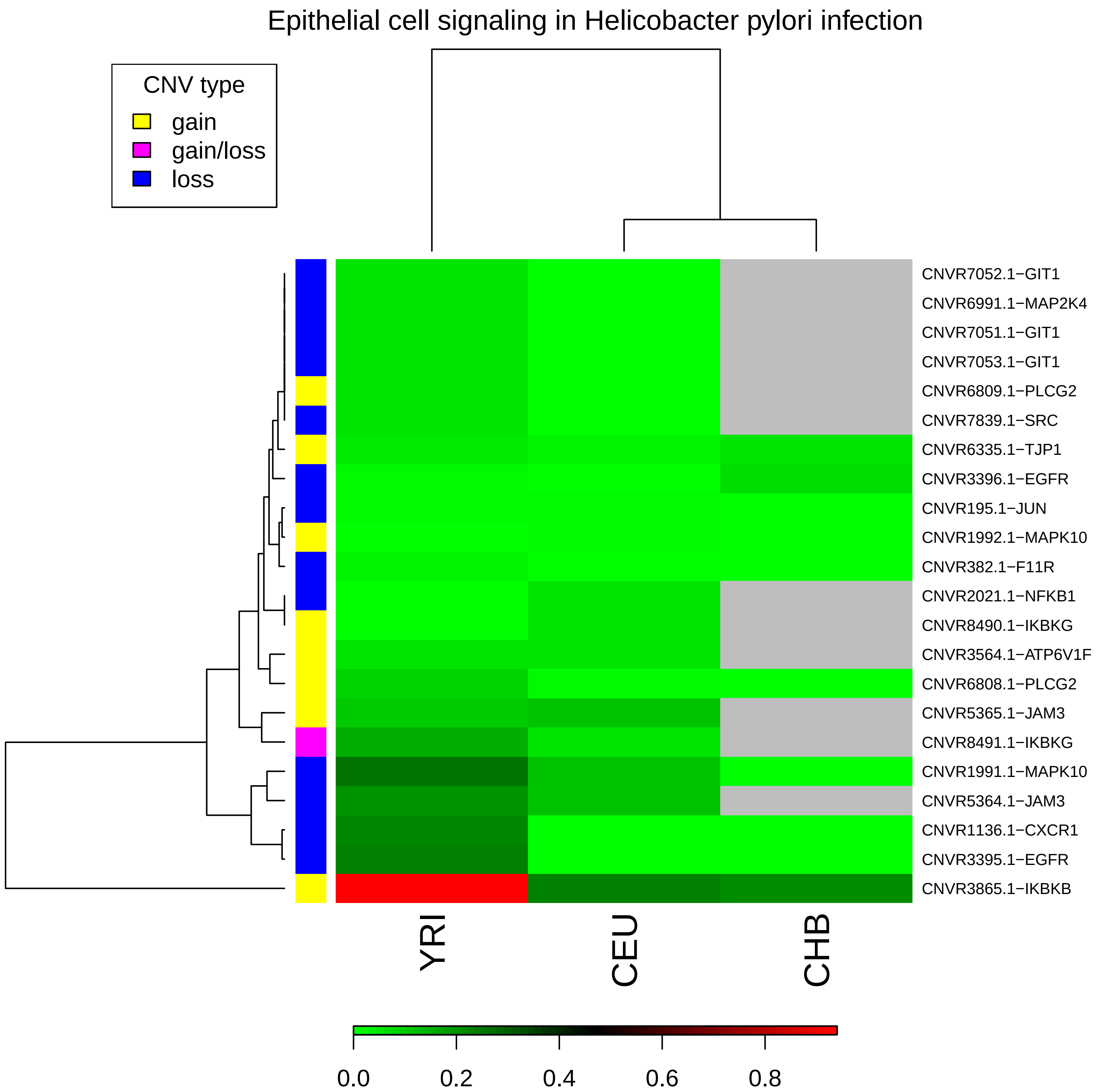
Epithelial cell signaling in Helicobacter pylori infection
CNV type
gain
gain/loss
loss
CNVR7052.1−GIT1
CNVR6991.1−MAP2K4
CNVR7051.1−GIT1
CNVR7053.1−GIT1
CNVR6809.1−PLCG2
CNVR7839.1−SRC
CNVR6335.1−TJP1
CNVR3396.1−EGFR
CNVR195.1−JUN
CNVR1992.1−MAPK10
CNVR382.1−F11R
CNVR2021.1−NFKB1
CNVR8490.1−IKBKG
CNVR3564.1−ATP6V1F
CNVR6808.1−PLCG2
CNVR5365.1−JAM3
CNVR8491.1−IKBKG
CNVR1991.1−MAPK10
CNVR5364.1−JAM3
CNVR1136.1−CXCR1
CNVR3395.1−EGFR
CNVR3865.1−IKBKB
YRI
CEU
CHB
0.0
0.2
0.4
0.6
0.8

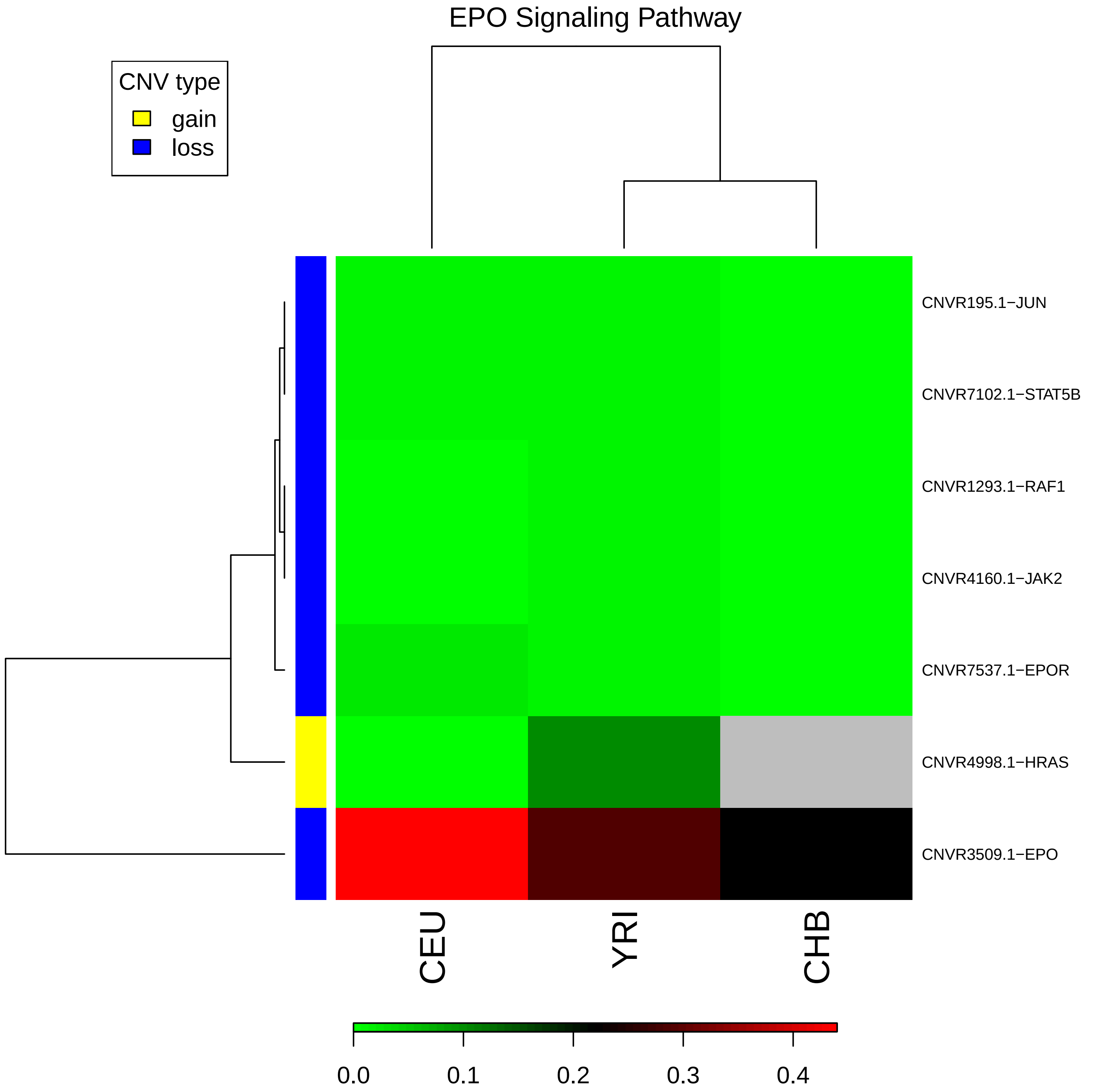
EPO Signaling Pathway
CNV type
gain
loss
CNVR195.1−JUN
CNVR7102.1−STAT5B
CNVR1293.1−RAF1
CNVR4160.1−JAK2
CNVR7537.1−EPOR
CNVR4998.1−HRAS
CNVR3509.1−EPO
CEU
YRI
CHB
0.0
0.1
0.2
0.3
0.4

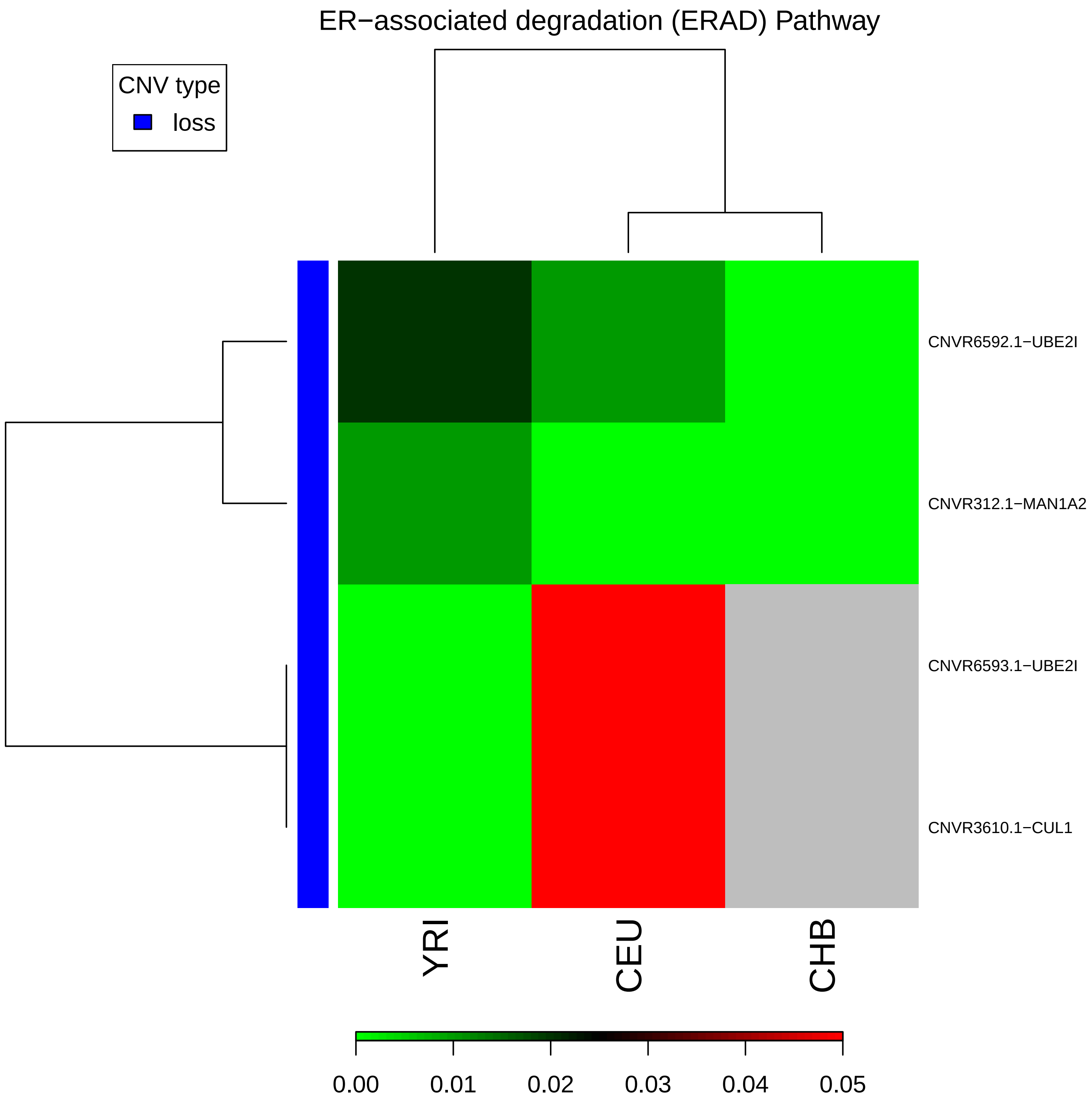
ER−associated degradation (ERAD) Pathway
CNV type
loss
CNVR6592.1−UBE2I
CNVR312.1−MAN1A2
CNVR6593.1−UBE2I
CNVR3610.1−CUL1
YRI
CEU
CHB
0.00
0.01
0.02
0.03
0.04
0.05

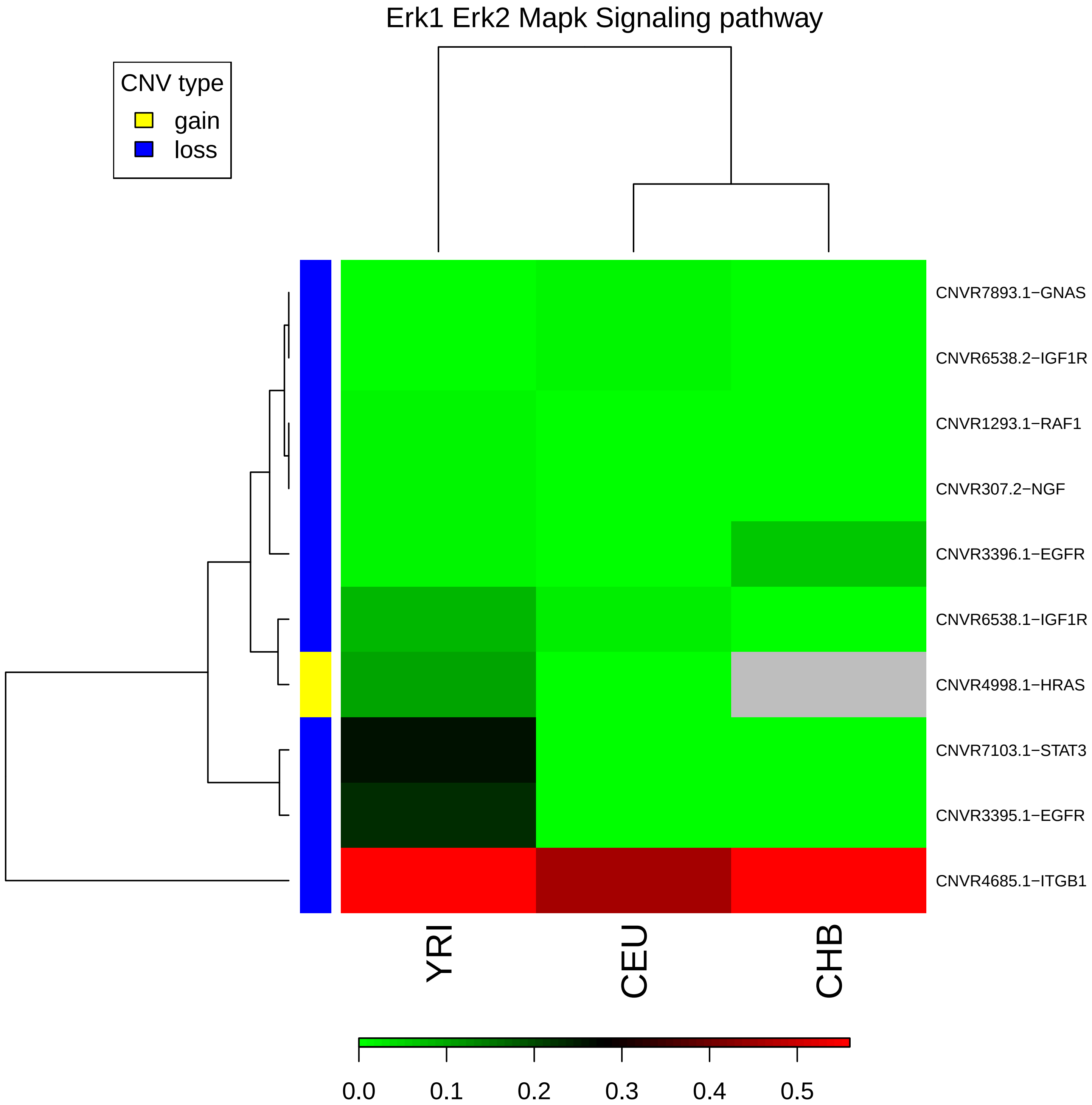

Erk1 Erk2 Mapk Signaling pathway
CNV type
gain
loss
CNVR7893.1−GNAS
CNVR6538.2−IGF1R
CNVR1293.1−RAF1
CNVR307.2−NGF
CNVR3396.1−EGFR
CNVR6538.1−IGF1R
CNVR4998.1−HRAS
CNVR7103.1−STAT3
CNVR3395.1−EGFR
CNVR4685.1−ITGB1
YRI
CEU
CHB
0.0
0.1
0.2
0.3
0.4
0.5

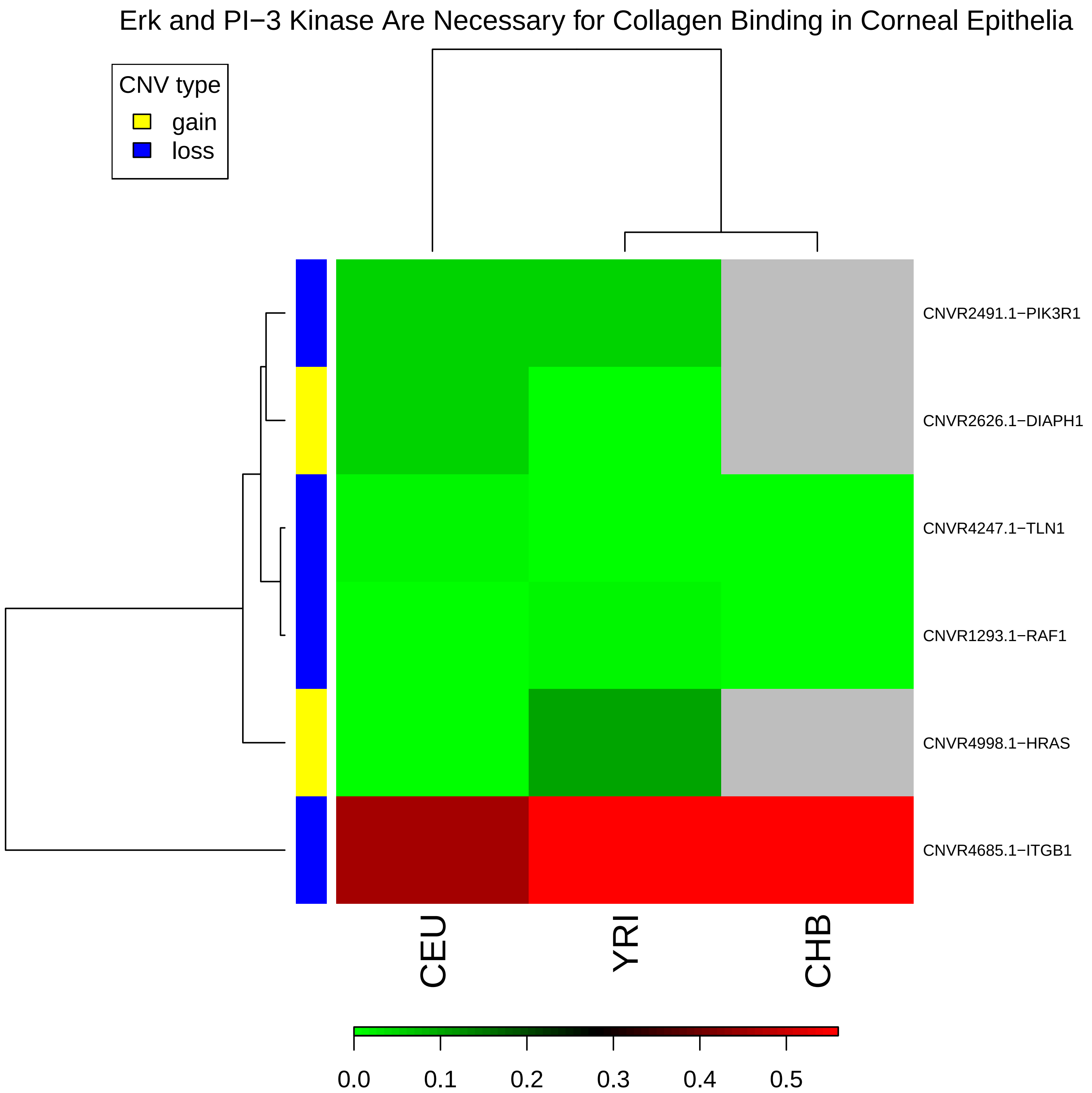
Erk and PI−3 Kinase Are Necessary for Collagen Binding in Corneal Epithelia
CNV type
gain
loss
CNVR2491.1−PIK3R1
CNVR2626.1−DIAPH1
CNVR4247.1−TLN1
CNVR1293.1−RAF1
CNVR4998.1−HRAS
CNVR4685.1−ITGB1
CEU
YRI
CHB
0.0
0.1
0.2
0.3
0.4
0.5

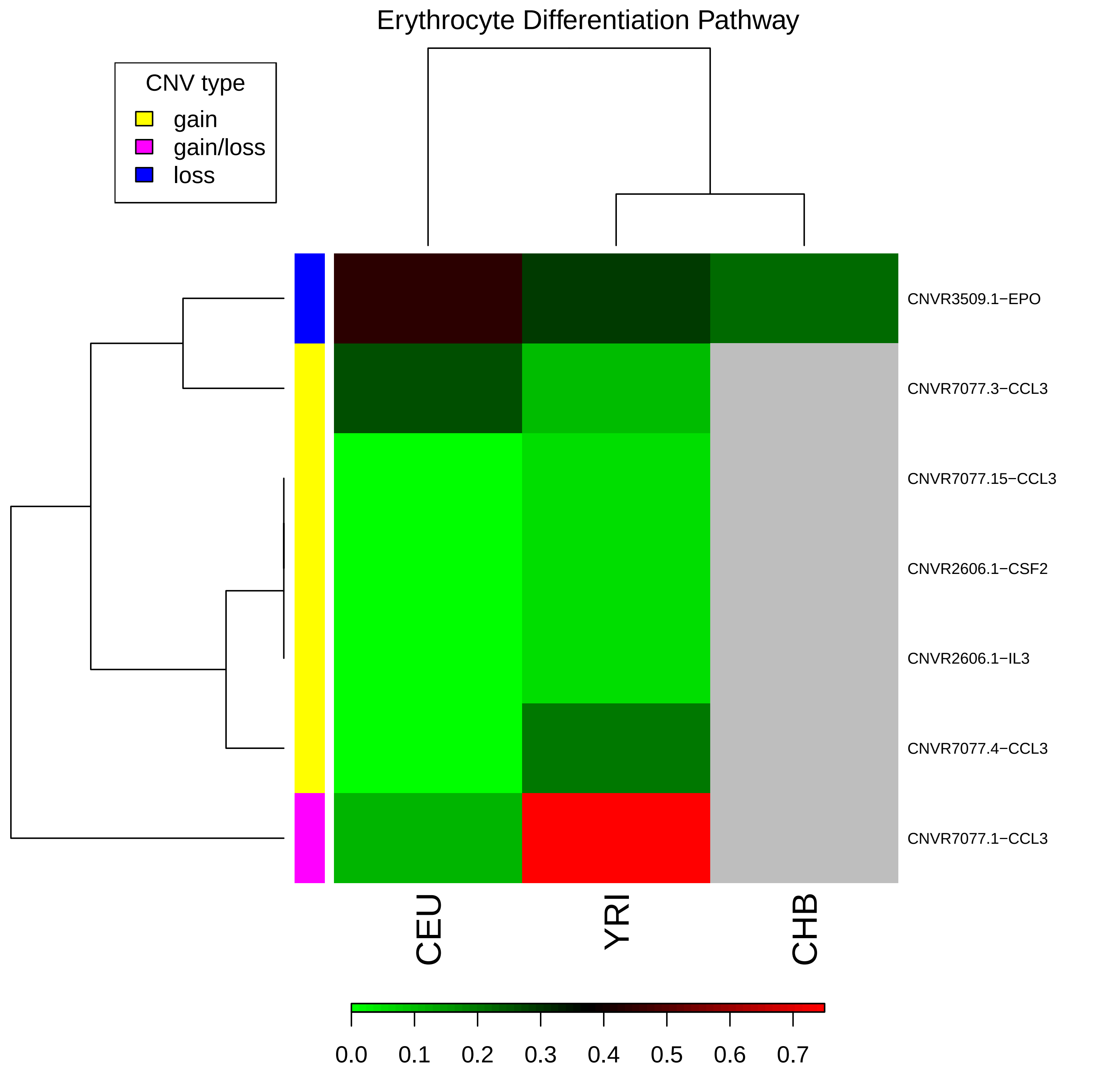
Erythrocyte Differentiation Pathway
CNV type
gain
gain/loss
loss
CNVR3509.1−EPO
CNVR7077.3−CCL3
CNVR7077.15−CCL3
CNVR2606.1−CSF2
CNVR2606.1−IL3
CNVR7077.4−CCL3
CNVR7077.1−CCL3
CEU
YRI
CHB
0.0
0.1
0.2
0.3
0.4
0.5
0.6
0.7

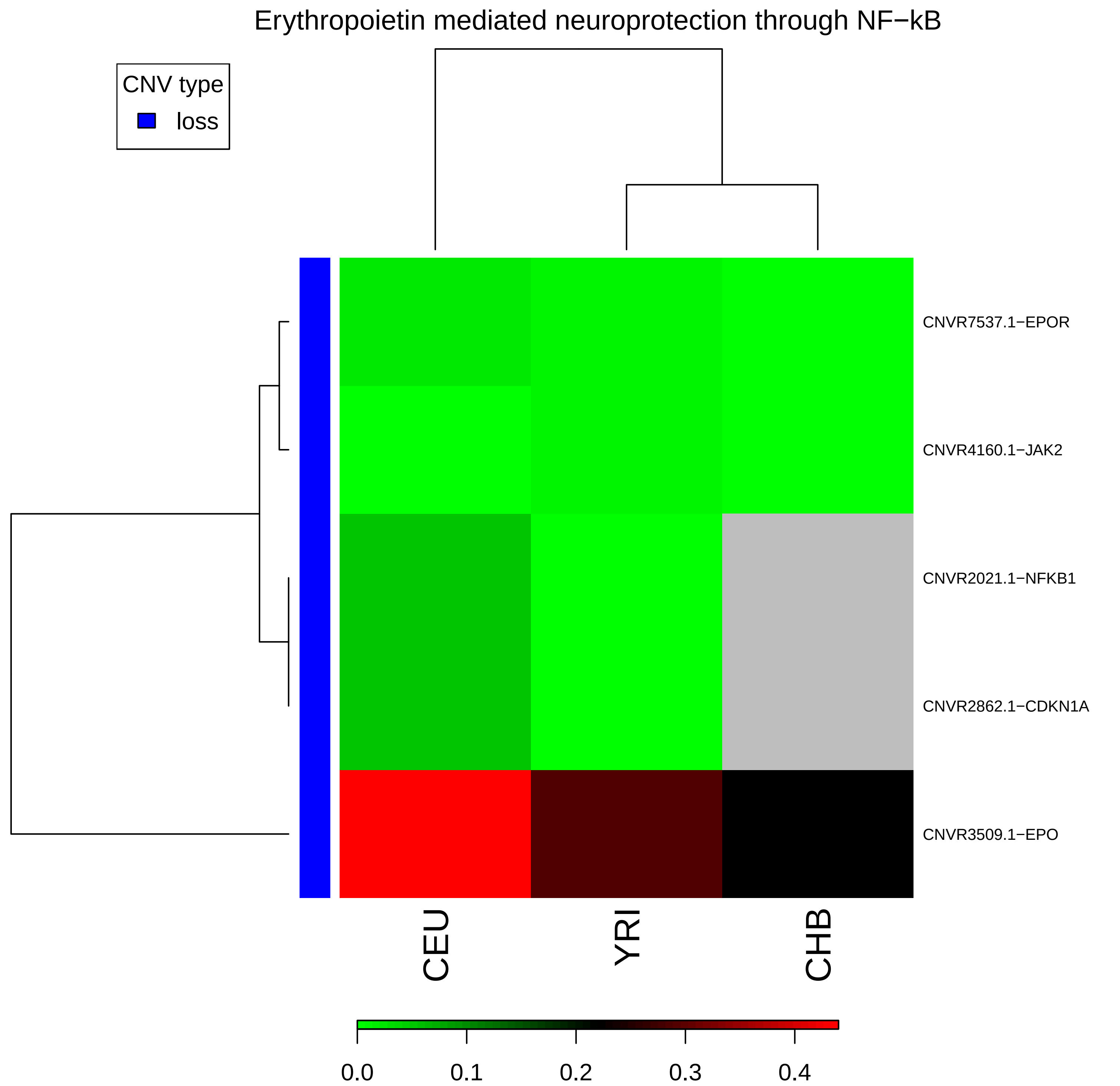
Erythropoietin mediated neuroprotection through NF−kB
CNV type
loss
CNVR7537.1−EPOR
CNVR4160.1−JAK2
CNVR2021.1−NFKB1
CNVR2862.1−CDKN1A
CNVR3509.1−EPO
CEU
YRI
CHB
0.0
0.1
0.2
0.3
0.4

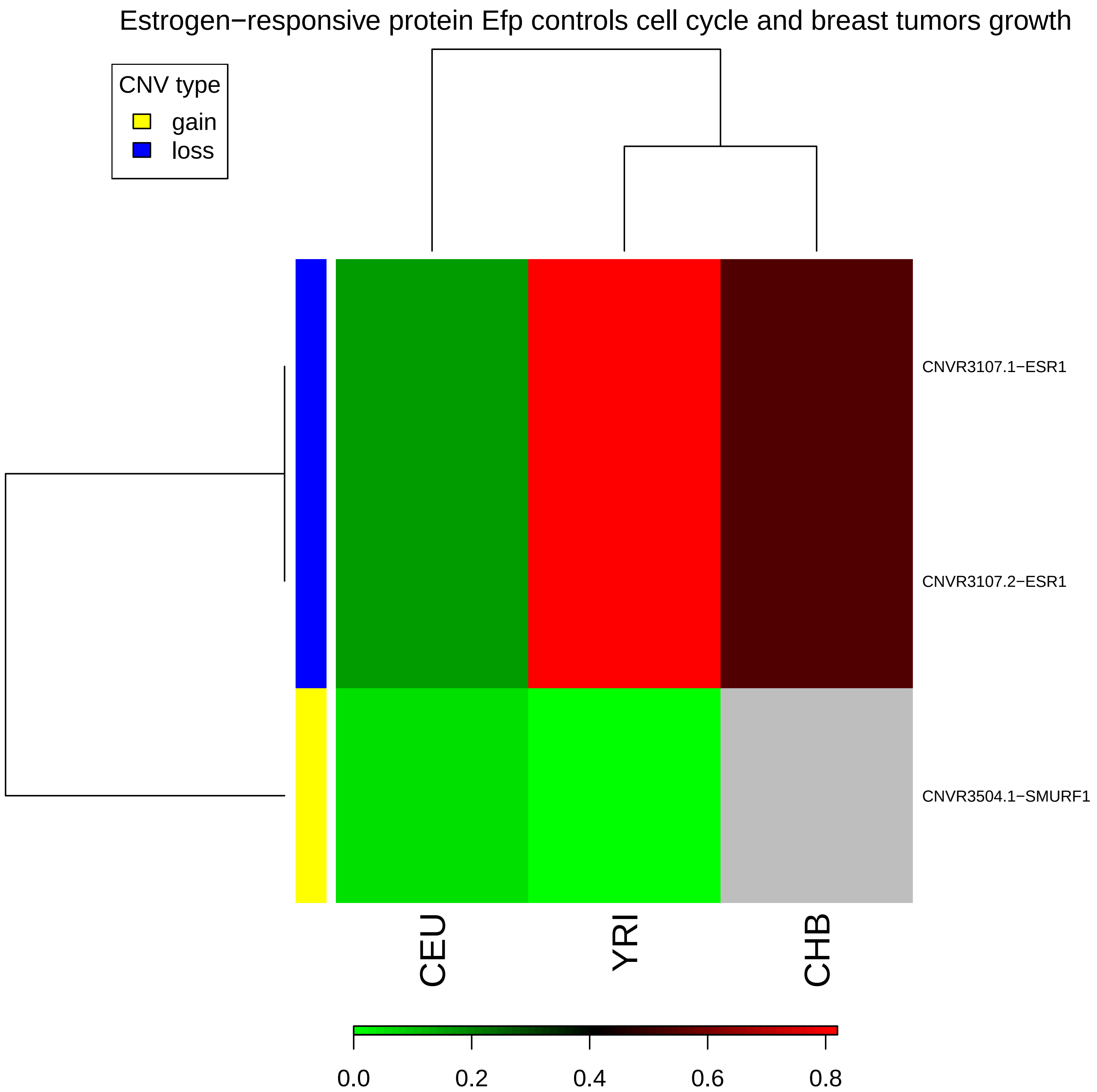
Estrogen−responsive protein Efp controls cell cycle and breast tumors growth
CNV type
gain
loss
CNVR3107.1−ESR1
CNVR3107.2−ESR1
CNVR3504.1−SMURF1
CEU
YRI
CHB
0.0
0.2
0.4
0.6
0.8

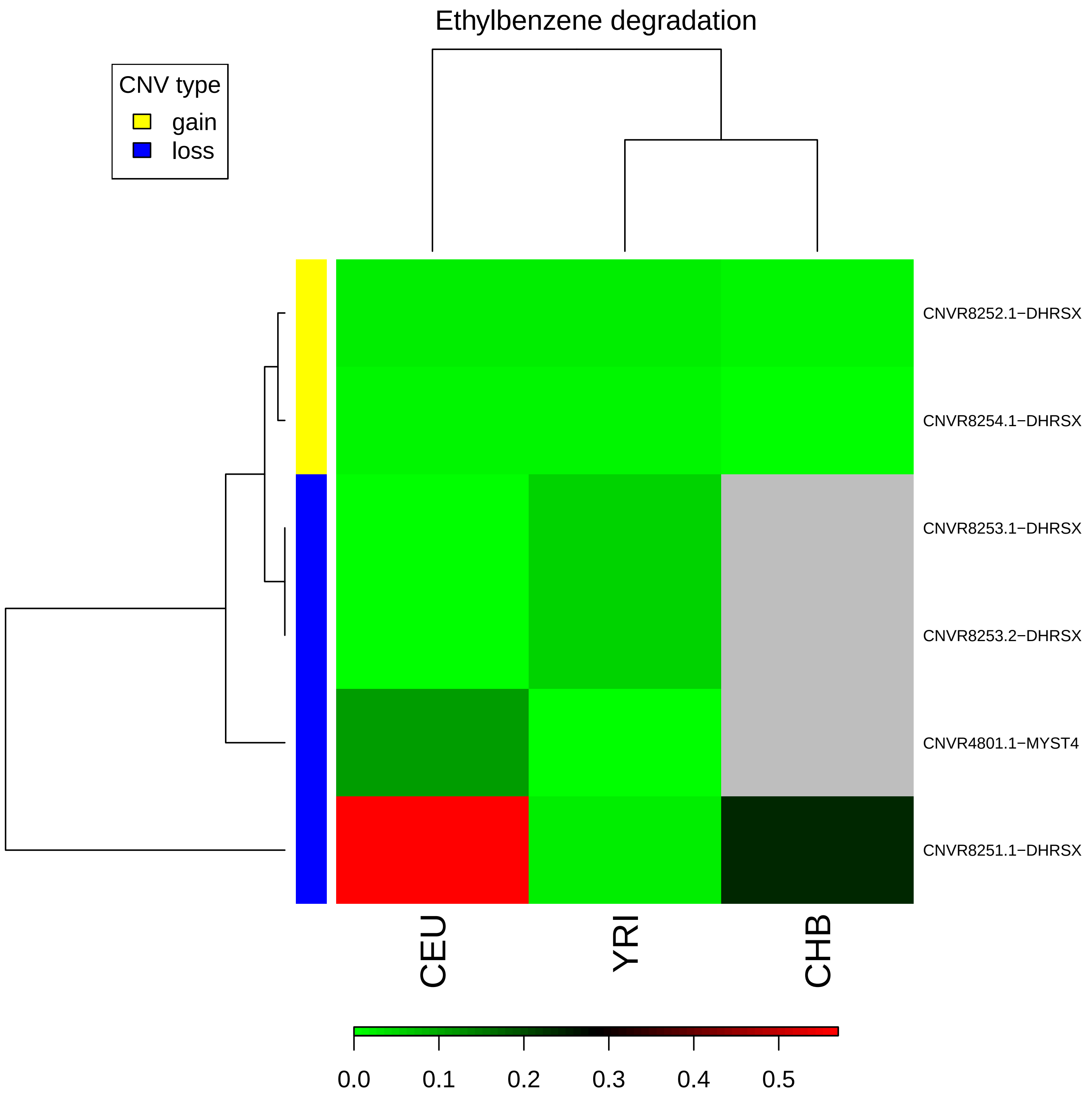

Ethylbenzene degradation
CNV type
gain
loss
CNVR8252.1−DHRSX
CNVR8254.1−DHRSX
CNVR8253.1−DHRSX
CNVR8253.2−DHRSX
CNVR4801.1−MYST4
CNVR8251.1−DHRSX
CEU
YRI
CHB
0.0
0.1
0.2
0.3
0.4
0.5

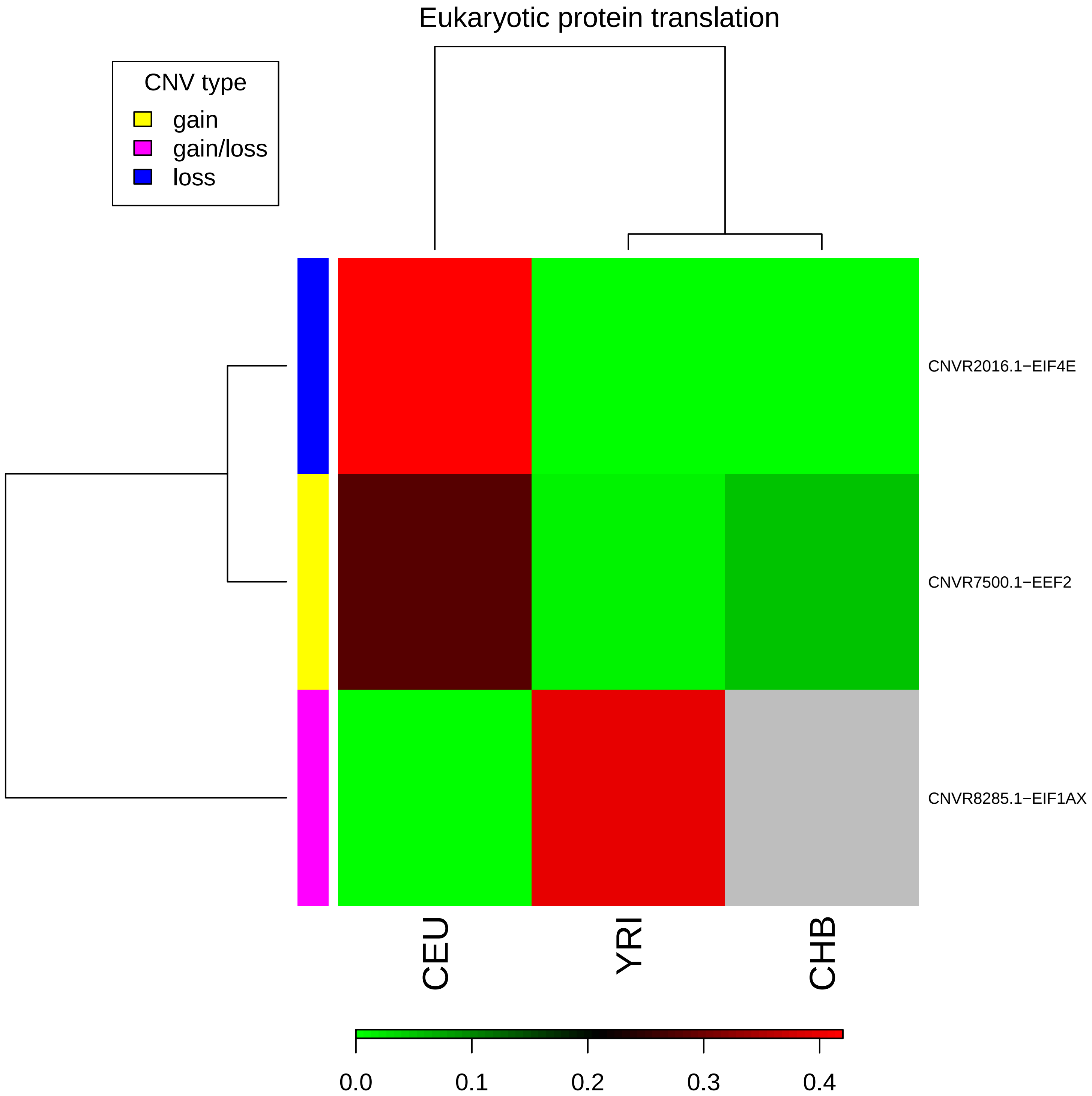

Eukaryotic protein translation
CNV type
gain
gain/loss
loss
CNVR2016.1−EIF4E
CNVR7500.1−EEF2
CNVR8285.1−EIF1AX
CEU
YRI
CHB
0.0
0.1
0.2
0.3
0.4

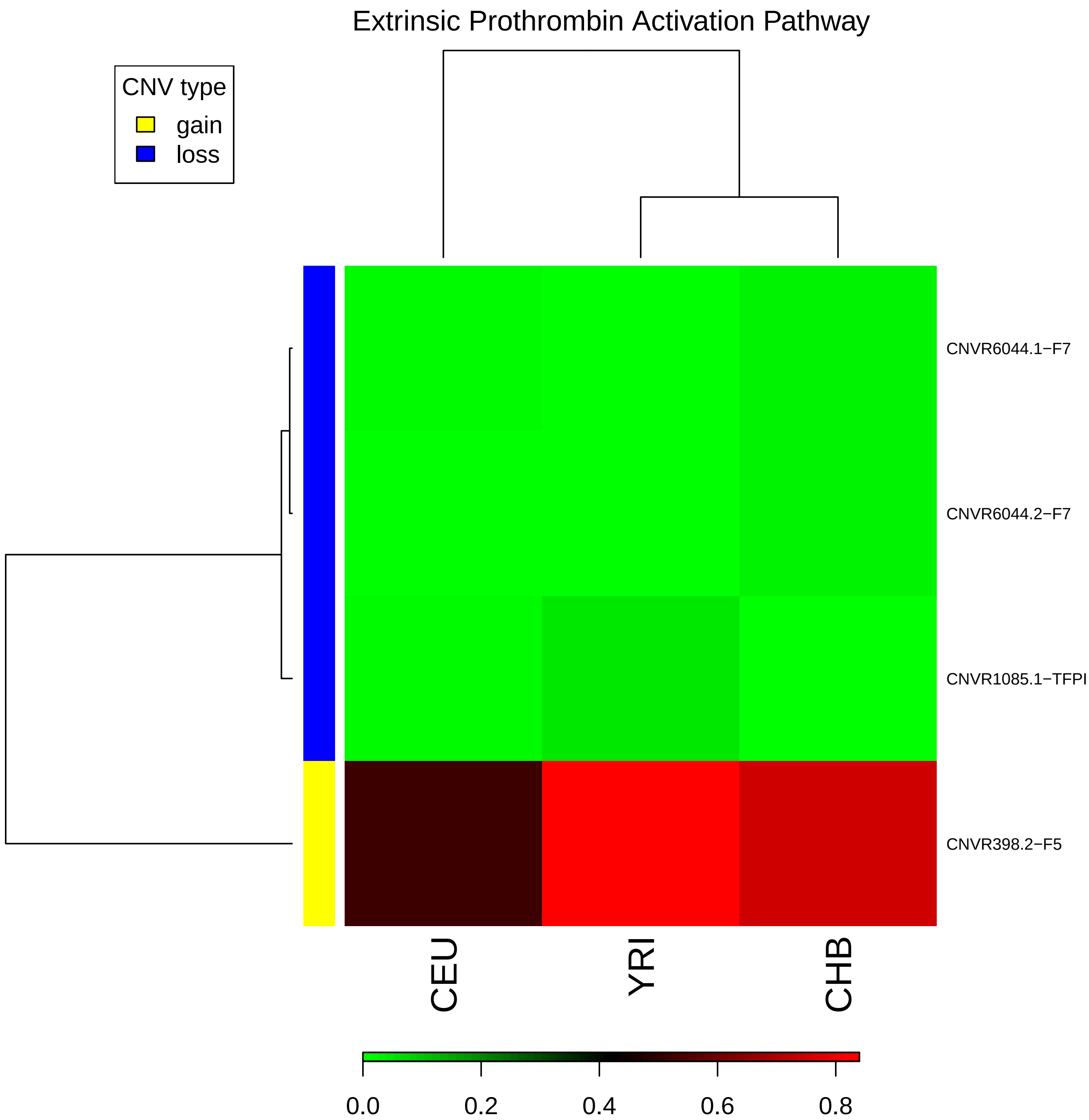

Extrinsic Prothrombin Activation Pathway
CNV type
gain
loss
CNVR6044.1−F7
CNVR6044.2−F7
CNVR1085.1−TFPI
CNVR398.2−F5
CEU
YRI
CHB
0.0
0.2
0.4
0.6
0.8

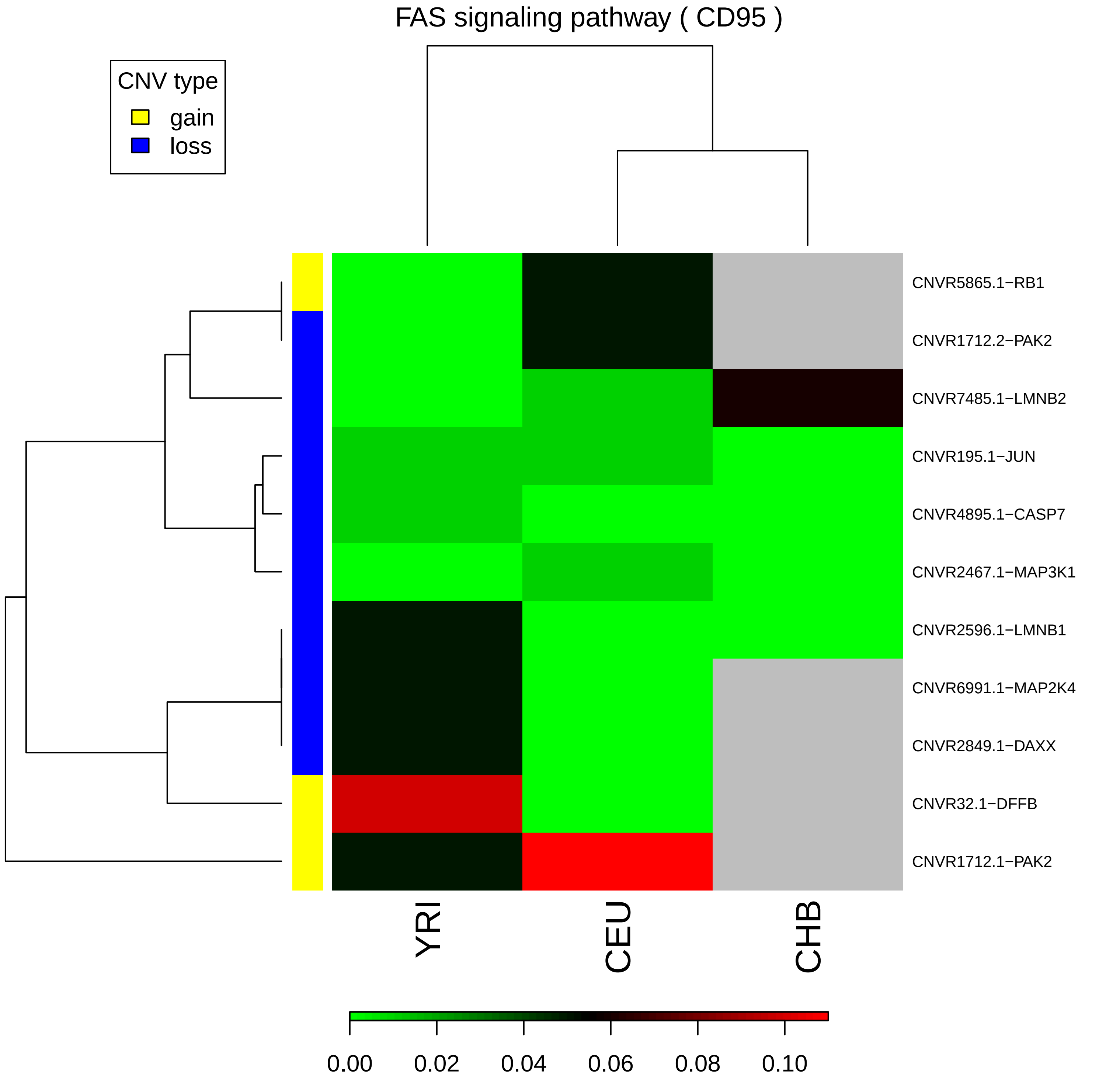

FAS signaling pathway ( CD95 )
CNV type
gain
loss
CNVR5865.1−RB1
CNVR1712.2−PAK2
CNVR7485.1−LMNB2
CNVR195.1−JUN
CNVR4895.1−CASP7
CNVR2467.1−MAP3K1
CNVR2596.1−LMNB1
CNVR6991.1−MAP2K4
CNVR2849.1−DAXX
CNVR32.1−DFFB
CNVR1712.1−PAK2
YRI
CEU
CHB
0.00
0.02
0.04
0.06
0.08
0.10

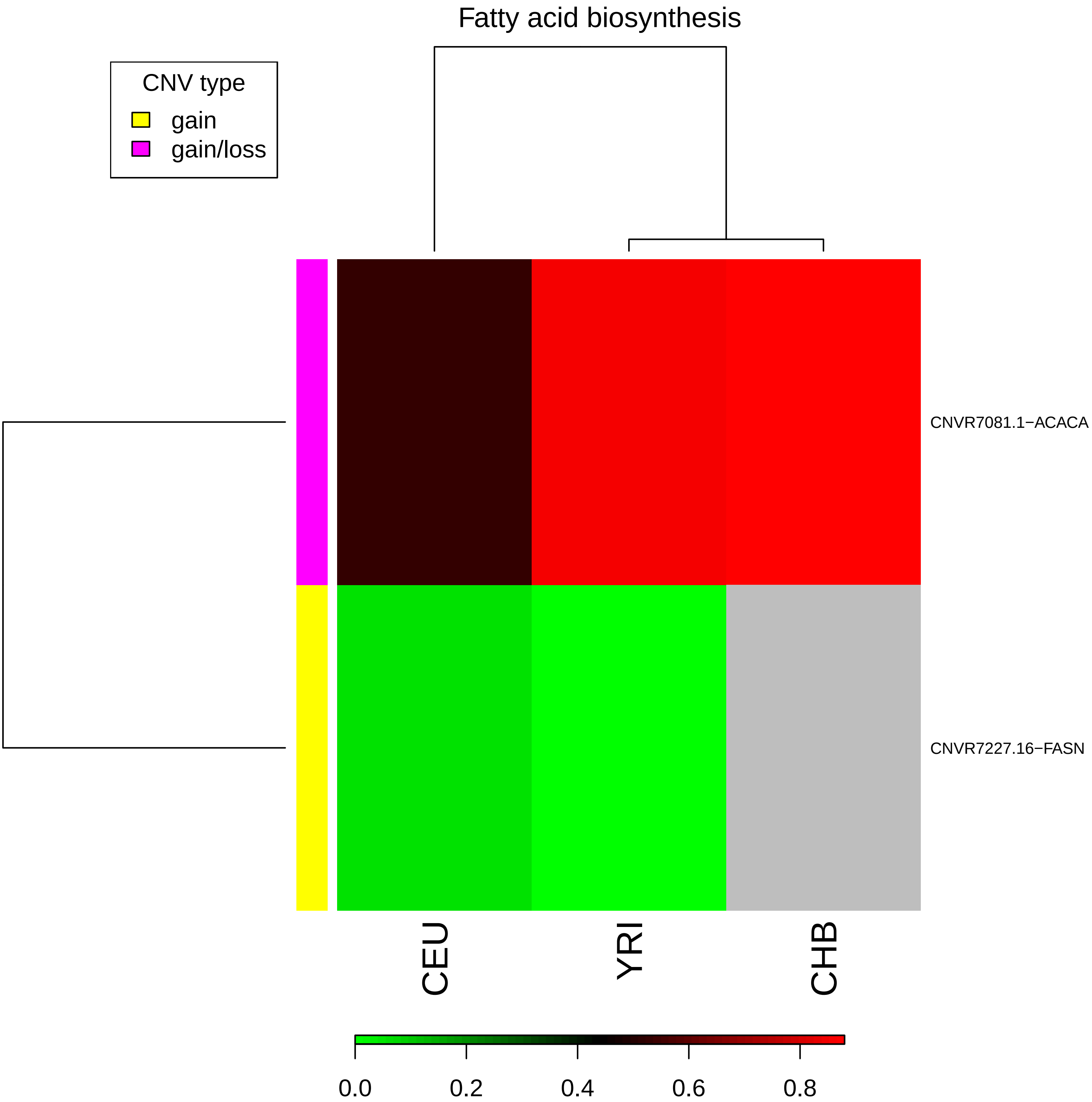

Fatty acid biosynthesis
CNV type
gain
gain/loss
CNVR7081.1−ACACA
CNVR7227.16−FASN
CEU
YRI
CHB
0.0
0.2
0.4
0.6
0.8

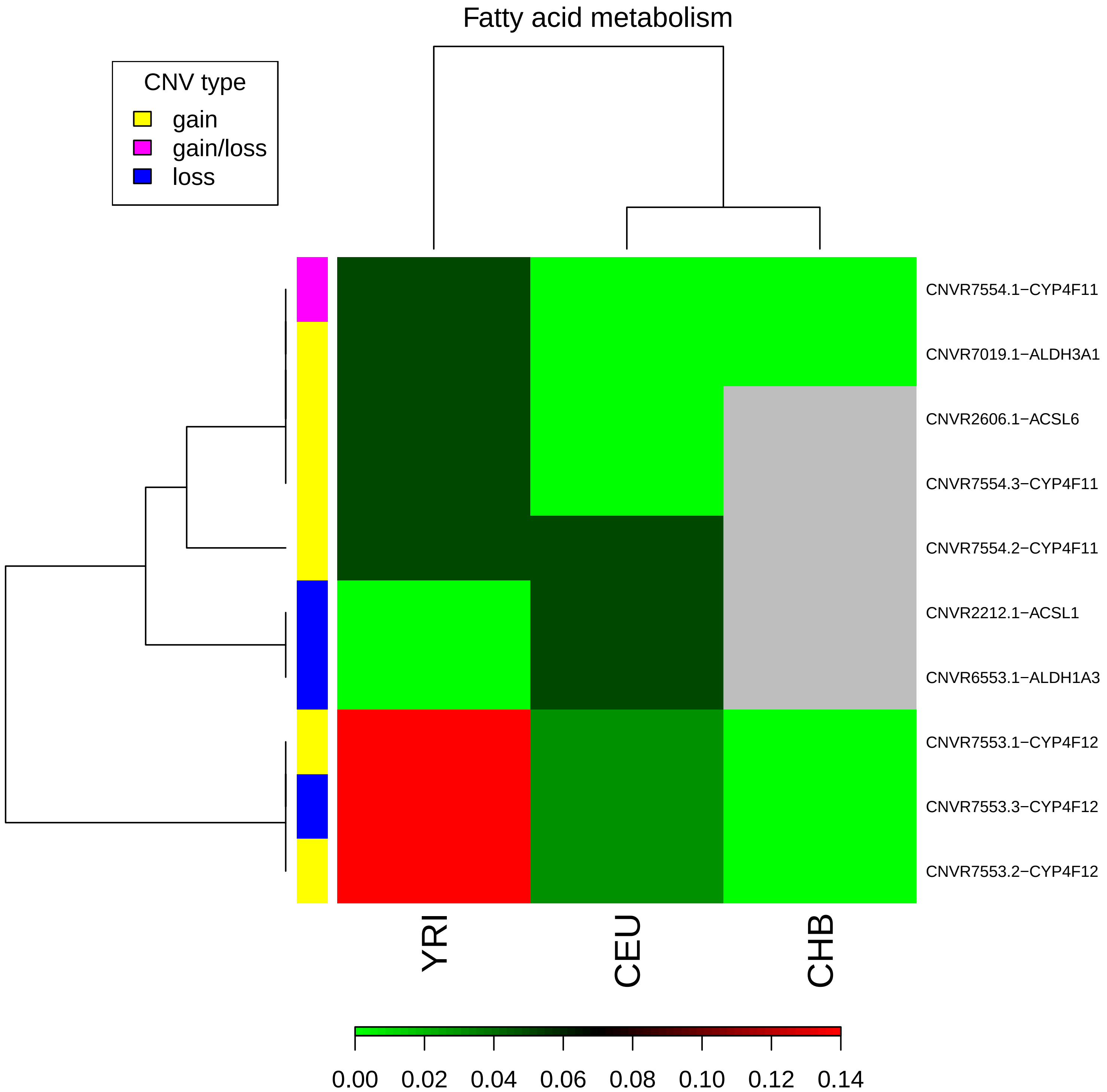

Fatty acid metabolism
CNV type
gain
gain/loss
loss
CNVR7554.1−CYP4F11
CNVR7019.1−ALDH3A1
CNVR2606.1−ACSL6
CNVR7554.3−CYP4F11
CNVR7554.2−CYP4F11
CNVR2212.1−ACSL1
CNVR6553.1−ALDH1A3
CNVR7553.1−CYP4F12
CNVR7553.3−CYP4F12
CNVR7553.2−CYP4F12
YRI
CEU
CHB
0.00
0.02
0.04
0.06
0.08
0.10
0.12
0.14

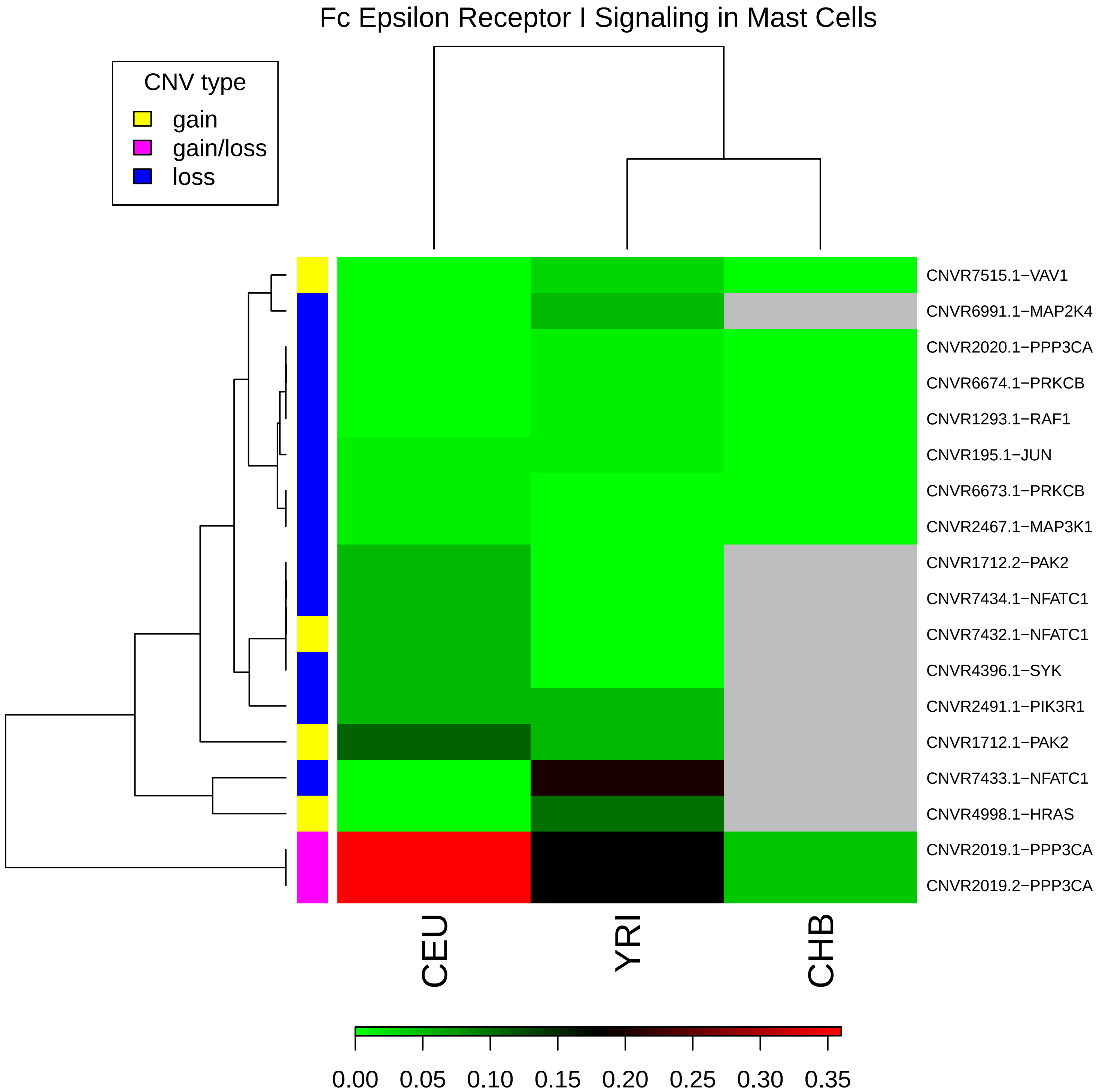
Fc Epsilon Receptor I Signaling in Mast Cells
CNV type
gain
gain/loss
loss
CNVR7515.1−VAV1
CNVR6991.1−MAP2K4
CNVR2020.1−PPP3CA
CNVR6674.1−PRKCB
CNVR1293.1−RAF1
CNVR195.1−JUN
CNVR6673.1−PRKCB
CNVR2467.1−MAP3K1
CNVR1712.2−PAK2
CNVR7434.1−NFATC1
CNVR7432.1−NFATC1
CNVR4396.1−SYK
CNVR2491.1−PIK3R1
CNVR1712.1−PAK2
CNVR7433.1−NFATC1
CNVR4998.1−HRAS
CNVR2019.1−PPP3CA
CNVR2019.2−PPP3CA
CEU
YRI
CHB
0.00
0.05
0.10
0.15
0.20
0.25
0.30
0.35

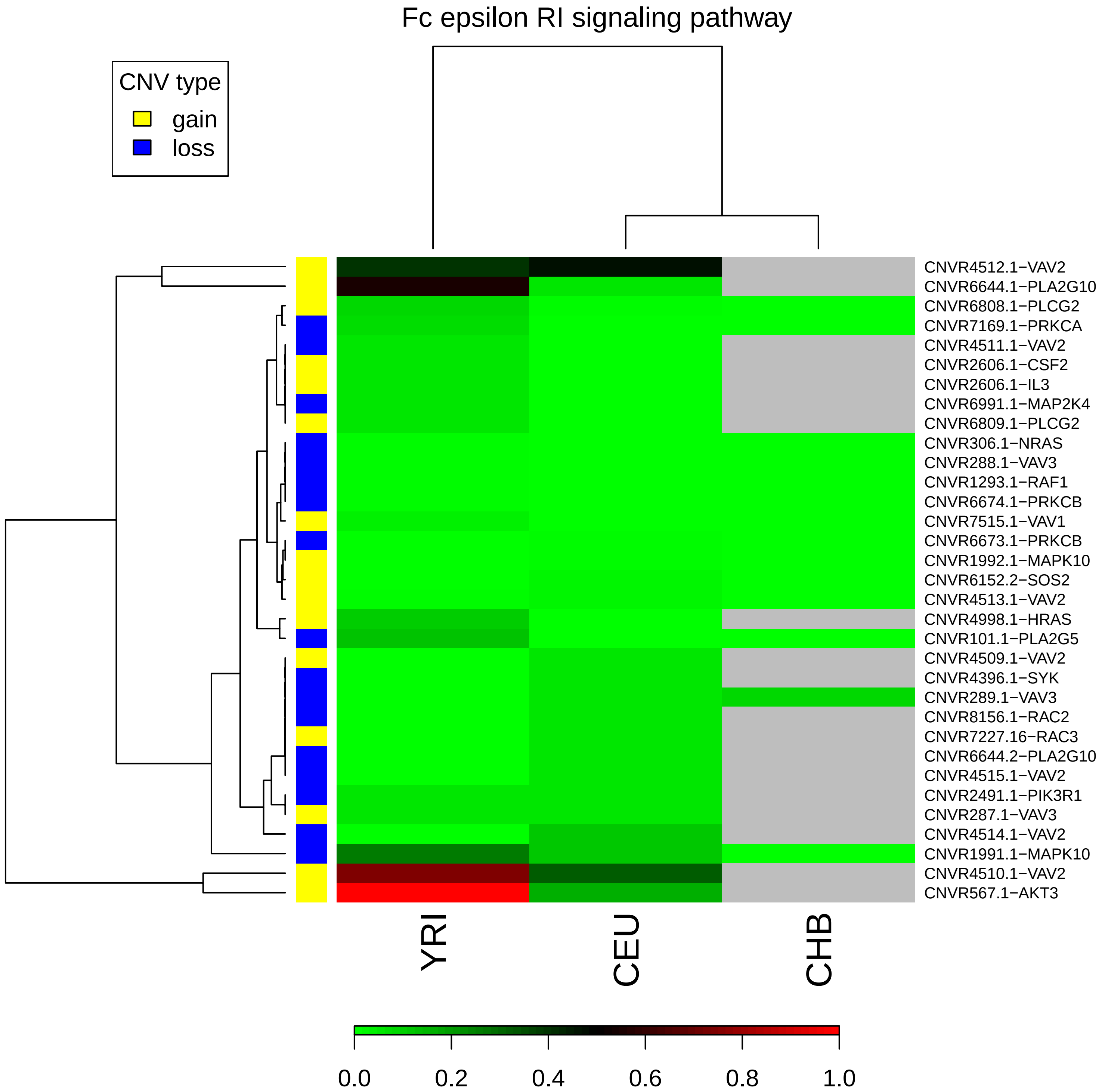

Fc epsilon RI signaling pathway
CNV type
gain
loss
CNVR4512.1−VAV2
CNVR6644.1−PLA2G10
CNVR6808.1−PLCG2
CNVR7169.1−PRKCA
CNVR4511.1−VAV2
CNVR2606.1−CSF2
CNVR2606.1−IL3
CNVR6991.1−MAP2K4
CNVR6809.1−PLCG2
CNVR306.1−NRAS
CNVR288.1−VAV3
CNVR1293.1−RAF1
CNVR6674.1−PRKCB
CNVR7515.1−VAV1
CNVR6673.1−PRKCB
CNVR1992.1−MAPK10
CNVR6152.2−SOS2
CNVR4513.1−VAV2
CNVR4998.1−HRAS
CNVR101.1−PLA2G5
CNVR4509.1−VAV2
CNVR4396.1−SYK
CNVR289.1−VAV3
CNVR8156.1−RAC2
CNVR7227.16−RAC3
CNVR6644.2−PLA2G10
CNVR4515.1−VAV2
CNVR2491.1−PIK3R1
CNVR287.1−VAV3
CNVR4514.1−VAV2
CNVR1991.1−MAPK10
CNVR4510.1−VAV2
CNVR567.1−AKT3
YRI
CEU
CHB
0.0
0.2
0.4
0.6
0.8
1.0

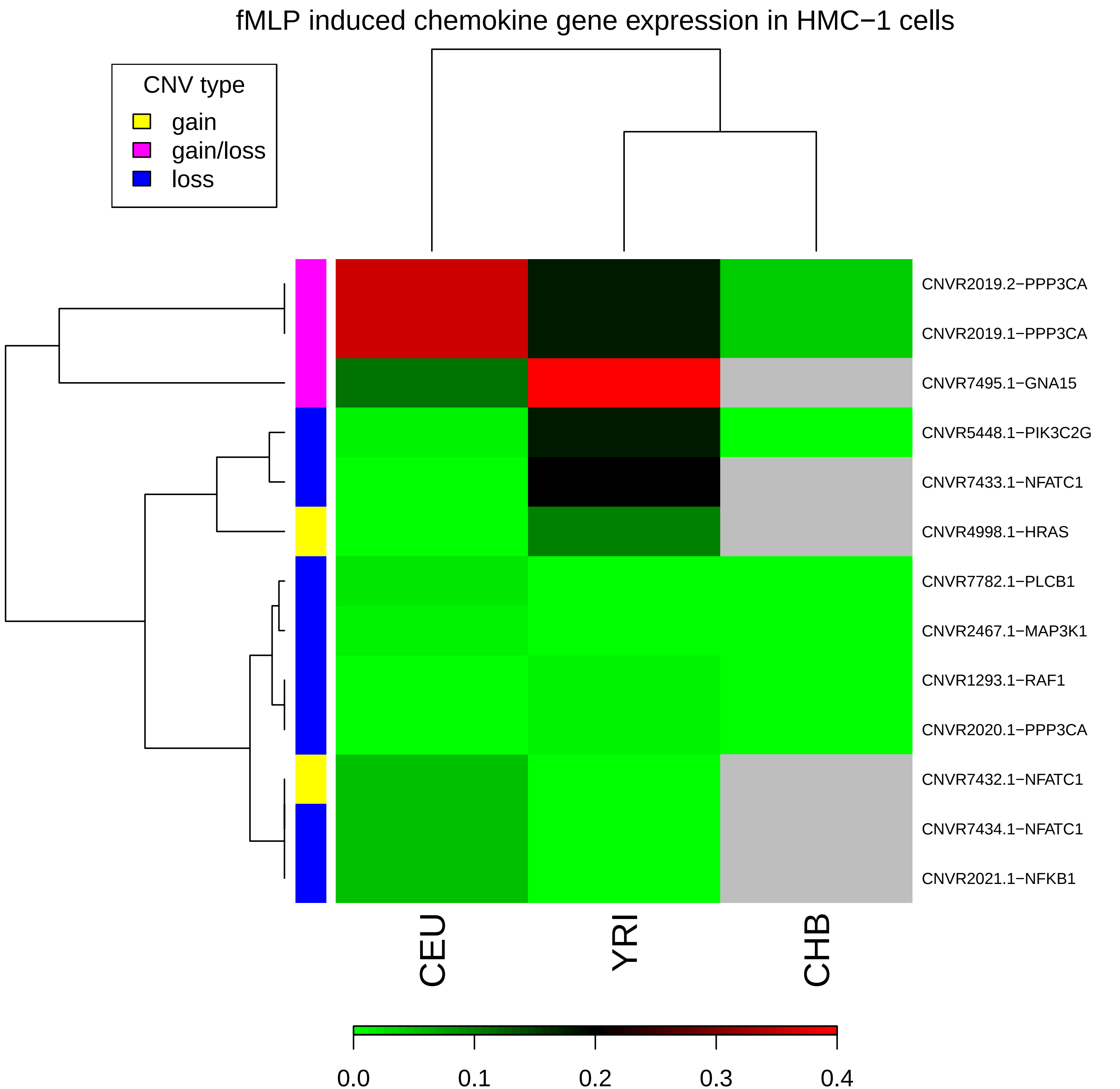
fMLP induced chemokine gene expression in HMC−1 cells
CNV type
gain
gain/loss
loss
CNVR2019.2−PPP3CA
CNVR2019.1−PPP3CA
CNVR7495.1−GNA15
CNVR5448.1−PIK3C2G
CNVR7433.1−NFATC1
CNVR4998.1−HRAS
CNVR7782.1−PLCB1
CNVR2467.1−MAP3K1
CNVR1293.1−RAF1
CNVR2020.1−PPP3CA
CNVR7432.1−NFATC1
CNVR7434.1−NFATC1
CNVR2021.1−NFKB1
CEU
YRI
CHB
0.0
0.1
0.2
0.3
0.4

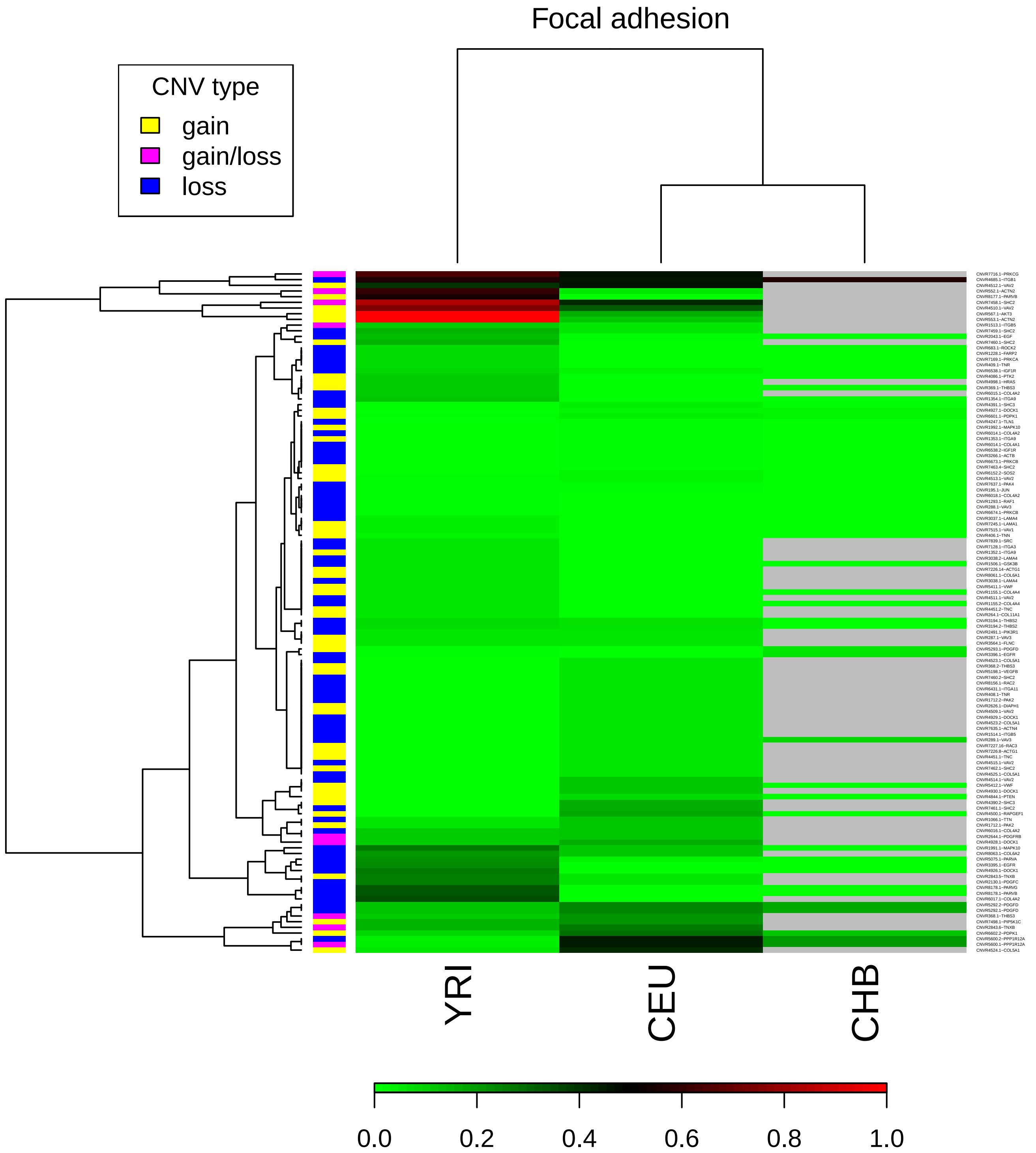

Focal adhesion
CNV type
gain
gain/loss
loss
YRI
CEU
CHB
0.0
0.2
0.4
0.6
0.8
1.0

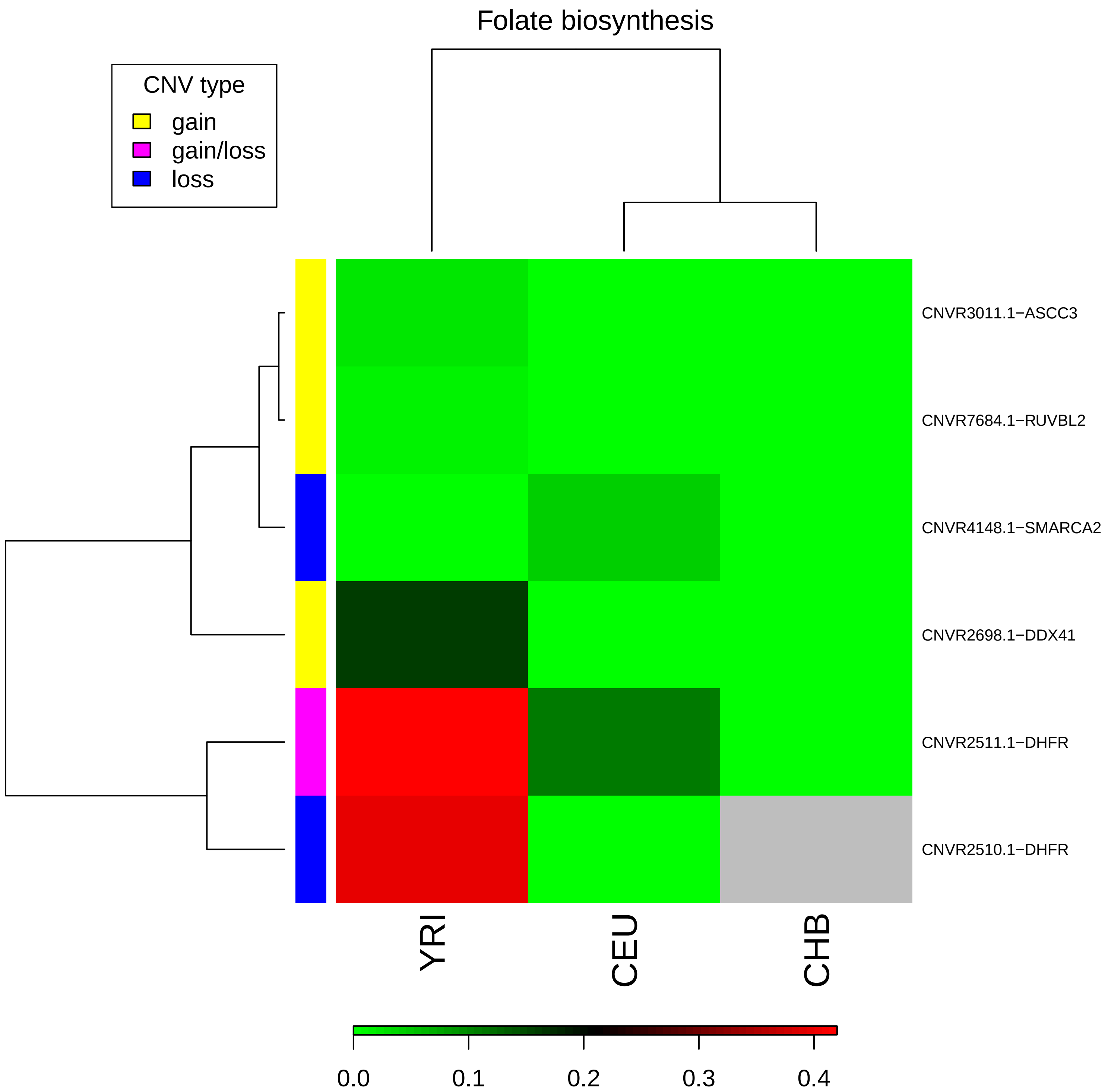
Folate biosynthesis
CNV type
gain
gain/loss
loss
CNVR3011.1−ASCC3
CNVR7684.1−RUVBL2
CNVR4148.1−SMARCA2
CNVR2698.1−DDX41
CNVR2511.1−DHFR
CNVR2510.1−DHFR
YRI
CEU
CHB
0.0
0.1
0.2
0.3
0.4

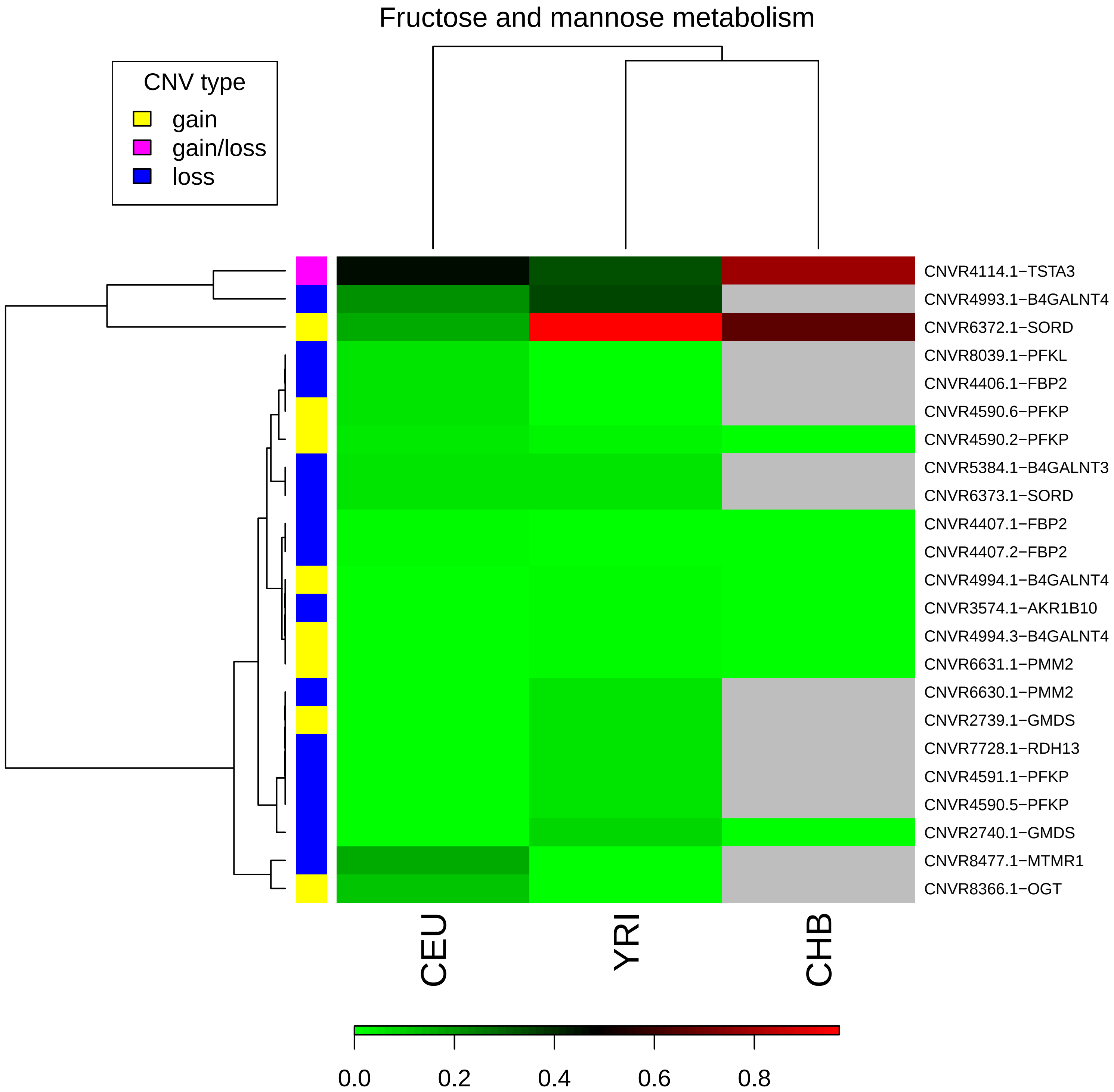
Fructose and mannose metabolism
CNV type
gain
gain/loss
loss
CNVR4114.1−TSTA3
CNVR4993.1−B4GALNT4
CNVR6372.1−SORD
CNVR8039.1−PFKL
CNVR4406.1−FBP2
CNVR4590.6−PFKP
CNVR4590.2−PFKP
CNVR5384.1−B4GALNT3
CNVR6373.1−SORD
CNVR4407.1−FBP2
CNVR4407.2−FBP2
CNVR4994.1−B4GALNT4
CNVR3574.1−AKR1B10
CNVR4994.3−B4GALNT4
CNVR6631.1−PMM2
CNVR6630.1−PMM2
CNVR2739.1−GMDS
CNVR7728.1−RDH13
CNVR4591.1−PFKP
CNVR4590.5−PFKP
CNVR2740.1−GMDS
CNVR8477.1−MTMR1
CNVR8366.1−OGT
CEU
YRI
CHB
0.0
0.2
0.4
0.6
0.8

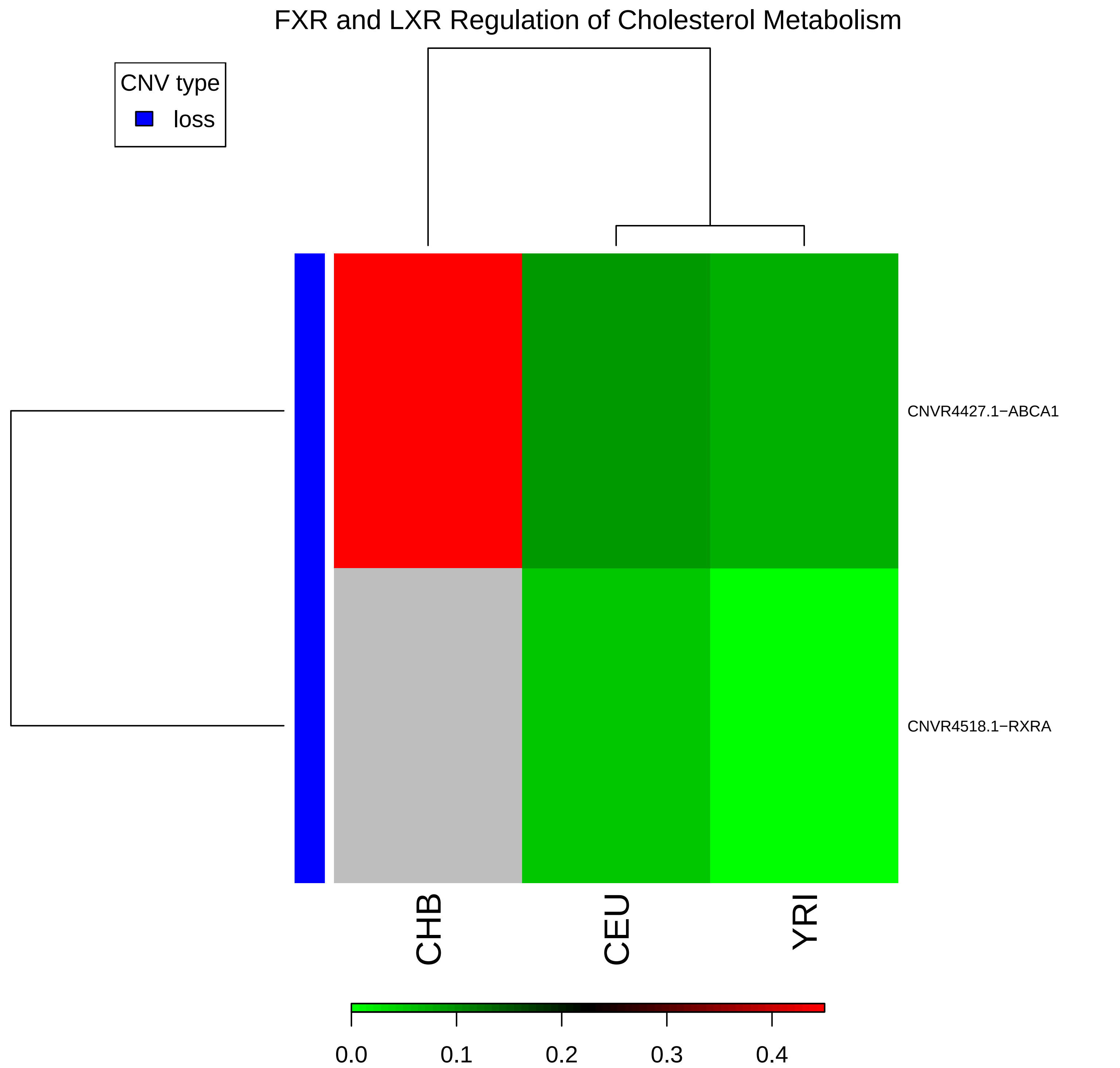
FXR and LXR Regulation of Cholesterol Metabolism
CNV type
loss
CNVR4427.1−ABCA1
CNVR4518.1−RXRA
CHB
CEU
YRI
0.0
0.1
0.2
0.3
0.4

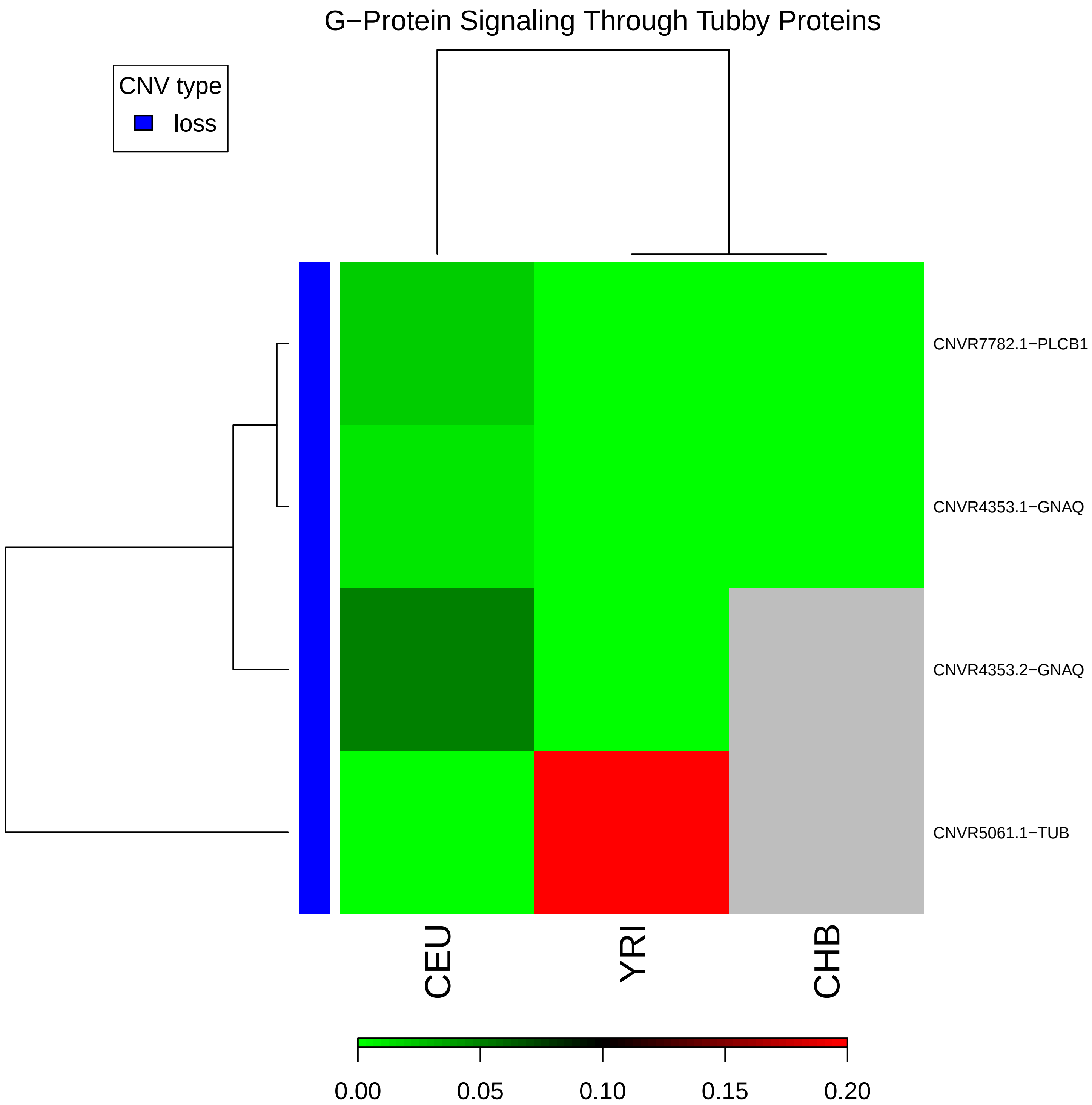
G−Protein Signaling Through Tubby Proteins
CNV type
loss
CNVR7782.1−PLCB1
CNVR4353.1−GNAQ
CNVR4353.2−GNAQ
CNVR5061.1−TUB
CEU
YRI
CHB
0.00
0.05
0.10
0.15
0.20

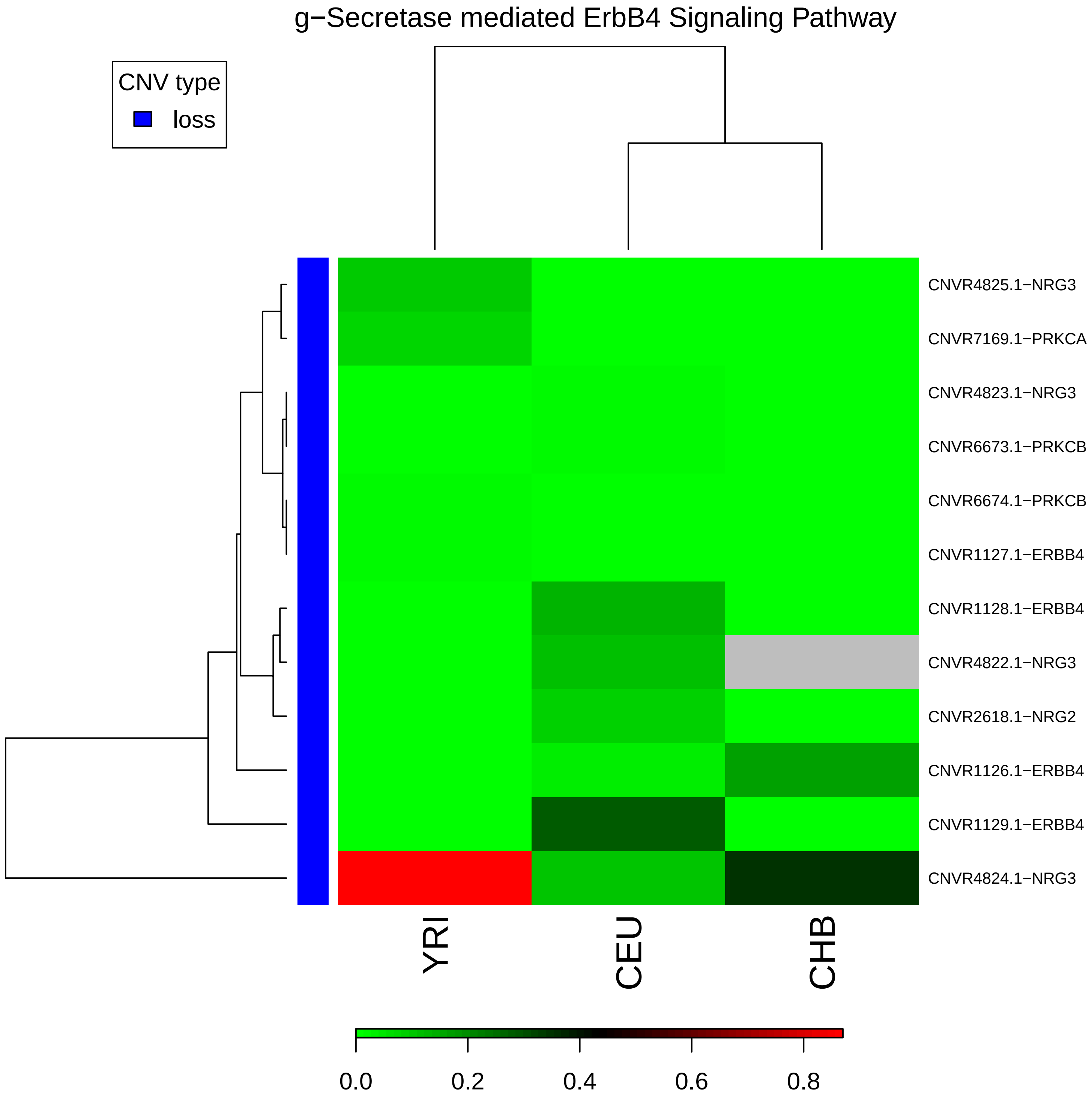

g−Secretase mediated ErbB4 Signaling Pathway
CNV type
loss
CNVR4825.1−NRG3
CNVR7169.1−PRKCA
CNVR4823.1−NRG3
CNVR6673.1−PRKCB
CNVR6674.1−PRKCB
CNVR1127.1−ERBB4
CNVR1128.1−ERBB4
CNVR4822.1−NRG3
CNVR2618.1−NRG2
CNVR1126.1−ERBB4
CNVR1129.1−ERBB4
CNVR4824.1−NRG3
YRI
CEU
CHB
0.0
0.2
0.4
0.6
0.8

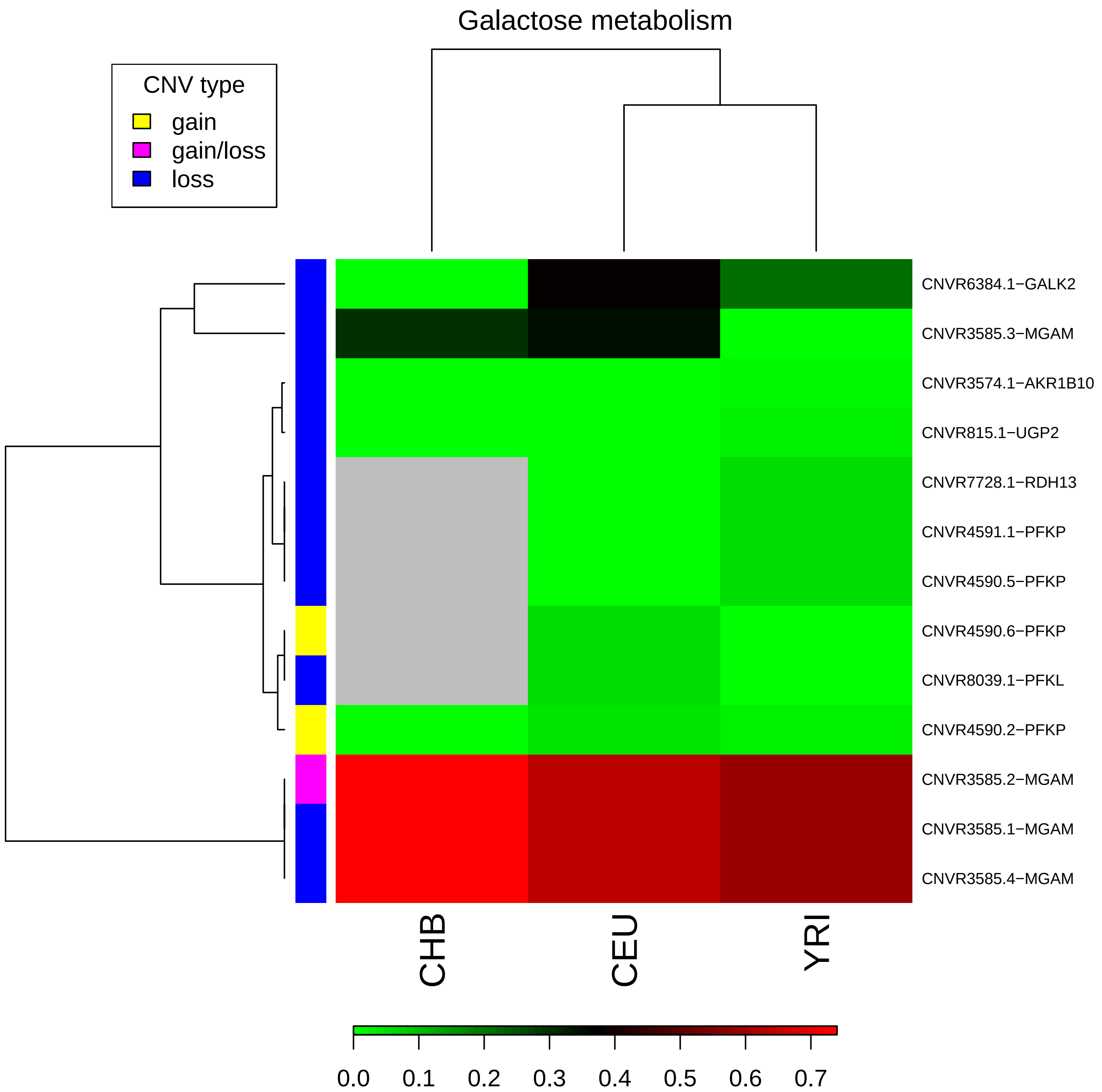

Galactose metabolism
CNV type
gain
gain/loss
loss
CNVR6384.1−GALK2
CNVR3585.3−MGAM
CNVR3574.1−AKR1B10
CNVR815.1−UGP2
CNVR7728.1−RDH13
CNVR4591.1−PFKP
CNVR4590.5−PFKP
CNVR4590.6−PFKP
CNVR8039.1−PFKL
CNVR4590.2−PFKP
CNVR3585.2−MGAM
CNVR3585.1−MGAM
CNVR3585.4−MGAM
CHB
CEU
YRI
0.0
0.1
0.2
0.3
0.4
0.5
0.6
0.7

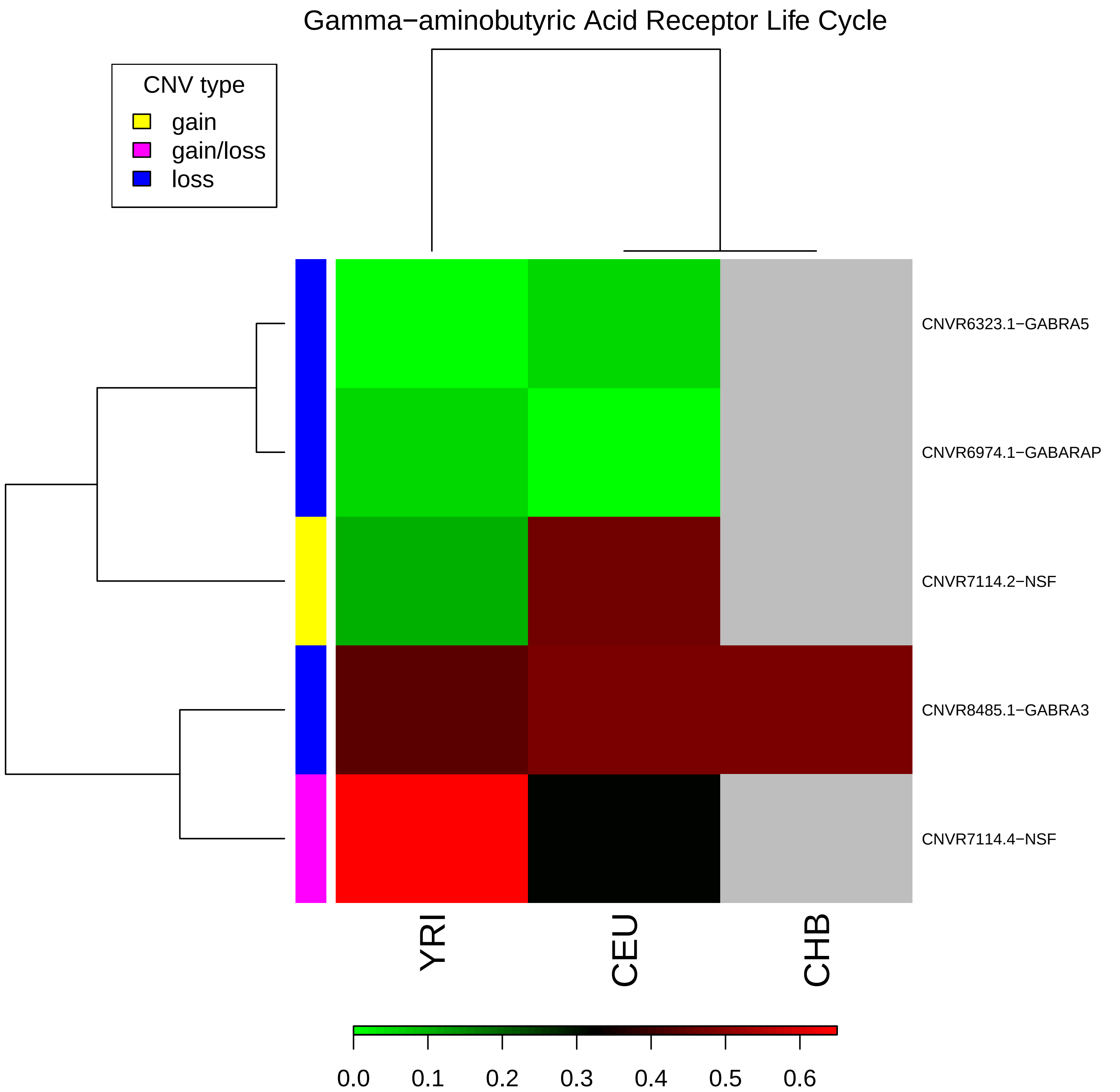

Gamma−aminobutyric Acid Receptor Life Cycle
CNV type
gain
gain/loss
loss
CNVR6323.1−GABRA5
CNVR6974.1−GABARAP
CNVR7114.2−NSF
CNVR8485.1−GABRA3
CNVR7114.4−NSF
YRI
CEU
CHB
0.0
0.1
0.2
0.3
0.4
0.5
0.6

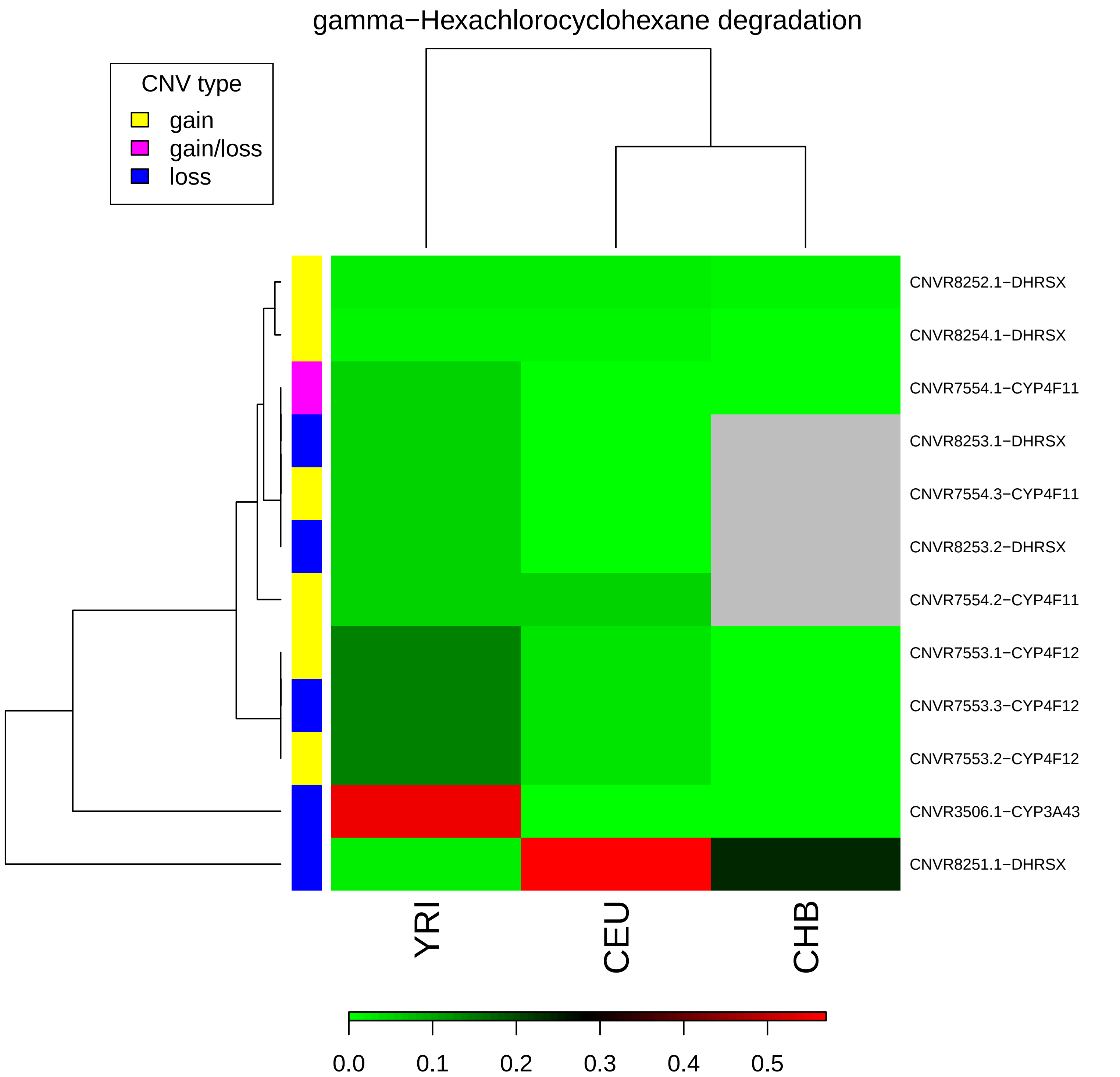
gamma−Hexachlorocyclohexane degradation
CNV type
gain
gain/loss
loss
CNVR8252.1−DHRSX
CNVR8254.1−DHRSX
CNVR7554.1−CYP4F11
CNVR8253.1−DHRSX
CNVR7554.3−CYP4F11
CNVR8253.2−DHRSX
CNVR7554.2−CYP4F11
CNVR7553.1−CYP4F12
CNVR7553.3−CYP4F12
CNVR7553.2−CYP4F12
CNVR3506.1−CYP3A43
CNVR8251.1−DHRSX
YRI
CEU
CHB
0.0
0.1
0.2
0.3
0.4
0.5

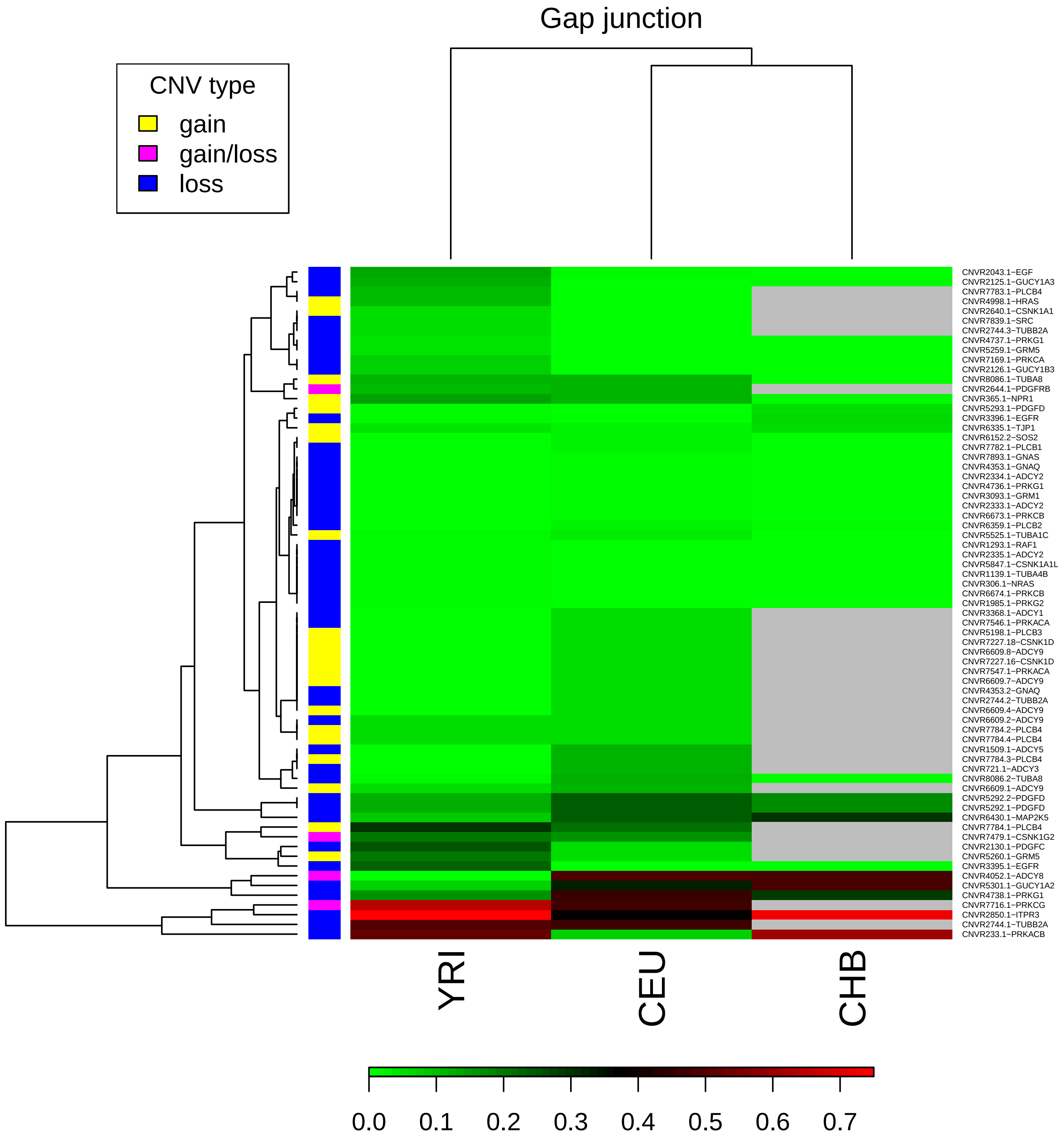

Gap junction
CNV type
gain
gain/loss
loss
CNVR2043.1−EGF
CNVR2125.1−GUCY1A3
CNVR7783.1−PLCB4
CNVR4998.1−HRAS
CNVR2640.1−CSNK1A1
CNVR7839.1−SRC
CNVR2744.3−TUBB2A
CNVR4737.1−PRKG1
CNVR5259.1−GRM5
CNVR7169.1−PRKCA
CNVR2126.1−GUCY1B3
CNVR8086.1−TUBA8
CNVR2644.1−PDGFRB
CNVR365.1−NPR1
CNVR5293.1−PDGFD
CNVR3396.1−EGFR
CNVR6335.1−TJP1
CNVR6152.2−SOS2
CNVR7782.1−PLCB1
CNVR7893.1−GNAS
CNVR4353.1−GNAQ
CNVR2334.1−ADCY2
CNVR4736.1−PRKG1
CNVR3093.1−GRM1
CNVR2333.1−ADCY2
CNVR6673.1−PRKCB
CNVR6359.1−PLCB2
CNVR5525.1−TUBA1C
CNVR1293.1−RAF1
CNVR2335.1−ADCY2
CNVR5847.1−CSNK1A1L
CNVR1139.1−TUBA4B
CNVR306.1−NRAS
CNVR6674.1−PRKCB
CNVR1985.1−PRKG2
CNVR3368.1−ADCY1
CNVR7546.1−PRKACA
CNVR5198.1−PLCB3
CNVR7227.18−CSNK1D
CNVR6609.8−ADCY9
CNVR7227.16−CSNK1D
CNVR7547.1−PRKACA
CNVR6609.7−ADCY9
CNVR4353.2−GNAQ
CNVR2744.2−TUBB2A
CNVR6609.4−ADCY9
CNVR6609.2−ADCY9
CNVR7784.2−PLCB4
CNVR7784.4−PLCB4
CNVR1509.1−ADCY5
CNVR7784.3−PLCB4
CNVR721.1−ADCY3
CNVR8086.2−TUBA8
CNVR6609.1−ADCY9
CNVR5292.2−PDGFD
CNVR5292.1−PDGFD
CNVR6430.1−MAP2K5
CNVR7784.1−PLCB4
CNVR7479.1−CSNK1G2
CNVR2130.1−PDGFC
CNVR5260.1−GRM5
CNVR3395.1−EGFR
CNVR4052.1−ADCY8
CNVR5301.1−GUCY1A2
CNVR4738.1−PRKG1
CNVR7716.1−PRKCG
CNVR2850.1−ITPR3
CNVR2744.1−TUBB2A
CNVR233.1−PRKACB
YRI
CEU
CHB
0.0
0.1
0.2
0.3
0.4
0.5
0.6
0.7

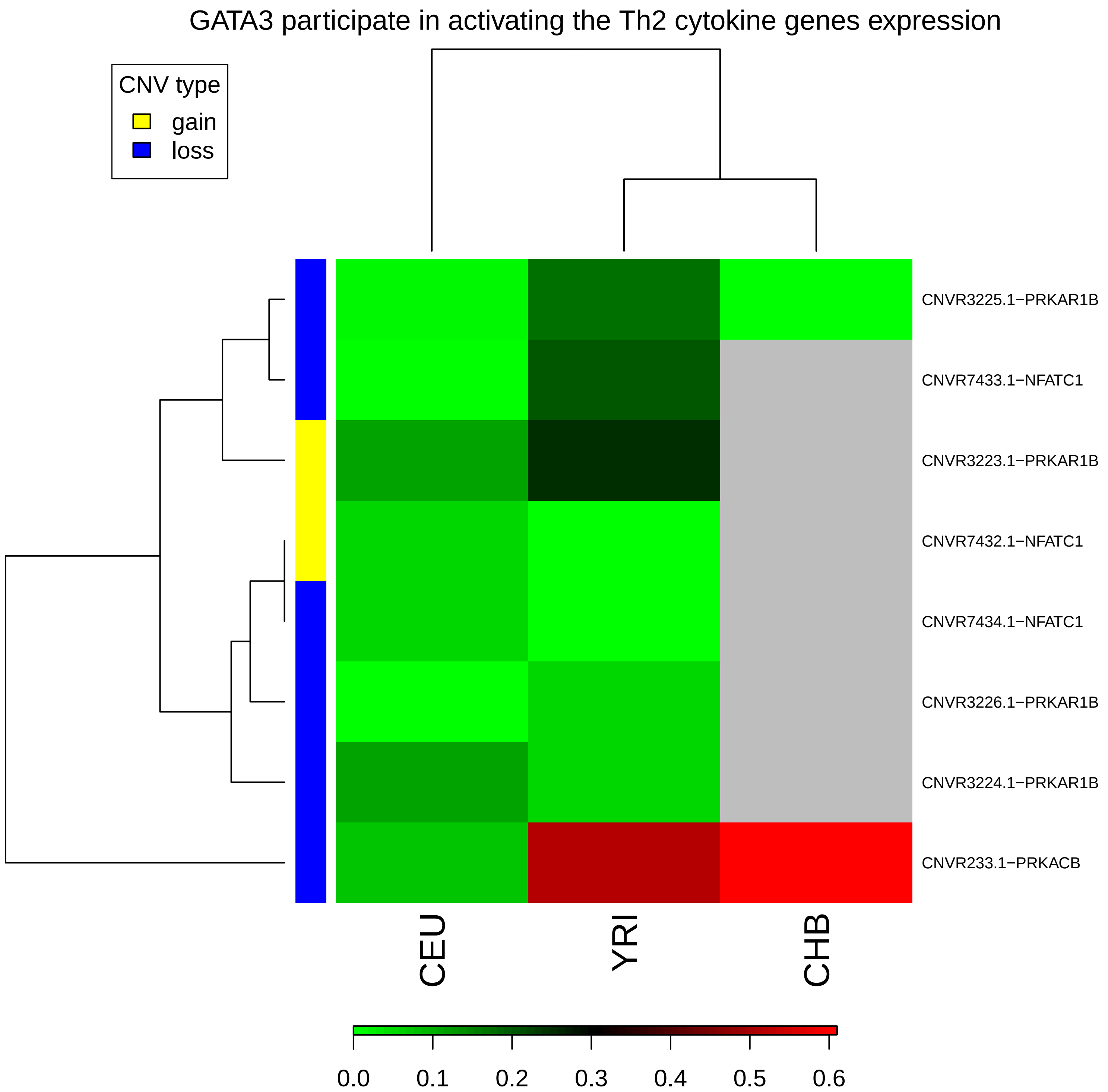
GATA3 participate in activating the Th2 cytokine genes expression
CNV type
gain
loss
CNVR3225.1−PRKAR1B
CNVR7433.1−NFATC1
CNVR3223.1−PRKAR1B
CNVR7432.1−NFATC1
CNVR7434.1−NFATC1
CNVR3226.1−PRKAR1B
CNVR3224.1−PRKAR1B
CNVR233.1−PRKACB
CEU
YRI
CHB
0.0
0.1
0.2
0.3
0.4
0.5
0.6

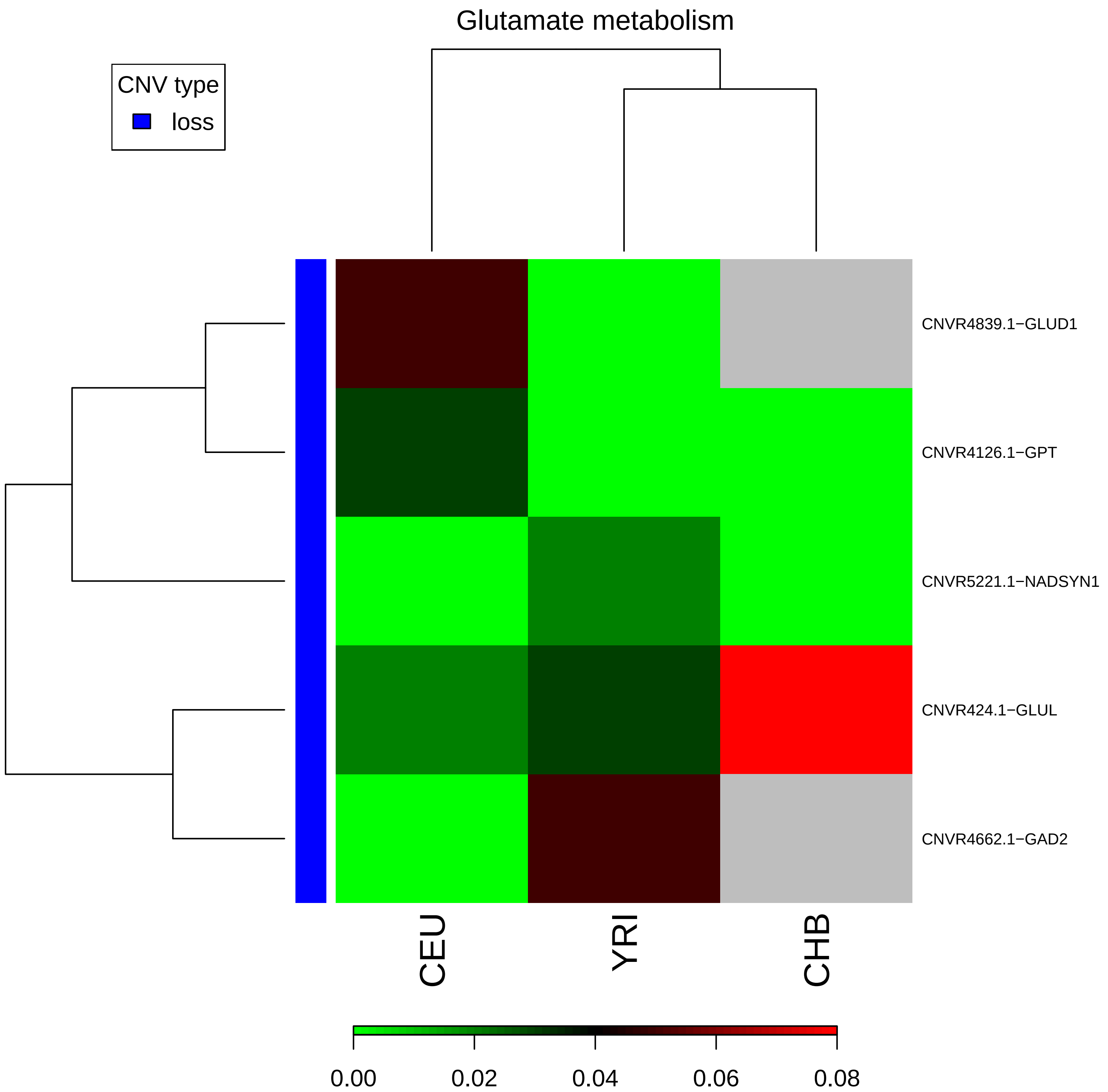

Glutamate metabolism
CNV type
loss
CNVR4839.1−GLUD1
CNVR4126.1−GPT
CNVR5221.1−NADSYN1
CNVR424.1−GLUL
CNVR4662.1−GAD2
CEU
YRI
CHB
0.00
0.02
0.04
0.06
0.08

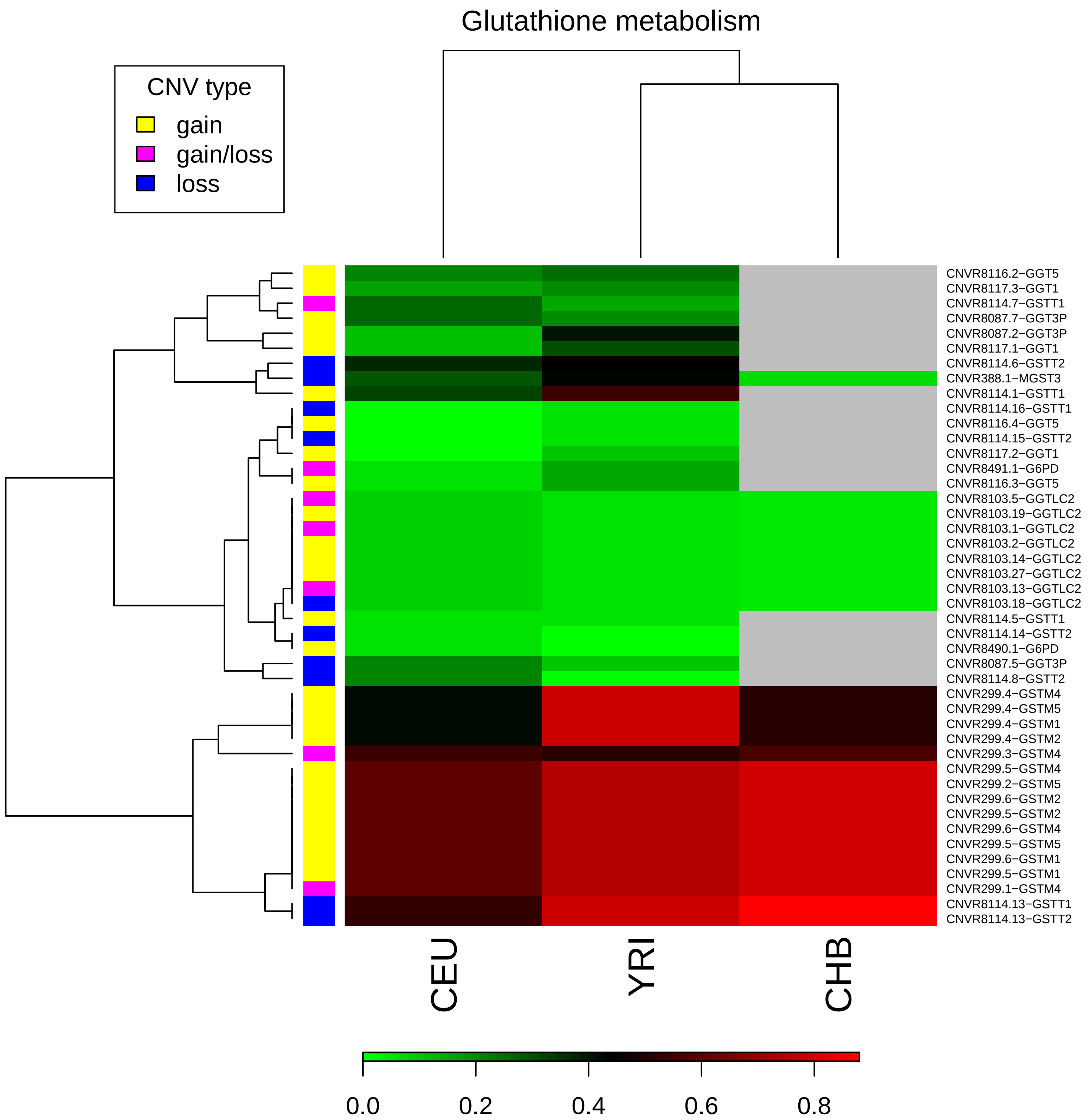

Glutathione metabolism
CNV type
gain
gain/loss
loss
CNVR8116.2−GGT5
CNVR8117.3−GGT1
CNVR8114.7−GSTT1
CNVR8087.7−GGT3P
CNVR8087.2−GGT3P
CNVR8117.1−GGT1
CNVR8114.6−GSTT2
CNVR388.1−MGST3
CNVR8114.1−GSTT1
CNVR8114.16−GSTT1
CNVR8116.4−GGT5
CNVR8114.15−GSTT2
CNVR8117.2−GGT1
CNVR8491.1−G6PD
CNVR8116.3−GGT5
CNVR8103.5−GGTLC2
CNVR8103.19−GGTLC2
CNVR8103.1−GGTLC2
CNVR8103.2−GGTLC2
CNVR8103.14−GGTLC2
CNVR8103.27−GGTLC2
CNVR8103.13−GGTLC2
CNVR8103.18−GGTLC2
CNVR8114.5−GSTT1
CNVR8114.14−GSTT2
CNVR8490.1−G6PD
CNVR8087.5−GGT3P
CNVR8114.8−GSTT2
CNVR299.4−GSTM4
CNVR299.4−GSTM5
CNVR299.4−GSTM1
CNVR299.4−GSTM2
CNVR299.3−GSTM4
CNVR299.5−GSTM4
CNVR299.2−GSTM5
CNVR299.6−GSTM2
CNVR299.5−GSTM2
CNVR299.6−GSTM4
CNVR299.5−GSTM5
CNVR299.6−GSTM1
CNVR299.5−GSTM1
CNVR299.1−GSTM4
CNVR8114.13−GSTT1
CNVR8114.13−GSTT2
CEU
YRI
CHB
0.0
0.2
0.4
0.6
0.8

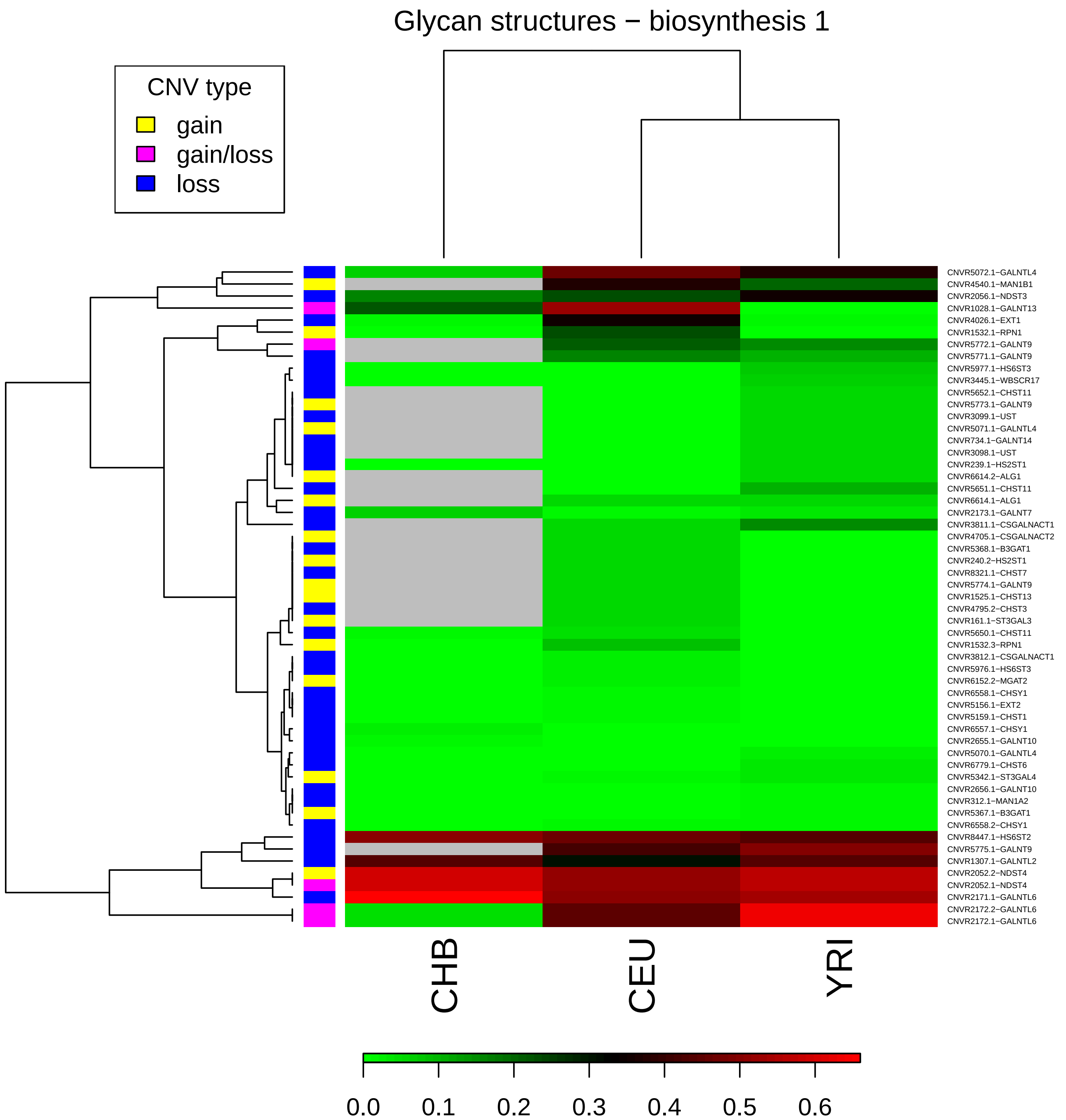

Glycan structures − biosynthesis 1
CNV type
gain
gain/loss
loss
CNVR5072.1−GALNTL4
CNVR4540.1−MAN1B1
CNVR2056.1−NDST3
CNVR1028.1−GALNT13
CNVR4026.1−EXT1
CNVR1532.1−RPN1
CNVR5772.1−GALNT9
CNVR5771.1−GALNT9
CNVR5977.1−HS6ST3
CNVR3445.1−WBSCR17
CNVR5652.1−CHST11
CNVR5773.1−GALNT9
CNVR3099.1−UST
CNVR5071.1−GALNTL4
CNVR734.1−GALNT14
CNVR3098.1−UST
CNVR239.1−HS2ST1
CNVR6614.2−ALG1
CNVR5651.1−CHST11
CNVR6614.1−ALG1
CNVR2173.1−GALNT7
CNVR3811.1−CSGALNACT1
CNVR4705.1−CSGALNACT2
CNVR5368.1−B3GAT1
CNVR240.2−HS2ST1
CNVR8321.1−CHST7
CNVR5774.1−GALNT9
CNVR1525.1−CHST13
CNVR4795.2−CHST3
CNVR161.1−ST3GAL3
CNVR5650.1−CHST11
CNVR1532.3−RPN1
CNVR3812.1−CSGALNACT1
CNVR5976.1−HS6ST3
CNVR6152.2−MGAT2
CNVR6558.1−CHSY1
CNVR5156.1−EXT2
CNVR5159.1−CHST1
CNVR6557.1−CHSY1
CNVR2655.1−GALNT10
CNVR5070.1−GALNTL4
CNVR6779.1−CHST6
CNVR5342.1−ST3GAL4
CNVR2656.1−GALNT10
CNVR312.1−MAN1A2
CNVR5367.1−B3GAT1
CNVR6558.2−CHSY1
CNVR8447.1−HS6ST2
CNVR5775.1−GALNT9
CNVR1307.1−GALNTL2
CNVR2052.2−NDST4
CNVR2052.1−NDST4
CNVR2171.1−GALNTL6
CNVR2172.2−GALNTL6
CNVR2172.1−GALNTL6
CHB
CEU
YRI
0.0
0.1
0.2
0.3
0.4
0.5
0.6

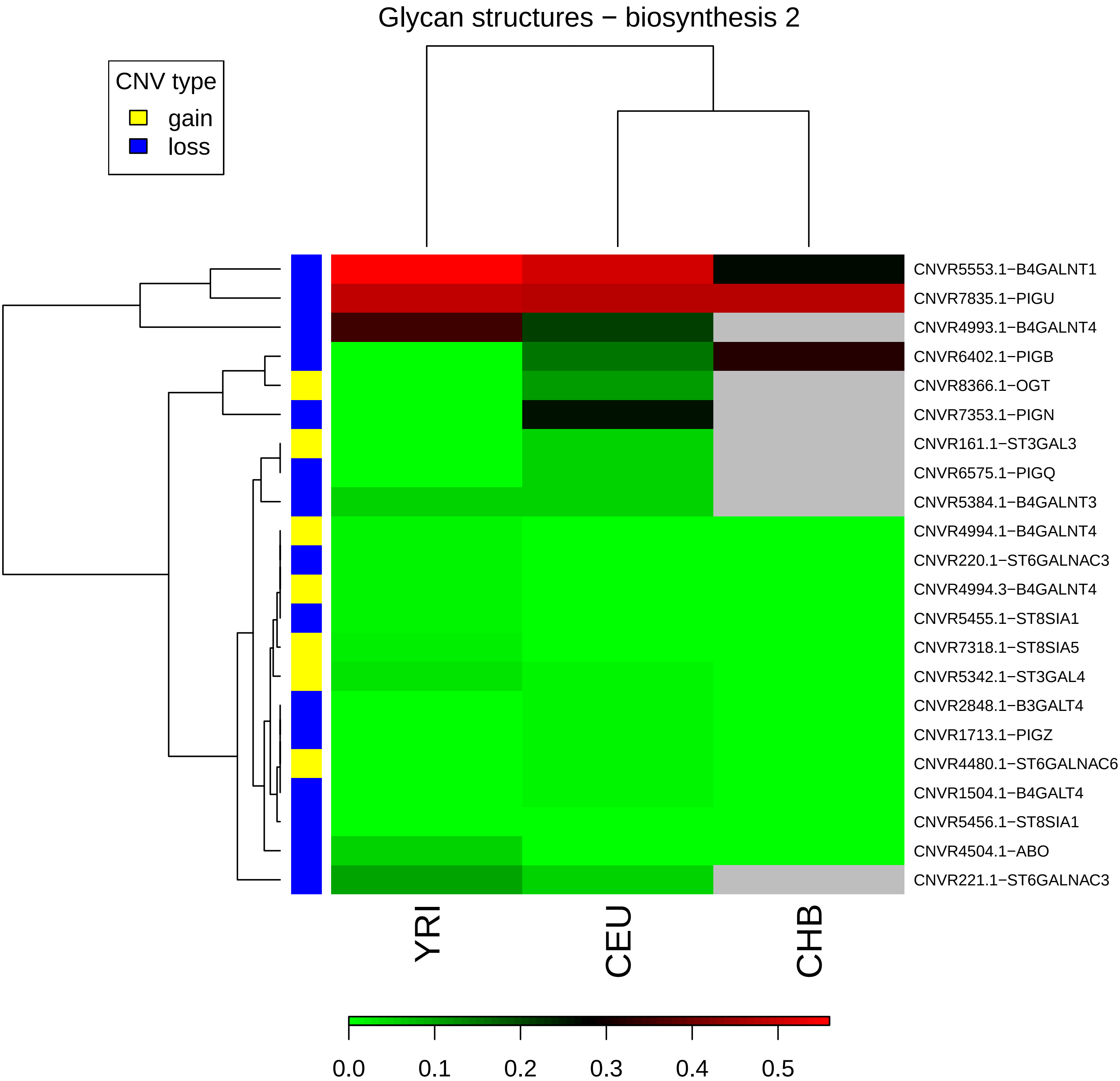
Glycan structures − biosynthesis 2
CNV type
gain
loss
CNVR5553.1−B4GALNT1
CNVR7835.1−PIGU
CNVR4993.1−B4GALNT4
CNVR6402.1−PIGB
CNVR8366.1−OGT
CNVR7353.1−PIGN
CNVR161.1−ST3GAL3
CNVR6575.1−PIGQ
CNVR5384.1−B4GALNT3
CNVR4994.1−B4GALNT4
CNVR220.1−ST6GALNAC3
CNVR4994.3−B4GALNT4
CNVR5455.1−ST8SIA1
CNVR7318.1−ST8SIA5
CNVR5342.1−ST3GAL4
CNVR2848.1−B3GALT4
CNVR1713.1−PIGZ
CNVR4480.1−ST6GALNAC6
CNVR1504.1−B4GALT4
CNVR5456.1−ST8SIA1
CNVR4504.1−ABO
CNVR221.1−ST6GALNAC3
YRI
CEU
CHB
0.0
0.1
0.2
0.3
0.4
0.5

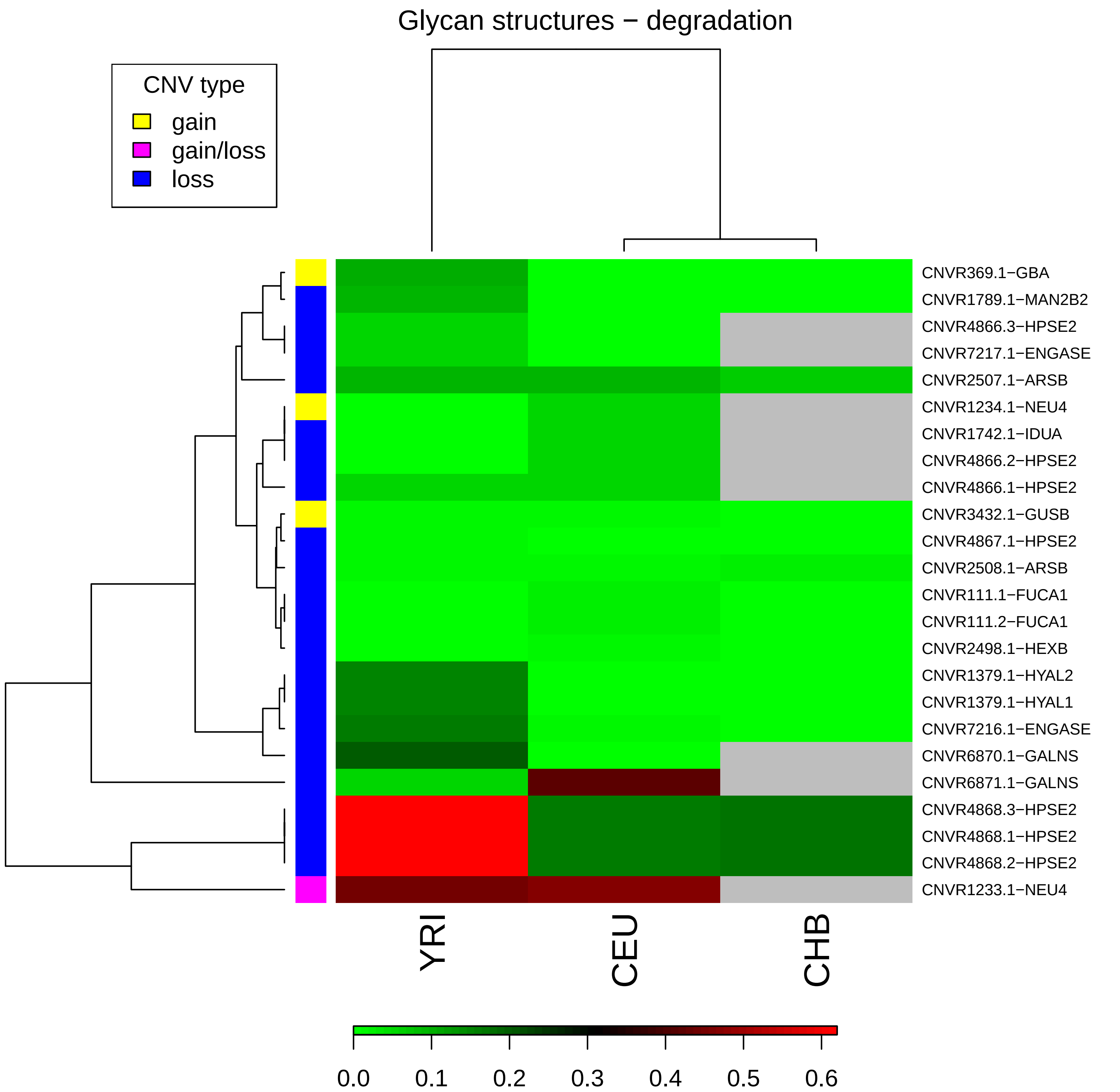

Glycan structures − degradation
CNV type
gain
gain/loss
loss
CNVR369.1−GBA
CNVR1789.1−MAN2B2
CNVR4866.3−HPSE2
CNVR7217.1−ENGASE
CNVR2507.1−ARSB
CNVR1234.1−NEU4
CNVR1742.1−IDUA
CNVR4866.2−HPSE2
CNVR4866.1−HPSE2
CNVR3432.1−GUSB
CNVR4867.1−HPSE2
CNVR2508.1−ARSB
CNVR111.1−FUCA1
CNVR111.2−FUCA1
CNVR2498.1−HEXB
CNVR1379.1−HYAL2
CNVR1379.1−HYAL1
CNVR7216.1−ENGASE
CNVR6870.1−GALNS
CNVR6871.1−GALNS
CNVR4868.3−HPSE2
CNVR4868.1−HPSE2
CNVR4868.2−HPSE2
CNVR1233.1−NEU4
YRI
CEU
CHB
0.0
0.1
0.2
0.3
0.4
0.5
0.6

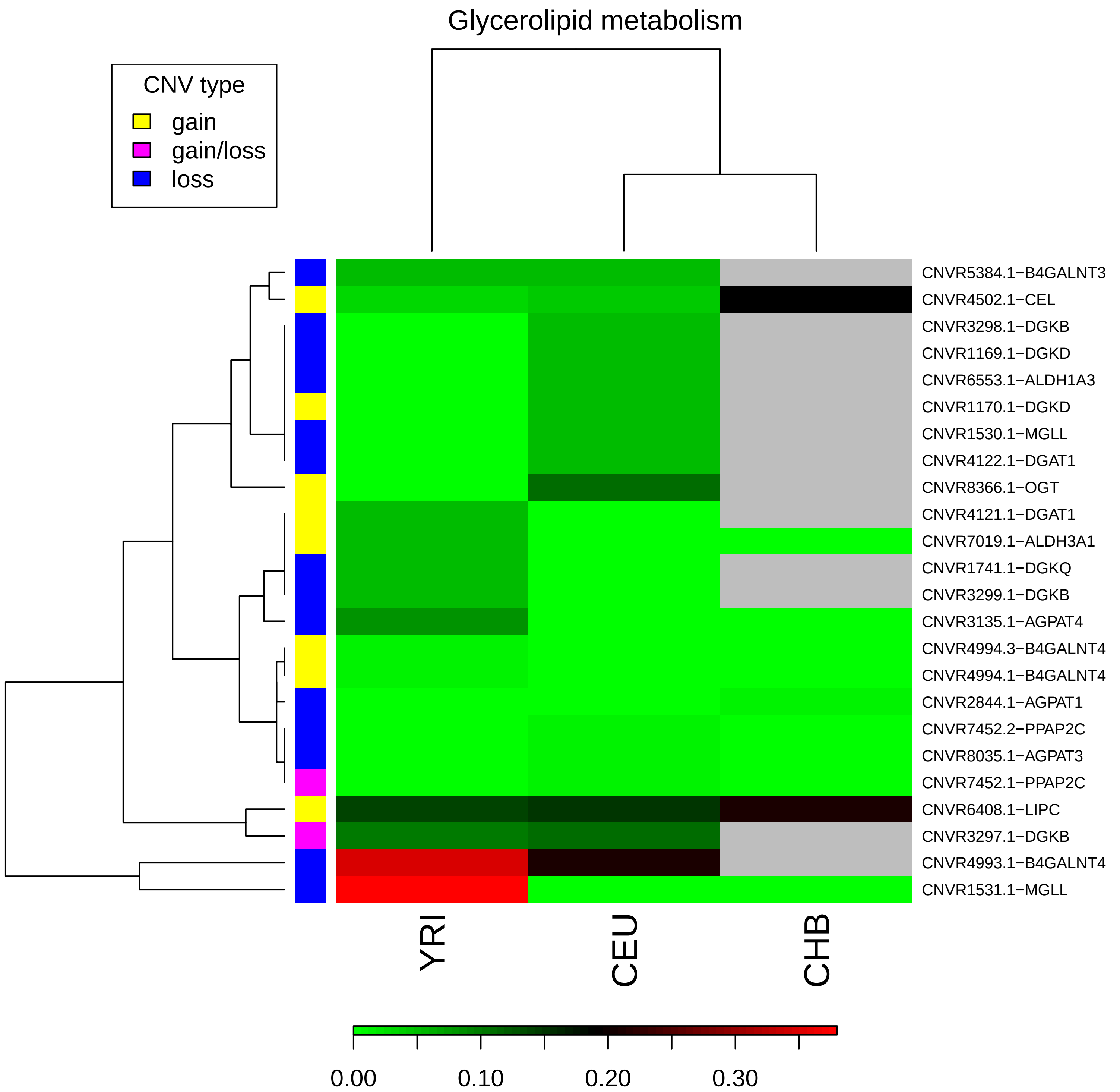
Glycerolipid metabolism
CNV type
gain
gain/loss
loss
CNVR5384.1−B4GALNT3
CNVR4502.1−CEL
CNVR3298.1−DGKB
CNVR1169.1−DGKD
CNVR6553.1−ALDH1A3
CNVR1170.1−DGKD
CNVR1530.1−MGLL
CNVR4122.1−DGAT1
CNVR8366.1−OGT
CNVR4121.1−DGAT1
CNVR7019.1−ALDH3A1
CNVR1741.1−DGKQ
CNVR3299.1−DGKB
CNVR3135.1−AGPAT4
CNVR4994.3−B4GALNT4
CNVR4994.1−B4GALNT4
CNVR2844.1−AGPAT1
CNVR7452.2−PPAP2C
CNVR8035.1−AGPAT3
CNVR7452.1−PPAP2C
CNVR6408.1−LIPC
CNVR3297.1−DGKB
CNVR4993.1−B4GALNT4
CNVR1531.1−MGLL
YRI
CEU
CHB
0.00
0.10
0.20
0.30

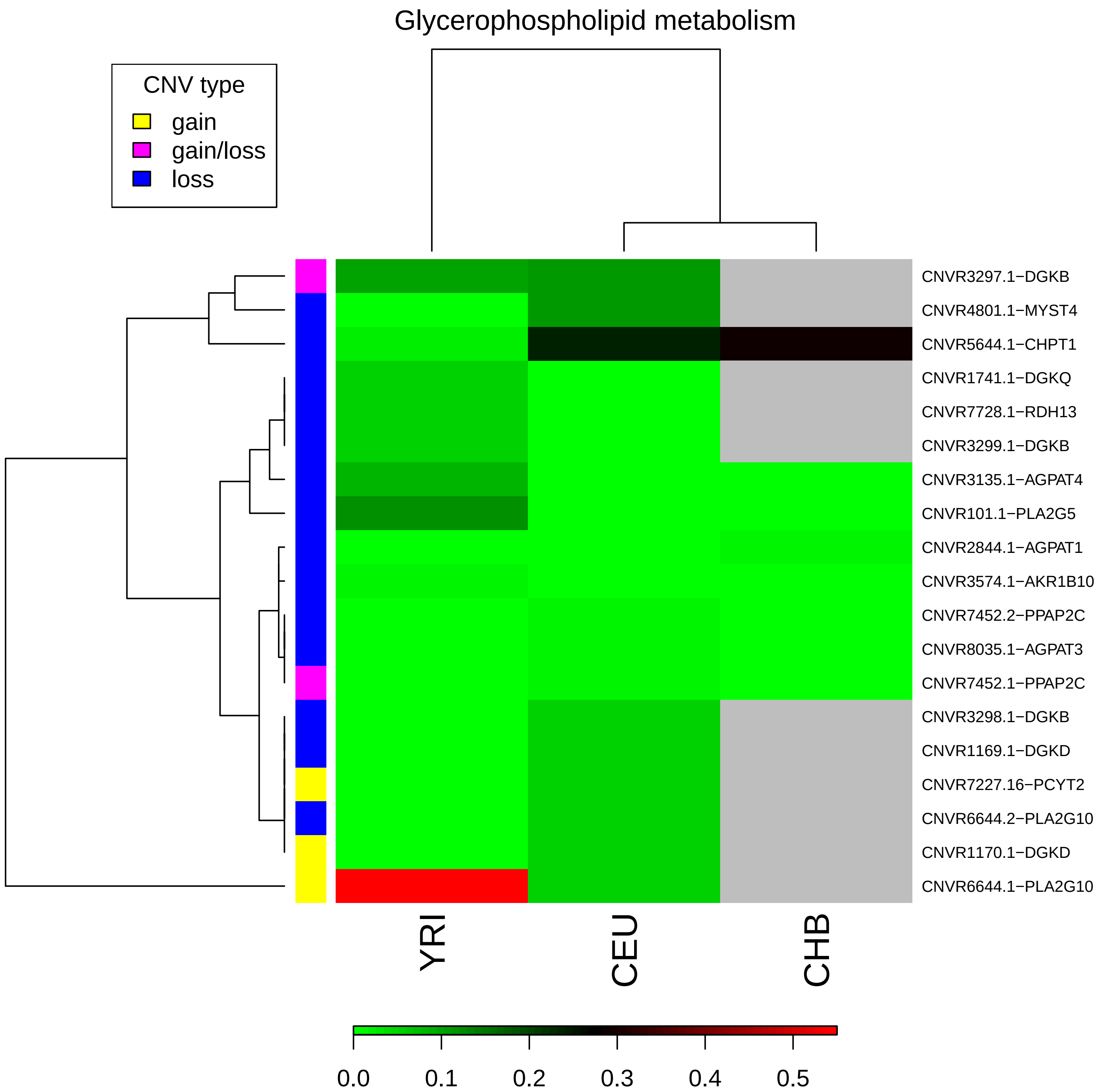
Glycerophospholipid metabolism
CNV type
gain
gain/loss
loss
CNVR3297.1−DGKB
CNVR4801.1−MYST4
CNVR5644.1−CHPT1
CNVR1741.1−DGKQ
CNVR7728.1−RDH13
CNVR3299.1−DGKB
CNVR3135.1−AGPAT4
CNVR101.1−PLA2G5
CNVR2844.1−AGPAT1
CNVR3574.1−AKR1B10
CNVR7452.2−PPAP2C
CNVR8035.1−AGPAT3
CNVR7452.1−PPAP2C
CNVR3298.1−DGKB
CNVR1169.1−DGKD
CNVR7227.16−PCYT2
CNVR6644.2−PLA2G10
CNVR1170.1−DGKD
CNVR6644.1−PLA2G10
YRI
CEU
CHB
0.0
0.1
0.2
0.3
0.4
0.5

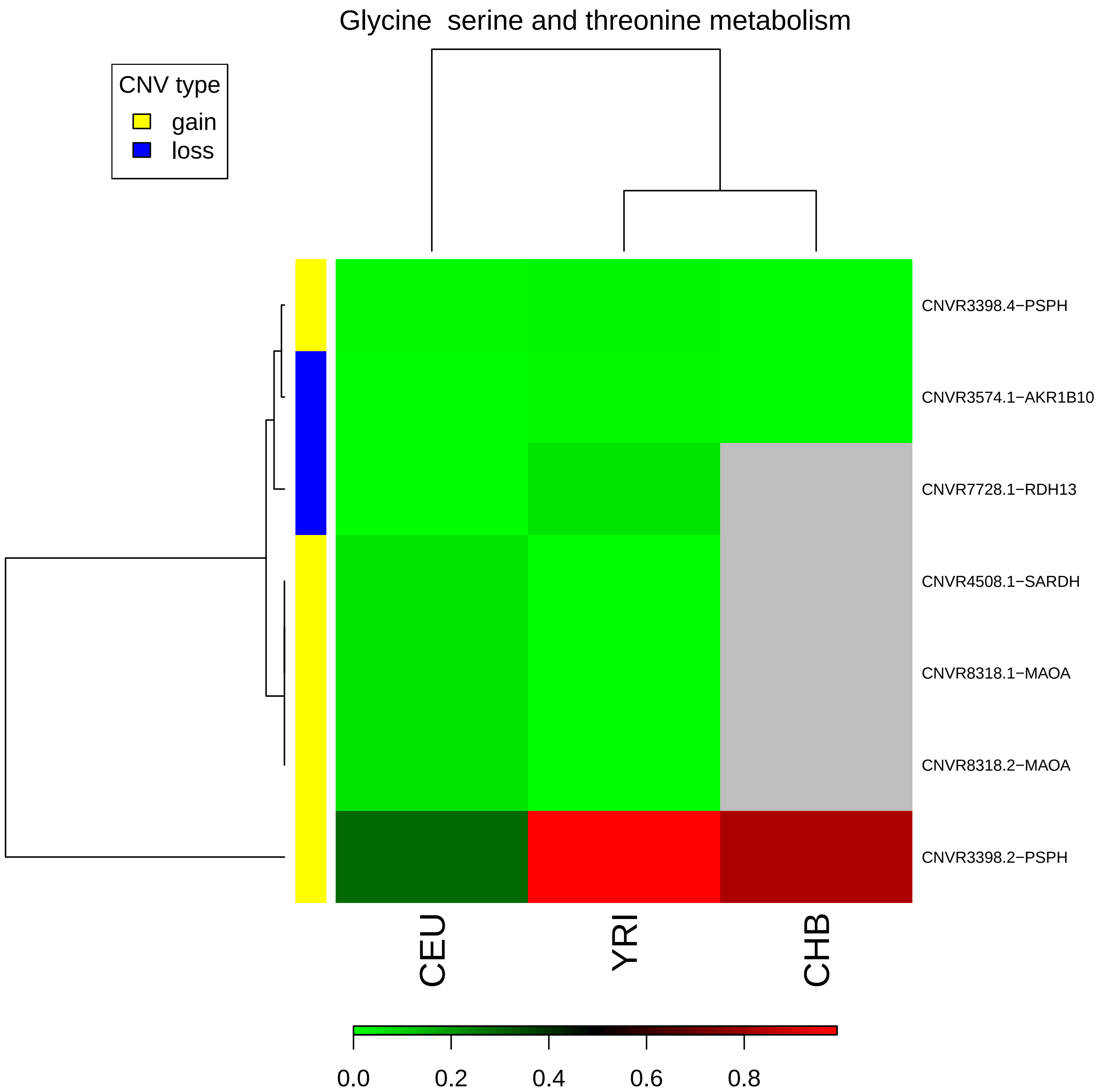

Glycine serine and threonine metabolism
CNV type
gain
loss
CNVR3398.4−PSPH
CNVR3574.1−AKR1B10
CNVR7728.1−RDH13
CNVR4508.1−SARDH
CNVR8318.1−MAOA
CNVR8318.2−MAOA
CNVR3398.2−PSPH
CEU
YRI
CHB
0.0
0.2
0.4
0.6
0.8

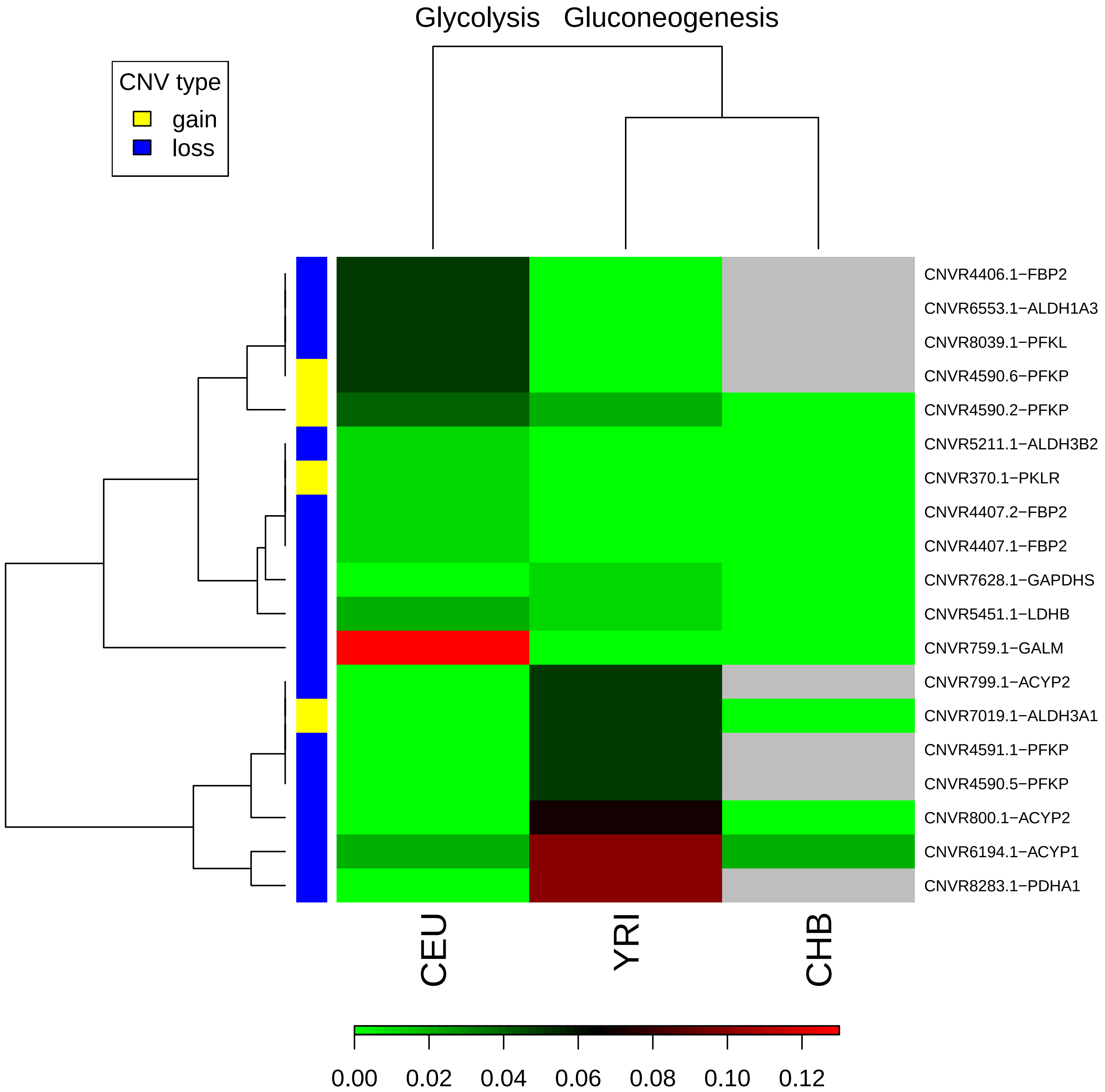

Glycolysis Gluconeogenesis
CNV type
gain
loss
CNVR4406.1−FBP2
CNVR6553.1−ALDH1A3
CNVR8039.1−PFKL
CNVR4590.6−PFKP
CNVR4590.2−PFKP
CNVR5211.1−ALDH3B2
CNVR370.1−PKLR
CNVR4407.2−FBP2
CNVR4407.1−FBP2
CNVR7628.1−GAPDHS
CNVR5451.1−LDHB
CNVR759.1−GALM
CNVR799.1−ACYP2
CNVR7019.1−ALDH3A1
CNVR4591.1−PFKP
CNVR4590.5−PFKP
CNVR800.1−ACYP2
CNVR6194.1−ACYP1
CNVR8283.1−PDHA1
CEU
YRI
CHB
0.00
0.02
0.04
0.06
0.08
0.10
0.12

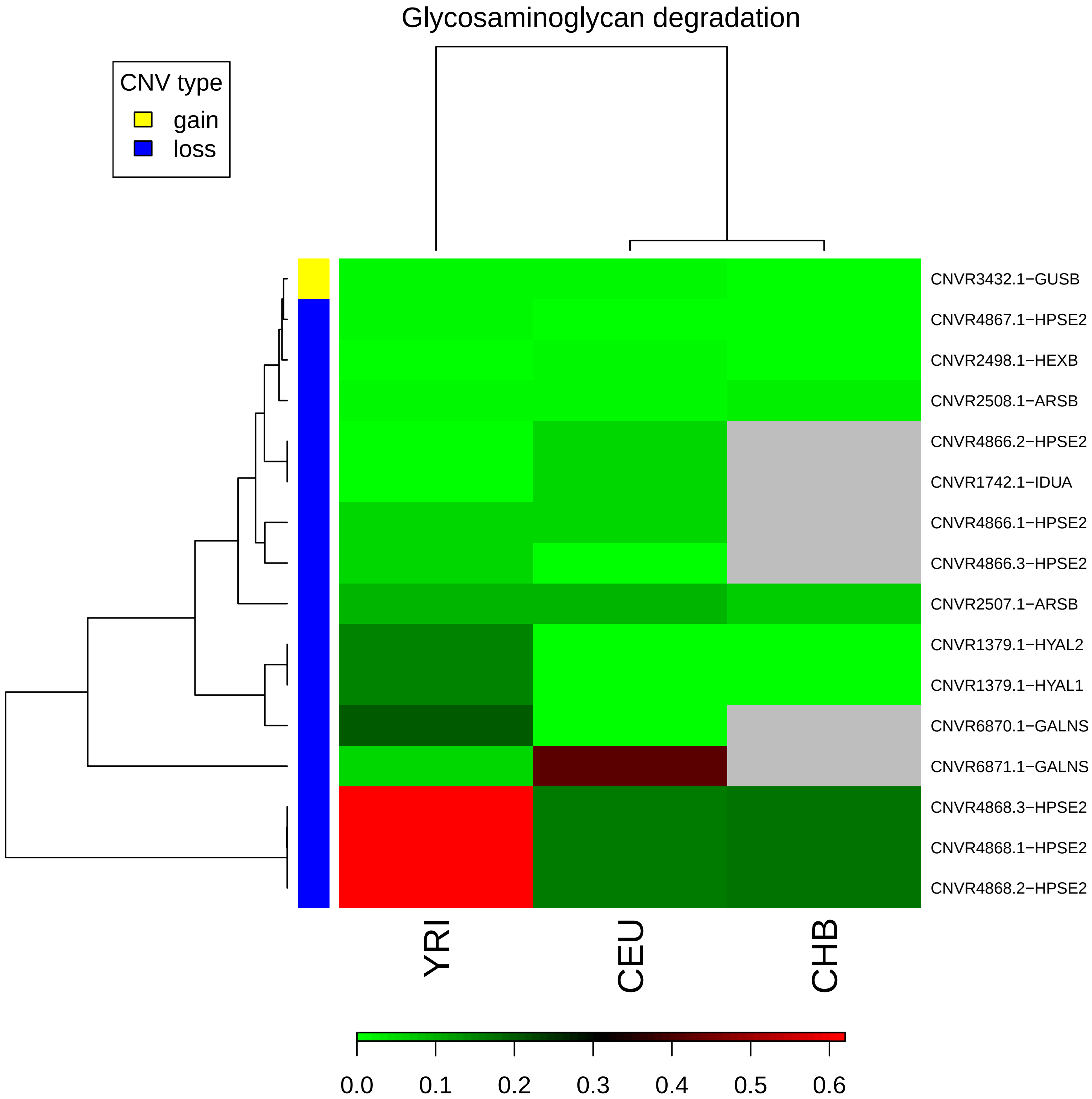
Glycosaminoglycan degradation
CNV type
gain
loss
CNVR3432.1−GUSB
CNVR4867.1−HPSE2
CNVR2498.1−HEXB
CNVR2508.1−ARSB
CNVR4866.2−HPSE2
CNVR1742.1−IDUA
CNVR4866.1−HPSE2
CNVR4866.3−HPSE2
CNVR2507.1−ARSB
CNVR1379.1−HYAL2
CNVR1379.1−HYAL1
CNVR6870.1−GALNS
CNVR6871.1−GALNS
CNVR4868.3−HPSE2
CNVR4868.1−HPSE2
CNVR4868.2−HPSE2
YRI
CEU
CHB
0.0
0.1
0.2
0.3
0.4
0.5
0.6

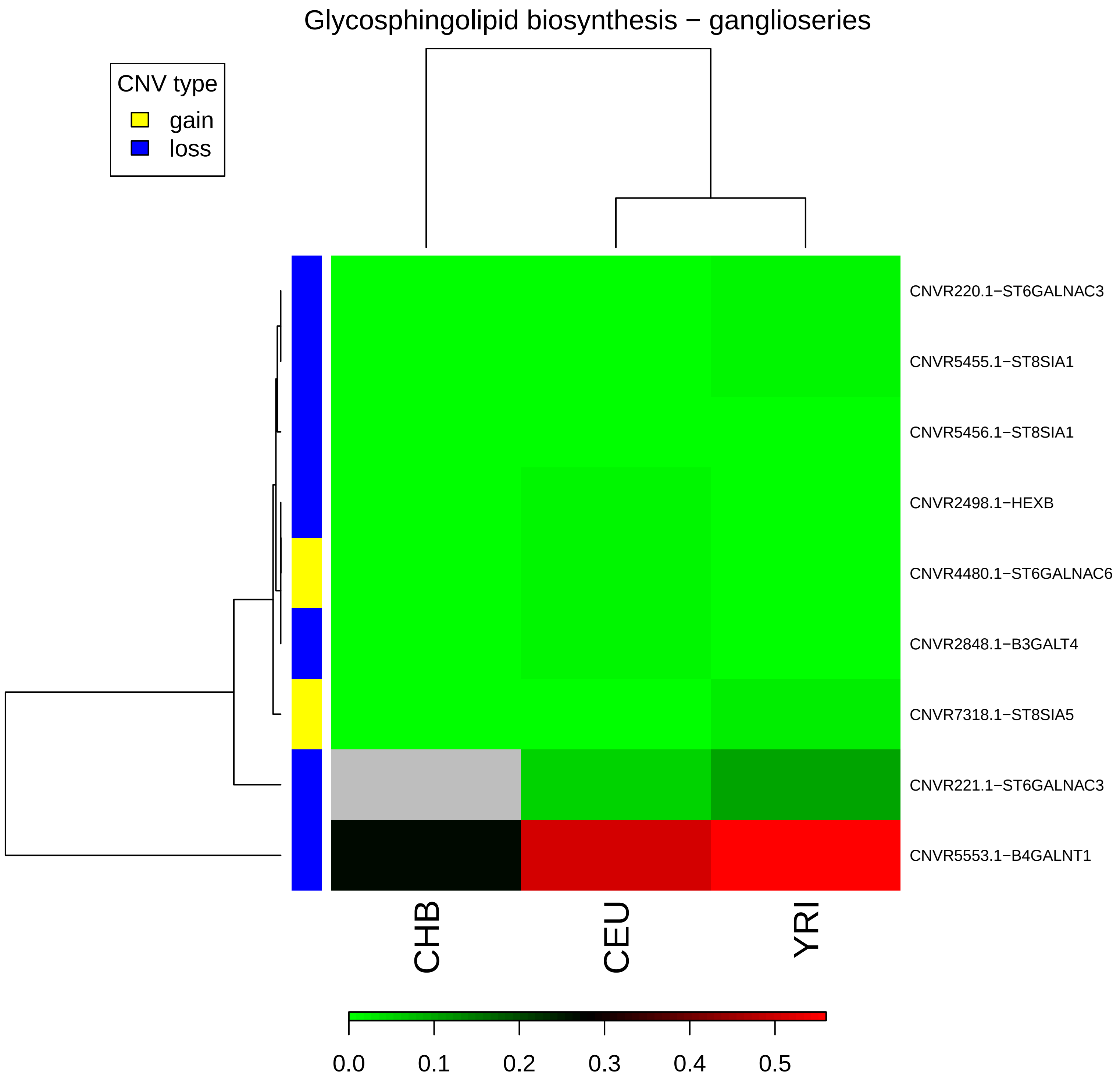
Glycosphingolipid biosynthesis − ganglioseries
CNV type
gain
loss
CNVR220.1−ST6GALNAC3
CNVR5455.1−ST8SIA1
CNVR5456.1−ST8SIA1
CNVR2498.1−HEXB
CNVR4480.1−ST6GALNAC6
CNVR2848.1−B3GALT4
CNVR7318.1−ST8SIA5
CNVR221.1−ST6GALNAC3
CNVR5553.1−B4GALNT1
CHB
CEU
YRI
0.0
0.1
0.2
0.3
0.4
0.5

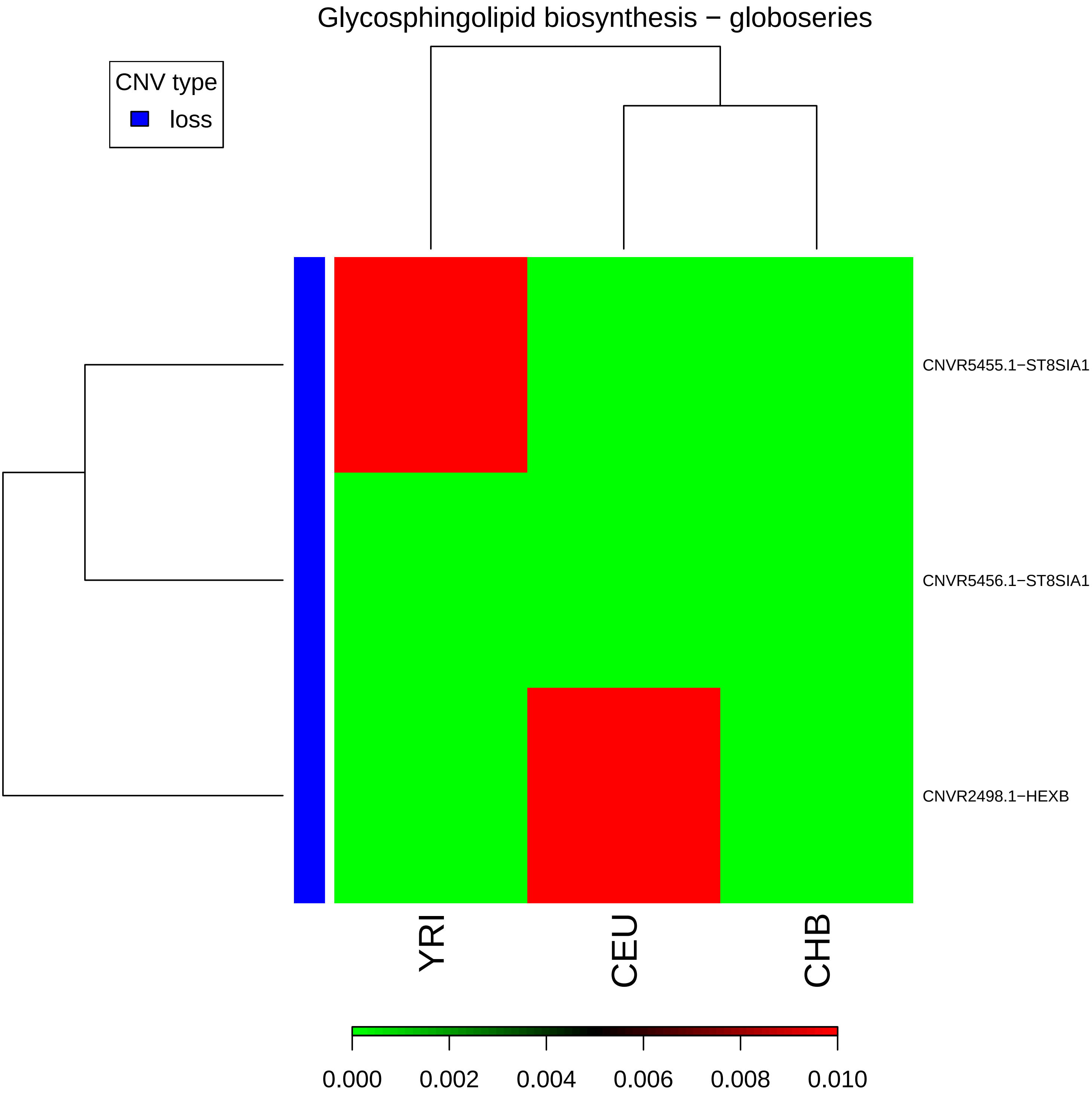

Glycosphingolipid biosynthesis − globoseries
CNV type
loss
CNVR5455.1−ST8SIA1
CNVR5456.1−ST8SIA1
CNVR2498.1−HEXB
YRI
CEU
CHB
0.000
0.002
0.004
0.006
0.008
0.010

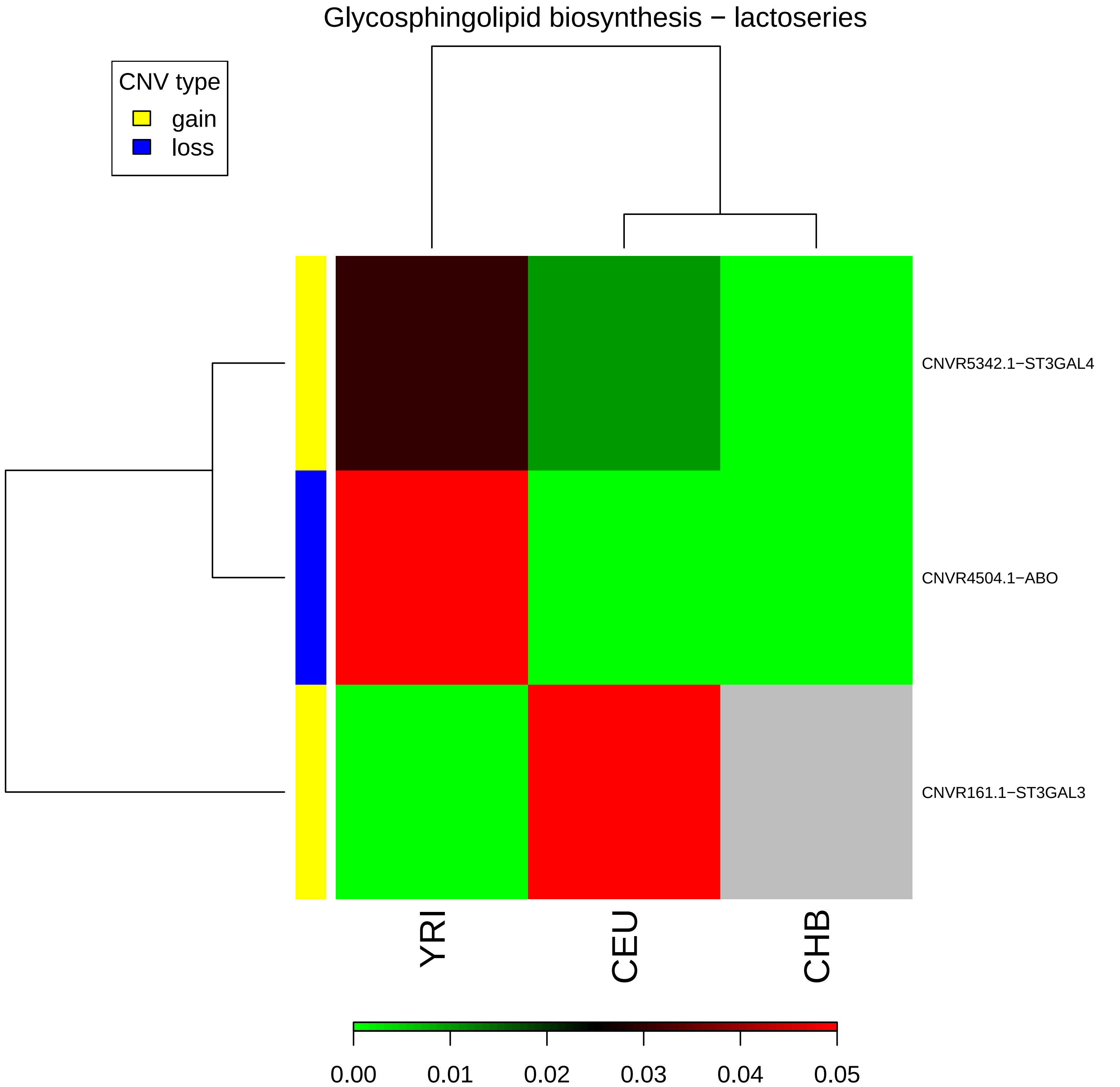
Glycosphingolipid biosynthesis − lactoseries
CNV type
gain
loss
CNVR5342.1−ST3GAL4
CNVR4504.1−ABO
CNVR161.1−ST3GAL3
YRI
CEU
CHB
0.00
0.01
0.02
0.03
0.04
0.05

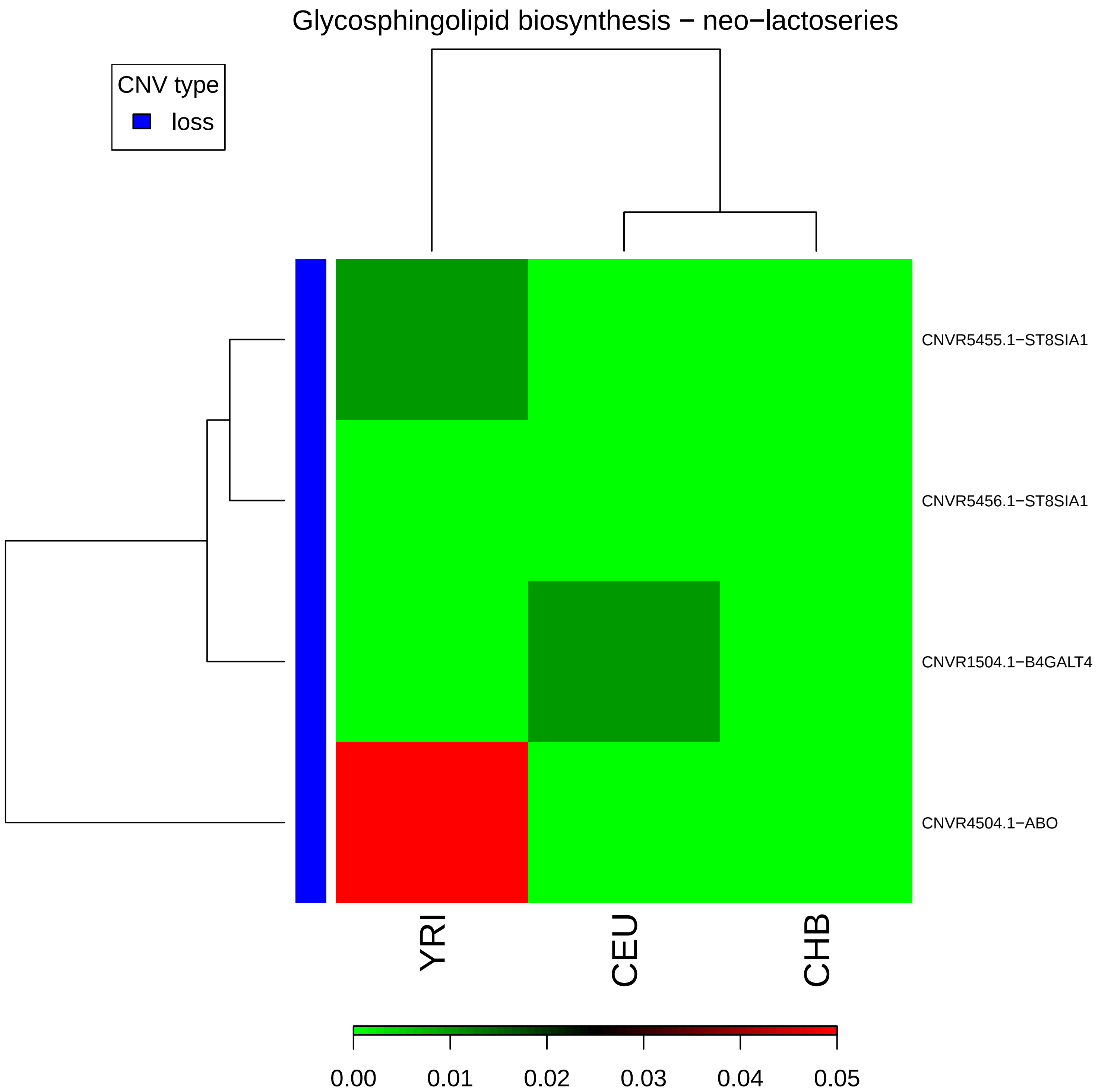
Glycosphingolipid biosynthesis − neo−lactoseries
CNV type
loss
CNVR5455.1−ST8SIA1
CNVR5456.1−ST8SIA1
CNVR1504.1−B4GALT4
CNVR4504.1−ABO
YRI
CEU
CHB
0.00
0.01
0.02
0.03
0.04
0.05

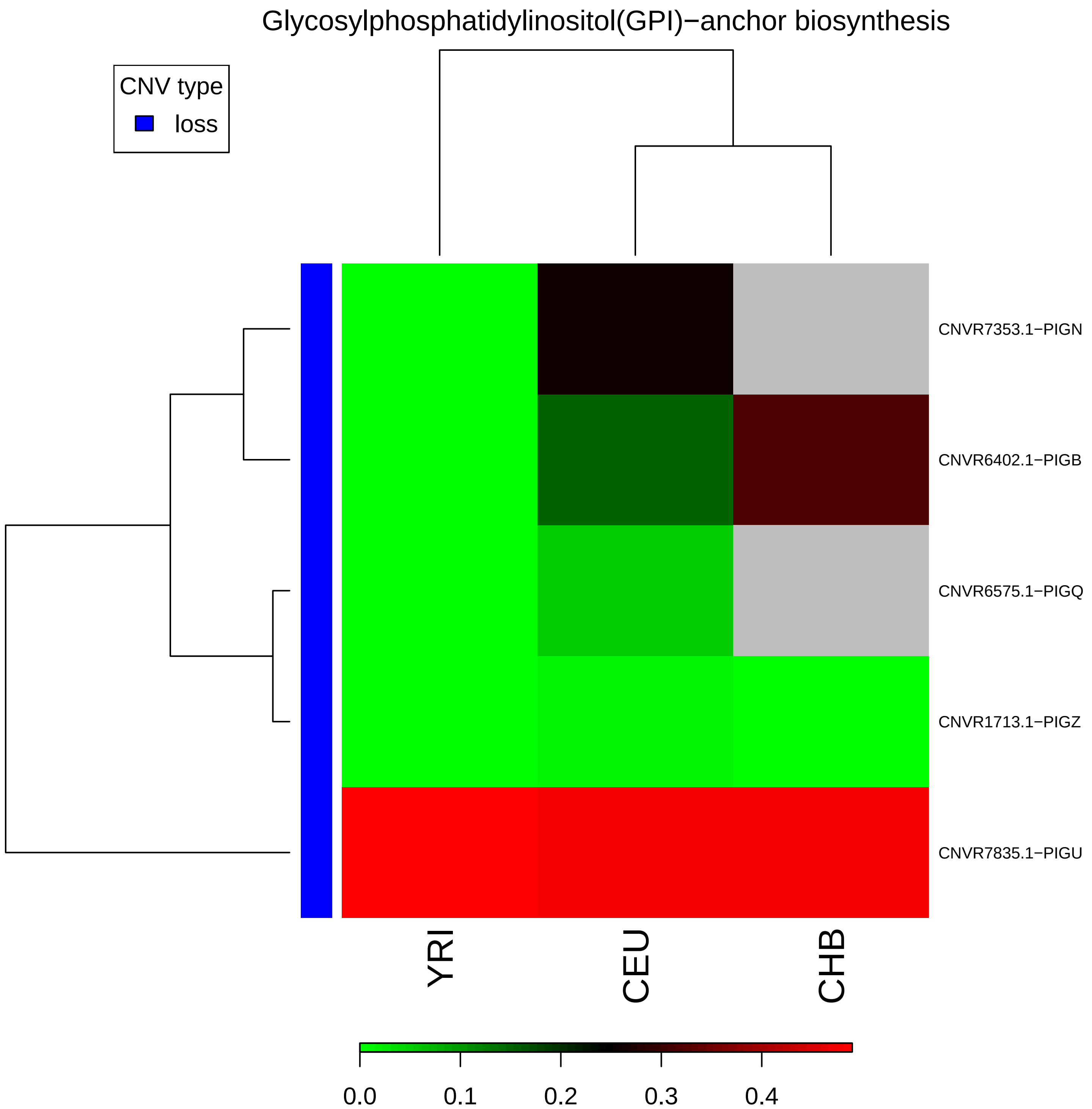

Glycosylphosphatidylinositol(GPI)−anchor biosynthesis
CNV type
loss
CNVR7353.1−PIGN
CNVR6402.1−PIGB
CNVR6575.1−PIGQ
CNVR1713.1−PIGZ
CNVR7835.1−PIGU
YRI
CEU
CHB
0.0
0.1
0.2
0.3
0.4

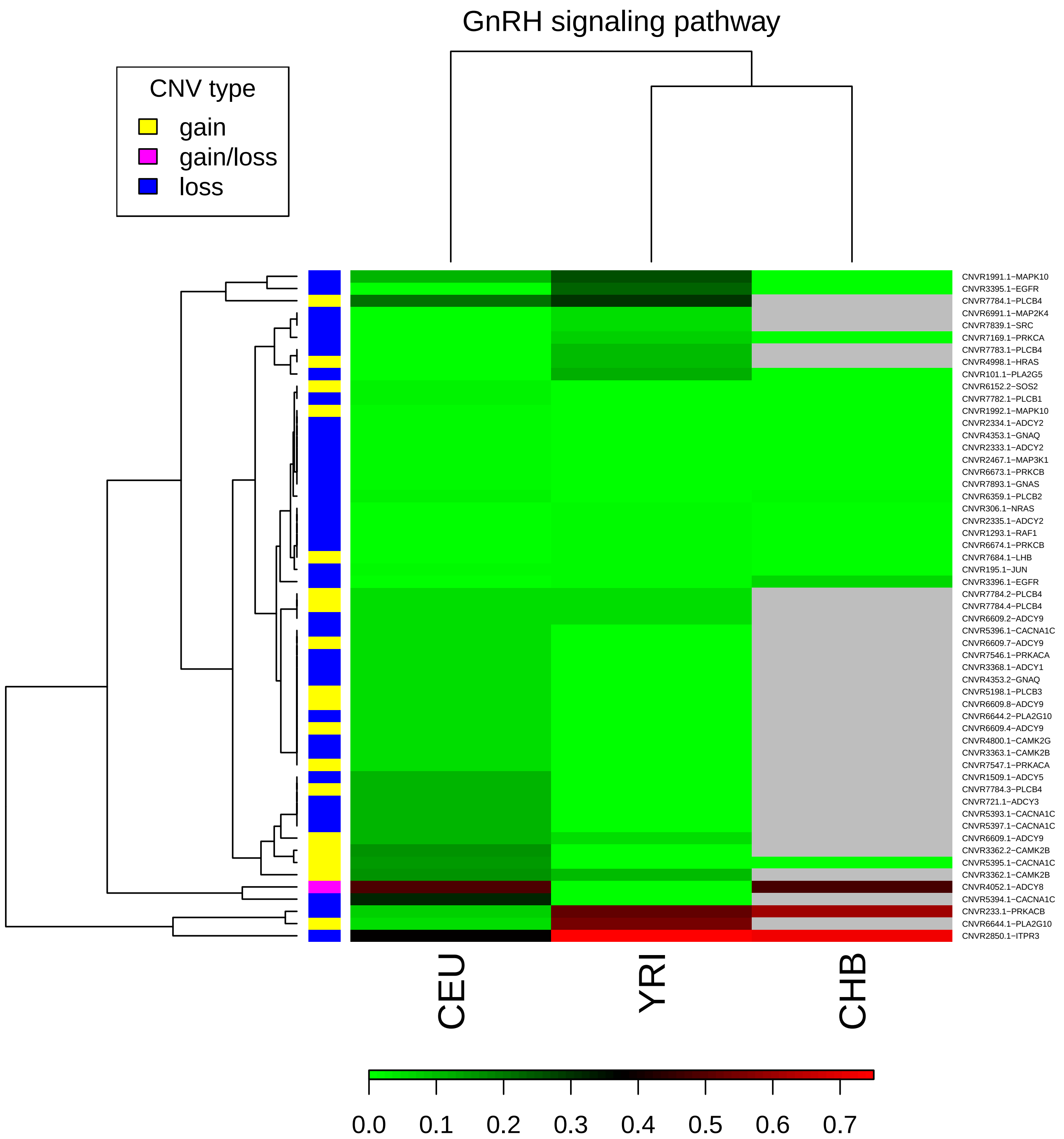

GnRH signaling pathway
CNV type
gain
gain/loss
loss
CNVR1991.1–MAPK10
CNVR3395.1–EGFR
CNVR7784.1–PLCB4
CNVR6991.1–MAP2K4
CNVR7839.1–SRC
CNVR7169.1–PRKCA
CNVR7783.1–PLCB4
CNVR4998.1–HRAS
CNVR101.1–PLA2G5
CNVR6152.2–SOS2
CNVR7782.1–PLCB1
CNVR1992.1–MAPK10
CNVR2334.1–ADCY2
CNVR4353.1–GNAQ
CNVR2333.1–ADCY2
CNVR2467.1–MAP3K1
CNVR6673.1–PRKCB
CNVR7893.1–GNAS
CNVR6359.1–PLCB2
CNVR306.1–NRAS
CNVR2335.1–ADCY2
CNVR1293.1–RAF1
CNVR6674.1–PRKCB
CNVR7684.1–LHB
CNVR195.1–JUN
CNVR3396.1–EGFR
CNVR7784.2–PLCB4
CNVR7784.4–PLCB4
CNVR6609.2–ADCY9
CNVR5396.1–CACNA1C
CNVR6609.7–ADCY9
CNVR7546.1–PRKACA
CNVR3368.1–ADCY1
CNVR4353.2–GNAQ
CNVR5198.1–PLCB3
CNVR6609.8–ADCY9
CNVR6644.2–PLA2G10
CNVR6609.4–ADCY9
CNVR4800.1–CAMK2G
CNVR3363.1–CAMK2B
CNVR7547.1–PRKACA
CNVR1509.1–ADCY5
CNVR7784.3–PLCB4
CNVR721.1–ADCY3
CNVR5393.1–CACNA1C
CNVR5397.1–CACNA1C
CNVR6609.1–ADCY9
CNVR3362.2–CAMK2B
CNVR5395.1–CACNA1C
CNVR3362.1–CAMK2B
CNVR4052.1–ADCY8
CNVR5394.1–CACNA1C
CNVR233.1–PRKACB
CNVR6644.1–PLA2G10
CNVR2850.1–ITPR3
CEU
YRI
CHB
0.0
0.1
0.2
0.3
0.4
0.5
0.6
0.7

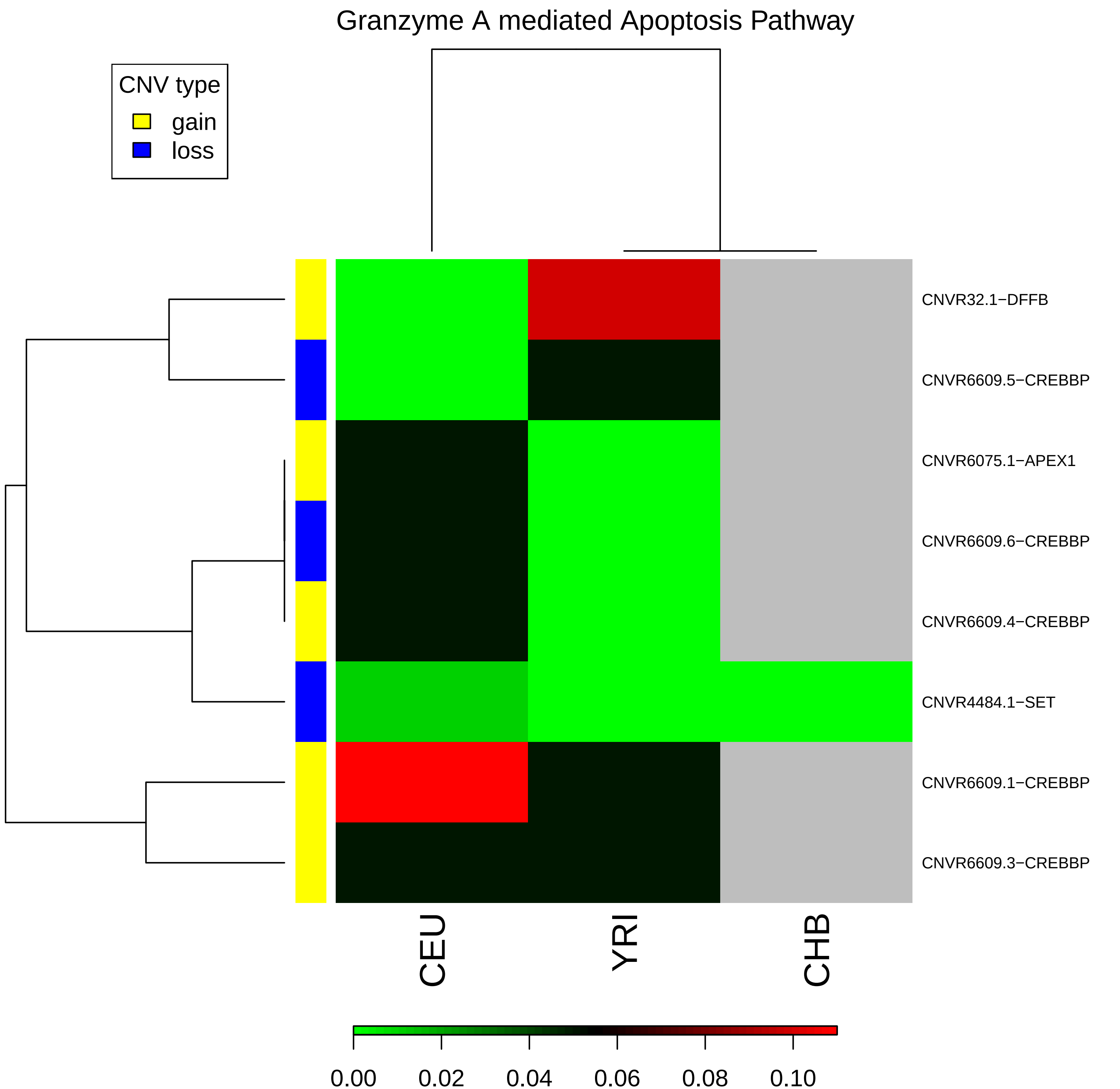

Granzyme A mediated Apoptosis Pathway
CNV type
gain
loss
CNVR32.1−DFFB
CNVR6609.5−CREBBP
CNVR6075.1−APEX1
CNVR6609.6−CREBBP
CNVR6609.4−CREBBP
CNVR4484.1−SET
CNVR6609.1−CREBBP
CNVR6609.3−CREBBP
CEU
YRI
CHB
0.00
0.02
0.04
0.06
0.08
0.10

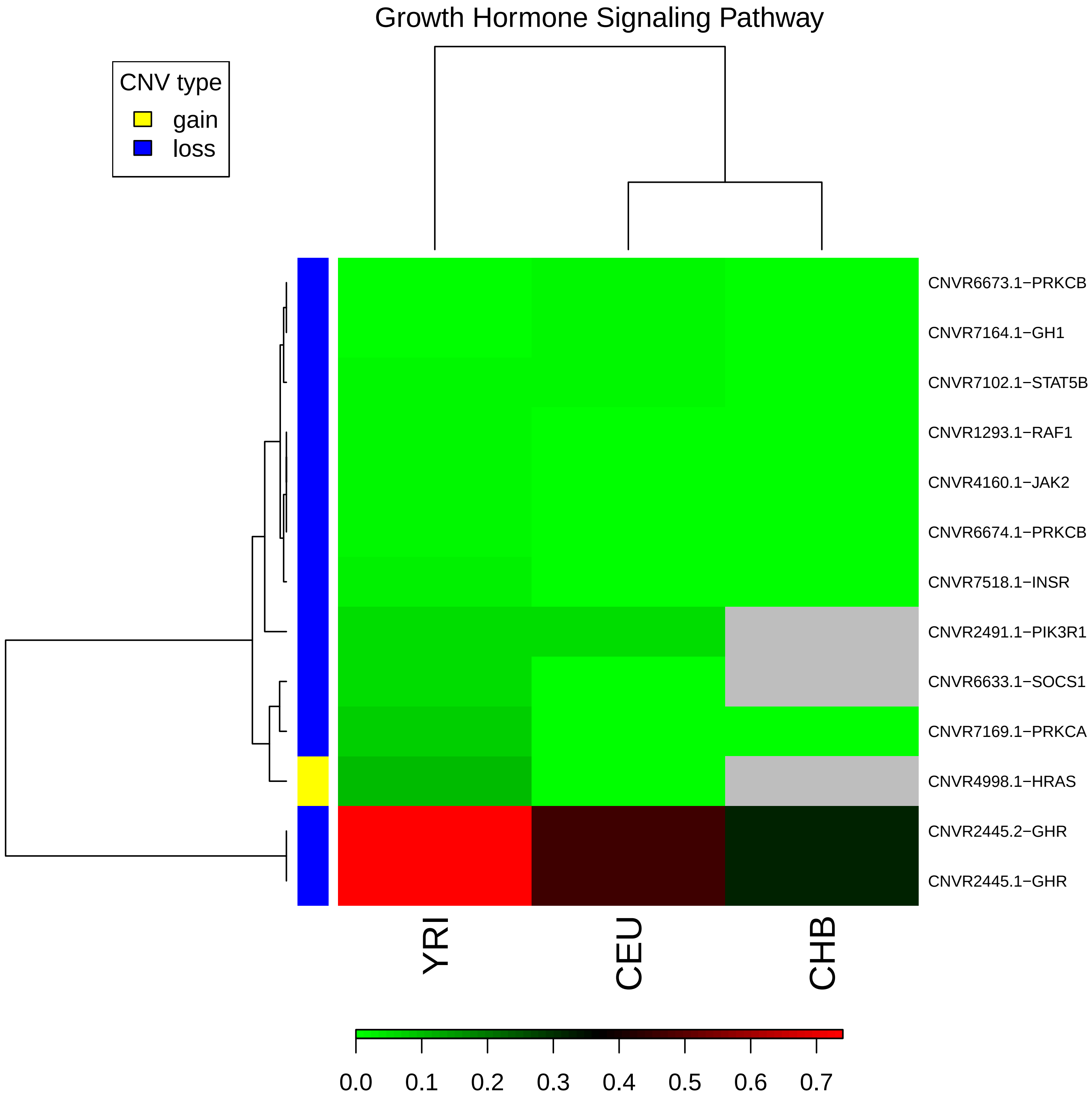

Growth Hormone Signaling Pathway
CNV type
gain
loss
CNVR6673.1−PRKCB
CNVR7164.1−GH1
CNVR7102.1−STAT5B
CNVR1293.1−RAF1
CNVR4160.1−JAK2
CNVR6674.1−PRKCB
CNVR7518.1−INSR
CNVR2491.1−PIK3R1
CNVR6633.1−SOCS1
CNVR7169.1−PRKCA
CNVR4998.1−HRAS
CNVR2445.2−GHR
CNVR2445.1−GHR
YRI
CEU
CHB
0.0
0.1
0.2
0.3
0.4
0.5
0.6
0.7

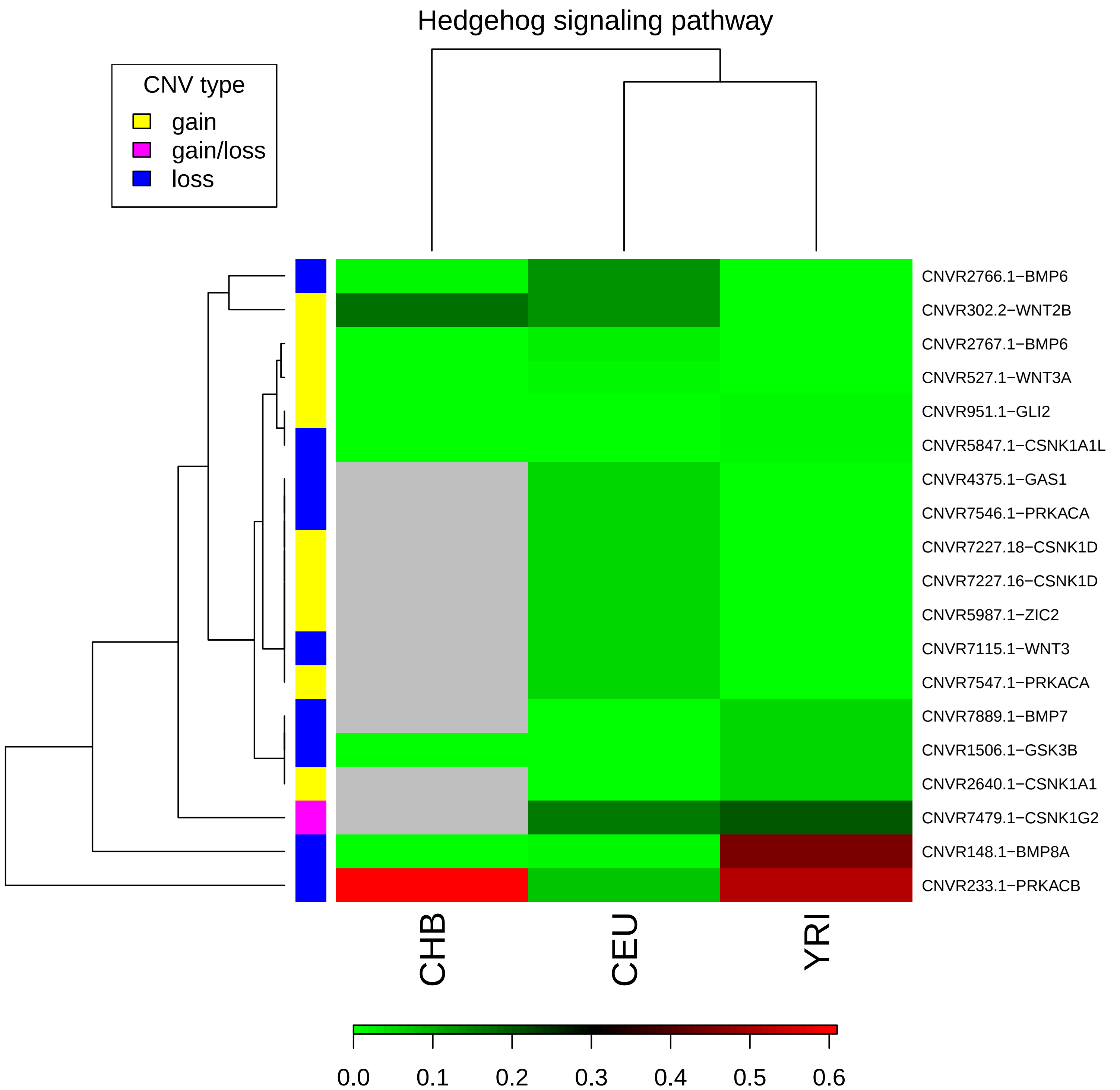
Hedgehog signaling pathway
CNV type
gain
gain/loss
loss
CNVR2766.1−BMP6
CNVR302.2−WNT2B
CNVR2767.1−BMP6
CNVR527.1−WNT3A
CNVR951.1−GLI2
CNVR5847.1−CSNK1A1L
CNVR4375.1−GAS1
CNVR7546.1−PRKACA
CNVR7227.18−CSNK1D
CNVR7227.16−CSNK1D
CNVR5987.1−ZIC2
CNVR7115.1−WNT3
CNVR7547.1−PRKACA
CNVR7889.1−BMP7
CNVR1506.1−GSK3B
CNVR2640.1−CSNK1A1
CNVR7479.1−CSNK1G2
CNVR148.1−BMP8A
CNVR233.1−PRKACB
CHB
CEU
YRI
0.0
0.1
0.2
0.3
0.4
0.5
0.6

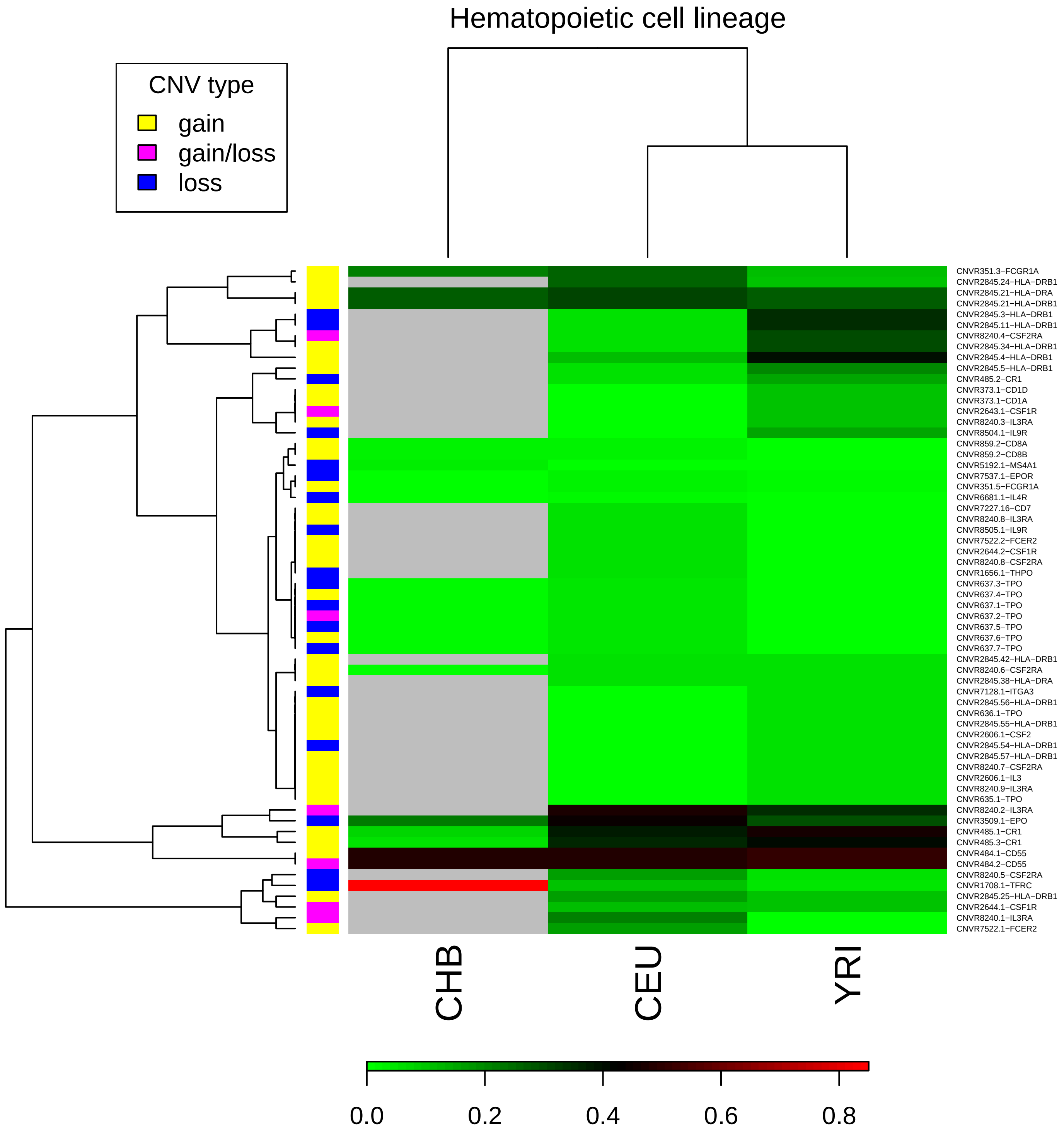

Hematopoietic cell lineage
CNV type
gain
gain/loss
loss
CNVR351.3−FCGR1A
CNVR2845.24−HLA−DRB1
CNVR2845.21−HLA−DRA
CNVR2845.21−HLA−DRB1
CNVR2845.3−HLA−DRB1
CNVR2845.11−HLA−DRB1
CNVR8240.4−CSF2RA
CNVR2845.34−HLA−DRB1
CNVR2845.4−HLA−DRB1
CNVR2845.5−HLA−DRB1
CNVR485.2−CR1
CNVR373.1−CD1D
CNVR373.1−CD1A
CNVR2643.1−CSF1R
CNVR8240.3−IL3RA
CNVR8504.1−IL9R
CNVR859.2−CD8A
CNVR859.2−CD8B
CNVR5192.1−MS4A1
CNVR7537.1−EPOR
CNVR351.5−FCGR1A
CNVR6681.1−IL4R
CNVR7227.16−CD7
CNVR8240.8−IL3RA
CNVR8505.1−IL9R
CNVR7522.2−FCER2
CNVR2644.2−CSF1R
CNVR8240.8−CSF2RA
CNVR1656.1−THPO
CNVR637.3−TPO
CNVR637.4−TPO
CNVR637.1−TPO
CNVR637.2−TPO
CNVR637.5−TPO
CNVR637.6−TPO
CNVR637.7−TPO
CNVR2845.42−HLA−DRB1
CNVR8240.6−CSF2RA
CNVR2845.38−HLA−DRA
CNVR7128.1−ITGA3
CNVR2845.56−HLA−DRB1
CNVR636.1−TPO
CNVR2845.55−HLA−DRB1
CNVR2606.1−CSF2
CNVR2845.54−HLA−DRB1
CNVR2845.57−HLA−DRB1
CNVR8240.7−CSF2RA
CNVR2606.1−IL3
CNVR8240.9−IL3RA
CNVR635.1−TPO
CNVR8240.2−IL3RA
CNVR3509.1−EPO
CNVR485.1−CR1
CNVR485.3−CR1
CNVR484.1−CD55
CNVR484.2−CD55
CNVR8240.5−CSF2RA
CNVR1708.1−TFRC
CNVR2845.25−HLA−DRB1
CNVR2644.1−CSF1R
CNVR8240.1−IL3RA
CNVR7522.1−FCER2
CHB
CEU
YRI
0.0
0.2
0.4
0.6
0.8

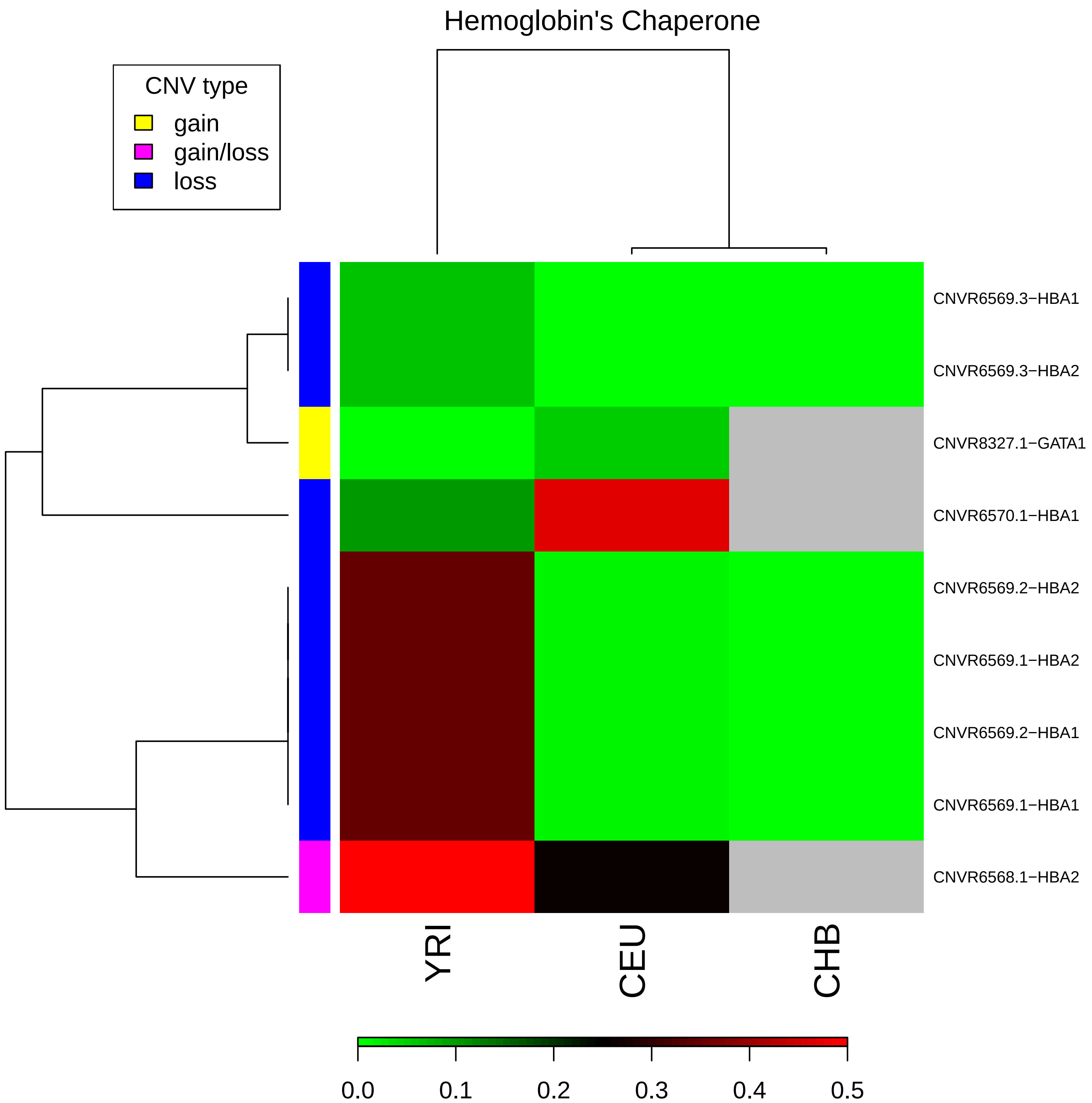

Hemoglobin's Chaperone
CNV type
gain
gain/loss
loss
CNVR6569.3−HBA1
CNVR6569.3−HBA2
CNVR8327.1−GATA1
CNVR6570.1−HBA1
CNVR6569.2−HBA2
CNVR6569.1−HBA2
CNVR6569.2−HBA1
CNVR6569.1−HBA1
CNVR6568.1−HBA2
YRI
CEU
CHB
0.0
0.1
0.2
0.3
0.4
0.5

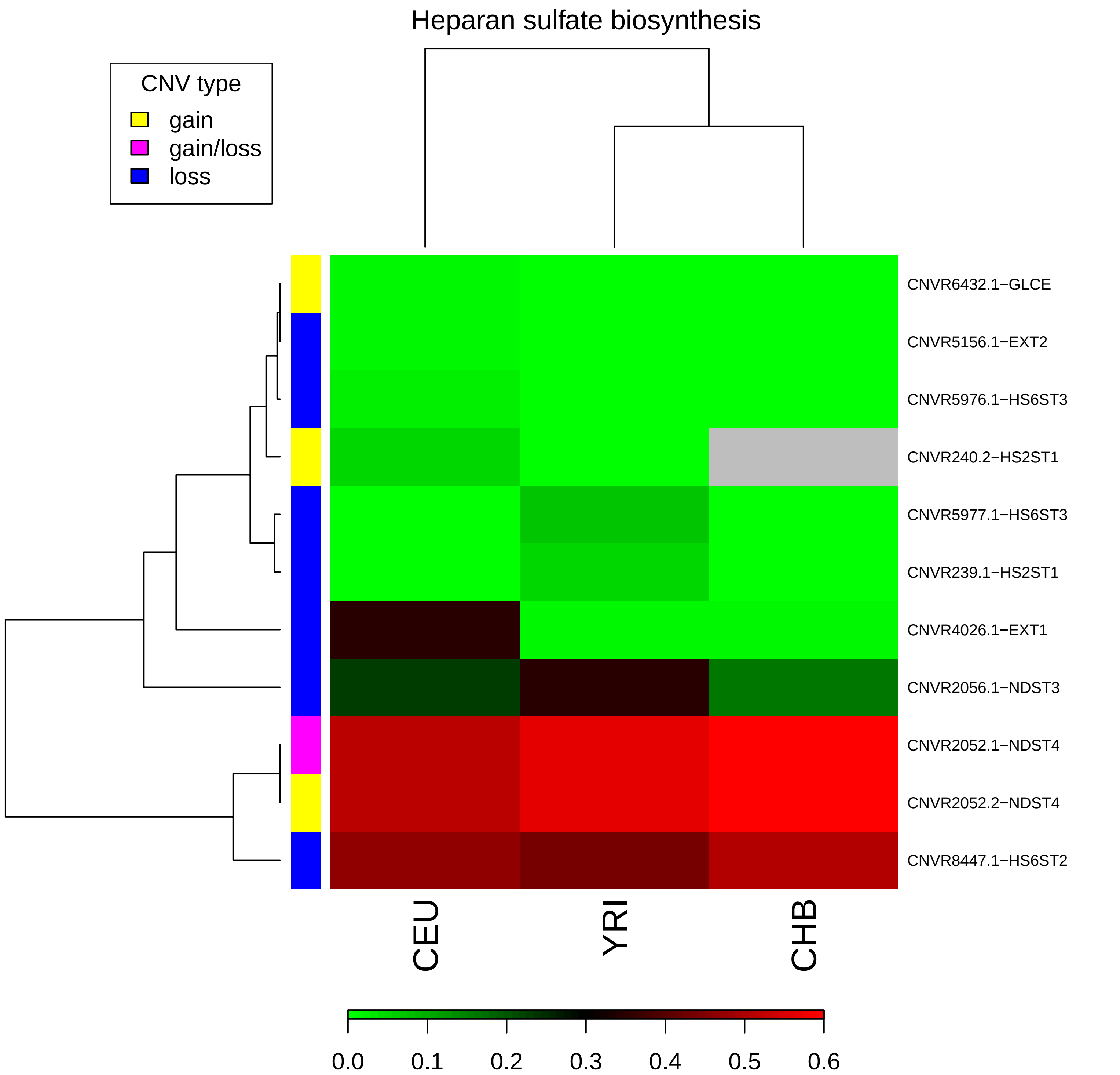

Heparan sulfate biosynthesis
CNV type
gain
gain/loss
loss
CNVR6432.1−GLCE
CNVR5156.1−EXT2
CNVR5976.1−HS6ST3
CNVR240.2−HS2ST1
CNVR5977.1−HS6ST3
CNVR239.1−HS2ST1
CNVR4026.1−EXT1
CNVR2056.1−NDST3
CNVR2052.1−NDST4
CNVR2052.2−NDST4
CNVR8447.1−HS6ST2
CEU
YRI
CHB
0.0
0.1
0.2
0.3
0.4
0.5
0.6

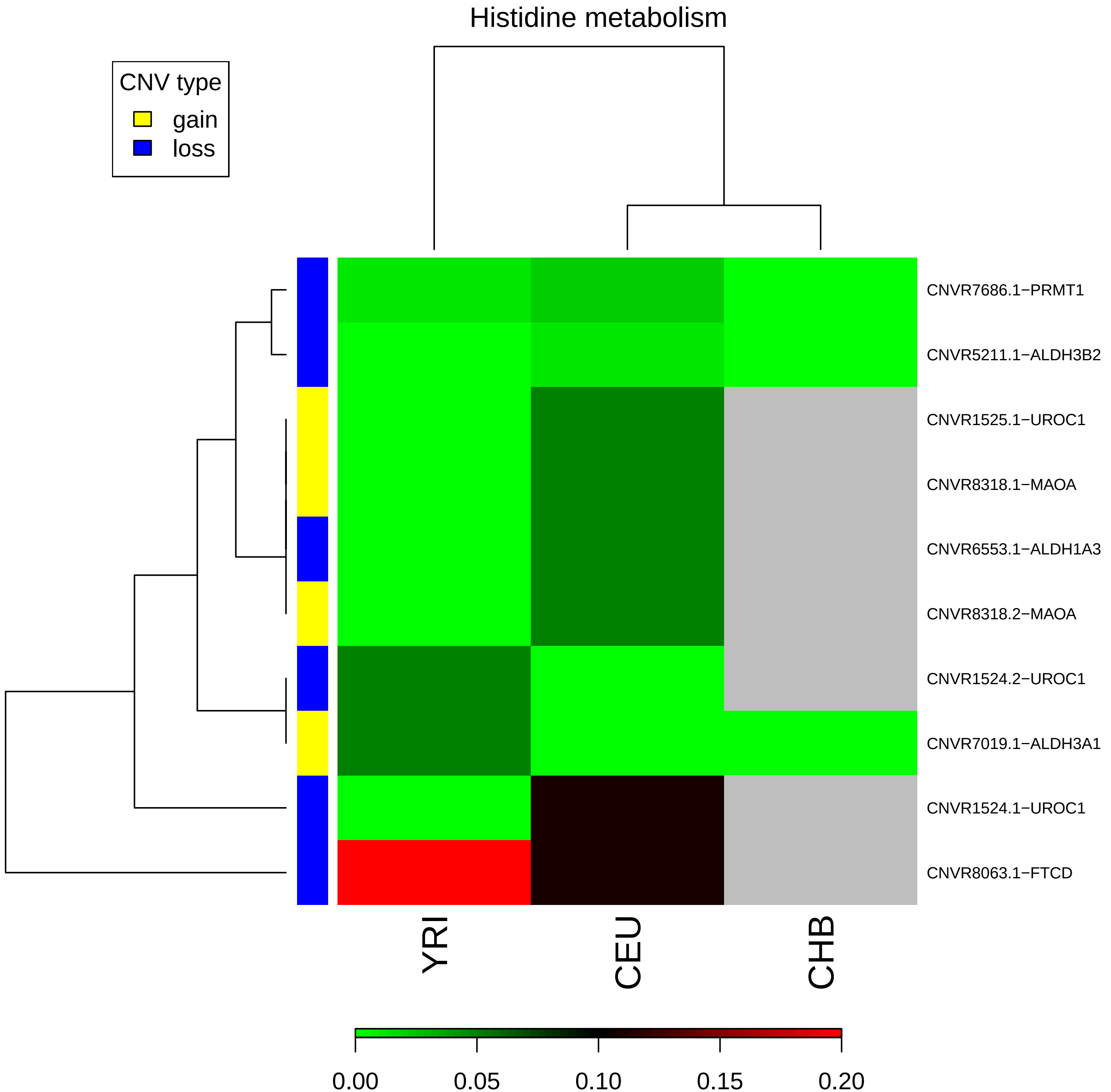

Histidine metabolism
CNV type
gain
loss
CNVR7686.1−PRMT1
CNVR5211.1−ALDH3B2
CNVR1525.1−UROC1
CNVR8318.1−MAOA
CNVR6553.1−ALDH1A3
CNVR8318.2−MAOA
CNVR1524.2−UROC1
CNVR7019.1−ALDH3A1
CNVR1524.1−UROC1
CNVR8063.1−FTCD
YRI
CEU
CHB
0.00
0.05
0.10
0.15
0.20

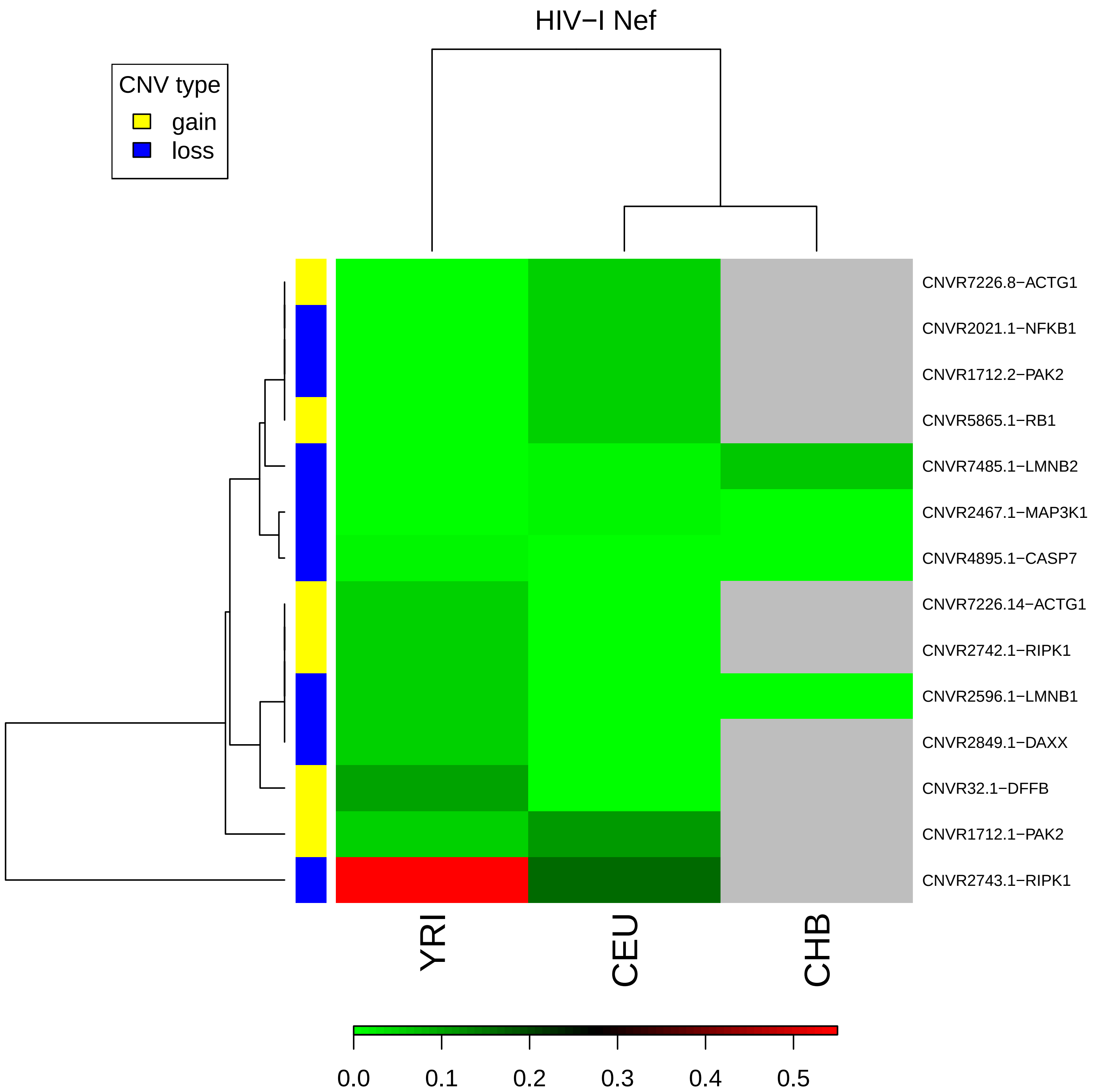

HIV−I Nef
CNV type
gain
loss
CNVR7226.8−ACTG1
CNVR2021.1−NFKB1
CNVR1712.2−PAK2
CNVR5865.1−RB1
CNVR7485.1−LMNB2
CNVR2467.1−MAP3K1
CNVR4895.1−CASP7
CNVR7226.14−ACTG1
CNVR2742.1−RIPK1
CNVR2596.1−LMNB1
CNVR2849.1−DAXX
CNVR32.1−DFFB
CNVR1712.1−PAK2
CNVR2743.1−RIPK1
YRI
CEU
CHB
0.0
0.1
0.2
0.3
0.4
0.5

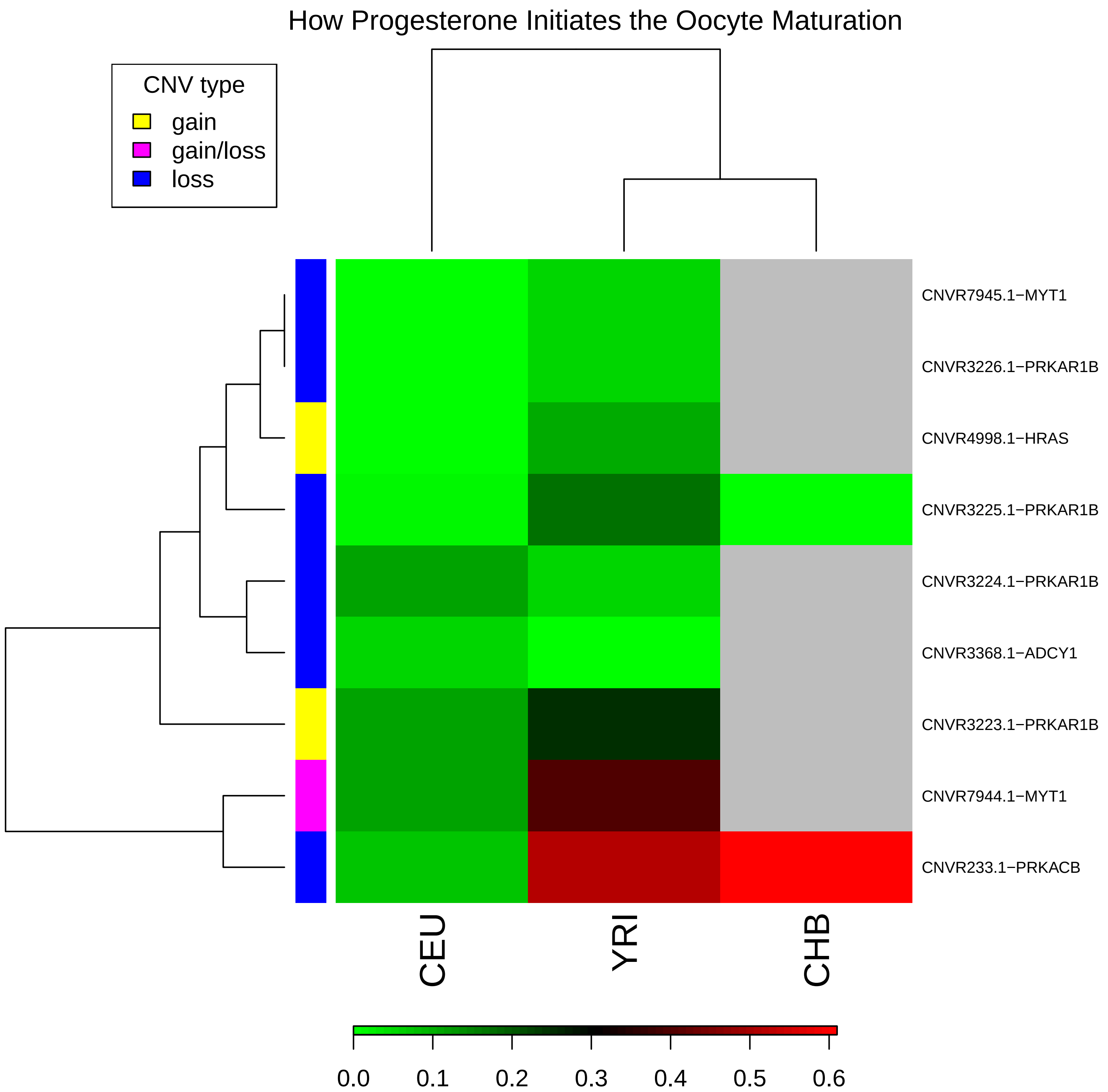

How Progesterone Initiates the Oocyte Maturation
CNV type
gain
gain/loss
loss
CNVR7945.1−MYT1
CNVR3226.1−PRKAR1B
CNVR4998.1−HRAS
CNVR3225.1−PRKAR1B
CNVR3224.1−PRKAR1B
CNVR3368.1−ADCY1
CNVR3223.1−PRKAR1B
CNVR7944.1−MYT1
CNVR233.1−PRKACB
CEU
YRI
CHB
0.0
0.1
0.2
0.3
0.4
0.5
0.6

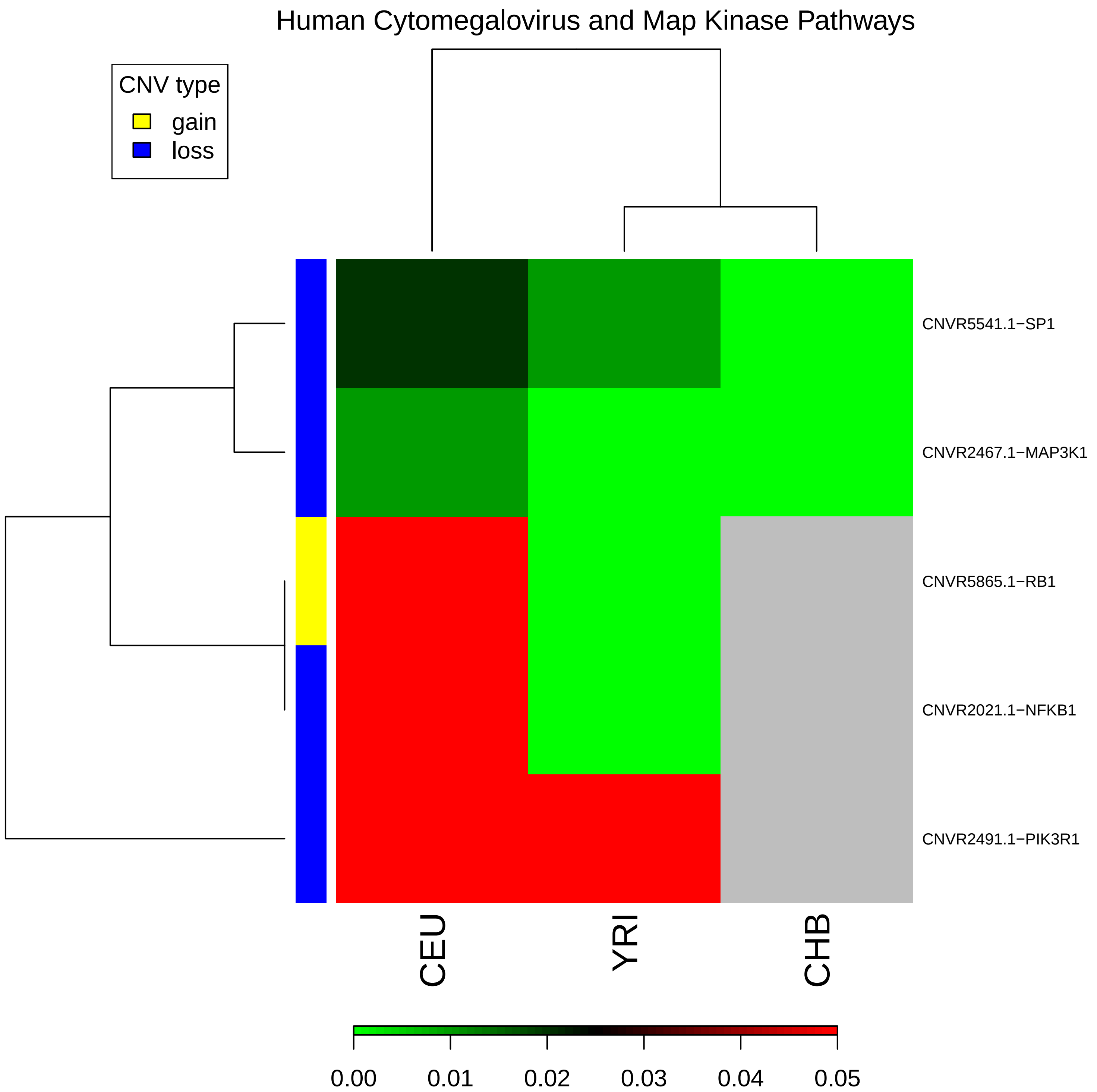

Human Cytomegalovirus and Map Kinase Pathways
CNV type
gain
loss
CNVR5541.1−SP1
CNVR2467.1−MAP3K1
CNVR5865.1−RB1
CNVR2021.1−NFKB1
CNVR2491.1−PIK3R1
CEU
YRI
CHB
0.00
0.01
0.02
0.03
0.04
0.05

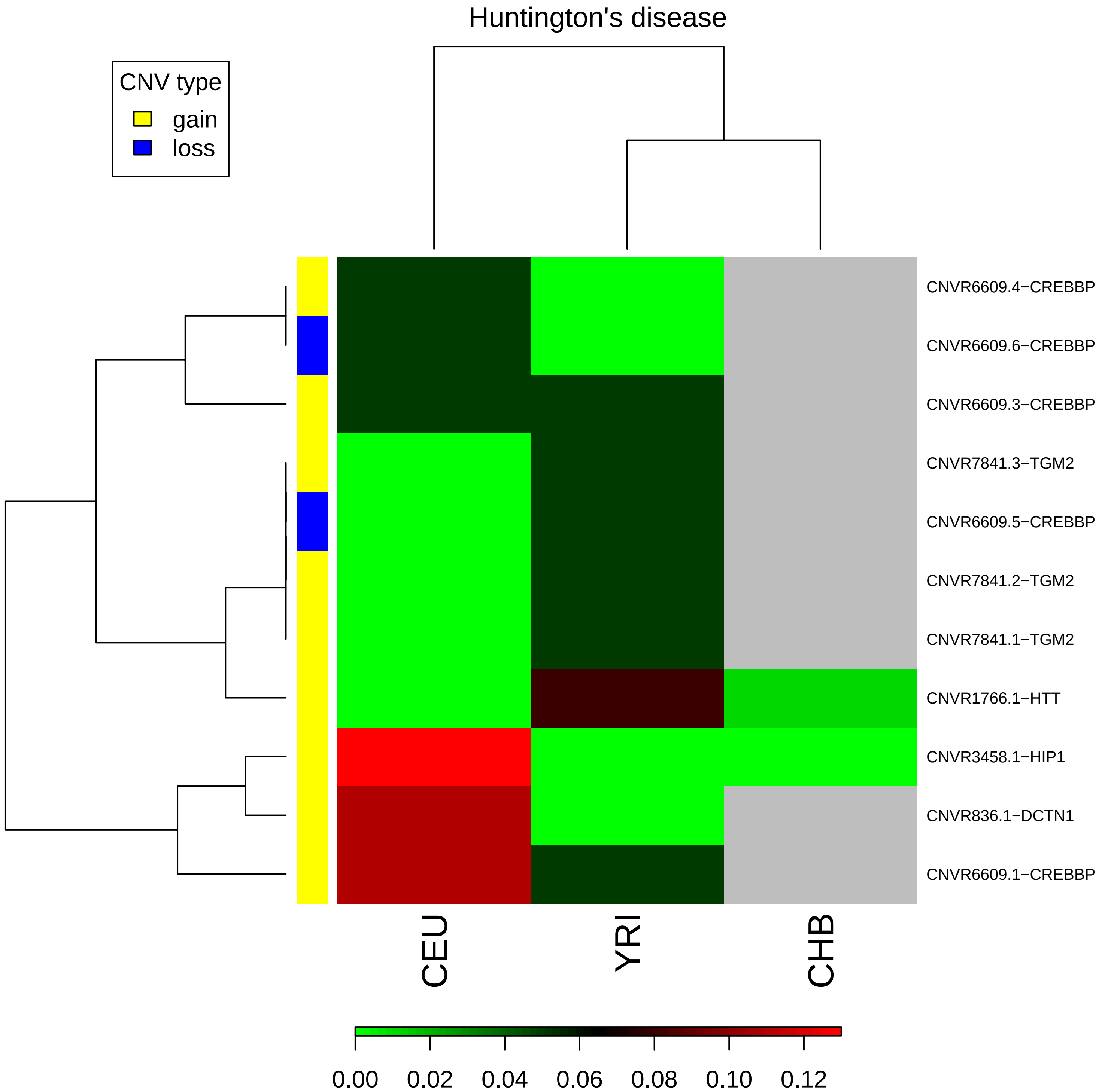

Huntington's disease
CNV type
gain
loss
CNVR6609.4−CREBBP
CNVR6609.6−CREBBP
CNVR6609.3−CREBBP
CNVR7841.3−TGM2
CNVR6609.5−CREBBP
CNVR7841.2−TGM2
CNVR7841.1−TGM2
CNVR1766.1−HTT
CNVR3458.1−HIP1
CNVR836.1−DCTN1
CNVR6609.1−CREBBP
CEU
YRI
CHB
0.00
0.02
0.04
0.06
0.08
0.10
0.12

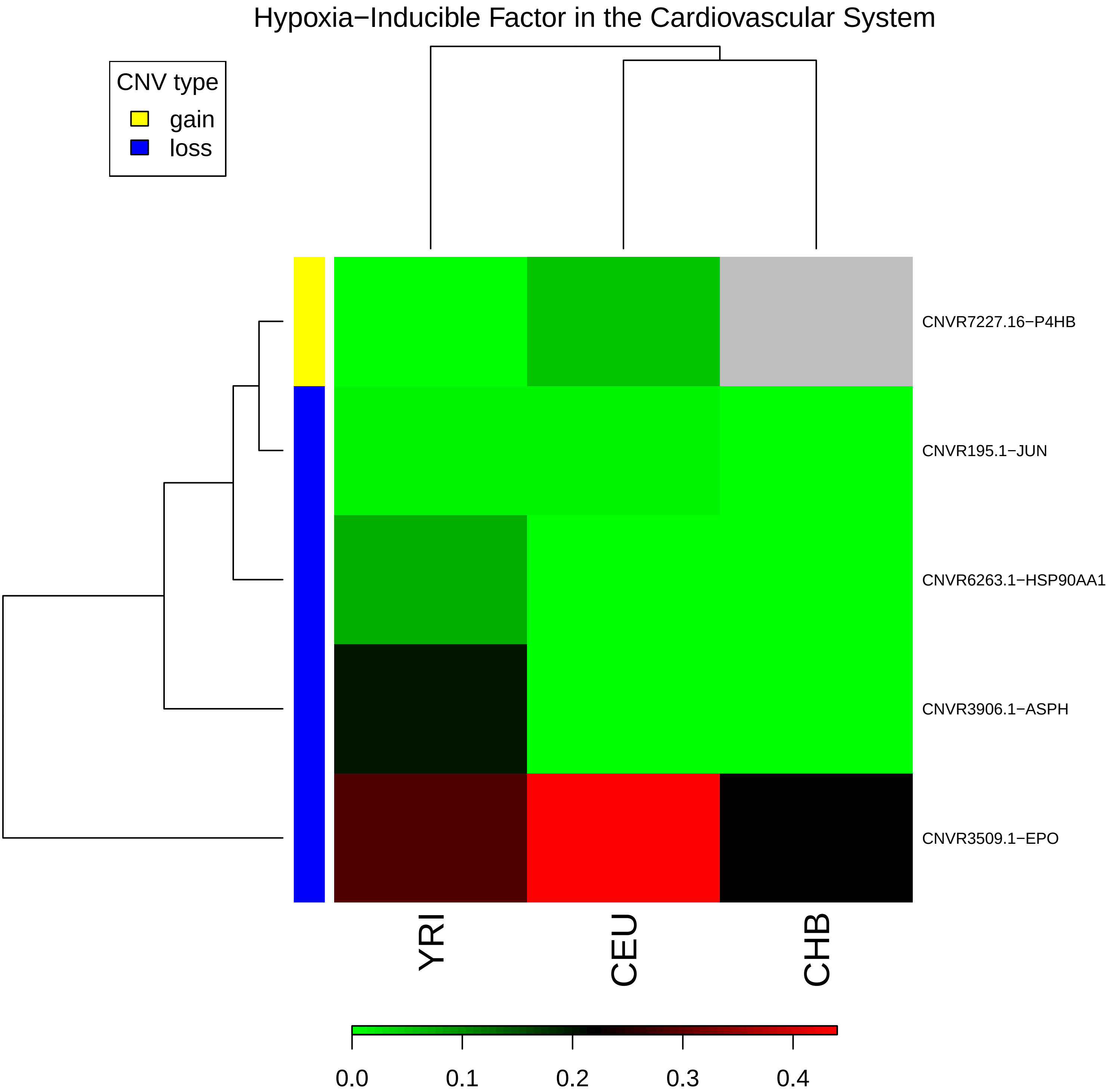
Hypoxia−Inducible Factor in the Cardiovascular System
CNV type
gain
loss
CNVR7227.16−P4HB
CNVR195.1−JUN
CNVR6263.1−HSP90AA1
CNVR3906.1−ASPH
CNVR3509.1−EPO
YRI
CEU
CHB
0.0
0.1
0.2
0.3
0.4

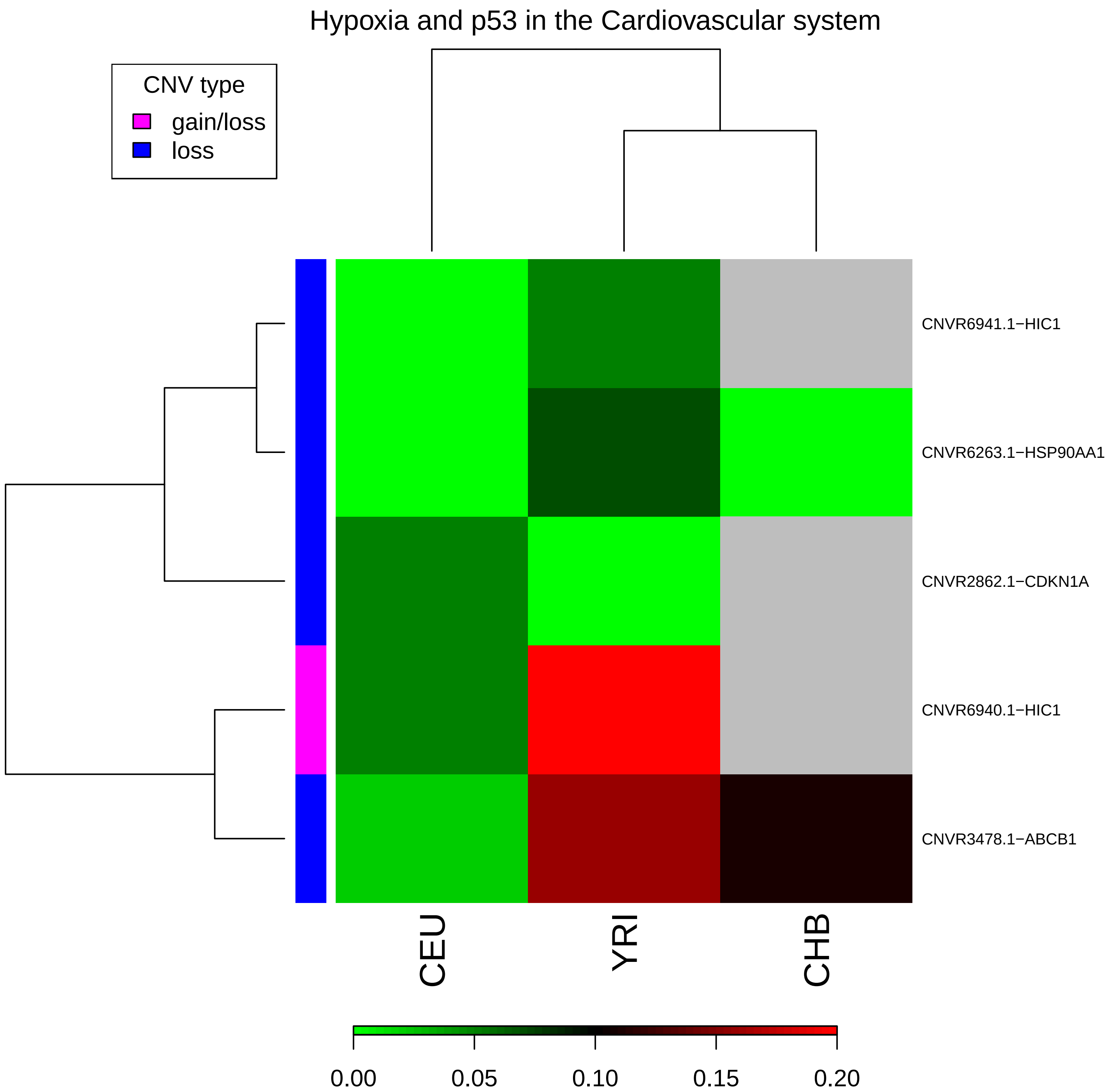

Hypoxia and p53 in the Cardiovascular system
CNV type
gain/loss
loss
CNVR6941.1−HIC1
CNVR6263.1−HSP90AA1
CNVR2862.1−CDKN1A
CNVR6940.1−HIC1
CNVR3478.1−ABCB1
CEU
YRI
CHB
0.00
0.05
0.10
0.15
0.20

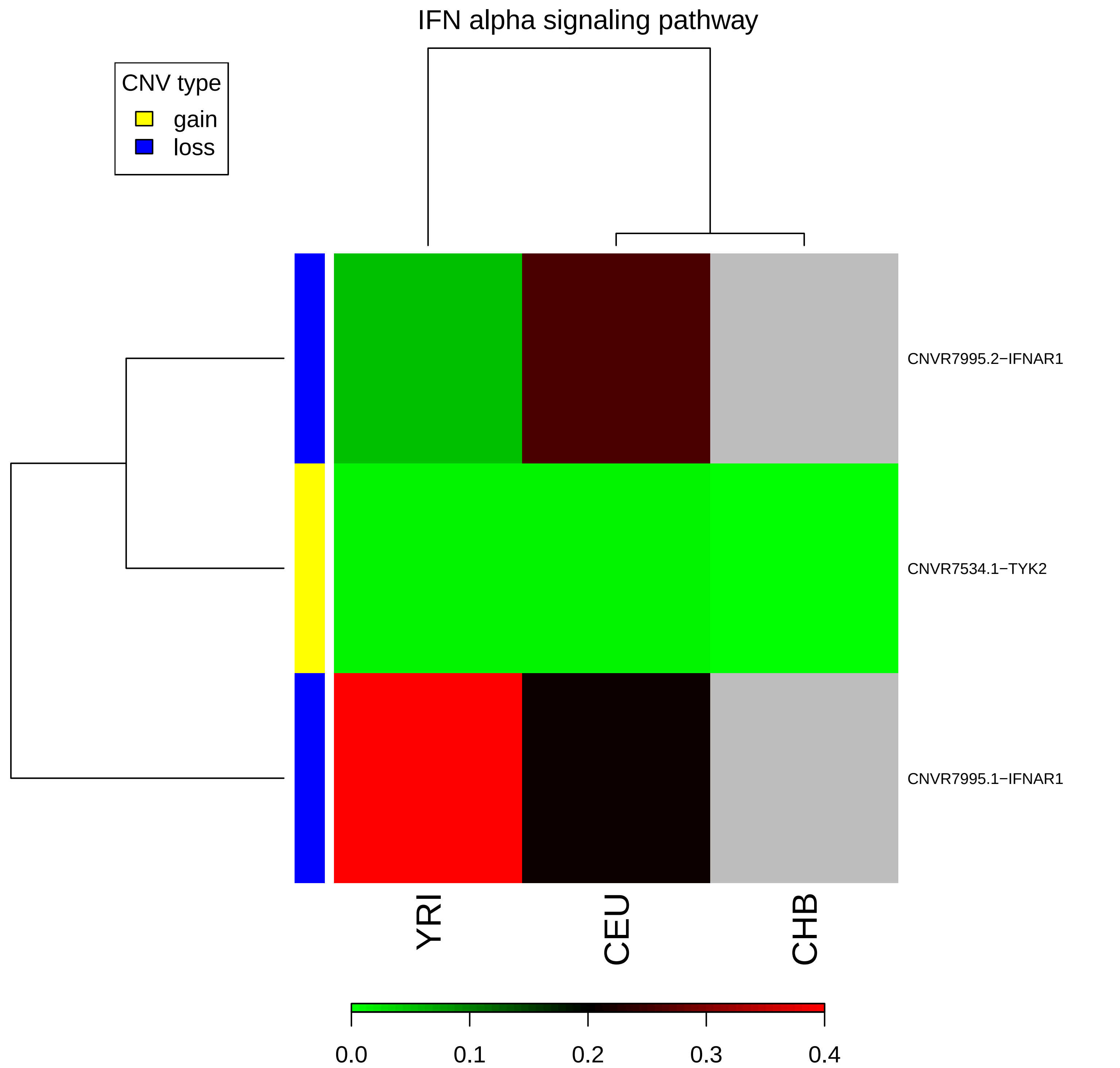
IFN alpha signaling pathway
CNV type
gain
loss
CNVR7995.2−IFNAR1
CNVR7534.1−TYK2
CNVR7995.1−IFNAR1
YRI
CEU
CHB
0.0
0.1
0.2
0.3
0.4

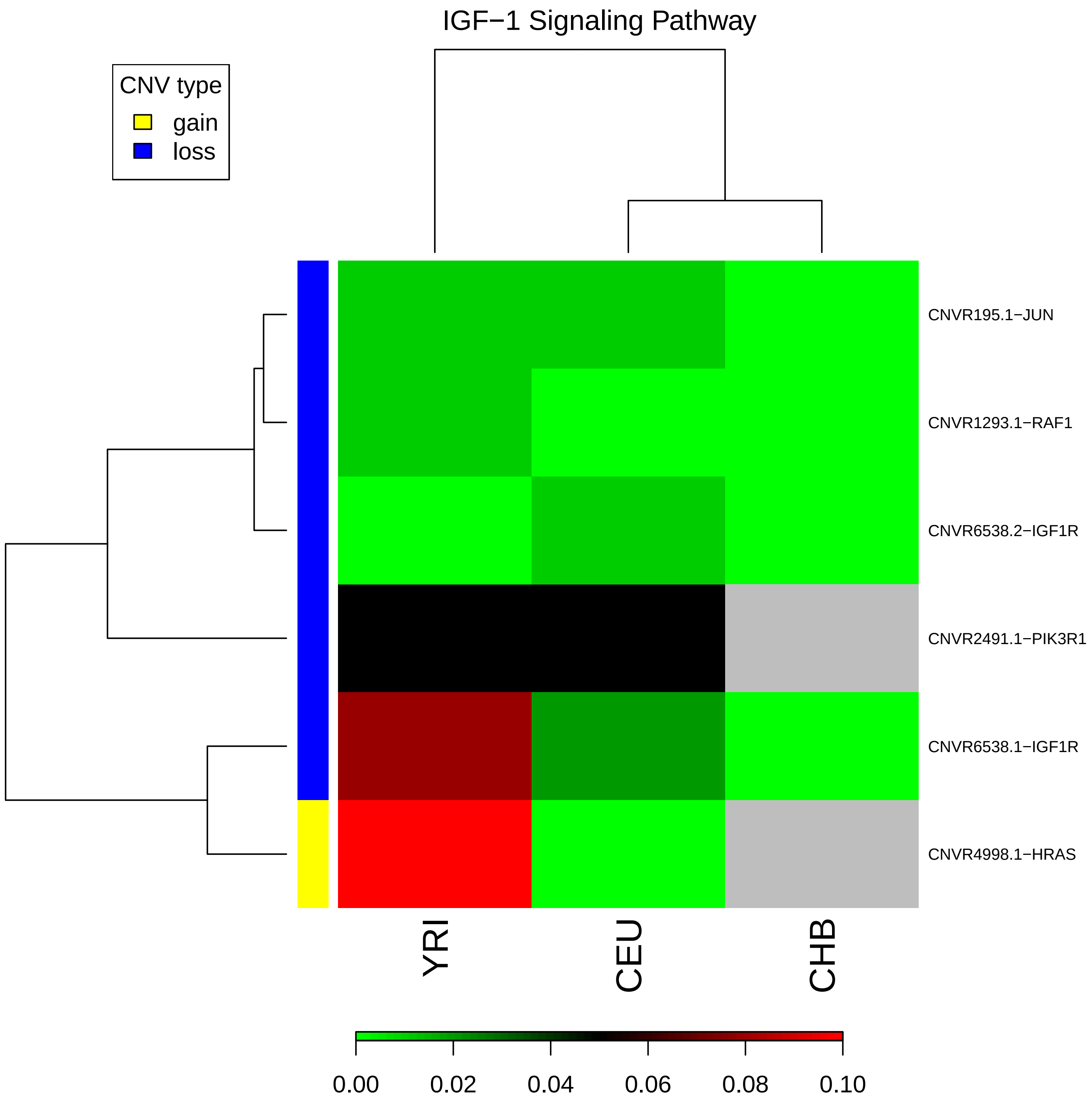
IGF−1 Signaling Pathway
CNV type
gain
loss
CNVR195.1−JUN
CNVR1293.1−RAF1
CNVR6538.2−IGF1R
CNVR2491.1−PIK3R1
CNVR6538.1−IGF1R
CNVR4998.1−HRAS
YRI
CEU
CHB
0.00
0.02
0.04
0.06
0.08
0.10

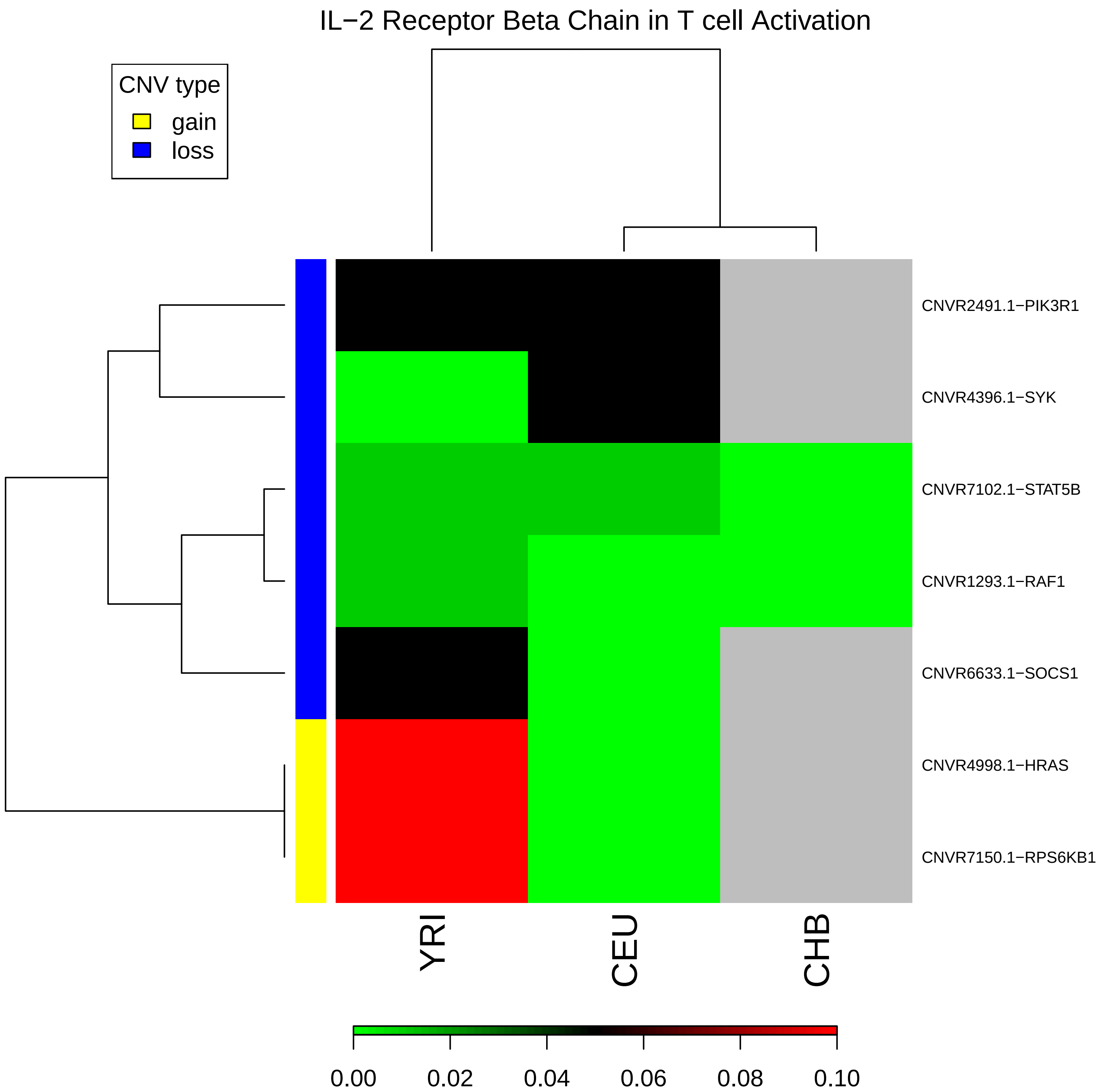
IL−2 Receptor Beta Chain in T cell Activation
CNV type
gain
loss
CNVR2491.1−PIK3R1
CNVR4396.1−SYK
CNVR7102.1−STAT5B
CNVR1293.1−RAF1
CNVR6633.1−SOCS1
CNVR4998.1−HRAS
CNVR7150.1−RPS6KB1
YRI
CEU
CHB
0.00
0.02
0.04
0.06
0.08
0.10

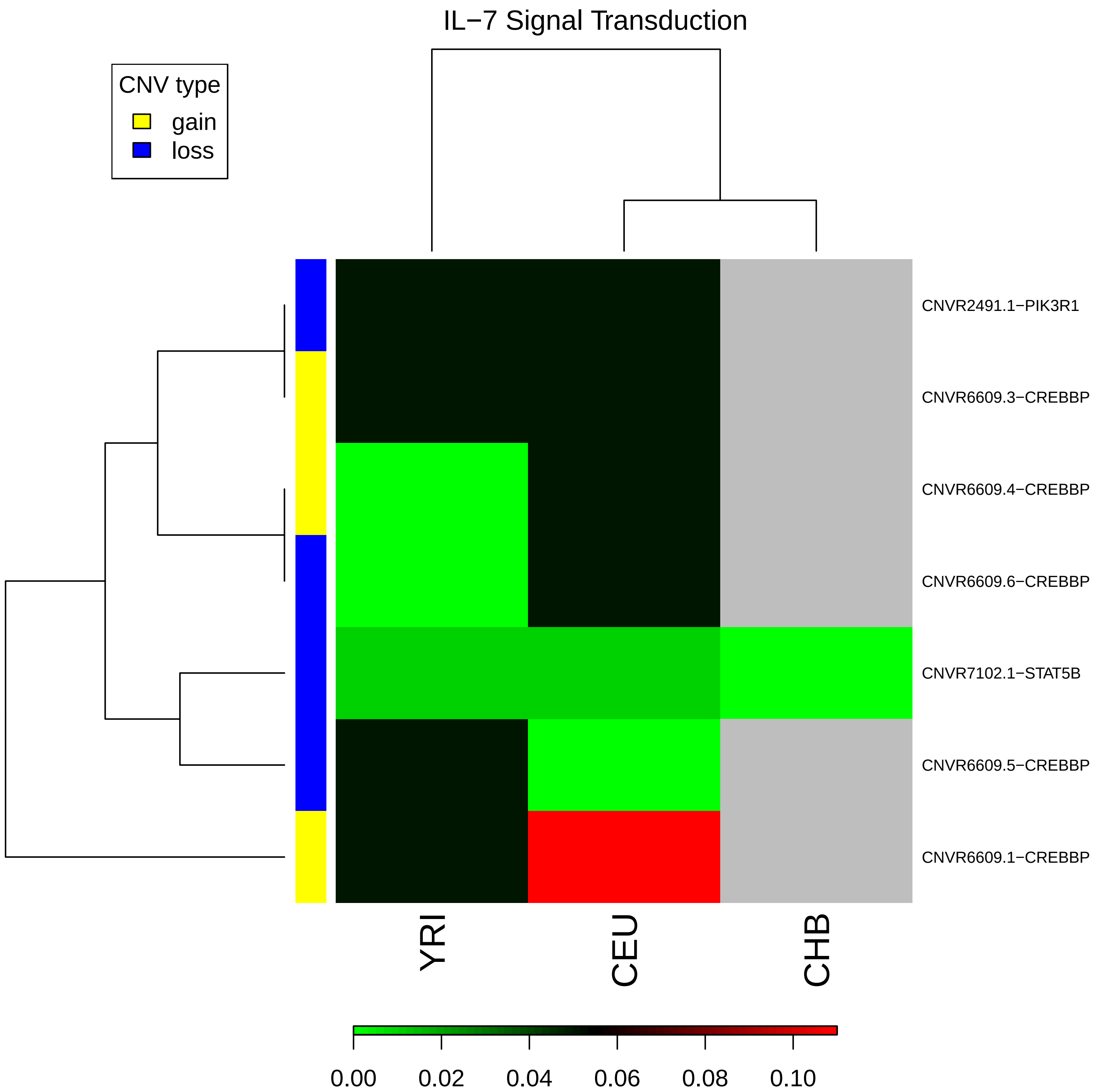
IL−7 Signal Transduction
CNV type
gain
loss
CNVR2491.1−PIK3R1
CNVR6609.3−CREBBP
CNVR6609.4−CREBBP
CNVR6609.6−CREBBP
CNVR7102.1−STAT5B
CNVR6609.5−CREBBP
CNVR6609.1−CREBBP
YRI
CEU
CHB
0.00
0.02
0.04
0.06
0.08
0.10

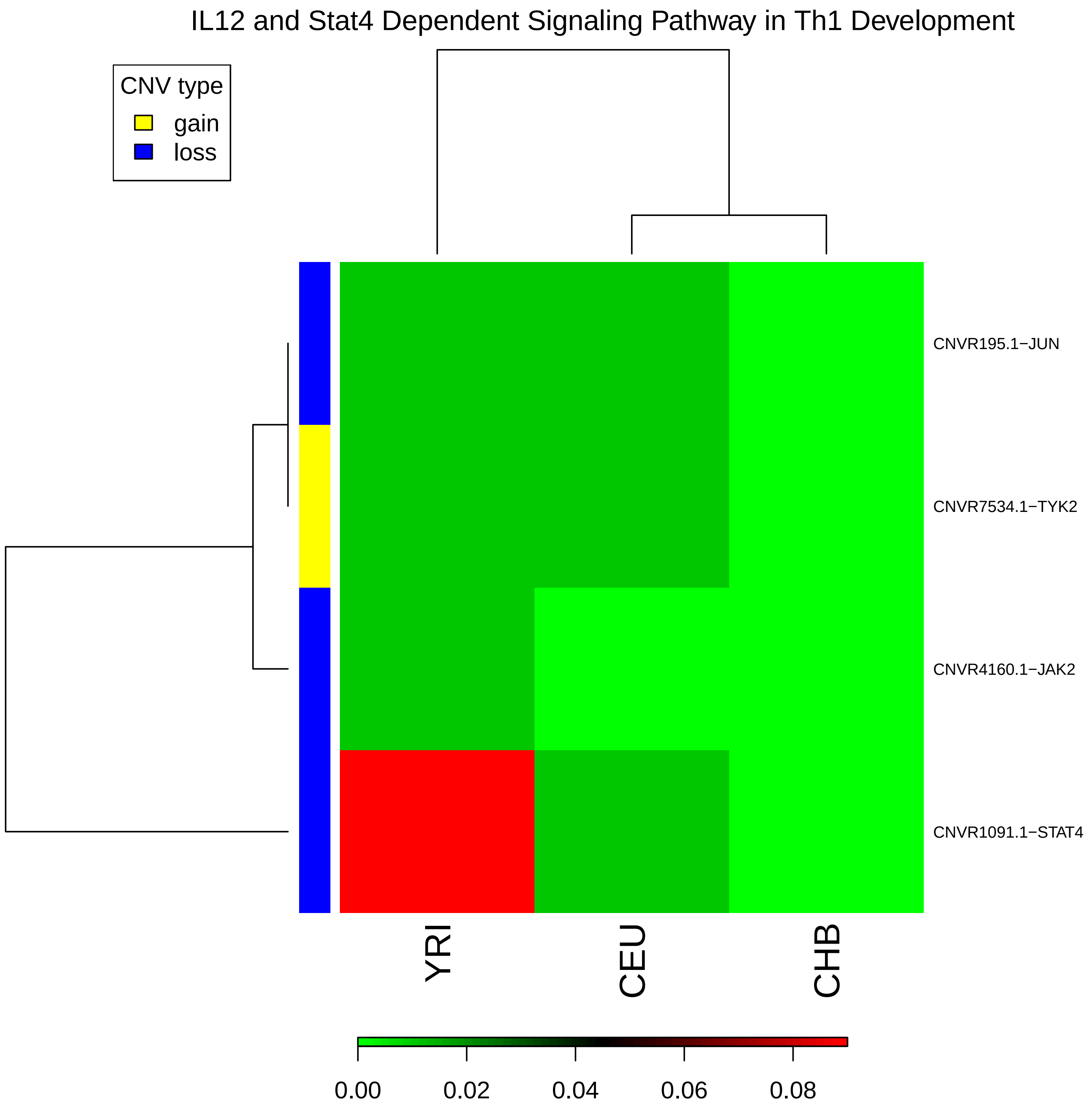

IL12 and Stat4 Dependent Signaling Pathway in Th1 Development
CNV type
gain
loss
CNVR195.1−JUN
CNVR7534.1−TYK2
CNVR4160.1−JAK2
CNVR1091.1−STAT4
YRI
CEU
CHB
0.00
0.02
0.04
0.06
0.08

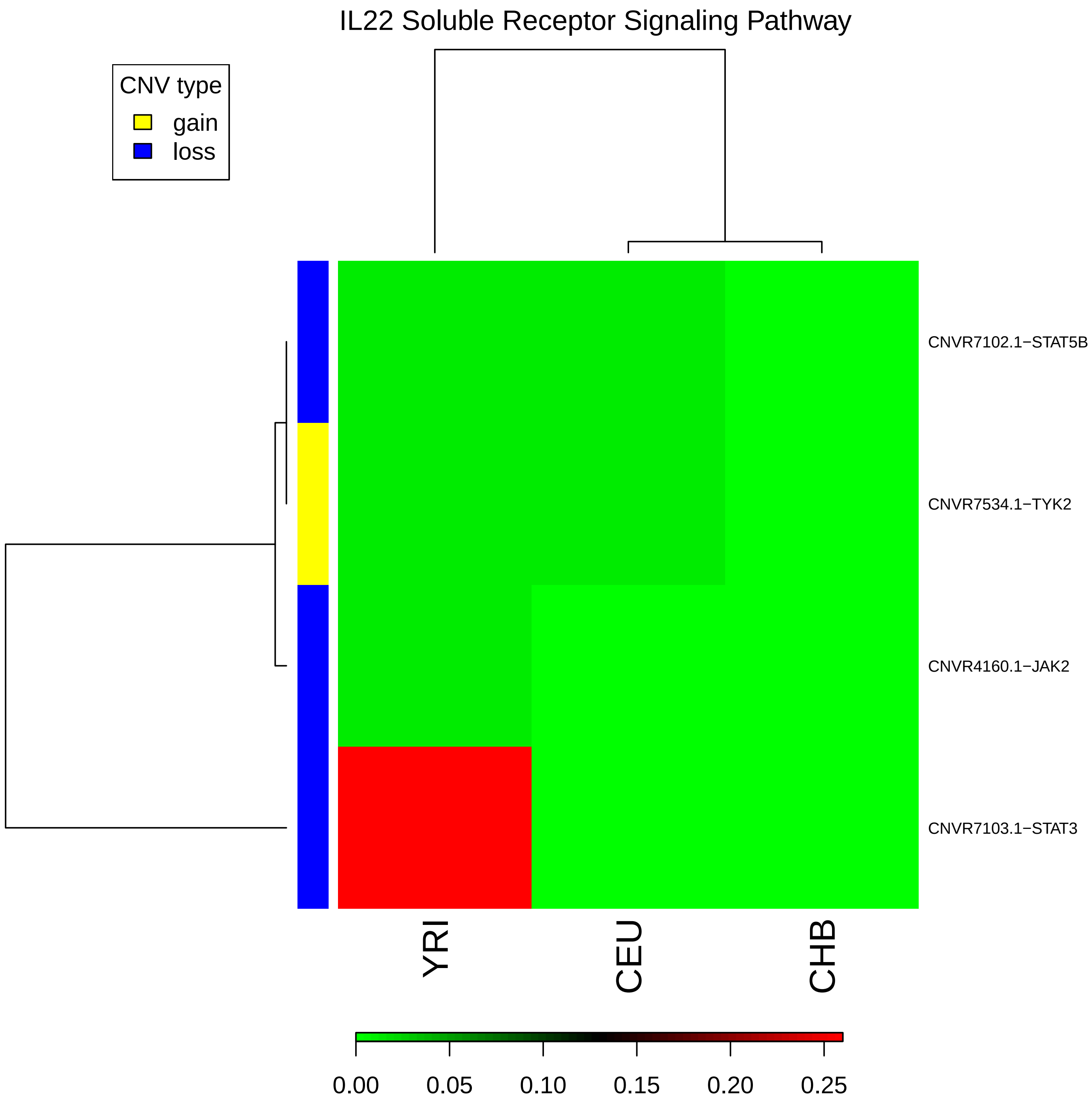
IL22 Soluble Receptor Signaling Pathway
CNV type
gain
loss
CNVR7102.1−STAT5B
CNVR7534.1−TYK2
CNVR4160.1−JAK2
CNVR7103.1−STAT3
YRI
CEU
CHB
0.00
0.05
0.10
0.15
0.20
0.25

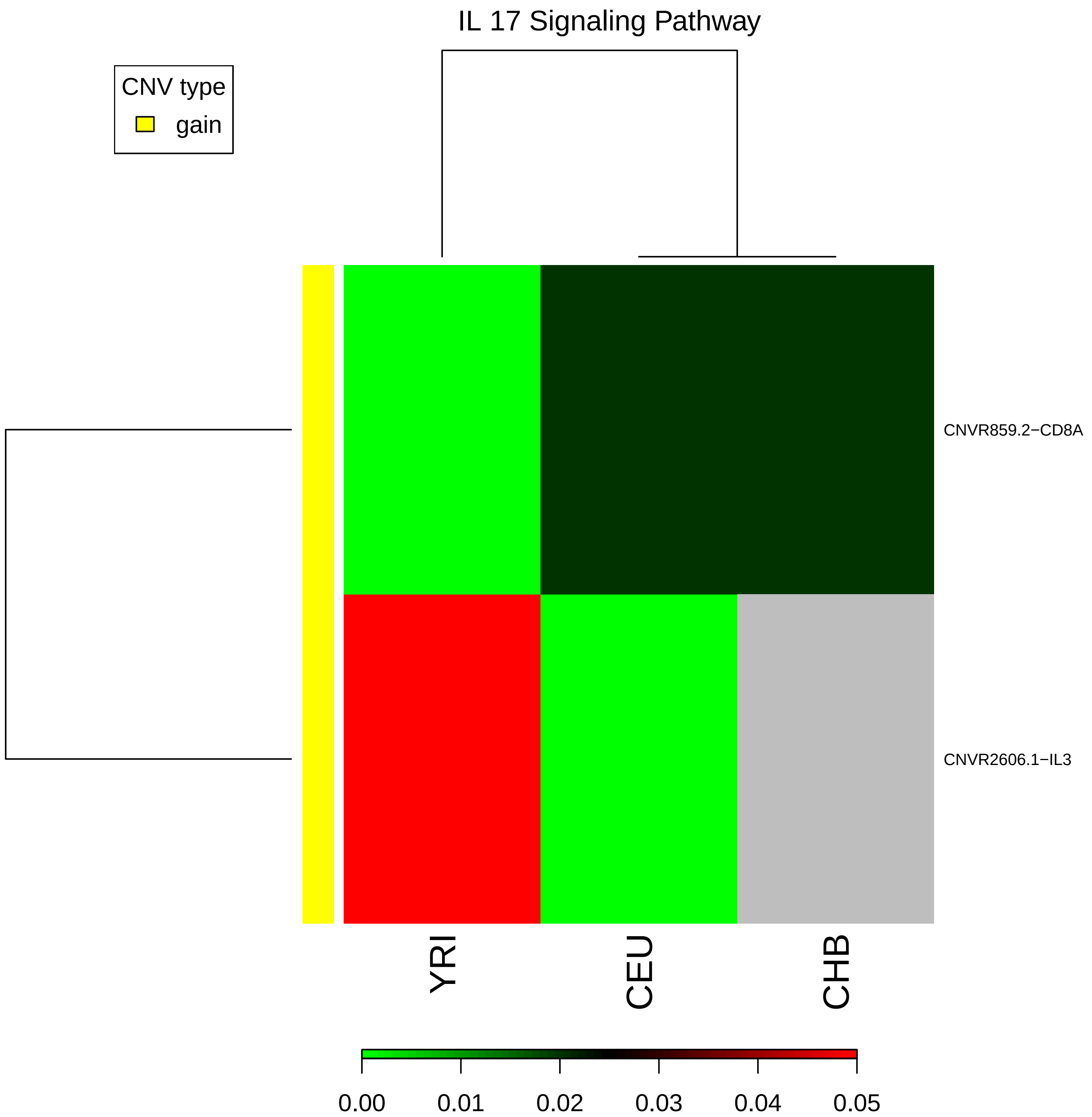
IL 17 Signaling Pathway
CNV type
gain
CNVR859.2−CD8A
CNVR2606.1−IL3
YRI
CEU
CHB
0.00
0.01
0.02
0.03
0.04
0.05

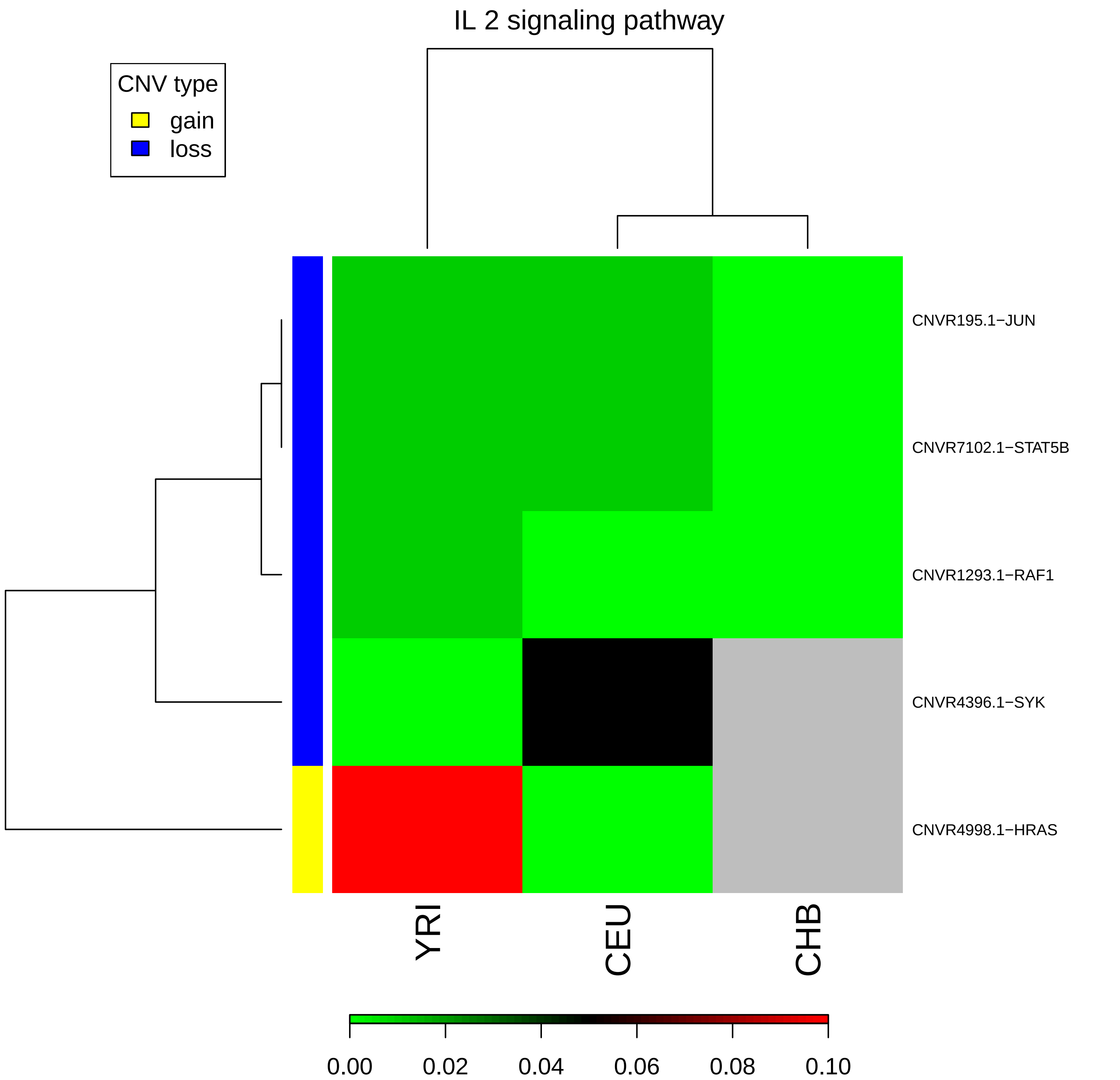

IL 2 signaling pathway
CNV type
gain
loss
CNVR195.1−JUN
CNVR7102.1−STAT5B
CNVR1293.1−RAF1
CNVR4396.1−SYK
CNVR4998.1−HRAS
YRI
CEU
CHB
0.00
0.02
0.04
0.06
0.08
0.10

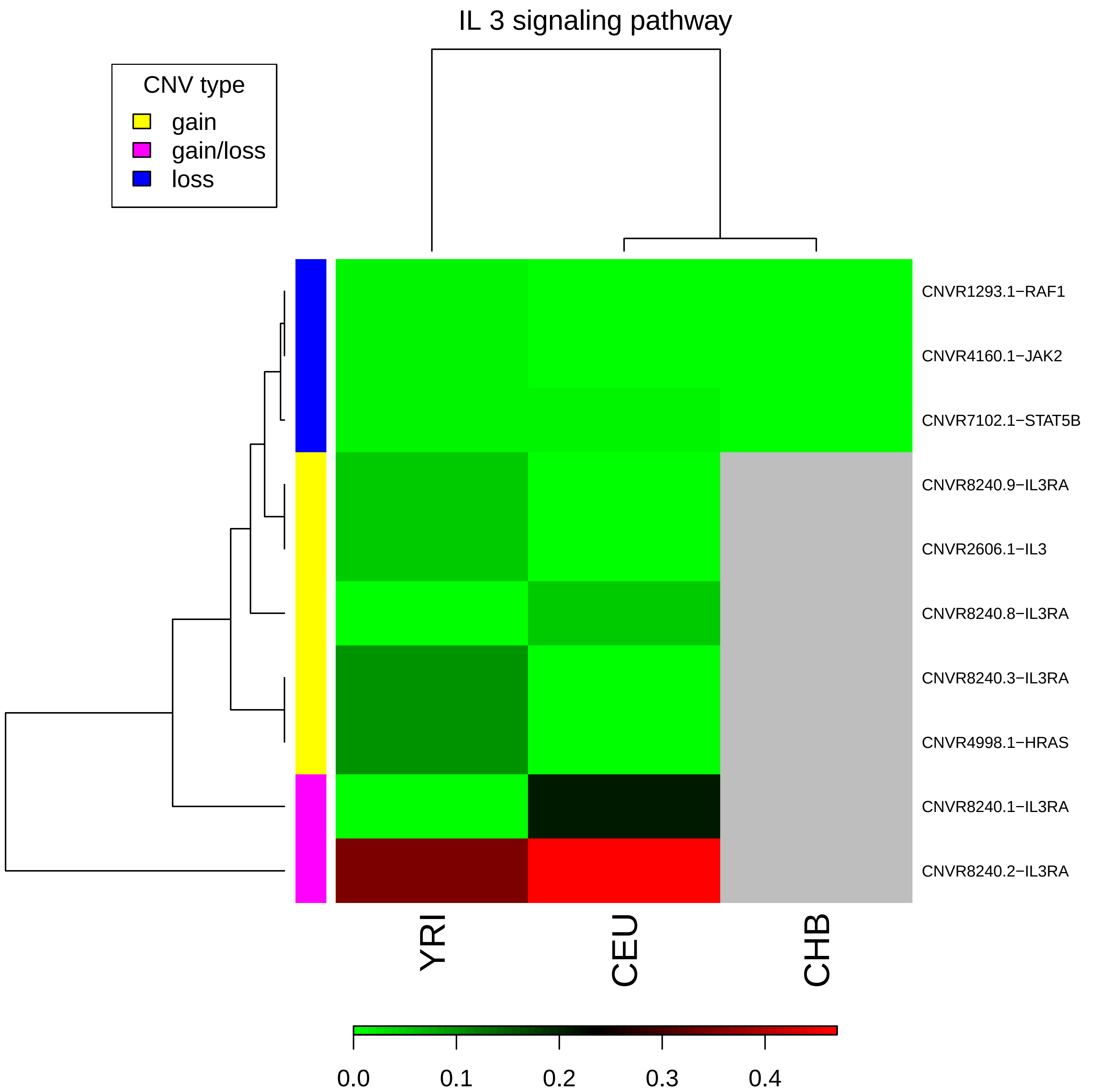
IL 3 signaling pathway
CNV type
gain
gain/loss
loss
CNVR1293.1−RAF1
CNVR4160.1−JAK2
CNVR7102.1−STAT5B
CNVR8240.9−IL3RA
CNVR2606.1−IL3
CNVR8240.8−IL3RA
CNVR8240.3−IL3RA
CNVR4998.1−HRAS
CNVR8240.1−IL3RA
CNVR8240.2−IL3RA
YRI
CEU
CHB
0.0
0.1
0.2
0.3
0.4

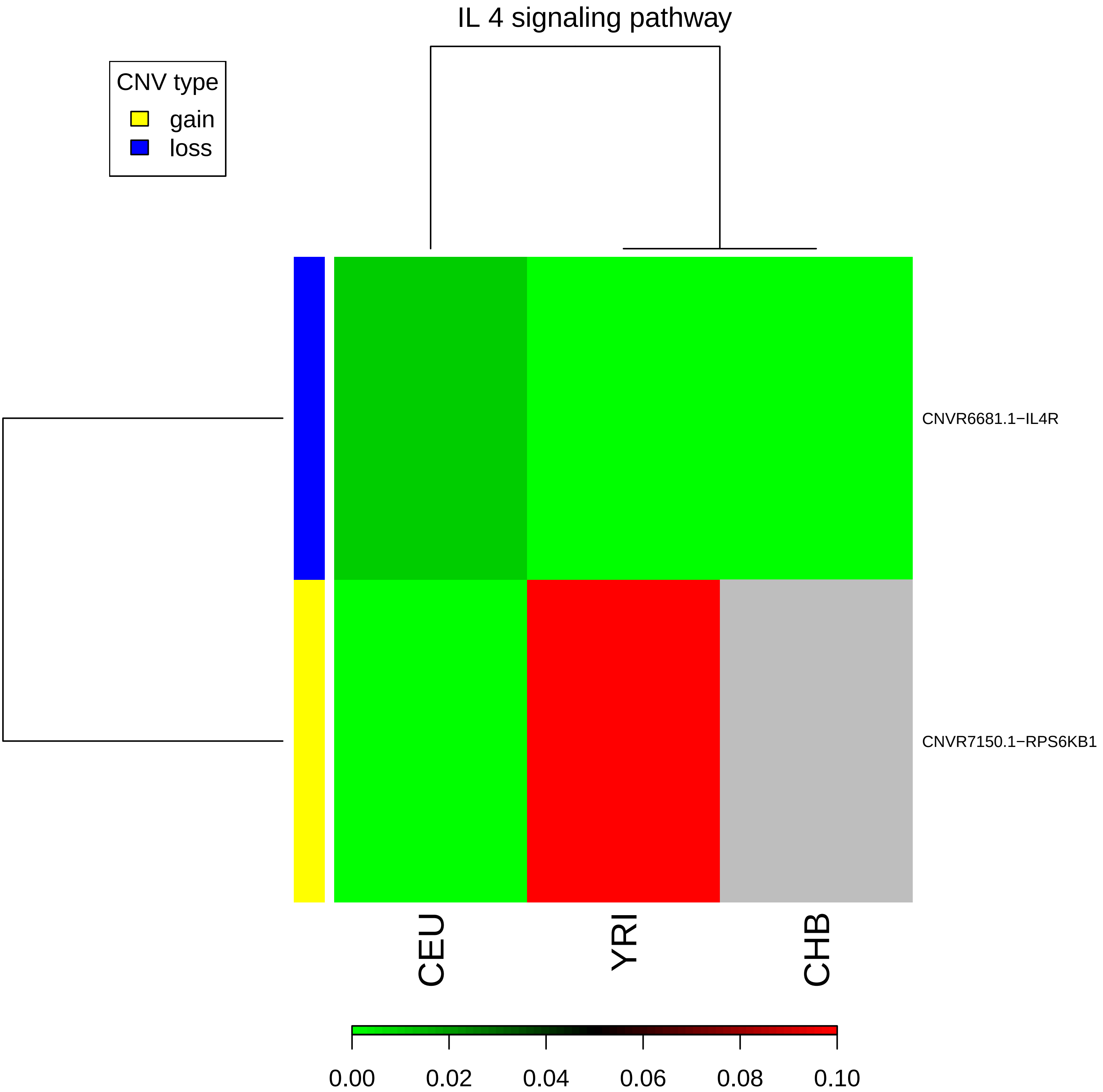

IL 4 signaling pathway
CNV type
gain
loss
CNVR6681.1−IL4R
CNVR7150.1−RPS6KB1
CEU
YRI
CHB
0.00
0.02
0.04
0.06
0.08
0.10

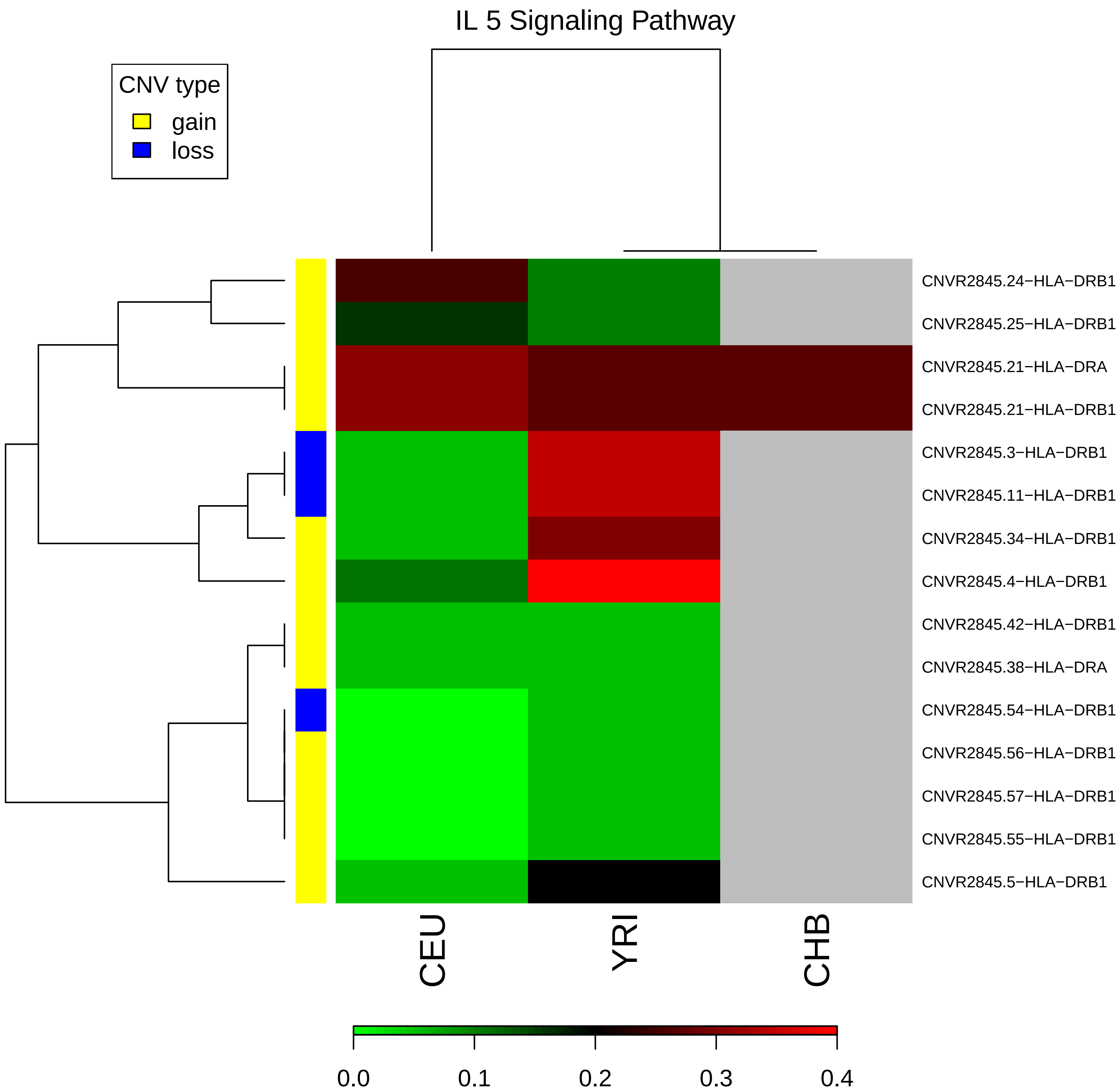
IL 5 Signaling Pathway
CNV type
gain
loss
CNVR2845.24−HLA−DRB1
CNVR2845.25−HLA−DRB1
CNVR2845.21−HLA−DRA
CNVR2845.21−HLA−DRB1
CNVR2845.3−HLA−DRB1
CNVR2845.11−HLA−DRB1
CNVR2845.34−HLA−DRB1
CNVR2845.4−HLA−DRB1
CNVR2845.42−HLA−DRB1
CNVR2845.38−HLA−DRA
CNVR2845.54−HLA−DRB1
CNVR2845.56−HLA−DRB1
CNVR2845.57−HLA−DRB1
CNVR2845.55−HLA−DRB1
CNVR2845.5−HLA−DRB1
CEU
YRI
CHB
0.0
0.1
0.2
0.3
0.4

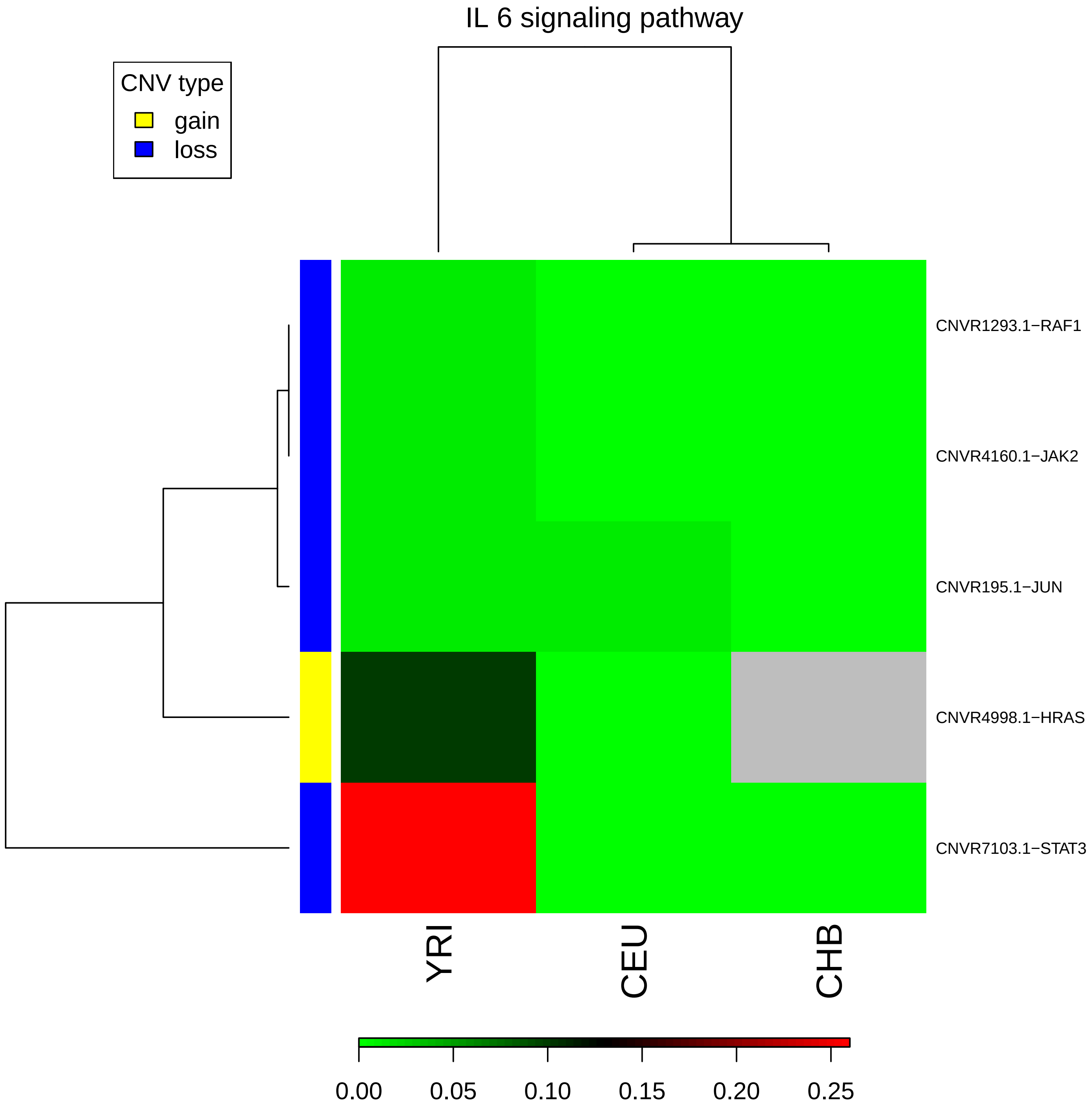
IL 6 signaling pathway
CNV type
gain
loss
CNVR1293.1−RAF1
CNVR4160.1−JAK2
CNVR195.1−JUN
CNVR4998.1−HRAS
CNVR7103.1−STAT3
YRI
CEU
CHB
0.00
0.05
0.10
0.15
0.20
0.25

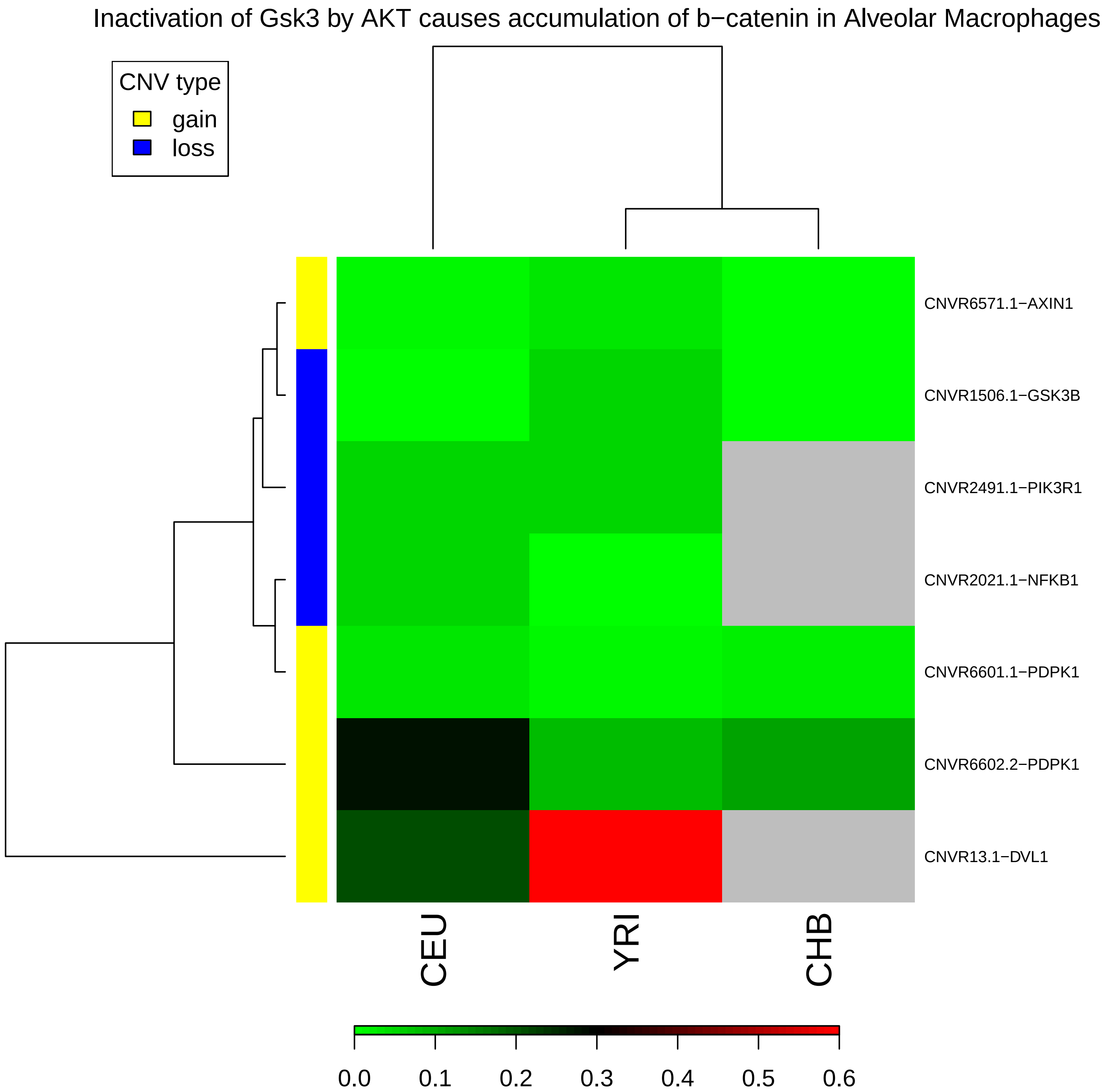

Inactivation of Gsk3 by AKT causes accumulation of b−catenin in Alveolar Macrophages
CNV type
gain
loss
CNVR6571.1−AXIN1
CNVR1506.1−GSK3B
CNVR2491.1−PIK3R1
CNVR2021.1−NFKB1
CNVR6601.1−PDPK1
CNVR6602.2−PDPK1
CNVR13.1−DVL1
CEU
YRI
CHB
0.0
0.1
0.2
0.3
0.4
0.5
0.6

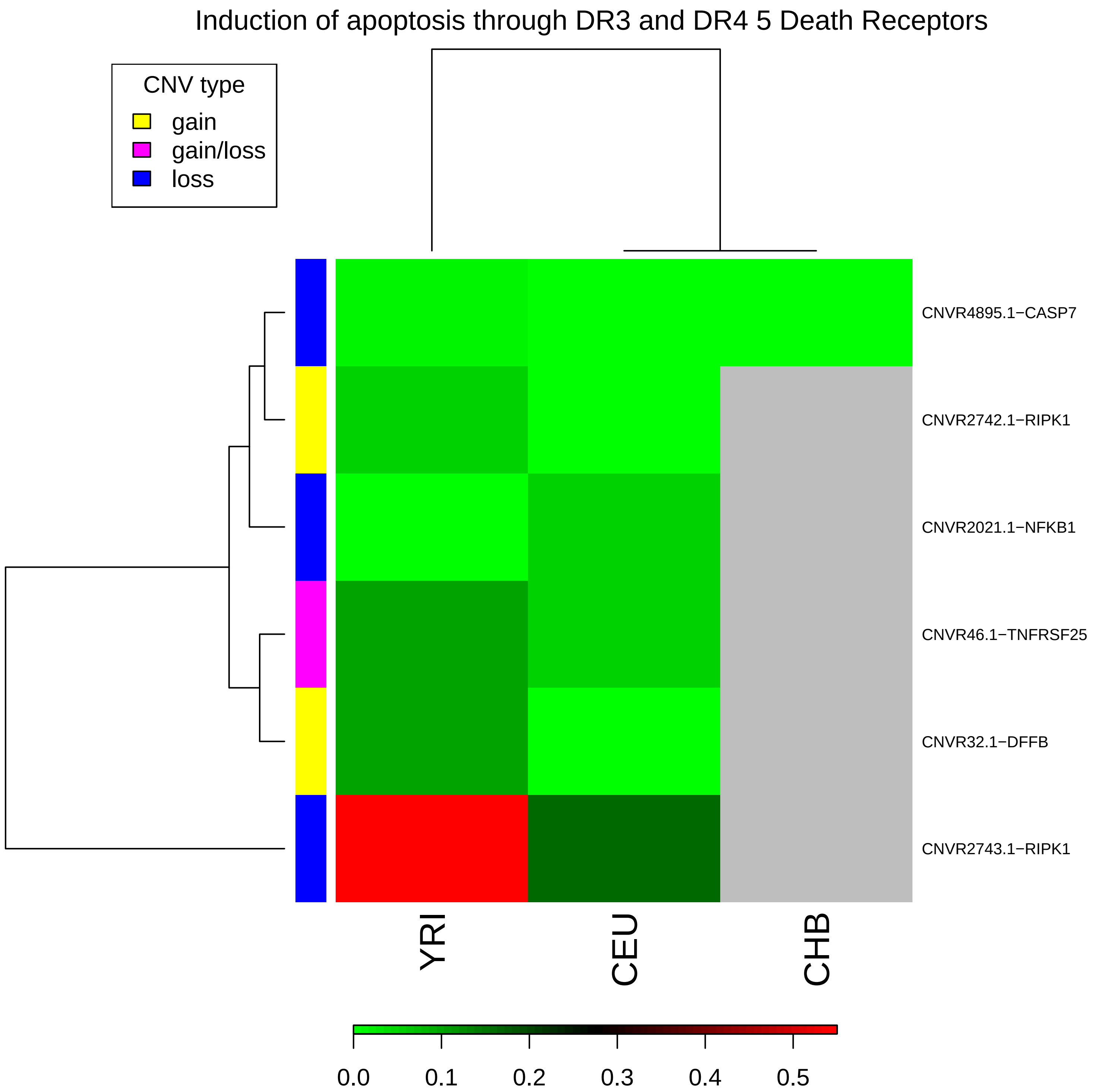

Induction of apoptosis through DR3 and DR4 5 Death Receptors
CNV type
gain
gain/loss
loss
CNVR4895.1−CASP7
CNVR2742.1−RIPK1
CNVR2021.1−NFKB1
CNVR46.1−TNFRSF25
CNVR32.1−DFFB
CNVR2743.1−RIPK1
YRI
CEU
CHB
0.0
0.1
0.2
0.3
0.4
0.5

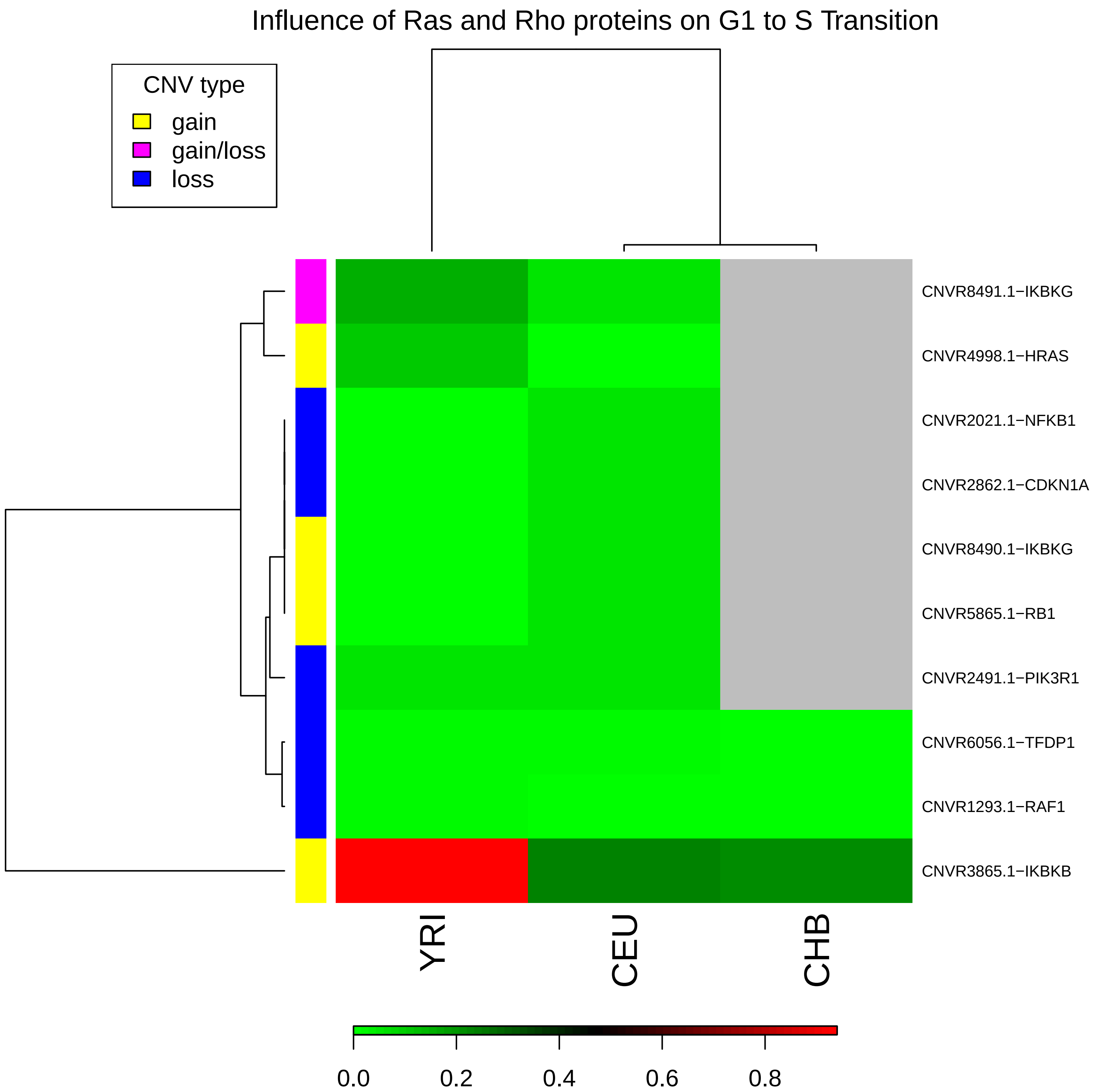

Influence of Ras and Rho proteins on G1 to S Transition
CNV type
gain
gain/loss
loss
CNVR8491.1−IKBKG
CNVR4998.1−HRAS
CNVR2021.1−NFKB1
CNVR2862.1−CDKN1A
CNVR8490.1−IKBKG
CNVR5865.1−RB1
CNVR2491.1−PIK3R1
CNVR6056.1−TFDP1
CNVR1293.1−RAF1
CNVR3865.1−IKBKB
YRI
CEU
CHB
0.0
0.2
0.4
0.6
0.8

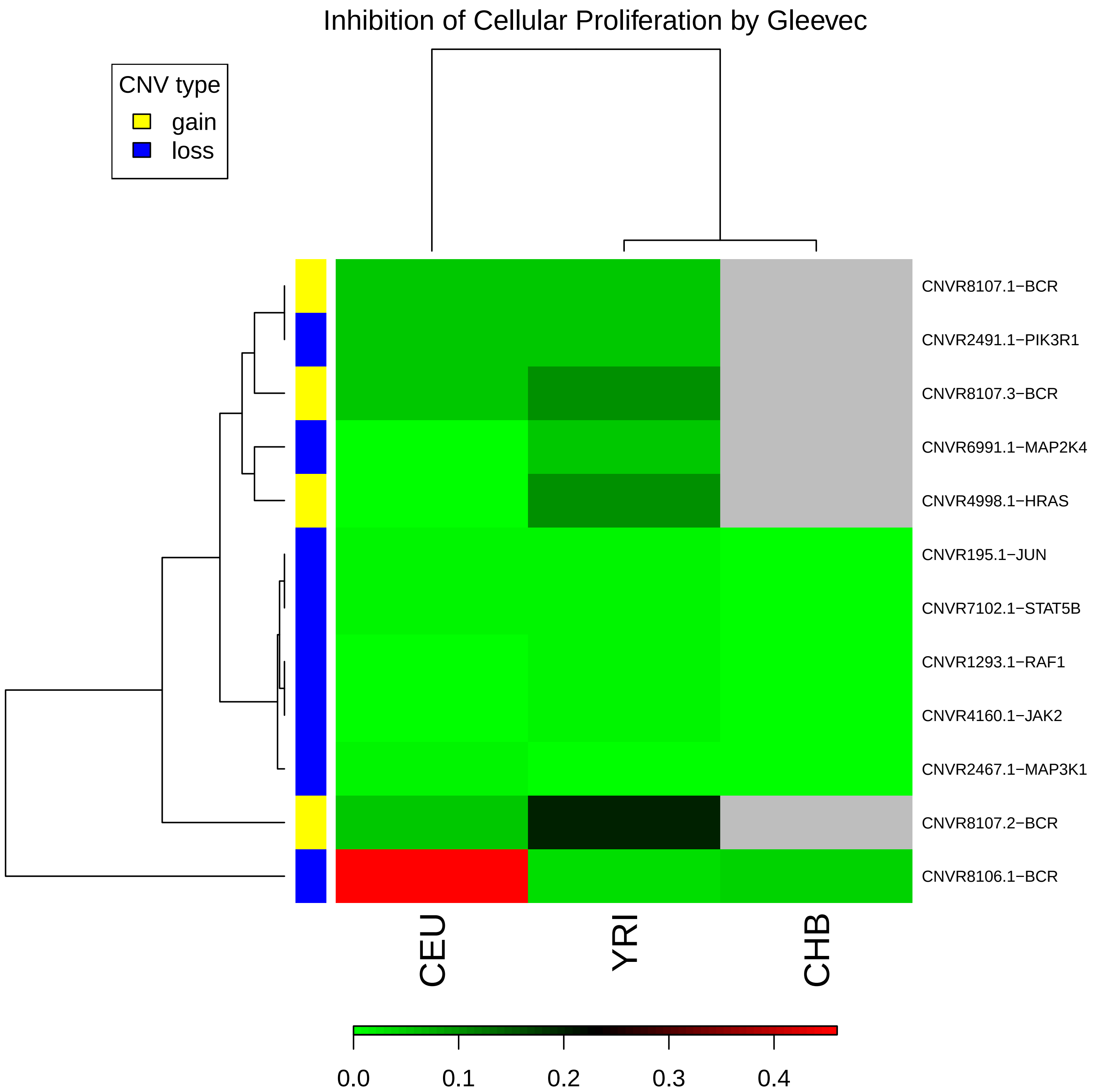
Inhibition of Cellular Proliferation by Gleevec
CNV type
gain
loss
CNVR8107.1−BCR
CNVR2491.1−PIK3R1
CNVR8107.3−BCR
CNVR6991.1−MAP2K4
CNVR4998.1−HRAS
CNVR195.1−JUN
CNVR7102.1−STAT5B
CNVR1293.1−RAF1
CNVR4160.1−JAK2
CNVR2467.1−MAP3K1
CNVR8107.2−BCR
CNVR8106.1−BCR
CEU
YRI
CHB
0.0
0.1
0.2
0.3
0.4

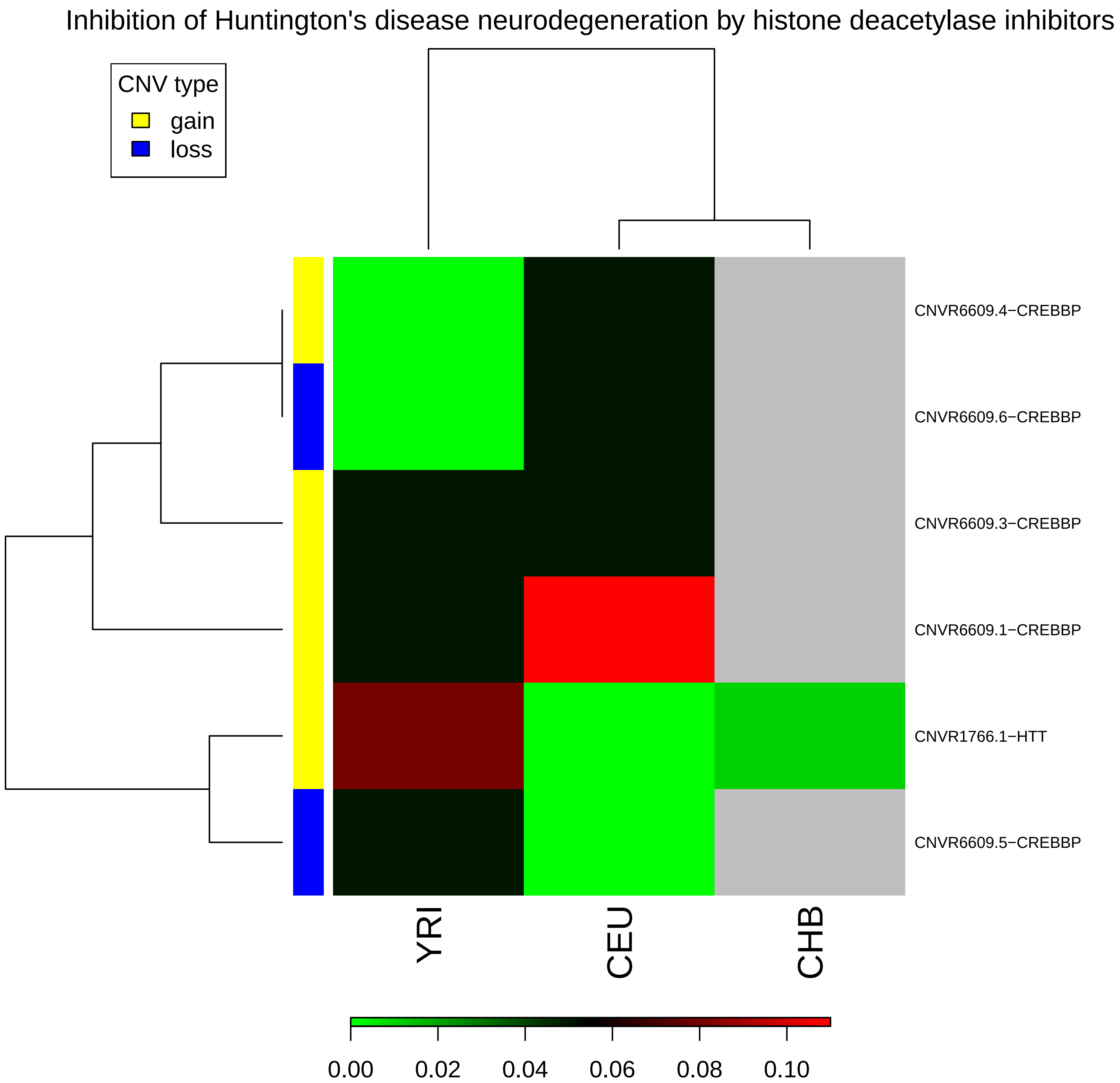

Inhibition of Huntington's disease neurodegeneration by histone deacetylase inhibitors
CNV type
gain
loss
CNVR6609.4−CREBBP
CNVR6609.6−CREBBP
CNVR6609.3−CREBBP
CNVR6609.1−CREBBP
CNVR1766.1−HTT
CNVR6609.5−CREBBP
YRI
CEU
CHB
0.00
0.02
0.04
0.06
0.08
0.10

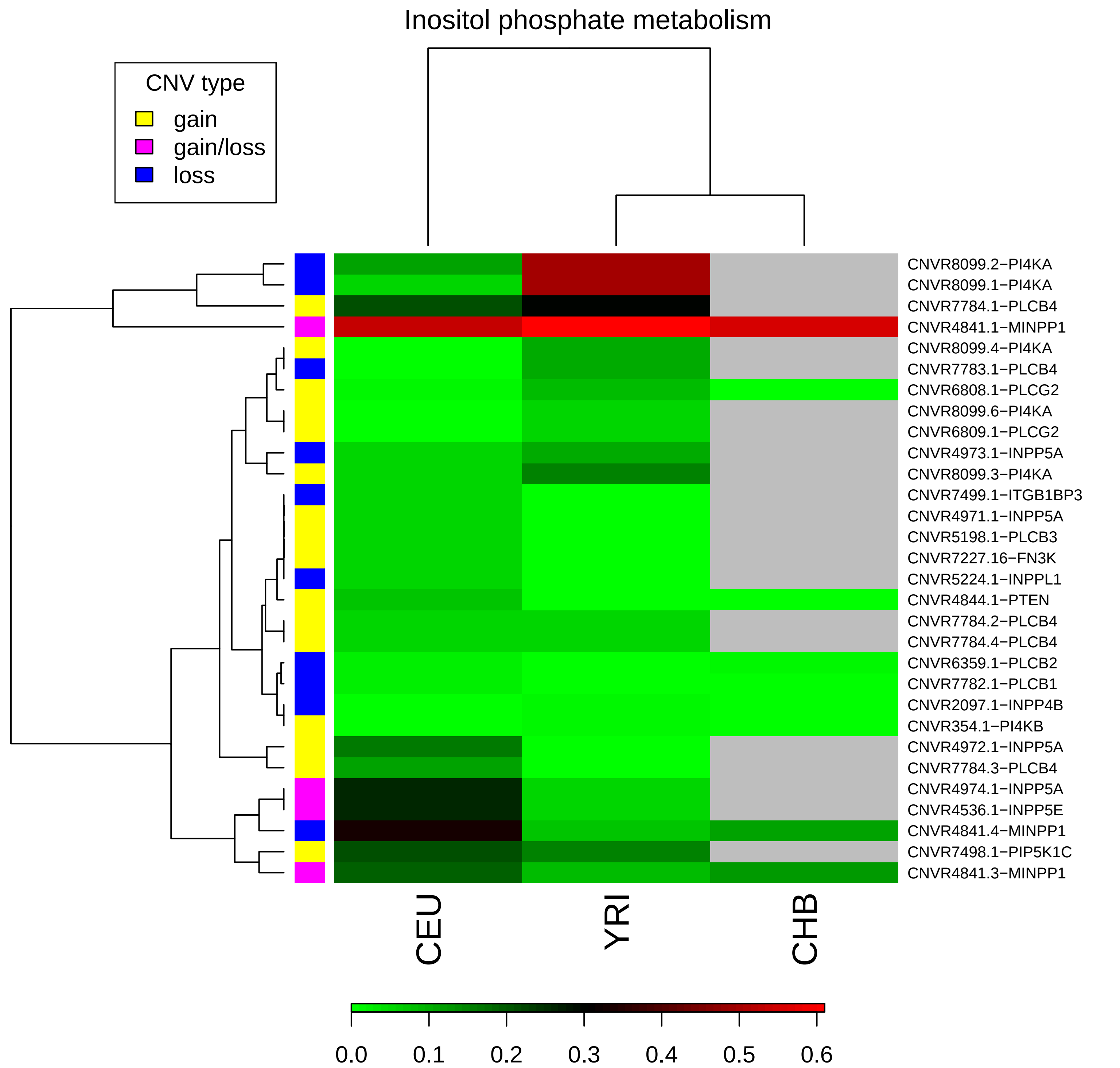
Inositol phosphate metabolism
CNV type
gain
gain/loss
loss
CNVR8099.2−PI4KA
CNVR8099.1−PI4KA
CNVR7784.1−PLCB4
CNVR4841.1−MINPP1
CNVR8099.4−PI4KA
CNVR7783.1−PLCB4
CNVR6808.1−PLCG2
CNVR8099.6−PI4KA
CNVR6809.1−PLCG2
CNVR4973.1−INPP5A
CNVR8099.3−PI4KA
CNVR7499.1−ITGB1BP3
CNVR4971.1−INPP5A
CNVR5198.1−PLCB3
CNVR7227.16−FN3K
CNVR5224.1−INPPL1
CNVR4844.1−PTEN
CNVR7784.2−PLCB4
CNVR7784.4−PLCB4
CNVR6359.1−PLCB2
CNVR7782.1−PLCB1
CNVR2097.1−INPP4B
CNVR354.1−PI4KB
CNVR4972.1−INPP5A
CNVR7784.3−PLCB4
CNVR4974.1−INPP5A
CNVR4536.1−INPP5E
CNVR4841.4−MINPP1
CNVR7498.1−PIP5K1C
CNVR4841.3−MINPP1
CEU
YRI
CHB
0.0
0.1
0.2
0.3
0.4
0.5
0.6

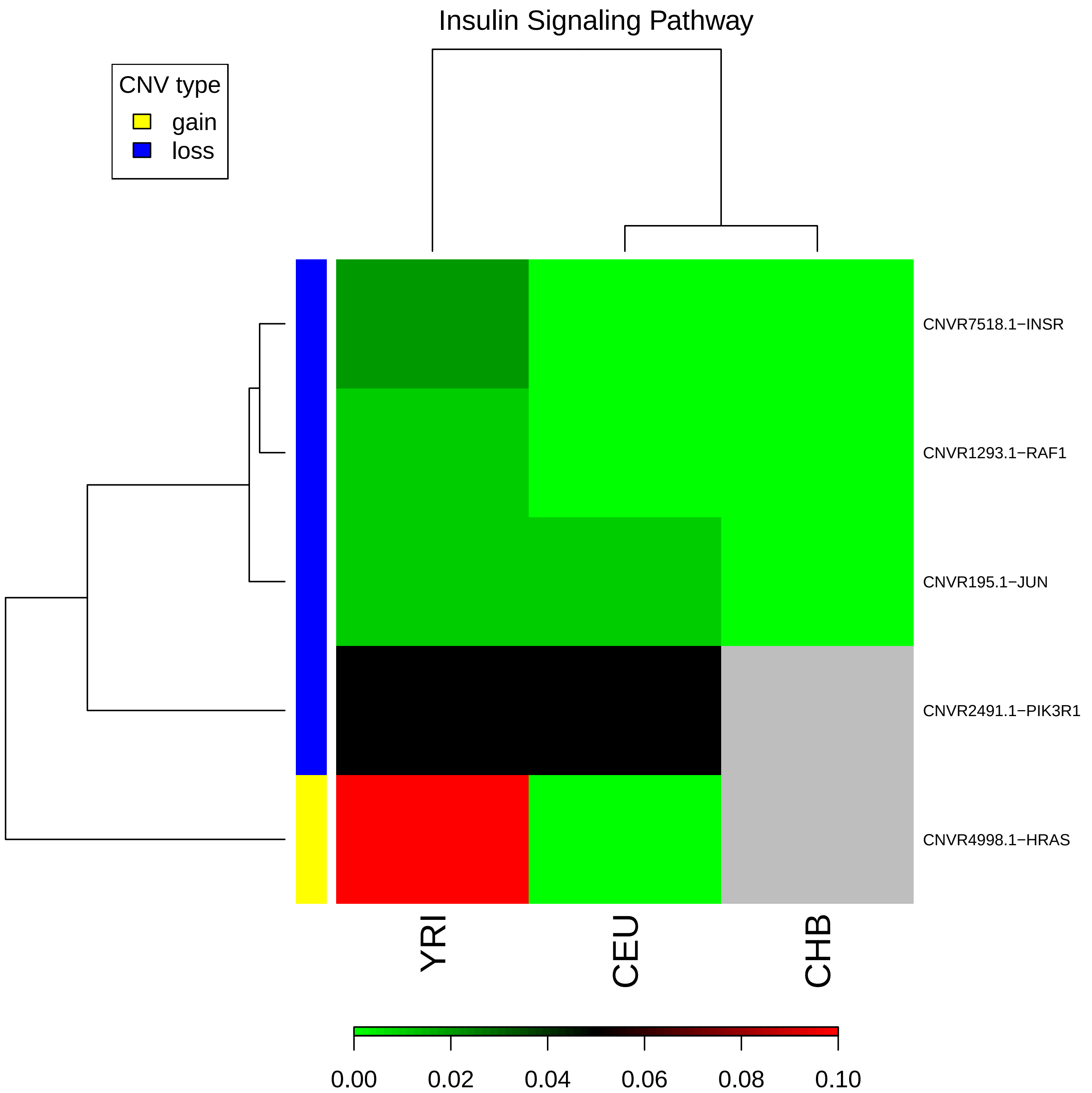
Insulin Signaling Pathway
CNV type
gain
loss
CNVR7518.1−INSR
CNVR1293.1−RAF1
CNVR195.1−JUN
CNVR2491.1−PIK3R1
CNVR4998.1−HRAS
YRI
CEU
CHB
0.00
0.02
0.04
0.06
0.08
0.10

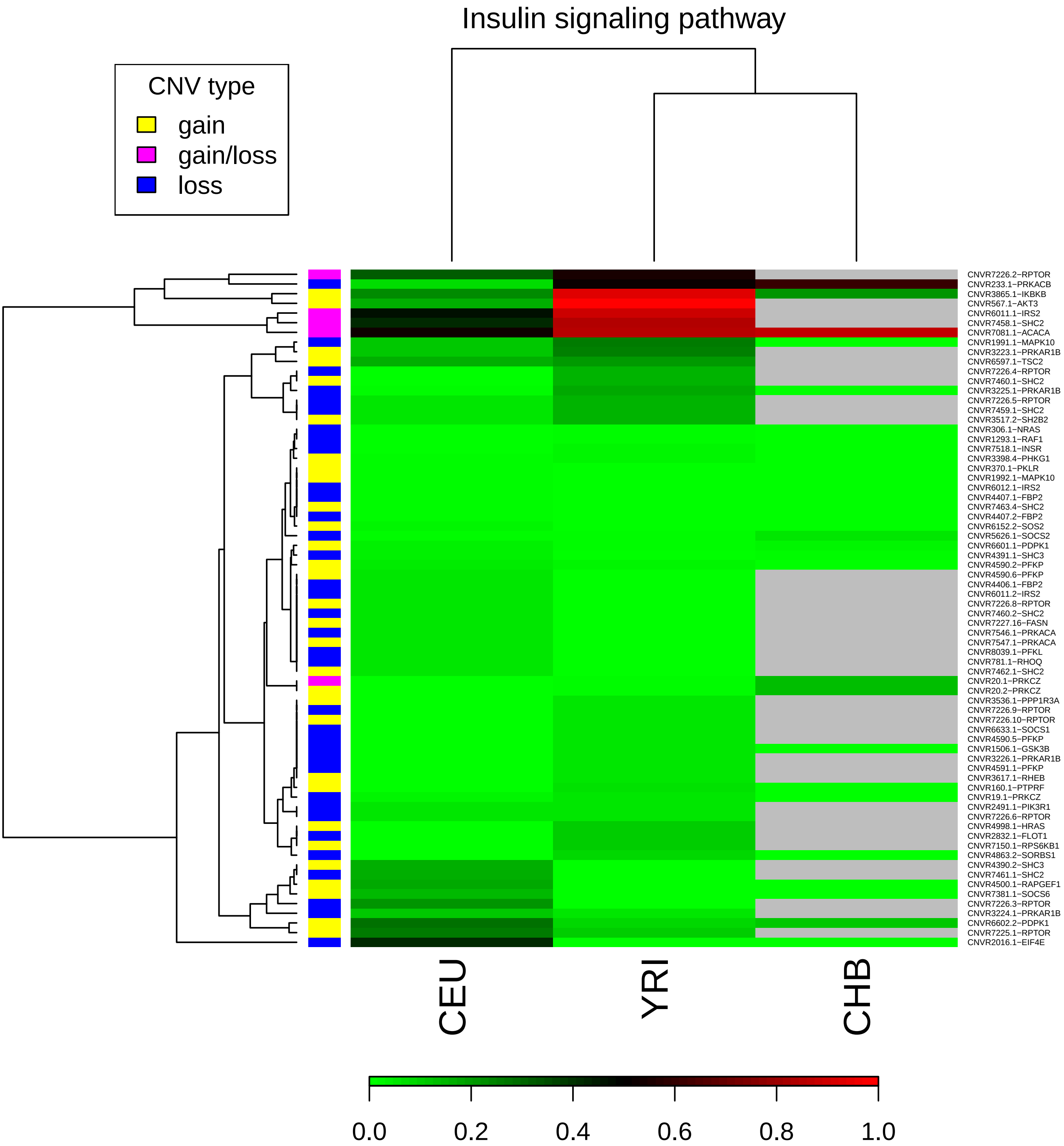

Insulin signaling pathway
CNV type
gain
gain/loss
loss
CNVR7226.2−RPTOR
CNVR233.1−PRKACB
CNVR3865.1−IKBKB
CNVR567.1−AKT3
CNVR6011.1−IRS2
CNVR7458.1−SHC2
CNVR7081.1−ACACA
CNVR1991.1−MAPK10
CNVR3223.1−PRKAR1B
CNVR6597.1−TSC2
CNVR7226.4−RPTOR
CNVR7460.1−SHC2
CNVR3225.1−PRKAR1B
CNVR7226.5−RPTOR
CNVR7459.1−SHC2
CNVR3517.2−SH2B2
CNVR306.1−NRAS
CNVR1293.1−RAF1
CNVR7518.1−INSR
CNVR3398.4−PHKG1
CNVR370.1−PKLR
CNVR1992.1−MAPK10
CNVR6012.1−IRS2
CNVR4407.1−FBP2
CNVR7463.4−SHC2
CNVR4407.2−FBP2
CNVR6152.2−SOS2
CNVR5626.1−SOCS2
CNVR6601.1−PDPK1
CNVR4391.1−SHC3
CNVR4590.2−PFKP
CNVR4590.6−PFKP
CNVR4406.1−FBP2
CNVR6011.2−IRS2
CNVR7226.8−RPTOR
CNVR7460.2−SHC2
CNVR7227.16−FASN
CNVR7546.1−PRKACA
CNVR7547.1−PRKACA
CNVR8039.1−PFKL
CNVR781.1−RHOQ
CNVR7462.1−SHC2
CNVR20.1−PRKCZ
CNVR20.2−PRKCZ
CNVR3536.1−PPP1R3A
CNVR7226.9−RPTOR
CNVR7226.10−RPTOR
CNVR6633.1−SOCS1
CNVR4590.5−PFKP
CNVR1506.1−GSK3B
CNVR3226.1−PRKAR1B
CNVR4591.1−PFKP
CNVR3617.1−RHEB
CNVR160.1−PTPRF
CNVR19.1−PRKCZ
CNVR2491.1−PIK3R1
CNVR7226.6−RPTOR
CNVR4998.1−HRAS
CNVR2832.1−FLOT1
CNVR7150.1−RPS6KB1
CNVR4863.2−SORBS1
CNVR4390.2−SHC3
CNVR7461.1−SHC2
CNVR4500.1−RAPGEF1
CNVR7381.1−SOCS6
CNVR7226.3−RPTOR
CNVR3224.1−PRKAR1B
CNVR6602.2−PDPK1
CNVR7225.1−RPTOR
CNVR2016.1−EIF4E
CEU
YRI
CHB
0.0
0.2
0.4
0.6
0.8
1.0

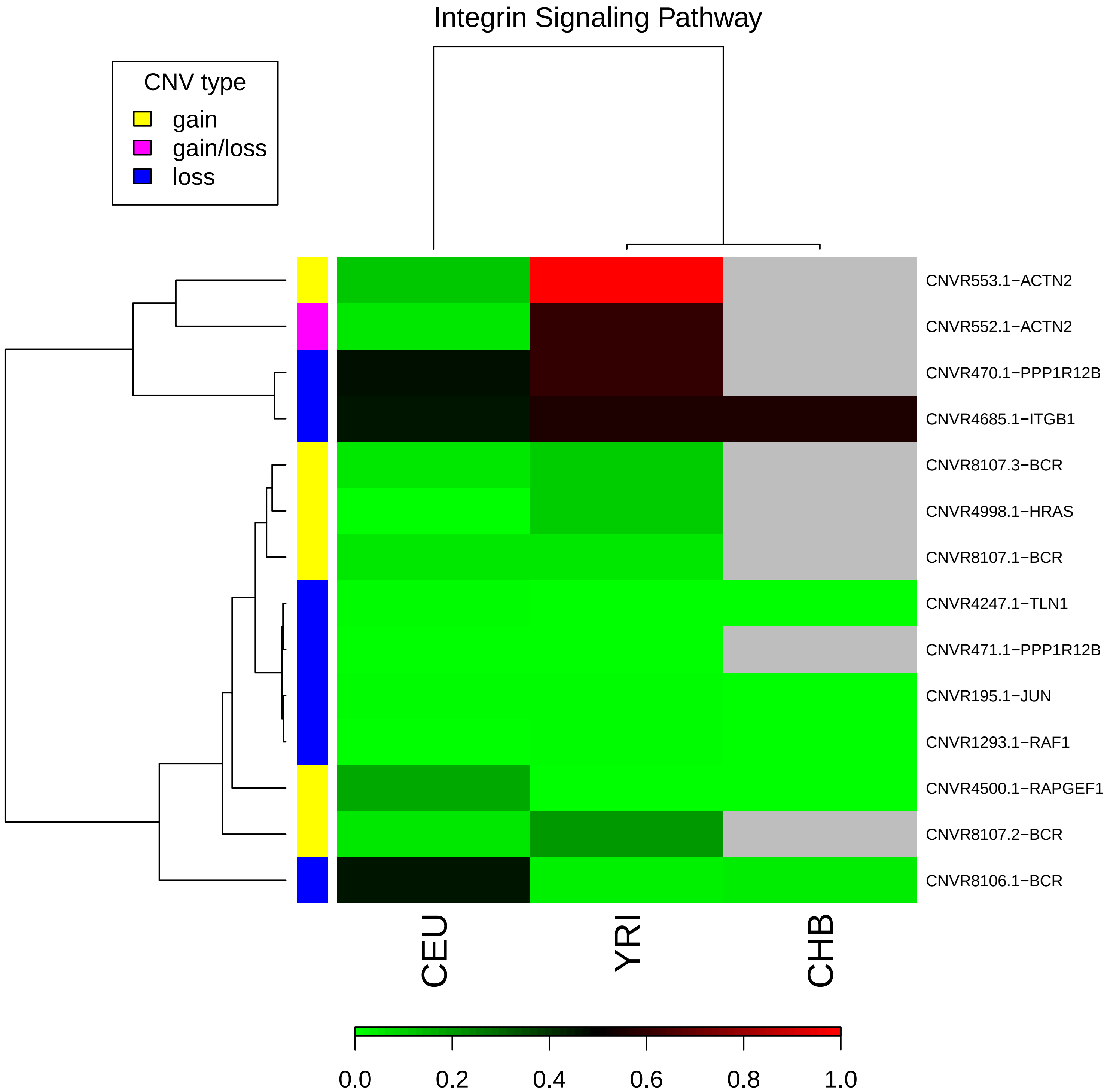
Integrin Signaling Pathway
CNV type
gain
gain/loss
loss
CNVR553.1−ACTN2
CNVR552.1−ACTN2
CNVR470.1−PPP1R12B
CNVR4685.1−ITGB1
CNVR8107.3−BCR
CNVR4998.1−HRAS
CNVR8107.1−BCR
CNVR4247.1−TLN1
CNVR471.1−PPP1R12B
CNVR195.1−JUN
CNVR1293.1−RAF1
CNVR4500.1−RAPGEF1
CNVR8107.2−BCR
CNVR8106.1−BCR
CEU
YRI
CHB
0.0
0.2
0.4
0.6
0.8
1.0

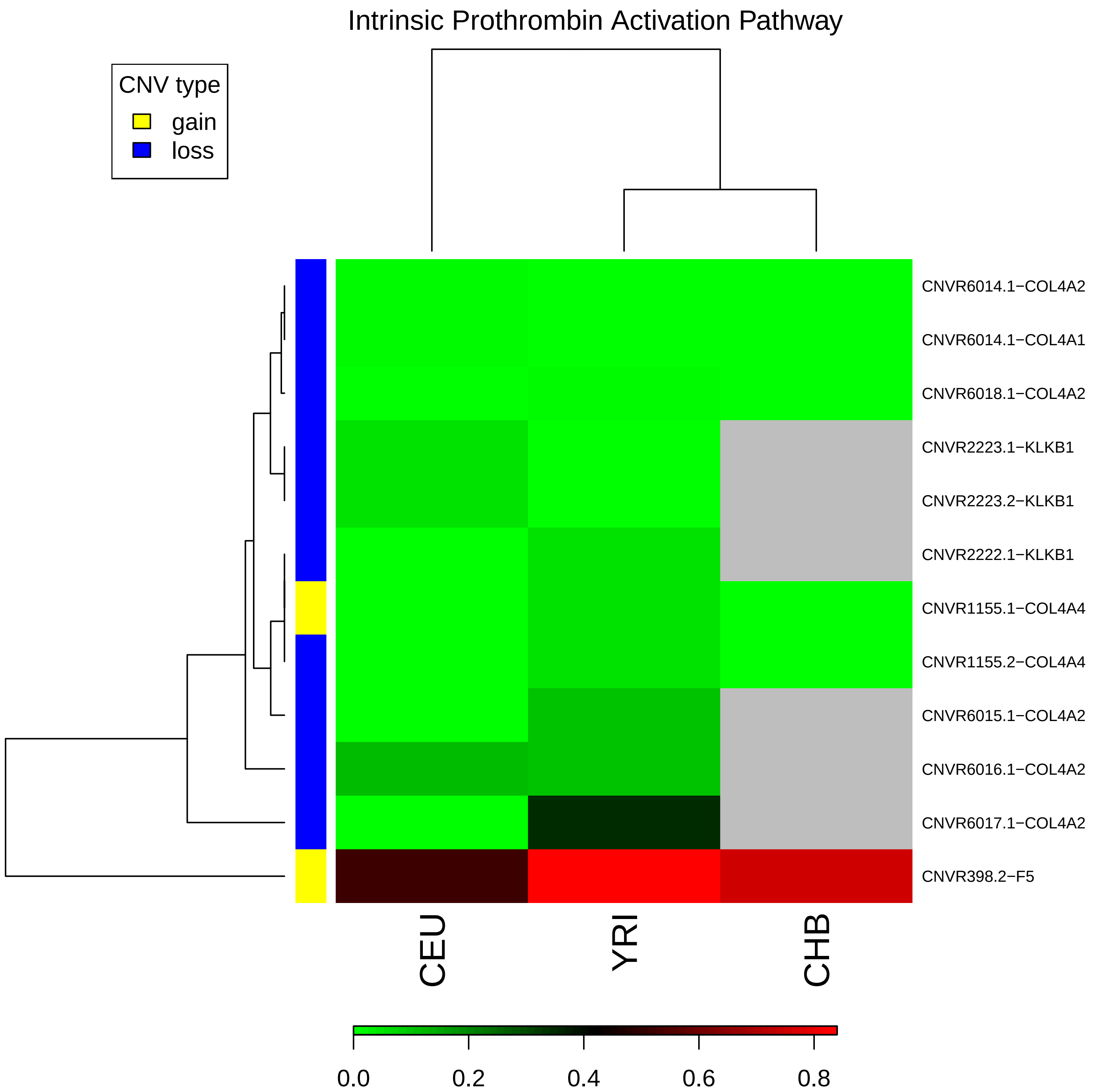
Intrinsic Prothrombin Activation Pathway
CNV type
gain
loss
CNVR6014.1−COL4A2
CNVR6014.1−COL4A1
CNVR6018.1−COL4A2
CNVR2223.1−KLKB1
CNVR2223.2−KLKB1
CNVR2222.1−KLKB1
CNVR1155.1−COL4A4
CNVR1155.2−COL4A4
CNVR6015.1−COL4A2
CNVR6016.1−COL4A2
CNVR6017.1−COL4A2
CNVR398.2−F5
CEU
YRI
CHB
0.0
0.2
0.4
0.6
0.8

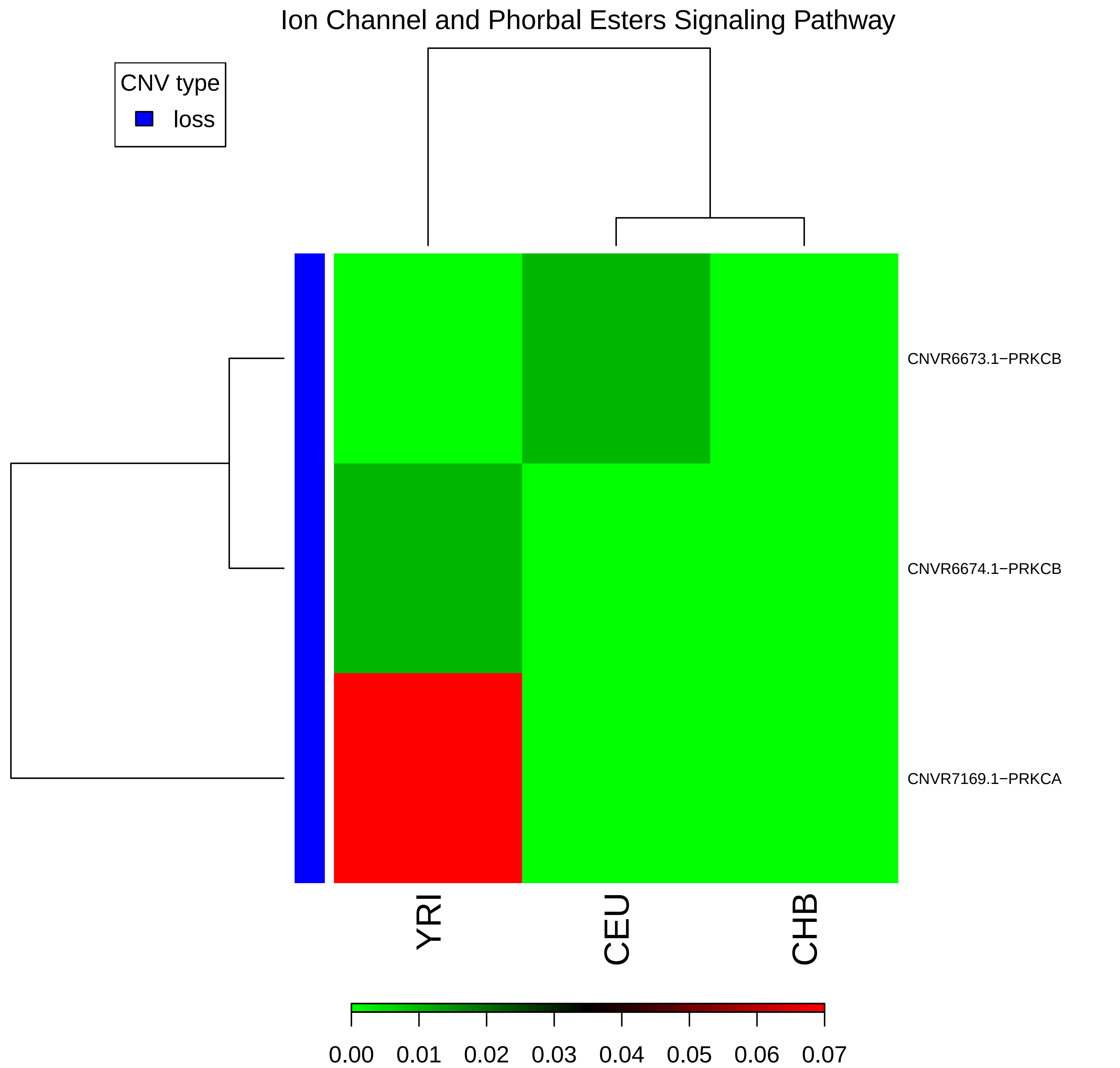

Ion Channel and Phorbal Esters Signaling Pathway
CNV type
loss
CNVR6673.1−PRKCB
CNVR6674.1−PRKCB
CNVR7169.1−PRKCA
YRI
CEU
CHB
0.00
0.01
0.02
0.03
0.04
0.05
0.06
0.07

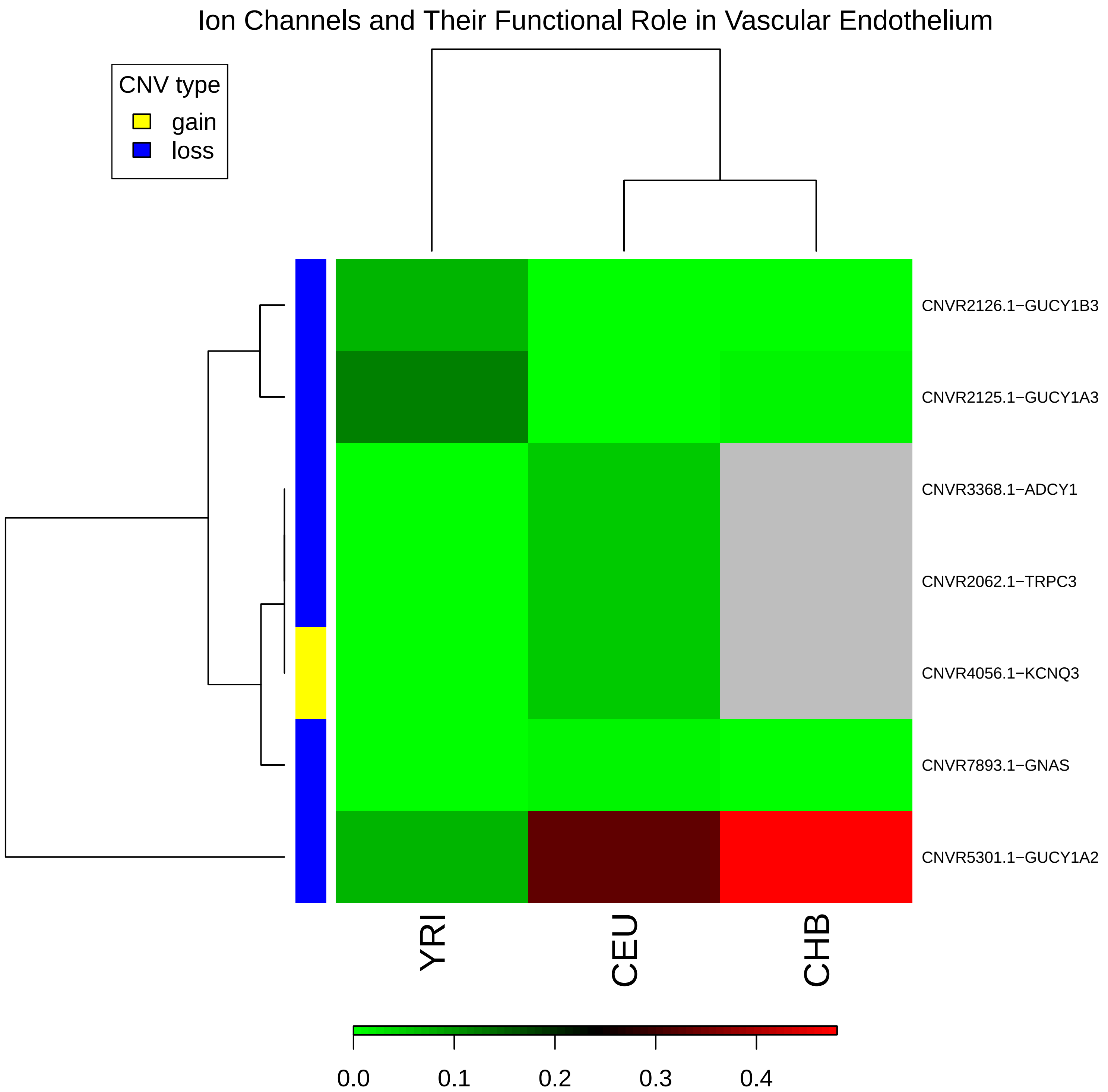
Ion Channels and Their Functional Role in Vascular Endothelium
CNV type
gain
loss
CNVR2126.1−GUCY1B3
CNVR2125.1−GUCY1A3
CNVR3368.1−ADCY1
CNVR2062.1−TRPC3
CNVR4056.1−KCNQ3
CNVR7893.1−GNAS
CNVR5301.1−GUCY1A2
YRI
CEU
CHB
0.0
0.1
0.2
0.3
0.4

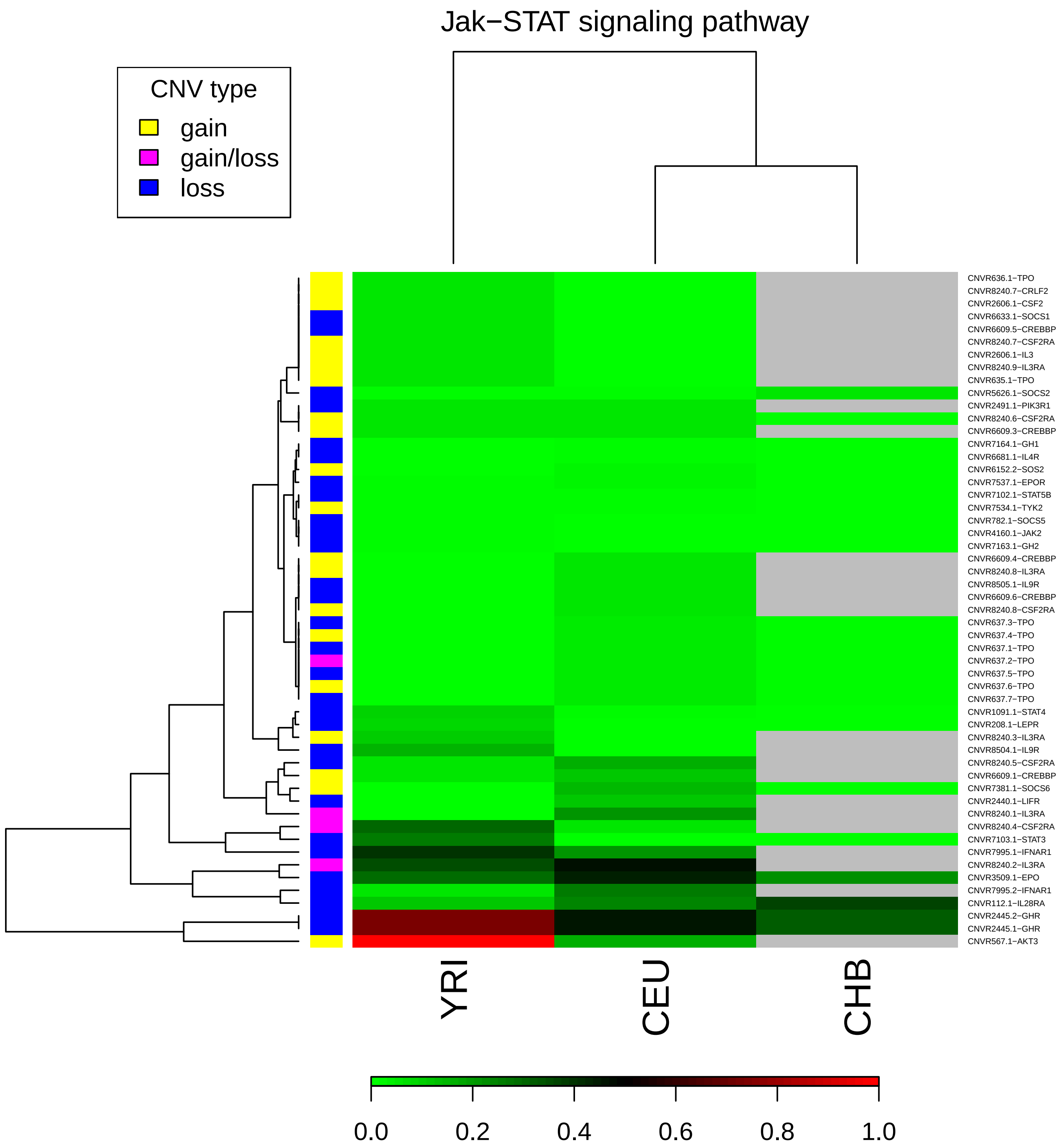
Jak−STAT signaling pathway
CNV type
gain
gain/loss
loss
CNVR636.1−TPO
CNVR8240.7−CRLF2
CNVR2606.1−CSF2
CNVR6633.1−SOCS1
CNVR6609.5−CREBBP
CNVR8240.7−CSF2RA
CNVR2606.1−IL3
CNVR8240.9−IL3RA
CNVR635.1−TPO
CNVR5626.1−SOCS2
CNVR2491.1−PIK3R1
CNVR8240.6−CSF2RA
CNVR6609.3−CREBBP
CNVR7164.1−GH1
CNVR6681.1−IL4R
CNVR6152.2−SOS2
CNVR7537.1−EPOR
CNVR7102.1−STAT5B
CNVR7534.1−TYK2
CNVR782.1−SOCS5
CNVR4160.1−JAK2
CNVR7163.1−GH2
CNVR6609.4−CREBBP
CNVR8240.8−IL3RA
CNVR8505.1−IL9R
CNVR6609.6−CREBBP
CNVR8240.8−CSF2RA
CNVR637.3−TPO
CNVR637.4−TPO
CNVR637.1−TPO
CNVR637.2−TPO
CNVR637.5−TPO
CNVR637.6−TPO
CNVR637.7−TPO
CNVR1091.1−STAT4
CNVR208.1−LEPR
CNVR8240.3−IL3RA
CNVR8504.1−IL9R
CNVR8240.5−CSF2RA
CNVR6609.1−CREBBP
CNVR7381.1−SOCS6
CNVR2440.1−LIFR
CNVR8240.1−IL3RA
CNVR8240.4−CSF2RA
CNVR7103.1−STAT3
CNVR7995.1−IFNAR1
CNVR8240.2−IL3RA
CNVR3509.1−EPO
CNVR7995.2−IFNAR1
CNVR112.1−IL28RA
CNVR2445.2−GHR
CNVR2445.1−GHR
CNVR567.1−AKT3
YRI
CEU
CHB
0.0
0.2
0.4
0.6
0.8
1.0

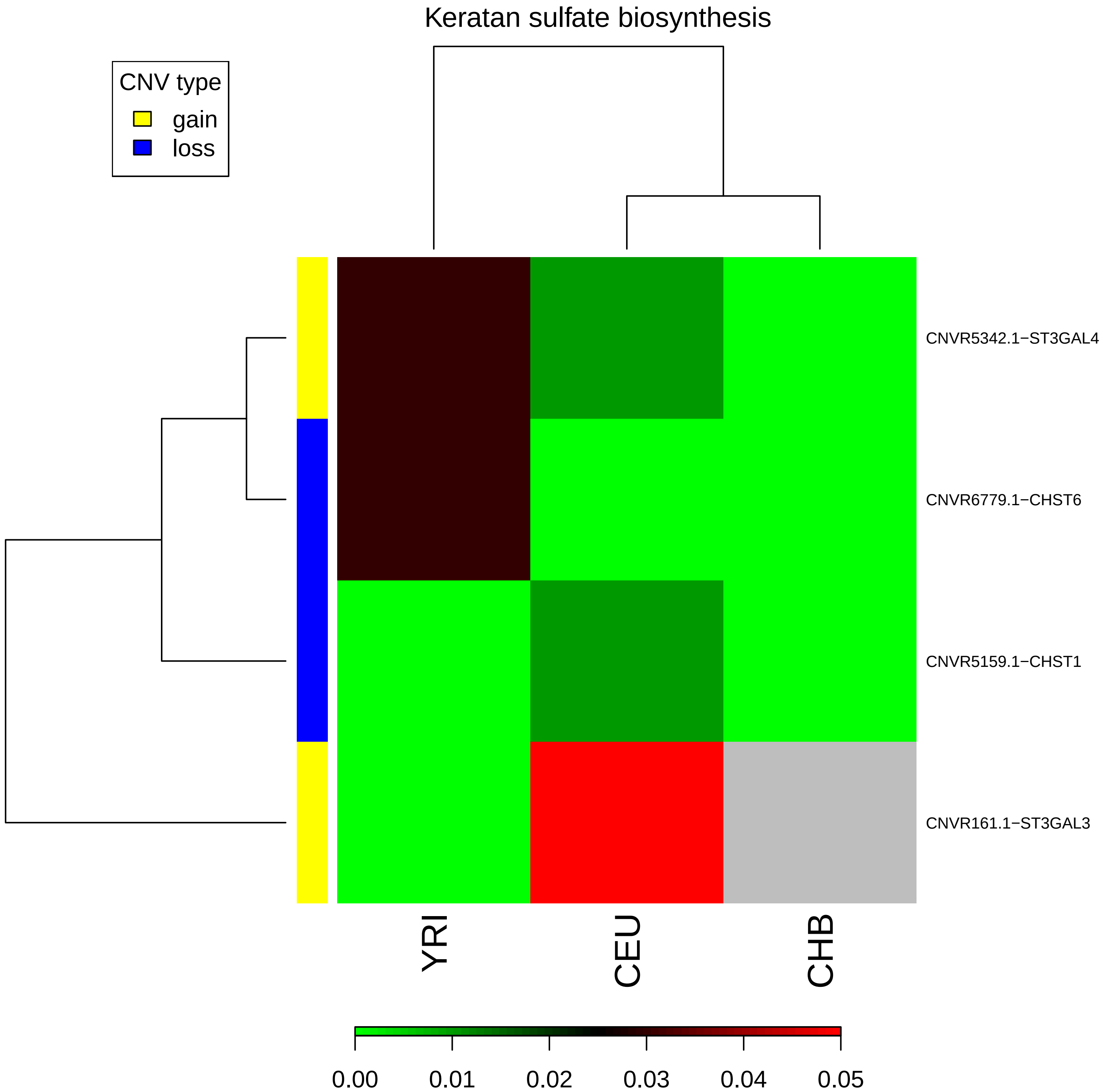
Keratan sulfate biosynthesis
CNV type
gain
loss
CNVR5342.1−ST3GAL4
CNVR6779.1−CHST6
CNVR5159.1−CHST1
CNVR161.1−ST3GAL3
YRI
CEU
CHB
0.00
0.01
0.02
0.03
0.04
0.05

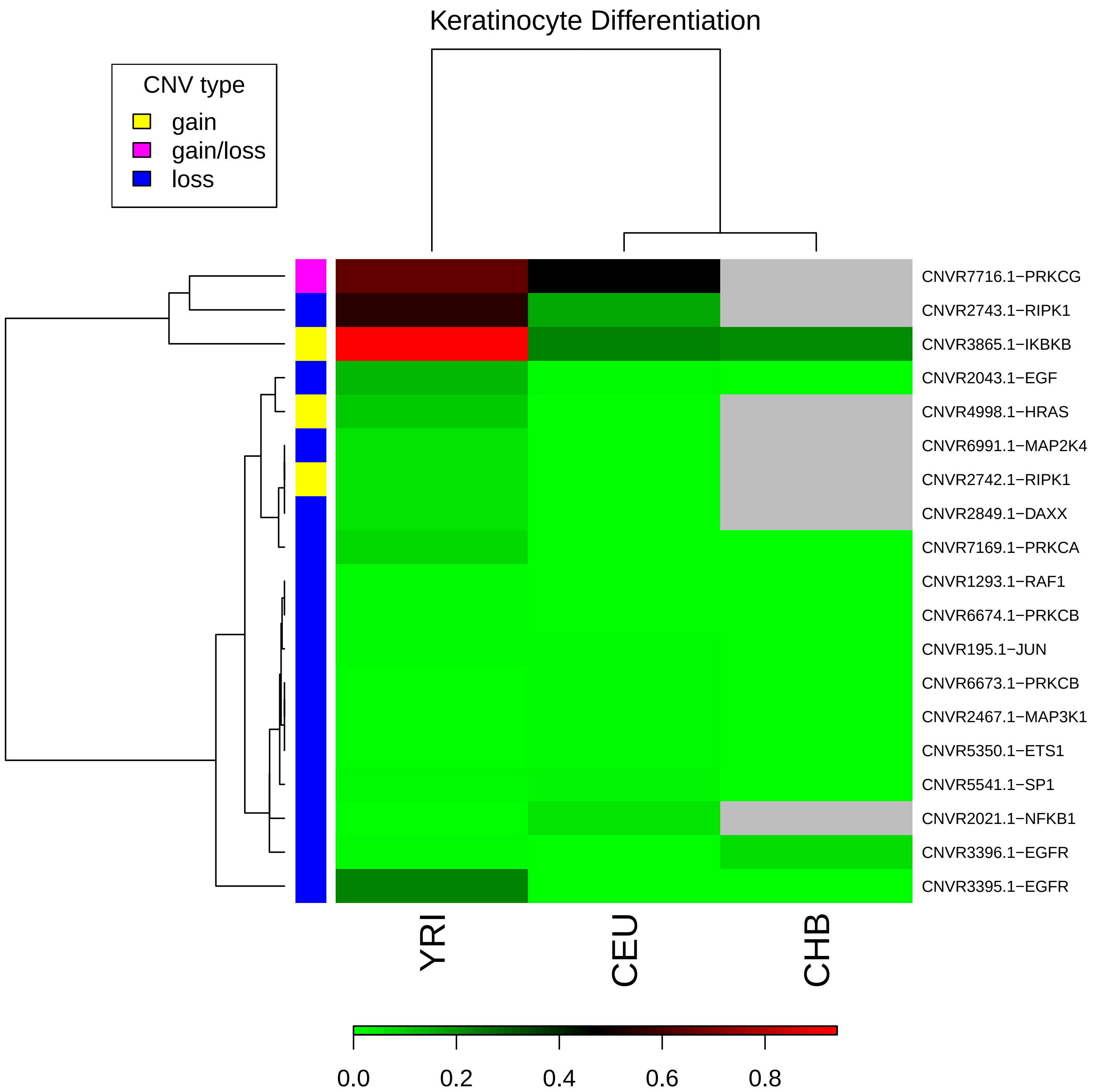
Keratinocyte Differentiation
CNV type
gain
gain/loss
loss
CNVR7716.1−PRKCG
CNVR2743.1−RIPK1
CNVR3865.1−IKBKB
CNVR2043.1−EGF
CNVR4998.1−HRAS
CNVR6991.1−MAP2K4
CNVR2742.1−RIPK1
CNVR2849.1−DAXX
CNVR7169.1−PRKCA
CNVR1293.1−RAF1
CNVR6674.1−PRKCB
CNVR195.1−JUN
CNVR6673.1−PRKCB
CNVR2467.1−MAP3K1
CNVR5350.1−ETS1
CNVR5541.1−SP1
CNVR2021.1−NFKB1
CNVR3396.1−EGFR
CNVR3395.1−EGFR
YRI
CEU
CHB
0.0
0.2
0.4
0.6
0.8

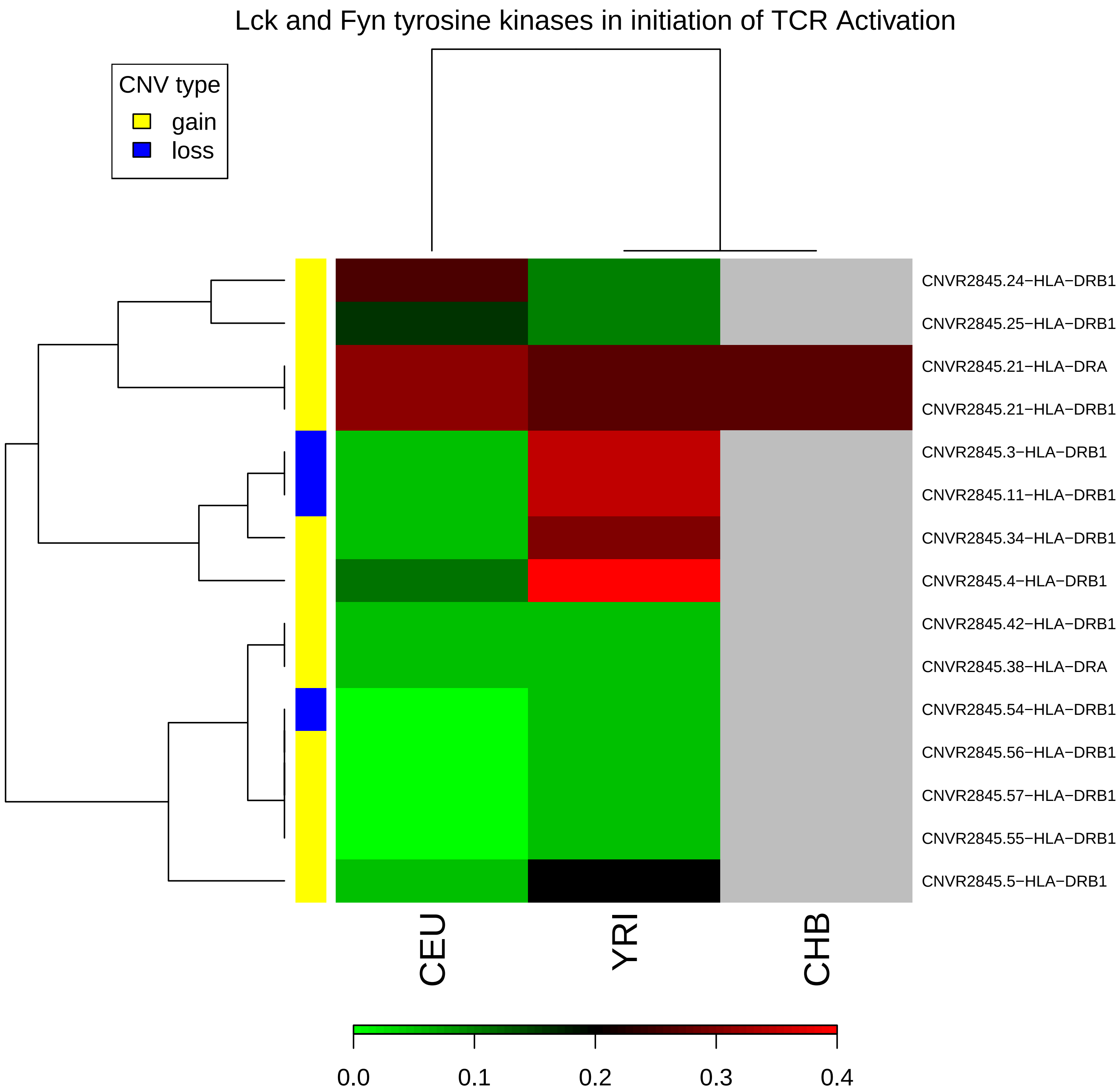
Lck and Fyn tyrosine kinases in initiation of TCR Activation
CNV type
gain
loss
CNVR2845.24−HLA−DRB1
CNVR2845.25−HLA−DRB1
CNVR2845.21−HLA−DRA
CNVR2845.21−HLA−DRB1
CNVR2845.3−HLA−DRB1
CNVR2845.11−HLA−DRB1
CNVR2845.34−HLA−DRB1
CNVR2845.4−HLA−DRB1
CNVR2845.42−HLA−DRB1
CNVR2845.38−HLA−DRA
CNVR2845.54−HLA−DRB1
CNVR2845.56−HLA−DRB1
CNVR2845.57−HLA−DRB1
CNVR2845.55−HLA−DRB1
CNVR2845.5−HLA−DRB1
CEU
YRI
CHB
0.0
0.1
0.2
0.3
0.4

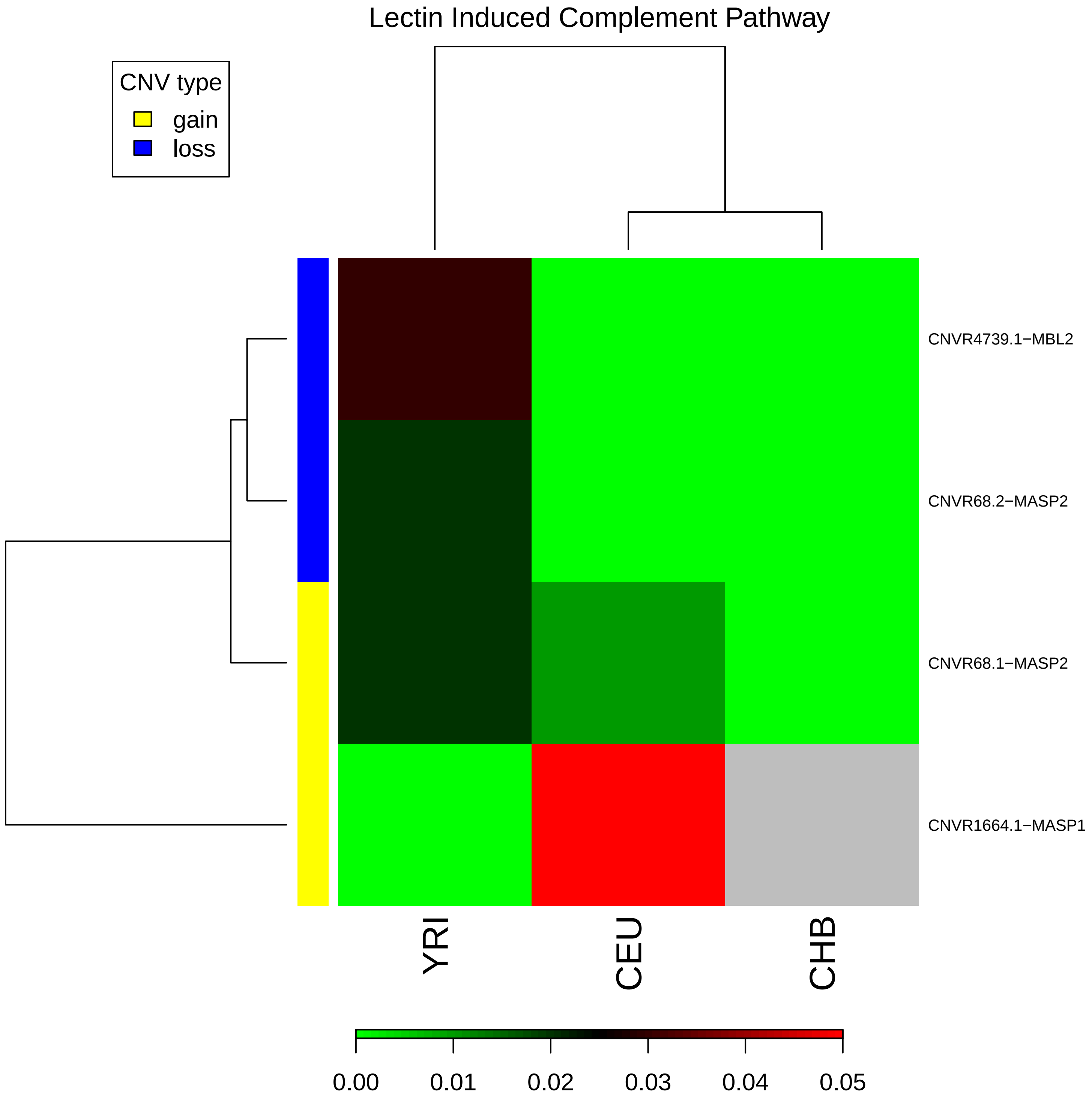
Lectin Induced Complement Pathway
CNV type
gain
loss
CNVR4739.1−MBL2
CNVR68.2−MASP2
CNVR68.1−MASP2
CNVR1664.1−MASP1
YRI
CEU
CHB
0.00
0.01
0.02
0.03
0.04
0.05

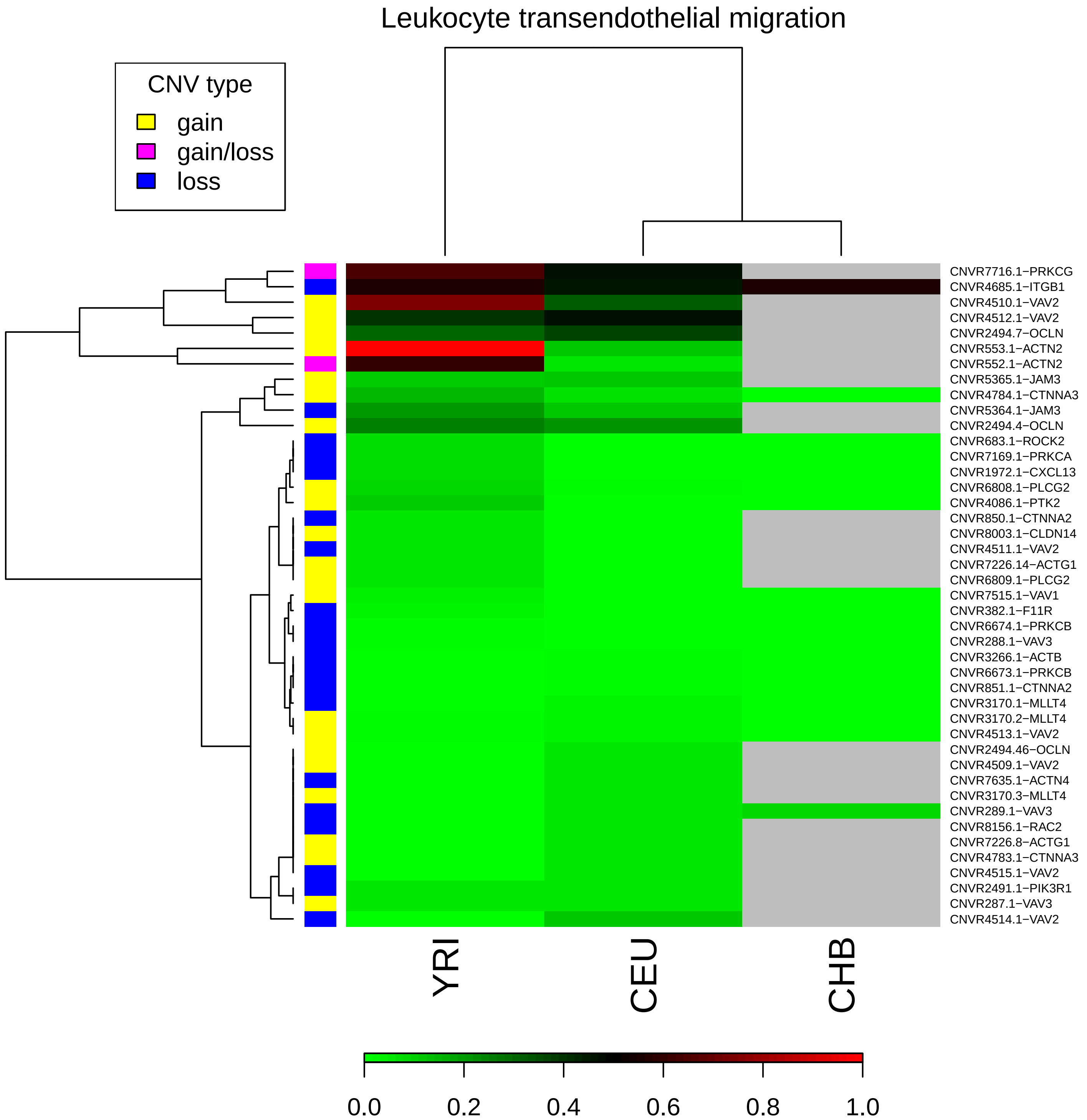

Leukocyte transendothelial migration
CNV type
gain
gain/loss
loss
CNVR7716.1−PRKCG
CNVR4685.1−ITGB1
CNVR4510.1−VAV2
CNVR4512.1−VAV2
CNVR2494.7−OCLN
CNVR553.1−ACTN2
CNVR552.1−ACTN2
CNVR5365.1−JAM3
CNVR4784.1−CTNNA3
CNVR5364.1−JAM3
CNVR2494.4−OCLN
CNVR683.1−ROCK2
CNVR7169.1−PRKCA
CNVR1972.1−CXCL13
CNVR6808.1−PLCG2
CNVR4086.1−PTK2
CNVR850.1−CTNNA2
CNVR8003.1−CLDN14
CNVR4511.1−VAV2
CNVR7226.14−ACTG1
CNVR6809.1−PLCG2
CNVR7515.1−VAV1
CNVR382.1−F11R
CNVR6674.1−PRKCB
CNVR288.1−VAV3
CNVR3266.1−ACTB
CNVR6673.1−PRKCB
CNVR851.1−CTNNA2
CNVR3170.1−MLLT4
CNVR3170.2−MLLT4
CNVR4513.1−VAV2
CNVR2494.46−OCLN
CNVR4509.1−VAV2
CNVR7635.1−ACTN4
CNVR3170.3−MLLT4
CNVR289.1−VAV3
CNVR8156.1−RAC2
CNVR7226.8−ACTG1
CNVR4783.1−CTNNA3
CNVR4515.1−VAV2
CNVR2491.1−PIK3R1
CNVR287.1−VAV3
CNVR4514.1−VAV2
YRI
CEU
CHB
0.0
0.2
0.4
0.6
0.8
1.0

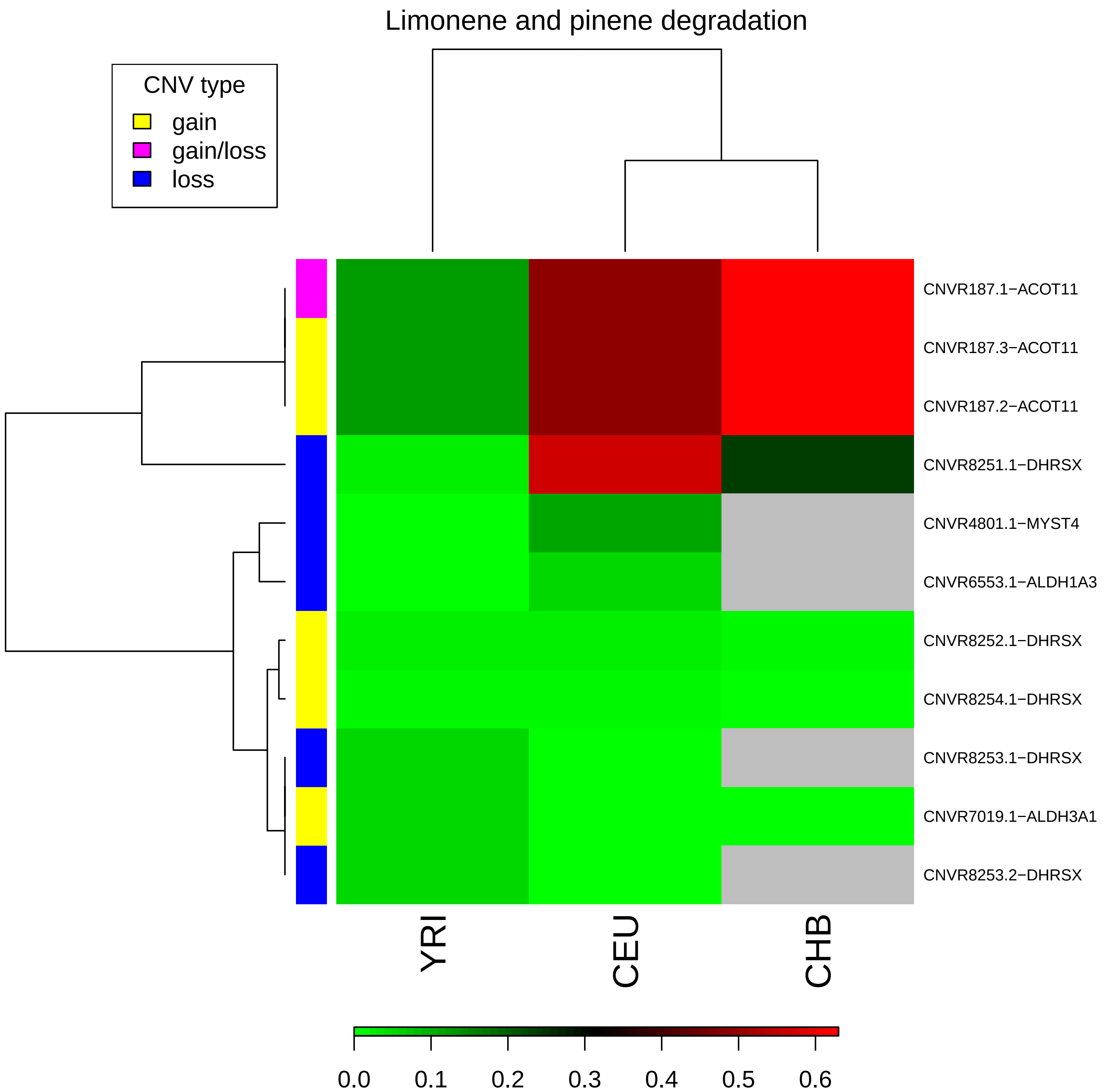

Limonene and pinene degradation
CNV type
gain
gain/loss
loss
CNVR187.1−ACOT11
CNVR187.3−ACOT11
CNVR187.2−ACOT11
CNVR8251.1−DHRSX
CNVR4801.1−MYST4
CNVR6553.1−ALDH1A3
CNVR8252.1−DHRSX
CNVR8254.1−DHRSX
CNVR8253.1−DHRSX
CNVR7019.1−ALDH3A1
CNVR8253.2−DHRSX
YRI
CEU
CHB
0.0
0.1
0.2
0.3
0.4
0.5
0.6

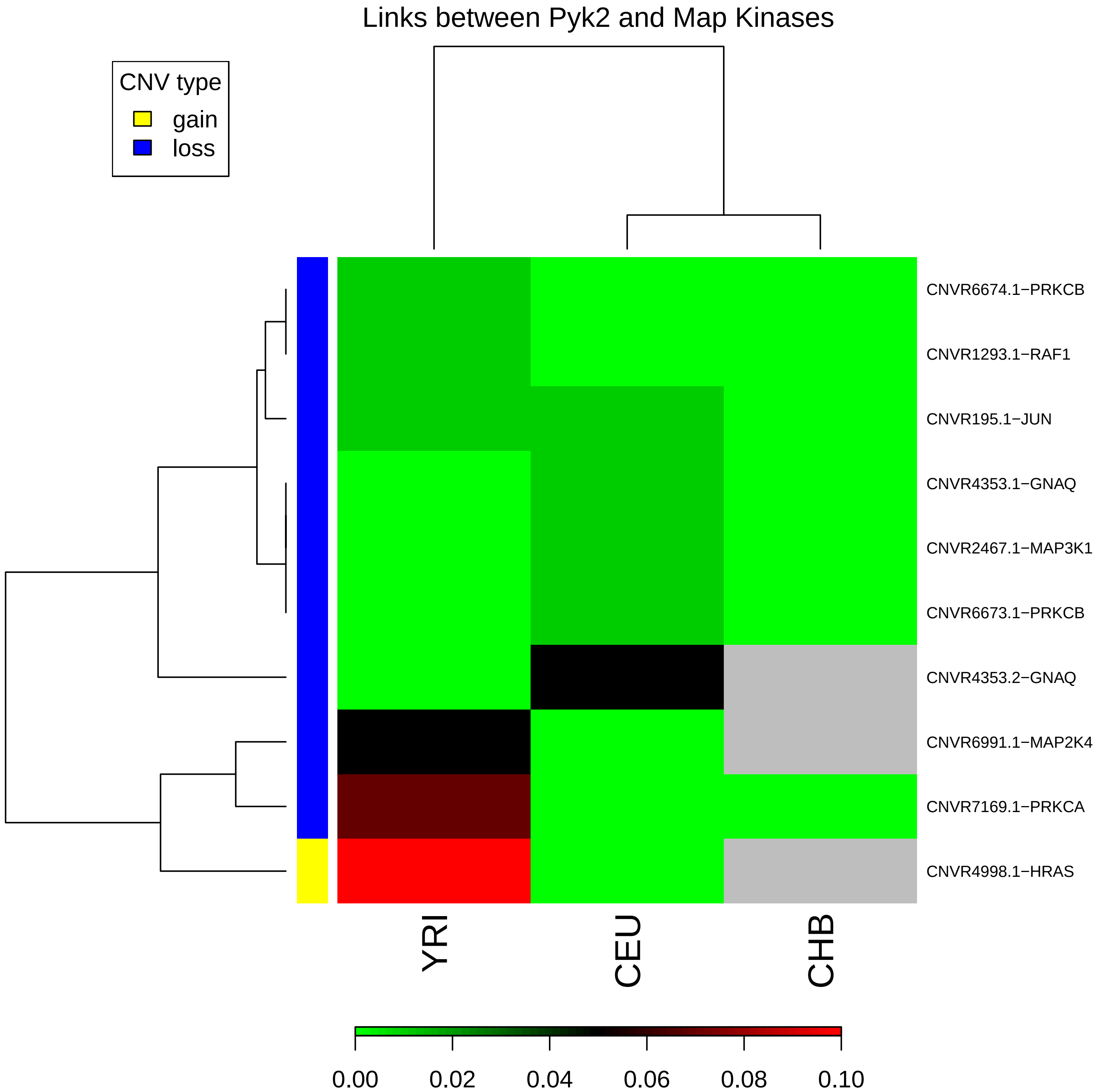
Links between Pyk2 and Map Kinases
CNV type
gain
loss
CNVR6674.1−PRKCB
CNVR1293.1−RAF1
CNVR195.1−JUN
CNVR4353.1−GNAQ
CNVR2467.1−MAP3K1
CNVR6673.1−PRKCB
CNVR4353.2−GNAQ
CNVR6991.1−MAP2K4
CNVR7169.1−PRKCA
CNVR4998.1−HRAS
YRI
CEU
CHB
0.00
0.02
0.04
0.06
0.08
0.10

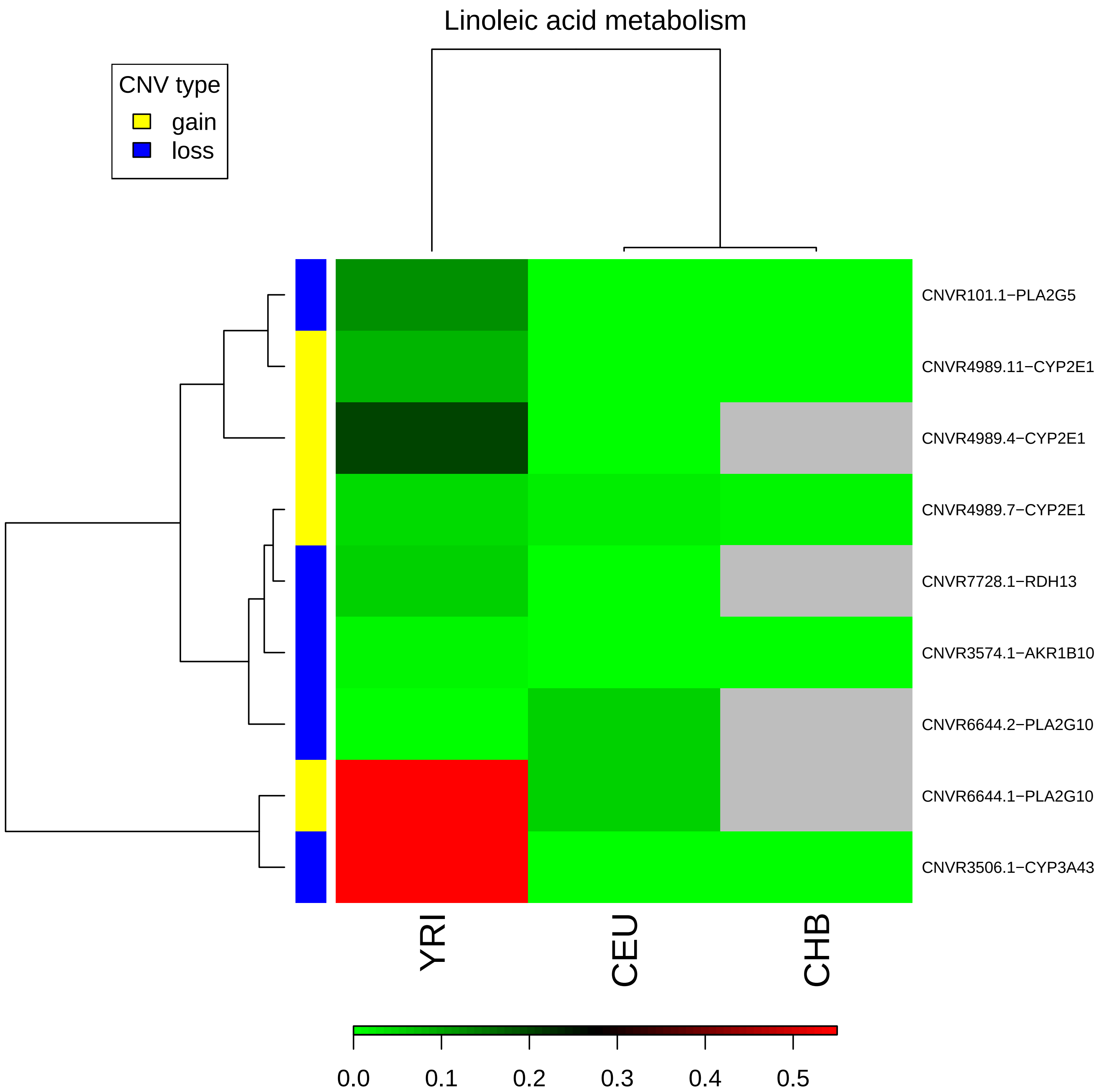
Linoleic acid metabolism
CNV type
gain
loss
CNVR101.1−PLA2G5
CNVR4989.11−CYP2E1
CNVR4989.4−CYP2E1
CNVR4989.7−CYP2E1
CNVR7728.1−RDH13
CNVR3574.1−AKR1B10
CNVR6644.2−PLA2G10
CNVR6644.1−PLA2G10
CNVR3506.1−CYP3A43
YRI
CEU
CHB
0.0
0.1
0.2
0.3
0.4
0.5

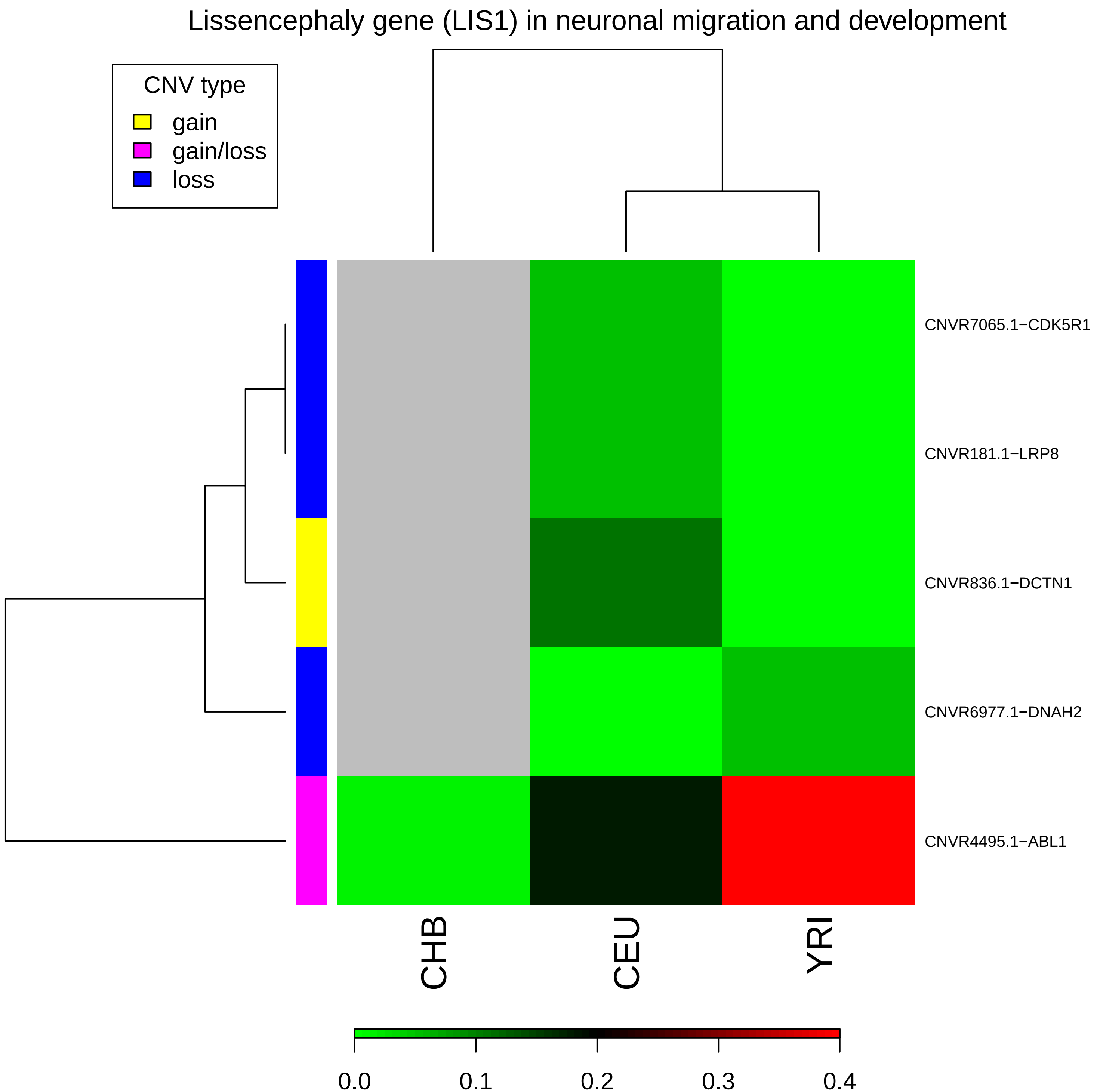

Lissencephaly gene (LIS1) in neuronal migration and development
CNV type
gain
gain/loss
loss
CNVR7065.1−CDK5R1
CNVR181.1−LRP8
CNVR836.1−DCTN1
CNVR6977.1−DNAH2
CNVR4495.1−ABL1
CHB
CEU
YRI
0.0
0.1
0.2
0.3
0.4

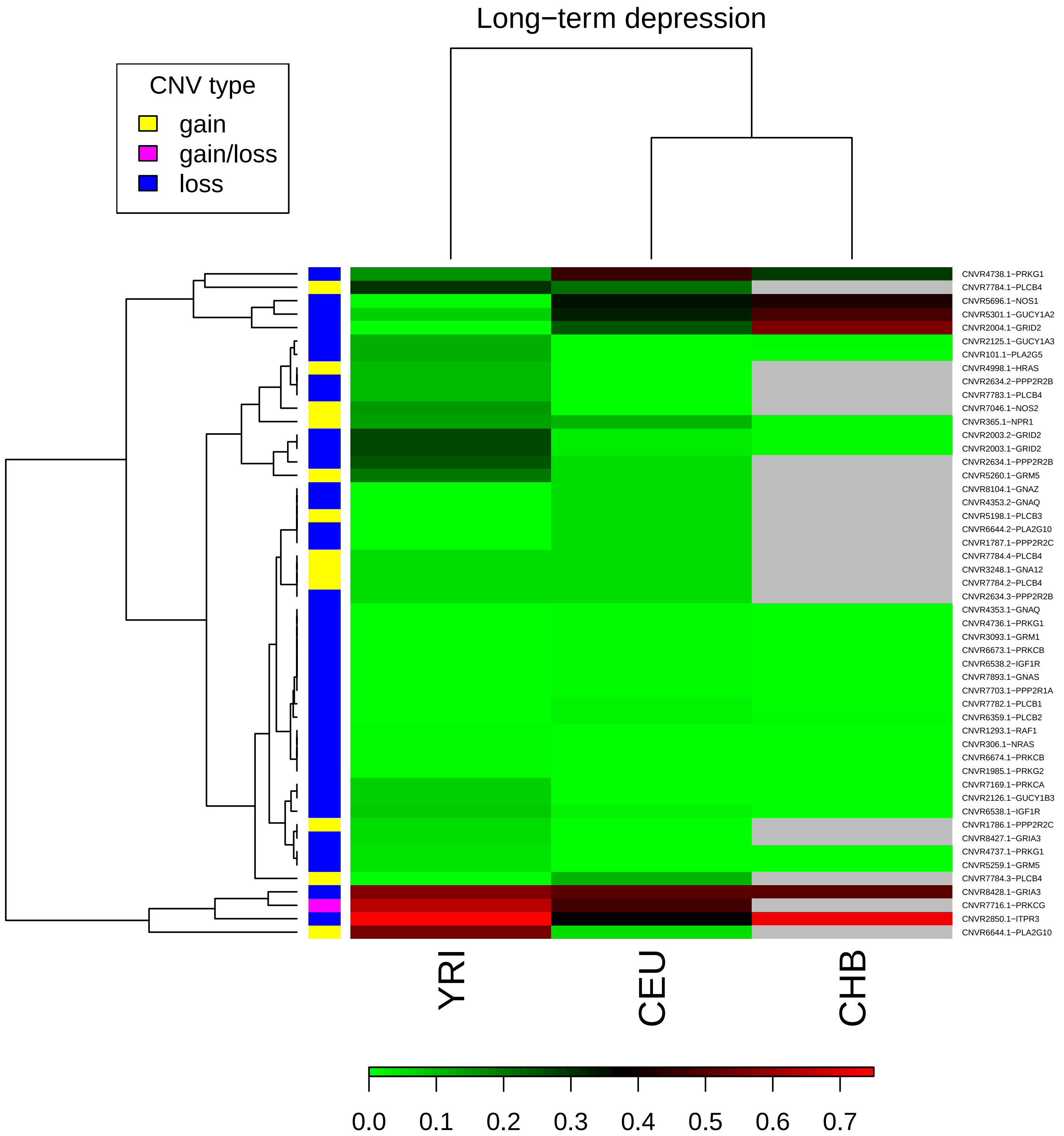

Long−term depression
CNV type
gain
gain/loss
loss
CNVR4738.1−PRKG1
CNVR7784.1−PLCB4
CNVR5696.1−NOS1
CNVR5301.1−GUCY1A2
CNVR2004.1−GRID2
CNVR2125.1−GUCY1A3
CNVR101.1−PLA2G5
CNVR4998.1−HRAS
CNVR2634.2−PPP2R2B
CNVR7783.1−PLCB4
CNVR7046.1−NOS2
CNVR365.1−NPR1
CNVR2003.2−GRID2
CNVR2003.1−GRID2
CNVR2634.1−PPP2R2B
CNVR5260.1−GRM5
CNVR8104.1−GNAZ
CNVR4353.2−GNAQ
CNVR5198.1−PLCB3
CNVR6644.2−PLA2G10
CNVR1787.1−PPP2R2C
CNVR7784.4−PLCB4
CNVR3248.1−GNA12
CNVR7784.2−PLCB4
CNVR2634.3−PPP2R2B
CNVR4353.1−GNAQ
CNVR4736.1−PRKG1
CNVR3093.1−GRM1
CNVR6673.1−PRKCB
CNVR6538.2−IGF1R
CNVR7893.1−GNAS
CNVR7703.1−PPP2R1A
CNVR7782.1−PLCB1
CNVR6359.1−PLCB2
CNVR1293.1−RAF1
CNVR306.1−NRAS
CNVR6674.1−PRKCB
CNVR1985.1−PRKG2
CNVR7169.1−PRKCA
CNVR2126.1−GUCY1B3
CNVR6538.1−IGF1R
CNVR1786.1−PPP2R2C
CNVR8427.1−GRIA3
CNVR4737.1−PRKG1
CNVR5259.1−GRM5
CNVR7784.3−PLCB4
CNVR8428.1−GRIA3
CNVR7716.1−PRKCG
CNVR2850.1−ITPR3
CNVR6644.1−PLA2G10
YRI
CEU
CHB
0.0
0.1
0.2
0.3
0.4
0.5
0.6
0.7

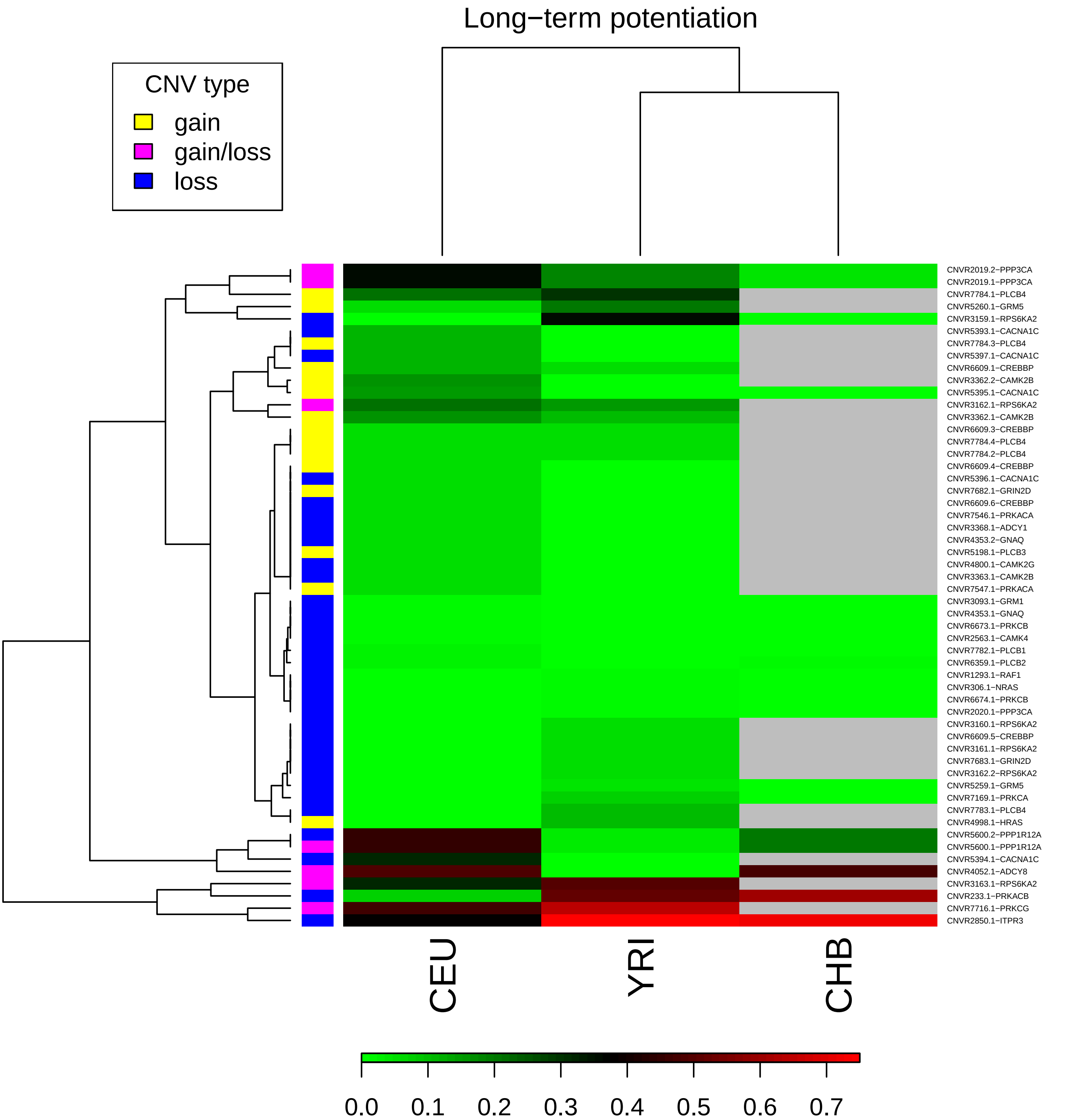

Long−term potentiation
CNV type
gain
gain/loss
loss
CNVR2019.2−PPP3CA
CNVR2019.1−PPP3CA
CNVR7784.1−PLCB4
CNVR5260.1−GRM5
CNVR3159.1−RPS6KA2
CNVR5393.1−CACNA1C
CNVR7784.3−PLCB4
CNVR5397.1−CACNA1C
CNVR6609.1−CREBBP
CNVR3362.2−CAMK2B
CNVR5395.1−CACNA1C
CNVR3162.1−RPS6KA2
CNVR3362.1−CAMK2B
CNVR6609.3−CREBBP
CNVR7784.4−PLCB4
CNVR7784.2−PLCB4
CNVR6609.4−CREBBP
CNVR5396.1−CACNA1C
CNVR7682.1−GRIN2D
CNVR6609.6−CREBBP
CNVR7546.1−PRKACA
CNVR3368.1−ADCY1
CNVR4353.2−GNAQ
CNVR5198.1−PLCB3
CNVR4800.1−CAMK2G
CNVR3363.1−CAMK2B
CNVR7547.1−PRKACA
CNVR3093.1−GRM1
CNVR4353.1−GNAQ
CNVR6673.1−PRKCB
CNVR2563.1−CAMK4
CNVR7782.1−PLCB1
CNVR6359.1−PLCB2
CNVR1293.1−RAF1
CNVR306.1−NRAS
CNVR6674.1−PRKCB
CNVR2020.1−PPP3CA
CNVR3160.1−RPS6KA2
CNVR6609.5−CREBBP
CNVR3161.1−RPS6KA2
CNVR7683.1−GRIN2D
CNVR3162.2−RPS6KA2
CNVR5259.1−GRM5
CNVR7169.1−PRKCA
CNVR7783.1−PLCB4
CNVR4998.1−HRAS
CNVR5600.2−PPP1R12A
CNVR5600.1−PPP1R12A
CNVR5394.1−CACNA1C
CNVR4052.1−ADCY8
CNVR3163.1−RPS6KA2
CNVR233.1−PRKACB
CNVR7716.1−PRKCG
CNVR2850.1−ITPR3
CEU
YRI
CHB
0.0
0.1
0.2
0.3
0.4
0.5
0.6
0.7

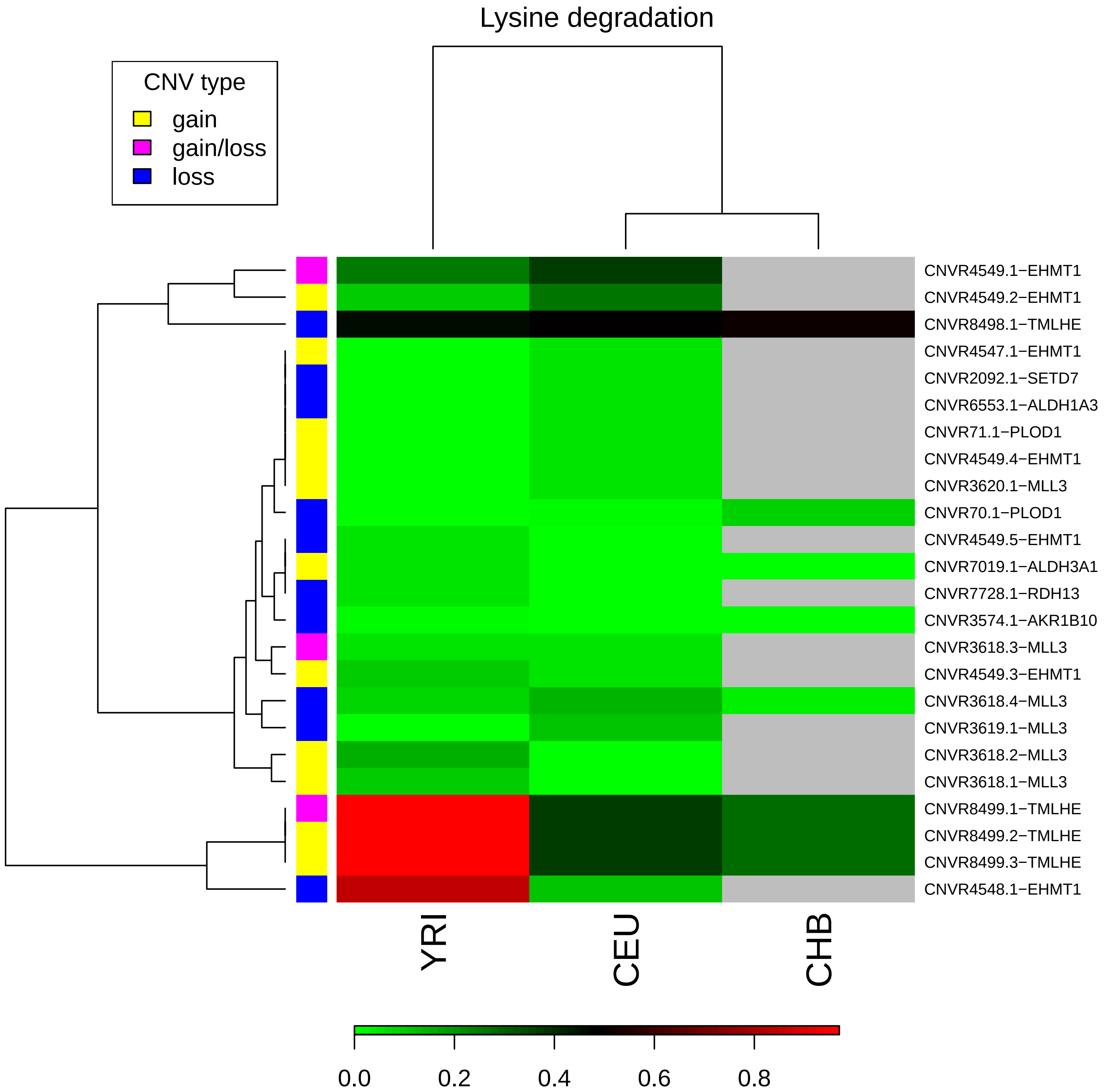

Lysine degradation
CNV type
gain
gain/loss
loss
CNVR4549.1−EHMT1
CNVR4549.2−EHMT1
CNVR8498.1−TMLHE
CNVR4547.1−EHMT1
CNVR2092.1−SETD7
CNVR6553.1−ALDH1A3
CNVR71.1−PLOD1
CNVR4549.4−EHMT1
CNVR3620.1−MLL3
CNVR70.1−PLOD1
CNVR4549.5−EHMT1
CNVR7019.1−ALDH3A1
CNVR7728.1−RDH13
CNVR3574.1−AKR1B10
CNVR3618.3−MLL3
CNVR4549.3−EHMT1
CNVR3618.4−MLL3
CNVR3619.1−MLL3
CNVR3618.2−MLL3
CNVR3618.1−MLL3
CNVR8499.1−TMLHE
CNVR8499.2−TMLHE
CNVR8499.3−TMLHE
CNVR4548.1−EHMT1
YRI
CEU
CHB
0.0
0.2
0.4
0.6
0.8

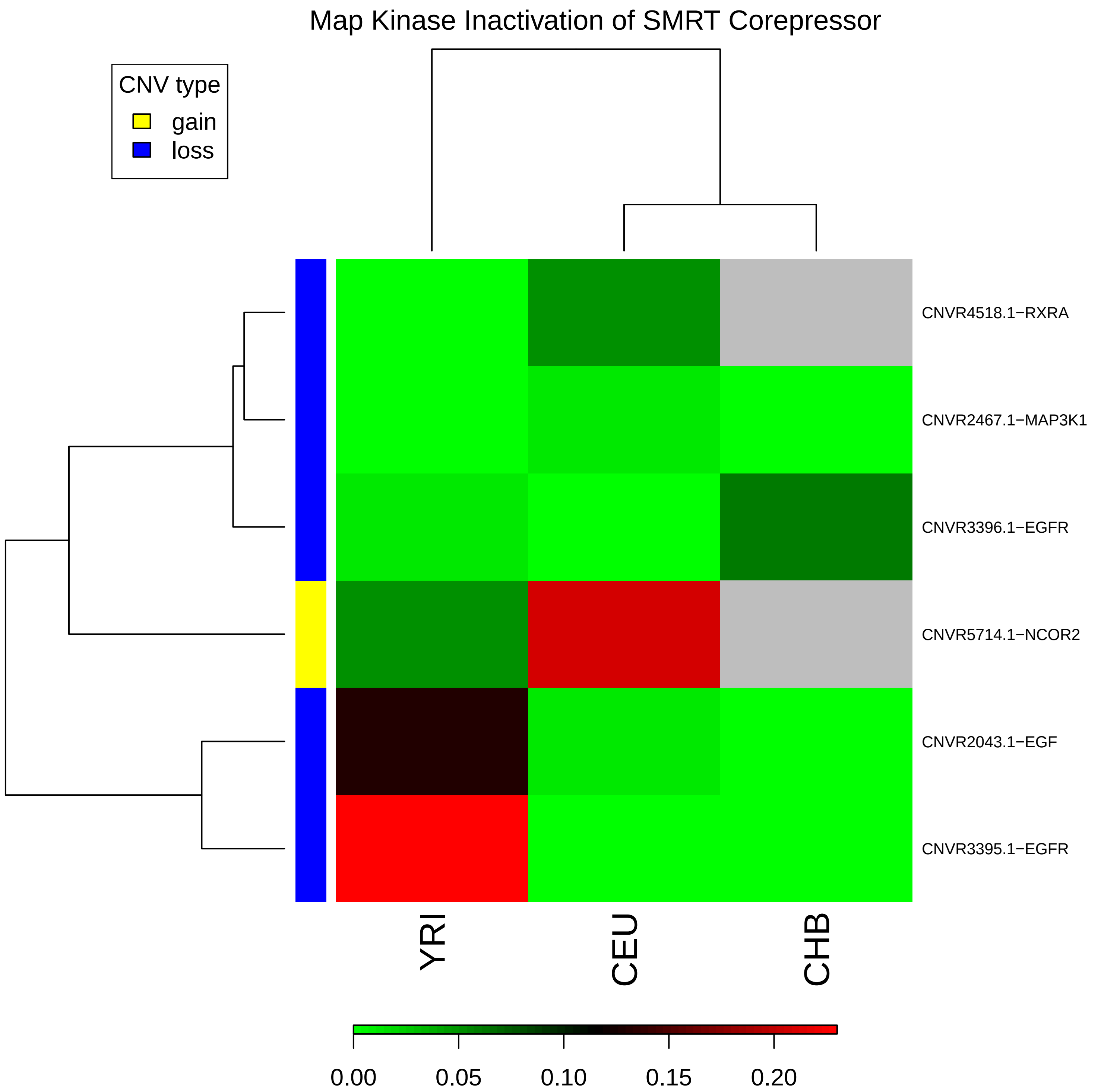

Map Kinase Inactivation of SMRT Corepressor
CNV type
gain
loss
CNVR4518.1−RXRA
CNVR2467.1−MAP3K1
CNVR3396.1−EGFR
CNVR5714.1−NCOR2
CNVR2043.1−EGF
CNVR3395.1−EGFR
YRI
CEU
CHB
0.00
0.05
0.10
0.15
0.20

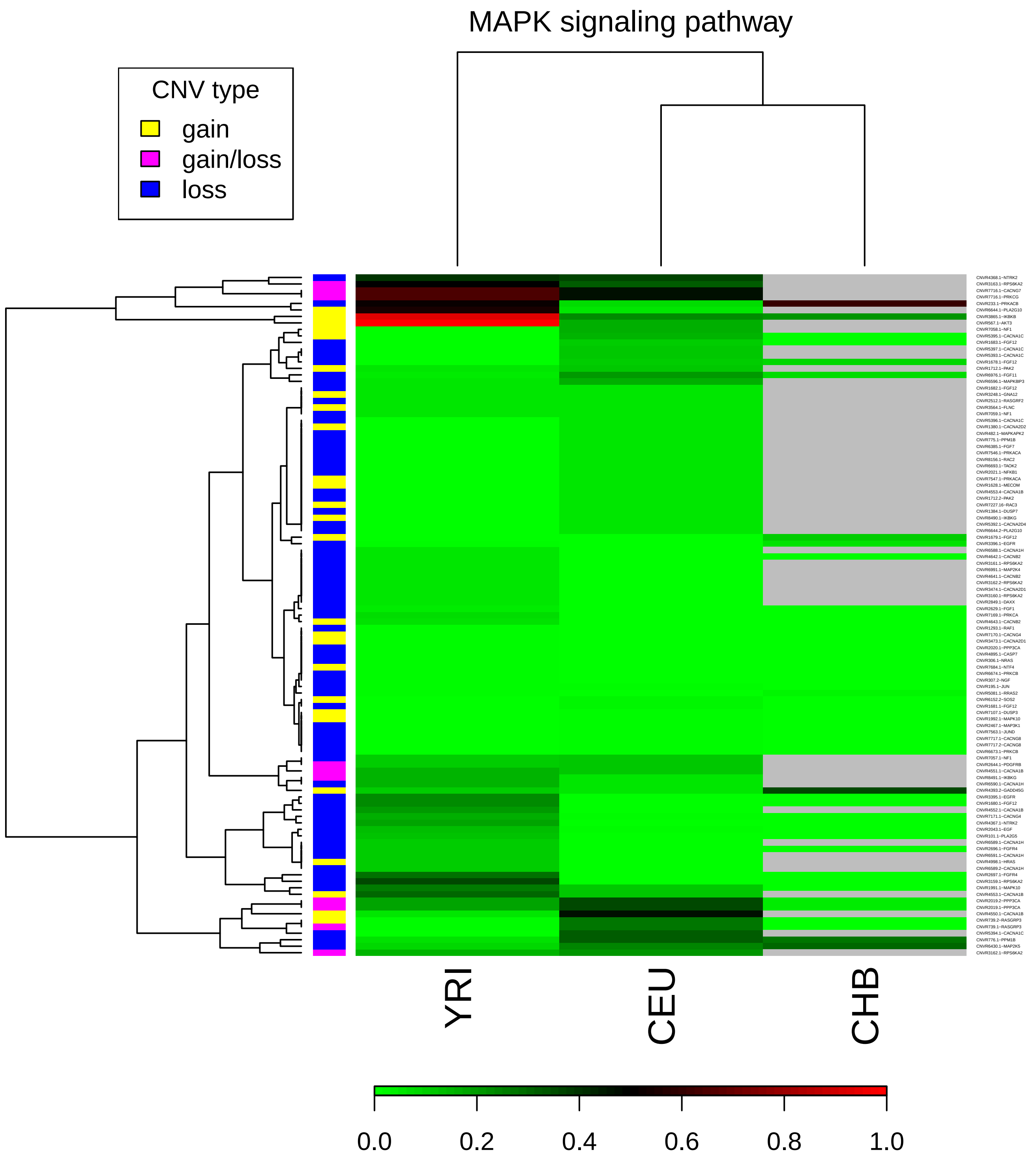
MAPK signaling pathway
CNV type
gain
gain/loss
loss
CNVR4368.1−NTRK2
CNVR3163.1−RPS6KA2
CNVR7716.1−CACNG7
CNVR7716.1−PRKCG
CNVR233.1−PRKACB
CNVR6644.1−PLA2G10
CNVR3865.1−IKBKB
CNVR567.1−AKT3
CNVR7058.1−NF1
CNVR5395.1−CACNA1C
CNVR1683.1−FGF12
CNVR5397.1−CACNA1C
CNVR5393.1−CACNA1C
CNVR1678.1−FGF12
CNVR1712.1−PAK2
CNVR6976.1−FGF11
CNVR6596.1−MAPK8IP3
CNVR1682.1−FGF12
CNVR3248.1−GNA12
CNVR2512.1−RASGRF2
CNVR3564.1−FLNC
CNVR7059.1−NF1
CNVR5396.1−CACNA1C
CNVR1380.1−CACNA2D2
CNVR482.1−MAPKAPK2
CNVR775.1−PPM1B
CNVR6385.1−FGF7
CNVR7546.1−PRKACA
CNVR8156.1−RAC2
CNVR6693.1−TAOK2
CNVR2021.1−NFKB1
CNVR7547.1−PRKACA
CNVR1628.1−MECOM
CNVR4553.4−CACNA1B
CNVR1712.2−PAK2
CNVR7227.16−RAC3
CNVR1384.1−DUSP7
CNVR8490.1−IKBKG
CNVR5392.1−CACNA2D4
CNVR6644.2−PLA2G10
CNVR1679.1−FGF12
CNVR3396.1−EGFR
CNVR6588.1−CACNA1H
CNVR4642.1−CACNB2
CNVR3161.1−RPS6KA2
CNVR6991.1−MAP2K4
CNVR4641.1−CACNB2
CNVR3162.2−RPS6KA2
CNVR3474.1−CACNA2D1
CNVR3160.1−RPS6KA2
CNVR2849.1−DAXX
CNVR2629.1−FGF1
CNVR7169.1−PRKCA
CNVR4643.1−CACNB2
CNVR1293.1−RAF1
CNVR7170.1−CACNG4
CNVR3473.1−CACNA2D1
CNVR2020.1−PPP3CA
CNVR4895.1−CASP7
CNVR306.1−NRAS
CNVR7684.1−NTF4
CNVR6674.1−PRKCB
CNVR307.2−NGF
CNVR195.1−JUN
CNVR5081.1−RRAS2
CNVR6152.2−SOS2
CNVR1681.1−FGF12
CNVR7107.1−DUSP3
CNVR1992.1−MAPK10
CNVR2467.1−MAP3K1
CNVR7563.1−JUND
CNVR7717.1−CACNG8
CNVR7717.2−CACNG8
CNVR6673.1−PRKCB
CNVR7057.1−NF1
CNVR2644.1−PDGFRB
CNVR4551.1−CACNA1B
CNVR8491.1−IKBKG
CNVR6590.1−CACNA1H
CNVR4393.2−GADD45G
CNVR3395.1−EGFR
CNVR1680.1−FGF12
CNVR4552.1−CACNA1B
CNVR7171.1−CACNG4
CNVR4367.1−NTRK2
CNVR2043.1−EGF
CNVR101.1−PLA2G5
CNVR6589.1−CACNA1H
CNVR2696.1−FGFR4
CNVR6591.1−CACNA1H
CNVR4998.1−HRAS
CNVR6589.2−CACNA1H
CNVR2697.1−FGFR4
CNVR3159.1−RPS6KA2
CNVR1991.1−MAPK10
CNVR4553.1−CACNA1B
CNVR2019.2−PPP3CA
CNVR2019.1−PPP3CA
CNVR4550.1−CACNA1B
CNVR739.2−RASGRP3
CNVR739.1−RASGRP3
CNVR5394.1−CACNA1C
CNVR776.1−PPM1B
CNVR6430.1−MAP2K5
CNVR3162.1−RPS6KA2
YRI
CEU
CHB
0.0
0.2
0.4
0.6
0.8
1.0

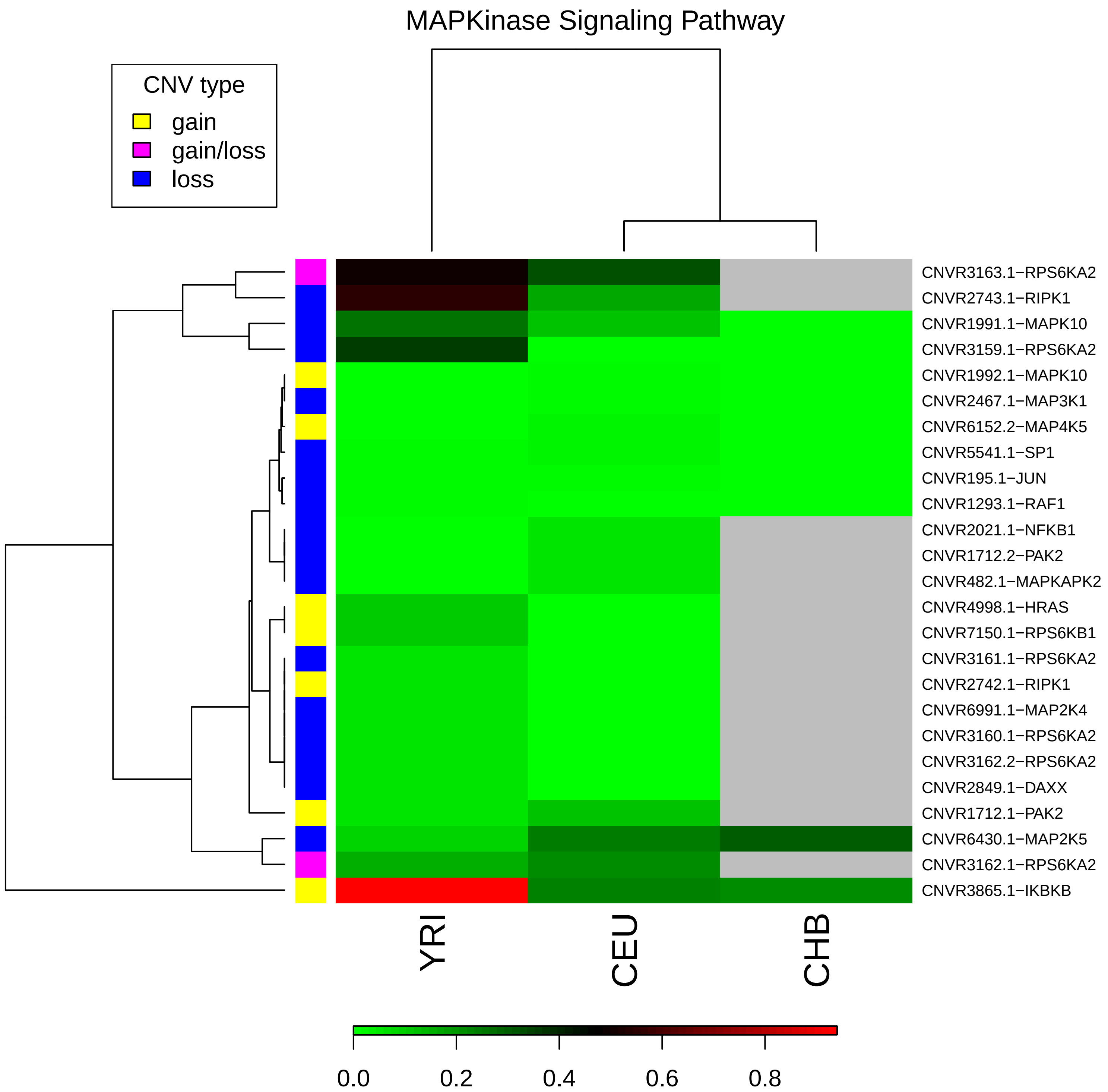
MAPKinase Signaling Pathway
CNV type
gain
gain/loss
loss
CNVR3163.1−RPS6KA2
CNVR2743.1−RIPK1
CNVR1991.1−MAPK10
CNVR3159.1−RPS6KA2
CNVR1992.1−MAPK10
CNVR2467.1−MAP3K1
CNVR6152.2−MAP4K5
CNVR5541.1−SP1
CNVR195.1−JUN
CNVR1293.1−RAF1
CNVR2021.1−NFKB1
CNVR1712.2−PAK2
CNVR482.1−MAPKAPK2
CNVR4998.1−HRAS
CNVR7150.1−RPS6KB1
CNVR3161.1−RPS6KA2
CNVR2742.1−RIPK1
CNVR6991.1−MAP2K4
CNVR3160.1−RPS6KA2
CNVR3162.2−RPS6KA2
CNVR2849.1−DAXX
CNVR1712.1−PAK2
CNVR6430.1−MAP2K5
CNVR3162.1−RPS6KA2
CNVR3865.1−IKBKB
YRI
CEU
CHB
0.0
0.2
0.4
0.6
0.8

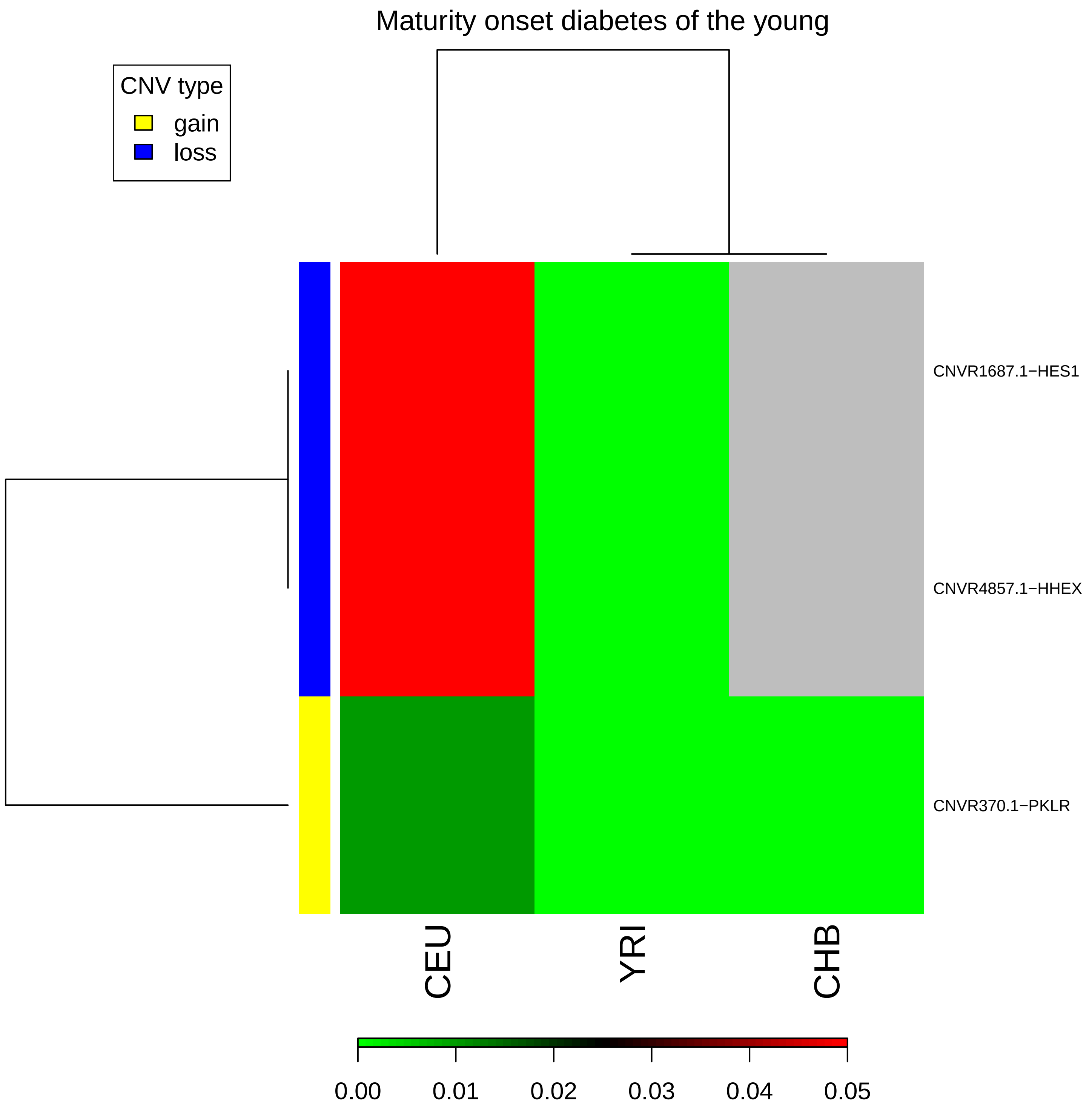
Maturity onset diabetes of the young
CNV type
gain
loss
CNVR1687.1−HES1
CNVR4857.1−HHEX
CNVR370.1−PKLR
CEU
YRI
CHB
0.00
0.01
0.02
0.03
0.04
0.05

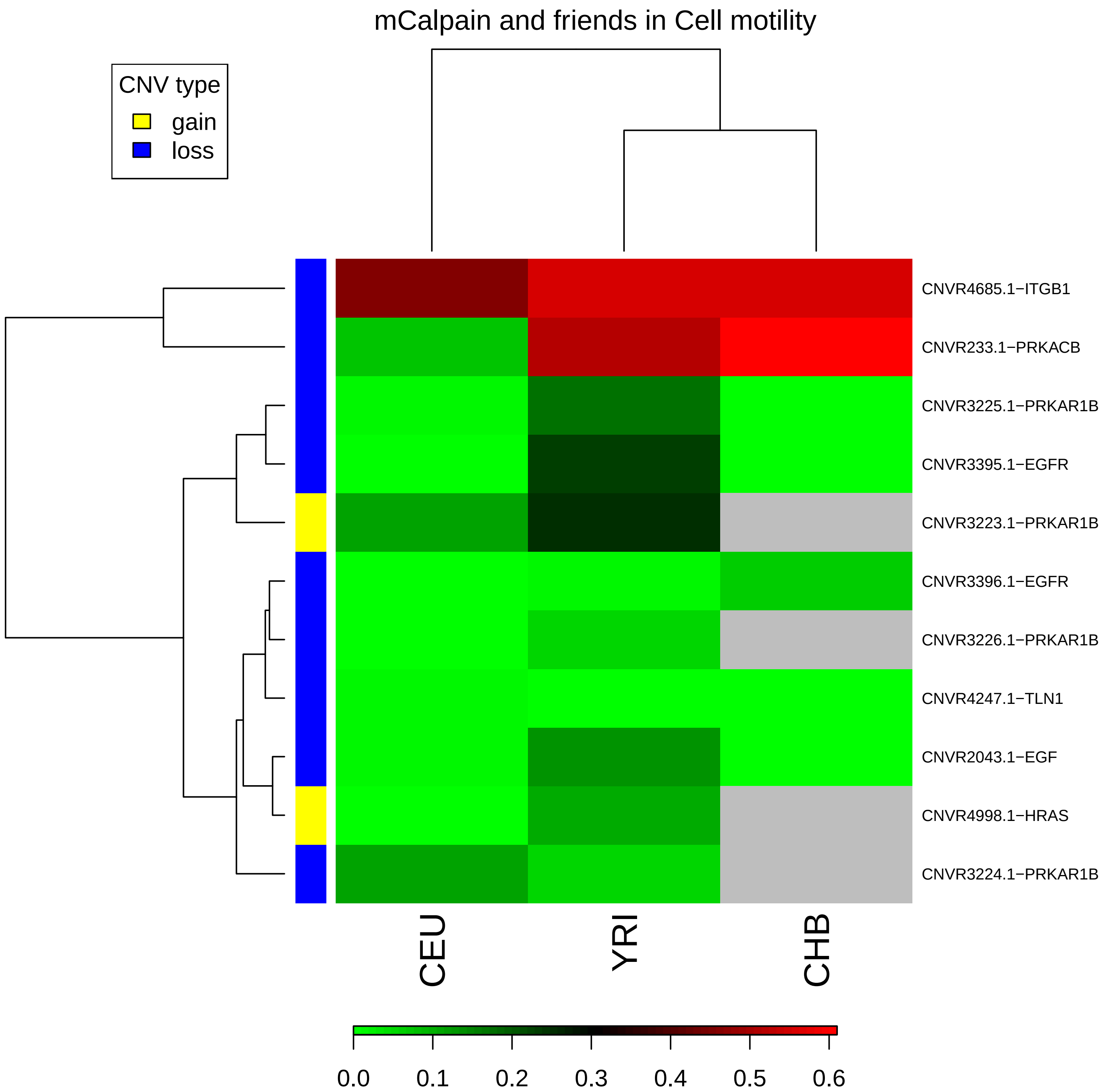
mCalpain and friends in Cell motility
CNV type
gain
loss
CNVR4685.1−ITGB1
CNVR233.1−PRKACB
CNVR3225.1−PRKAR1B
CNVR3395.1−EGFR
CNVR3223.1−PRKAR1B
CNVR3396.1−EGFR
CNVR3226.1−PRKAR1B
CNVR4247.1−TLN1
CNVR2043.1−EGF
CNVR4998.1−HRAS
CNVR3224.1−PRKAR1B
CEU
YRI
CHB
0.0
0.1
0.2
0.3
0.4
0.5
0.6

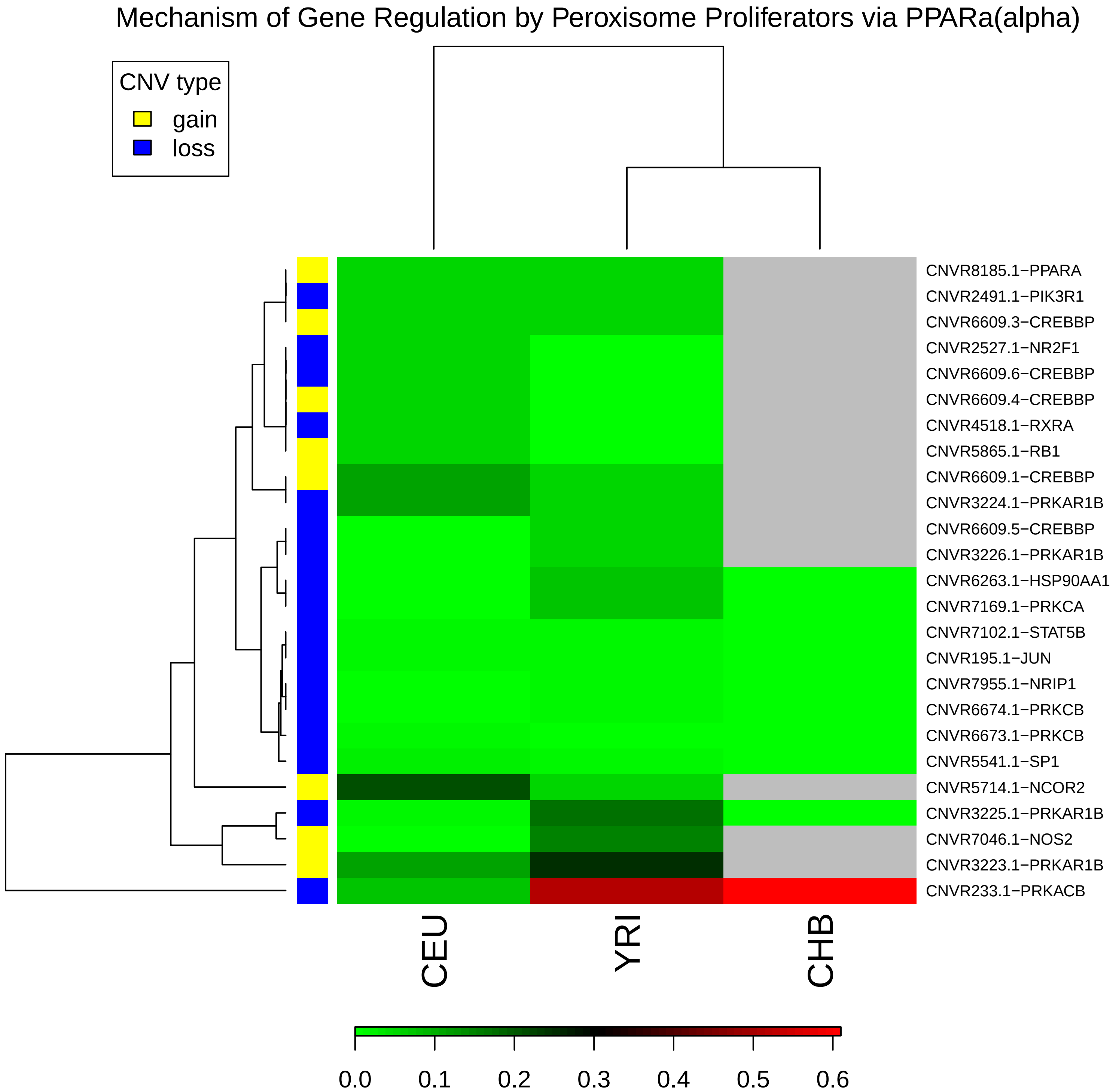

Mechanism of Gene Regulation by Peroxisome Proliferators via PPARa(alpha)
CNV type
gain
loss
CNVR8185.1−PPARA
CNVR2491.1−PIK3R1
CNVR6609.3−CREBBP
CNVR2527.1−NR2F1
CNVR6609.6−CREBBP
CNVR6609.4−CREBBP
CNVR4518.1−RXRA
CNVR5865.1−RB1
CNVR6609.1−CREBBP
CNVR3224.1−PRKAR1B
CNVR6609.5−CREBBP
CNVR3226.1−PRKAR1B
CNVR6263.1−HSP90AA1
CNVR7169.1−PRKCA
CNVR7102.1−STAT5B
CNVR195.1−JUN
CNVR7955.1−NRIP1
CNVR6674.1−PRKCB
CNVR6673.1−PRKCB
CNVR5541.1−SP1
CNVR5714.1−NCOR2
CNVR3225.1−PRKAR1B
CNVR7046.1−NOS2
CNVR3223.1−PRKAR1B
CNVR233.1−PRKACB
CEU
YRI
CHB
0.0
0.1
0.2
0.3
0.4
0.5
0.6

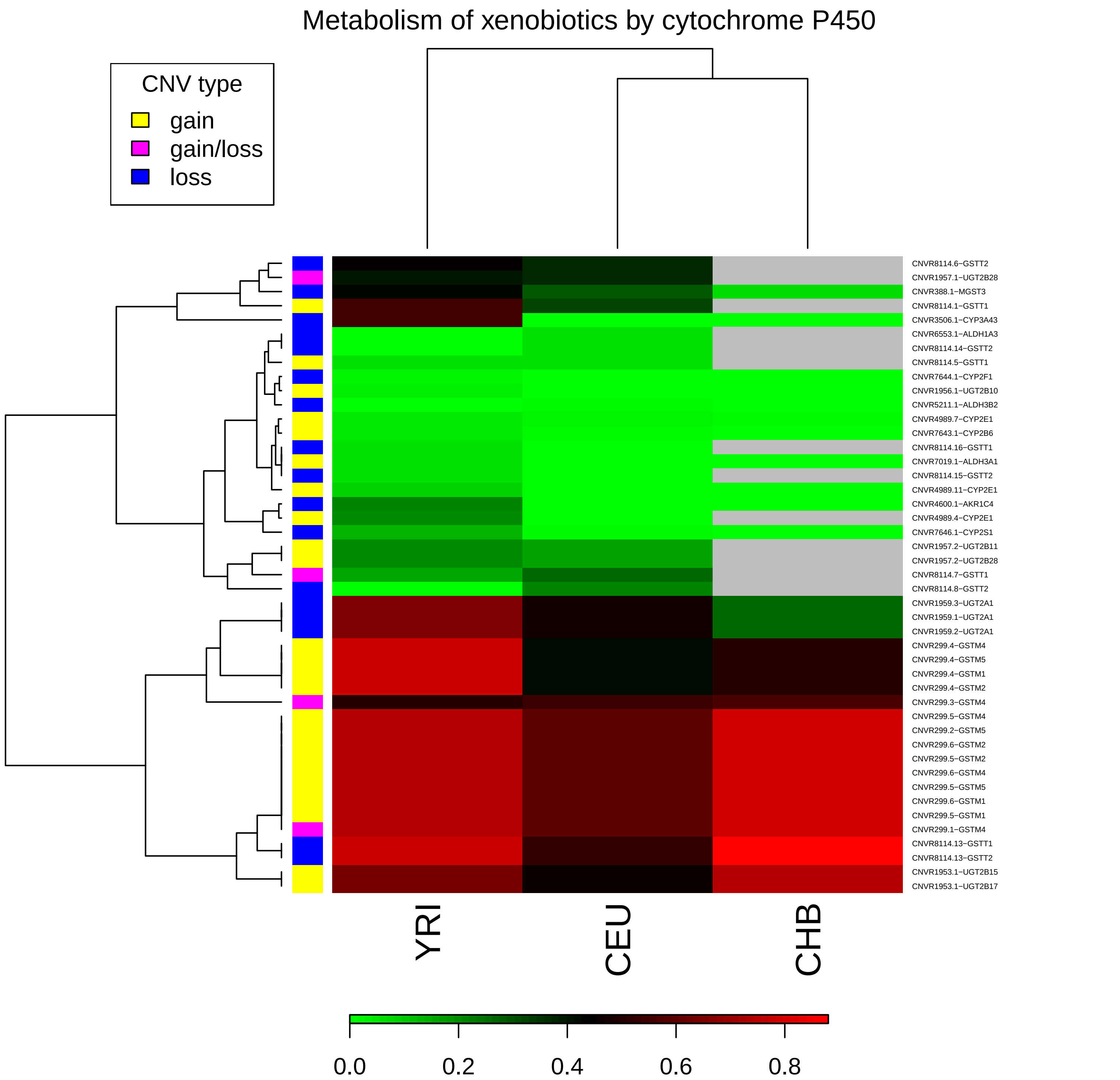
Metabolism of xenobiotics by cytochrome P450
CNV type
gain
gain/loss
loss
CNVR8114.6−GSTT2
CNVR1957.1−UGT2B28
CNVR388.1−MGST3
CNVR8114.1−GSTT1
CNVR3506.1−CYP3A43
CNVR6553.1−ALDH1A3
CNVR8114.14−GSTT2
CNVR8114.5−GSTT1
CNVR7644.1−CYP2F1
CNVR1956.1−UGT2B10
CNVR5211.1−ALDH3B2
CNVR4989.7−CYP2E1
CNVR7643.1−CYP2B6
CNVR8114.16−GSTT1
CNVR7019.1−ALDH3A1
CNVR8114.15−GSTT2
CNVR4989.11−CYP2E1
CNVR4600.1−AKR1C4
CNVR4989.4−CYP2E1
CNVR7646.1−CYP2S1
CNVR1957.2−UGT2B11
CNVR1957.2−UGT2B28
CNVR8114.7−GSTT1
CNVR8114.8−GSTT2
CNVR1959.3−UGT2A1
CNVR1959.1−UGT2A1
CNVR1959.2−UGT2A1
CNVR299.4−GSTM4
CNVR299.4−GSTM5
CNVR299.4−GSTM1
CNVR299.4−GSTM2
CNVR299.3−GSTM4
CNVR299.5−GSTM4
CNVR299.2−GSTM5
CNVR299.6−GSTM2
CNVR299.5−GSTM2
CNVR299.6−GSTM4
CNVR299.5−GSTM5
CNVR299.6−GSTM1
CNVR299.5−GSTM1
CNVR299.1−GSTM4
CNVR8114.13−GSTT1
CNVR8114.13−GSTT2
CNVR1953.1−UGT2B15
CNVR1953.1−UGT2B17
YRI
CEU
CHB
0.0
0.2
0.4
0.6
0.8

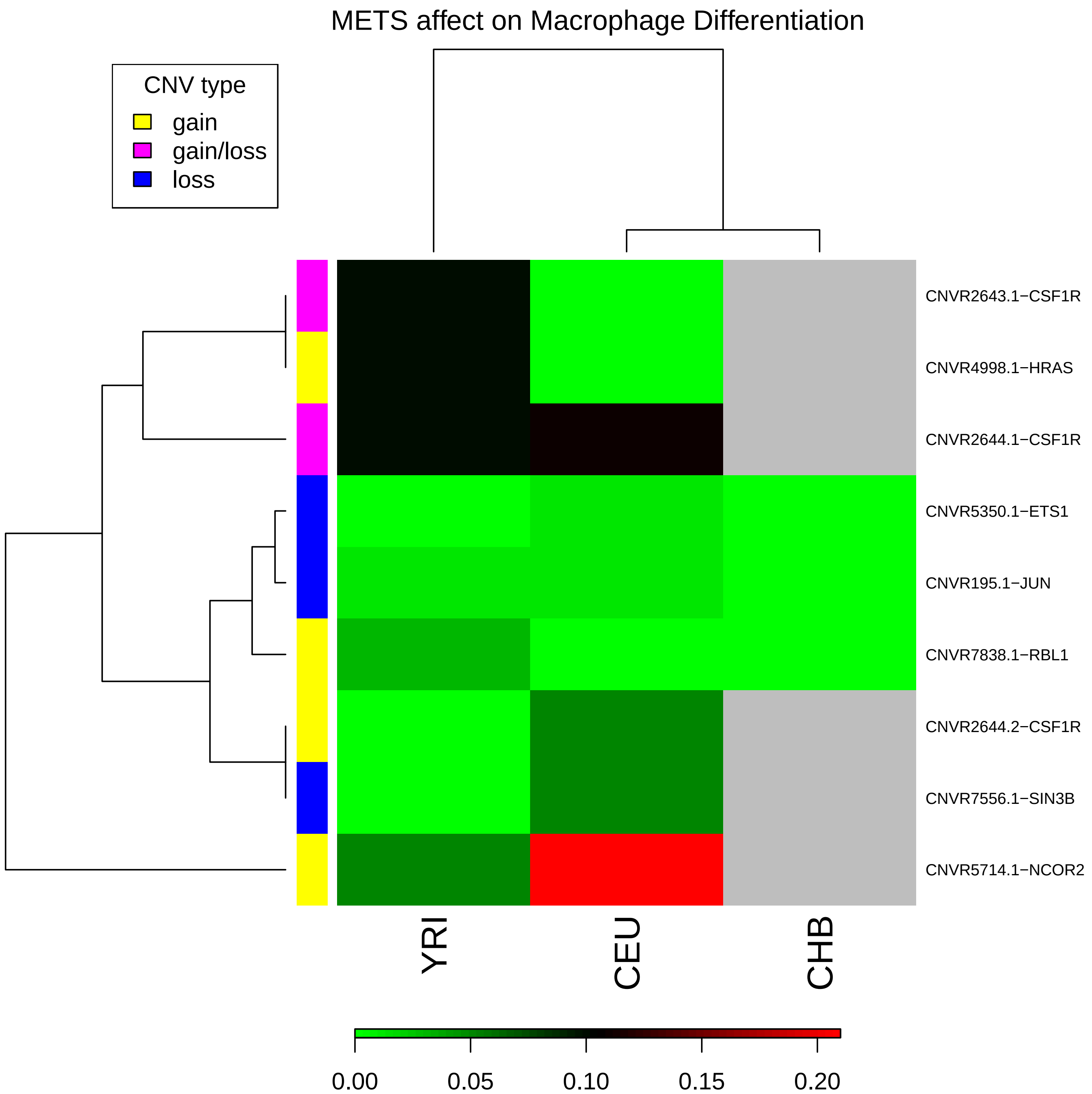

METS affect on Macrophage Differentiation
CNV type
gain
gain/loss
loss
CNVR2643.1−CSF1R
CNVR4998.1−HRAS
CNVR2644.1−CSF1R
CNVR5350.1−ETS1
CNVR195.1−JUN
CNVR7838.1−RBL1
CNVR2644.2−CSF1R
CNVR7556.1−SIN3B
CNVR5714.1−NCOR2
YRI
CEU
CHB
0.00
0.05
0.10
0.15
0.20

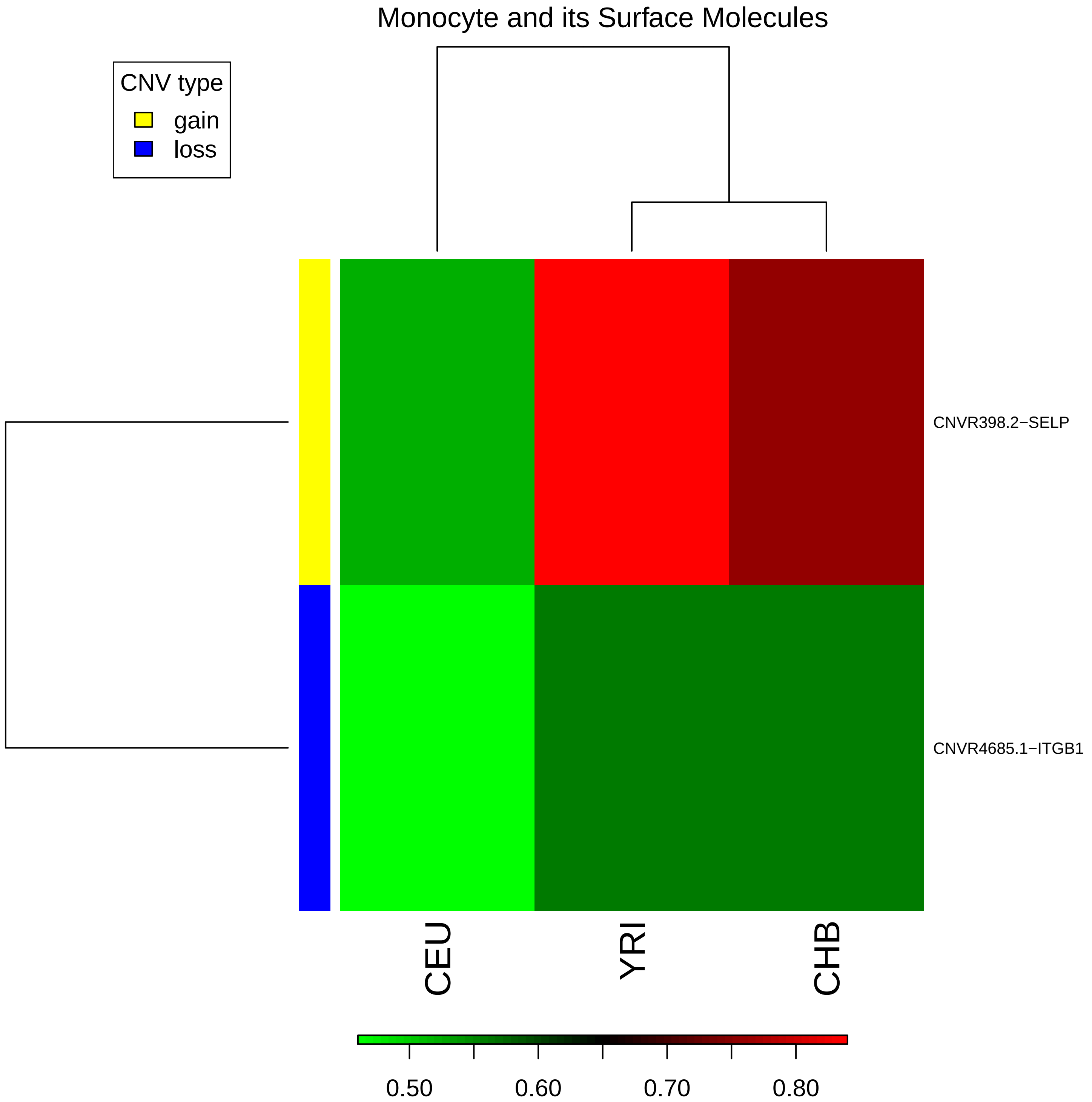
Monocyte and its Surface Molecules
CNV type
gain
loss
CNVR398.2−SELP
CNVR4685.1−ITGB1
CEU
YRI
CHB
0.50
0.60
0.70
0.80

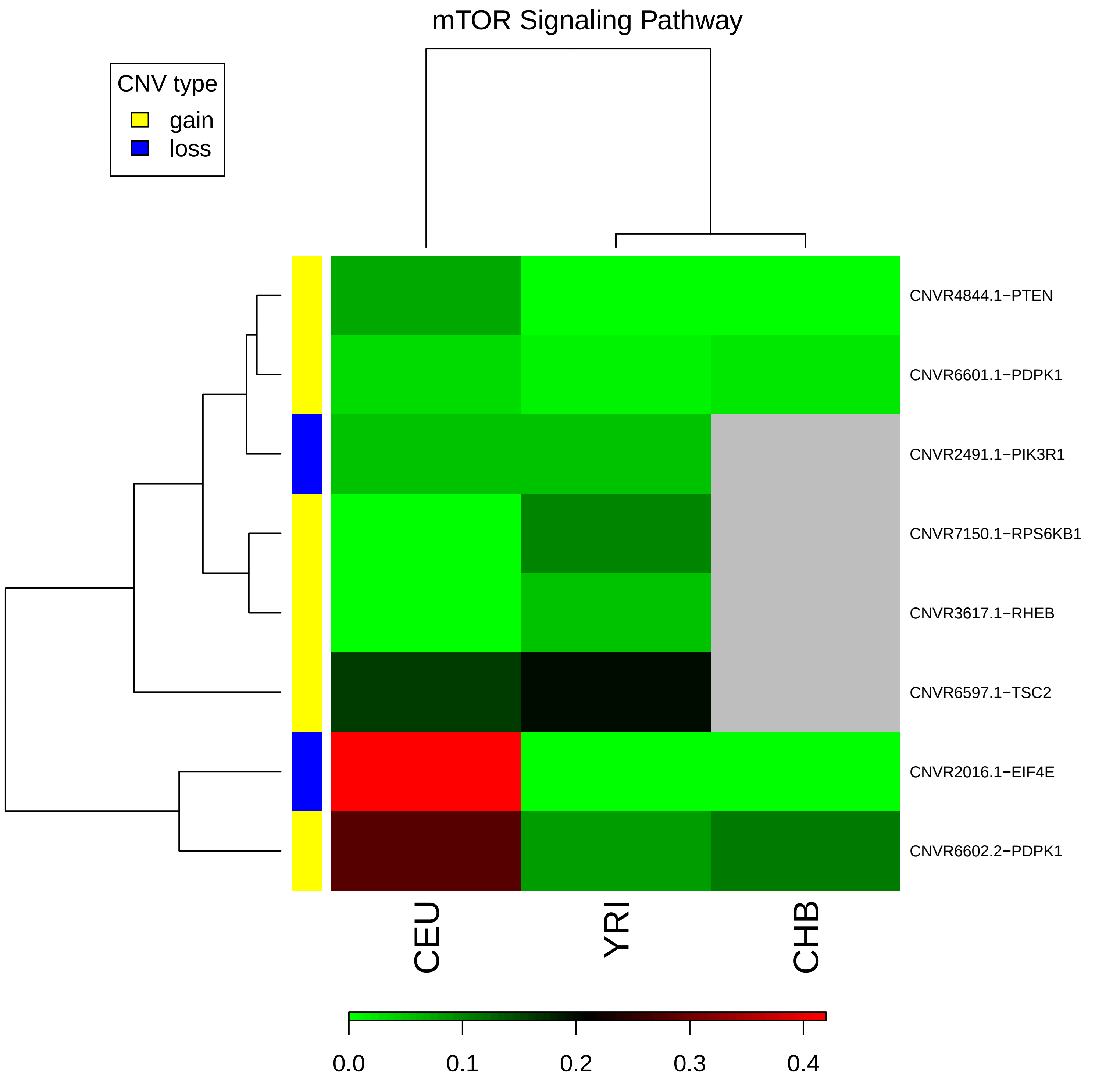
mTOR Signaling Pathway
CNV type
gain
loss
CNVR4844.1−PTEN
CNVR6601.1−PDPK1
CNVR2491.1−PIK3R1
CNVR7150.1−RPS6KB1
CNVR3617.1−RHEB
CNVR6597.1−TSC2
CNVR2016.1−EIF4E
CNVR6602.2−PDPK1
CEU
YRI
CHB
0.0
0.1
0.2
0.3
0.4

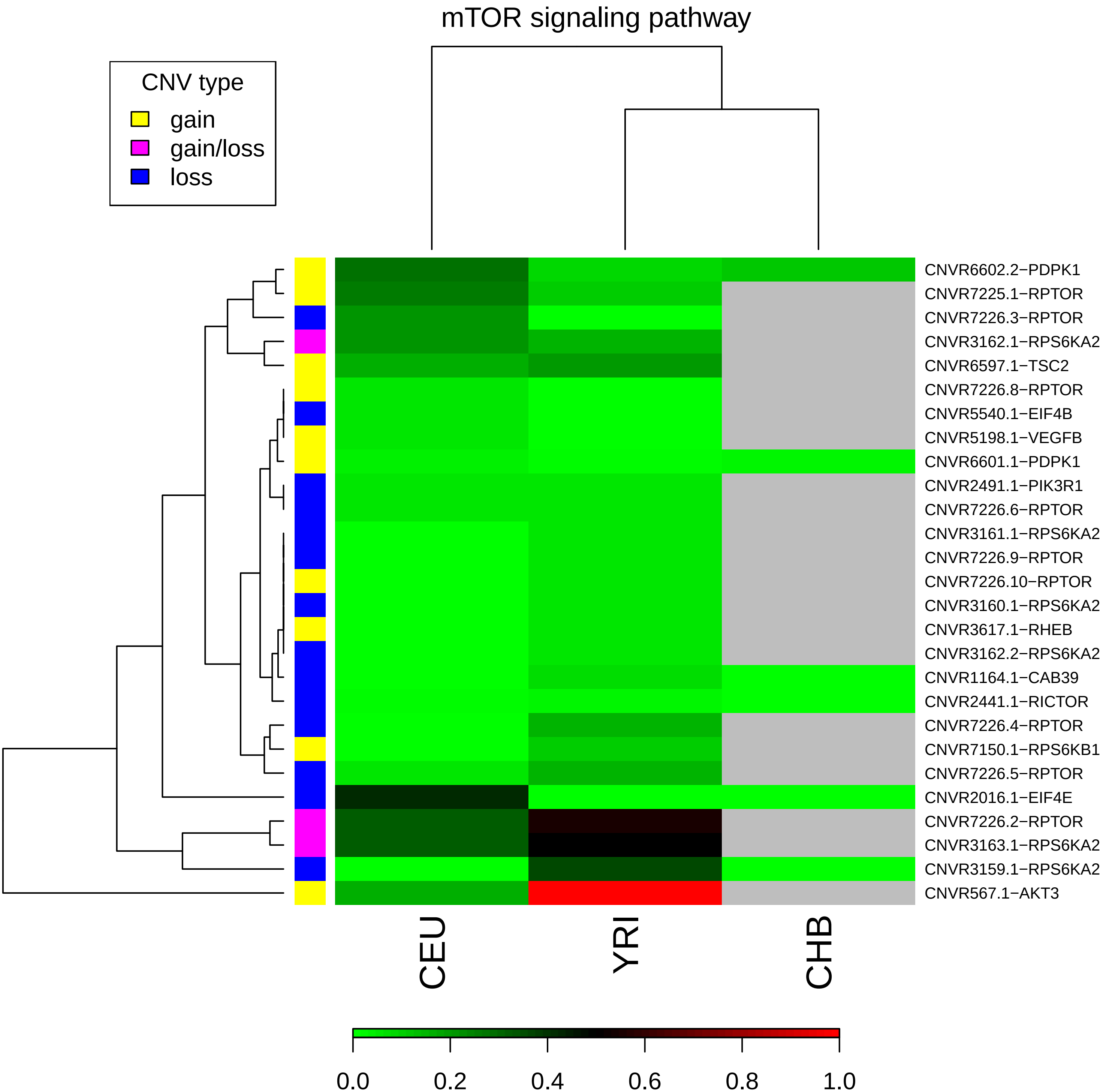
mTOR signaling pathway
CNV type
gain
gain/loss
loss
CNVR6602.2−PDPK1
CNVR7225.1−RPTOR
CNVR7226.3−RPTOR
CNVR3162.1−RPS6KA2
CNVR6597.1−TSC2
CNVR7226.8−RPTOR
CNVR5540.1−EIF4B
CNVR5198.1−VEGFB
CNVR6601.1−PDPK1
CNVR2491.1−PIK3R1
CNVR7226.6−RPTOR
CNVR3161.1−RPS6KA2
CNVR7226.9−RPTOR
CNVR7226.10−RPTOR
CNVR3160.1−RPS6KA2
CNVR3617.1−RHEB
CNVR3162.2−RPS6KA2
CNVR1164.1−CAB39
CNVR2441.1−RICTOR
CNVR7226.4−RPTOR
CNVR7150.1−RPS6KB1
CNVR7226.5−RPTOR
CNVR2016.1−EIF4E
CNVR7226.2−RPTOR
CNVR3163.1−RPS6KA2
CNVR3159.1−RPS6KA2
CNVR567.1−AKT3
CEU
YRI
CHB
0.0
0.2
0.4
0.6
0.8
1.0

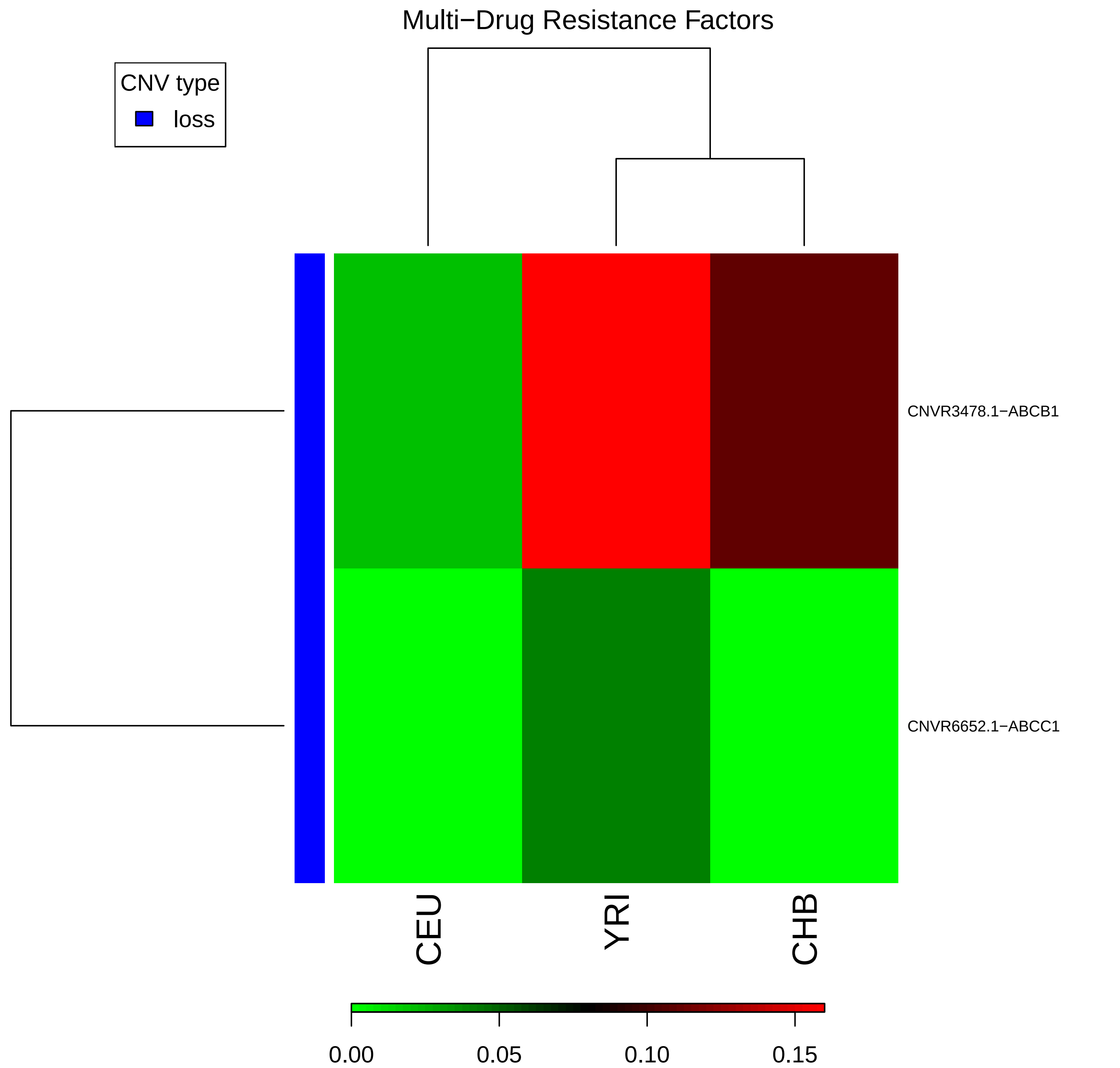
Multi−Drug Resistance Factors
CNV type
loss
CNVR3478.1−ABCB1
CNVR6652.1−ABCC1
CEU
YRI
CHB
0.00
0.05
0.10
0.15

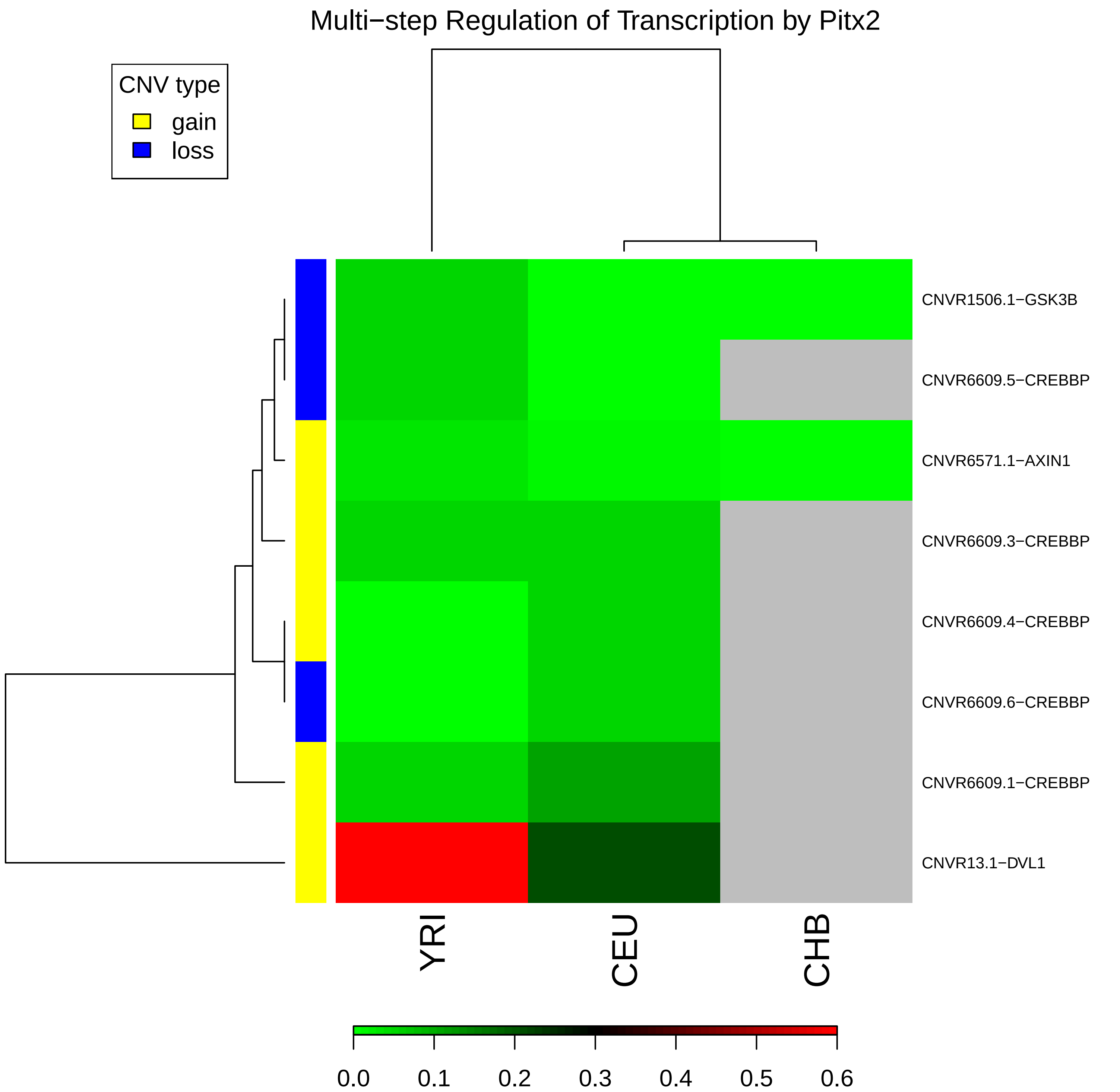

Multi−step Regulation of Transcription by Pitx2
CNV type
gain
loss
CNVR1506.1−GSK3B
CNVR6609.5−CREBBP
CNVR6571.1−AXIN1
CNVR6609.3−CREBBP
CNVR6609.4−CREBBP
CNVR6609.6−CREBBP
CNVR6609.1−CREBBP
CNVR13.1−DVL1
YRI
CEU
CHB
0.0
0.1
0.2
0.3
0.4
0.5
0.6

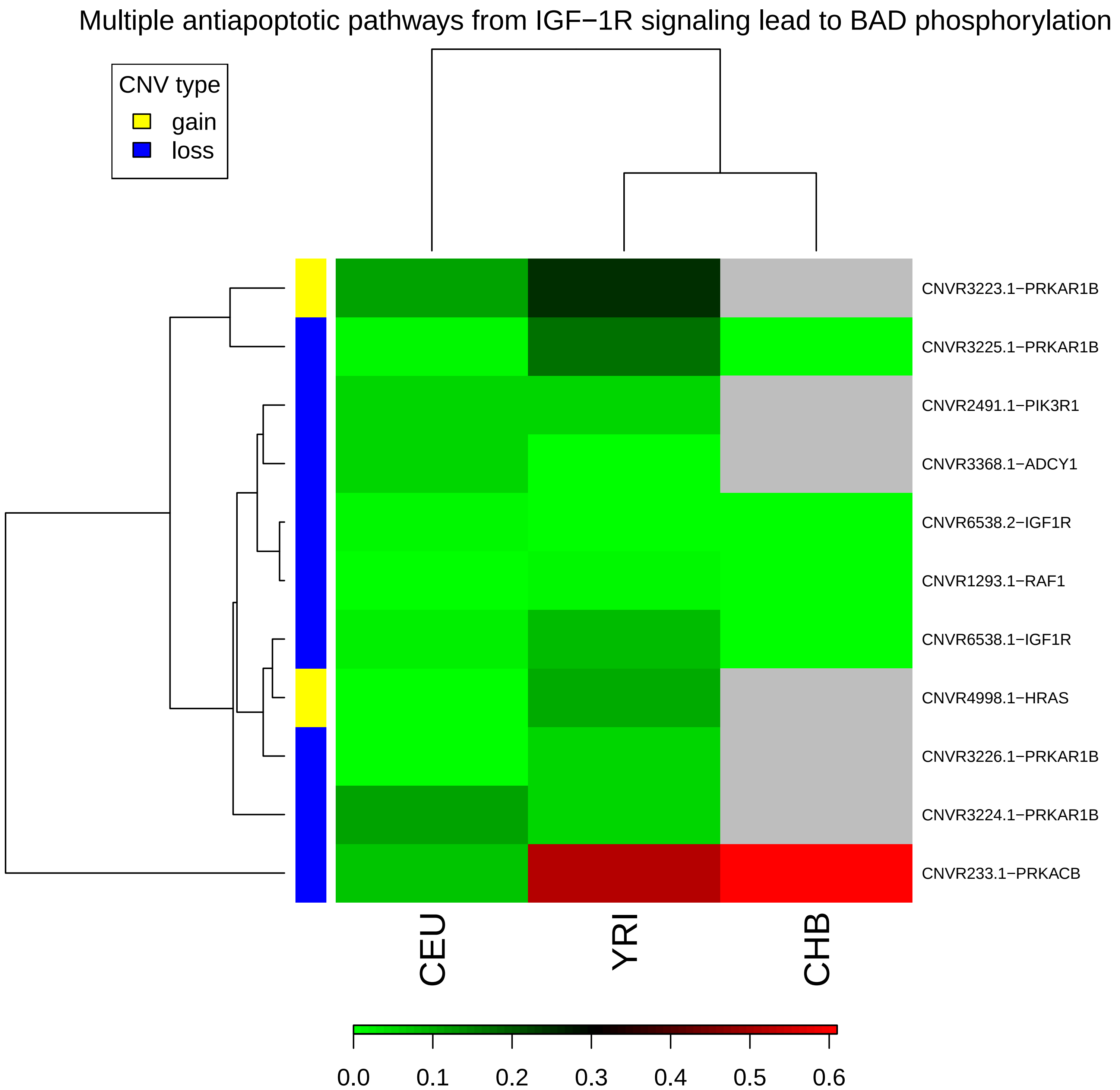
Multiple antiapoptotic pathways from IGF−1R signaling lead to BAD phosphorylation
CNV type
gain
loss
CNVR3223.1−PRKAR1B
CNVR3225.1−PRKAR1B
CNVR2491.1−PIK3R1
CNVR3368.1−ADCY1
CNVR6538.2−IGF1R
CNVR1293.1−RAF1
CNVR6538.1−IGF1R
CNVR4998.1−HRAS
CNVR3226.1−PRKAR1B
CNVR3224.1−PRKAR1B
CNVR233.1−PRKACB
CEU
YRI
CHB
0.0
0.1
0.2
0.3
0.4
0.5
0.6

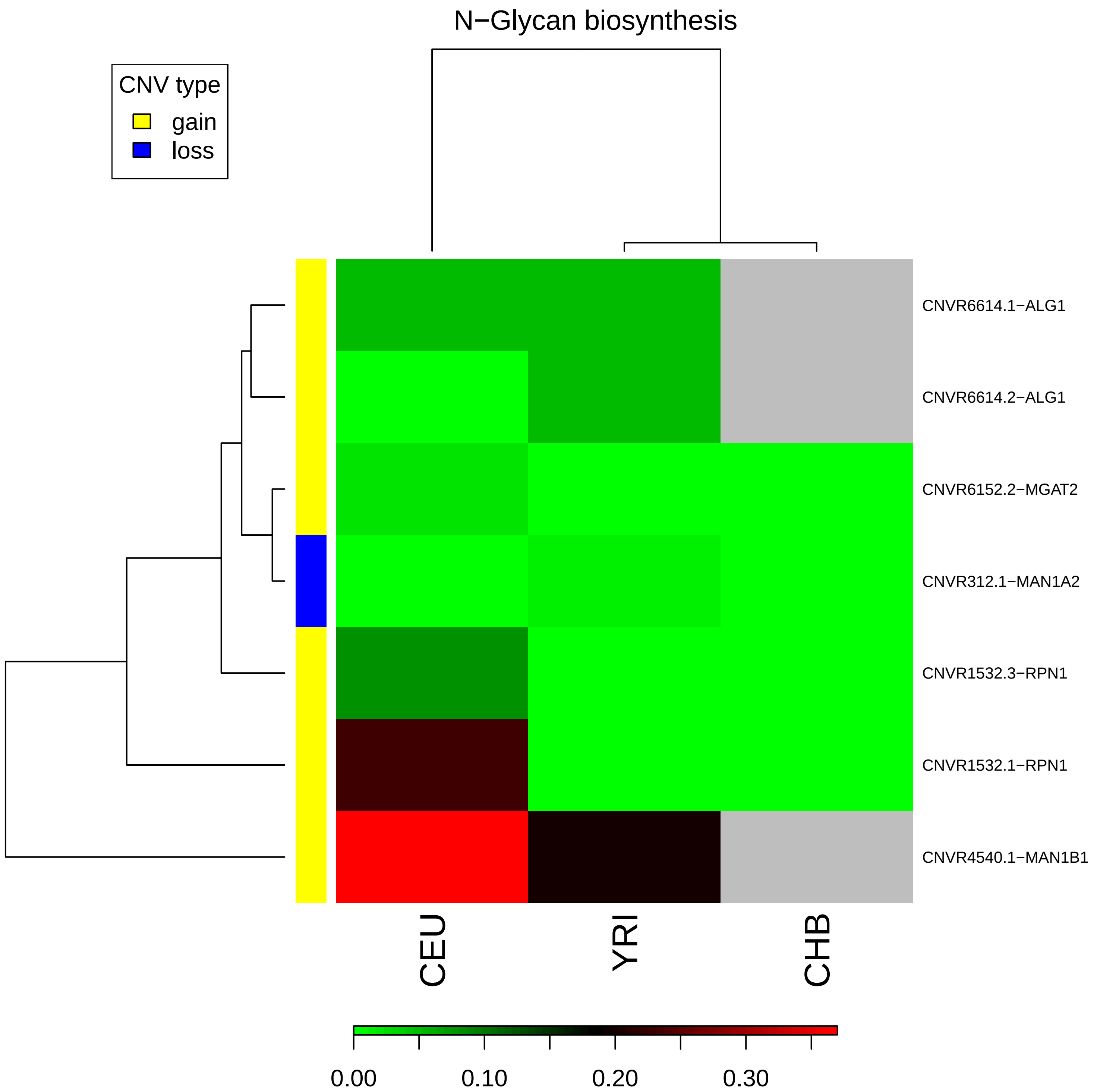
N−Glycan biosynthesis
CNV type
gain
loss
CNVR6614.1−ALG1
CNVR6614.2−ALG1
CNVR6152.2−MGAT2
CNVR312.1−MAN1A2
CNVR1532.3−RPN1
CNVR1532.1−RPN1
CNVR4540.1−MAN1B1
CEU
YRI
CHB
0.00
0.10
0.20
0.30

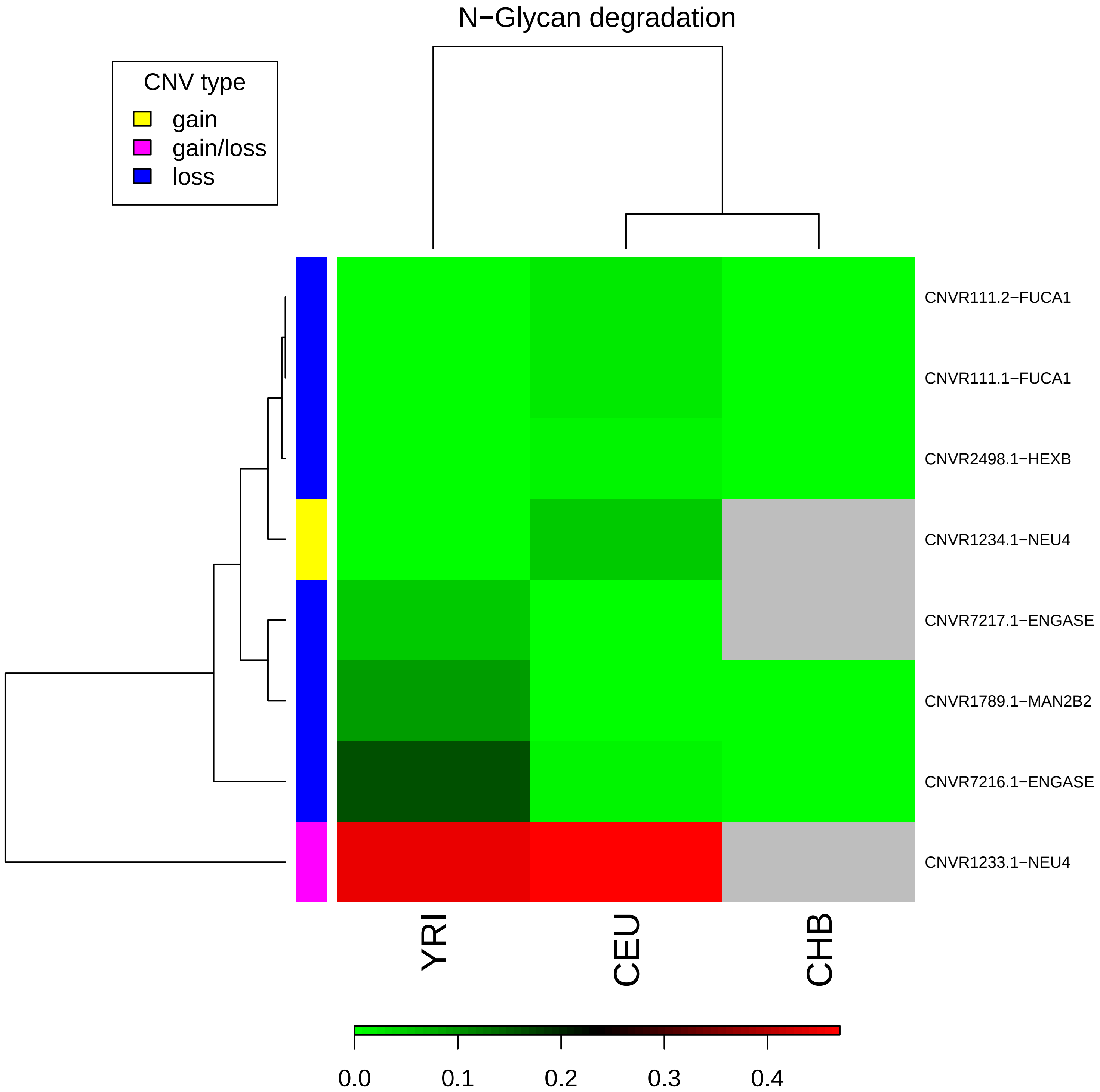

N−Glycan degradation
CNV type
gain
gain/loss
loss
CNVR111.2−FUCA1
CNVR111.1−FUCA1
CNVR2498.1−HEXB
CNVR1234.1−NEU4
CNVR7217.1−ENGASE
CNVR1789.1−MAN2B2
CNVR7216.1−ENGASE
CNVR1233.1−NEU4
YRI
CEU
CHB
0.0
0.1
0.2
0.3
0.4

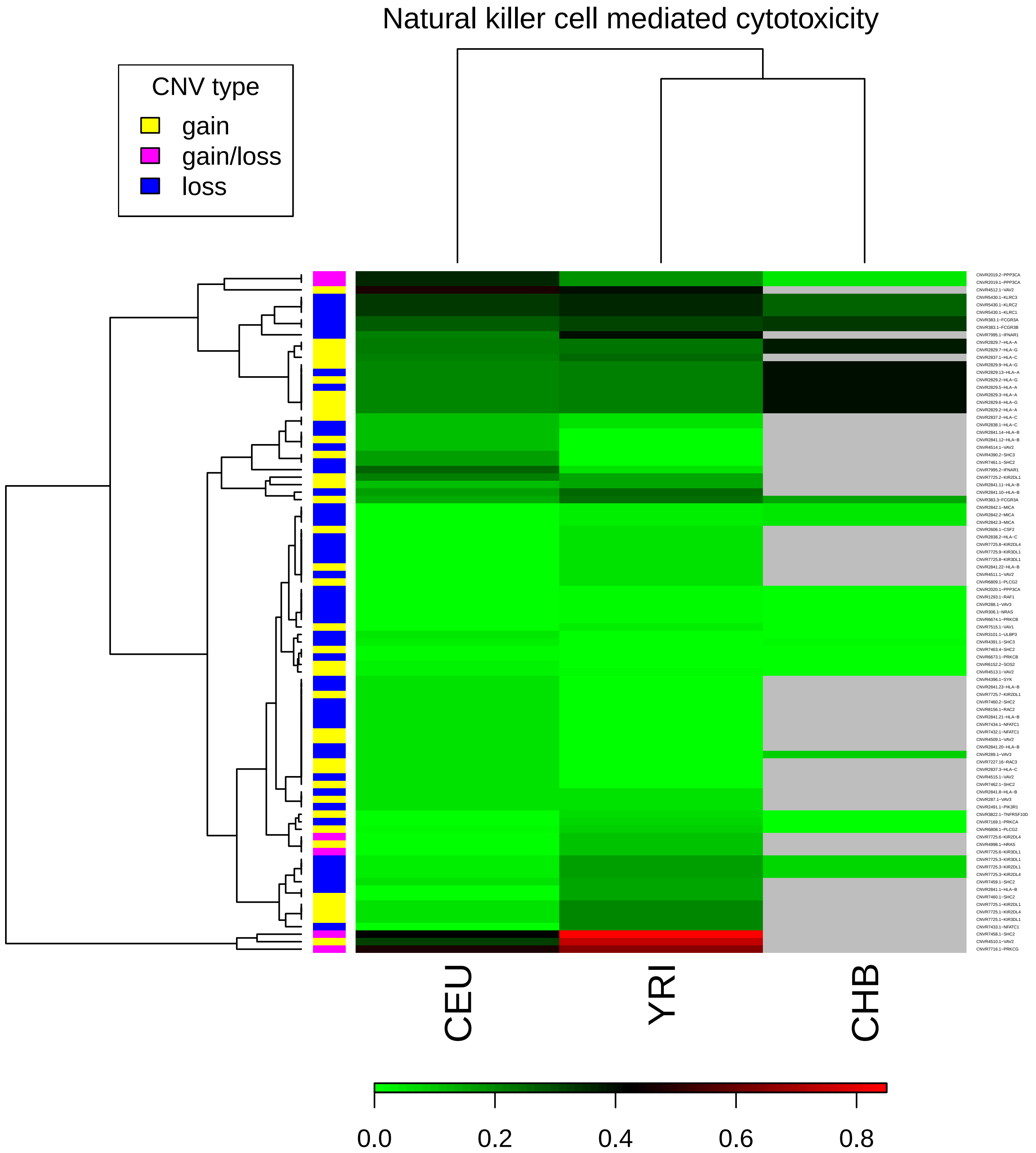

Natural killer cell mediated cytotoxicity
CNV type
gain
gain/loss
loss
CNVR2019.2−PPP3CA
CNVR2019.1−PPP3CA
CNVR4512.1−VAV2
CNVR5430.1−KLRC3
CNVR5430.1−KLRC2
CNVR5430.1−KLRC1
CNVR383.1−FCGR3A
CNVR383.1−FCGR3B
CNVR7995.1−IFNAR1
CNVR2829.7−HLA−A
CNVR2829.7−HLA−G
CNVR2837.1−HLA−C
CNVR2829.9−HLA−G
CNVR2829.13−HLA−A
CNVR2829.2−HLA−G
CNVR2829.5−HLA−A
CNVR2829.3−HLA−A
CNVR2829.6−HLA−G
CNVR2829.2−HLA−A
CNVR2837.2−HLA−C
CNVR2838.1−HLA−C
CNVR2841.14−HLA−B
CNVR2841.12−HLA−B
CNVR4514.1−VAV2
CNVR4390.2−SHC3
CNVR7461.1−SHC2
CNVR7995.2−IFNAR1
CNVR7725.2−KIR2DL1
CNVR2841.11−HLA−B
CNVR2841.10−HLA−B
CNVR383.3−FCGR3A
CNVR2842.1−MICA
CNVR2842.2−MICA
CNVR2842.3−MICA
CNVR2606.1−CSF2
CNVR2838.2−HLA−C
CNVR7725.8−KIR2DL4
CNVR7725.9−KIR3DL1
CNVR7725.8−KIR3DL1
CNVR2841.22−HLA−B
CNVR4511.1−VAV2
CNVR6809.1−PLCG2
CNVR2020.1−PPP3CA
CNVR1293.1−RAF1
CNVR288.1−VAV3
CNVR306.1−NRAS
CNVR6674.1−PRKCB
CNVR7515.1−VAV1
CNVR3101.1−ULBP3
CNVR4391.1−SHC3
CNVR7463.4−SHC2
CNVR6673.1−PRKCB
CNVR6152.2−SOS2
CNVR4513.1−VAV2
CNVR4396.1−SYK
CNVR2841.23−HLA−B
CNVR7725.7−KIR2DL1
CNVR7460.2−SHC2
CNVR8156.1−RAC2
CNVR2841.21−HLA−B
CNVR7434.1−NFATC1
CNVR7432.1−NFATC1
CNVR4509.1−VAV2
CNVR2841.20−HLA−B
CNVR289.1−VAV3
CNVR7227.16−RAC3
CNVR2837.3−HLA−C
CNVR4515.1−VAV2
CNVR7462.1−SHC2
CNVR2841.8−HLA−B
CNVR287.1−VAV3
CNVR2491.1−PIK3R1
CNVR3822.1−TNFRSF10D
CNVR7169.1−PRKCA
CNVR6808.1−PLCG2
CNVR7725.6−KIR2DL4
CNVR4998.1−HRAS
CNVR7725.6−KIR3DL1
CNVR7725.3−KIR3DL1
CNVR7725.3−KIR2DL1
CNVR7725.3−KIR2DL4
CNVR7459.1−SHC2
CNVR2841.1−HLA−B
CNVR7460.1−SHC2
CNVR7725.1−KIR2DL1
CNVR7725.1−KIR2DL4
CNVR7725.1−KIR3DL1
CNVR7433.1−NFATC1
CNVR7458.1−SHC2
CNVR4510.1−VAV2
CNVR7716.1−PRKCG
CEU
YRI
CHB
0.0
0.2
0.4
0.6
0.8

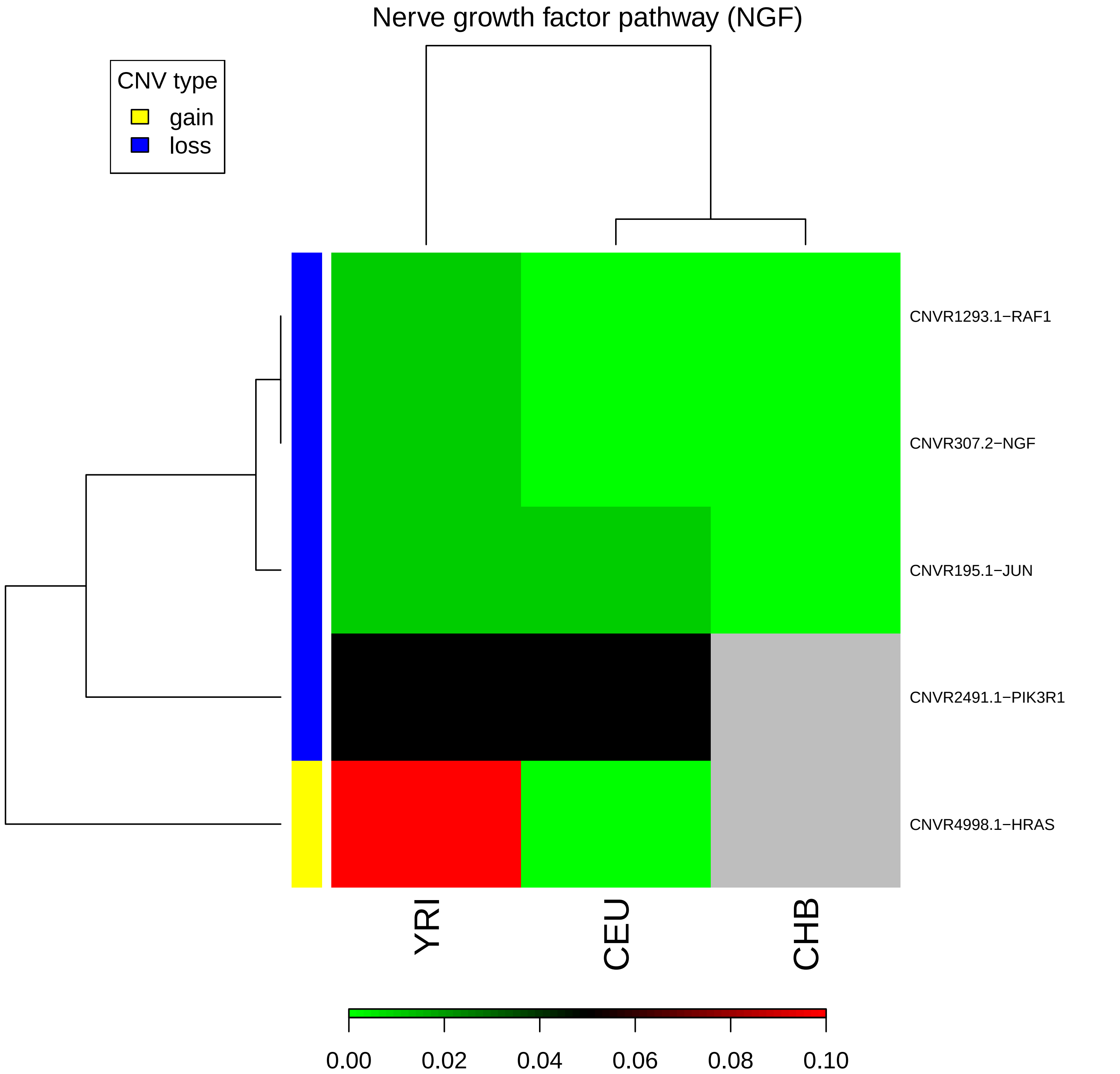
Nerve growth factor pathway (NGF)
CNV type
gain
loss
CNVR1293.1−RAF1
CNVR307.2−NGF
CNVR195.1−JUN
CNVR2491.1−PIK3R1
CNVR4998.1−HRAS
YRI
CEU
CHB
0.00
0.02
0.04
0.06
0.08
0.10

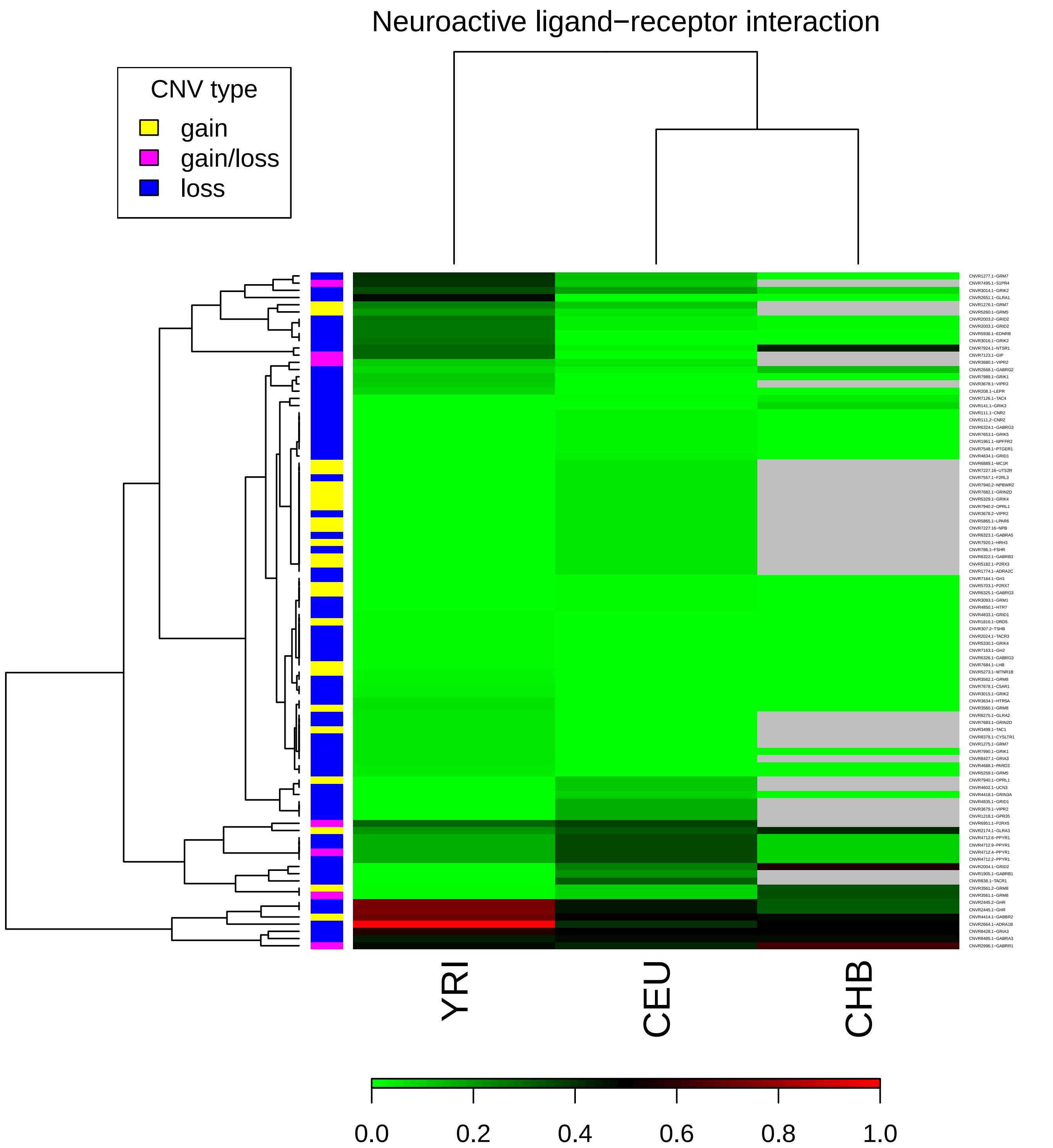
Neuroactive ligand−receptor interaction
CNV type
gain
gain/loss
loss
CNVR1277.1−GRM7
CNVR7495.1−S1PR4
CNVR3014.1−GRIK2
CNVR2651.1−GLRA1
CNVR1276.1−GRM7
CNVR5260.1−GRM5
CNVR2003.2−GRID2
CNVR2003.1−GRID2
CNVR5936.1−EDNRB
CNVR3016.1−GRIK2
CNVR7924.1−NTSR1
CNVR7123.1−GIP
CNVR3680.1−VIPR2
CNVR2668.1−GABRG2
CNVR7989.1−GRIK1
CNVR3678.1−VIPR2
CNVR208.1−LEPR
CNVR7126.1−TAC4
CNVR141.1−GRIK3
CNVR111.1−CNR2
CNVR111.2−CNR2
CNVR6324.1−GABRG3
CNVR7653.1−GRIK5
CNVR1961.1−NPFFR2
CNVR7548.1−PTGER1
CNVR4834.1−GRID1
CNVR6889.1−MC1R
CNVR7227.16−UTS2R
CNVR7557.1−F2RL3
CNVR7940.2−NPBWR2
CNVR7682.1−GRIN2D
CNVR5329.1−GRIK4
CNVR7940.2−OPRL1
CNVR3678.2−VIPR2
CNVR5865.1−LPAR6
CNVR7227.16−NPB
CNVR6323.1−GABRA5
CNVR7920.1−HRH3
CNVR786.1−FSHR
CNVR6322.1−GABRB3
CNVR5182.1−P2RX3
CNVR1774.1−ADRA2C
CNVR7164.1−GH1
CNVR5703.1−P2RX7
CNVR6325.1−GABRG3
CNVR3093.1−GRM1
CNVR4850.1−HTR7
CNVR4833.1−GRID1
CNVR1816.1−DRD5
CNVR307.2−TSHB
CNVR2024.1−TACR3
CNVR5330.1−GRIK4
CNVR7163.1−GH2
CNVR6326.1−GABRG3
CNVR7684.1−LHB
CNVR5273.1−MTNR1B
CNVR3562.1−GRM8
CNVR7678.1−C5AR1
CNVR3015.1−GRIK2
CNVR3634.1−HTR5A
CNVR3560.1−GRM8
CNVR8275.1−GLRA2
CNVR7683.1−GRIN2D
CNVR3499.1−TAC1
CNVR8378.1−CYSLTR1
CNVR1275.1−GRM7
CNVR7990.1−GRIK1
CNVR8427.1−GRIA3
CNVR4688.1−PARD3
CNVR5259.1−GRM5
CNVR7940.1−OPRL1
CNVR4602.1−UCN3
CNVR4418.1−GRIN3A
CNVR4835.1−GRID1
CNVR3679.1−VIPR2
CNVR1218.1−GPR35
CNVR6951.1−P2RX5
CNVR2174.1−GLRA3
CNVR4712.6−PPYR1
CNVR4712.9−PPYR1
CNVR4712.4−PPYR1
CNVR4712.2−PPYR1
CNVR2004.1−GRID2
CNVR1905.1−GABRB1
CNVR838.1−TACR1
CNVR3561.2−GRM8
CNVR3561.1−GRM8
CNVR2445.2−GHR
CNVR2445.1−GHR
CNVR4414.1−GABBR2
CNVR2664.1−ADRA1B
CNVR8428.1−GRIA3
CNVR8485.1−GABRA3
CNVR2996.1−GABRR1
YRI
CEU
CHB
0.0
0.2
0.4
0.6
0.8
1.0

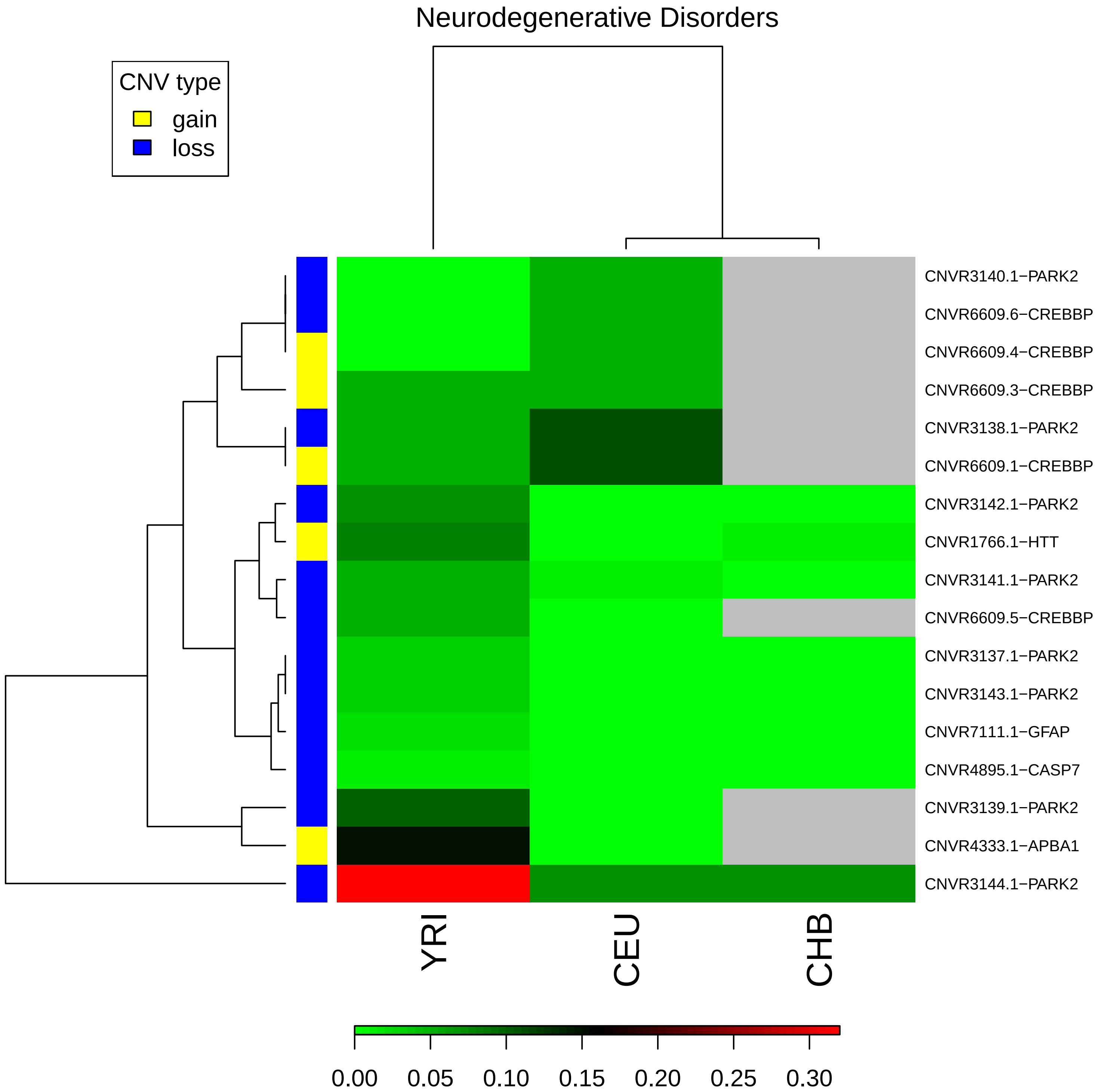

Neurodegenerative Disorders
CNV type
gain
loss
CNVR3140.1−PARK2
CNVR6609.6−CREBBP
CNVR6609.4−CREBBP
CNVR6609.3−CREBBP
CNVR3138.1−PARK2
CNVR6609.1−CREBBP
CNVR3142.1−PARK2
CNVR1766.1−HTT
CNVR3141.1−PARK2
CNVR6609.5−CREBBP
CNVR3137.1−PARK2
CNVR3143.1−PARK2
CNVR7111.1−GFAP
CNVR4895.1−CASP7
CNVR3139.1−PARK2
CNVR4333.1−APBA1
CNVR3144.1−PARK2
YRI
CEU
CHB
0.00
0.05
0.10
0.15
0.20
0.25
0.30

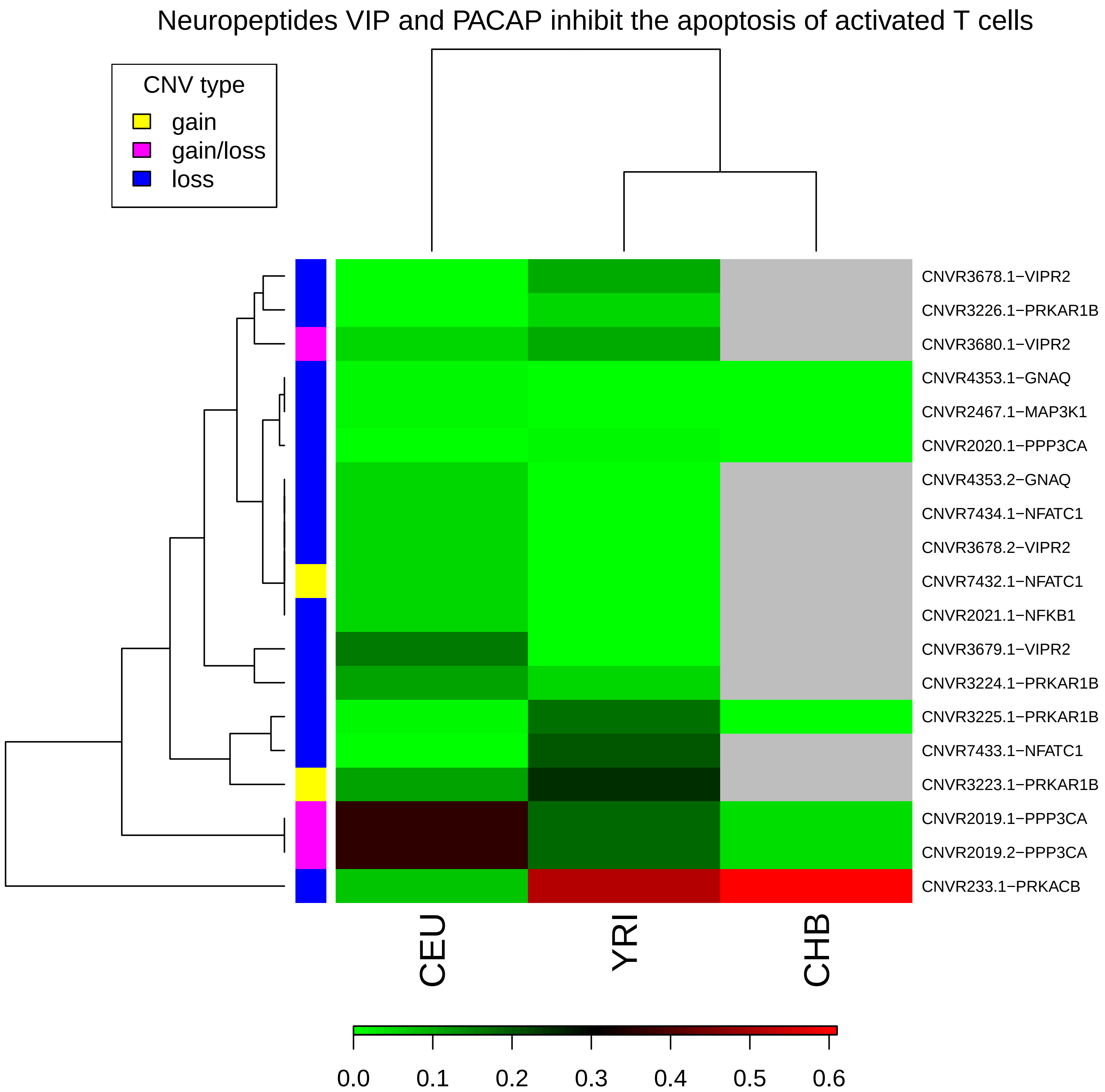

Neuropeptides VIP and PACAP inhibit the apoptosis of activated T cells
CNV type
gain
gain/loss
loss
CNVR3678.1−VIPR2
CNVR3226.1−PRKAR1B
CNVR3680.1−VIPR2
CNVR4353.1−GNAQ
CNVR2467.1−MAP3K1
CNVR2020.1−PPP3CA
CNVR4353.2−GNAQ
CNVR7434.1−NFATC1
CNVR3678.2−VIPR2
CNVR7432.1−NFATC1
CNVR2021.1−NFKB1
CNVR3679.1−VIPR2
CNVR3224.1−PRKAR1B
CNVR3225.1−PRKAR1B
CNVR7433.1−NFATC1
CNVR3223.1−PRKAR1B
CNVR2019.1−PPP3CA
CNVR2019.2−PPP3CA
CNVR233.1−PRKACB
CEU
YRI
CHB
0.0
0.1
0.2
0.3
0.4
0.5
0.6

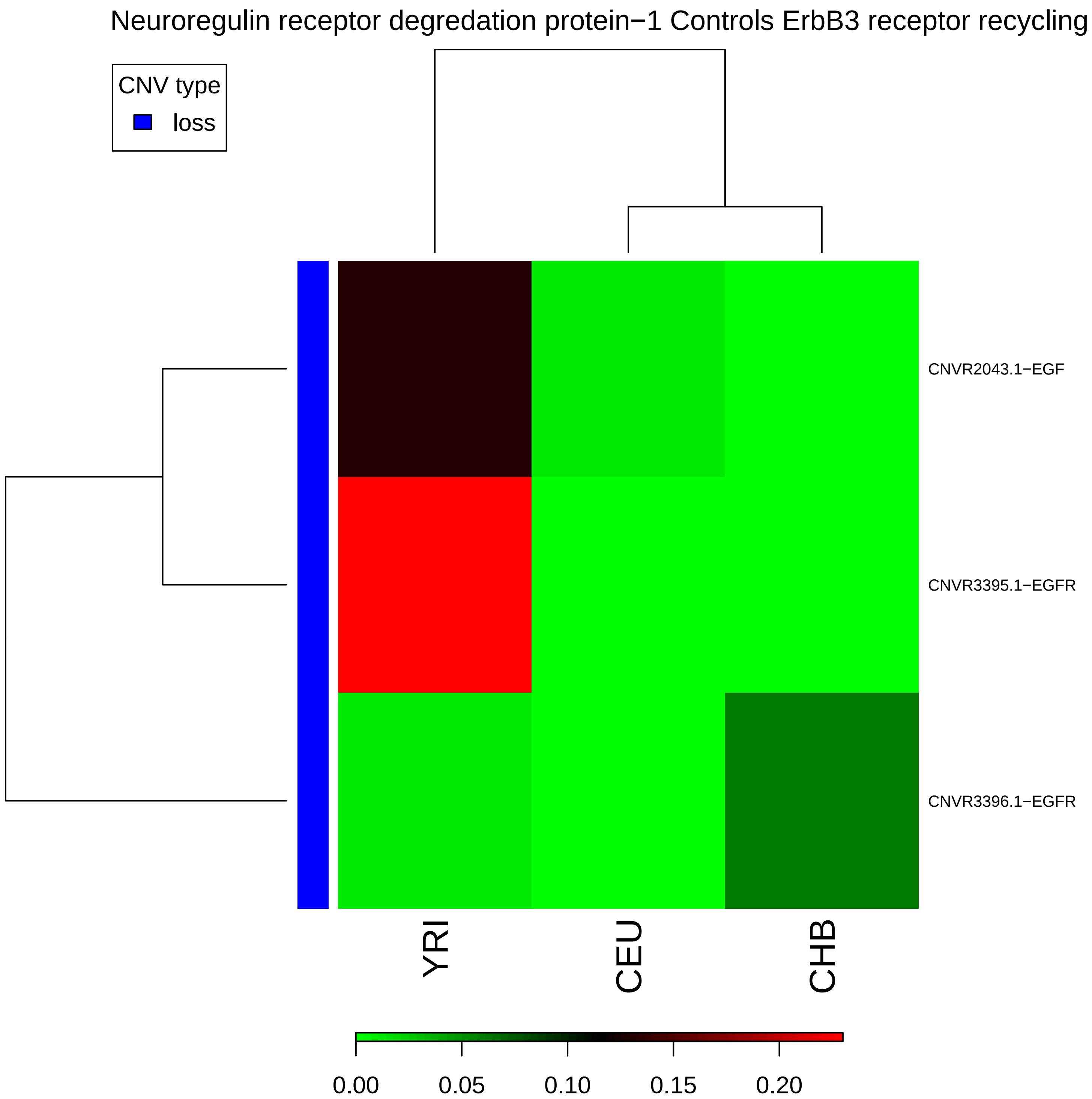
Neuroregulin receptor degradation protein−1 Controls ErbB3 receptor recycling
CNV type
loss
CNVR2043.1−EGF
CNVR3395.1−EGFR
CNVR3396.1−EGFR
YRI
CEU
CHB
0.00
0.05
0.10
0.15
0.20

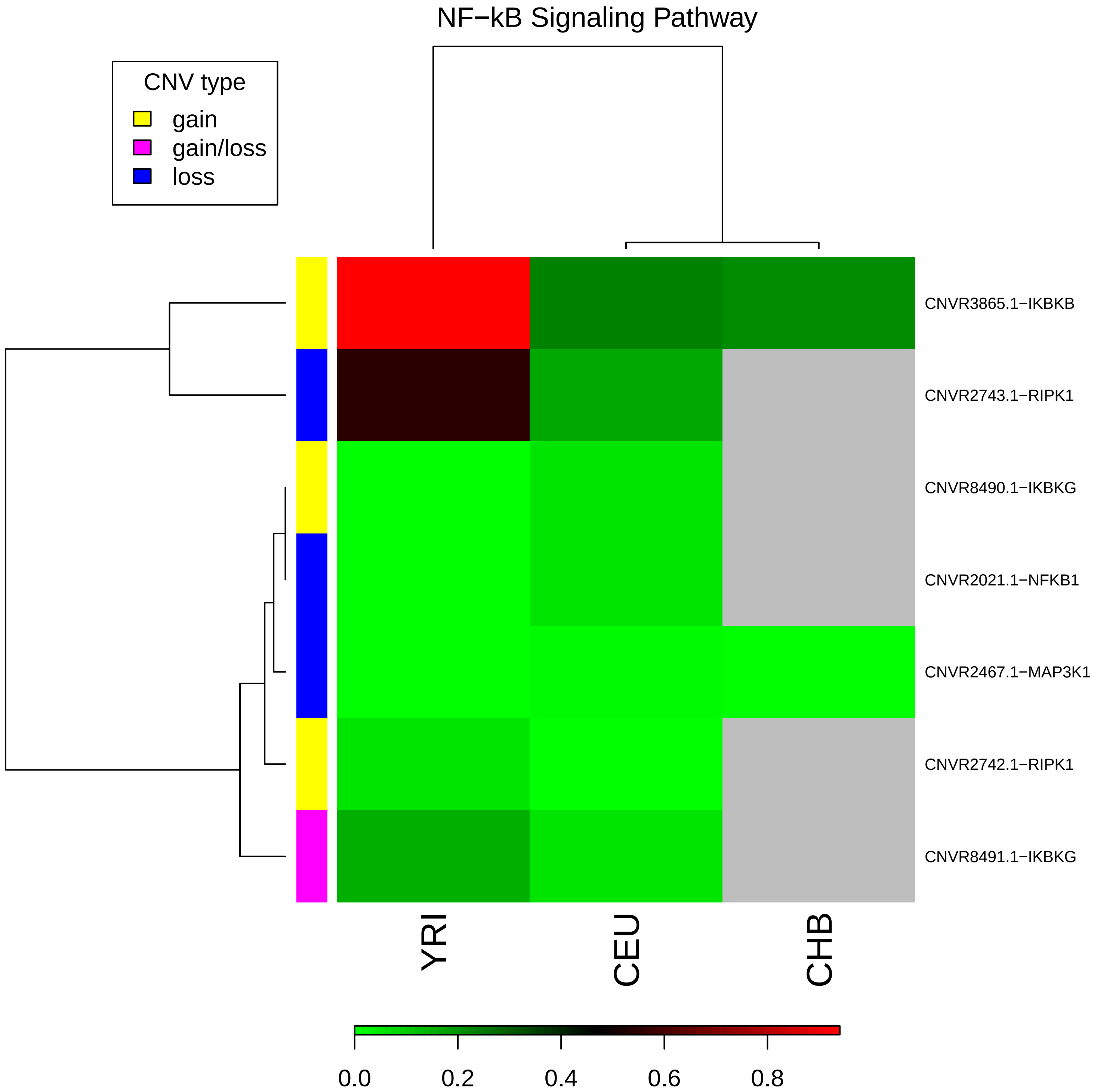
NF−kB Signaling Pathway
CNV type
gain
gain/loss
loss
CNVR3865.1−IKBKB
CNVR2743.1−RIPK1
CNVR8490.1−IKBKG
CNVR2021.1−NFKB1
CNVR2467.1−MAP3K1
CNVR2742.1−RIPK1
CNVR8491.1−IKBKG
YRI
CEU
CHB
0.0
0.2
0.4
0.6
0.8

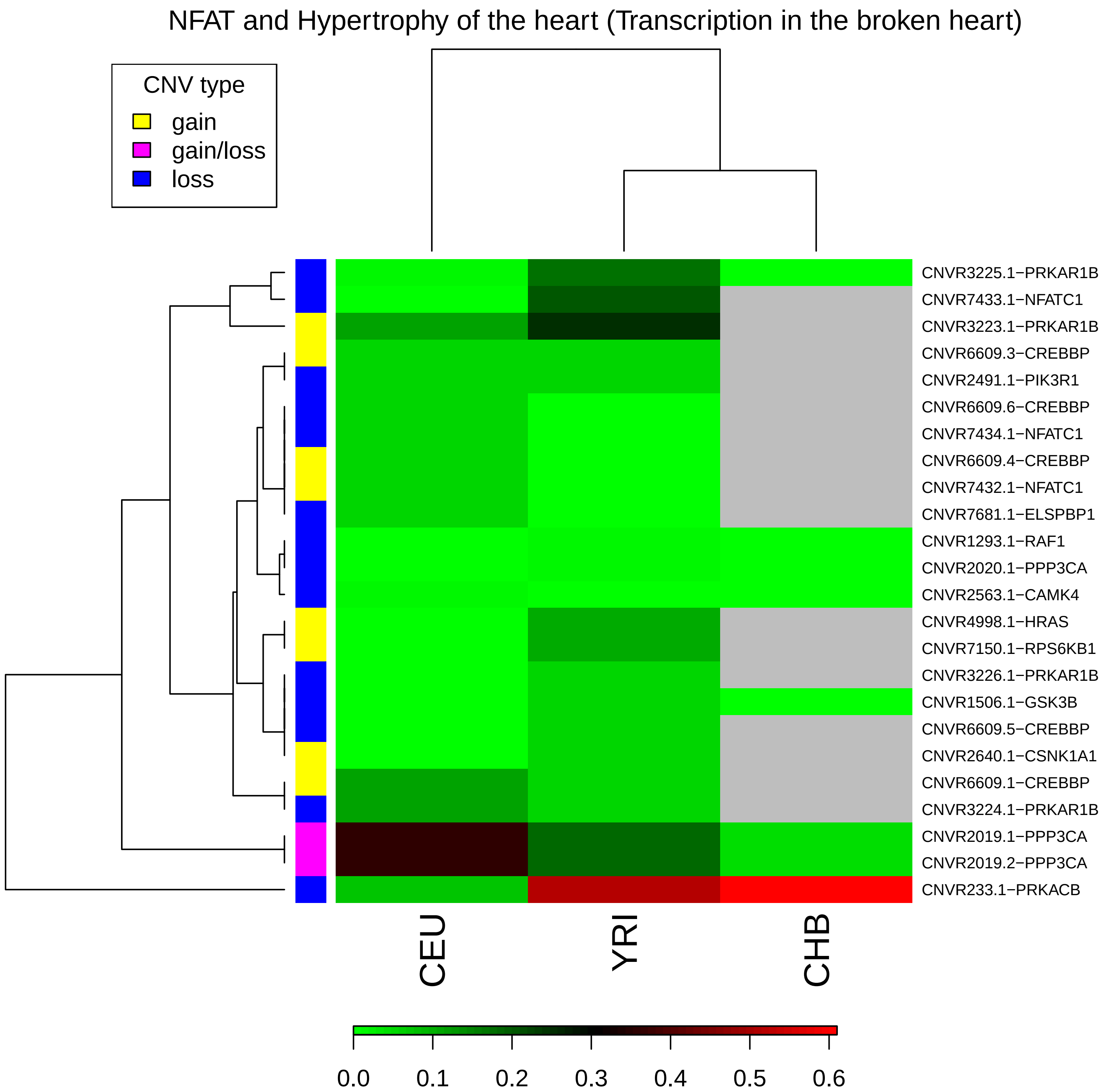

NFAT and Hypertrophy of the heart (Transcription in the broken heart)
CNV type
gain
gain/loss
loss
CNVR3225.1−PRKAR1B
CNVR7433.1−NFATC1
CNVR3223.1−PRKAR1B
CNVR6609.3−CREBBP
CNVR2491.1−PIK3R1
CNVR6609.6−CREBBP
CNVR7434.1−NFATC1
CNVR6609.4−CREBBP
CNVR7432.1−NFATC1
CNVR7681.1−ELSPBP1
CNVR1293.1−RAF1
CNVR2020.1−PPP3CA
CNVR2563.1−CAMK4
CNVR4998.1−HRAS
CNVR7150.1−RPS6KB1
CNVR3226.1−PRKAR1B
CNVR1506.1−GSK3B
CNVR6609.5−CREBBP
CNVR2640.1−CSNK1A1
CNVR6609.1−CREBBP
CNVR3224.1−PRKAR1B
CNVR2019.1−PPP3CA
CNVR2019.2−PPP3CA
CNVR233.1−PRKACB
CEU
YRI
CHB
0.0
0.1
0.2
0.3
0.4
0.5
0.6

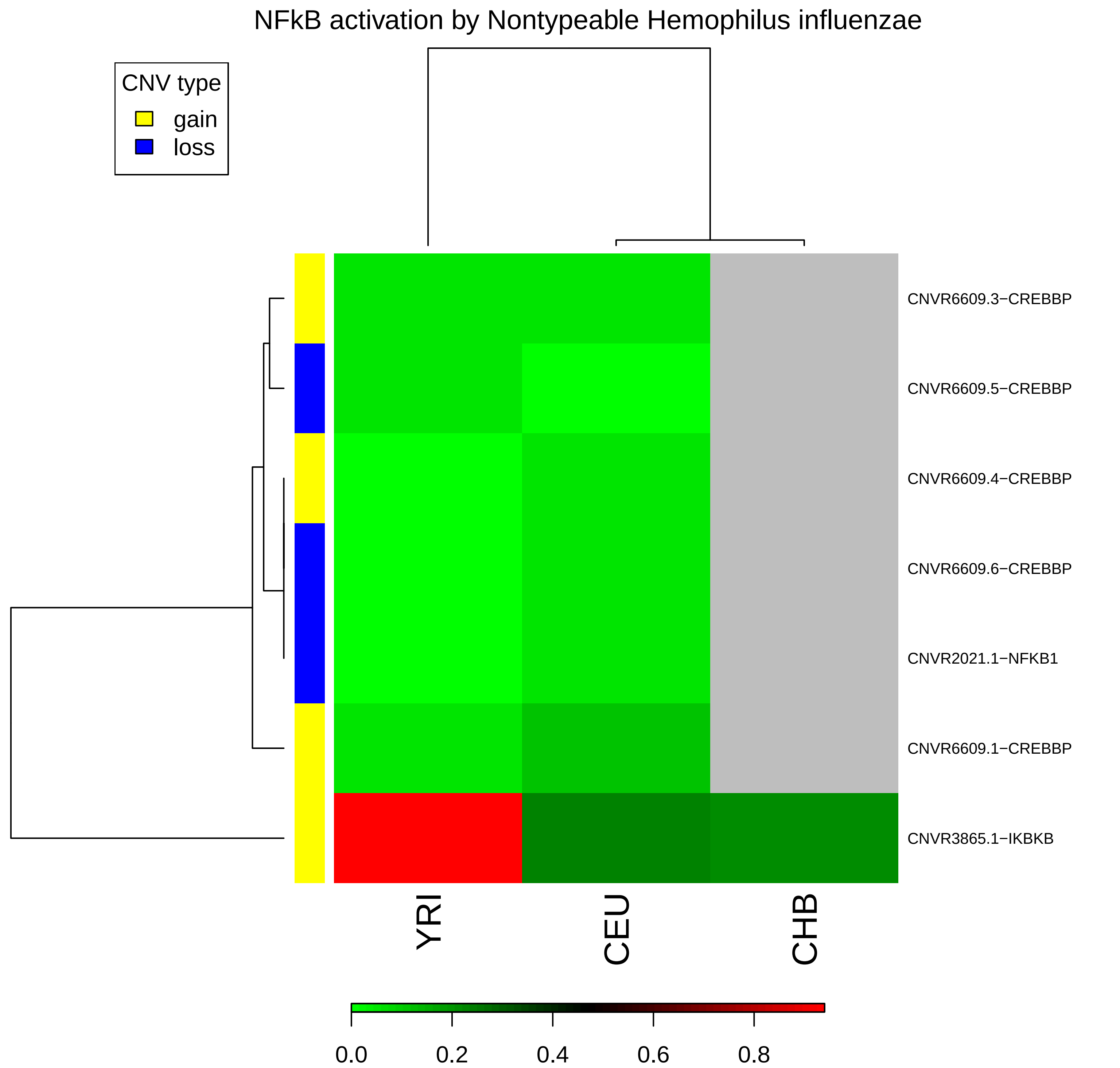
NFkB activation by Nontypeable Hemophilus influenzae
CNV type
gain
loss
CNVR6609.3−CREBBP
CNVR6609.5−CREBBP
CNVR6609.4−CREBBP
CNVR6609.6−CREBBP
CNVR2021.1−NFKB1
CNVR6609.1−CREBBP
CNVR3865.1−IKBKB
YRI
CEU
CHB
0.0
0.2
0.4
0.6
0.8

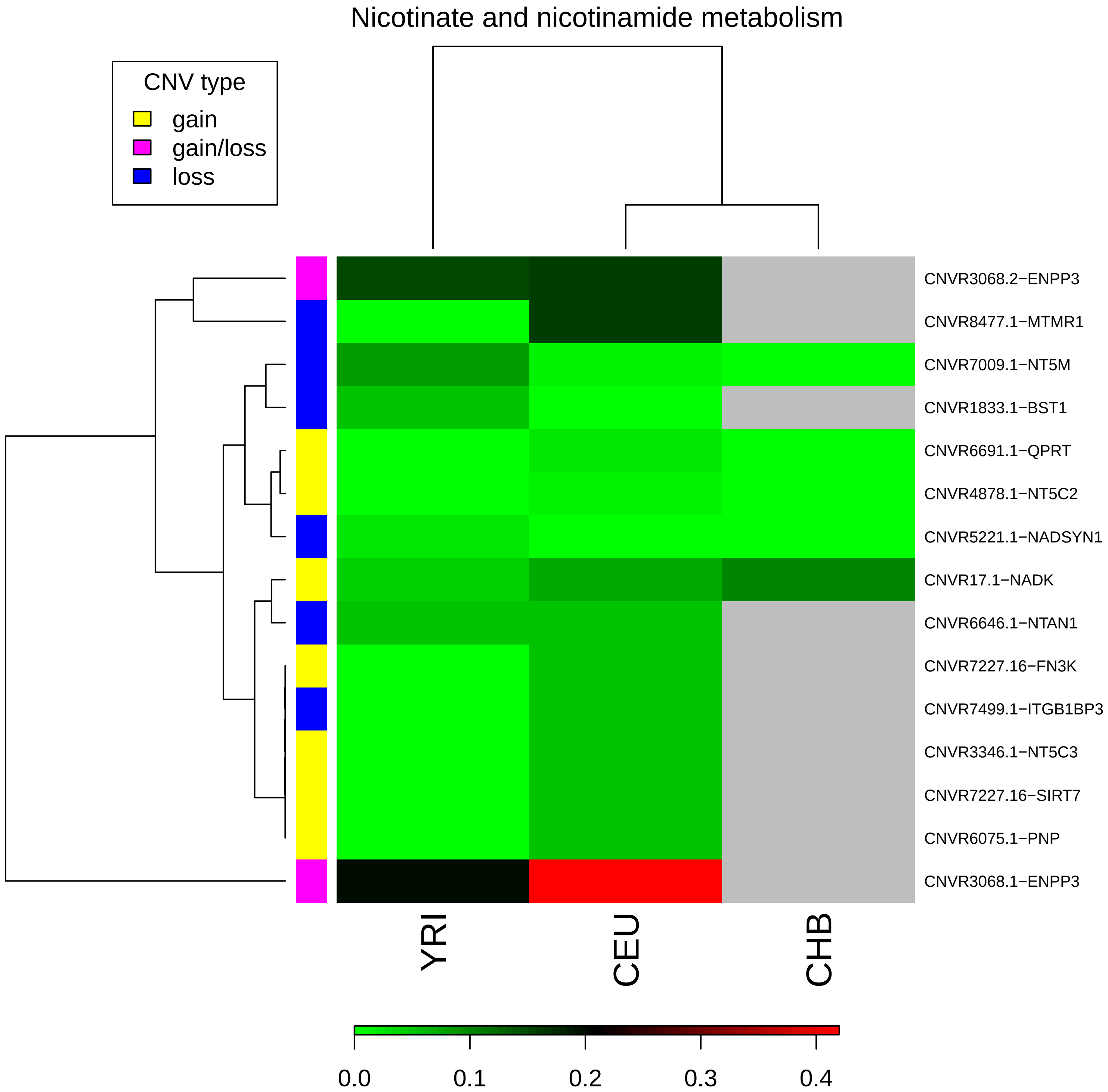
Nicotinate and nicotinamide metabolism
CNV type
gain
gain/loss
loss
CNVR3068.2−ENPP3
CNVR8477.1−MTMR1
CNVR7009.1−NT5M
CNVR1833.1−BST1
CNVR6691.1−QPRT
CNVR4878.1−NT5C2
CNVR5221.1−NADSYN1
CNVR17.1−NADK
CNVR6646.1−NTAN1
CNVR7227.16−FN3K
CNVR7499.1−ITGB1BP3
CNVR3346.1−NT5C3
CNVR7227.16−SIRT7
CNVR6075.1−PNP
CNVR3068.1−ENPP3
YRI
CEU
CHB
0.0
0.1
0.2
0.3
0.4

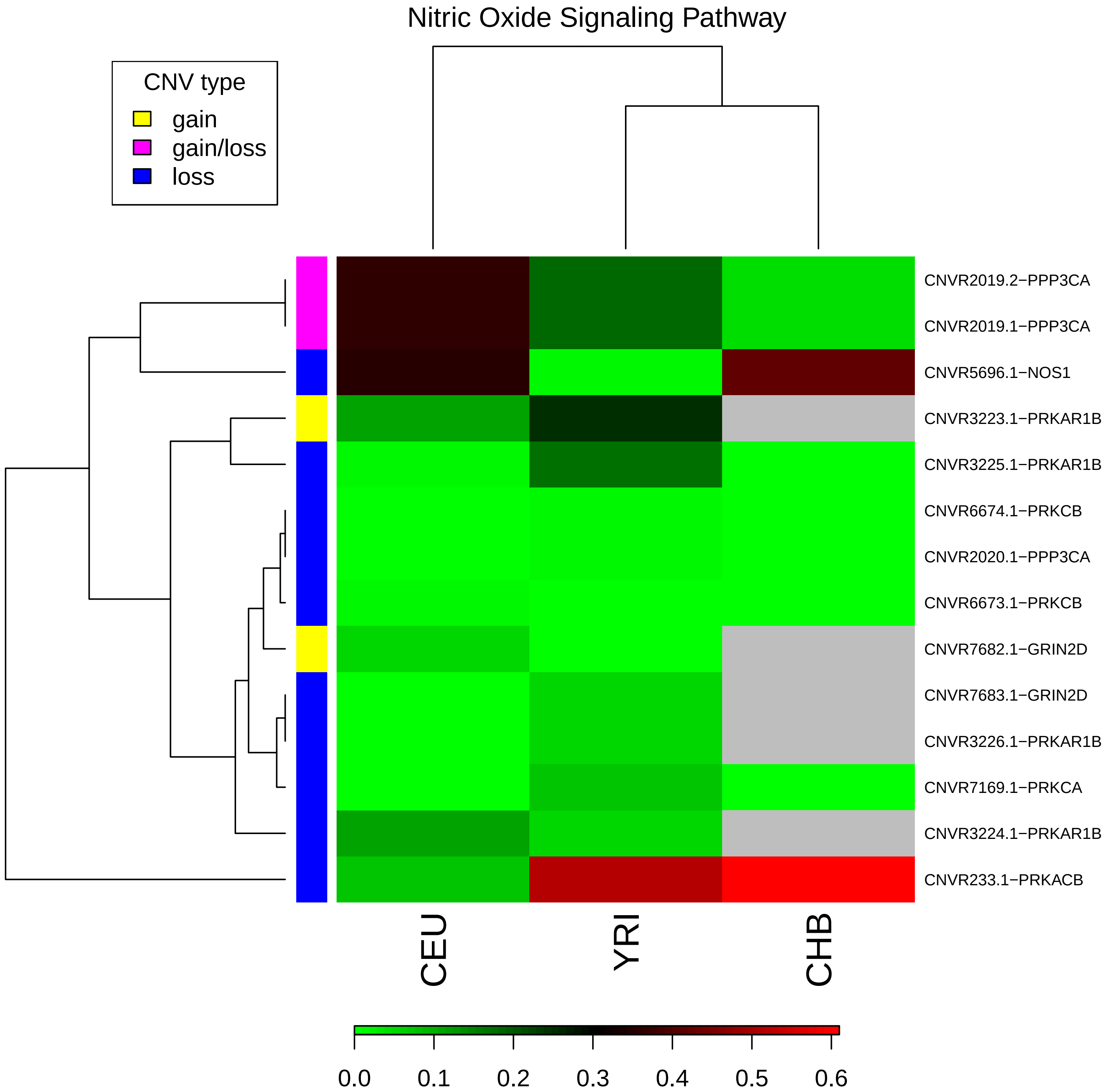

Nitric Oxide Signaling Pathway
CNV type
gain
gain/loss
loss
CNVR2019.2−PPP3CA
CNVR2019.1−PPP3CA
CNVR5696.1−NOS1
CNVR3223.1−PRKAR1B
CNVR3225.1−PRKAR1B
CNVR6674.1−PRKCB
CNVR2020.1−PPP3CA
CNVR6673.1−PRKCB
CNVR7682.1−GRIN2D
CNVR7683.1−GRIN2D
CNVR3226.1−PRKAR1B
CNVR7169.1−PRKCA
CNVR3224.1−PRKAR1B
CNVR233.1−PRKACB
CEU
YRI
CHB
0.0
0.1
0.2
0.3
0.4
0.5
0.6

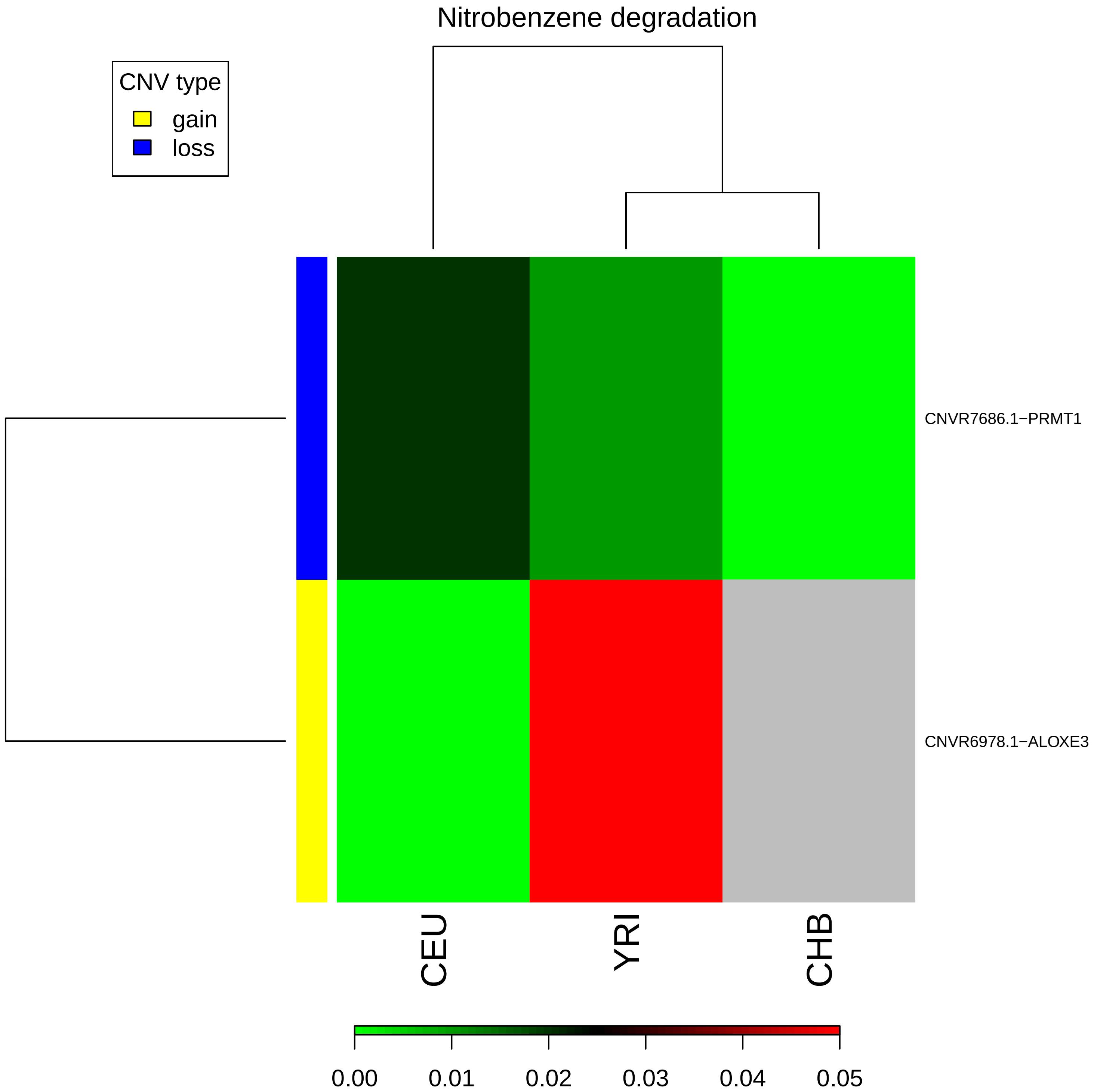
Nitrobenzene degradation
CNV type
gain
loss
CNVR7686.1−PRMT1
CNVR6978.1−ALOXE3
CEU
YRI
CHB
0.00
0.01
0.02
0.03
0.04
0.05

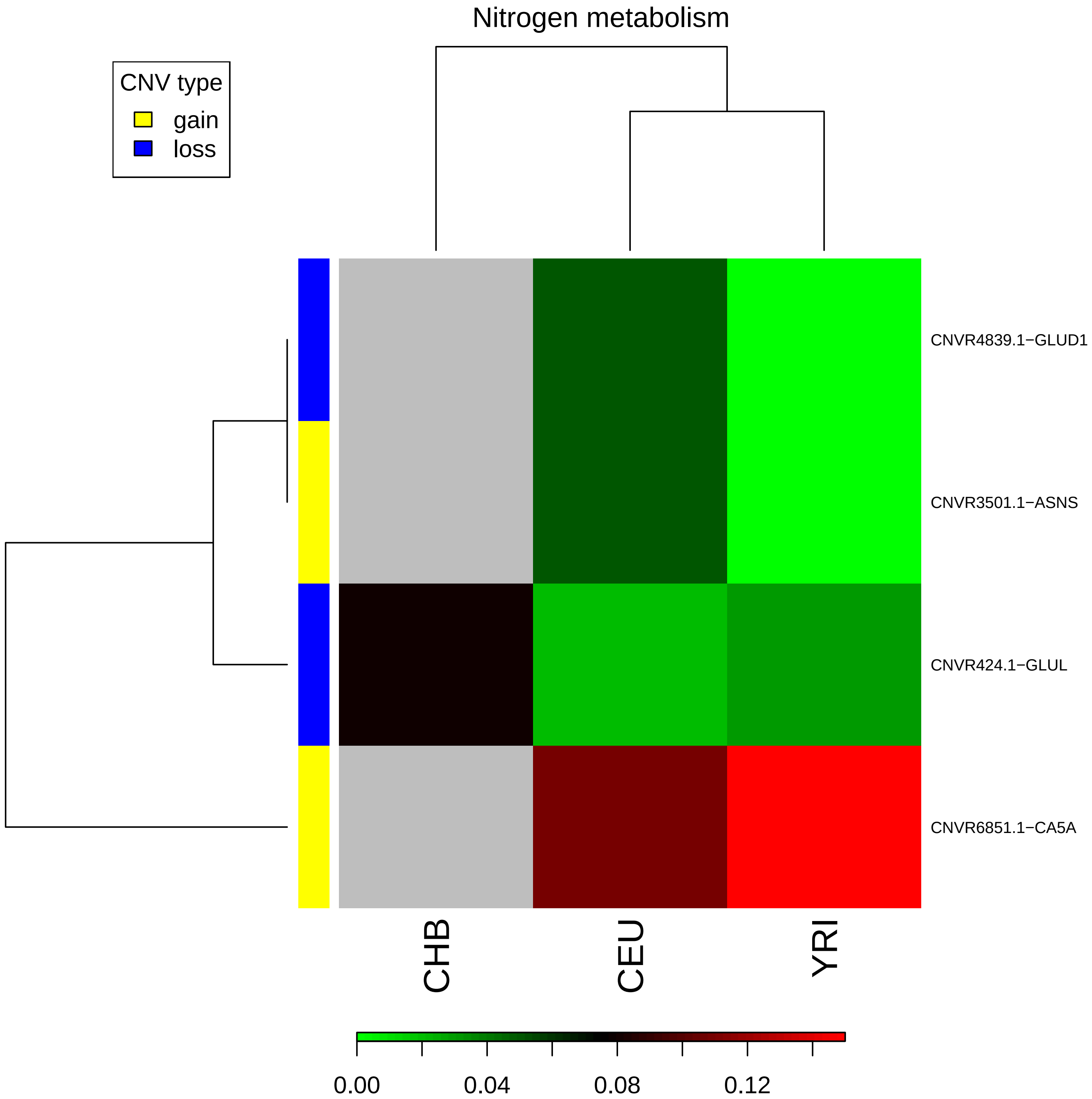

Nitrogen metabolism
CNV type
gain
loss
CNVR4839.1−GLUD1
CNVR3501.1−ASNS
CNVR424.1−GLUL
CNVR6851.1−CA5A
CHB
CEU
YRI
0.00
0.04
0.08
0.12

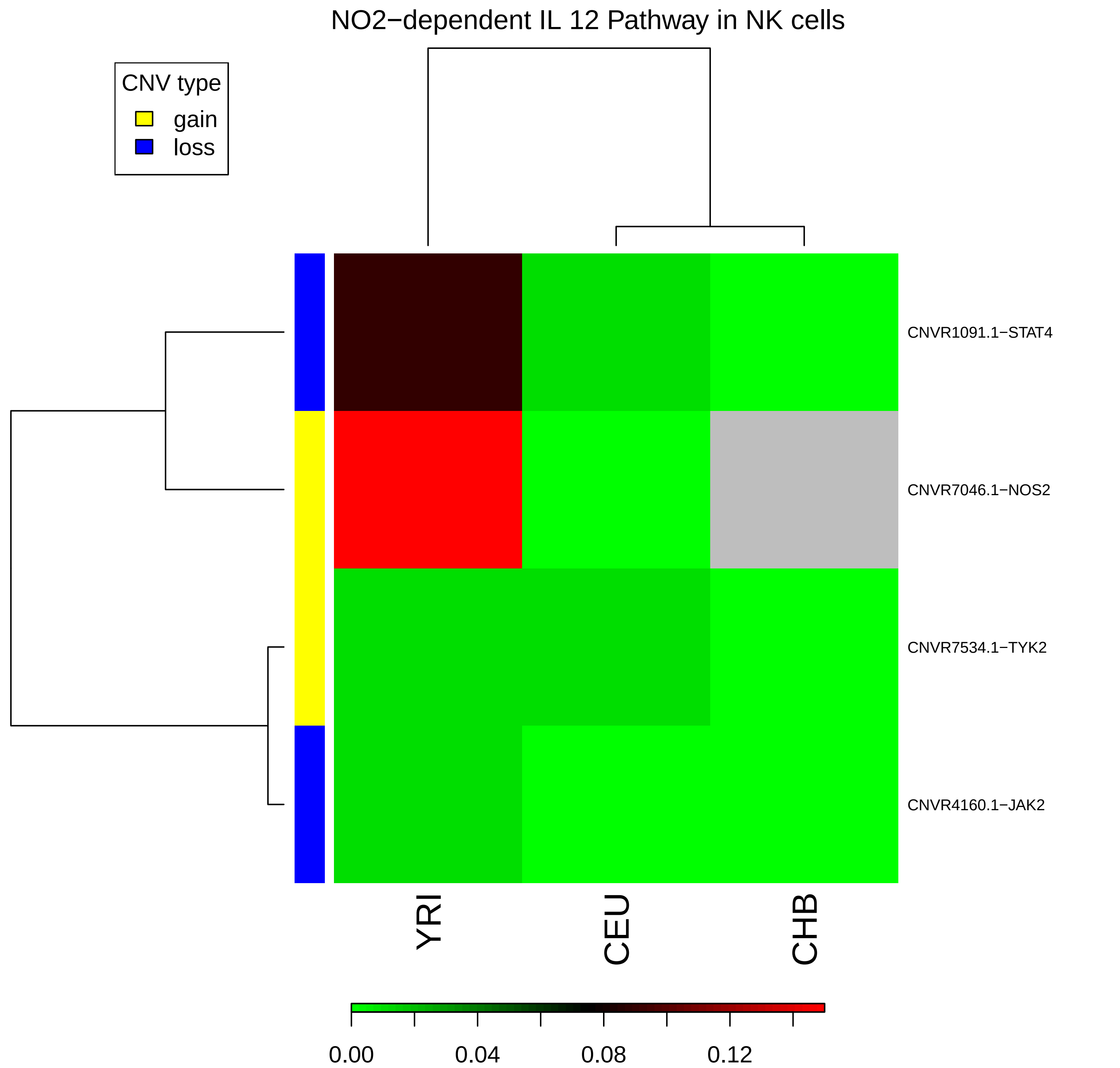

NO2−dependent IL 12 Pathway in NK cells
CNV type
gain
loss
CNVR1091.1−STAT4
CNVR7046.1−NOS2
CNVR7534.1−TYK2
CNVR4160.1−JAK2
YRI
CEU
CHB
0.00
0.04
0.08
0.12

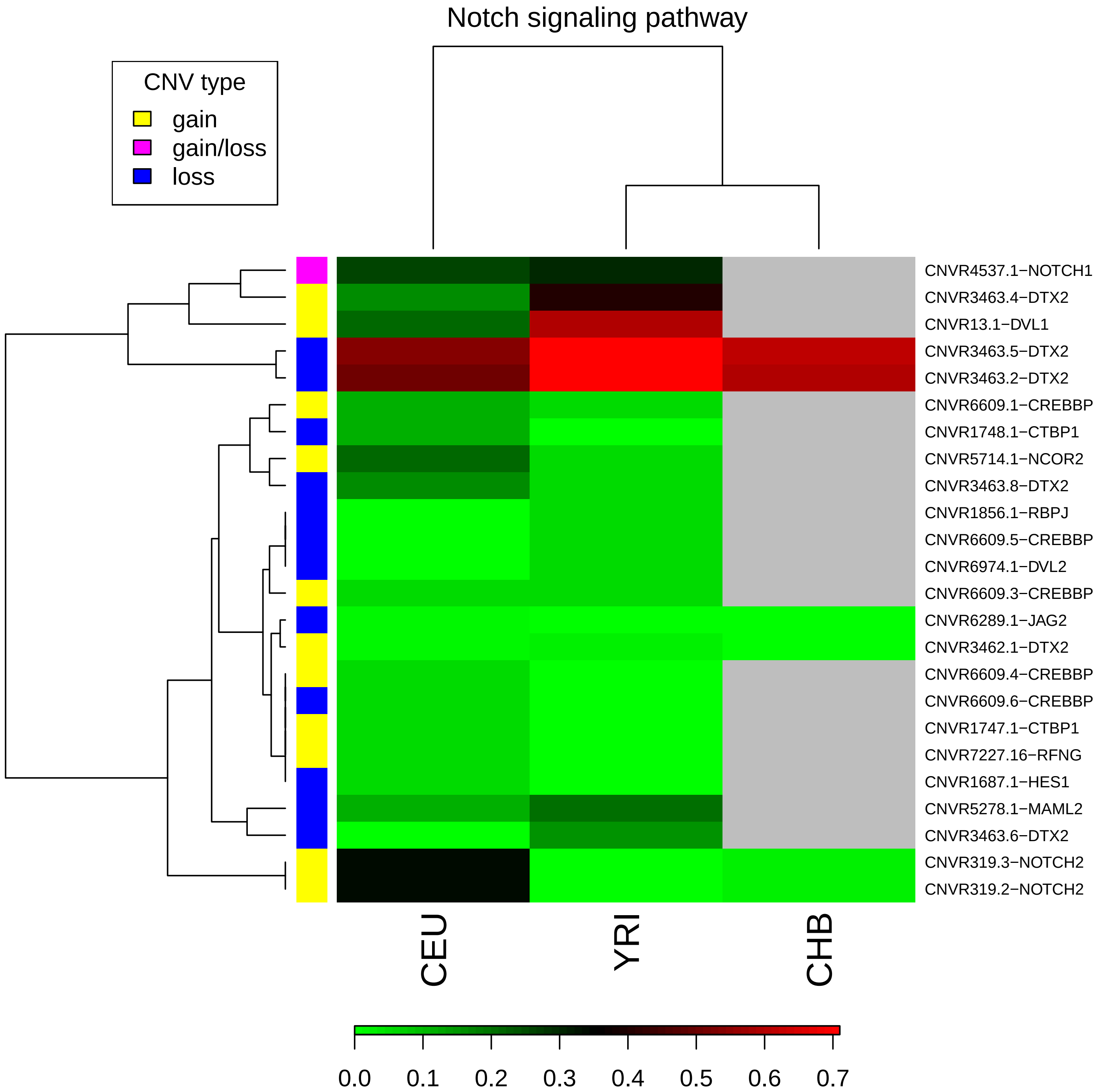

Notch signaling pathway
CNV type
gain
gain/loss
loss
CNVR4537.1−NOTCH1
CNVR3463.4−DTX2
CNVR13.1−DVL1
CNVR3463.5−DTX2
CNVR3463.2−DTX2
CNVR6609.1−CREBBP
CNVR1748.1−CTBP1
CNVR5714.1−NCOR2
CNVR3463.8−DTX2
CNVR1856.1−RBPJ
CNVR6609.5−CREBBP
CNVR6974.1−DVL2
CNVR6609.3−CREBBP
CNVR6289.1−JAG2
CNVR3462.1−DTX2
CNVR6609.4−CREBBP
CNVR6609.6−CREBBP
CNVR1747.1−CTBP1
CNVR7227.16−RFNG
CNVR1687.1−HES1
CNVR5278.1−MAML2
CNVR3463.6−DTX2
CNVR319.3−NOTCH2
CNVR319.2−NOTCH2
CEU
YRI
CHB
0.0
0.1
0.2
0.3
0.4
0.5
0.6
0.7

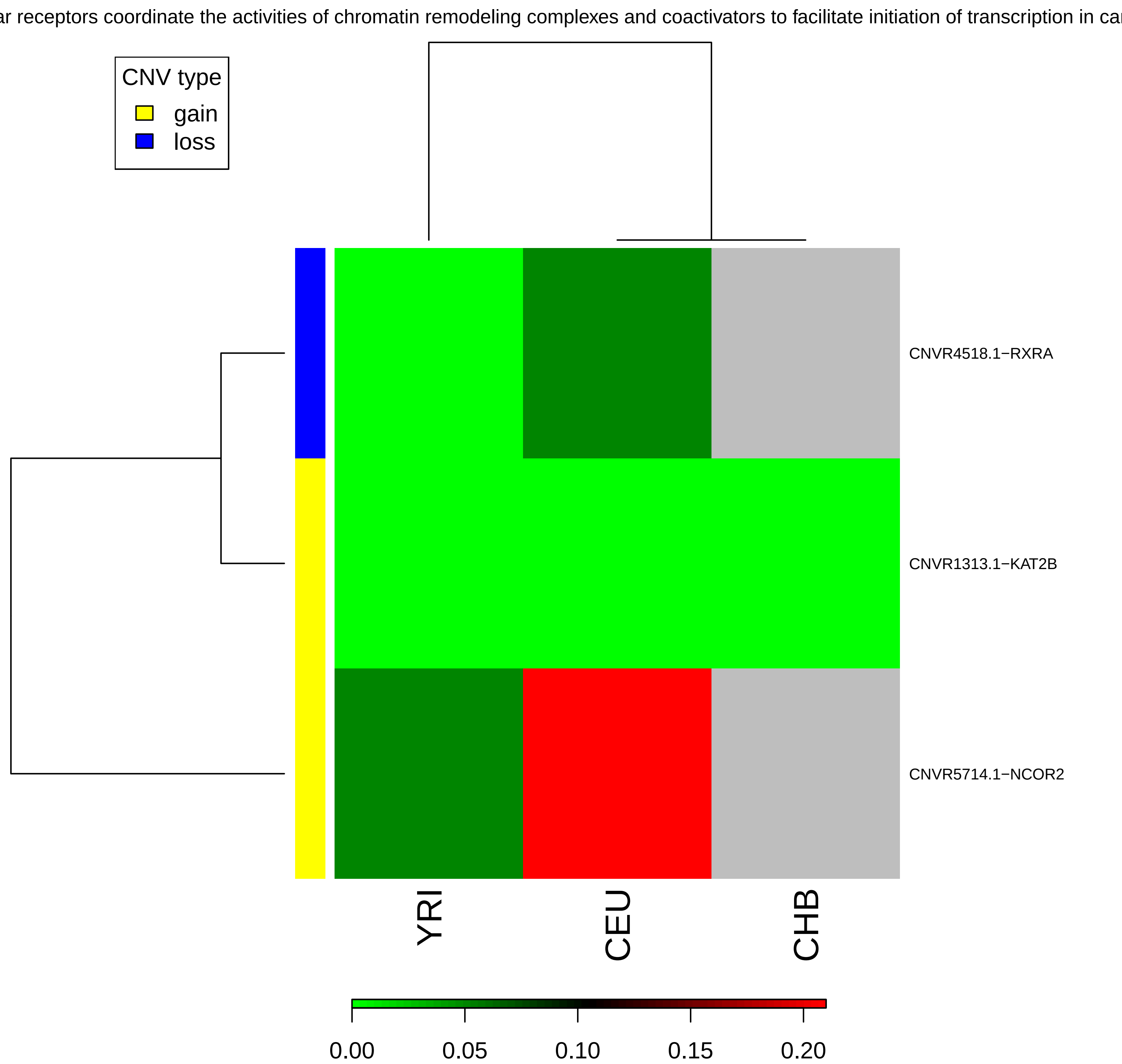

ar receptors coordinate the activities of chromatin remodeling complexes and coactivators to facilitate initiation of transcription in ca
CNV type
gain
loss
CNVR4518.1−RXRA
CNVR1313.1−KAT2B
CNVR5714.1−NCOR2
YRI
CEU
CHB
0.00
0.05
0.10
0.15
0.20

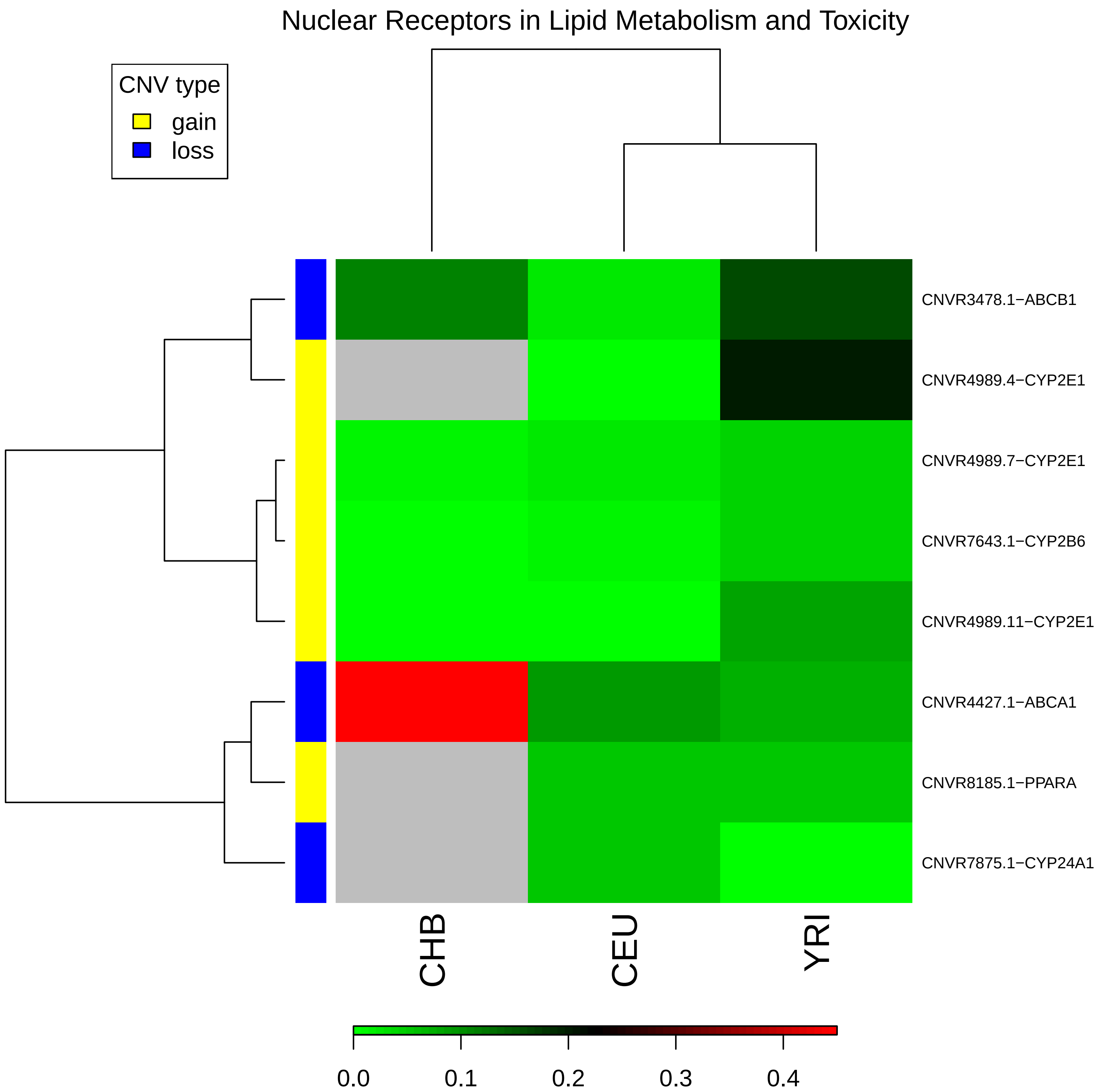

Nuclear Receptors in Lipid Metabolism and Toxicity
CNV type
gain
loss
CNVR3478.1−ABCB1
CNVR4989.4−CYP2E1
CNVR4989.7−CYP2E1
CNVR7643.1−CYP2B6
CNVR4989.11−CYP2E1
CNVR4427.1−ABCA1
CNVR8185.1−PPARA
CNVR7875.1−CYP24A1
CHB
CEU
YRI
0.0
0.1
0.2
0.3
0.4

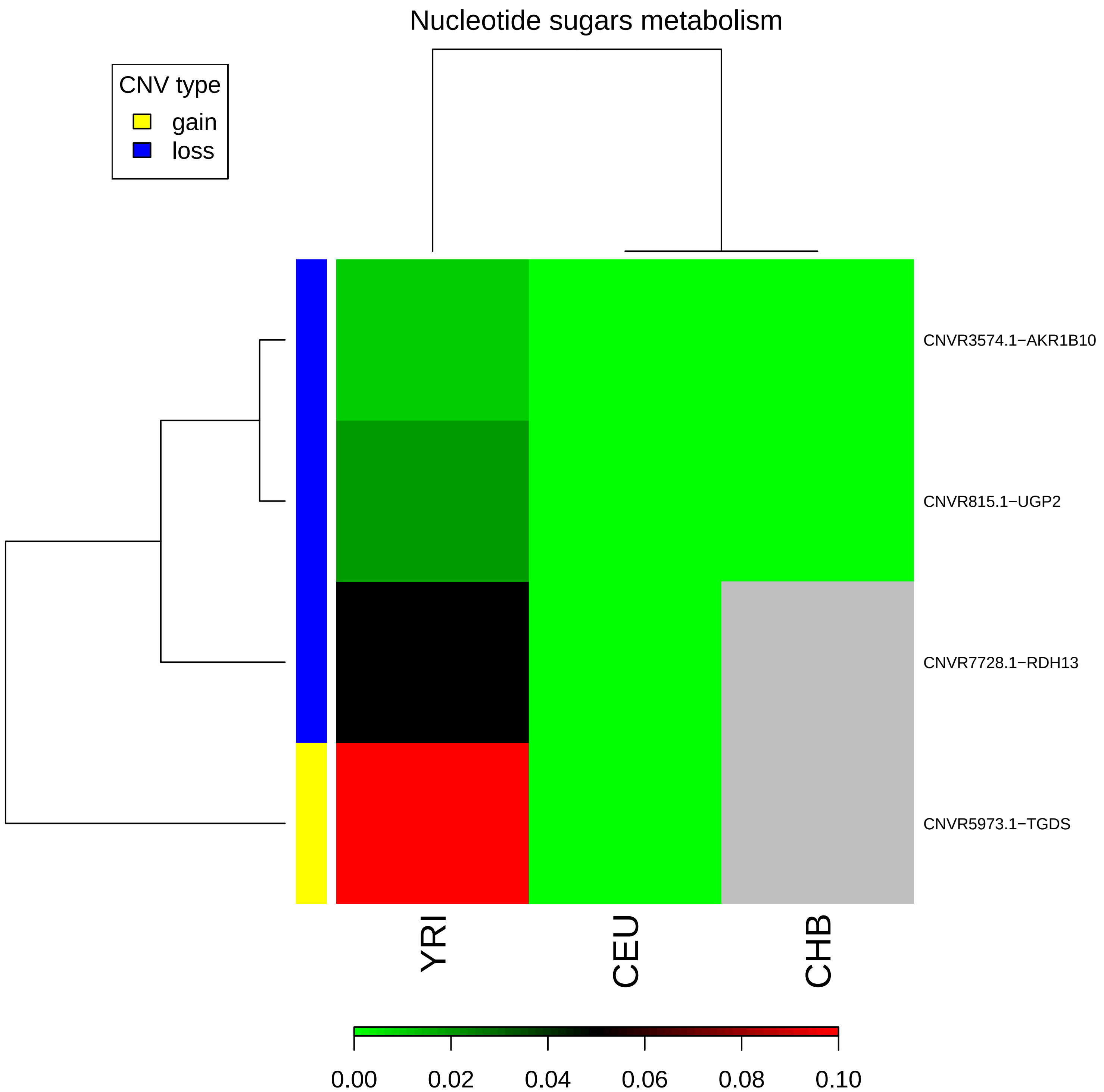
Nucleotide sugars metabolism
CNV type
gain
loss
CNVR3574.1−AKR1B10
CNVR815.1−UGP2
CNVR7728.1−RDH13
CNVR5973.1−TGDS
YRI
CEU
CHB
0.00
0.02
0.04
0.06
0.08
0.10

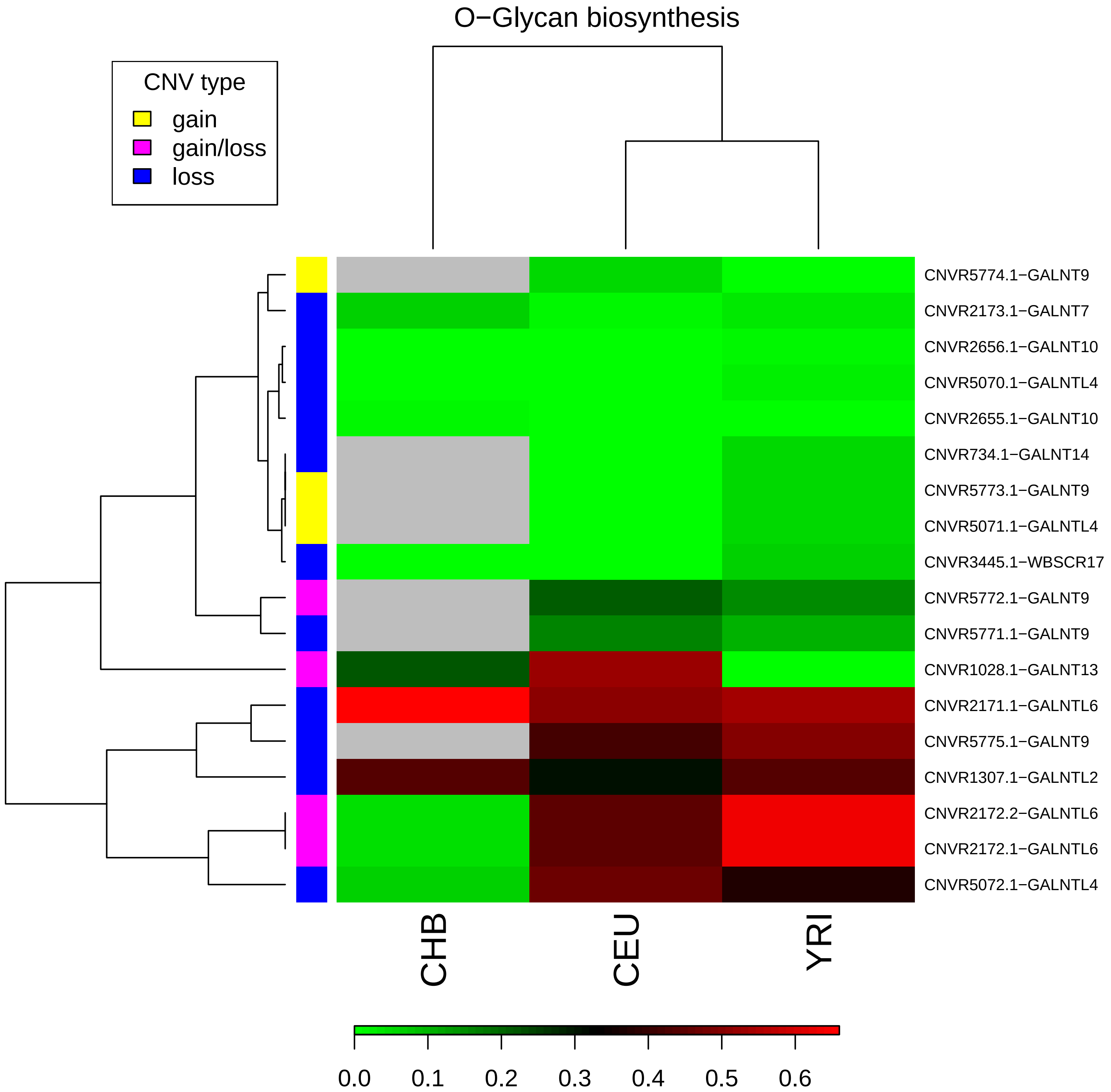

O−Glycan biosynthesis
CNV type
gain
gain/loss
loss
CNVR5774.1−GALNT9
CNVR2173.1−GALNT7
CNVR2656.1−GALNT10
CNVR5070.1−GALNTL4
CNVR2655.1−GALNT10
CNVR734.1−GALNT14
CNVR5773.1−GALNT9
CNVR5071.1−GALNTL4
CNVR3445.1−WBSCR17
CNVR5772.1−GALNT9
CNVR5771.1−GALNT9
CNVR1028.1−GALNT13
CNVR2171.1−GALNTL6
CNVR5775.1−GALNT9
CNVR1307.1−GALNTL2
CNVR2172.2−GALNTL6
CNVR2172.1−GALNTL6
CNVR5072.1−GALNTL4
CHB
CEU
YRI
0.0
0.1
0.2
0.3
0.4
0.5
0.6

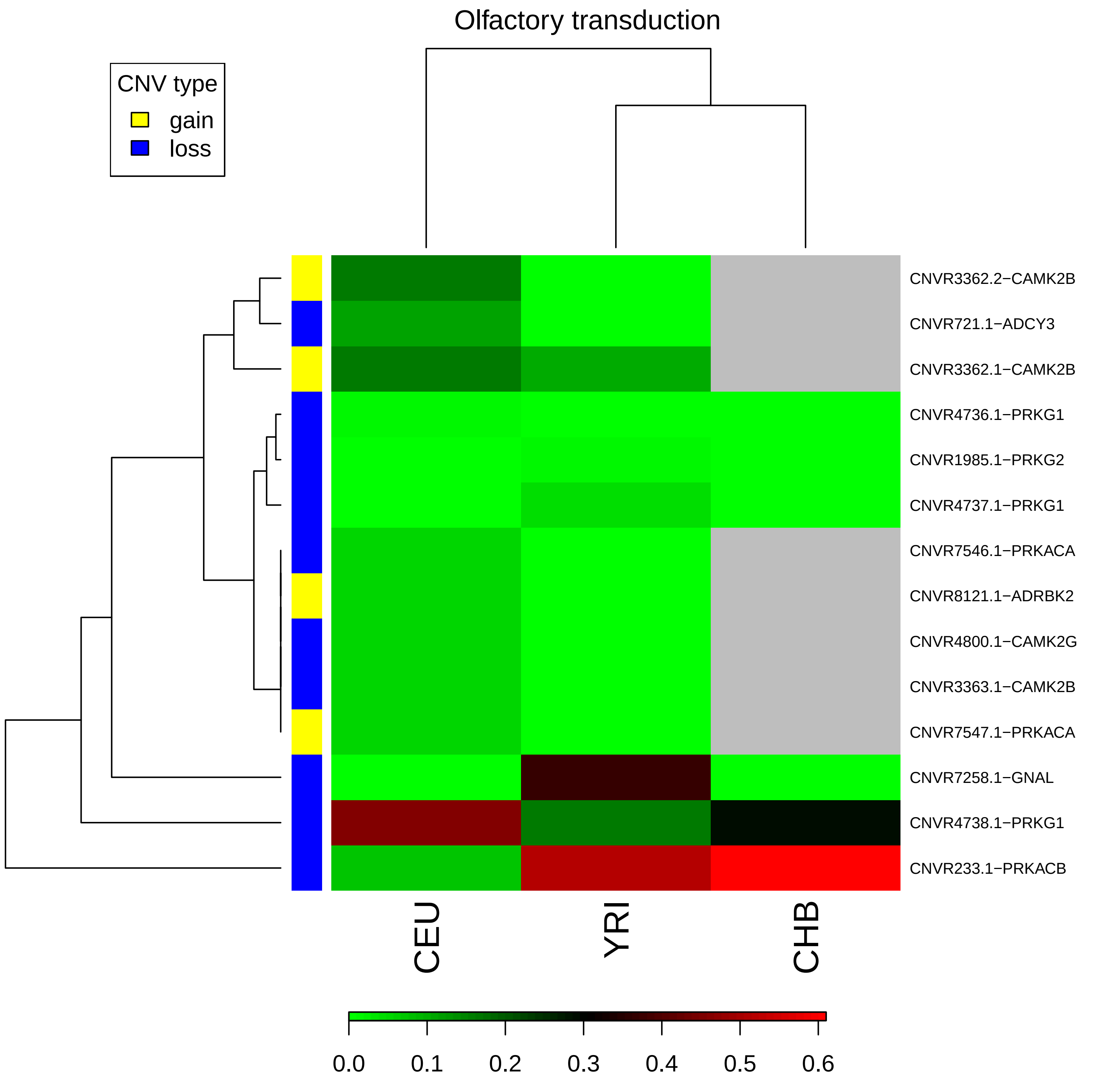
Olfactory transduction
CNV type
gain
loss
CNVR3362.2−CAMK2B
CNVR721.1−ADCY3
CNVR3362.1−CAMK2B
CNVR4736.1−PRKG1
CNVR1985.1−PRKG2
CNVR4737.1−PRKG1
CNVR7546.1−PRKACA
CNVR8121.1−ADRBK2
CNVR4800.1−CAMK2G
CNVR3363.1−CAMK2B
CNVR7547.1−PRKACA
CNVR7258.1−GNAL
CNVR4738.1−PRKG1
CNVR233.1−PRKACB
CEU
YRI
CHB
0.0
0.1
0.2
0.3
0.4
0.5
0.6

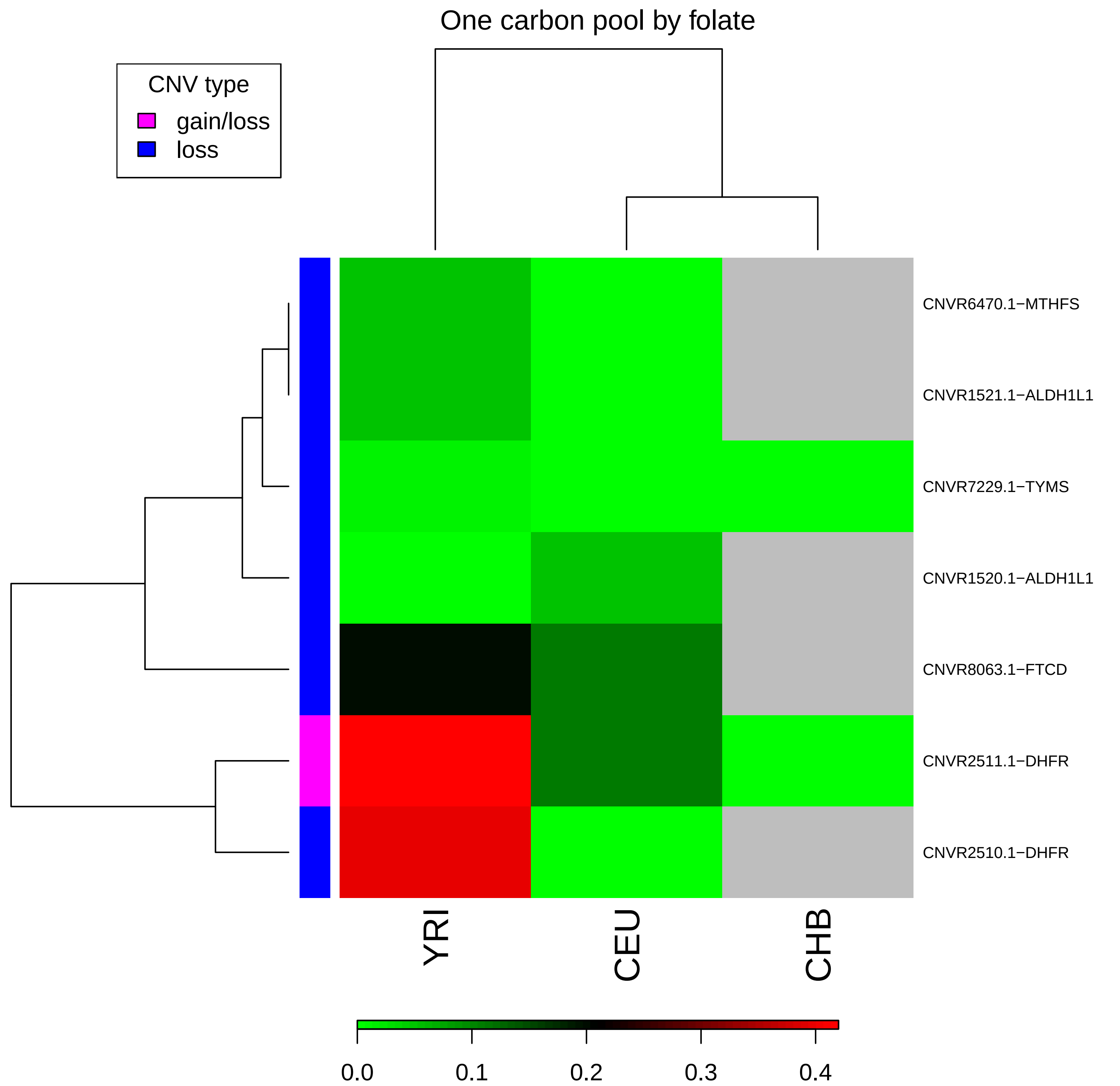

One carbon pool by folate
CNV type
gain/loss
loss
CNVR6470.1−MTHFS
CNVR1521.1−ALDH1L1
CNVR7229.1−TYMS
CNVR1520.1−ALDH1L1
CNVR8063.1−FTCD
CNVR2511.1−DHFR
CNVR2510.1−DHFR
YRI
CEU
CHB
0.0
0.1
0.2
0.3
0.4

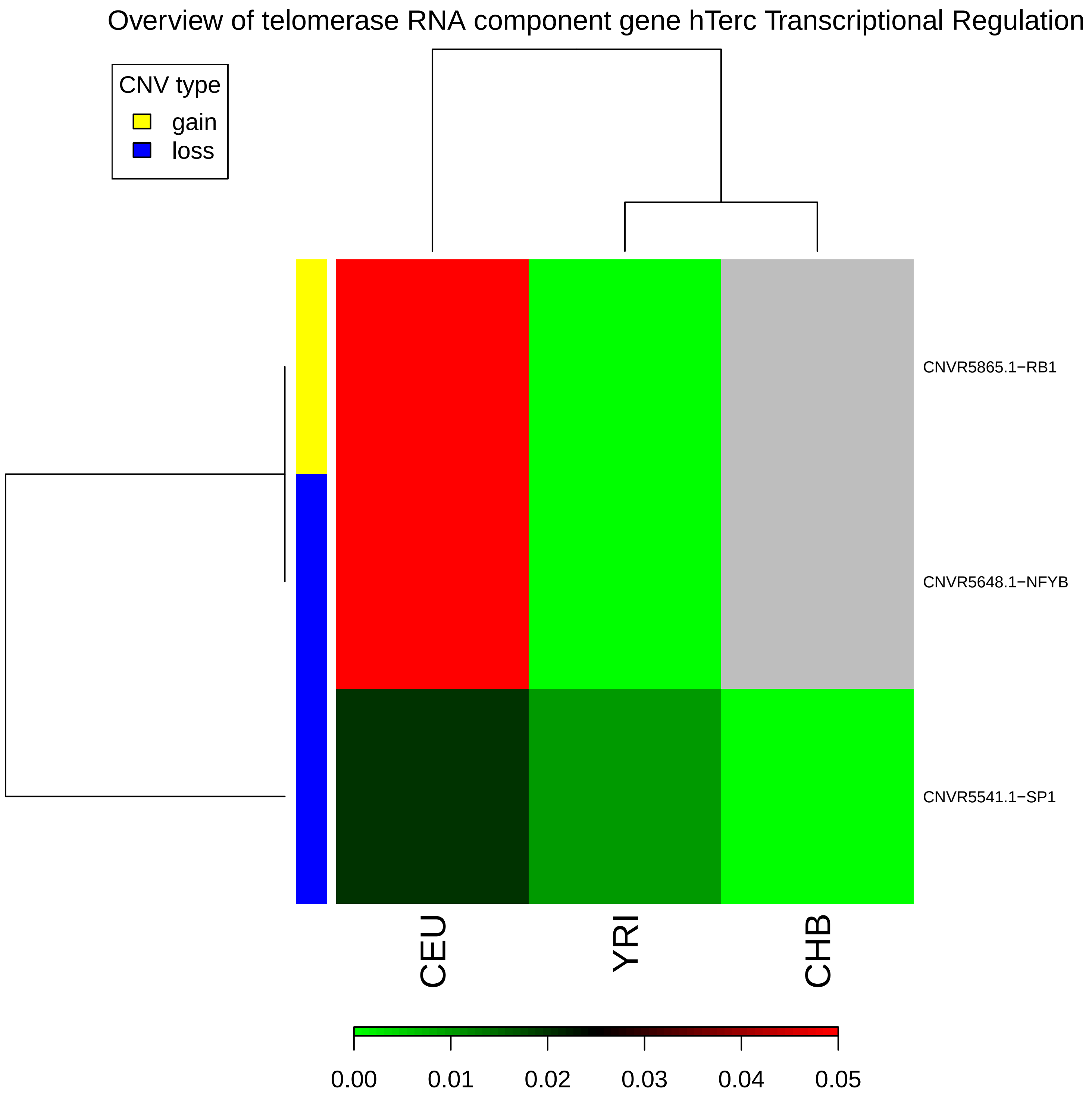
Overview of telomerase RNA component gene hTerc Transcriptional Regulation
CNV type
gain
loss
CNVR5865.1−RB1
CNVR5648.1−NFYB
CNVR5541.1−SP1
CEU
YRI
CHB
0.00
0.01
0.02
0.03
0.04
0.05

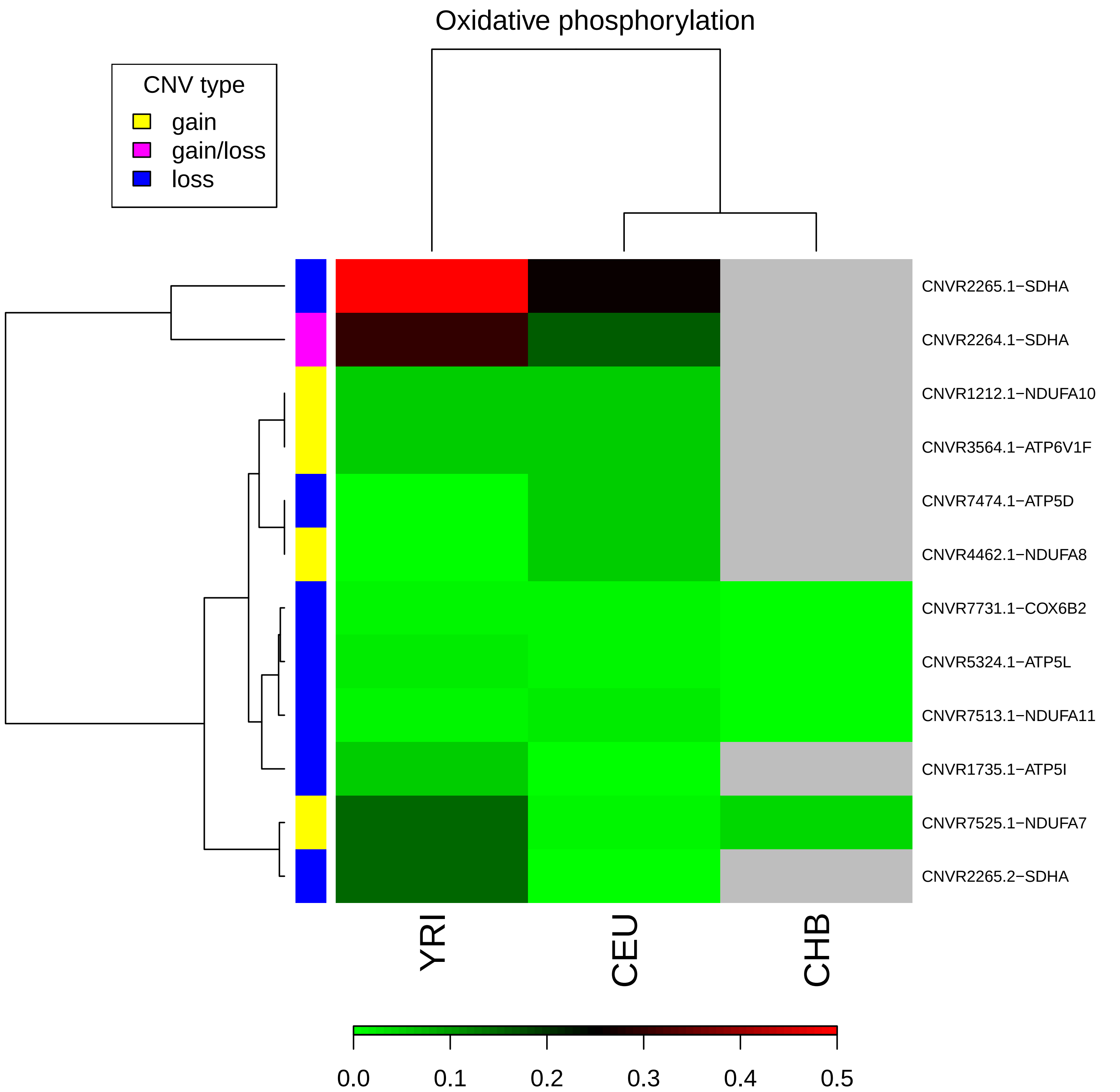

Oxidative phosphorylation
CNV type
gain
gain/loss
loss
CNVR2265.1−SDHA
CNVR2264.1−SDHA
CNVR1212.1−NDUFA10
CNVR3564.1−ATP6V1F
CNVR7474.1−ATP5D
CNVR4462.1−NDUFA8
CNVR7731.1−COX6B2
CNVR5324.1−ATP5L
CNVR7513.1−NDUFA11
CNVR1735.1−ATP5I
CNVR7525.1−NDUFA7
CNVR2265.2−SDHA
YRI
CEU
CHB
0.0
0.1
0.2
0.3
0.4
0.5

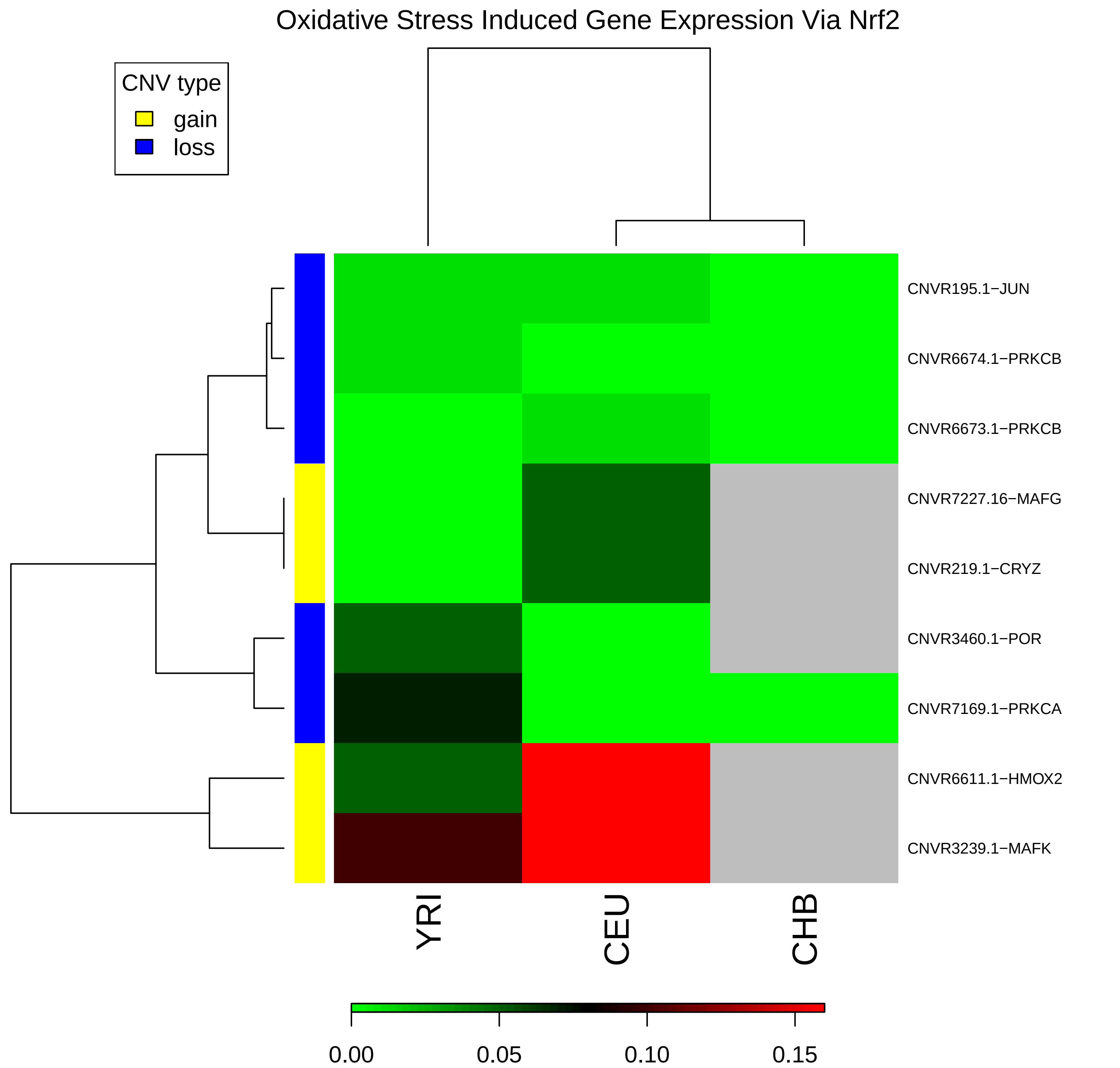

Oxidative Stress Induced Gene Expression Via Nrf2
CNV type
gain
loss
CNVR195.1−JUN
CNVR6674.1−PRKCB
CNVR6673.1−PRKCB
CNVR7227.16−MAFG
CNVR219.1−CRYZ
CNVR3460.1−POR
CNVR7169.1−PRKCA
CNVR6611.1−HMOX2
CNVR3239.1−MAFK
YRI
CEU
CHB
0.00
0.05
0.10
0.15

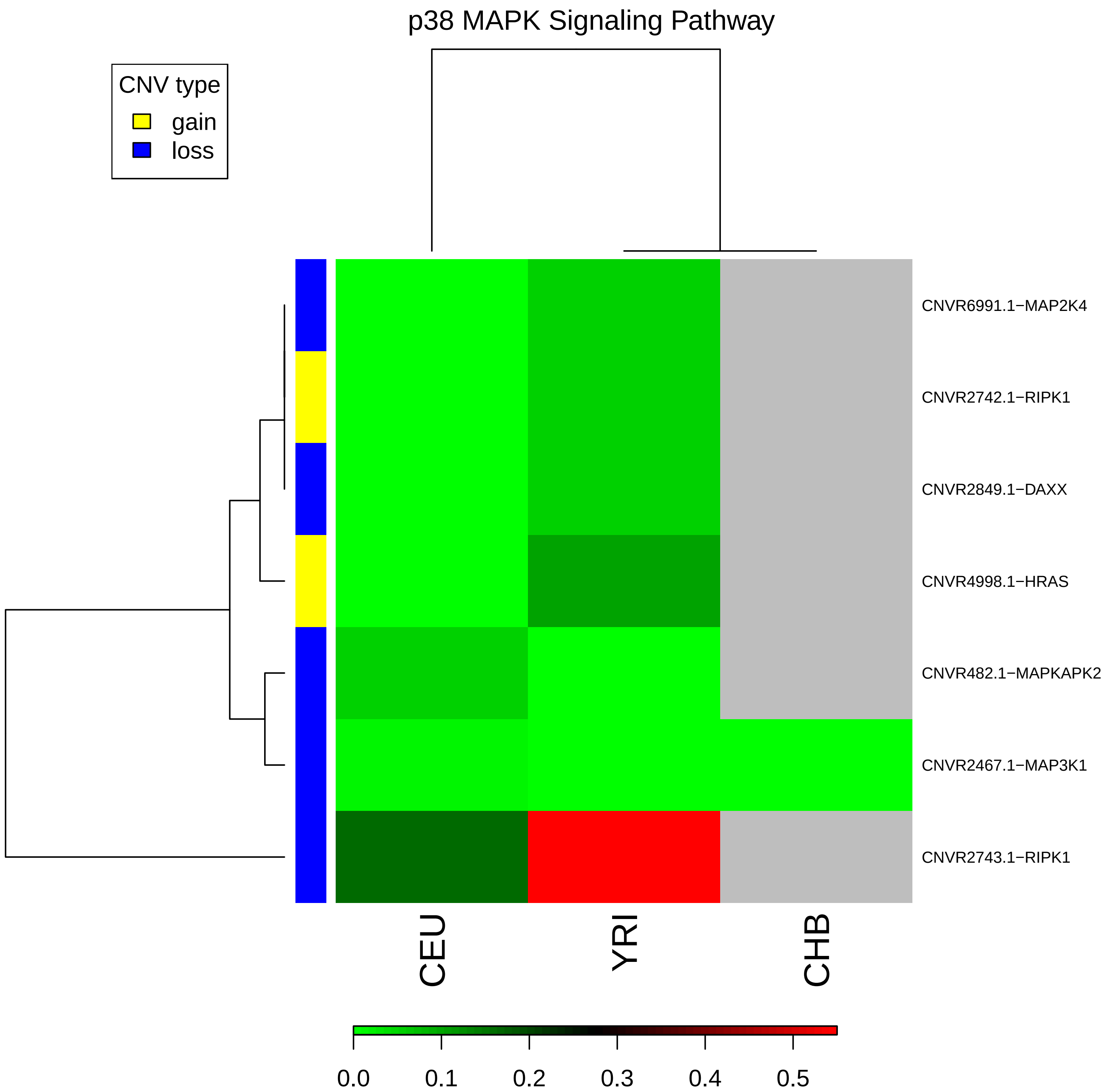
p38 MAPK Signaling Pathway
CNV type
gain
loss
CNVR6991.1−MAP2K4
CNVR2742.1−RIPK1
CNVR2849.1−DAXX
CNVR4998.1−HRAS
CNVR482.1−MAPKAPK2
CNVR2467.1−MAP3K1
CNVR2743.1−RIPK1
CEU
YRI
CHB
0.0
0.1
0.2
0.3
0.4
0.5

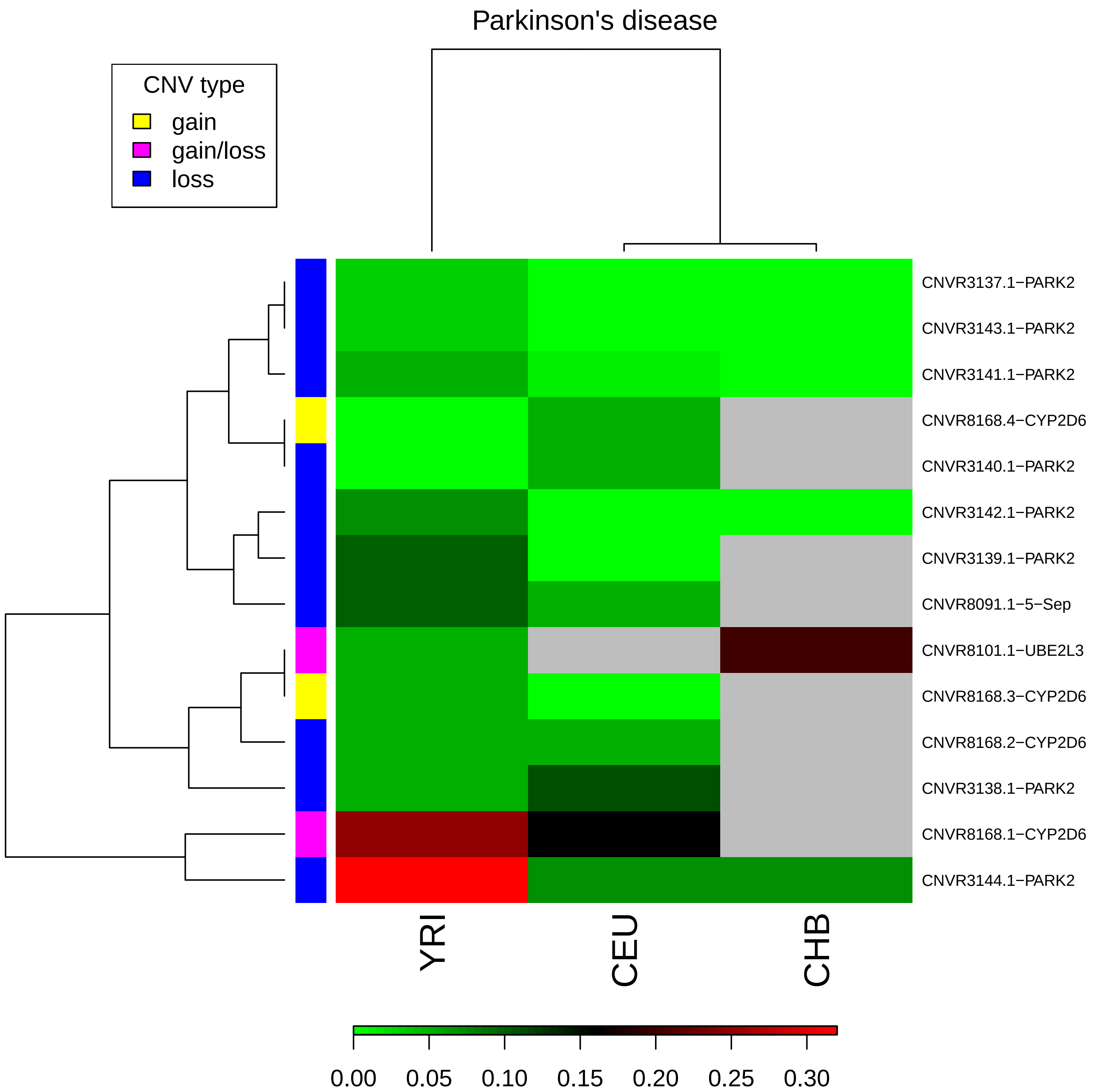
Parkinson's disease
CNV type
gain
gain/loss
loss
CNVR3137.1−PARK2
CNVR3143.1−PARK2
CNVR3141.1−PARK2
CNVR8168.4−CYP2D6
CNVR3140.1−PARK2
CNVR3142.1−PARK2
CNVR3139.1−PARK2
CNVR8091.1−5−Sep
CNVR8101.1−UBE2L3
CNVR8168.3−CYP2D6
CNVR8168.2−CYP2D6
CNVR3138.1−PARK2
CNVR8168.1−CYP2D6
CNVR3144.1−PARK2
YRI
CEU
CHB
0.00
0.05
0.10
0.15
0.20
0.25
0.30

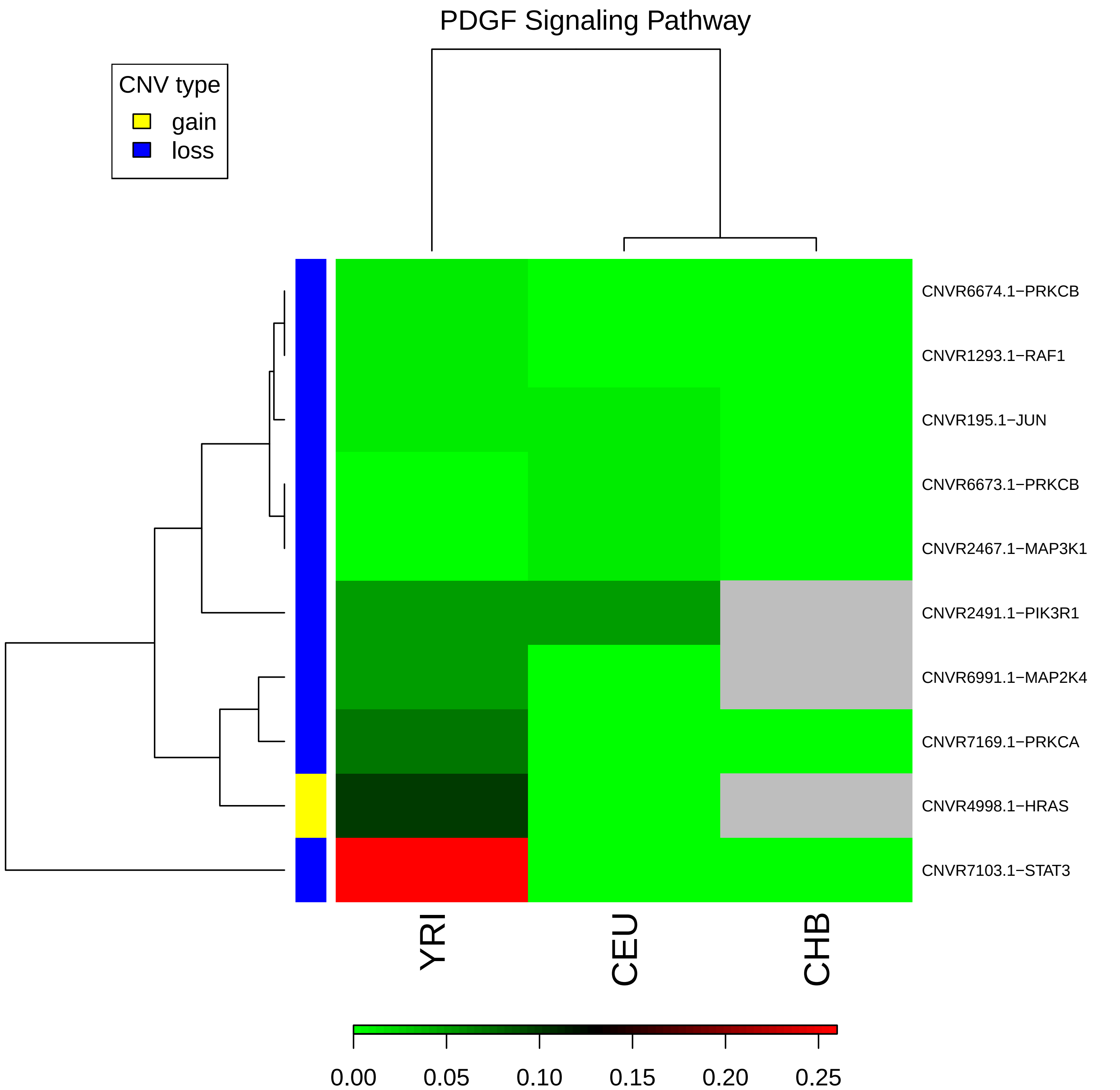
PDGF Signaling Pathway
CNV type
gain
loss
CNVR6674.1−PRKCB
CNVR1293.1−RAF1
CNVR195.1−JUN
CNVR6673.1−PRKCB
CNVR2467.1−MAP3K1
CNVR2491.1−PIK3R1
CNVR6991.1−MAP2K4
CNVR7169.1−PRKCA
CNVR4998.1−HRAS
CNVR7103.1−STAT3
YRI
CEU
CHB
0.00
0.05
0.10
0.15
0.20
0.25

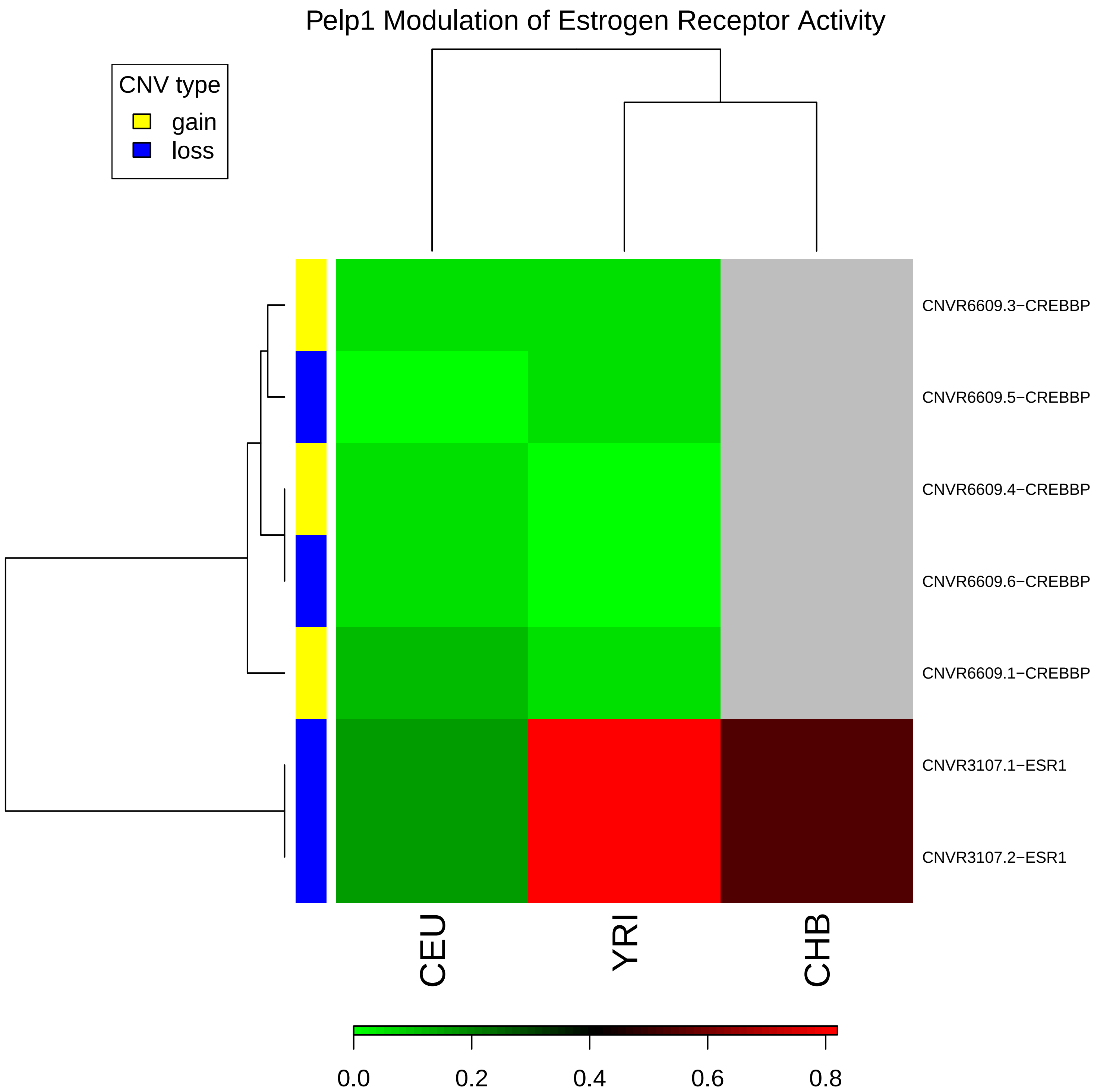
Pelp1 Modulation of Estrogen Receptor Activity
CNV type
gain
loss
CNVR6609.3−CREBBP
CNVR6609.5−CREBBP
CNVR6609.4−CREBBP
CNVR6609.6−CREBBP
CNVR6609.1−CREBBP
CNVR3107.1−ESR1
CNVR3107.2−ESR1
CEU
YRI
CHB
0.0
0.2
0.4
0.6
0.8

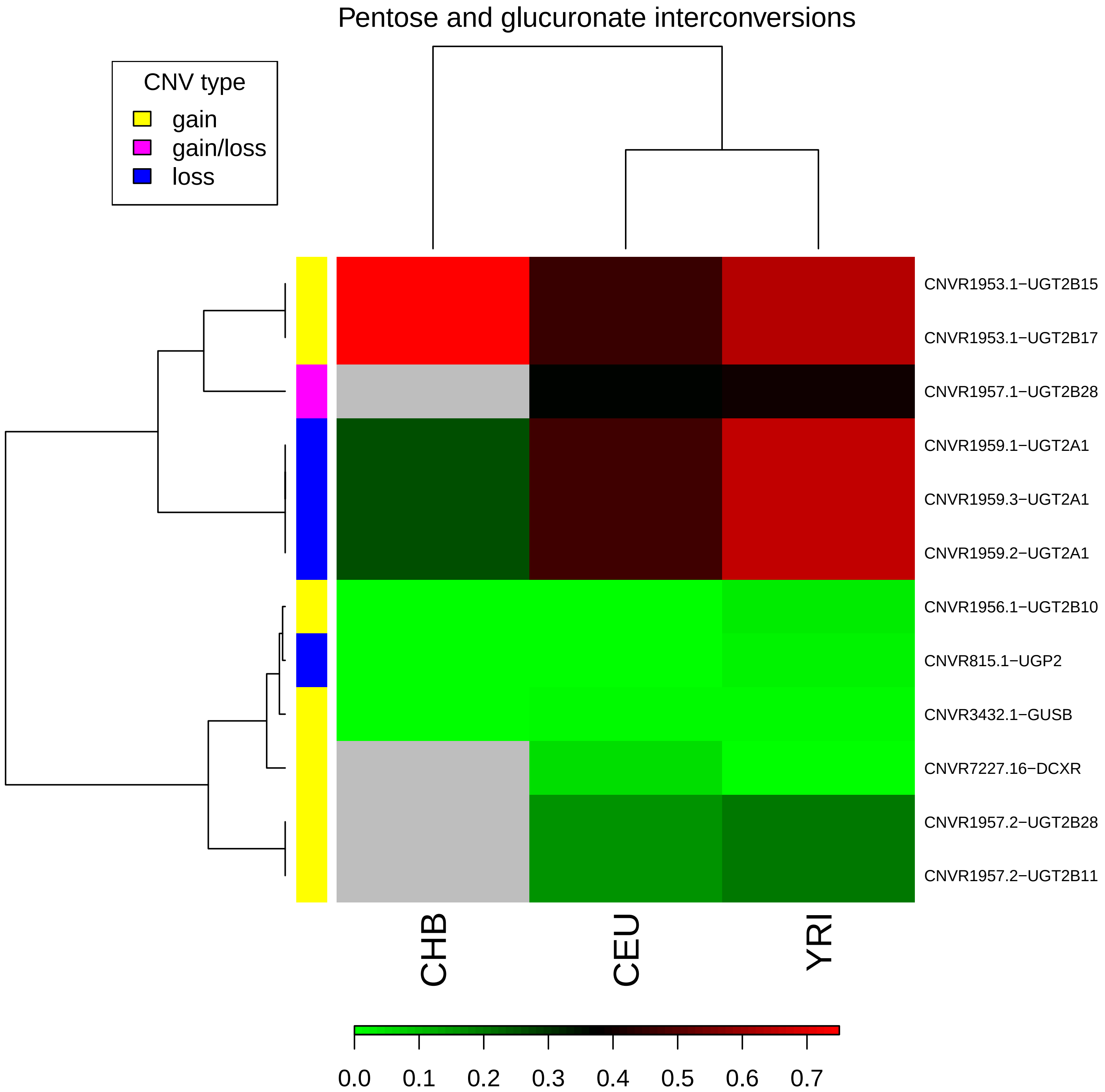

Pentose and glucuronate interconversions
CNV type
gain
gain/loss
loss
CNVR1953.1−UGT2B15
CNVR1953.1−UGT2B17
CNVR1957.1−UGT2B28
CNVR1959.1−UGT2A1
CNVR1959.3−UGT2A1
CNVR1959.2−UGT2A1
CNVR1956.1−UGT2B10
CNVR815.1−UGP2
CNVR3432.1−GUSB
CNVR7227.16−DCXR
CNVR1957.2−UGT2B28
CNVR1957.2−UGT2B11
CHB
CEU
YRI
0.0
0.1
0.2
0.3
0.4
0.5
0.6
0.7

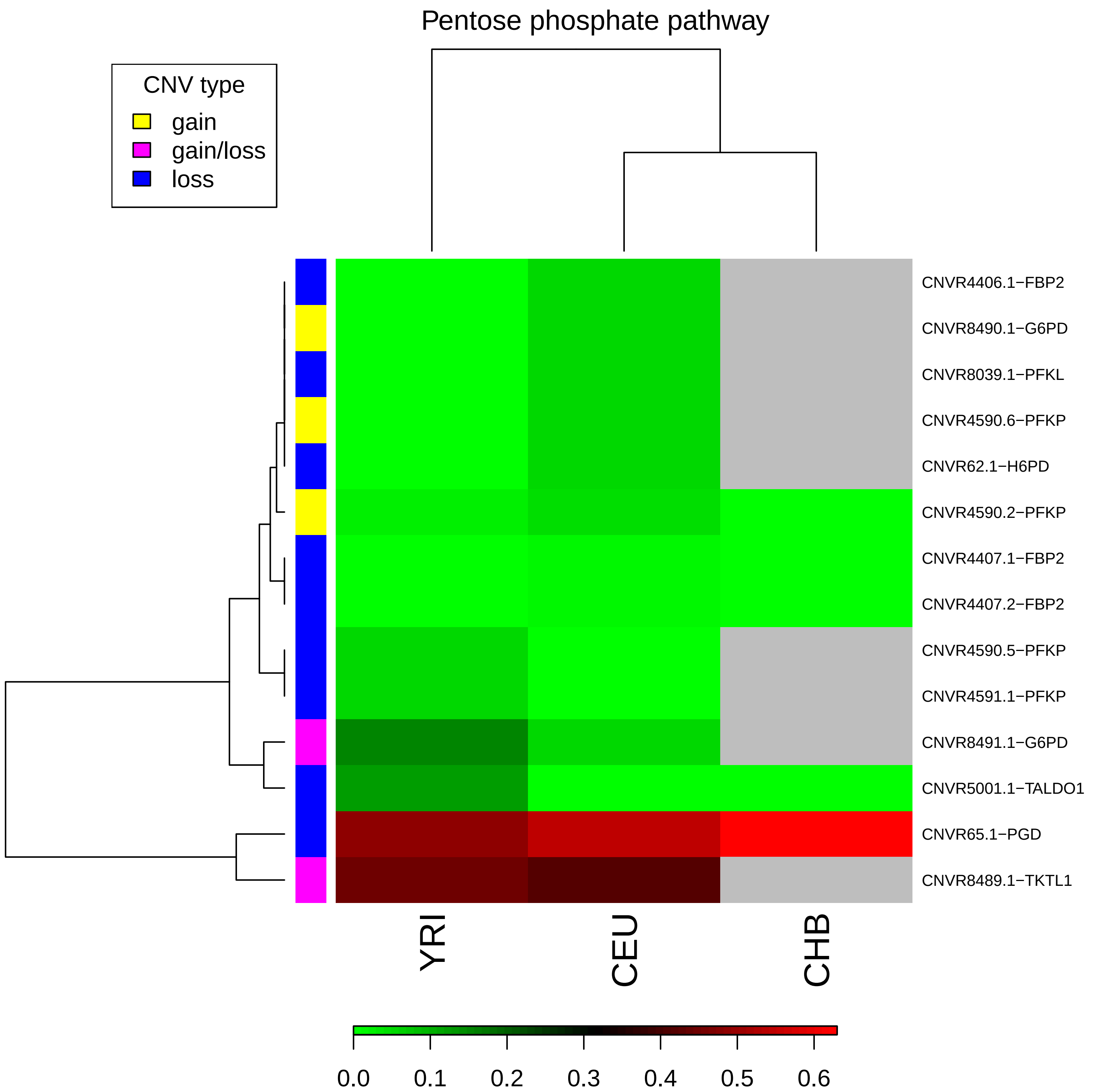
Pentose phosphate pathway
CNV type
gain
gain/loss
loss
CNVR4406.1−FBP2
CNVR8490.1−G6PD
CNVR8039.1−PFKL
CNVR4590.6−PFKP
CNVR62.1−H6PD
CNVR4590.2−PFKP
CNVR4407.1−FBP2
CNVR4407.2−FBP2
CNVR4590.5−PFKP
CNVR4591.1−PFKP
CNVR8491.1−G6PD
CNVR5001.1−TALDO1
CNVR65.1−PGD
CNVR8489.1−TKTL1
YRI
CEU
CHB
0.0
0.1
0.2
0.3
0.4
0.5
0.6

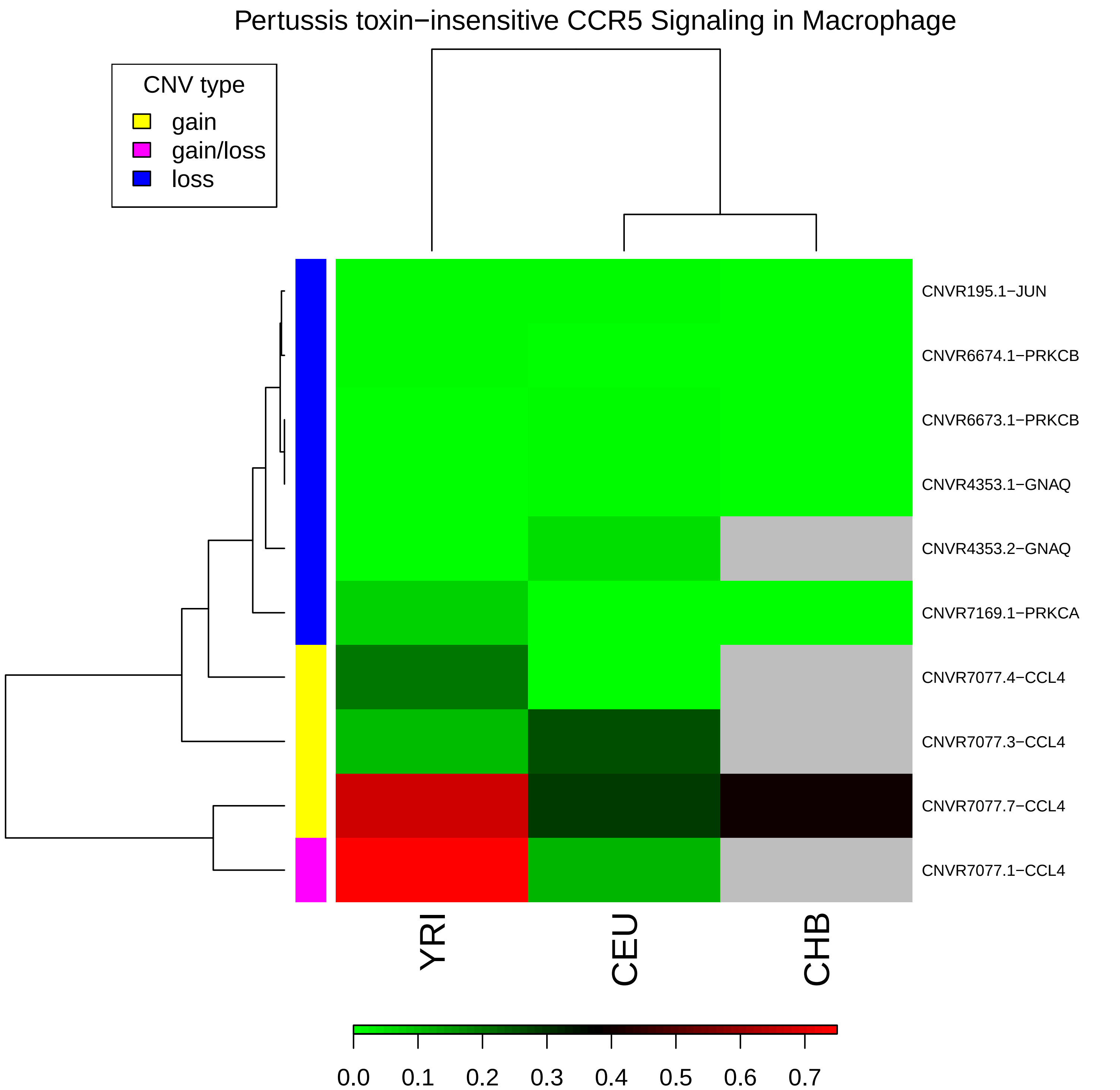
Pertussis toxin−insensitive CCR5 Signaling in Macrophage
CNV type
gain
gain/loss
loss
CNVR195.1−JUN
CNVR6674.1−PRKCB
CNVR6673.1−PRKCB
CNVR4353.1−GNAQ
CNVR4353.2−GNAQ
CNVR7169.1−PRKCA
CNVR7077.4−CCL4
CNVR7077.3−CCL4
CNVR7077.7−CCL4
CNVR7077.1−CCL4
YRI
CEU
CHB
0.0
0.1
0.2
0.3
0.4
0.5
0.6
0.7

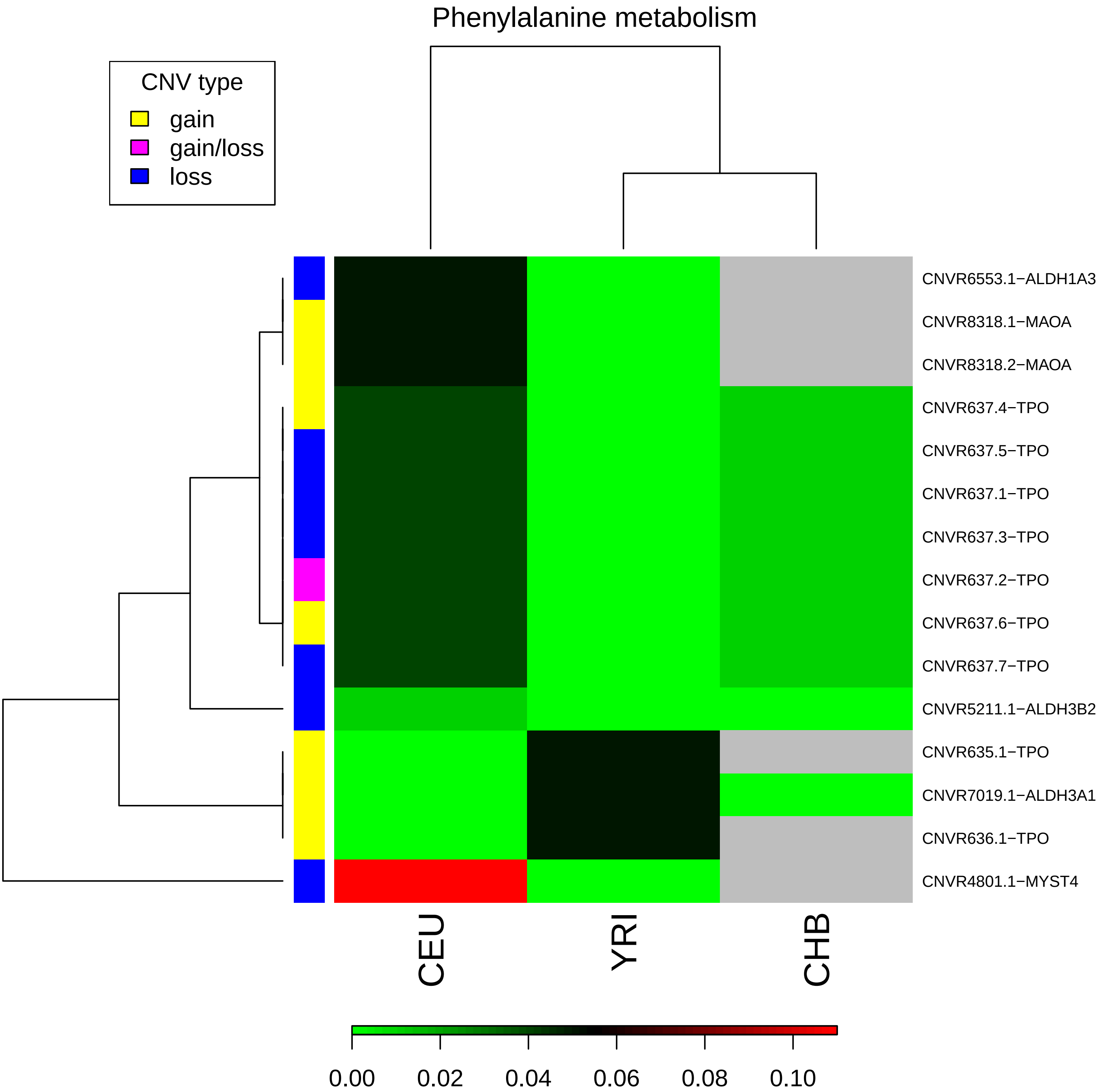

Phenylalanine metabolism
CNV type
gain
gain/loss
loss
CNVR6553.1−ALDH1A3
CNVR8318.1−MAOA
CNVR8318.2−MAOA
CNVR637.4−TPO
CNVR637.5−TPO
CNVR637.1−TPO
CNVR637.3−TPO
CNVR637.2−TPO
CNVR637.6−TPO
CNVR637.7−TPO
CNVR5211.1−ALDH3B2
CNVR635.1−TPO
CNVR7019.1−ALDH3A1
CNVR636.1−TPO
CNVR4801.1−MYST4
CEU
YRI
CHB
0.00
0.02
0.04
0.06
0.08
0.10

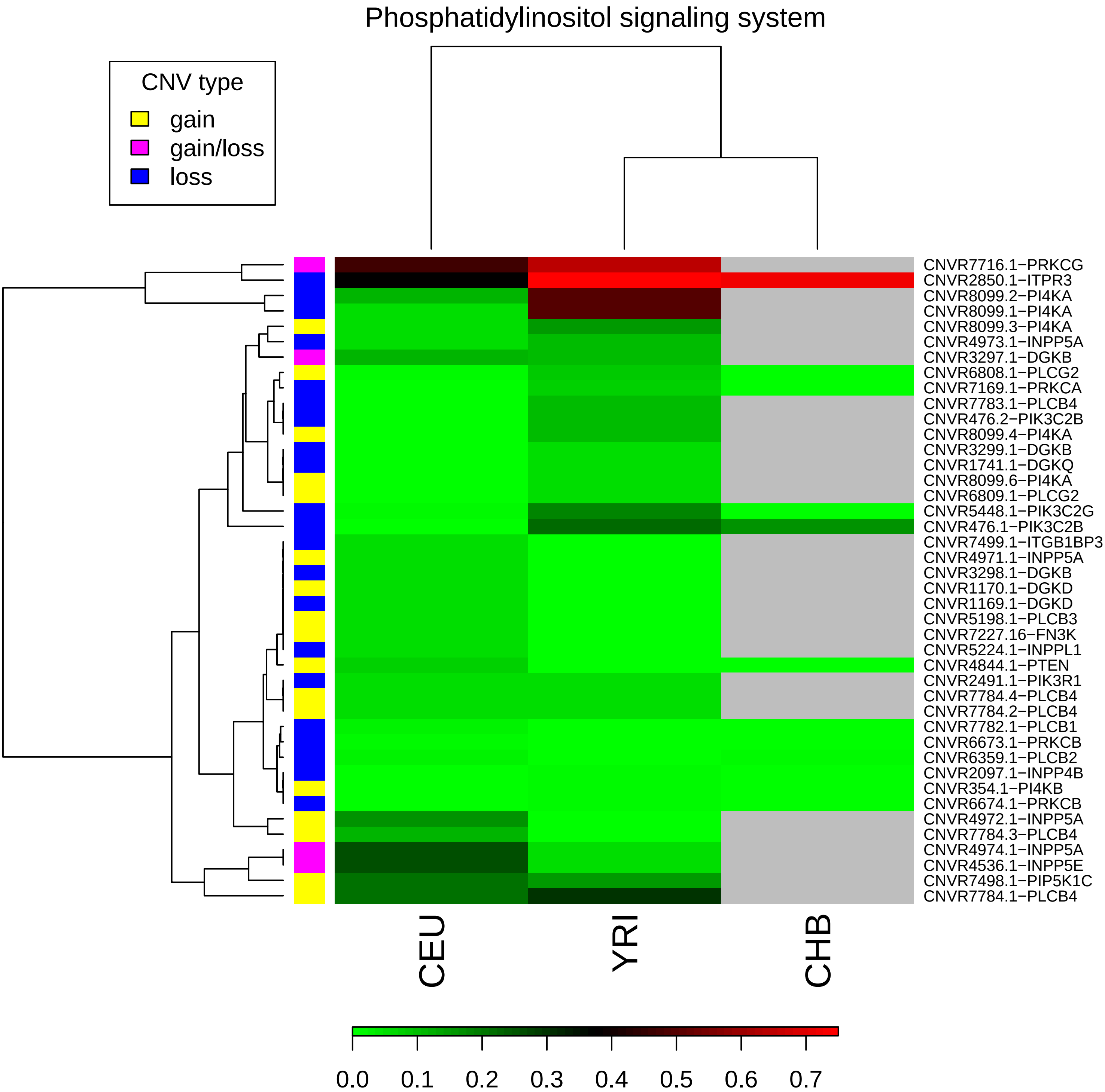
Phosphatidylinositol signaling system
CNV type
gain
gain/loss
loss
CNVR7716.1−PRKCG
CNVR2850.1−ITPR3
CNVR8099.2−PI4KA
CNVR8099.1−PI4KA
CNVR8099.3−PI4KA
CNVR4973.1−INPP5A
CNVR3297.1−DGKB
CNVR6808.1−PLCG2
CNVR7169.1−PRKCA
CNVR7783.1−PLCB4
CNVR476.2−PIK3C2B
CNVR8099.4−PI4KA
CNVR3299.1−DGKB
CNVR1741.1−DGKQ
CNVR8099.6−PI4KA
CNVR6809.1−PLCG2
CNVR5448.1−PIK3C2G
CNVR476.1−PIK3C2B
CNVR7499.1−ITGB1BP3
CNVR4971.1−INPP5A
CNVR3298.1−DGKB
CNVR1170.1−DGKD
CNVR1169.1−DGKD
CNVR5198.1−PLCB3
CNVR7227.16−FN3K
CNVR5224.1−INPPL1
CNVR4844.1−PTEN
CNVR2491.1−PIK3R1
CNVR7784.4−PLCB4
CNVR7784.2−PLCB4
CNVR7782.1−PLCB1
CNVR6673.1−PRKCB
CNVR6359.1−PLCB2
CNVR2097.1−INPP4B
CNVR354.1−PI4KB
CNVR6674.1−PRKCB
CNVR4972.1−INPP5A
CNVR7784.3−PLCB4
CNVR4974.1−INPP5A
CNVR4536.1−INPP5E
CNVR7498.1−PIP5K1C
CNVR7784.1−PLCB4
CEU
YRI
CHB
0.0
0.1
0.2
0.3
0.4
0.5
0.6
0.7

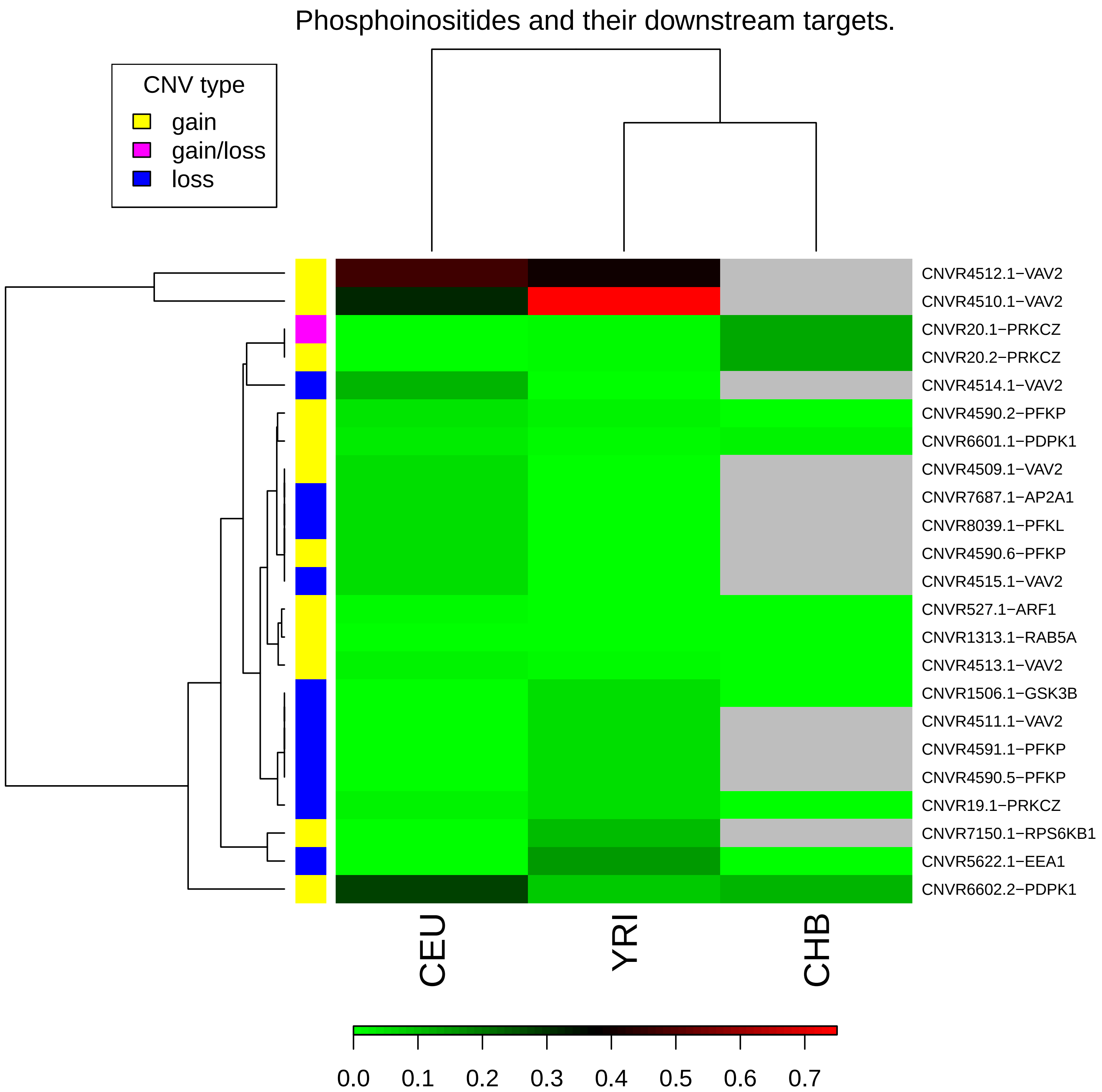
Phosphoinositides and their downstream targets.
CNV type
gain
gain/loss
loss
CNVR4512.1−VAV2
CNVR4510.1−VAV2
CNVR20.1−PRKCZ
CNVR20.2−PRKCZ
CNVR4514.1−VAV2
CNVR4590.2−PFKP
CNVR6601.1−PDPK1
CNVR4509.1−VAV2
CNVR7687.1−AP2A1
CNVR8039.1−PFKL
CNVR4590.6−PFKP
CNVR4515.1−VAV2
CNVR527.1−ARF1
CNVR1313.1−RAB5A
CNVR4513.1−VAV2
CNVR1506.1−GSK3B
CNVR4511.1−VAV2
CNVR4591.1−PFKP
CNVR4590.5−PFKP
CNVR19.1−PRKCZ
CNVR7150.1−RPS6KB1
CNVR5622.1−EEA1
CNVR6602.2−PDPK1
CEU
YRI
CHB
0.0
0.1
0.2
0.3
0.4
0.5
0.6
0.7

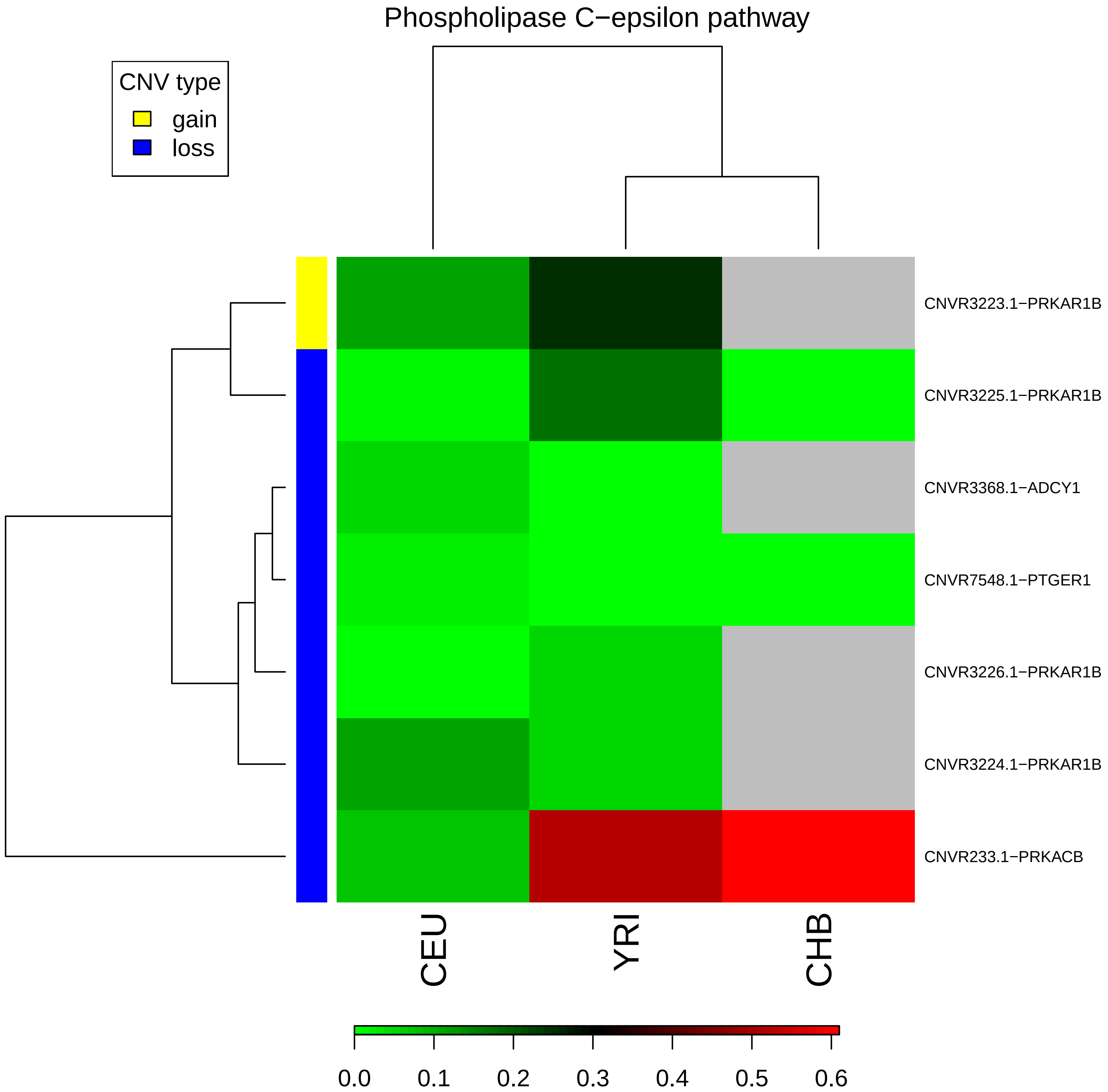
Phospholipase C−epsilon pathway
CNV type
gain
loss
CNVR3223.1−PRKAR1B
CNVR3225.1−PRKAR1B
CNVR3368.1−ADCY1
CNVR7548.1−PTGER1
CNVR3226.1−PRKAR1B
CNVR3224.1−PRKAR1B
CNVR233.1−PRKACB
CEU
YRI
CHB
0.0
0.1
0.2
0.3
0.4
0.5
0.6

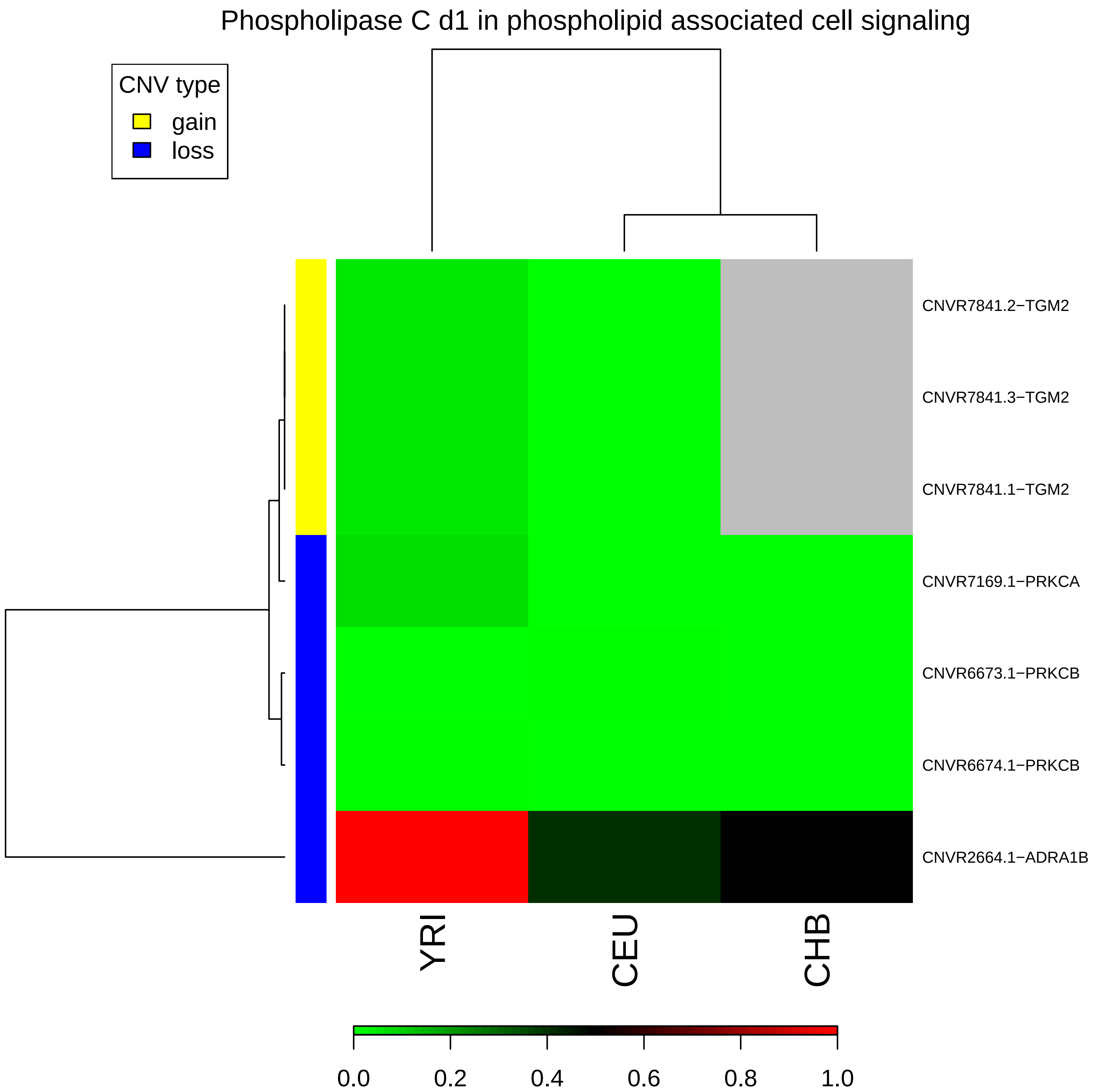
Phospholipase C d1 in phospholipid associated cell signaling
CNV type
gain
loss
CNVR7841.2−TGM2
CNVR7841.3−TGM2
CNVR7841.1−TGM2
CNVR7169.1−PRKCA
CNVR6673.1−PRKCB
CNVR6674.1−PRKCB
CNVR2664.1−ADRA1B
YRI
CEU
CHB
0.0
0.2
0.4
0.6
0.8
1.0

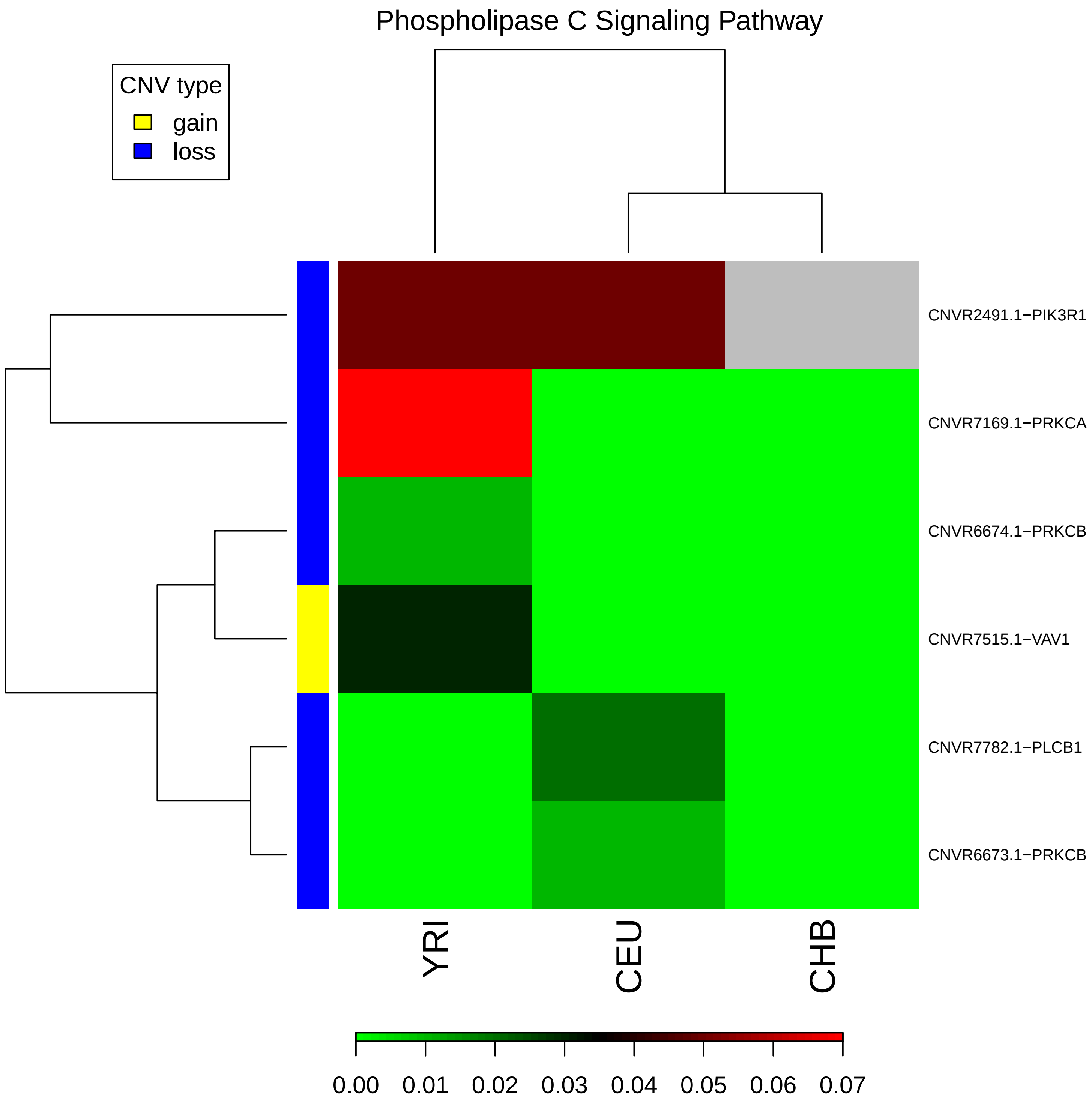

Phospholipase C Signaling Pathway
CNV type
gain
loss
CNVR2491.1−PIK3R1
CNVR7169.1−PRKCA
CNVR6674.1−PRKCB
CNVR7515.1−VAV1
CNVR7782.1−PLCB1
CNVR6673.1−PRKCB
YRI
CEU
CHB
0.00
0.01
0.02
0.03
0.04
0.05
0.06
0.07

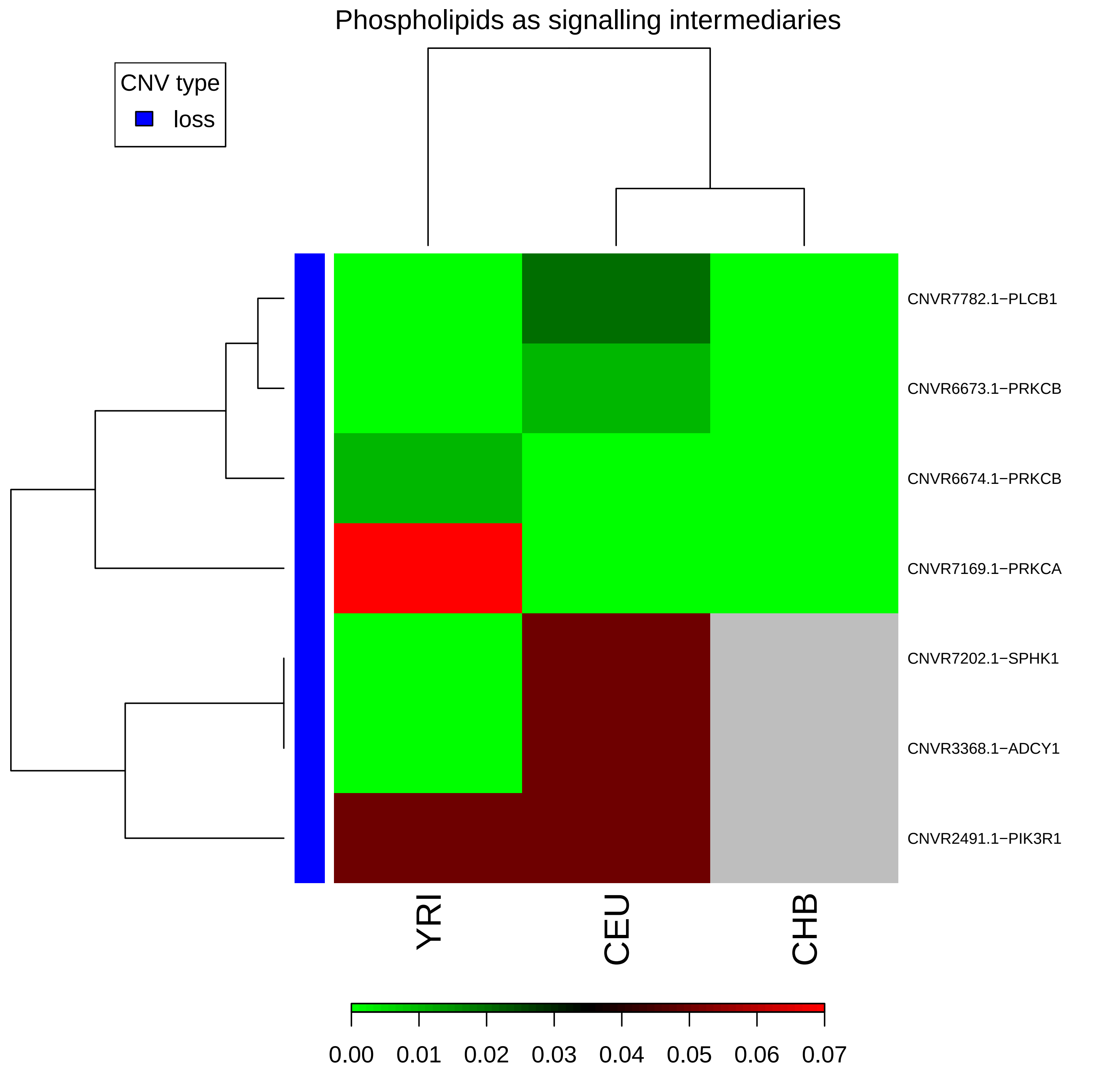

Phospholipids as signalling intermediaries
CNV type
loss
CNVR7782.1−PLCB1
CNVR6673.1−PRKCB
CNVR6674.1−PRKCB
CNVR7169.1−PRKCA
CNVR7202.1−SPHK1
CNVR3368.1−ADCY1
CNVR2491.1−PIK3R1
YRI
CEU
CHB
0.00
0.01
0.02
0.03
0.04
0.05
0.06
0.07

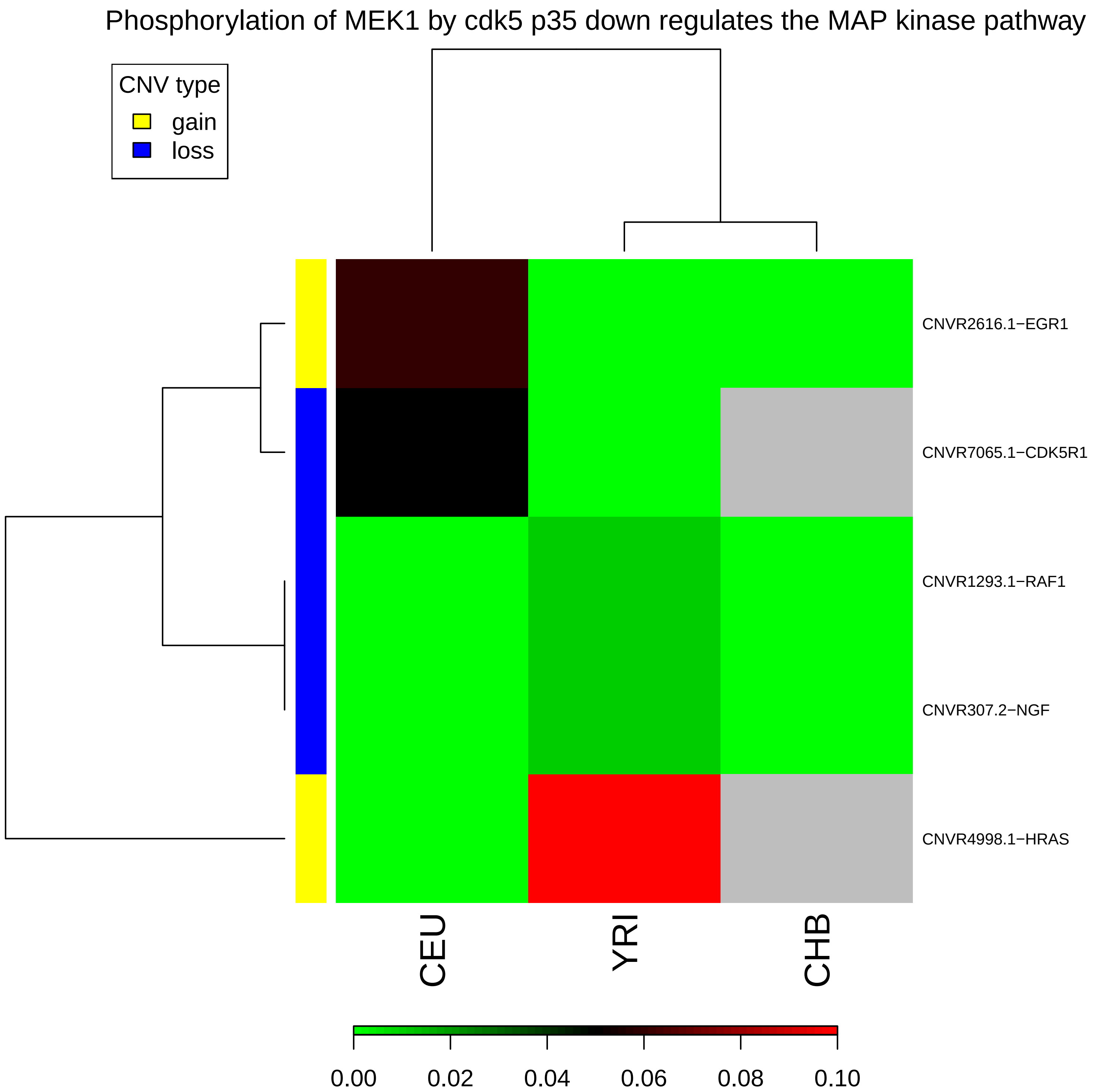

Phosphorylation of MEK1 by cdk5 p35 down regulates the MAP kinase pathway
CNV type
gain
loss
CNVR2616.1−EGR1
CNVR7065.1−CDK5R1
CNVR1293.1−RAF1
CNVR307.2−NGF
CNVR4998.1−HRAS
CEU
YRI
CHB
0.00
0.02
0.04
0.06
0.08
0.10

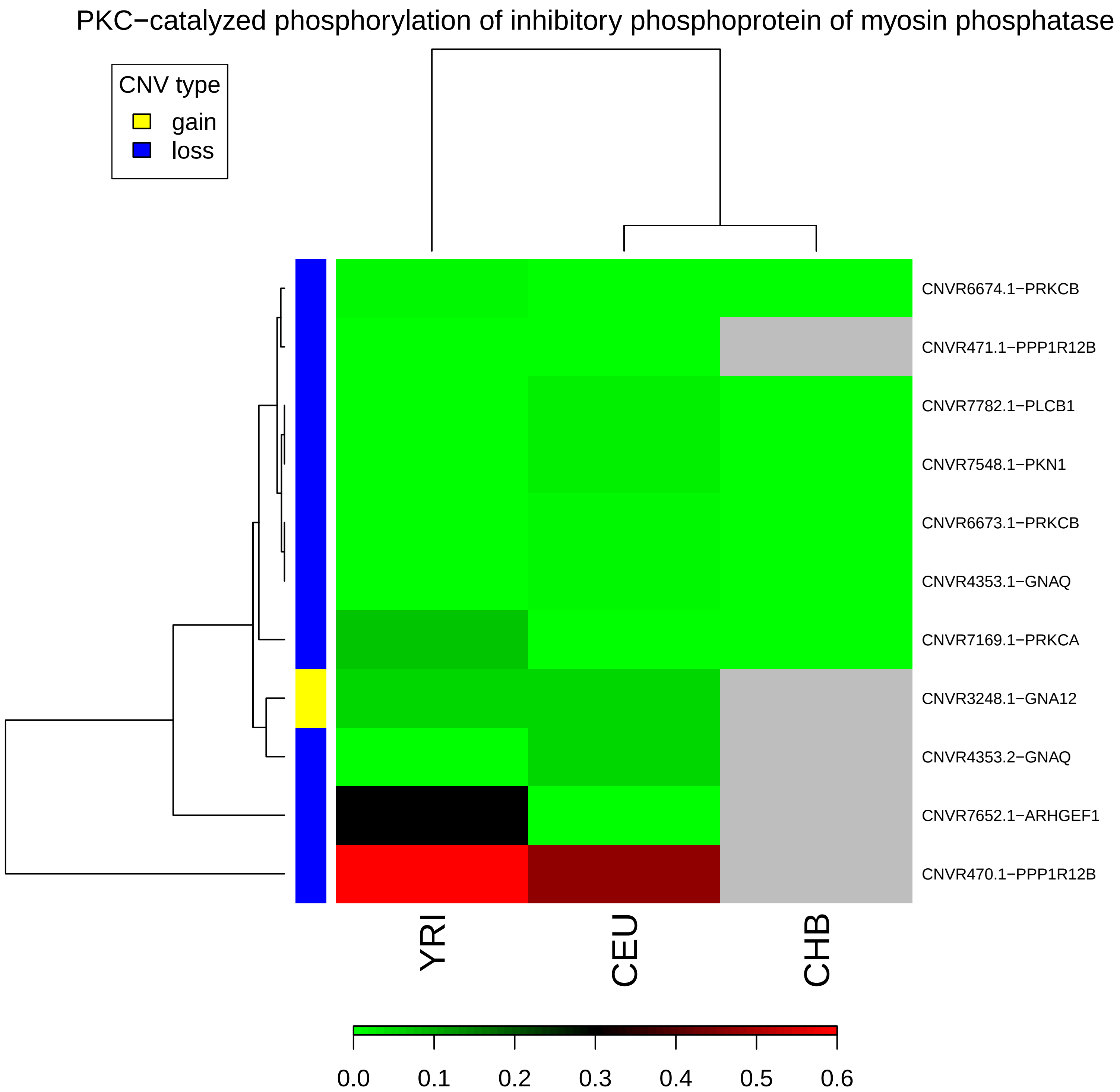

PKC−catalyzed phosphorylation of inhibitory phosphoprotein of myosin phosphatase
CNV type
gain
loss
CNVR6674.1−PRKCB
CNVR471.1−PPP1R12B
CNVR7782.1−PLCB1
CNVR7548.1−PKN1
CNVR6673.1−PRKCB
CNVR4353.1−GNAQ
CNVR7169.1−PRKCA
CNVR3248.1−GNA12
CNVR4353.2−GNAQ
CNVR7652.1−ARHGEF1
CNVR470.1−PPP1R12B
YRI
CEU
CHB
0.0
0.1
0.2
0.3
0.4
0.5
0.6

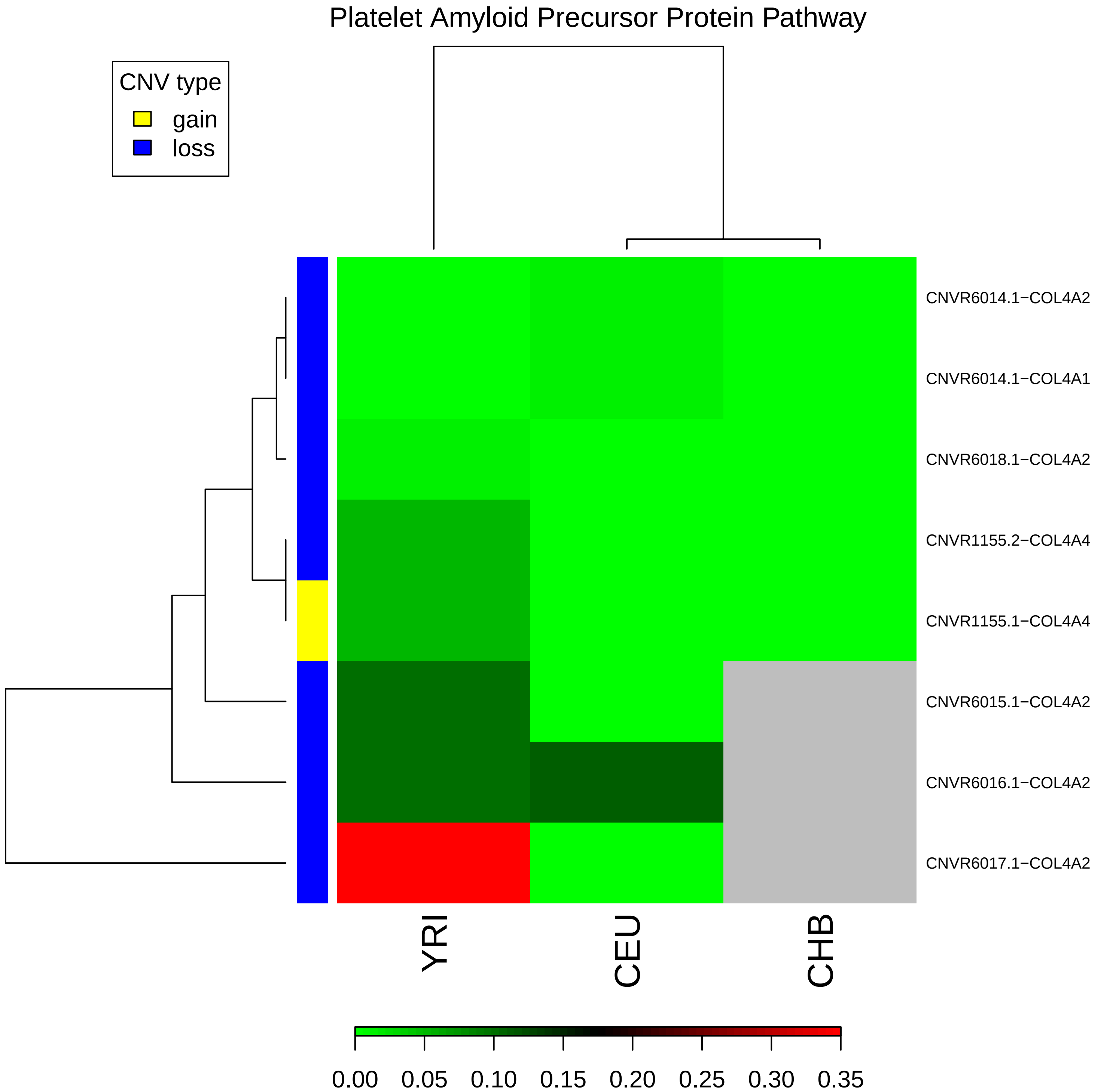

Platelet Amyloid Precursor Protein Pathway
CNV type
gain
loss
CNVR6014.1−COL4A2
CNVR6014.1−COL4A1
CNVR6018.1−COL4A2
CNVR1155.2−COL4A4
CNVR1155.1−COL4A4
CNVR6015.1−COL4A2
CNVR6016.1−COL4A2
CNVR6017.1−COL4A2
YRI
CEU
CHB
0.00
0.05
0.10
0.15
0.20
0.25
0.30
0.35

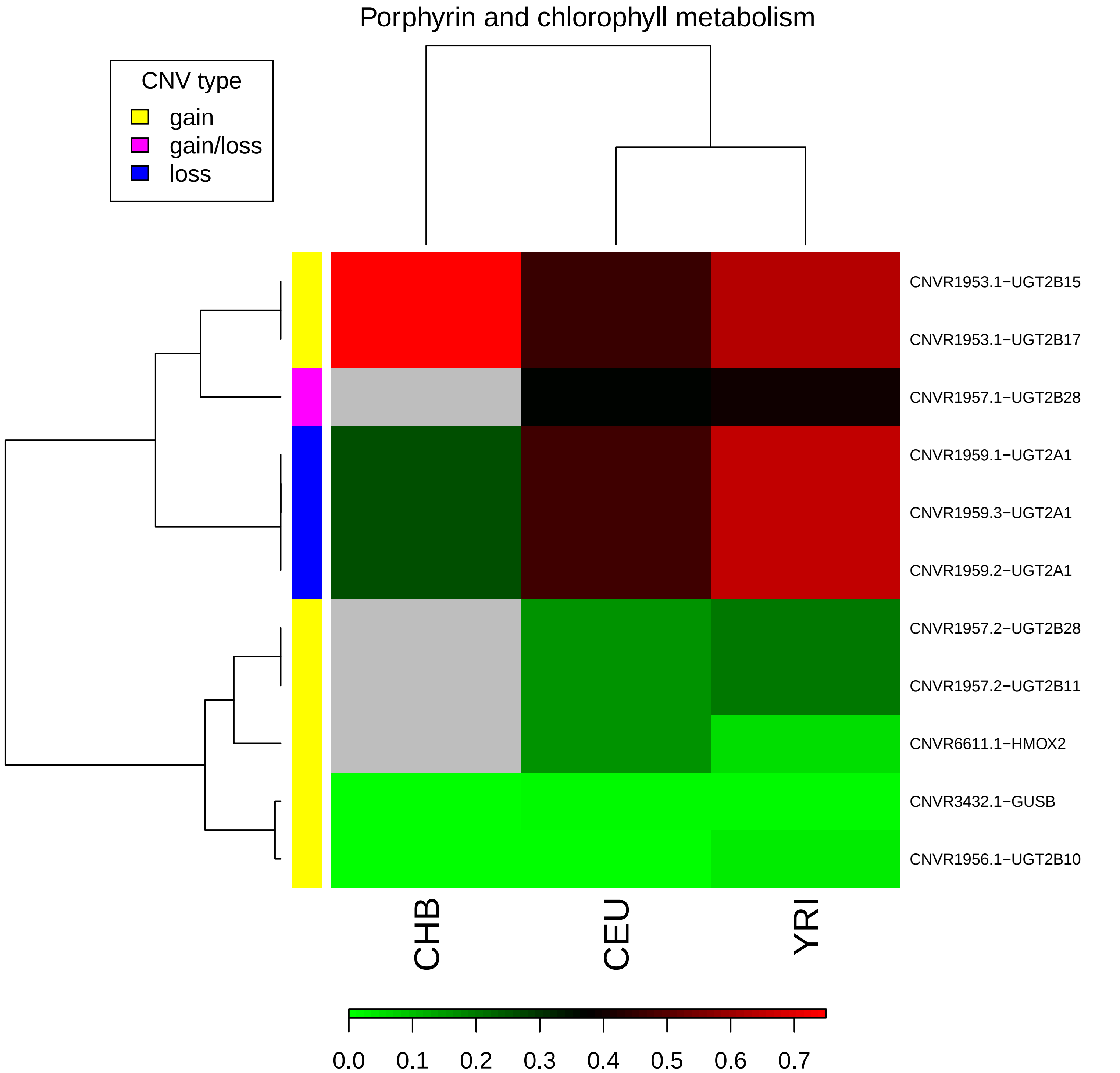

Porphyrin and chlorophyll metabolism
CNV type
gain
gain/loss
loss
CNVR1953.1−UGT2B15
CNVR1953.1−UGT2B17
CNVR1957.1−UGT2B28
CNVR1959.1−UGT2A1
CNVR1959.3−UGT2A1
CNVR1959.2−UGT2A1
CNVR1957.2−UGT2B28
CNVR1957.2−UGT2B11
CNVR6611.1−HMOX2
CNVR3432.1−GUSB
CNVR1956.1−UGT2B10
CHB
CEU
YRI
0.0
0.1
0.2
0.3
0.4
0.5
0.6
0.7

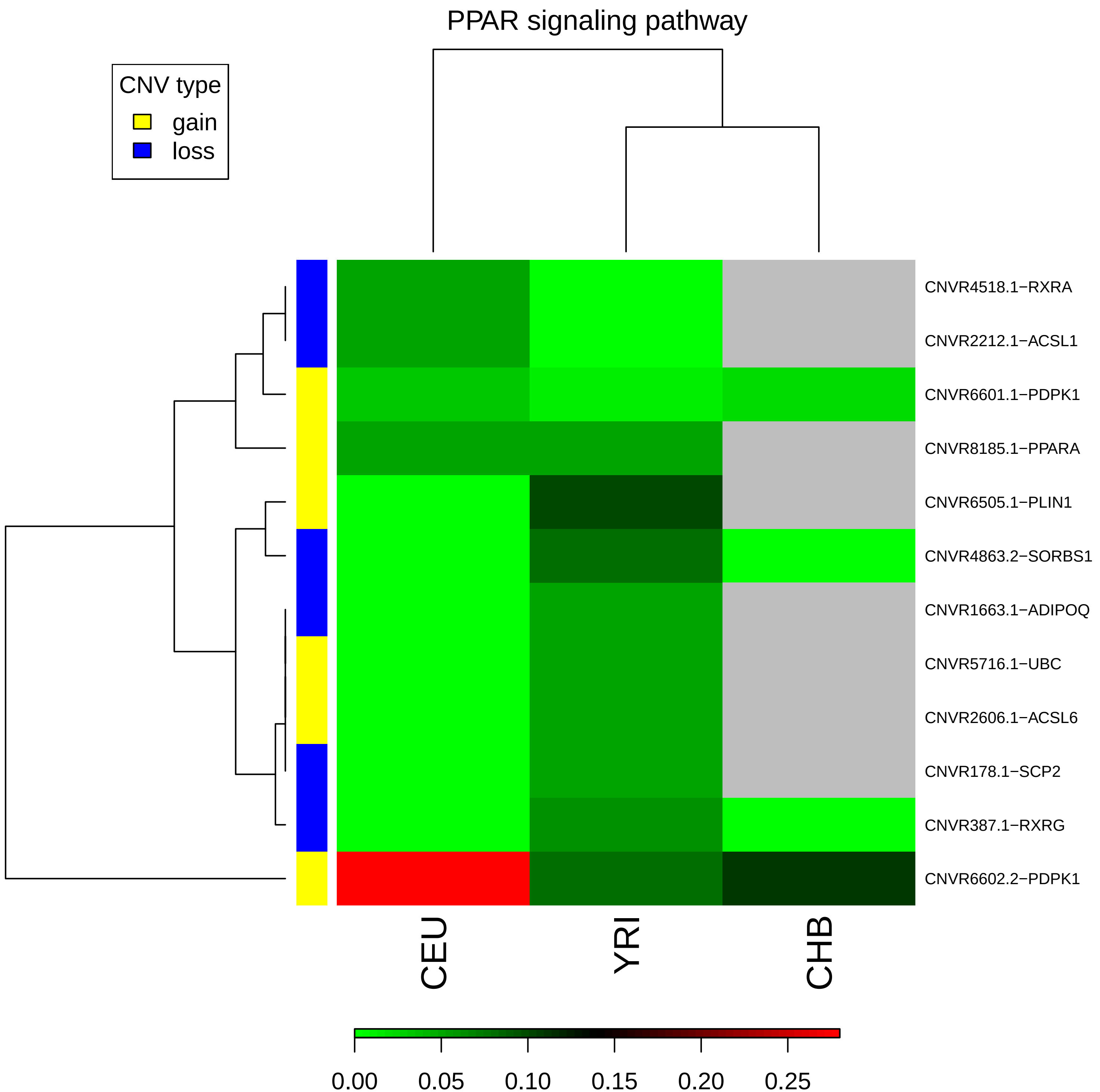
PPAR signaling pathway
CNV type
gain
loss
CNVR4518.1−RXRA
CNVR2212.1−ACSL1
CNVR6601.1−PDPK1
CNVR8185.1−PPARA
CNVR6505.1−PLIN1
CNVR4863.2−SORBS1
CNVR1663.1−ADIPOQ
CNVR5716.1−UBC
CNVR2606.1−ACSL6
CNVR178.1−SCP2
CNVR387.1−RXRG
CNVR6602.2−PDPK1
CEU
YRI
CHB
0.00
0.05
0.10
0.15
0.20
0.25

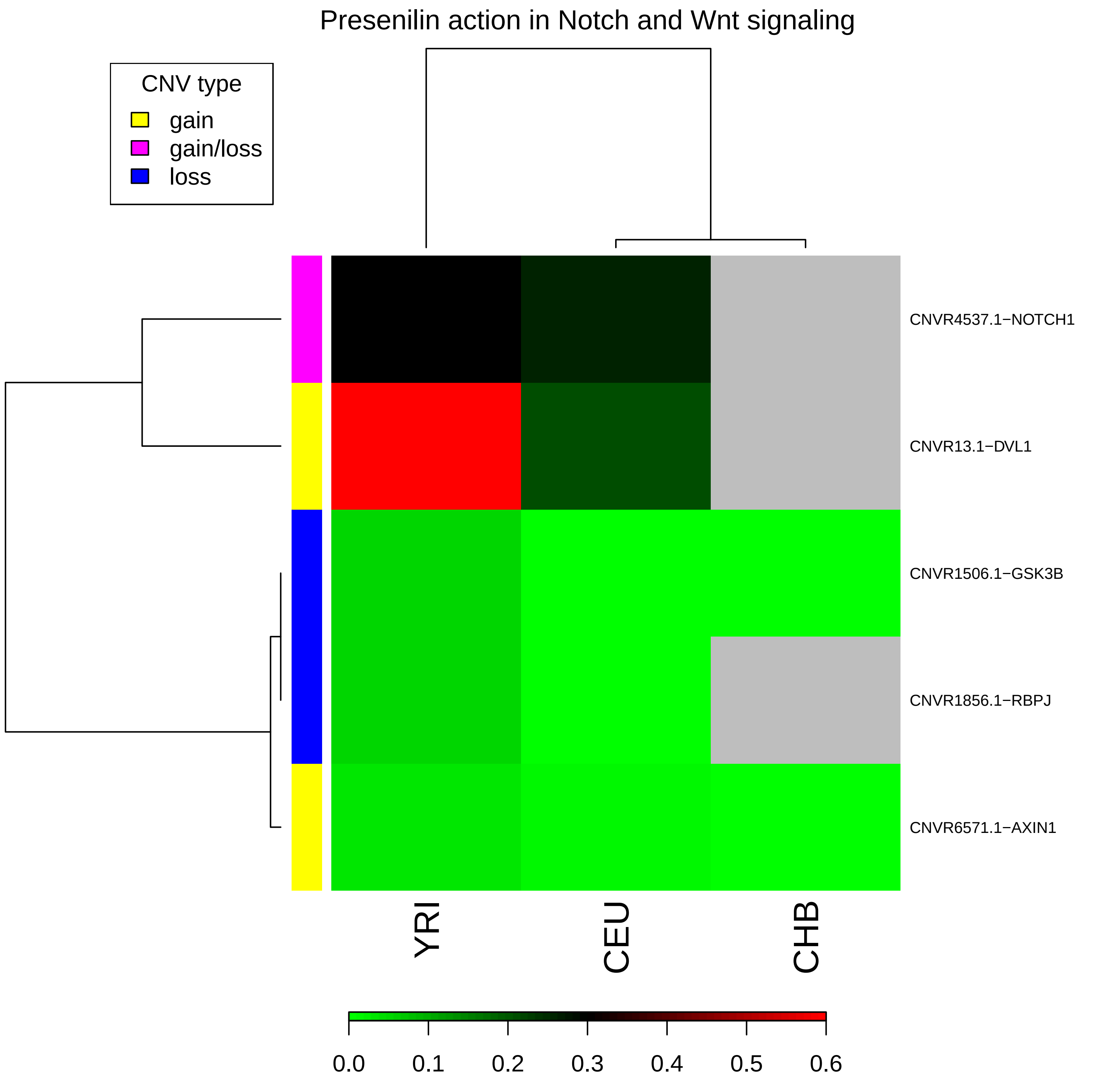
Presenilin action in Notch and Wnt signaling
CNV type
gain
gain/loss
loss
CNVR4537.1−NOTCH1
CNVR13.1−DVL1
CNVR1506.1−GSK3B
CNVR1856.1−RBPJ
CNVR6571.1−AXIN1
YRI
CEU
CHB
0.0
0.1
0.2
0.3
0.4
0.5
0.6

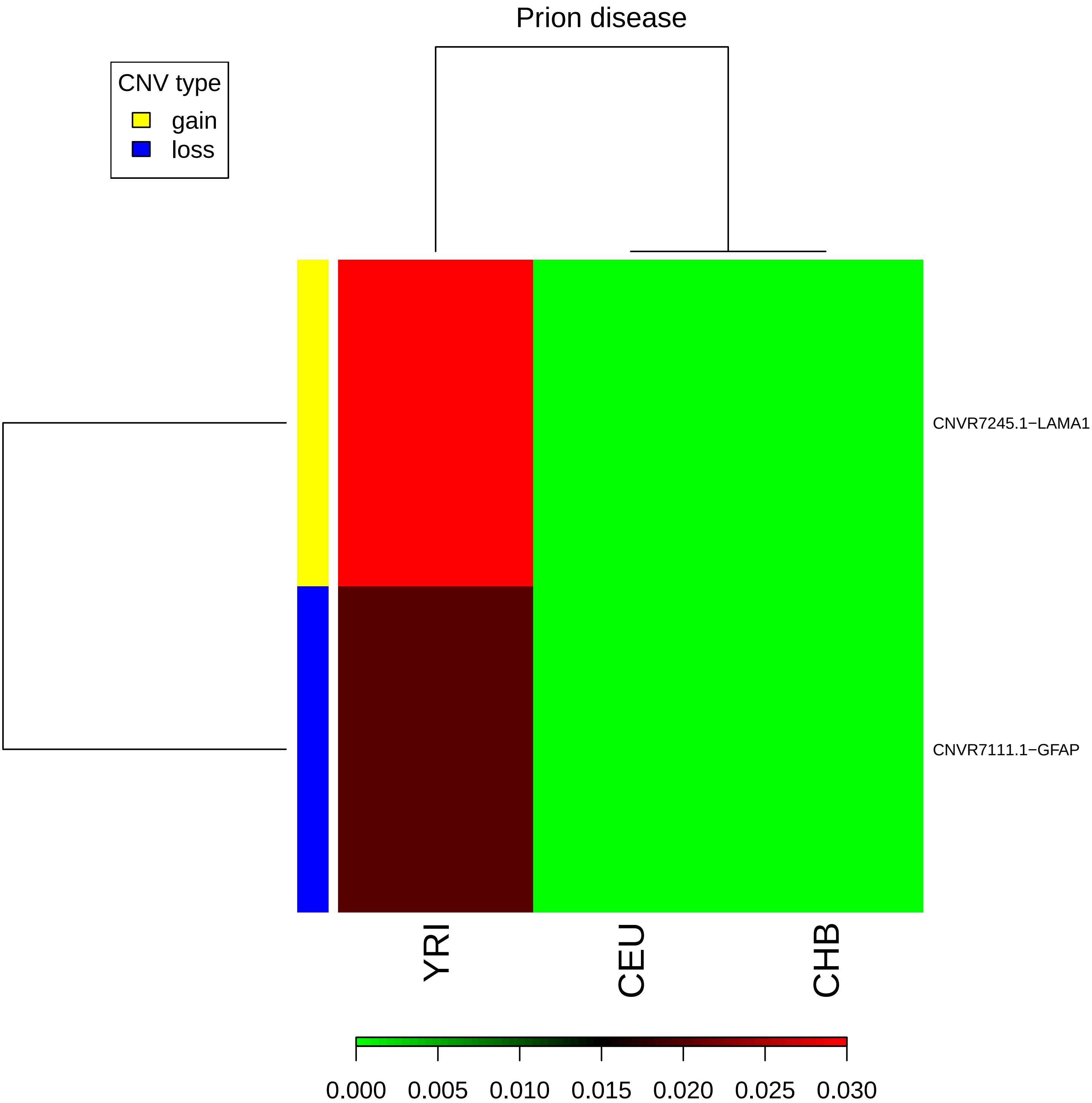
Prion disease
CNV type
gain
loss
CNVR7245.1−LAMA1
CNVR7111.1−GFAP
YRI
CEU
CHB
0.000
0.005
0.010
0.015
0.020
0.025
0.030

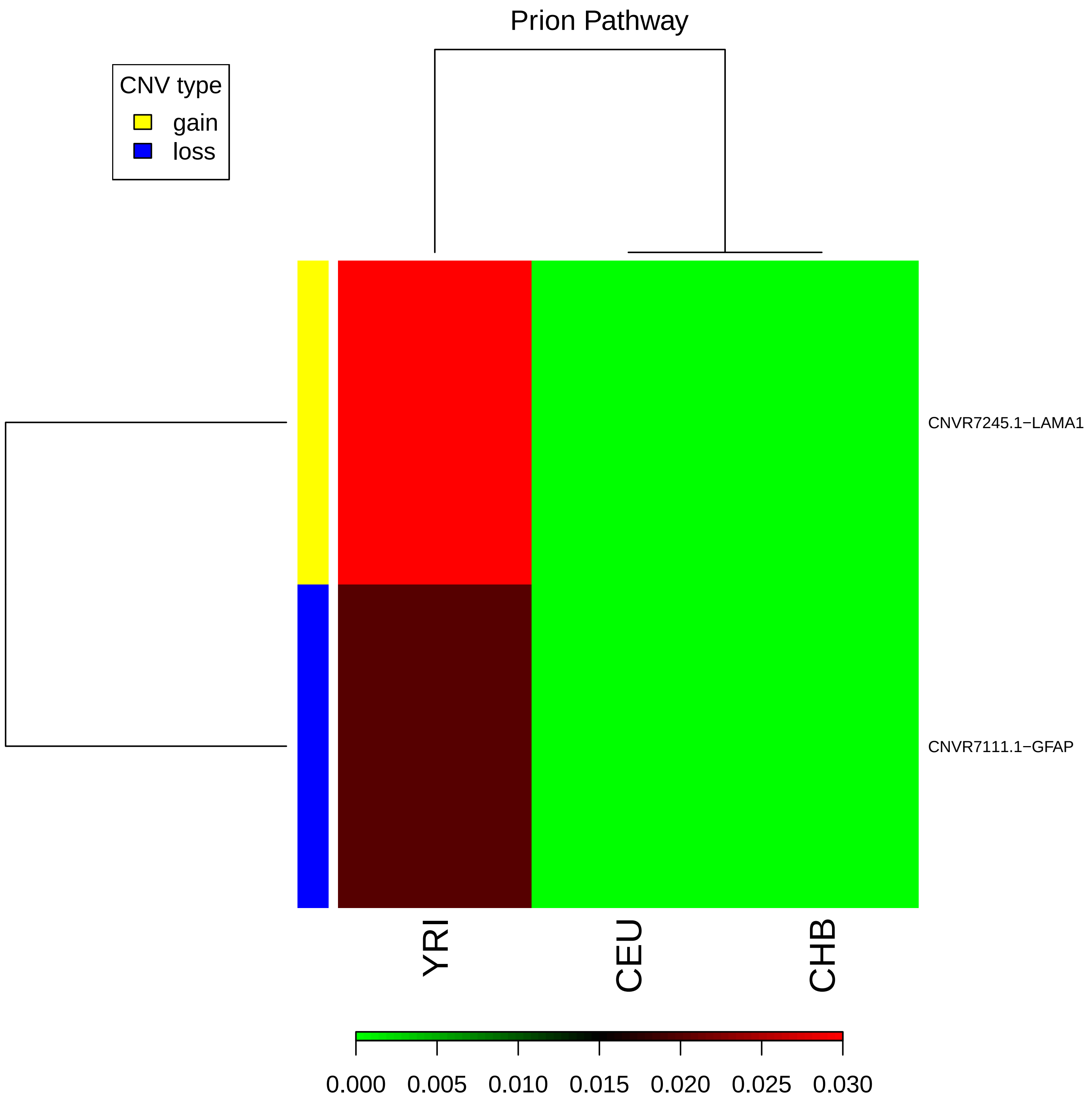
Prion Pathway
CNV type
gain
loss
CNVR7245.1−LAMA1
CNVR7111.1−GFAP
YRI
CEU
CHB
0.000
0.005
0.010
0.015
0.020
0.025
0.030

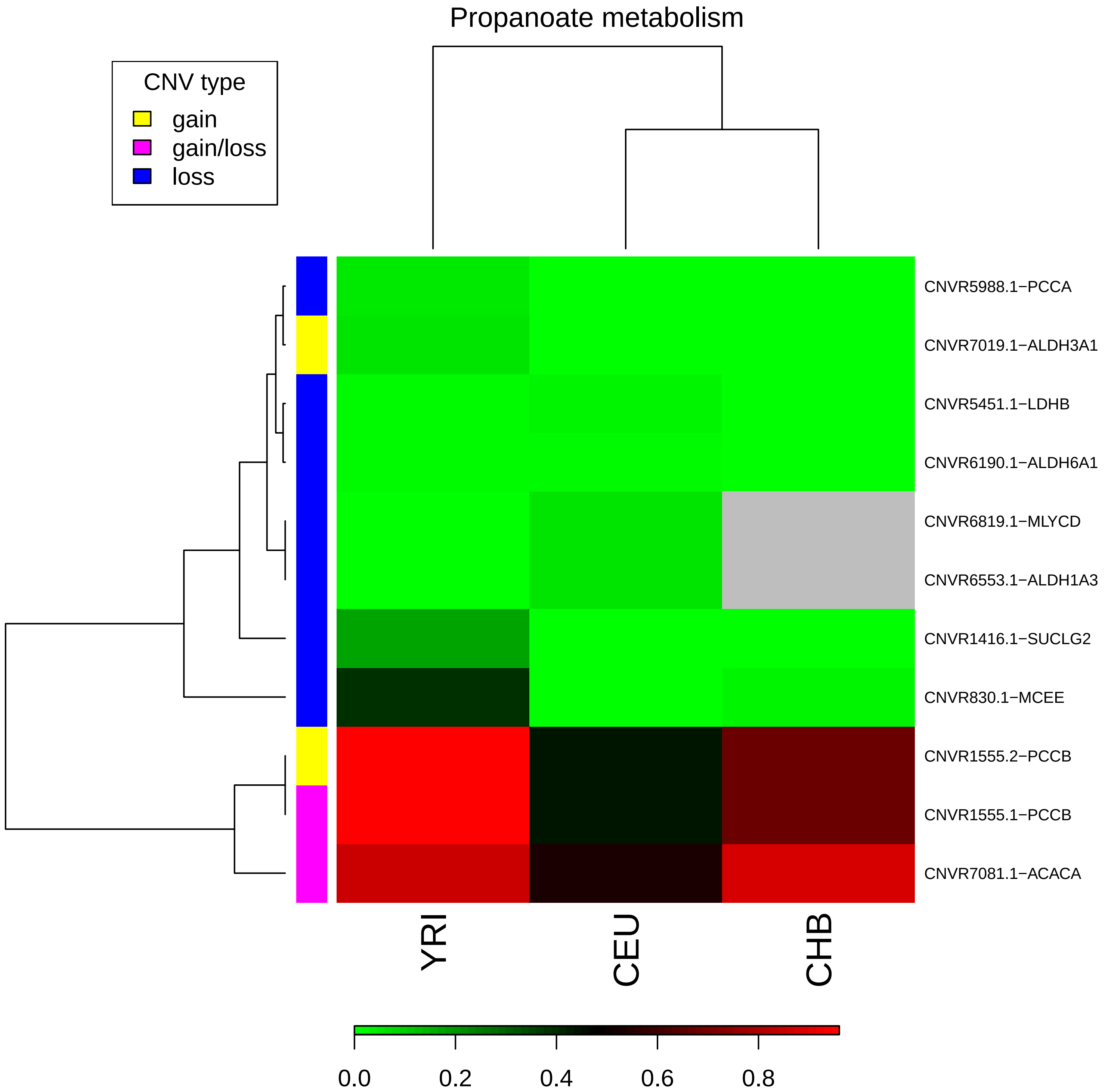
Propanoate metabolism
CNV type
gain
gain/loss
loss
CNVR5988.1−PCCA
CNVR7019.1−ALDH3A1
CNVR5451.1−LDHB
CNVR6190.1−ALDH6A1
CNVR6819.1−MLYCD
CNVR6553.1−ALDH1A3
CNVR1416.1−SUCLG2
CNVR830.1−MCEE
CNVR1555.2−PCCB
CNVR1555.1−PCCB
CNVR7081.1−ACACA
YRI
CEU
CHB
0.0
0.2
0.4
0.6
0.8

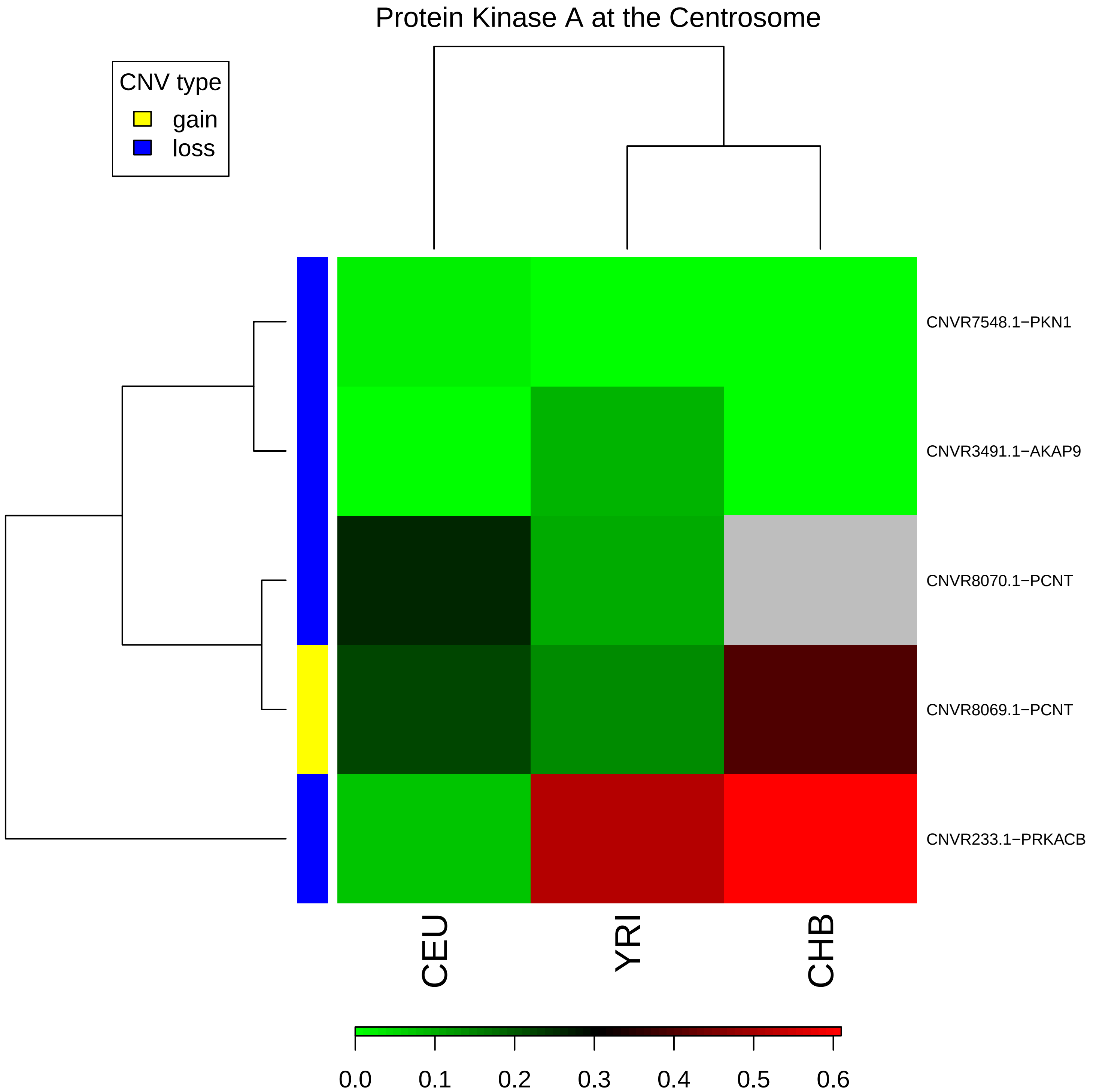
Protein Kinase A at the Centrosome
CNV type
gain
loss
CNVR7548.1−PKN1
CNVR3491.1−AKAP9
CNVR8070.1−PCNT
CNVR8069.1−PCNT
CNVR233.1−PRKACB
CEU
YRI
CHB
0.0
0.1
0.2
0.3
0.4
0.5
0.6

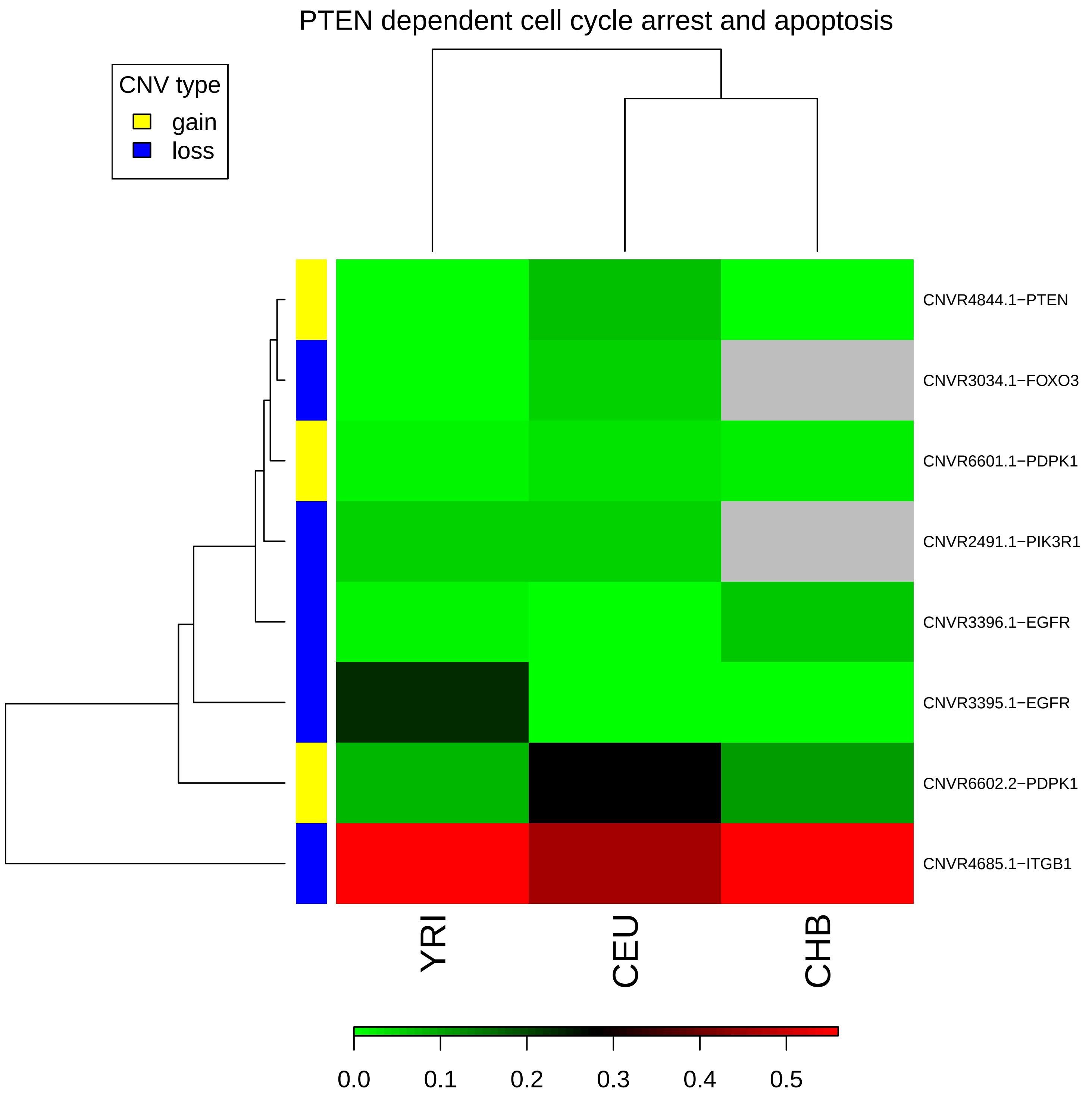
PTEN dependent cell cycle arrest and apoptosis
CNV type
gain
loss
CNVR4844.1−PTEN
CNVR3034.1−FOXO3
CNVR6601.1−PDPK1
CNVR2491.1−PIK3R1
CNVR3396.1−EGFR
CNVR3395.1−EGFR
CNVR6602.2−PDPK1
CNVR4685.1−ITGB1
YRI
CEU
CHB
0.0
0.1
0.2
0.3
0.4
0.5

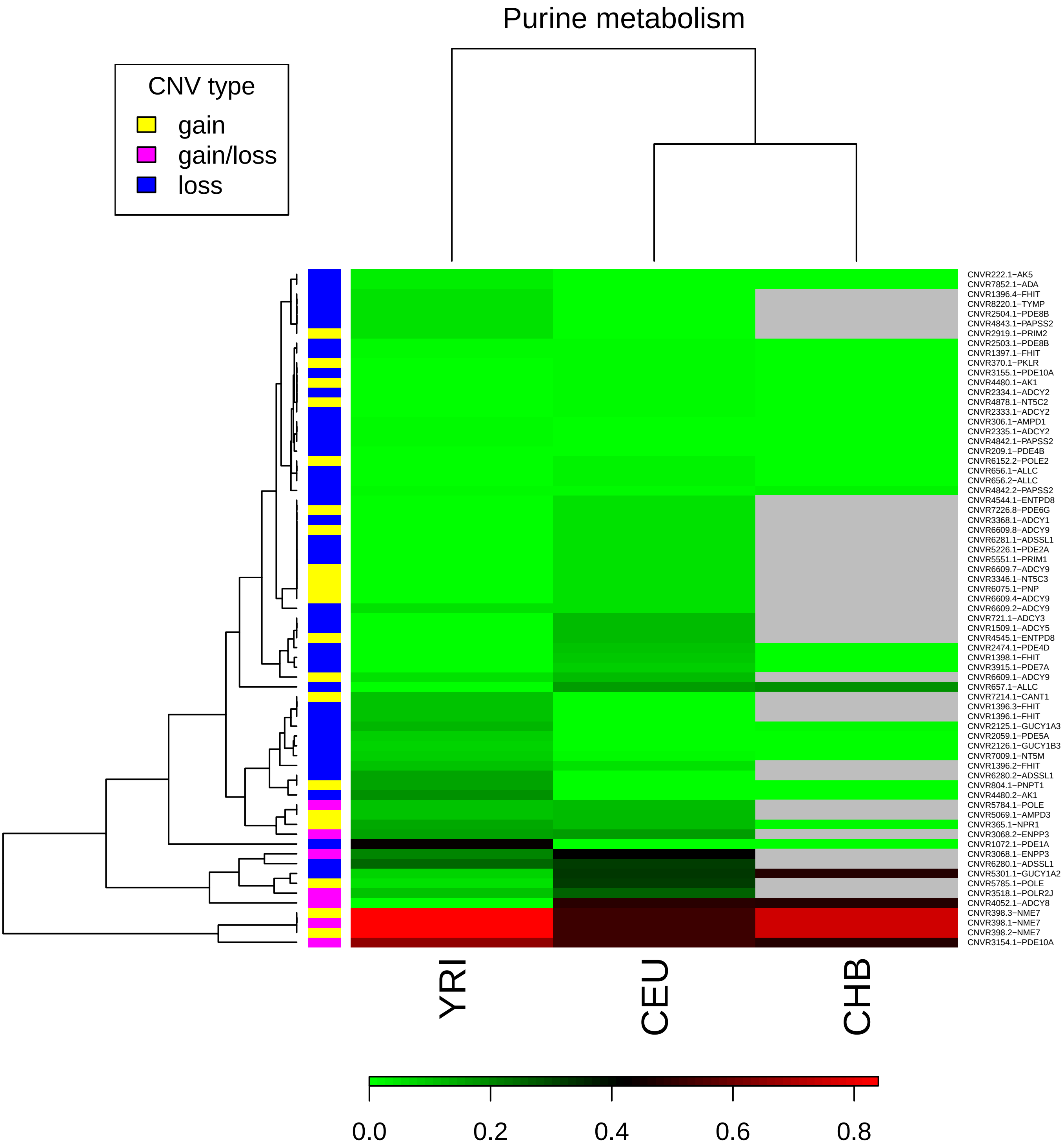
Purine metabolism
CNV type
gain
gain/loss
loss
CNVR222.1−AK5
CNVR7852.1−ADA
CNVR1396.4−FHIT
CNVR8220.1−TYMP
CNVR2504.1−PDE8B
CNVR4843.1−PAPSS2
CNVR2919.1−PRIM2
CNVR2503.1−PDE8B
CNVR1397.1−FHIT
CNVR370.1−PKLR
CNVR3155.1−PDE10A
CNVR4480.1−AK1
CNVR2334.1−ADCY2
CNVR4878.1−NT5C2
CNVR2333.1−ADCY2
CNVR306.1−AMPD1
CNVR2335.1−ADCY2
CNVR4842.1−PAPSS2
CNVR209.1−PDE4B
CNVR6152.2−POLE2
CNVR656.1−ALLC
CNVR656.2−ALLC
CNVR4842.2−PAPSS2
CNVR4544.1−ENTPD8
CNVR7226.8−PDE6G
CNVR3368.1−ADCY1
CNVR6609.8−ADCY9
CNVR6281.1−ADSSL1
CNVR5226.1−PDE2A
CNVR5551.1−PRIM1
CNVR6609.7−ADCY9
CNVR3346.1−NT5C3
CNVR6075.1−PNP
CNVR6609.4−ADCY9
CNVR6609.2−ADCY9
CNVR721.1−ADCY3
CNVR1509.1−ADCY5
CNVR4545.1−ENTPD8
CNVR2474.1−PDE4D
CNVR1398.1−FHIT
CNVR3915.1−PDE7A
CNVR6609.1−ADCY9
CNVR657.1−ALLC
CNVR7214.1−CANT1
CNVR1396.3−FHIT
CNVR1396.1−FHIT
CNVR2125.1−GUCY1A3
CNVR2059.1−PDE5A
CNVR2126.1−GUCY1B3
CNVR7009.1−NT5M
CNVR1396.2−FHIT
CNVR6280.2−ADSSL1
CNVR804.1−PNPT1
CNVR4480.2−AK1
CNVR5784.1−POLE
CNVR5069.1−AMPD3
CNVR365.1−NPR1
CNVR3068.2−ENPP3
CNVR1072.1−PDE1A
CNVR3068.1−ENPP3
CNVR6280.1−ADSSL1
CNVR5301.1−GUCY1A2
CNVR5785.1−POLE
CNVR3518.1−POLR2J
CNVR4052.1−ADCY8
CNVR398.3−NME7
CNVR398.1−NME7
CNVR398.2−NME7
CNVR3154.1−PDE10A
YRI
CEU
CHB
0.0
0.2
0.4
0.6
0.8

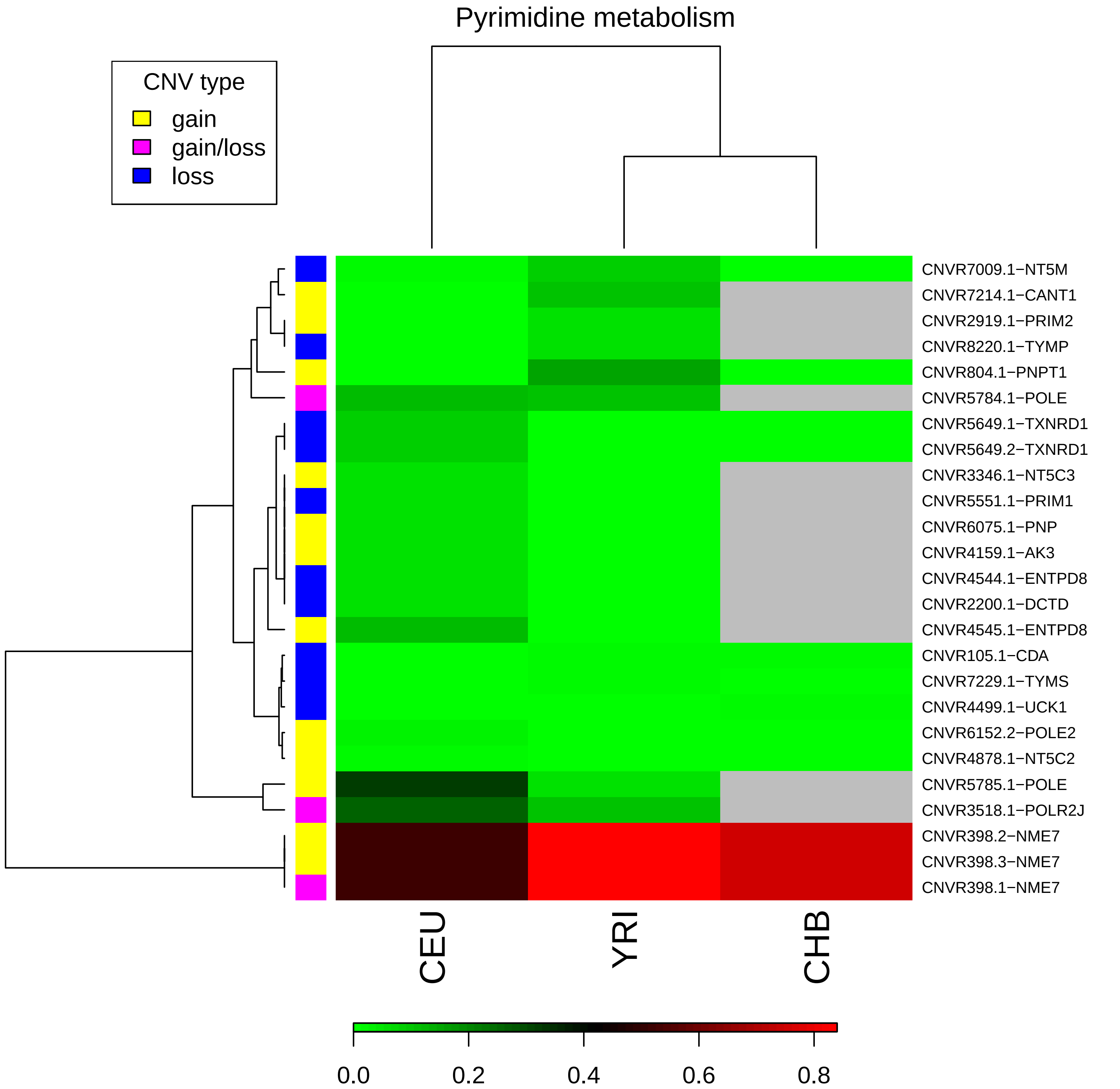

Pyrimidine metabolism
CNV type
gain
gain/loss
loss
CNVR7009.1−NT5M
CNVR7214.1−CANT1
CNVR2919.1−PRIM2
CNVR8220.1−TYMP
CNVR804.1−PNPT1
CNVR5784.1−POLE
CNVR5649.1−TXNRD1
CNVR5649.2−TXNRD1
CNVR3346.1−NT5C3
CNVR5551.1−PRIM1
CNVR6075.1−PNP
CNVR4159.1−AK3
CNVR4544.1−ENTPD8
CNVR2200.1−DCTD
CNVR4545.1−ENTPD8
CNVR105.1−CDA
CNVR7229.1−TYMS
CNVR4499.1−UCK1
CNVR6152.2−POLE2
CNVR4878.1−NT5C2
CNVR5785.1−POLE
CNVR3518.1−POLR2J
CNVR398.2−NME7
CNVR398.3−NME7
CNVR398.1−NME7
CEU
YRI
CHB
0.0
0.2
0.4
0.6
0.8

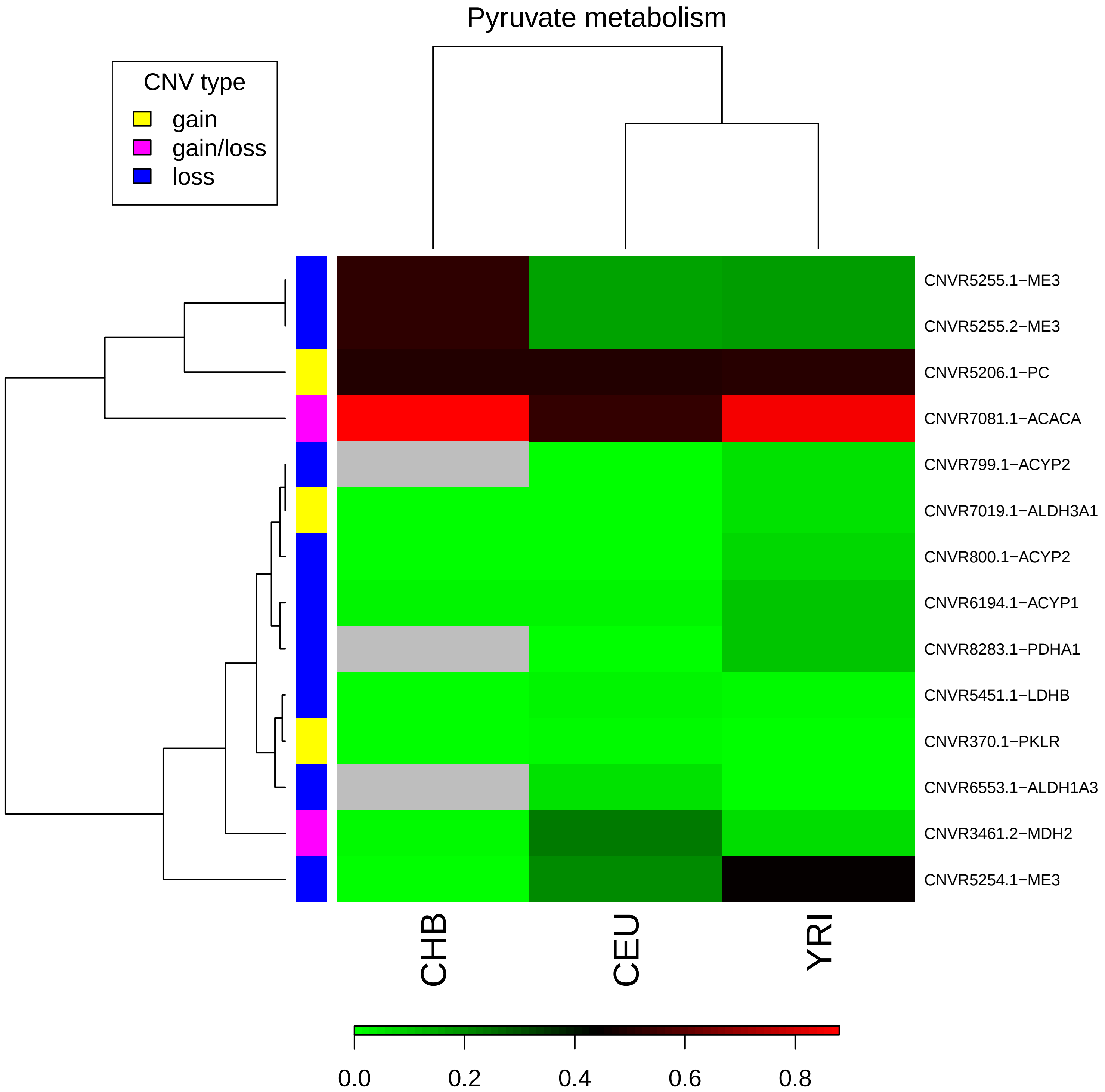
Pyruvate metabolism
CNV type
gain
gain/loss
loss
CNVR5255.1−ME3
CNVR5255.2−ME3
CNVR5206.1−PC
CNVR7081.1−ACACA
CNVR799.1−ACYP2
CNVR7019.1−ALDH3A1
CNVR800.1−ACYP2
CNVR6194.1−ACYP1
CNVR8283.1−PDHA1
CNVR5451.1−LDHB
CNVR370.1−PKLR
CNVR6553.1−ALDH1A3
CNVR3461.2−MDH2
CNVR5254.1−ME3
CHB
CEU
YRI
0.0
0.2
0.4
0.6
0.8

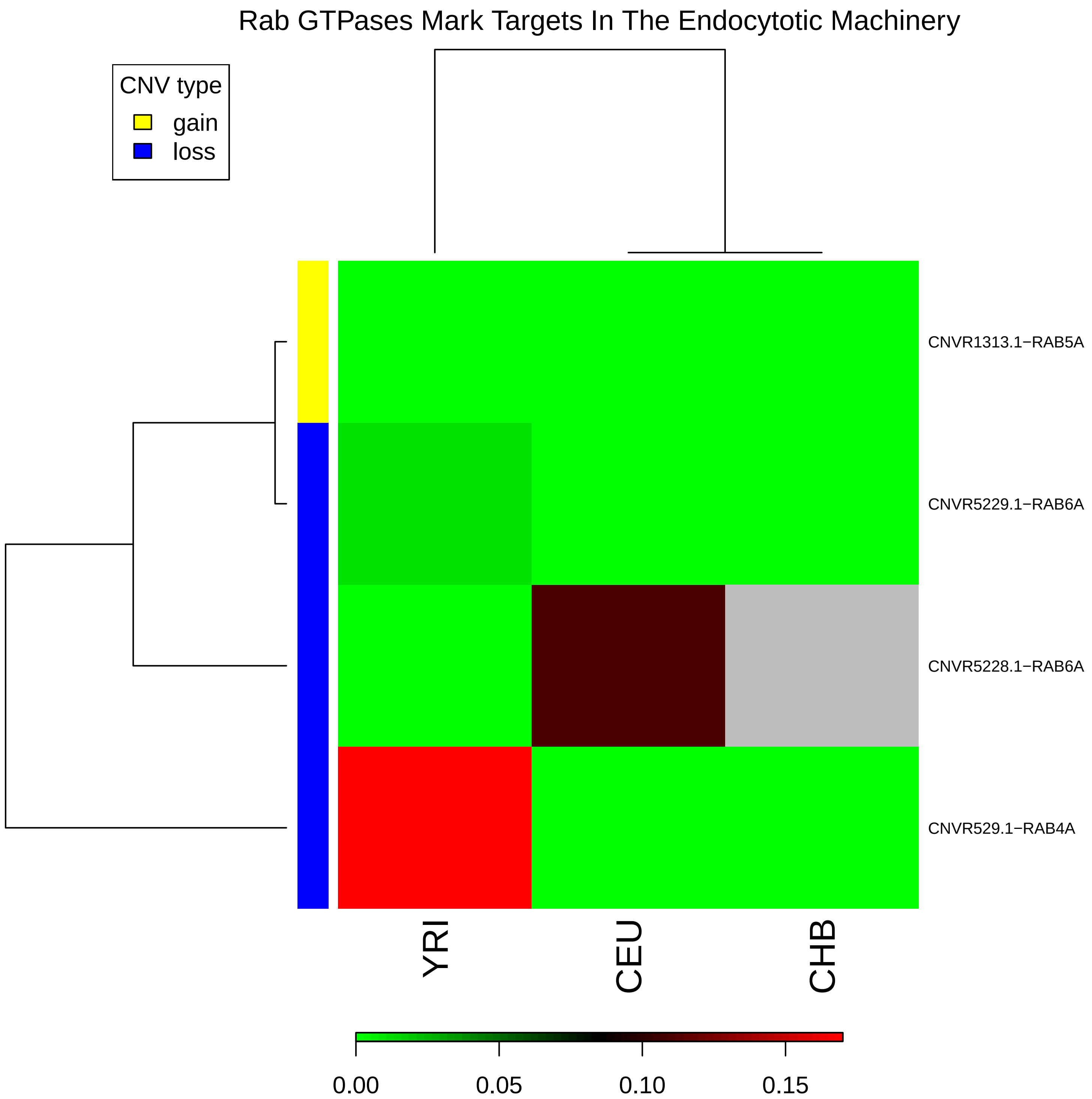

Rab GTPases Mark Targets In The Endocytotic Machinery
CNV type
gain
loss
CNVR1313.1−RAB5A
CNVR5229.1−RAB6A
CNVR5228.1−RAB6A
CNVR529.1−RAB4A
YRI
CEU
CHB
0.00
0.05
0.10
0.15

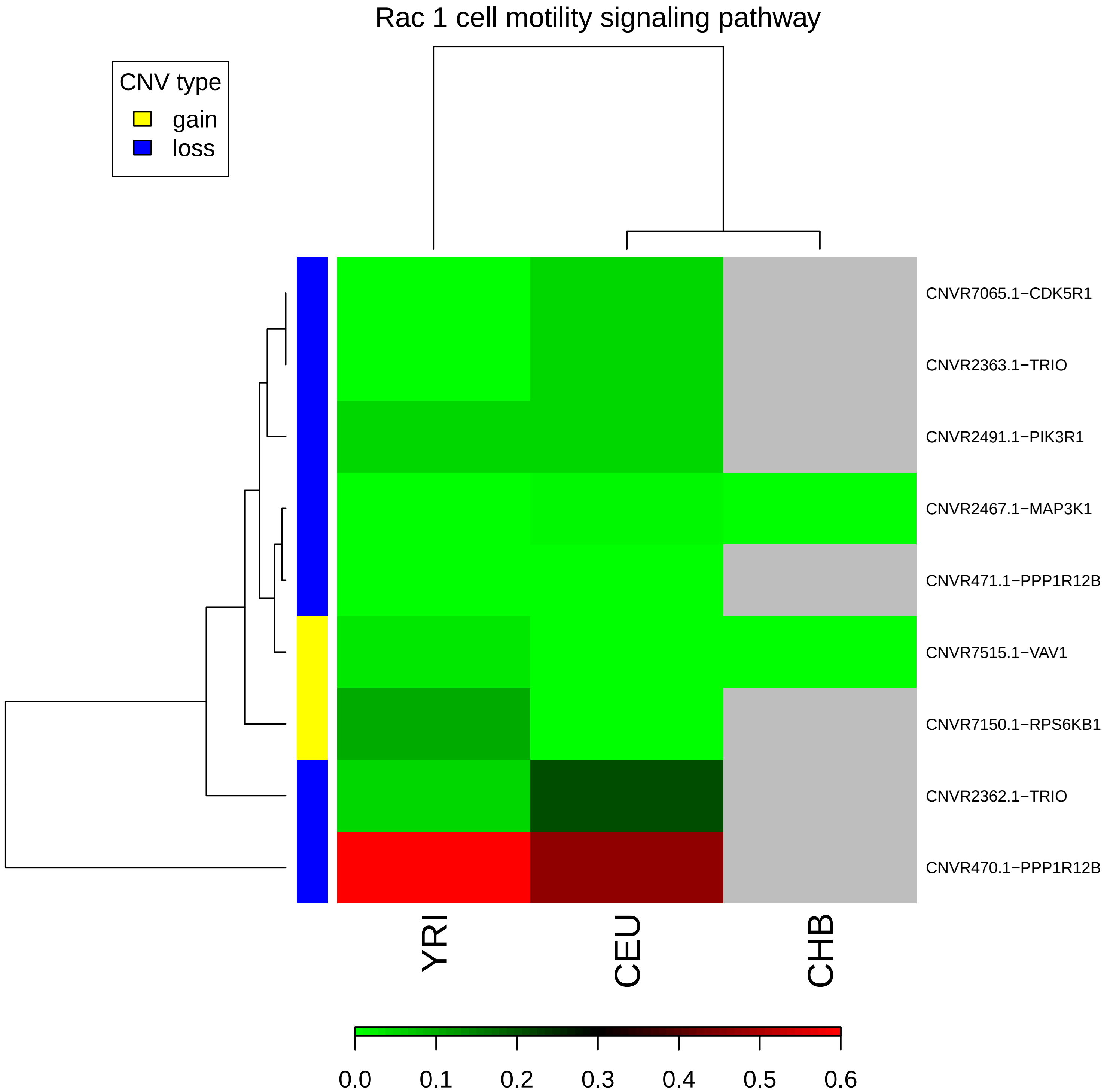

Rac 1 cell motility signaling pathway
CNV type
gain
loss
CNVR7065.1−CDK5R1
CNVR2363.1−TRIO
CNVR2491.1−PIK3R1
CNVR2467.1−MAP3K1
CNVR471.1−PPP1R12B
CNVR7515.1−VAV1
CNVR7150.1−RPS6KB1
CNVR2362.1−TRIO
CNVR470.1−PPP1R12B
YRI
CEU
CHB
0.0
0.1
0.2
0.3
0.4
0.5
0.6

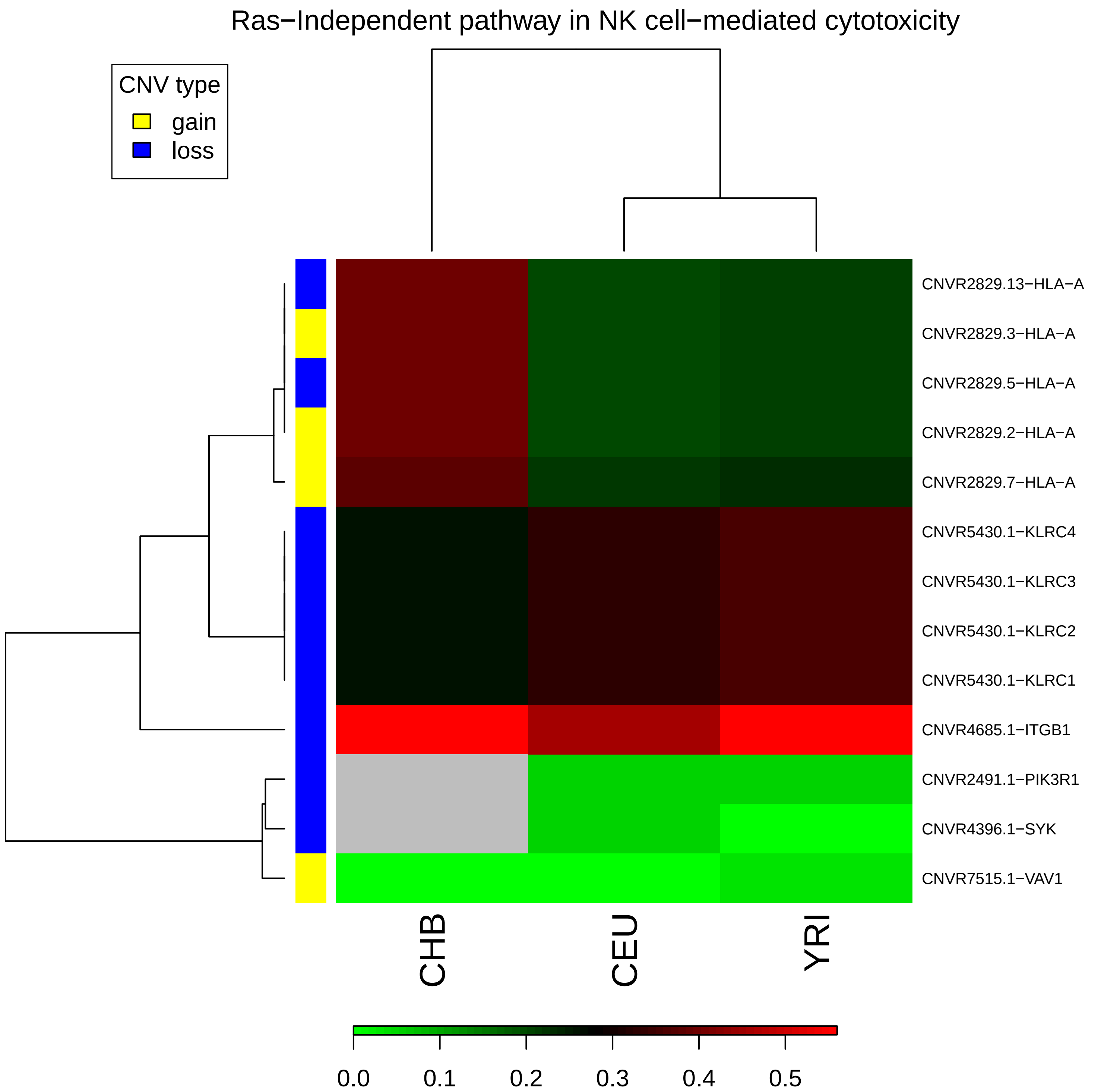

Ras−Independent pathway in NK cell−mediated cytotoxicity
CNV type
gain
loss
CNVR2829.13−HLA−A
CNVR2829.3−HLA−A
CNVR2829.5−HLA−A
CNVR2829.2−HLA−A
CNVR2829.7−HLA−A
CNVR5430.1−KLRC4
CNVR5430.1−KLRC3
CNVR5430.1−KLRC2
CNVR5430.1−KLRC1
CNVR4685.1−ITGB1
CNVR2491.1−PIK3R1
CNVR4396.1−SYK
CNVR7515.1−VAV1
CHB
CEU
YRI
0.0
0.1
0.2
0.3
0.4
0.5

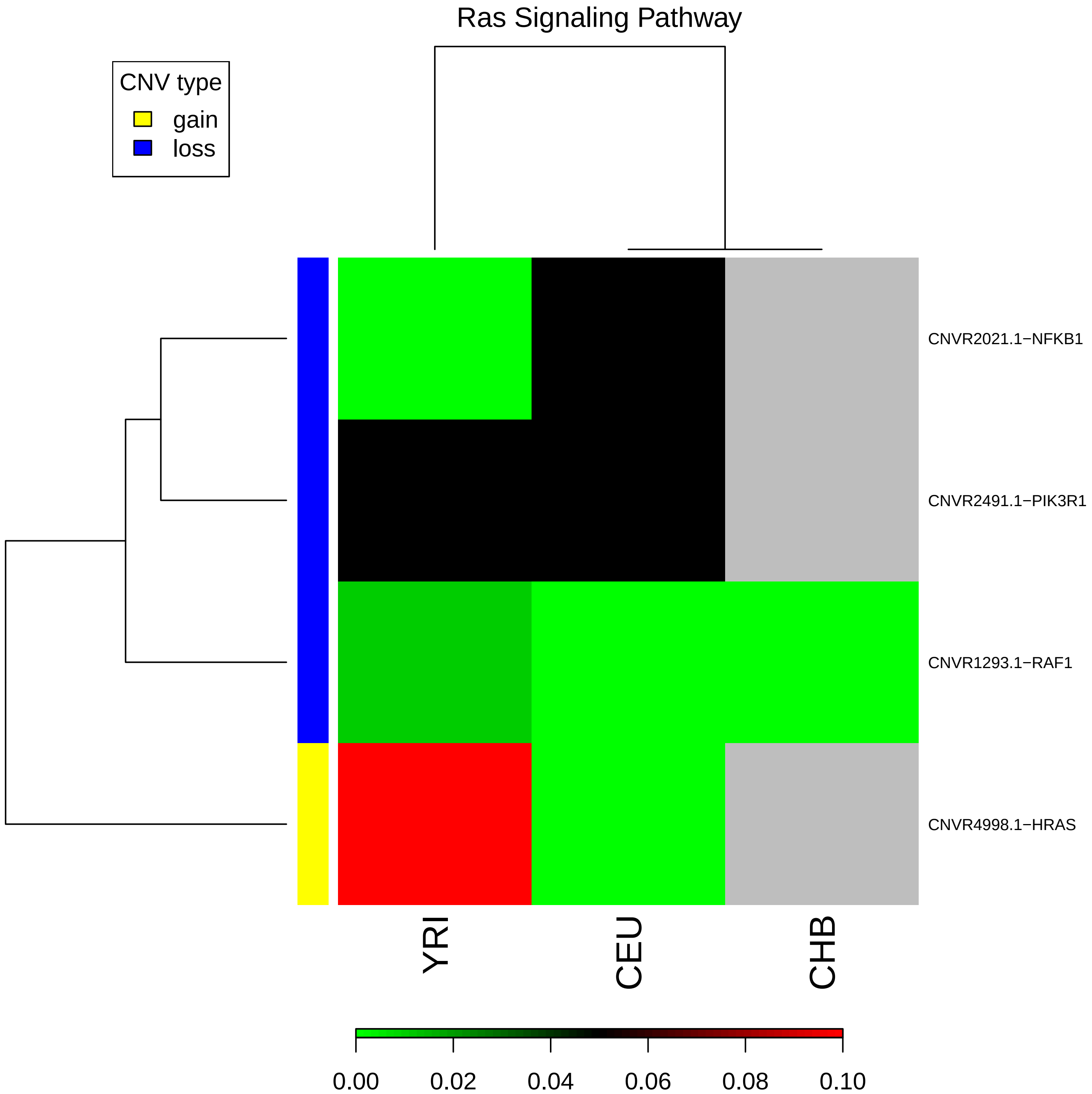

Ras Signaling Pathway
CNV type
gain
loss
CNVR2021.1−NFKB1
CNVR2491.1−PIK3R1
CNVR1293.1−RAF1
CNVR4998.1−HRAS
YRI
CEU
CHB
0.00
0.02
0.04
0.06
0.08
0.10

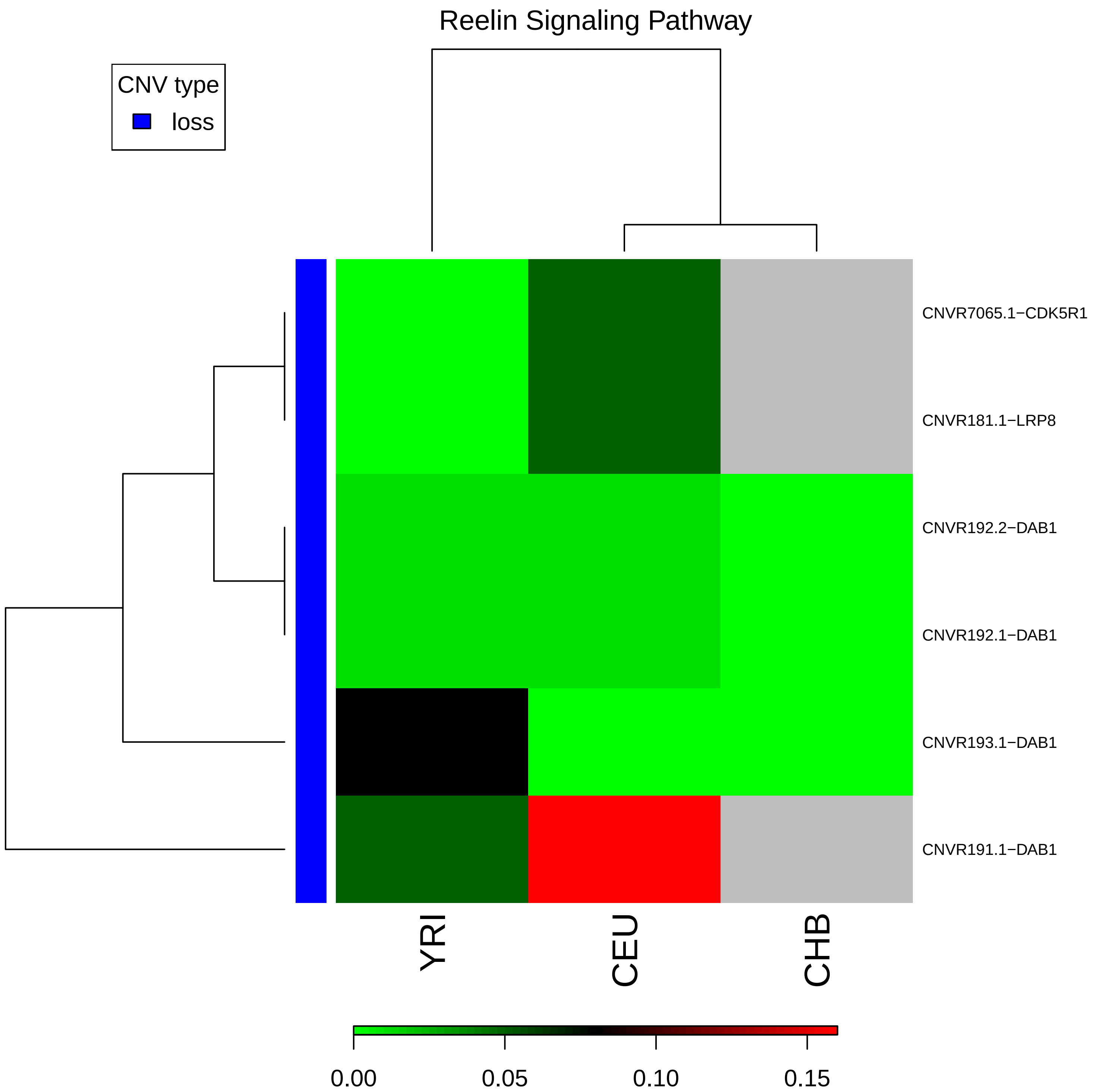

Reelin Signaling Pathway
CNV type
loss
CNVR7065.1−CDK5R1
CNVR181.1−LRP8
CNVR192.2−DAB1
CNVR192.1−DAB1
CNVR193.1−DAB1
CNVR191.1−DAB1
YRI
CEU
CHB
0.00
0.05
0.10
0.15

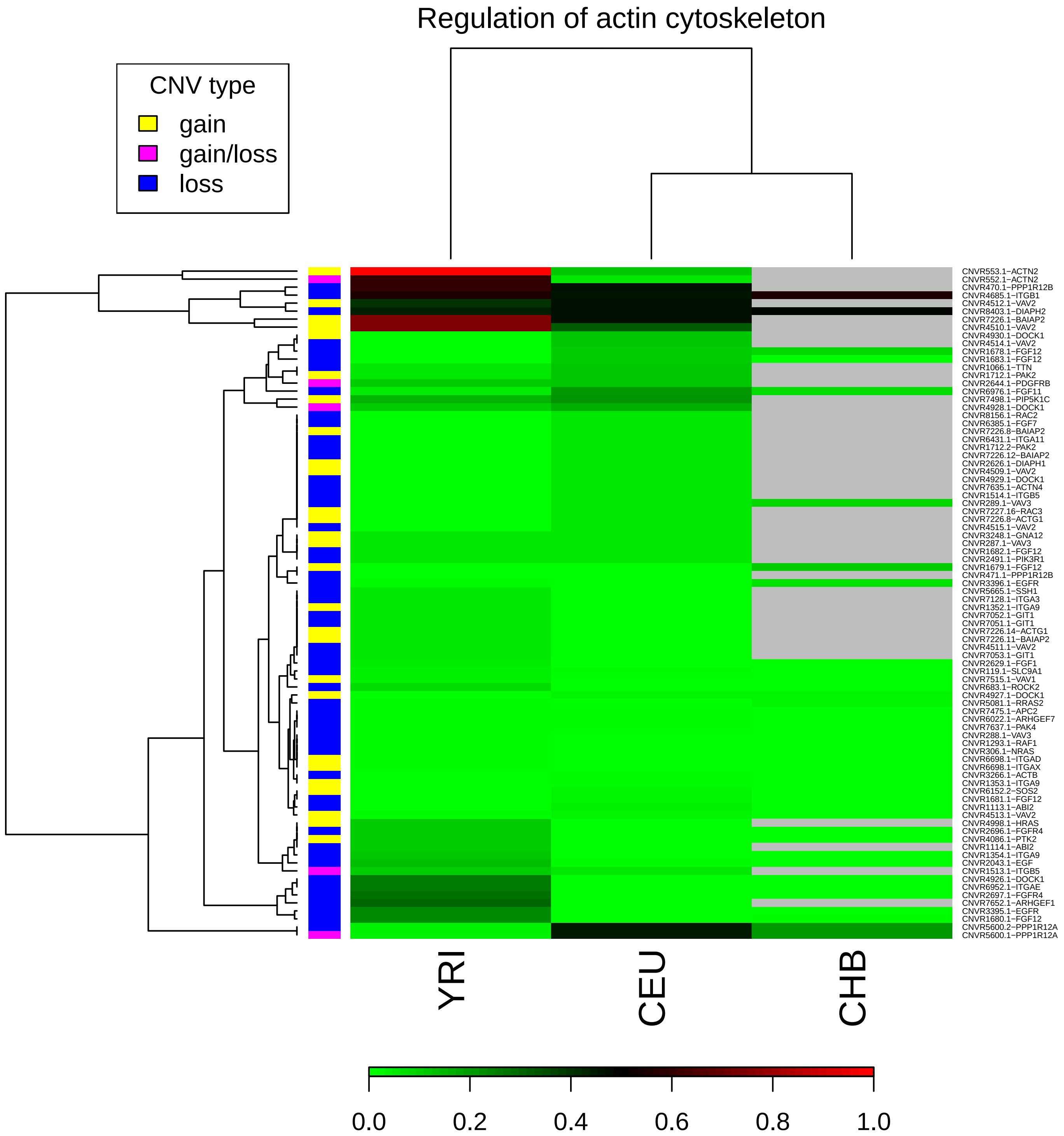
Regulation of actin cytoskeleton
CNV type
gain
gain/loss
loss
CNVR553.1−ACTN2
CNVR552.1−ACTN2
CNVR470.1−PPP1R12B
CNVR4685.1−ITGB1
CNVR4512.1−VAV2
CNVR8403.1−DIAPH2
CNVR7226.1−BAIAP2
CNVR4510.1−VAV2
CNVR4930.1−DOCK1
CNVR4514.1−VAV2
CNVR1678.1−FGF12
CNVR1683.1−FGF12
CNVR1066.1−TTN
CNVR1712.1−PAK2
CNVR2644.1−PDGFRB
CNVR6976.1−FGF11
CNVR7498.1−PIP5K1C
CNVR4928.1−DOCK1
CNVR8156.1−RAC2
CNVR6385.1−FGF7
CNVR7226.8−BAIAP2
CNVR6431.1−ITGA11
CNVR1712.2−PAK2
CNVR7226.12−BAIAP2
CNVR2626.1−DIAPH1
CNVR4509.1−VAV2
CNVR4929.1−DOCK1
CNVR7635.1−ACTN4
CNVR1514.1−ITGB5
CNVR289.1−VAV3
CNVR7227.16−RAC3
CNVR7226.8−ACTG1
CNVR4515.1−VAV2
CNVR3248.1−GNA12
CNVR287.1−VAV3
CNVR1682.1−FGF12
CNVR2491.1−PIK3R1
CNVR1679.1−FGF12
CNVR471.1−PPP1R12B
CNVR3396.1−EGFR
CNVR5665.1−SSH1
CNVR7128.1−ITGA3
CNVR1352.1−ITGA9
CNVR7052.1−GIT1
CNVR7051.1−GIT1
CNVR7226.14−ACTG1
CNVR7226.11−BAIAP2
CNVR4511.1−VAV2
CNVR7053.1−GIT1
CNVR2629.1−FGF1
CNVR119.1−SLC9A1
CNVR7515.1−VAV1
CNVR683.1−ROCK2
CNVR4927.1−DOCK1
CNVR5081.1−RRAS2
CNVR7475.1−APC2
CNVR6022.1−ARHGEF7
CNVR7637.1−PAK4
CNVR288.1−VAV3
CNVR1293.1−RAF1
CNVR306.1−NRAS
CNVR6698.1−ITGAD
CNVR6698.1−ITGAX
CNVR3266.1−ACTB
CNVR1353.1−ITGA9
CNVR6152.2−SOS2
CNVR1681.1−FGF12
CNVR1113.1−ABI2
CNVR4513.1−VAV2
CNVR4998.1−HRAS
CNVR2696.1−FGFR4
CNVR4086.1−PTK2
CNVR1114.1−ABI2
CNVR1354.1−ITGA9
CNVR2043.1−EGF
CNVR1513.1−ITGB5
CNVR4926.1−DOCK1
CNVR6952.1−ITGAE
CNVR2697.1−FGFR4
CNVR7652.1−ARHGEF1
CNVR3395.1−EGFR
CNVR1680.1−FGF12
CNVR5600.2−PPP1R12A
CNVR5600.1−PPP1R12A
YRI
CEU
CHB
0.0
0.2
0.4
0.6
0.8
1.0

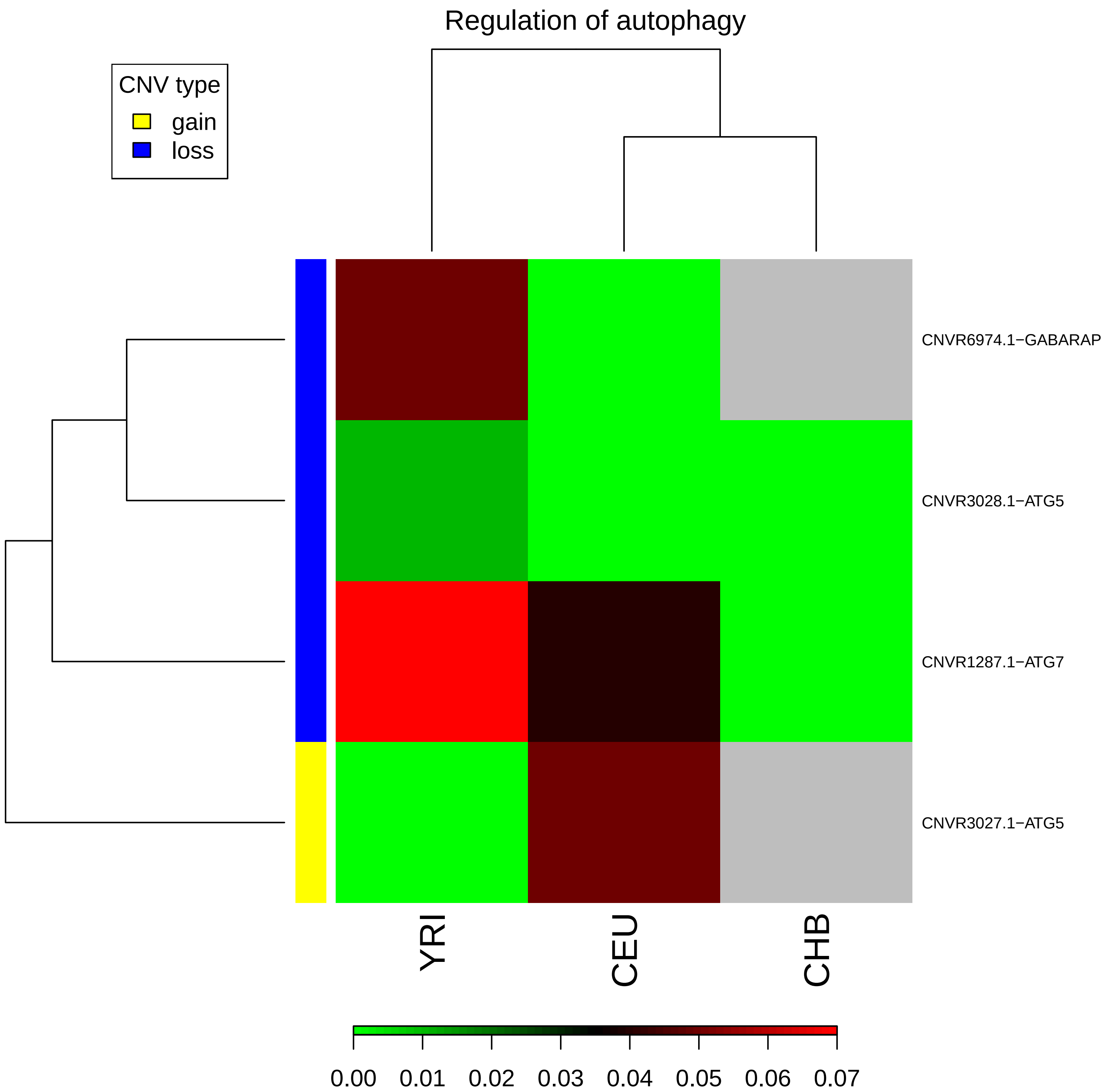
Regulation of autophagy
CNV type
gain
loss
CNVR6974.1−GABARAP
CNVR3028.1−ATG5
CNVR1287.1−ATG7
CNVR3027.1−ATG5
YRI
CEU
CHB
0.00
0.01
0.02
0.03
0.04
0.05
0.06
0.07

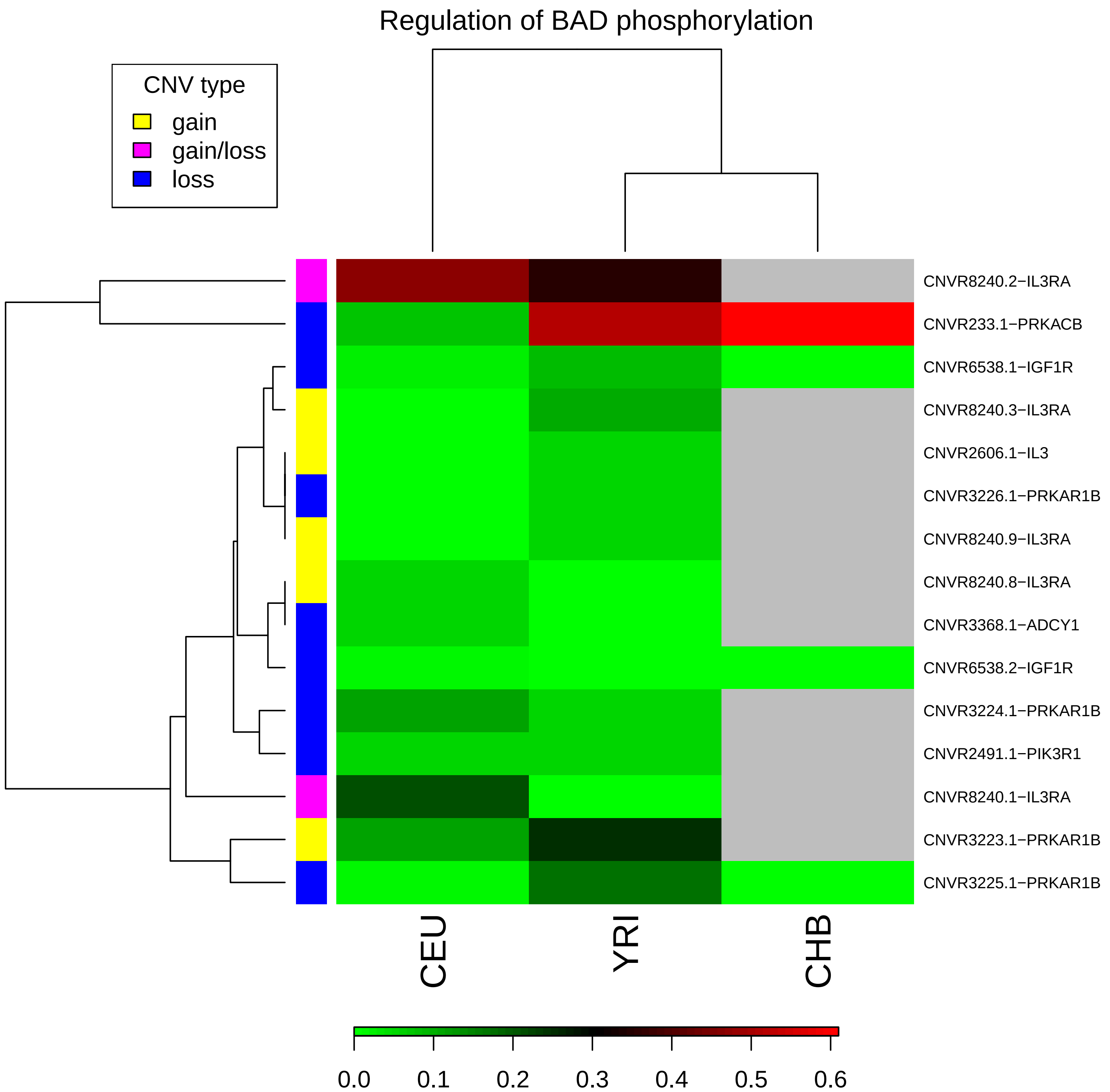
Regulation of BAD phosphorylation
CNV type
gain
gain/loss
loss
CNVR8240.2−IL3RA
CNVR233.1−PRKACB
CNVR6538.1−IGF1R
CNVR8240.3−IL3RA
CNVR2606.1−IL3
CNVR3226.1−PRKAR1B
CNVR8240.9−IL3RA
CNVR8240.8−IL3RA
CNVR3368.1−ADCY1
CNVR6538.2−IGF1R
CNVR3224.1−PRKAR1B
CNVR2491.1−PIK3R1
CNVR8240.1−IL3RA
CNVR3223.1−PRKAR1B
CNVR3225.1−PRKAR1B
CEU
YRI
CHB
0.0
0.1
0.2
0.3
0.4
0.5
0.6

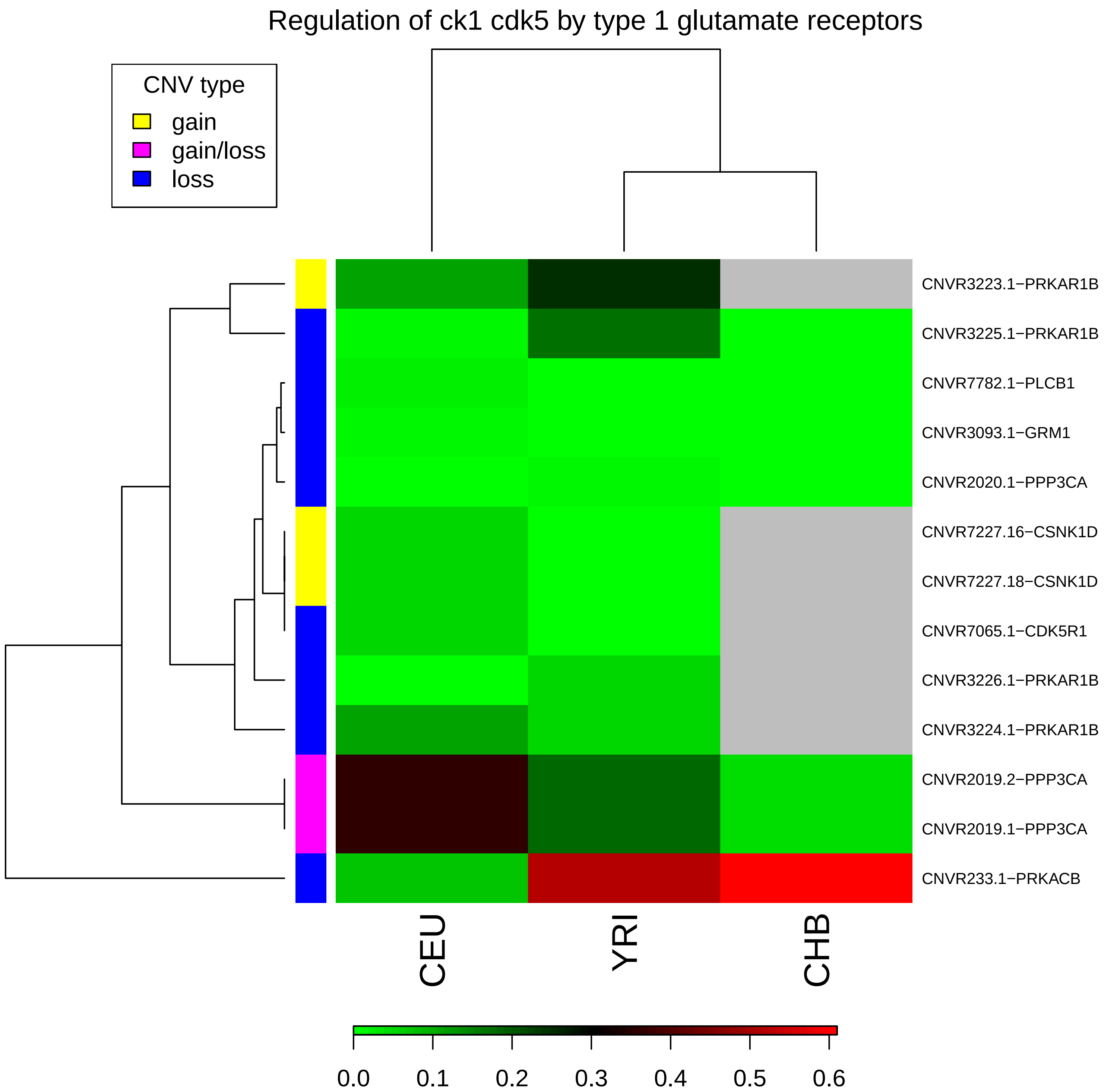

Regulation of ck1 cdk5 by type 1 glutamate receptors
CNV type
gain
gain/loss
loss
CNVR3223.1−PRKAR1B
CNVR3225.1−PRKAR1B
CNVR7782.1−PLCB1
CNVR3093.1−GRM1
CNVR2020.1−PPP3CA
CNVR7227.16−CSNK1D
CNVR7227.18−CSNK1D
CNVR7065.1−CDK5R1
CNVR3226.1−PRKAR1B
CNVR3224.1−PRKAR1B
CNVR2019.2−PPP3CA
CNVR2019.1−PPP3CA
CNVR233.1−PRKACB
CEU
YRI
CHB
0.0
0.1
0.2
0.3
0.4
0.5
0.6

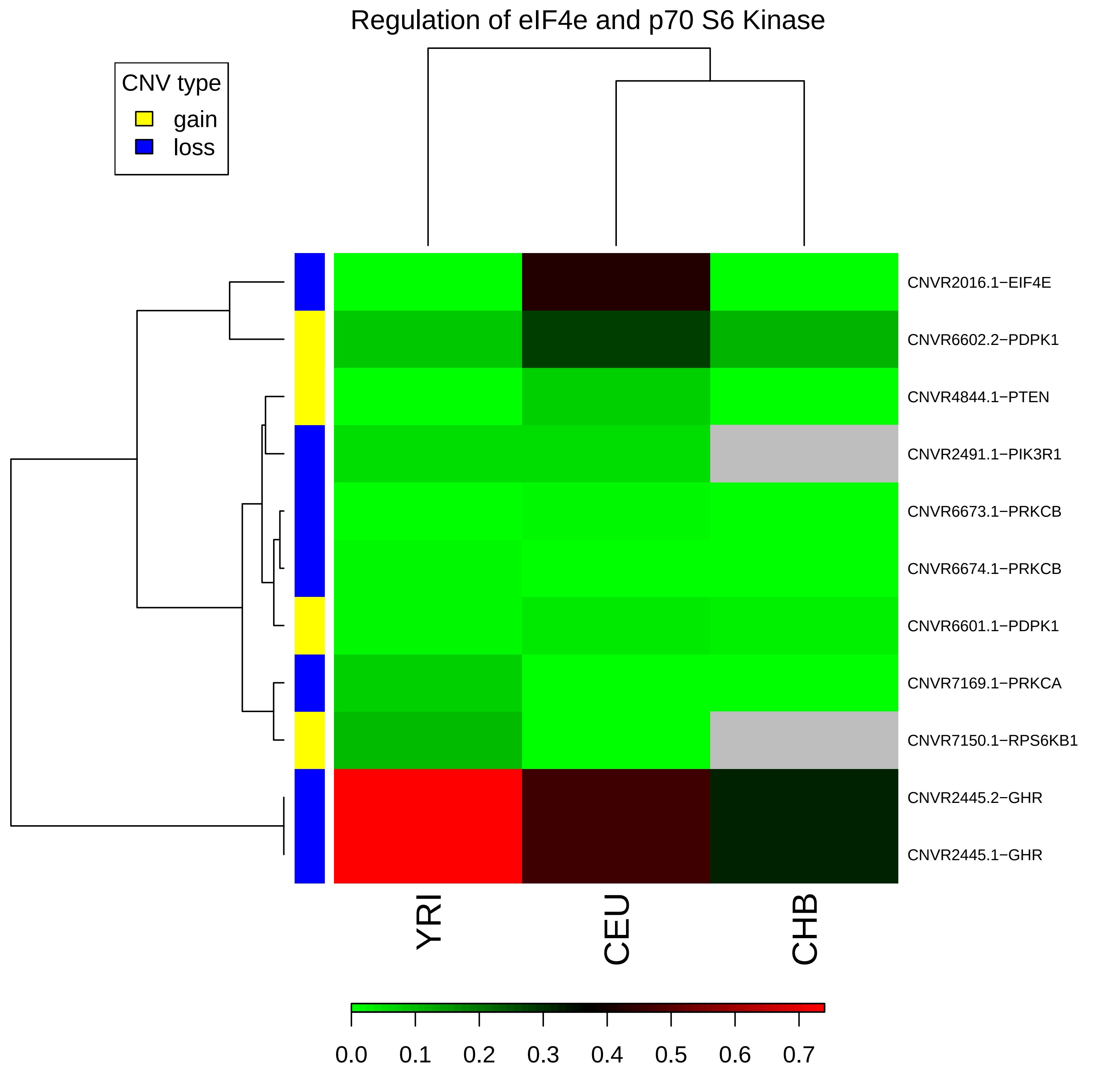

Regulation of eIF4e and p70 S6 Kinase
CNV type
gain
loss
CNVR2016.1−EIF4E
CNVR6602.2−PDPK1
CNVR4844.1−PTEN
CNVR2491.1−PIK3R1
CNVR6673.1−PRKCB
CNVR6674.1−PRKCB
CNVR6601.1−PDPK1
CNVR7169.1−PRKCA
CNVR7150.1−RPS6KB1
CNVR2445.2−GHR
CNVR2445.1−GHR
YRI
CEU
CHB
0.0
0.1
0.2
0.3
0.4
0.5
0.6
0.7

## Regulation of hematopoiesis by cytokines

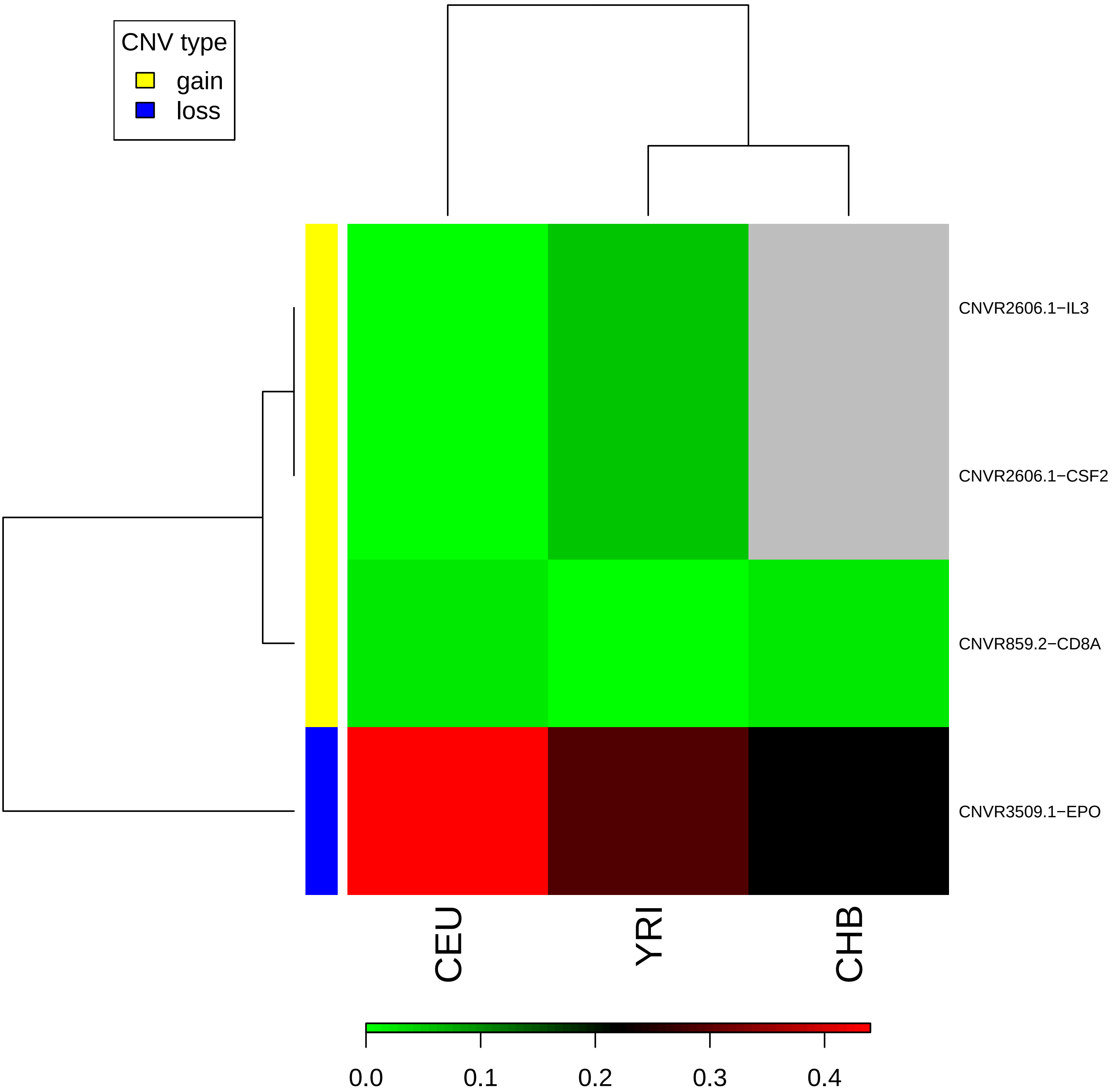

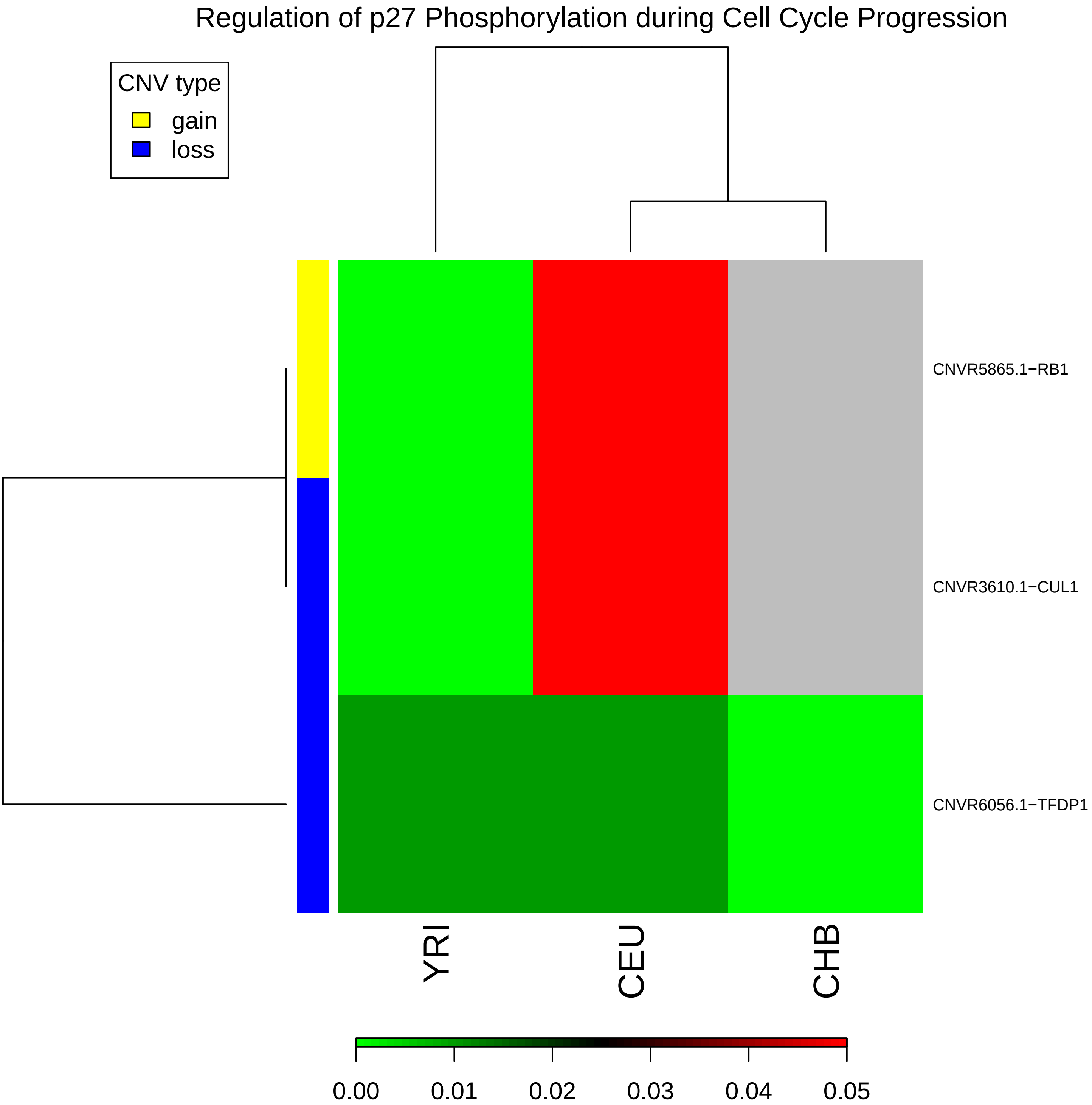

Regulation of p27 Phosphorylation during Cell Cycle Progression
CNV type
gain
loss
CNVR5865.1−RB1
CNVR3610.1−CUL1
CNVR6056.1−TFDP1
YRI
CEU
CHB
0.00
0.01
0.02
0.03
0.04
0.05

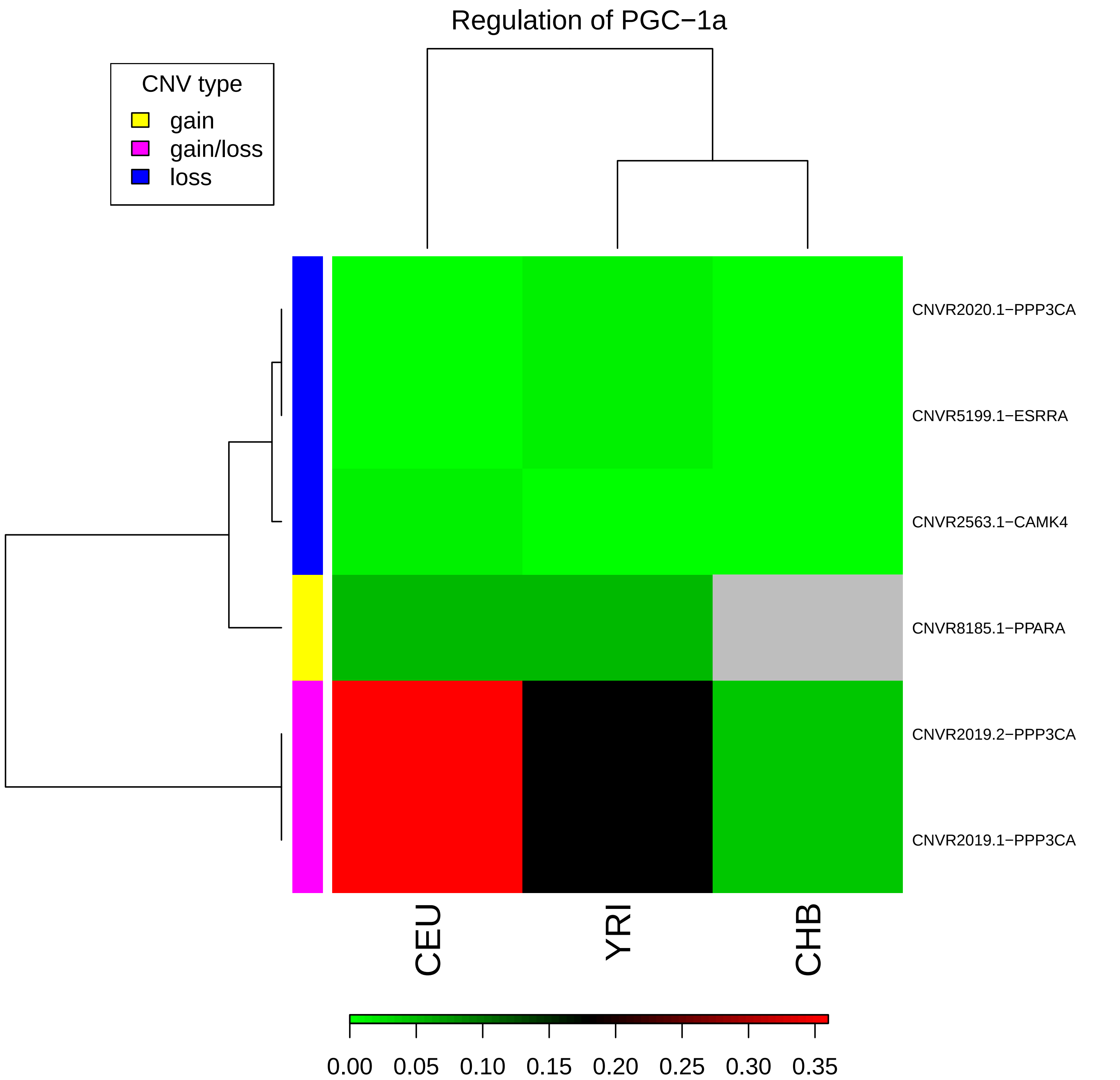

Regulation of PGC−1a
CNV type
gain
gain/loss
loss
CNVR2020.1−PPP3CA
CNVR5199.1−ESRRA
CNVR2563.1−CAMK4
CNVR8185.1−PPARA
CNVR2019.2−PPP3CA
CNVR2019.1−PPP3CA
CEU
YRI
CHB
0.00
0.05
0.10
0.15
0.20
0.25
0.30
0.35

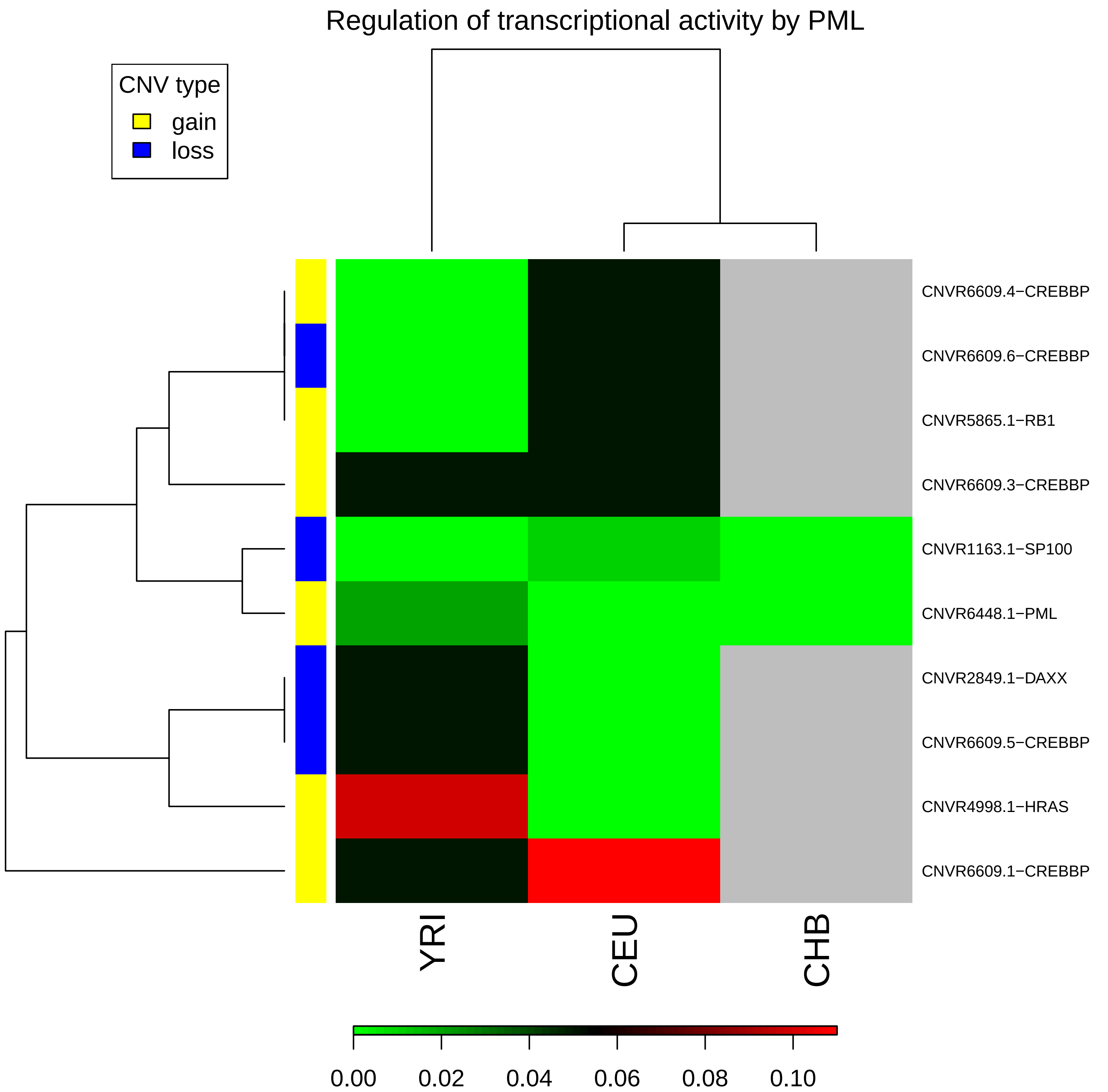
Regulation of transcriptional activity by PML
CNV type
gain
loss
CNVR6609.4−CREBBP
CNVR6609.6−CREBBP
CNVR5865.1−RB1
CNVR6609.3−CREBBP
CNVR1163.1−SP100
CNVR6448.1−PML
CNVR2849.1−DAXX
CNVR6609.5−CREBBP
CNVR4998.1−HRAS
CNVR6609.1−CREBBP
YRI
CEU
CHB
0.00
0.02
0.04
0.06
0.08
0.10

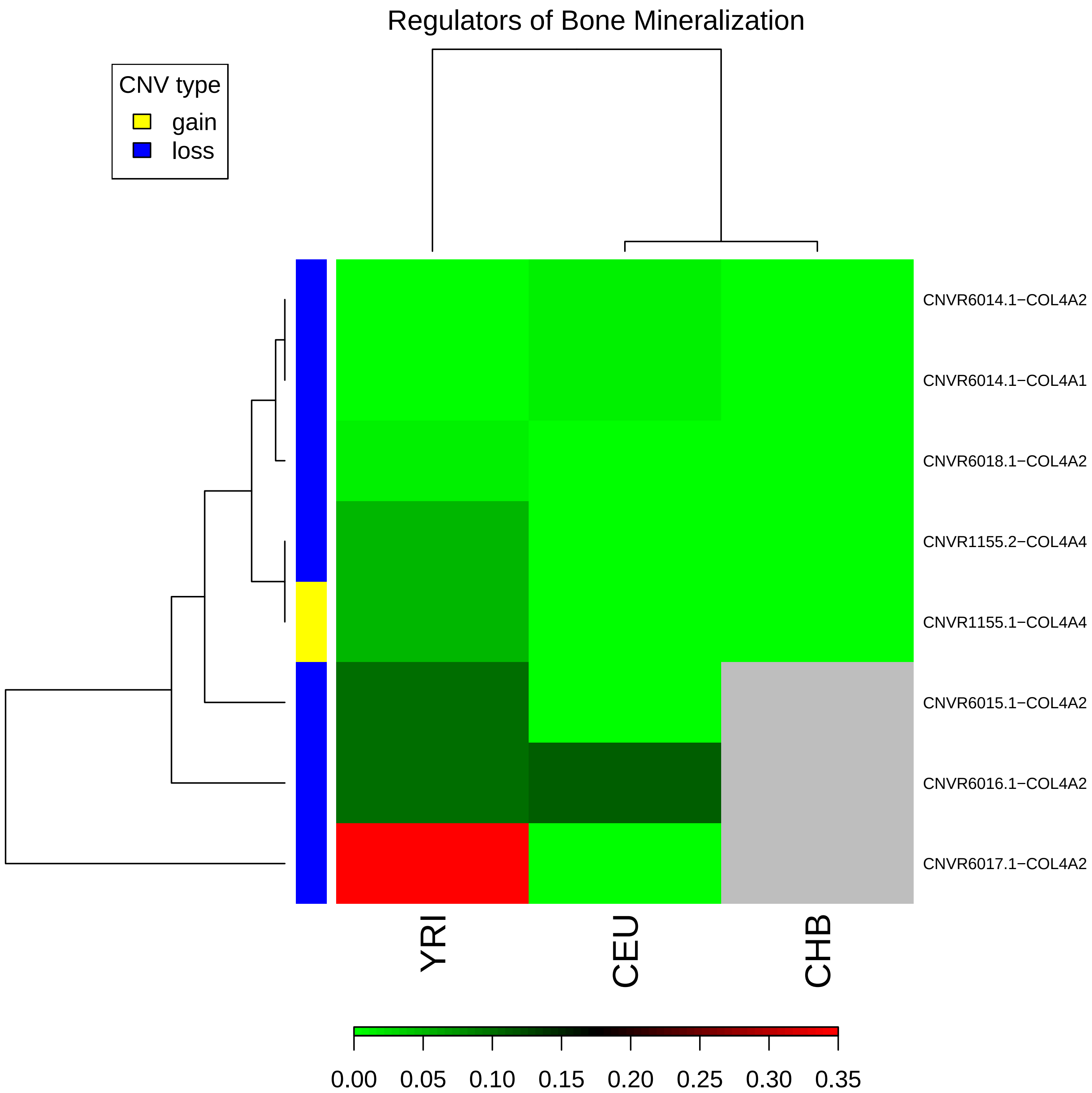
Regulators of Bone Mineralization
CNV type
gain
loss
CNVR6014.1−COL4A2
CNVR6014.1−COL4A1
CNVR6018.1−COL4A2
CNVR1155.2−COL4A4
CNVR1155.1−COL4A4
CNVR6015.1−COL4A2
CNVR6016.1−COL4A2
CNVR6017.1−COL4A2
YRI
CEU
CHB
0.00
0.05
0.10
0.15
0.20
0.25
0.30
0.35

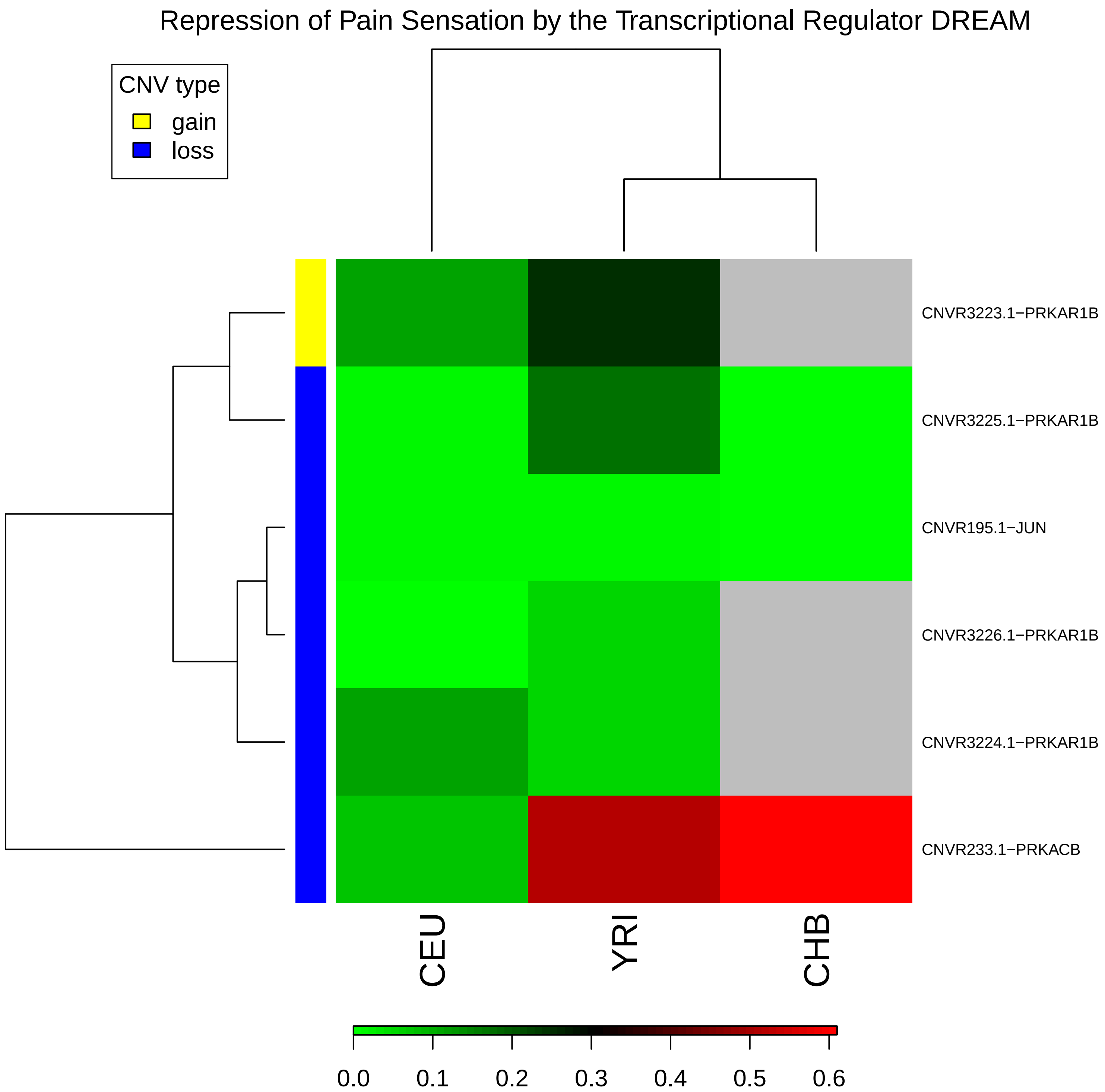

Repression of Pain Sensation by the Transcriptional Regulator DREAM
CNV type
gain
loss
CNVR3223.1−PRKAR1B
CNVR3225.1−PRKAR1B
CNVR195.1−JUN
CNVR3226.1−PRKAR1B
CNVR3224.1−PRKAR1B
CNVR233.1−PRKACB
CEU
YRI
CHB
0.0
0.1
0.2
0.3
0.4
0.5
0.6

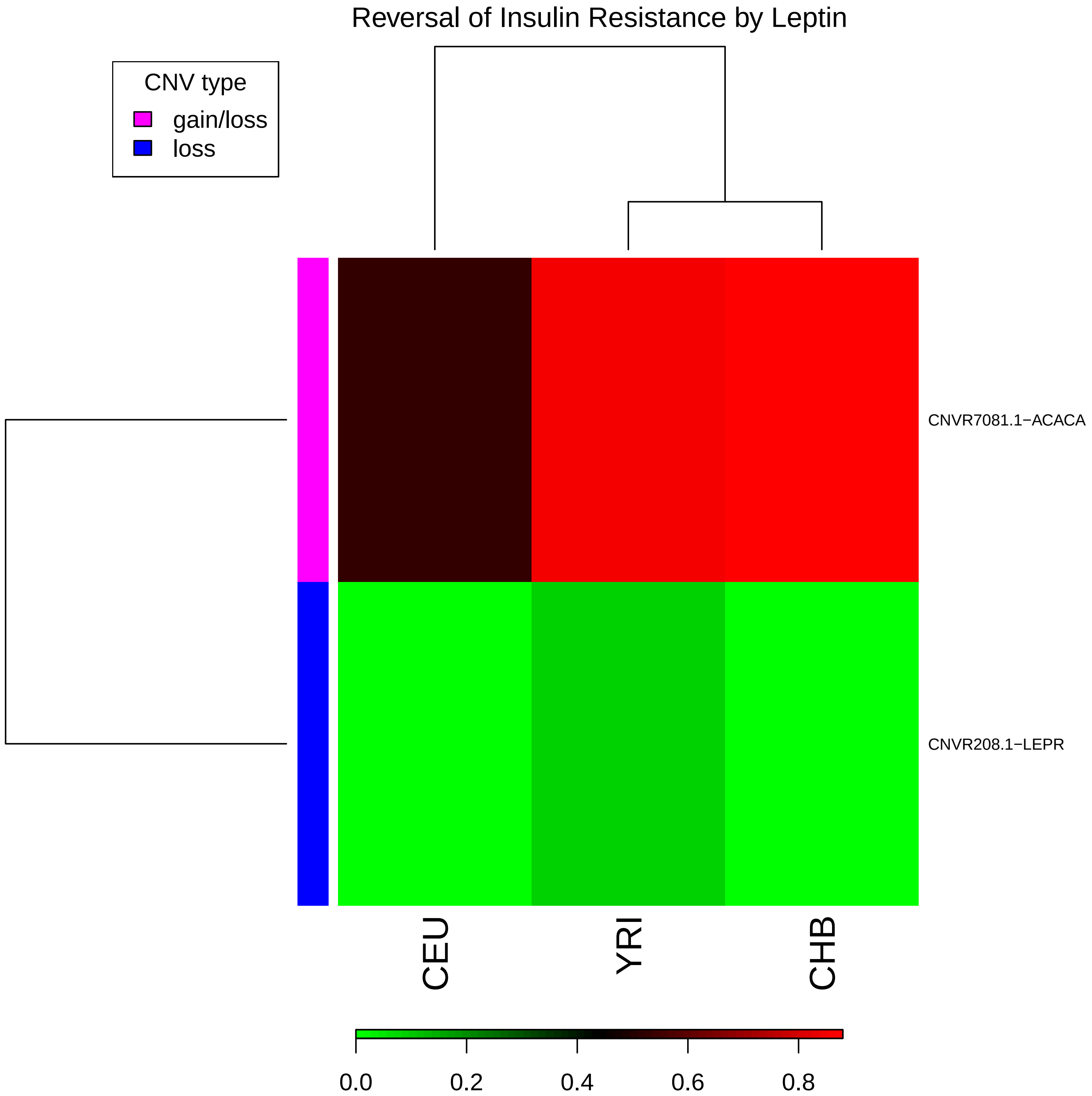
Reversal of Insulin Resistance by Leptin
CNV type
gain/loss
loss
CNVR7081.1−ACACA
CNVR208.1−LEPR
CEU
YRI
CHB
0.0
0.2
0.4
0.6
0.8

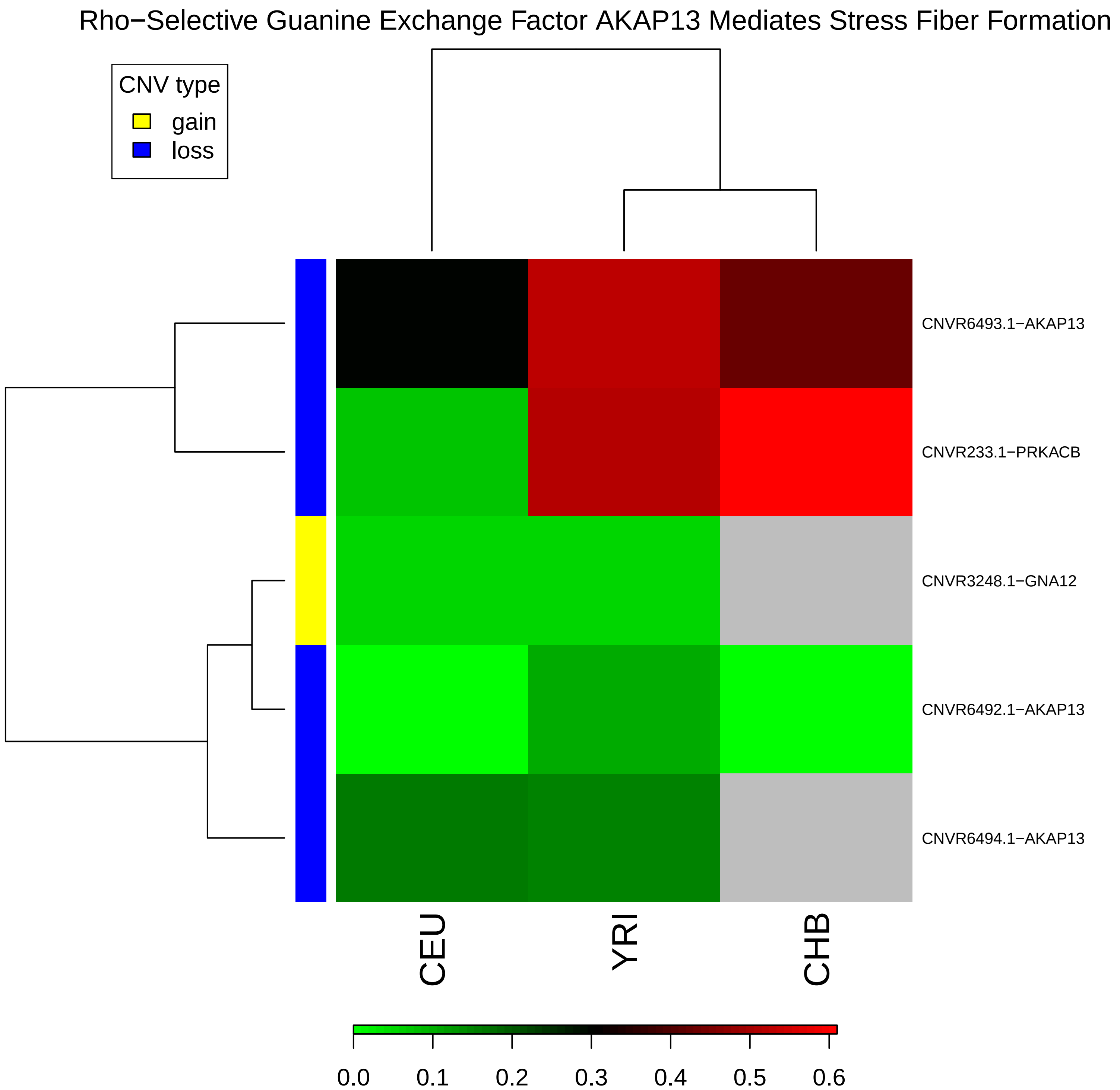
Rho−Selective Guanine Exchange Factor AKAP13 Mediates Stress Fiber Formation
CNV type
gain
loss
CNVR6493.1−AKAP13
CNVR233.1−PRKACB
CNVR3248.1−GNA12
CNVR6492.1−AKAP13
CNVR6494.1−AKAP13
CEU
YRI
CHB
0.0
0.1
0.2
0.3
0.4
0.5
0.6

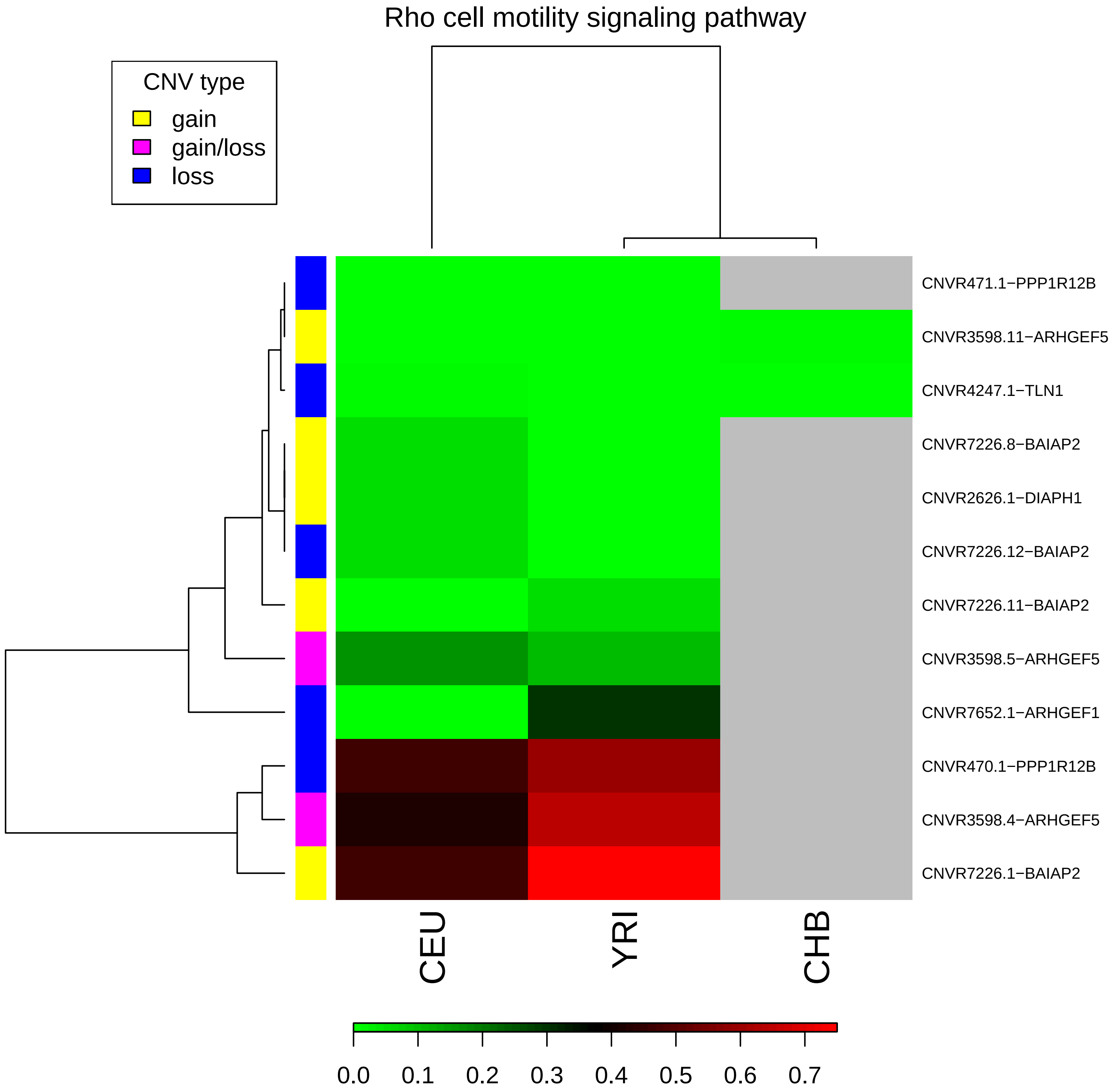

Rho cell motility signaling pathway
CNV type
gain
gain/loss
loss
CNVR471.1−PPP1R12B
CNVR3598.11−ARHGEF5
CNVR4247.1−TLN1
CNVR7226.8−BAIAP2
CNVR2626.1−DIAPH1
CNVR7226.12−BAIAP2
CNVR7226.11−BAIAP2
CNVR3598.5−ARHGEF5
CNVR7652.1−ARHGEF1
CNVR470.1−PPP1R12B
CNVR3598.4−ARHGEF5
CNVR7226.1−BAIAP2
CEU
YRI
CHB
0.0
0.1
0.2
0.3
0.4
0.5
0.6
0.7

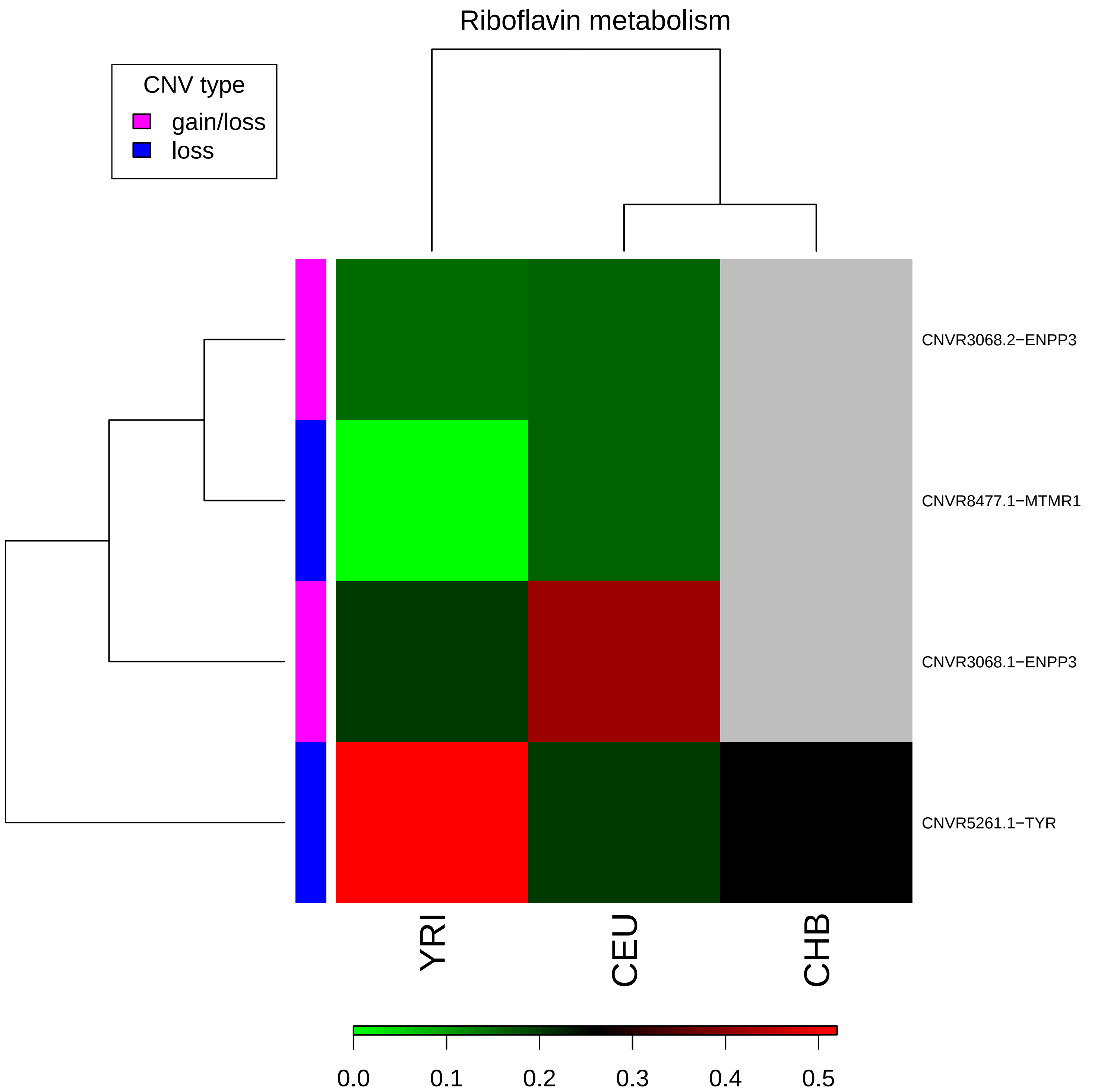
Riboflavin metabolism
CNV type
gain/loss
loss
CNVR3068.2−ENPP3
CNVR8477.1−MTMR1
CNVR3068.1−ENPP3
CNVR5261.1−TYR
YRI
CEU
CHB
0.0
0.1
0.2
0.3
0.4
0.5

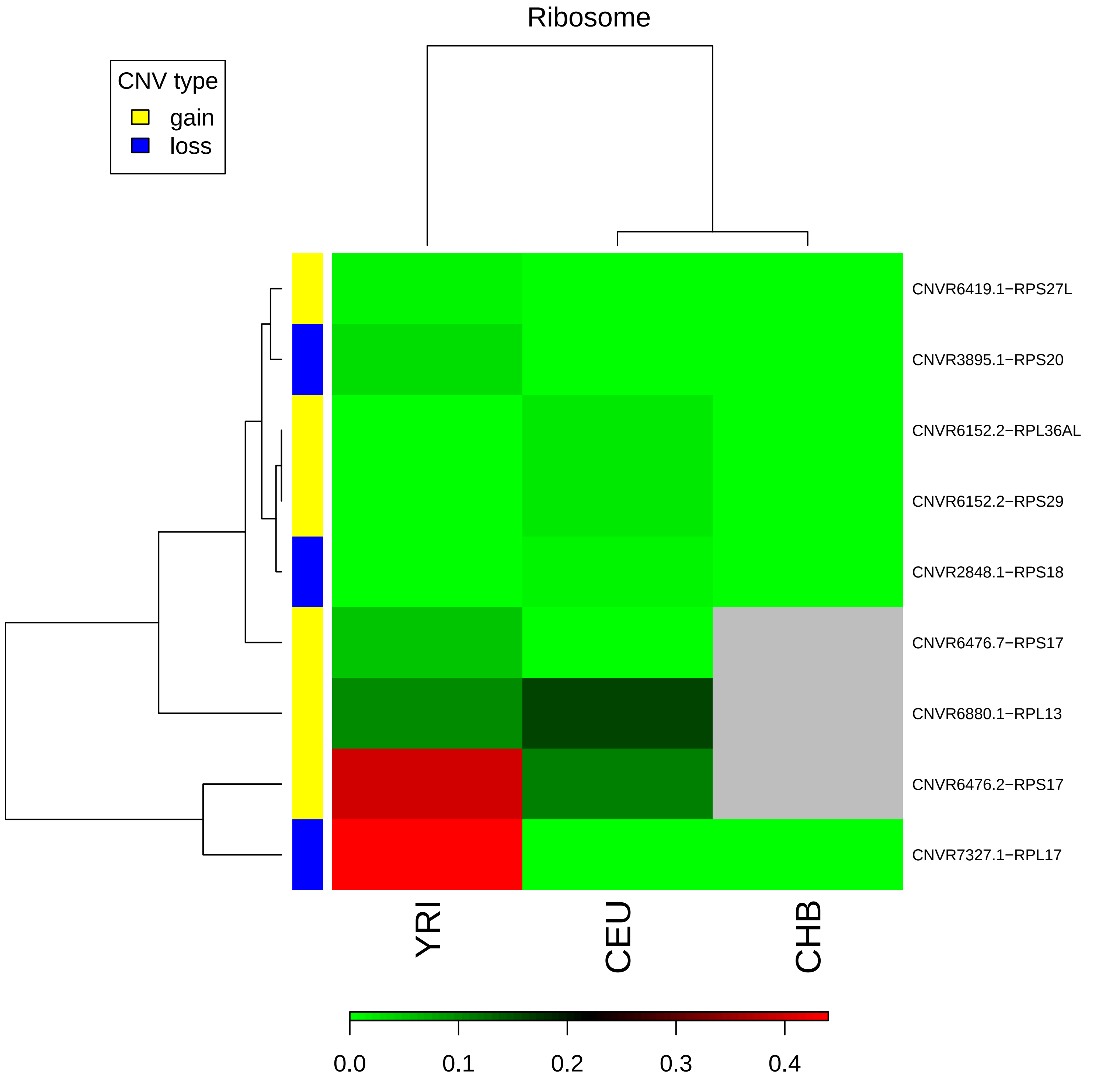

Ribosome
CNV type
gain
loss
CNVR6419.1−RPS27L
CNVR3895.1−RPS20
CNVR6152.2−RPL36AL
CNVR6152.2−RPS29
CNVR2848.1−RPS18
CNVR6476.7−RPS17
CNVR6880.1−RPL13
CNVR6476.2−RPS17
CNVR7327.1−RPL17
YRI
CEU
CHB
0.0
0.1
0.2
0.3
0.4

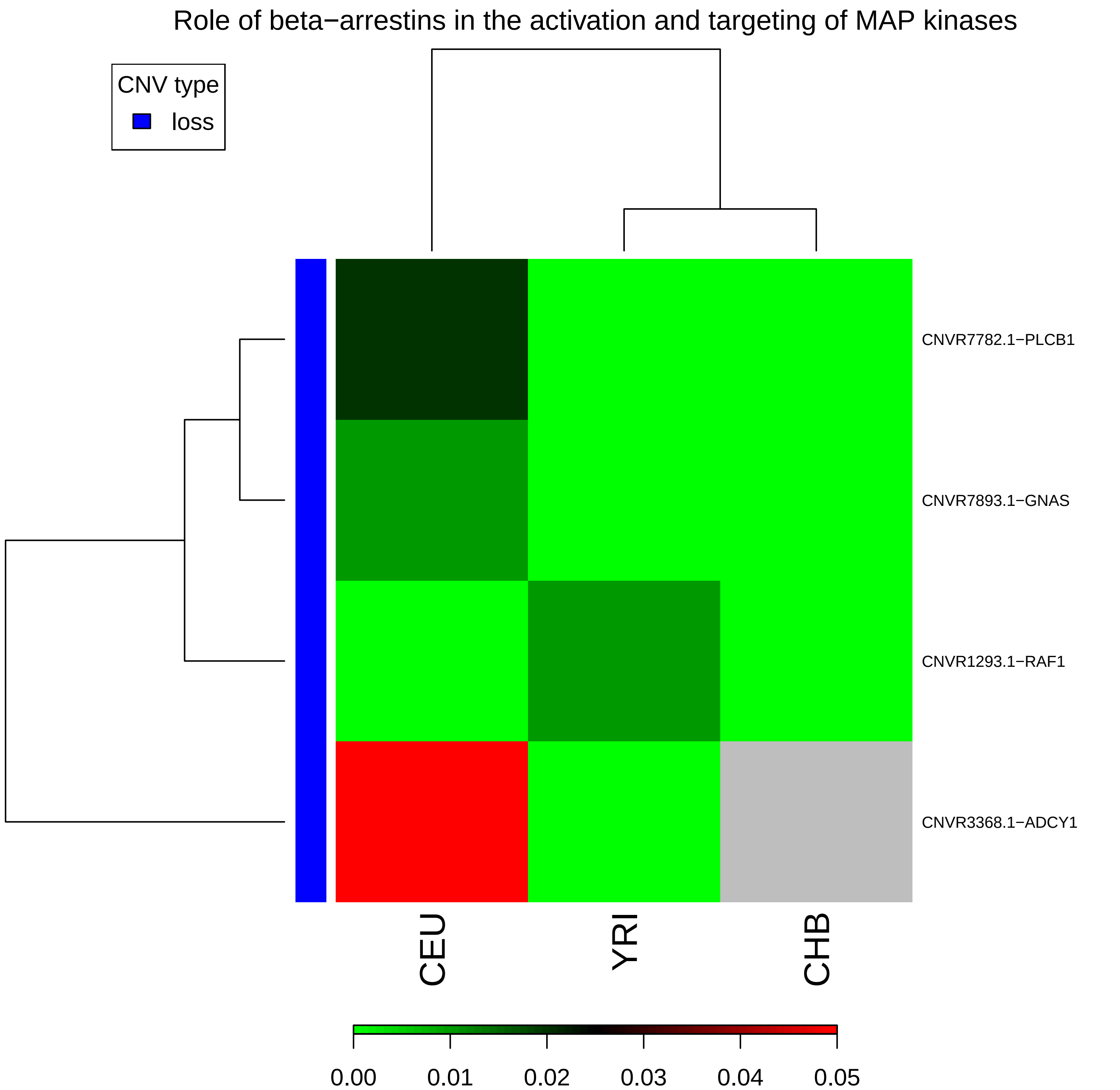

Role of beta−arrestins in the activation and targeting of MAP kinases
CNV type
loss
CNVR7782.1−PLCB1
CNVR7893.1−GNAS
CNVR1293.1−RAF1
CNVR3368.1−ADCY1
CEU
YRI
CHB
0.00
0.01
0.02
0.03
0.04
0.05

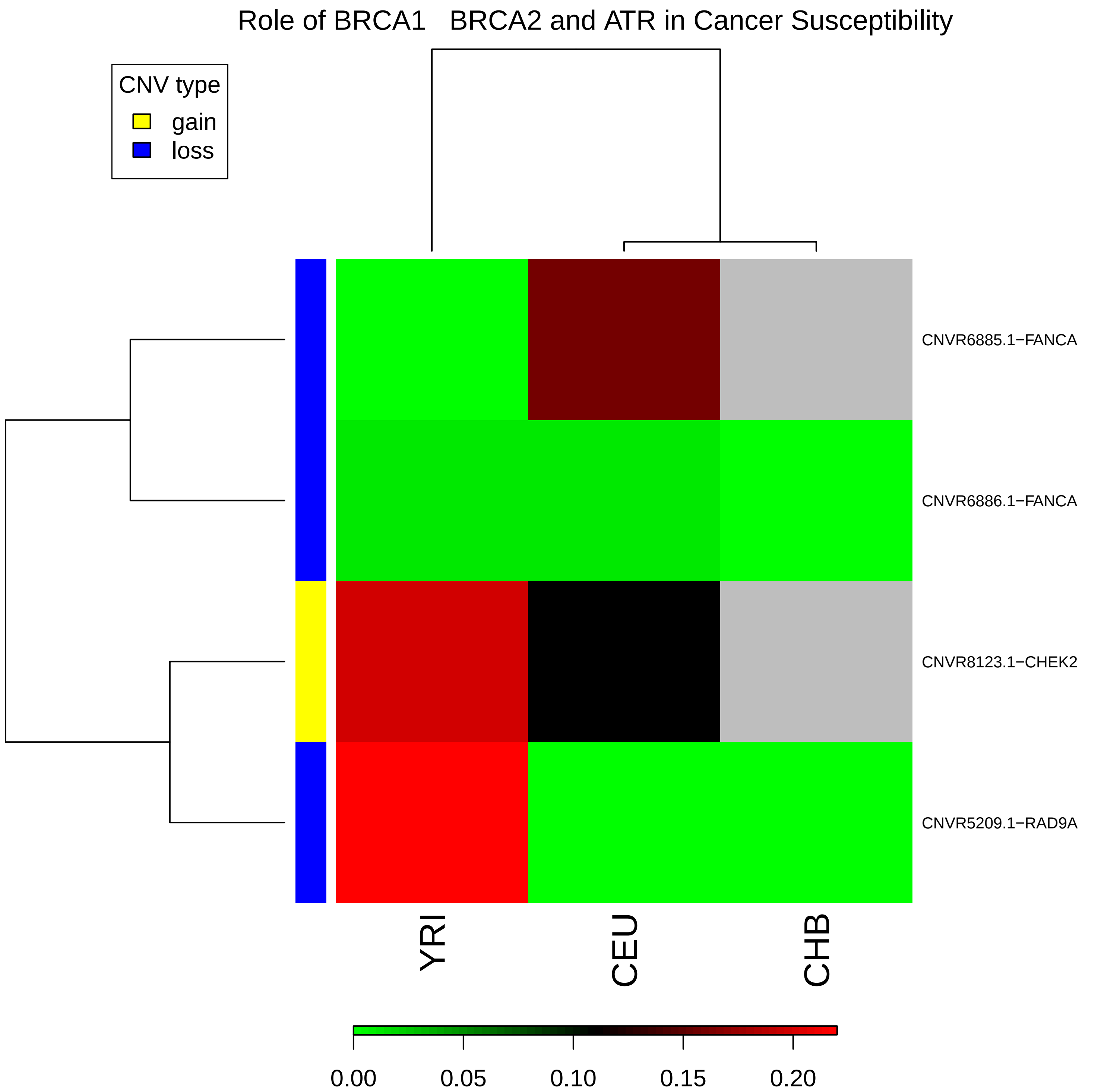

Role of BRCA1 BRCA2 and ATR in Cancer Susceptibility
CNV type
gain
loss
CNVR6885.1−FANCA
CNVR6886.1−FANCA
CNVR8123.1−CHEK2
CNVR5209.1−RAD9A
YRI
CEU
CHB
0.00
0.05
0.10
0.15
0.20

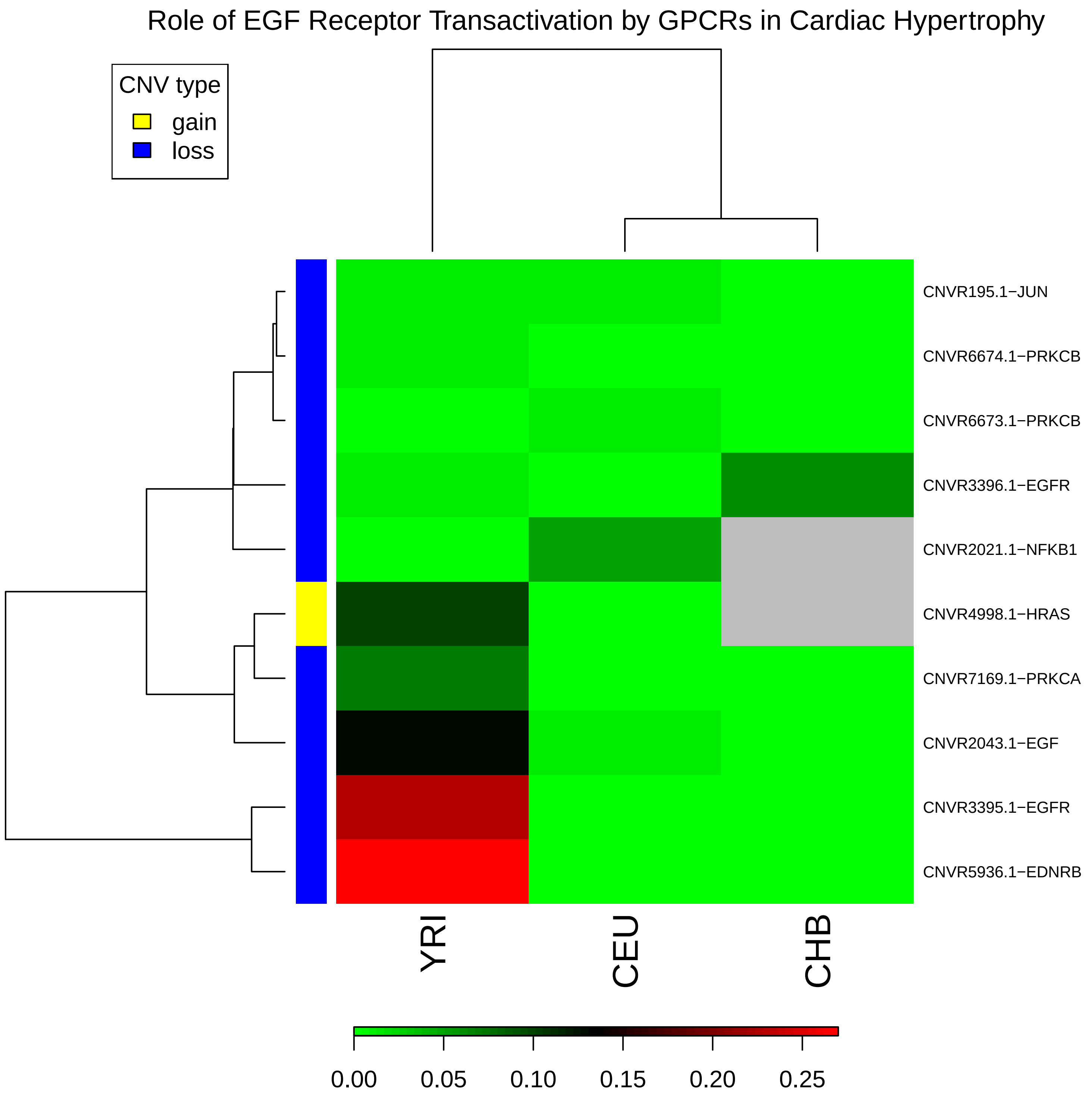
Role of EGF Receptor Transactivation by GPCRs in Cardiac Hypertrophy
CNV type
gain
loss
CNVR195.1−JUN
CNVR6674.1−PRKCB
CNVR6673.1−PRKCB
CNVR3396.1−EGFR
CNVR2021.1−NFKB1
CNVR4998.1−HRAS
CNVR7169.1−PRKCA
CNVR2043.1−EGF
CNVR3395.1−EGFR
CNVR5936.1−EDNRB
YRI
CEU
CHB
0.00
0.05
0.10
0.15
0.20
0.25

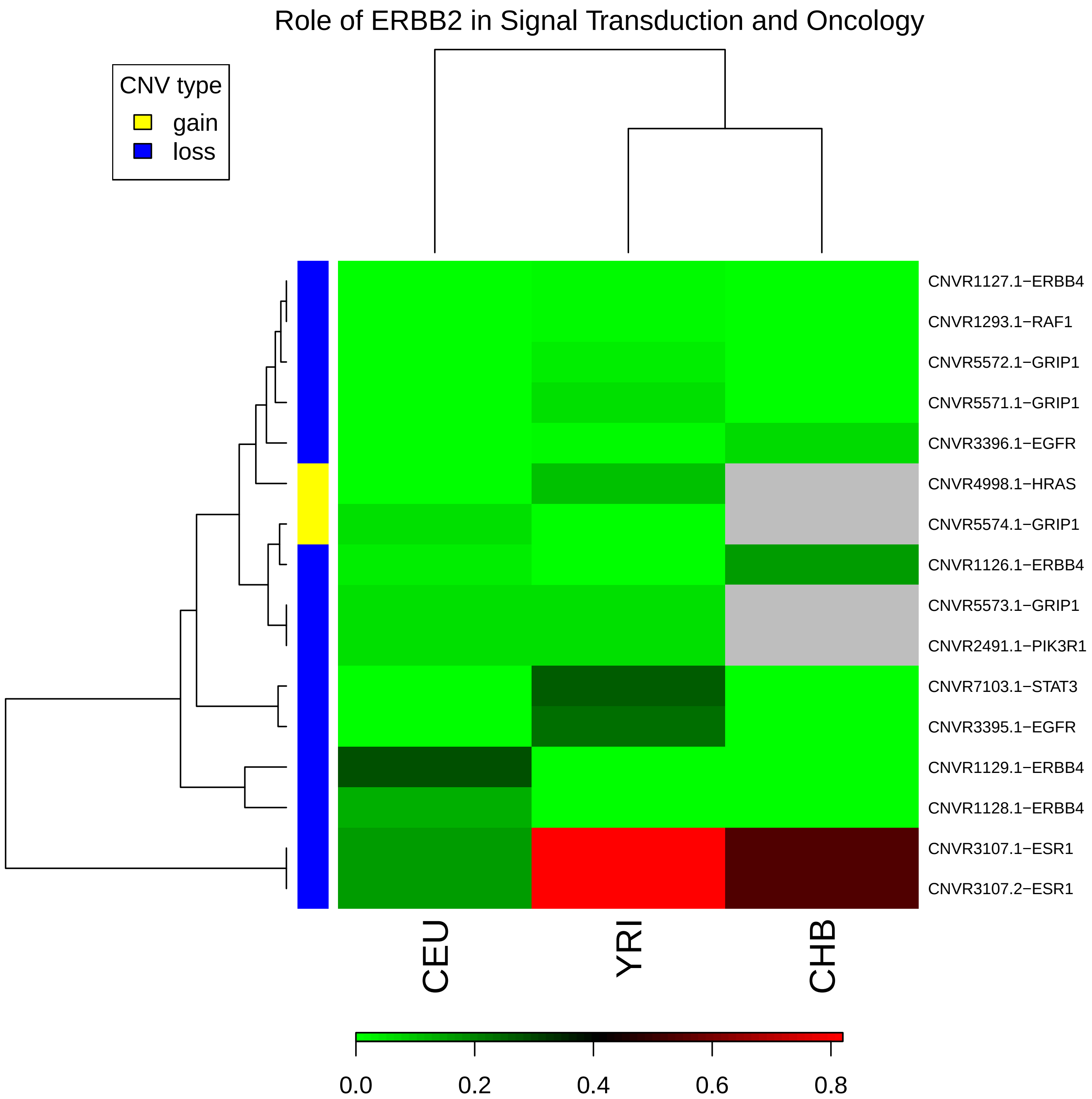
Role of ERBB2 in Signal Transduction and Oncology
CNV type
gain
loss
CNVR1127.1−ERBB4
CNVR1293.1−RAF1
CNVR5572.1−GRIP1
CNVR5571.1−GRIP1
CNVR3396.1−EGFR
CNVR4998.1−HRAS
CNVR5574.1−GRIP1
CNVR1126.1−ERBB4
CNVR5573.1−GRIP1
CNVR2491.1−PIK3R1
CNVR7103.1−STAT3
CNVR3395.1−EGFR
CNVR1129.1−ERBB4
CNVR1128.1−ERBB4
CNVR3107.1−ESR1
CNVR3107.2−ESR1
CEU
YRI
CHB
0.0
0.2
0.4
0.6
0.8

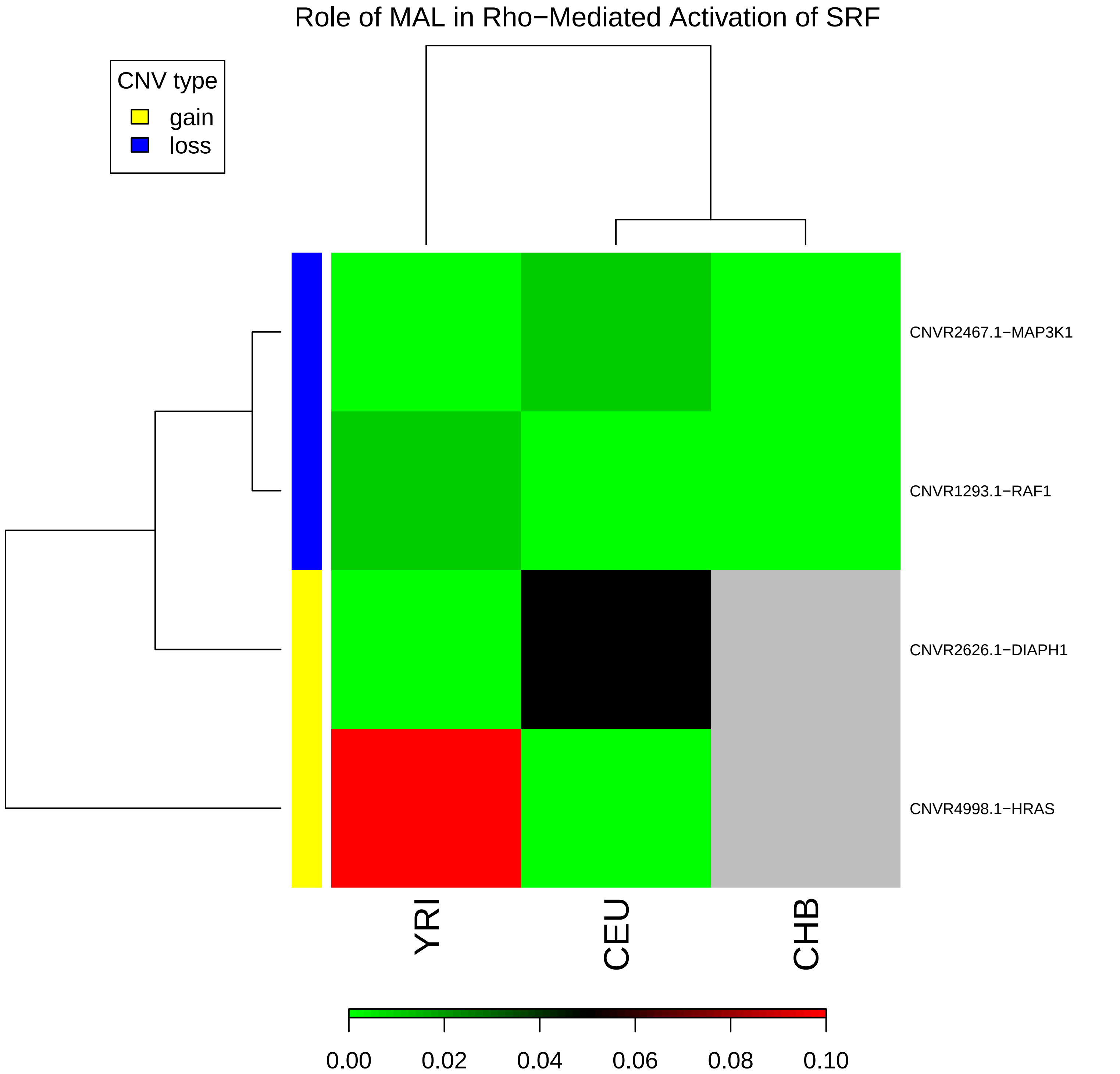

Role of MAL in Rho−Mediated Activation of SRF
CNV type
gain
loss
CNVR2467.1−MAP3K1
CNVR1293.1−RAF1
CNVR2626.1−DIAPH1
CNVR4998.1−HRAS
YRI
CEU
CHB
0.00
0.02
0.04
0.06
0.08
0.10

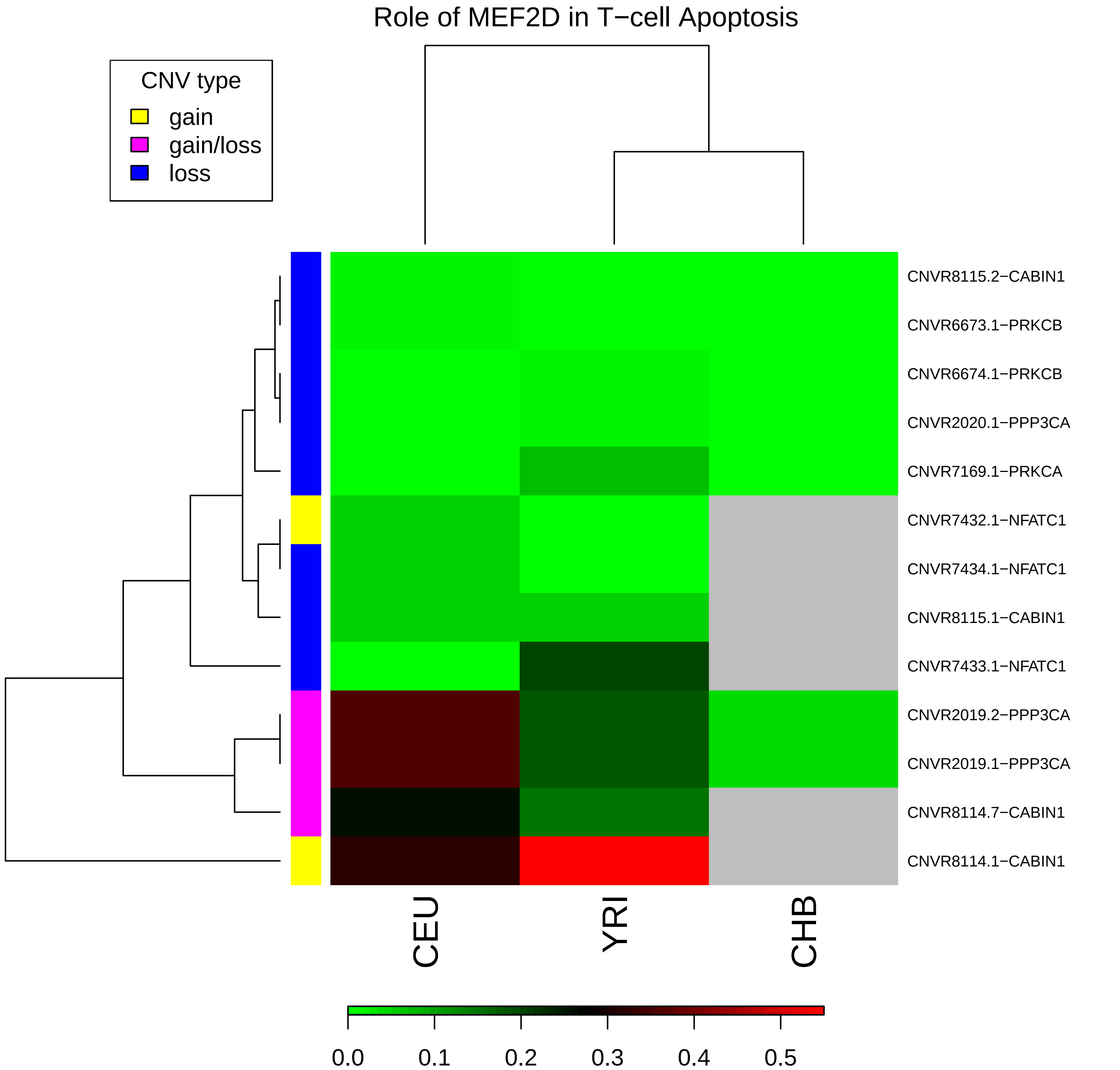

Role of MEF2D in T−cell Apoptosis
CNV type
gain
gain/loss
loss
CNVR8115.2−CABIN1
CNVR6673.1−PRKCB
CNVR6674.1−PRKCB
CNVR2020.1−PPP3CA
CNVR7169.1−PRKCA
CNVR7432.1−NFATC1
CNVR7434.1−NFATC1
CNVR8115.1−CABIN1
CNVR7433.1−NFATC1
CNVR2019.2−PPP3CA
CNVR2019.1−PPP3CA
CNVR8114.7−CABIN1
CNVR8114.1−CABIN1
CEU
YRI
CHB
0.0
0.1
0.2
0.3
0.4
0.5

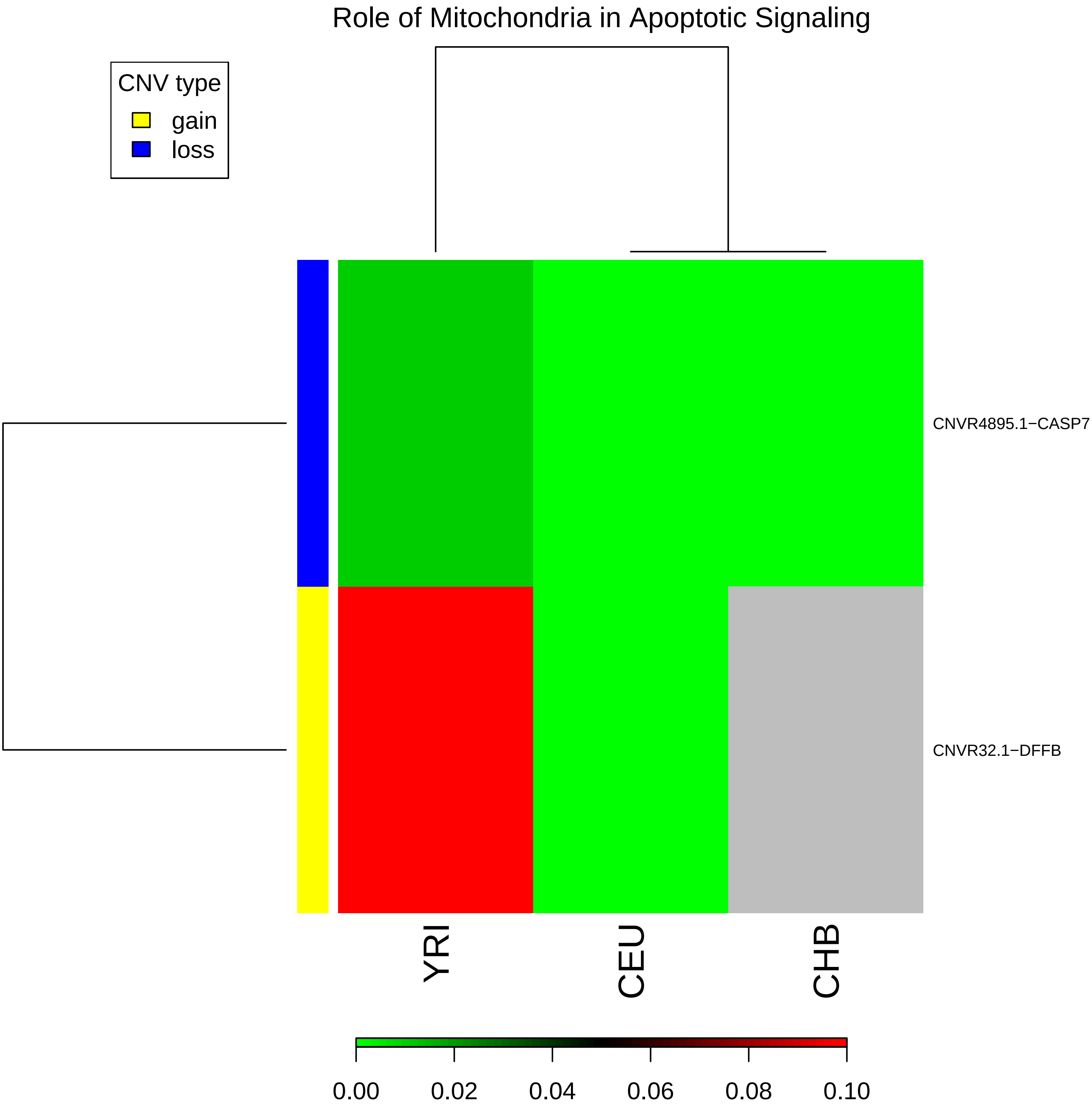
Role of Mitochondria in Apoptotic Signaling
CNV type
gain
loss
CNVR4895.1−CASP7
CNVR32.1−DFFB
YRI
CEU
CHB
0.00
0.02
0.04
0.06
0.08
0.10

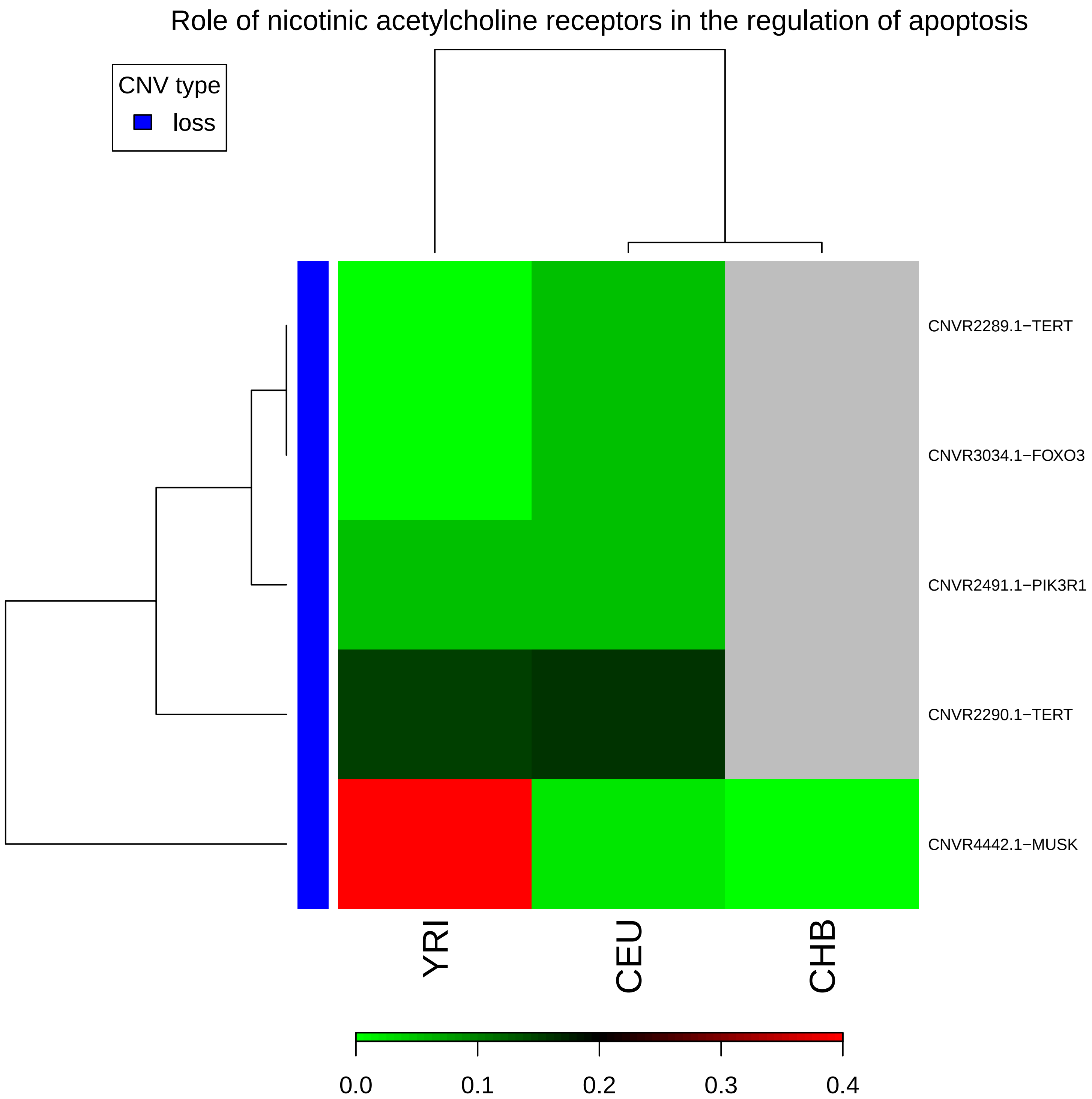

Role of nicotinic acetylcholine receptors in the regulation of apoptosis
CNV type
loss
CNVR2289.1−TERT
CNVR3034.1−FOXO3
CNVR2491.1−PIK3R1
CNVR2290.1−TERT
CNVR4442.1−MUSK
YRI
CEU
CHB
0.0
0.1
0.2
0.3
0.4

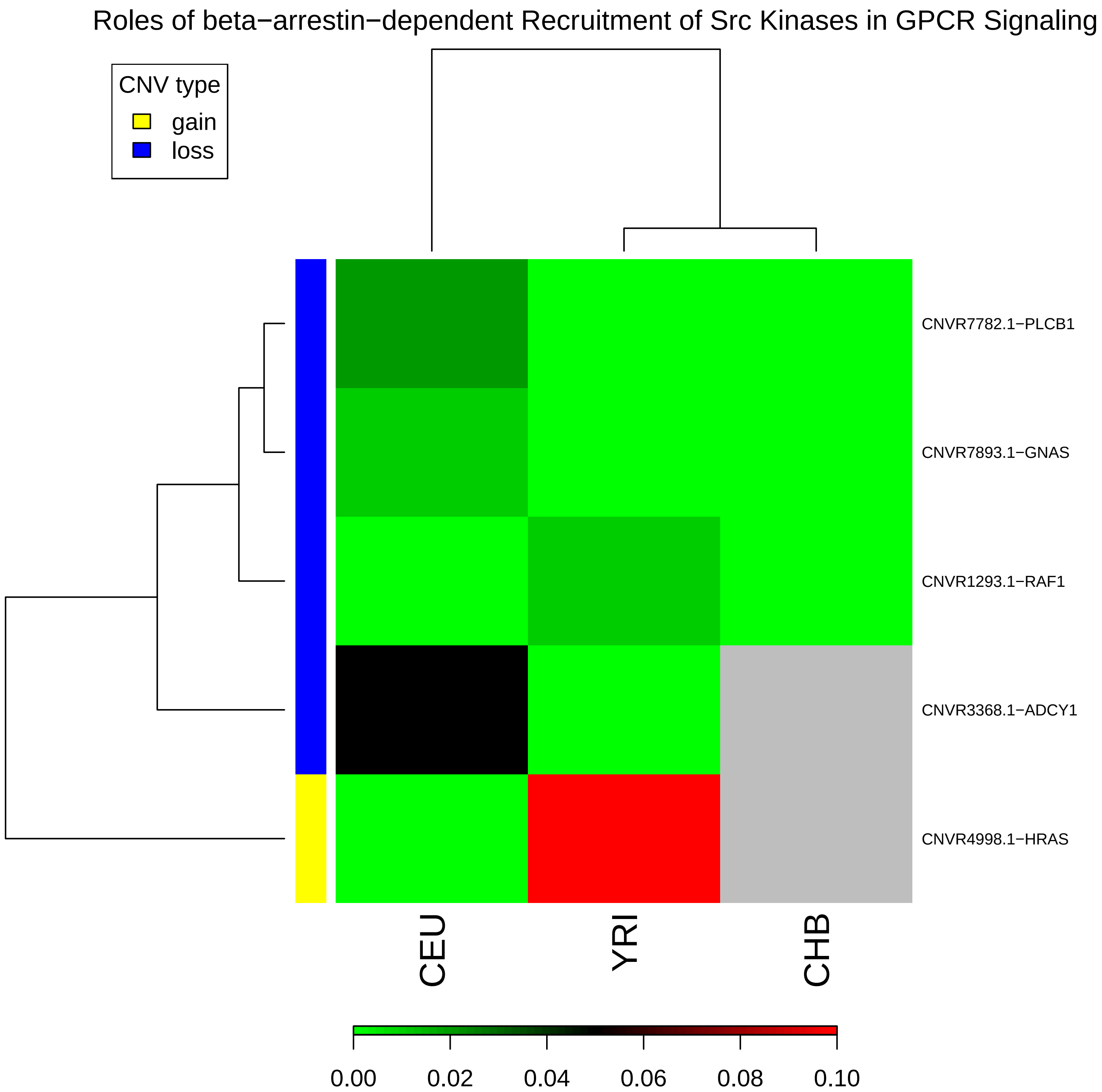

Roles of beta−arrestin−dependent Recruitment of Src Kinases in GPCR Signaling
CNV type
gain
loss
CNVR7782.1−PLCB1
CNVR7893.1−GNAS
CNVR1293.1−RAF1
CNVR3368.1−ADCY1
CNVR4998.1−HRAS
CEU
YRI
CHB
0.00
0.02
0.04
0.06
0.08
0.10

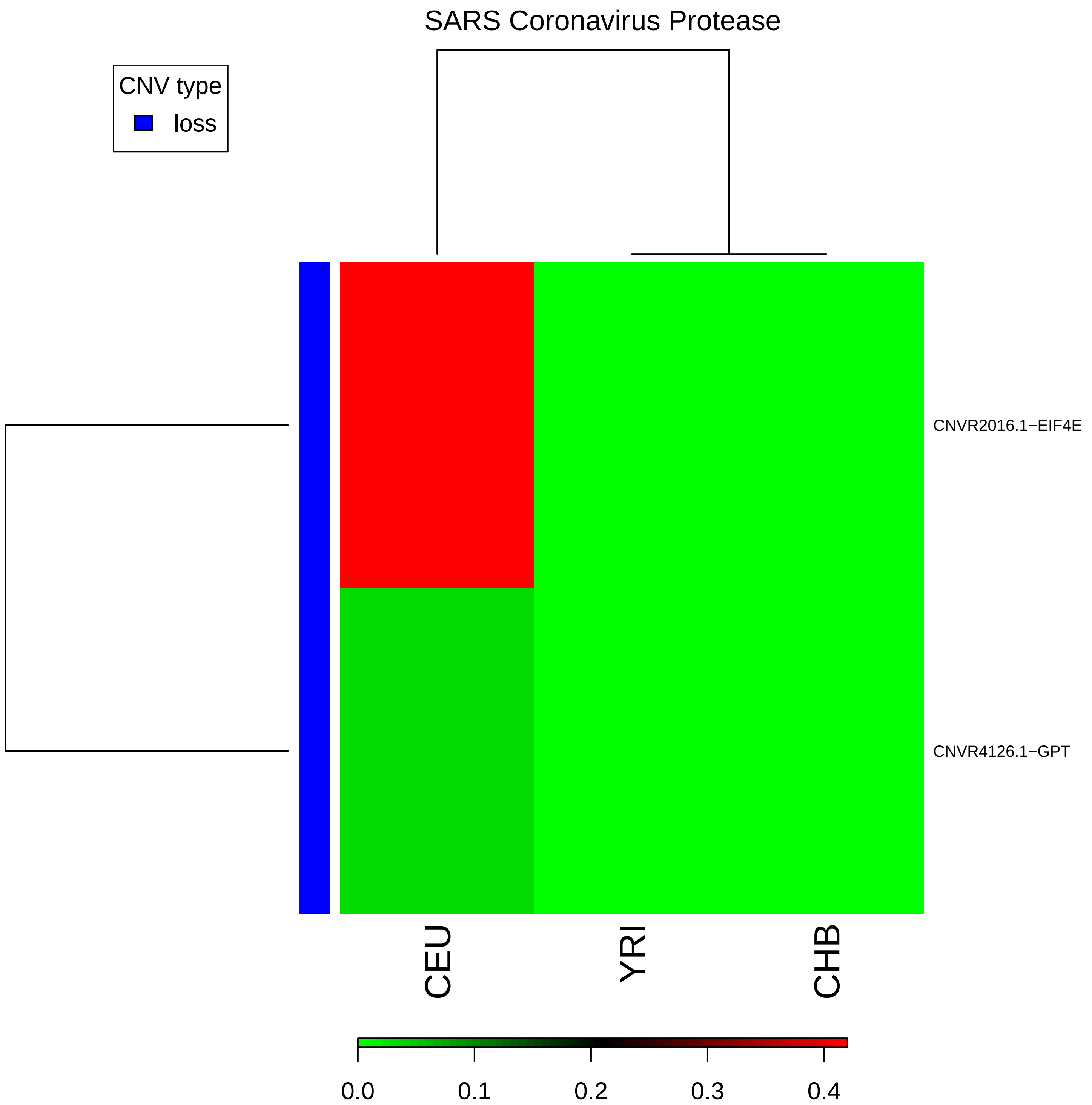
SARS Coronavirus Protease
CNV type
loss
CNVR2016.1−EIF4E
CNVR4126.1−GPT
CEU
YRI
CHB
0.0
0.1
0.2
0.3
0.4

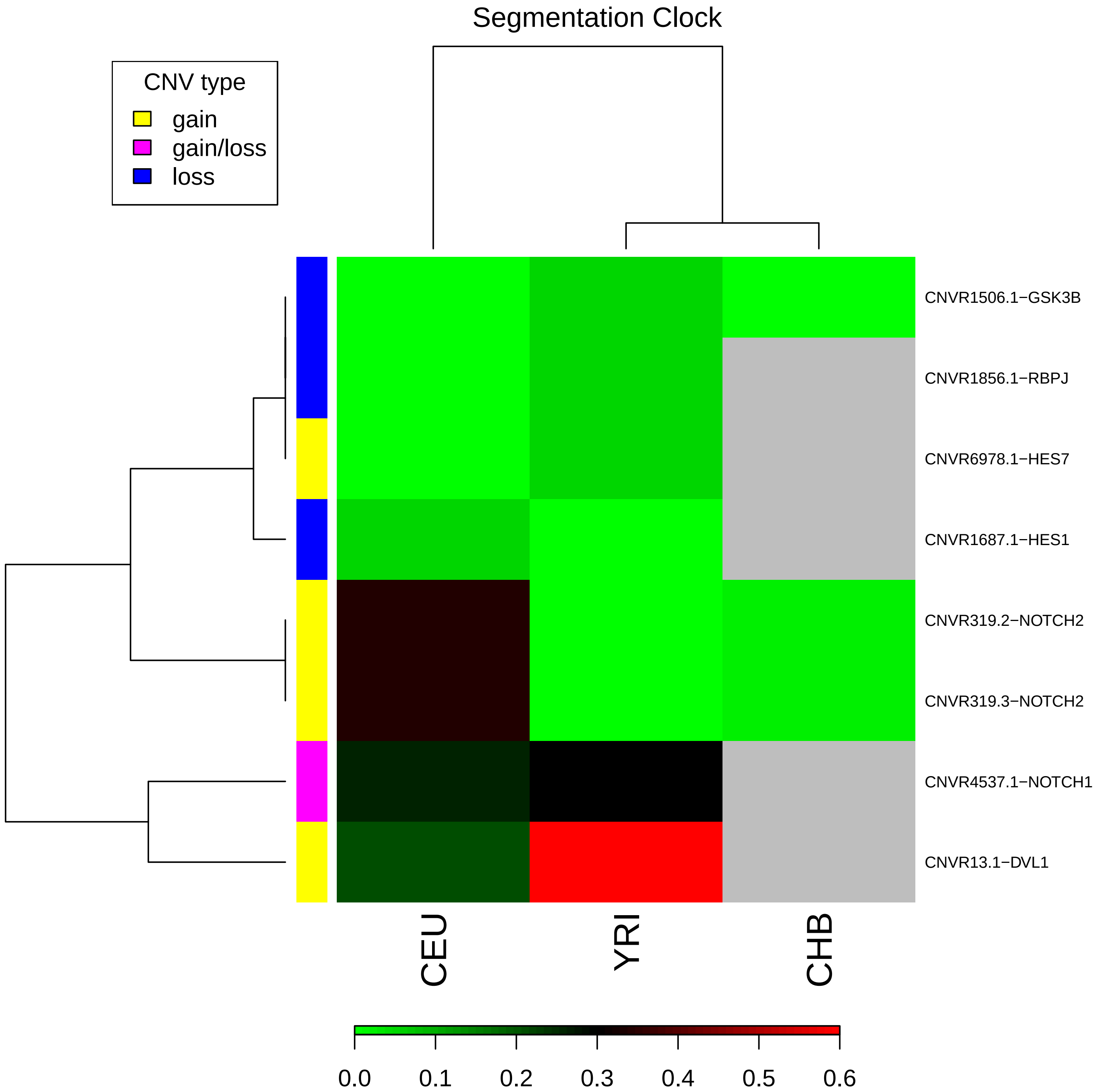
Segmentation Clock
CNV type
gain
gain/loss
loss
CNVR1506.1−GSK3B
CNVR1856.1−RBPJ
CNVR6978.1−HES7
CNVR1687.1−HES1
CNVR319.2−NOTCH2
CNVR319.3−NOTCH2
CNVR4537.1−NOTCH1
CNVR13.1−DVL1
CEU
YRI
CHB
0.0
0.1
0.2
0.3
0.4
0.5
0.6

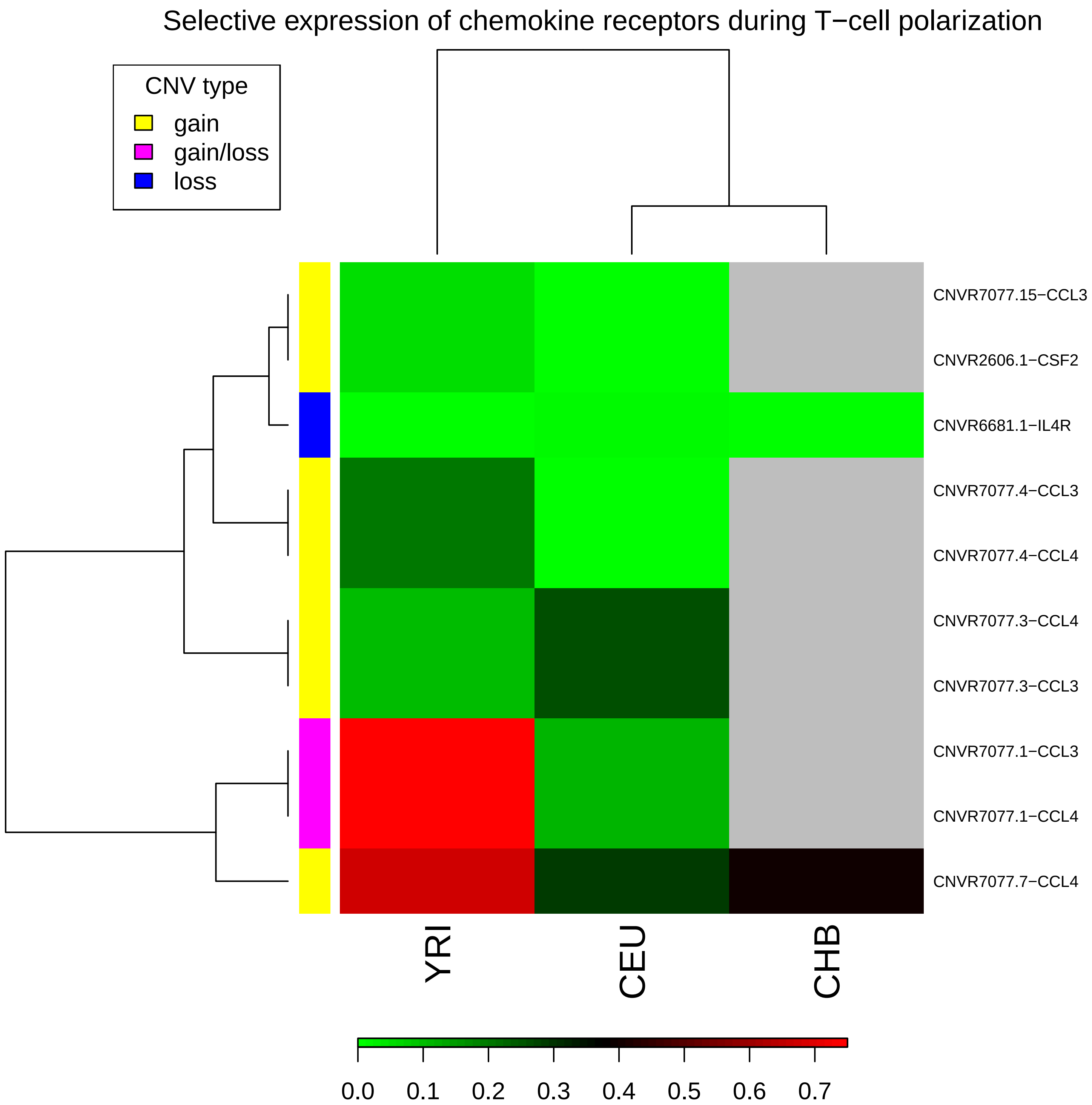

Selective expression of chemokine receptors during T−cell polarization
CNV type
gain
gain/loss
loss
CNVR7077.15−CCL3
CNVR2606.1−CSF2
CNVR6681.1−IL4R
CNVR7077.4−CCL3
CNVR7077.4−CCL4
CNVR7077.3−CCL4
CNVR7077.3−CCL3
CNVR7077.1−CCL3
CNVR7077.1−CCL4
CNVR7077.7−CCL4
YRI
CEU
CHB
0.0
0.1
0.2
0.3
0.4
0.5
0.6
0.7

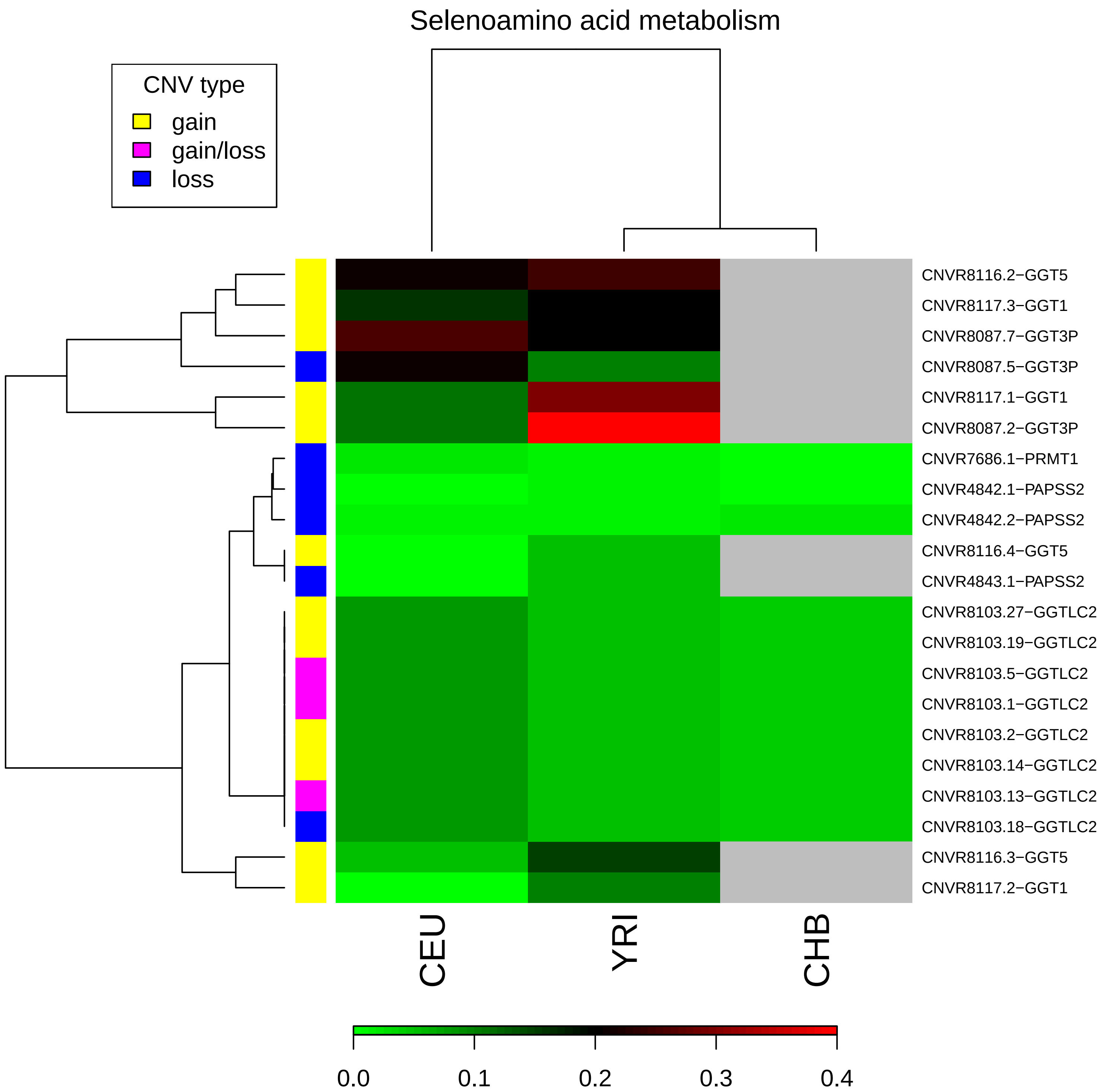

Selenoamino acid metabolism
CNV type
gain
gain/loss
loss
CNVR8116.2−GGT5
CNVR8117.3−GGT1
CNVR8087.7−GGT3P
CNVR8087.5−GGT3P
CNVR8117.1−GGT1
CNVR8087.2−GGT3P
CNVR7686.1−PRMT1
CNVR4842.1−PAPSS2
CNVR4842.2−PAPSS2
CNVR8116.4−GGT5
CNVR4843.1−PAPSS2
CNVR8103.27−GGTLC2
CNVR8103.19−GGTLC2
CNVR8103.5−GGTLC2
CNVR8103.1−GGTLC2
CNVR8103.2−GGTLC2
CNVR8103.14−GGTLC2
CNVR8103.13−GGTLC2
CNVR8103.18−GGTLC2
CNVR8116.3−GGT5
CNVR8117.2−GGT1
CEU
YRI
CHB
0.0
0.1
0.2
0.3
0.4

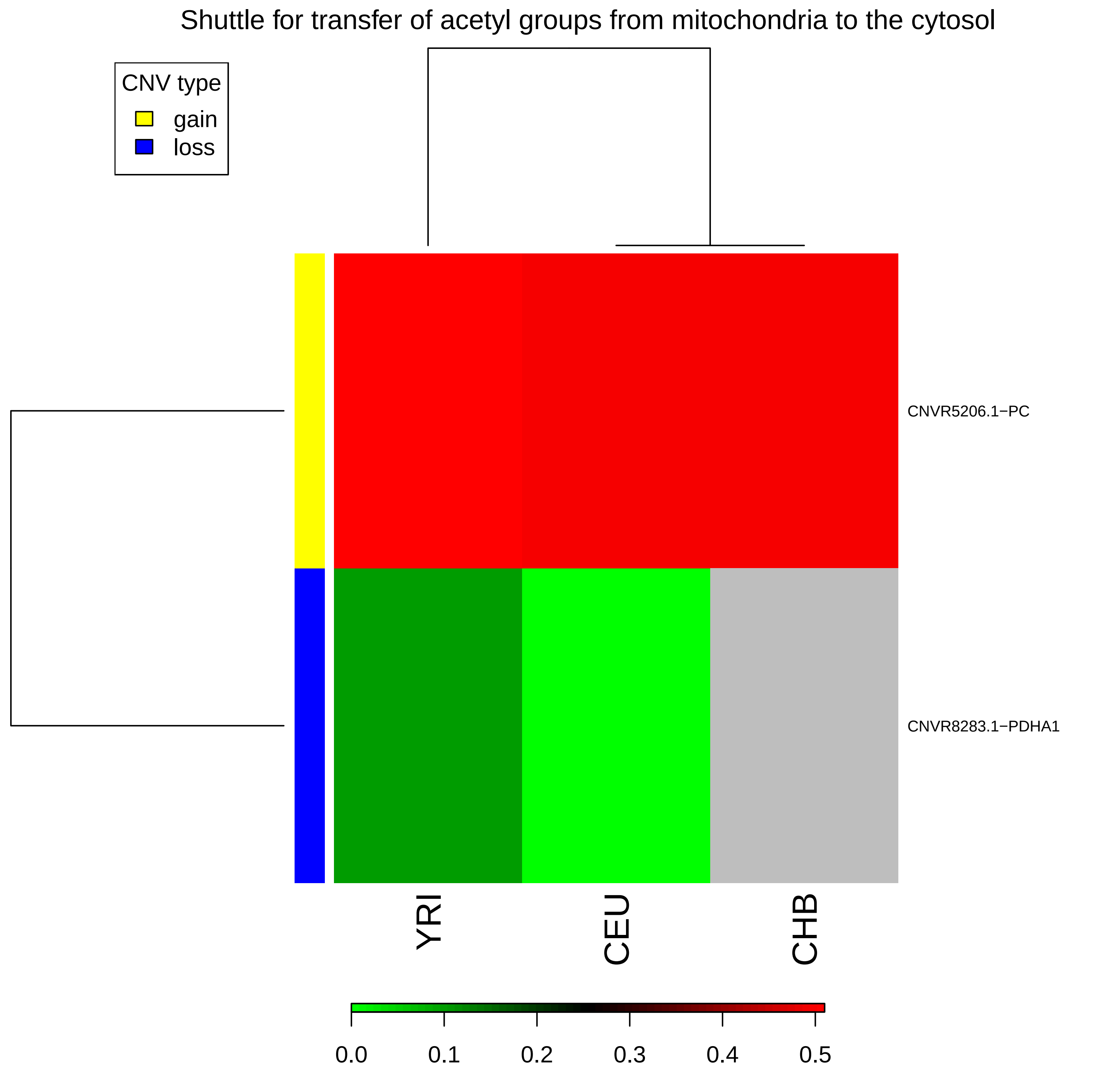

Shuttle for transfer of acetyl groups from mitochondria to the cytosol
CNV type
gain
loss
CNVR5206.1−PC
CNVR8283.1−PDHA1
YRI
CEU
CHB
0.0
0.1
0.2
0.3
0.4
0.5

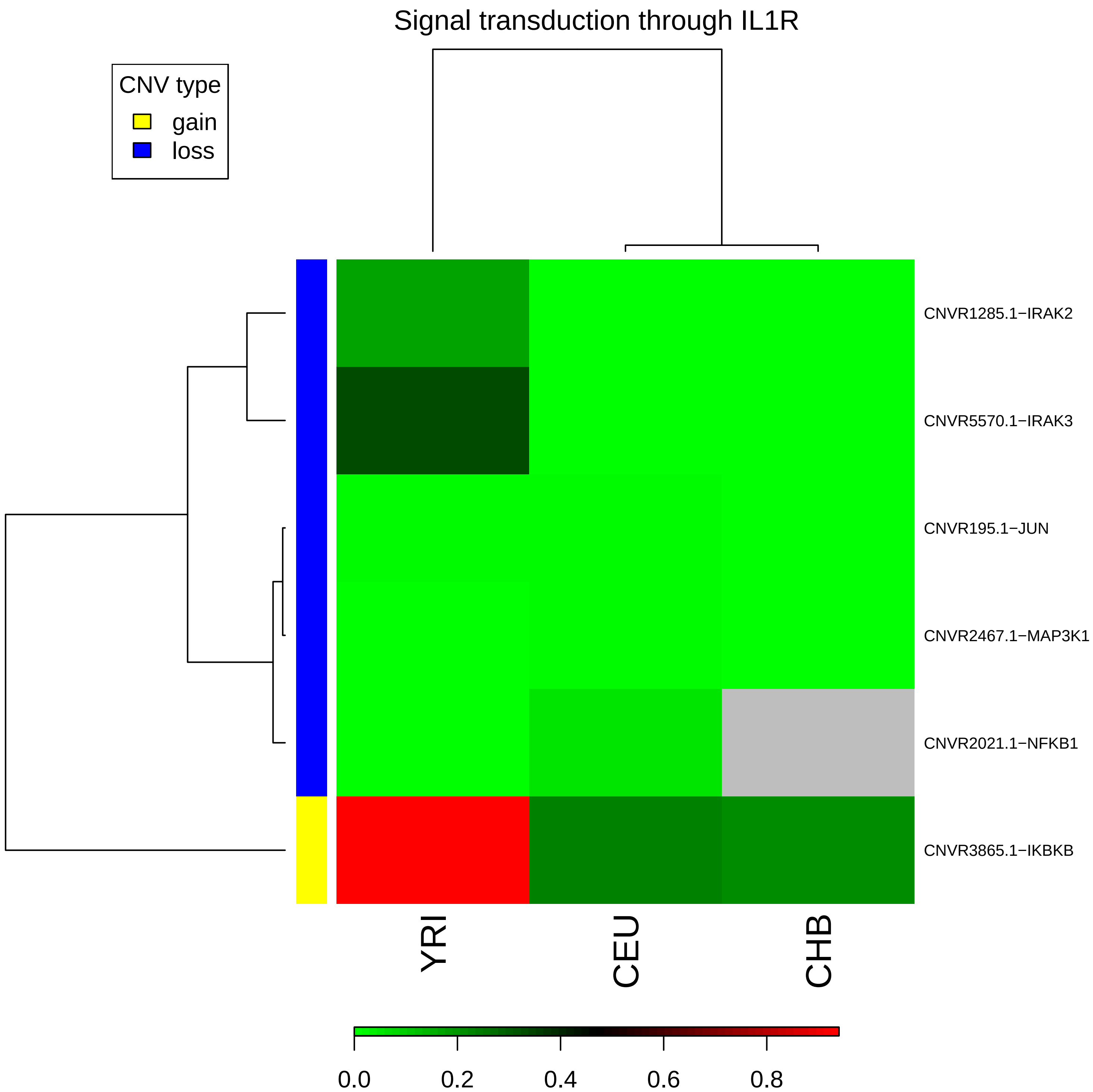
Signal transduction through IL1R
CNV type
gain
loss
CNVR1285.1−IRAK2
CNVR5570.1−IRAK3
CNVR195.1−JUN
CNVR2467.1−MAP3K1
CNVR2021.1−NFKB1
CNVR3865.1−IKBKB
YRI
CEU
CHB
0.0
0.2
0.4
0.6
0.8

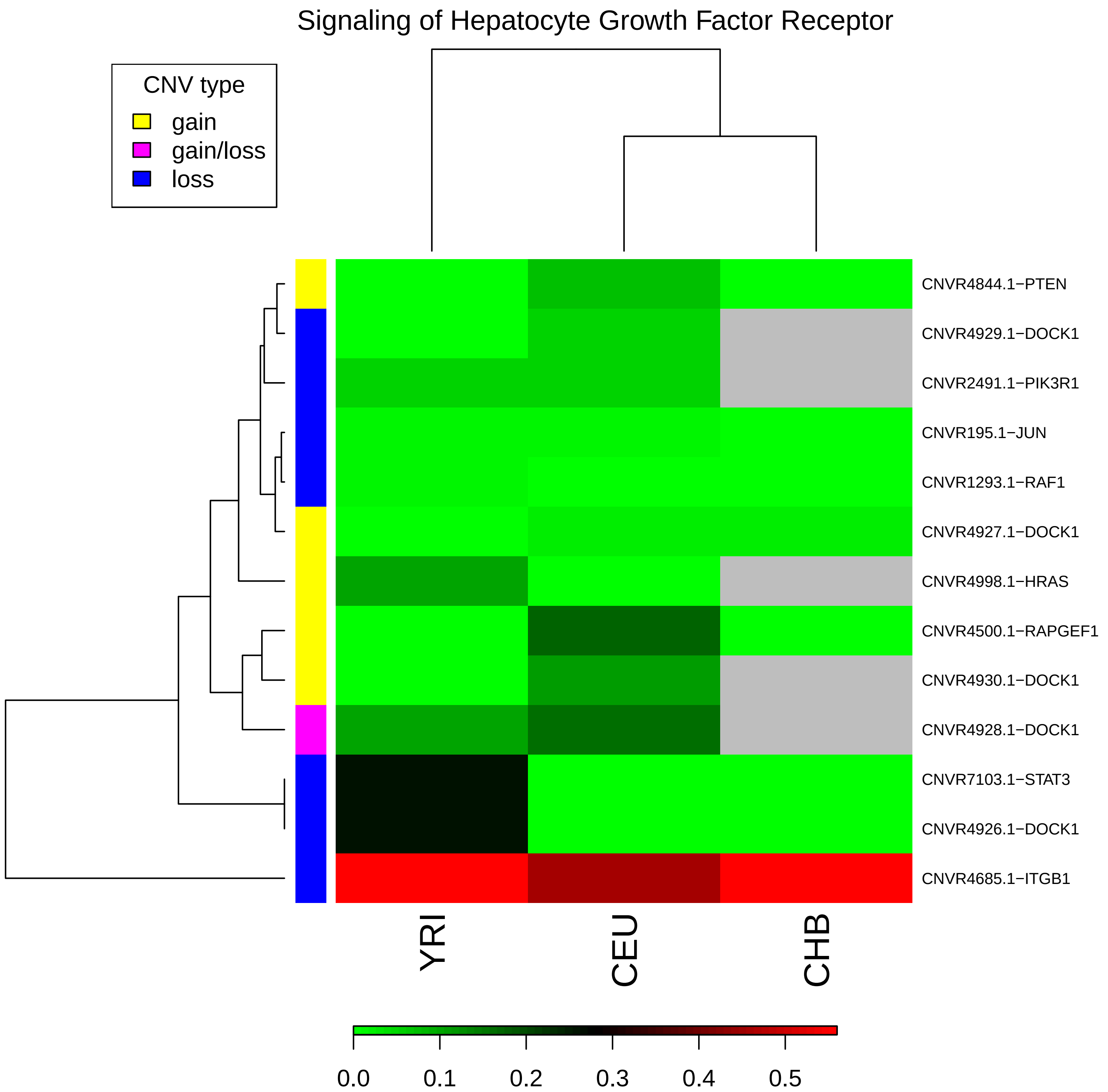
Signaling of Hepatocyte Growth Factor Receptor
CNV type
gain
gain/loss
loss
CNVR4844.1−PTEN
CNVR4929.1−DOCK1
CNVR2491.1−PIK3R1
CNVR195.1−JUN
CNVR1293.1−RAF1
CNVR4927.1−DOCK1
CNVR4998.1−HRAS
CNVR4500.1−RAPGEF1
CNVR4930.1−DOCK1
CNVR4928.1−DOCK1
CNVR7103.1−STAT3
CNVR4926.1−DOCK1
CNVR4685.1−ITGB1
YRI
CEU
CHB
0.0
0.1
0.2
0.3
0.4
0.5

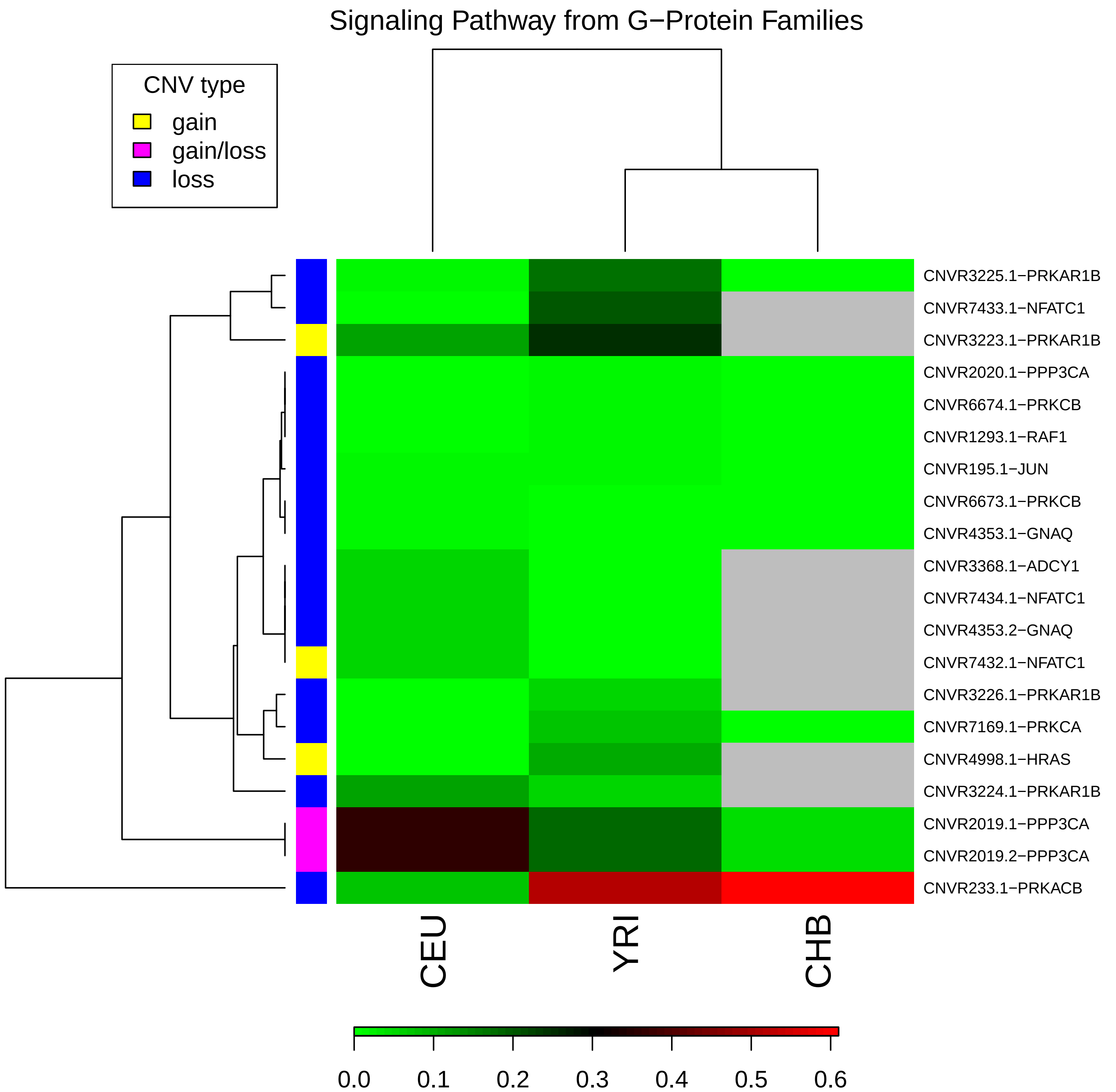
Signaling Pathway from G−Protein Families
CNV type
gain
gain/loss
loss
CNVR3225.1−PRKAR1B
CNVR7433.1−NFATC1
CNVR3223.1−PRKAR1B
CNVR2020.1−PPP3CA
CNVR6674.1−PRKCB
CNVR1293.1−RAF1
CNVR195.1−JUN
CNVR6673.1−PRKCB
CNVR4353.1−GNAQ
CNVR3368.1−ADCY1
CNVR7434.1−NFATC1
CNVR4353.2−GNAQ
CNVR7432.1−NFATC1
CNVR3226.1−PRKAR1B
CNVR7169.1−PRKCA
CNVR4998.1−HRAS
CNVR3224.1−PRKAR1B
CNVR2019.1−PPP3CA
CNVR2019.2−PPP3CA
CNVR233.1−PRKACB
CEU
YRI
CHB
0.0
0.1
0.2
0.3
0.4
0.5
0.6

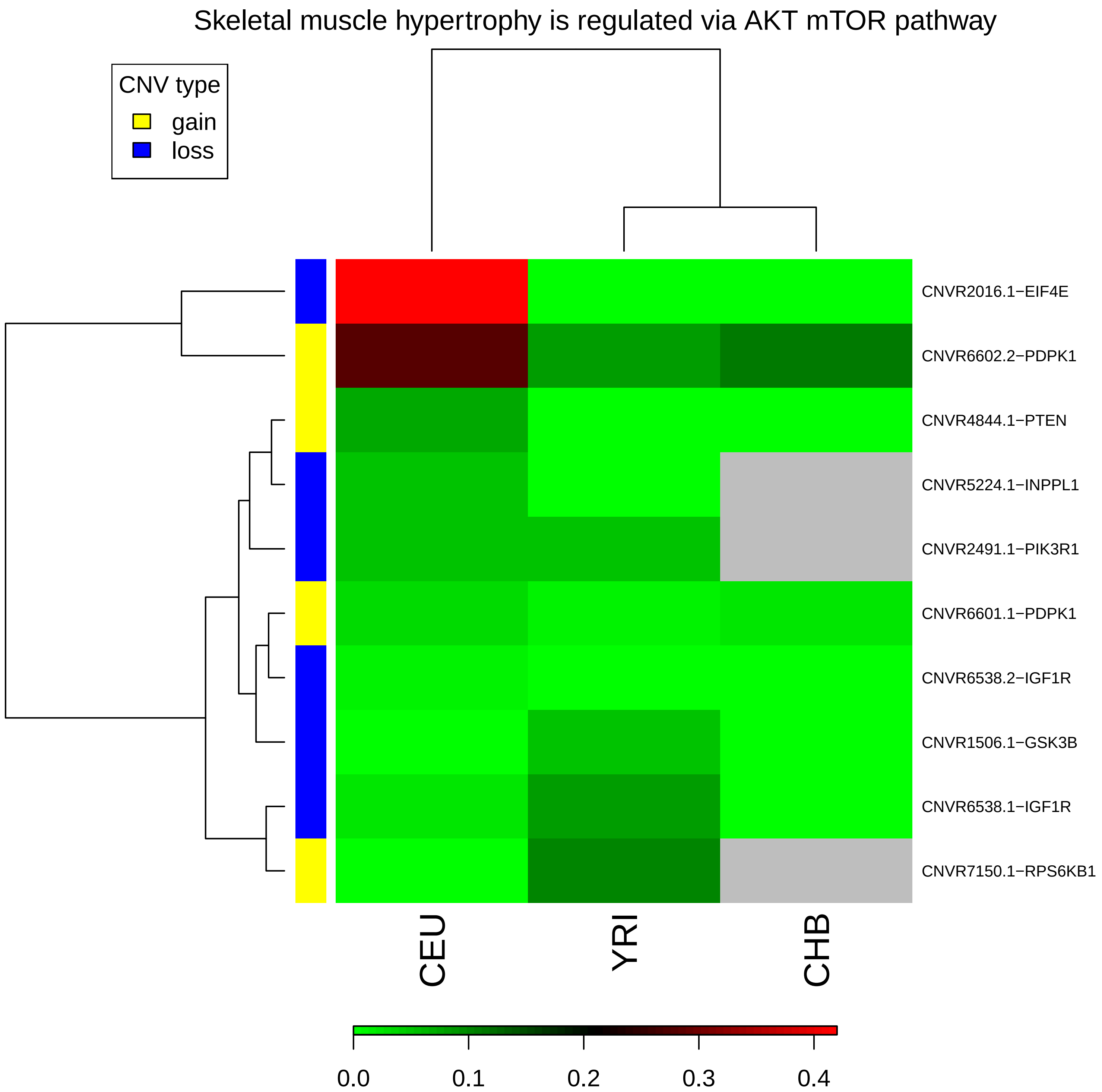
Skeletal muscle hypertrophy is regulated via AKT mTOR pathway
CNV type
gain
loss
CNVR2016.1−EIF4E
CNVR6602.2−PDPK1
CNVR4844.1−PTEN
CNVR5224.1−INPPL1
CNVR2491.1−PIK3R1
CNVR6601.1−PDPK1
CNVR6538.2−IGF1R
CNVR1506.1−GSK3B
CNVR6538.1−IGF1R
CNVR7150.1−RPS6KB1
CEU
YRI
CHB
0.0
0.1
0.2
0.3
0.4

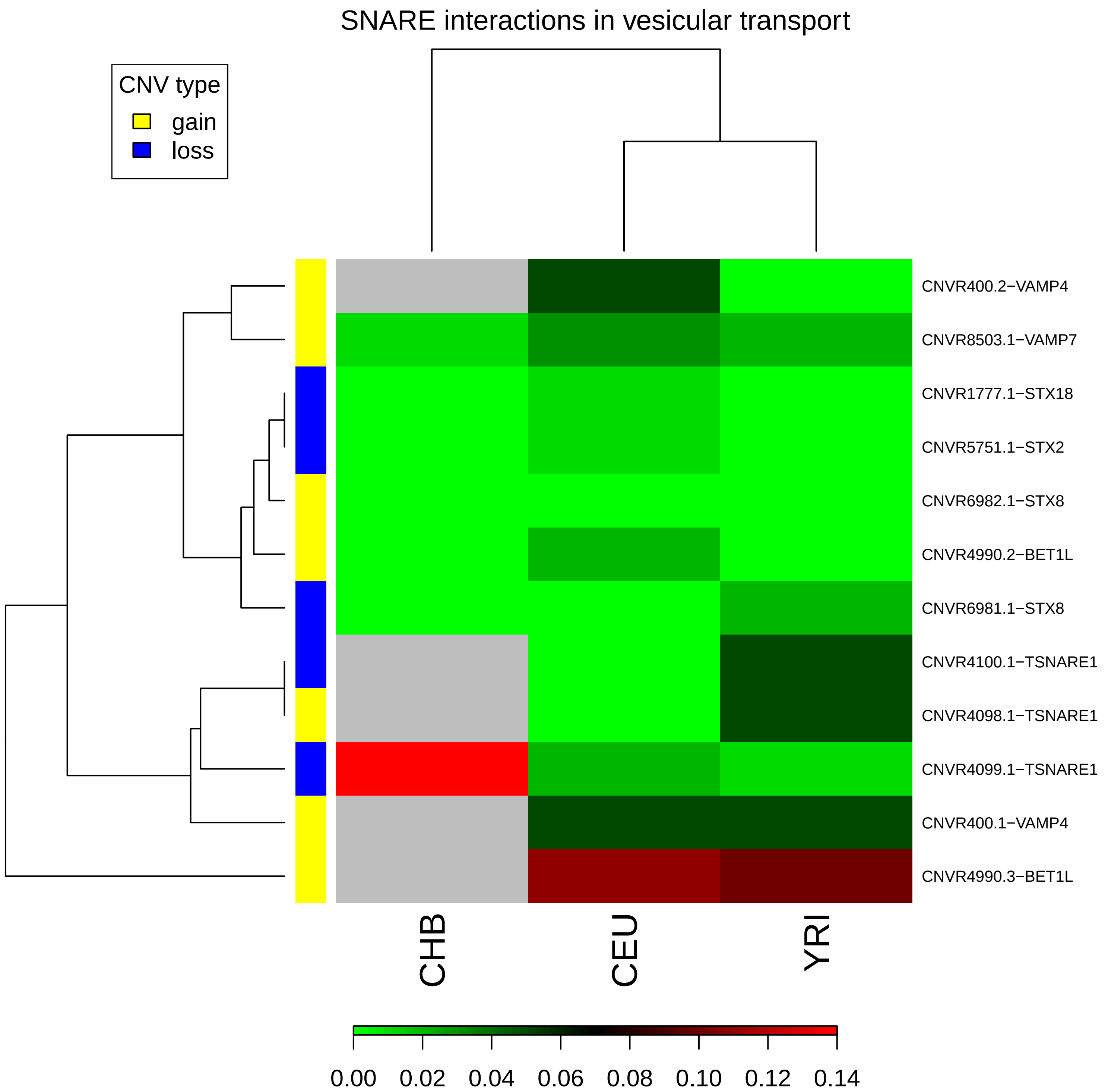

SNARE interactions in vesicular transport
CNV type
gain
loss
CNVR400.2−VAMP4
CNVR8503.1−VAMP7
CNVR1777.1−STX18
CNVR5751.1−STX2
CNVR6982.1−STX8
CNVR4990.2−BET1L
CNVR6981.1−STX8
CNVR4100.1−TSNARE1
CNVR4098.1−TSNARE1
CNVR4099.1−TSNARE1
CNVR400.1−VAMP4
CNVR4990.3−BET1L
CHB
CEU
YRI
0.00
0.02
0.04
0.06
0.08
0.10
0.12
0.14

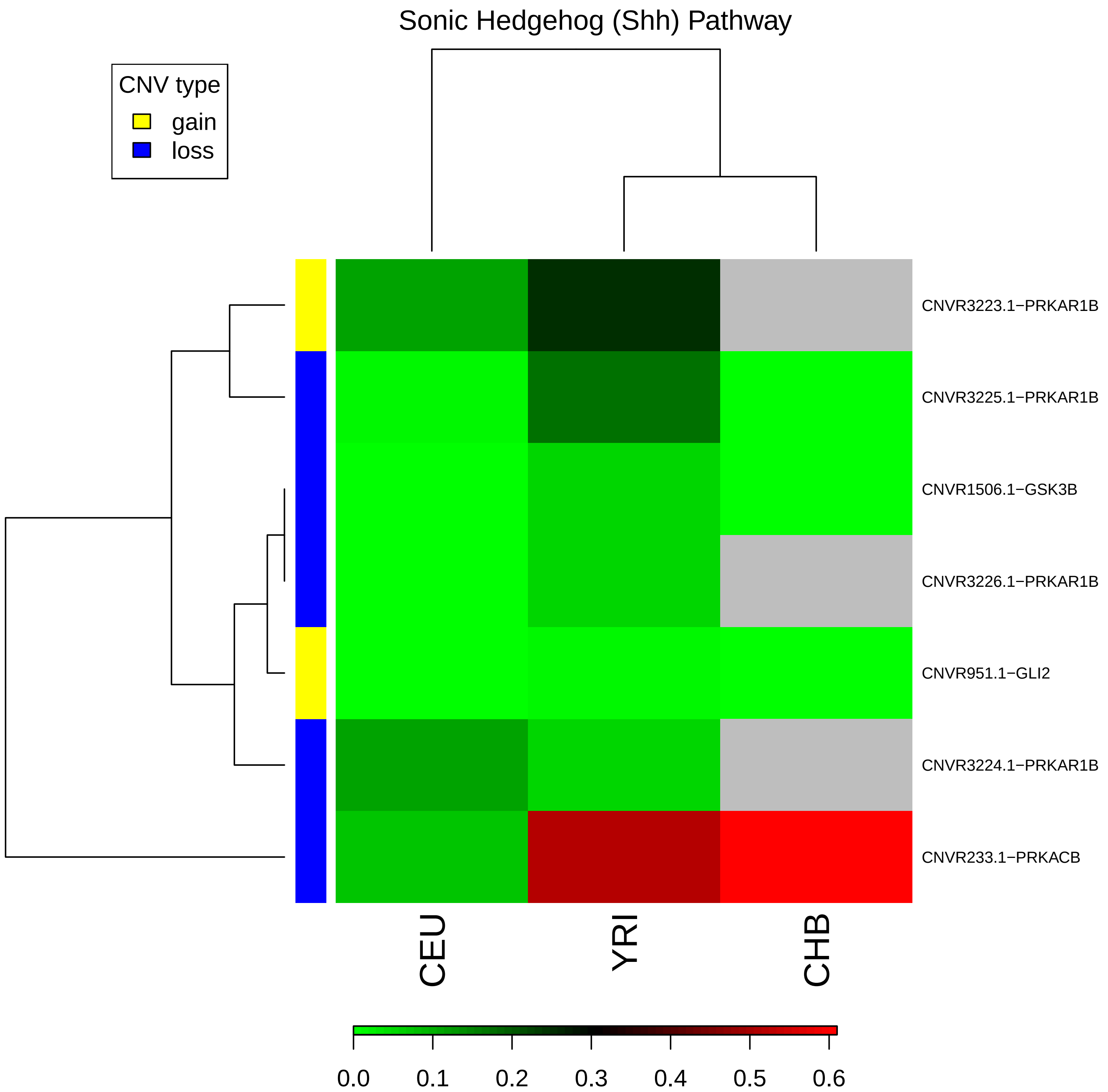

Sonic Hedgehog (Shh) Pathway
CNV type
gain
loss
CNVR3223.1−PRKAR1B
CNVR3225.1−PRKAR1B
CNVR1506.1−GSK3B
CNVR3226.1−PRKAR1B
CNVR951.1−GLI2
CNVR3224.1−PRKAR1B
CNVR233.1−PRKACB
CEU
YRI
CHB
0.0
0.1
0.2
0.3
0.4
0.5
0.6

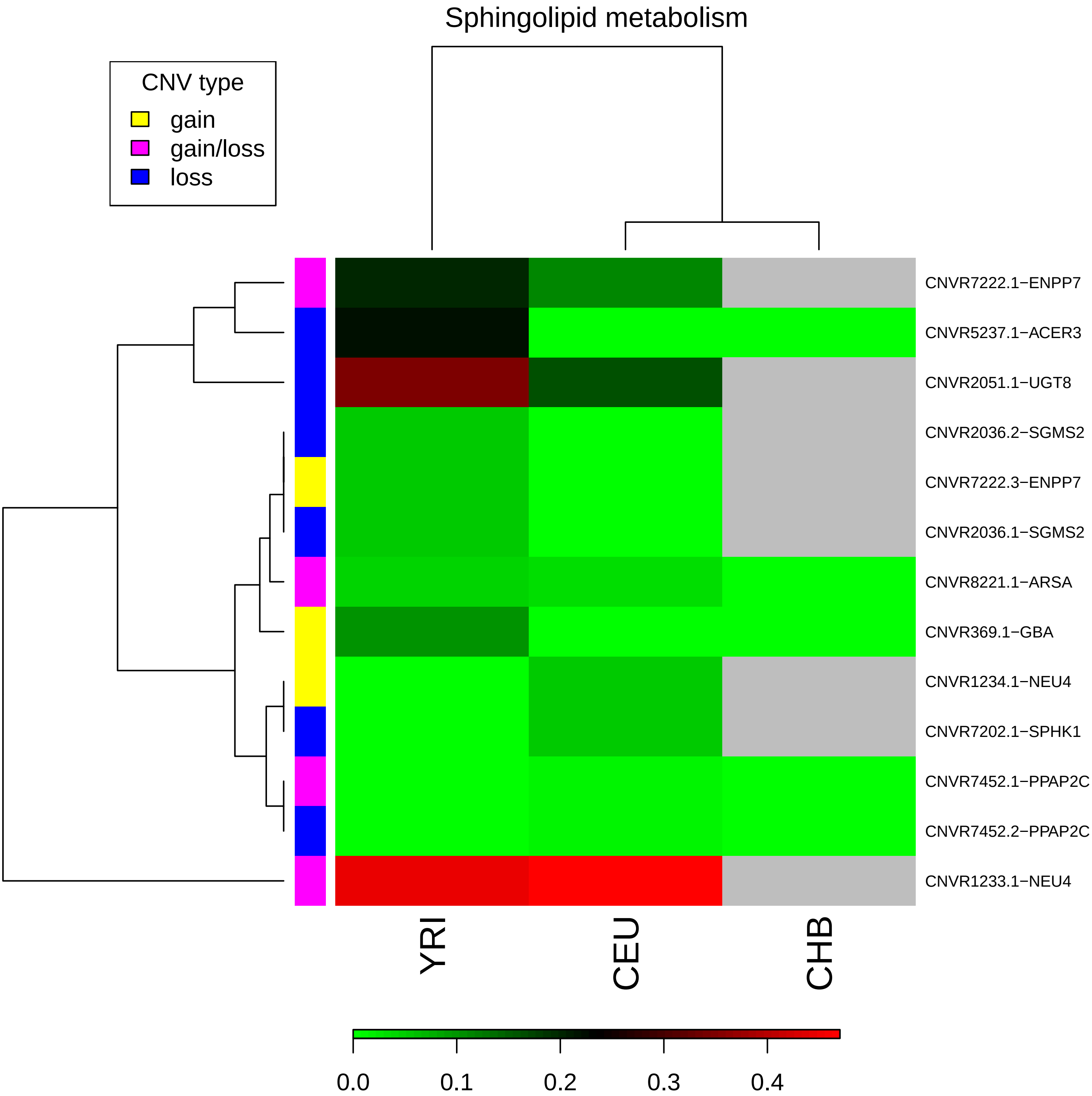
Sphingolipid metabolism
CNV type
gain
gain/loss
loss
CNVR7222.1−ENPP7
CNVR5237.1−ACER3
CNVR2051.1−UGT8
CNVR2036.2−SGMS2
CNVR7222.3−ENPP7
CNVR2036.1−SGMS2
CNVR8221.1−ARSA
CNVR369.1−GBA
CNVR1234.1−NEU4
CNVR7202.1−SPHK1
CNVR7452.1−PPAP2C
CNVR7452.2−PPAP2C
CNVR1233.1−NEU4
YRI
CEU
CHB
0.0
0.1
0.2
0.3
0.4

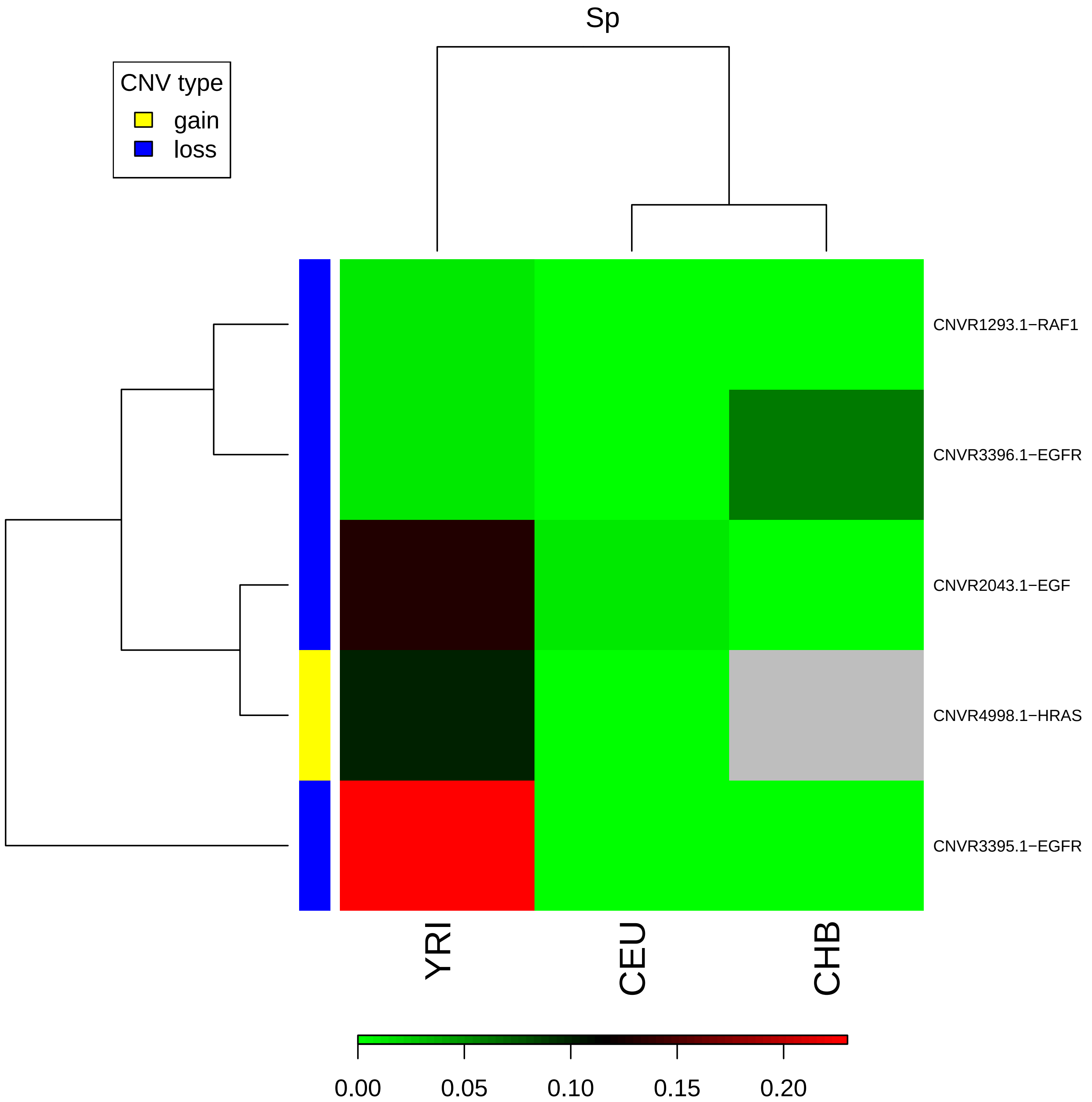

Sp
CNV type
gain
loss
CNVR1293.1−RAF1
CNVR3396.1−EGFR
CNVR2043.1−EGF
CNVR4998.1−HRAS
CNVR3395.1−EGFR
YRI
CEU
CHB
0.00
0.05
0.10
0.15
0.20

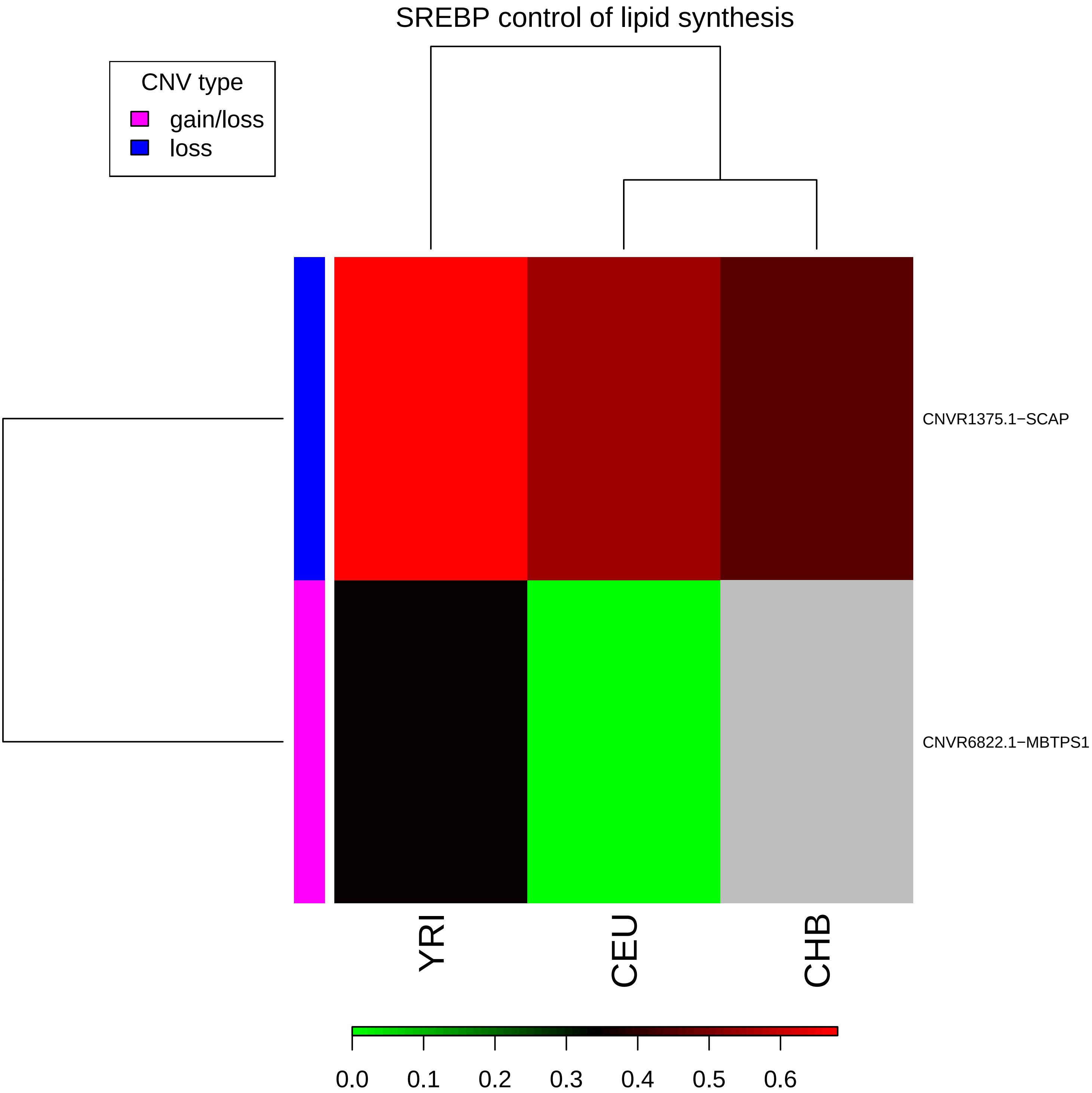

SREBP control of lipid synthesis
CNV type
gain/loss
loss
CNVR1375.1−SCAP
CNVR6822.1−MBTPS1
YRI
CEU
CHB
0.0
0.1
0.2
0.3
0.4
0.5
0.6

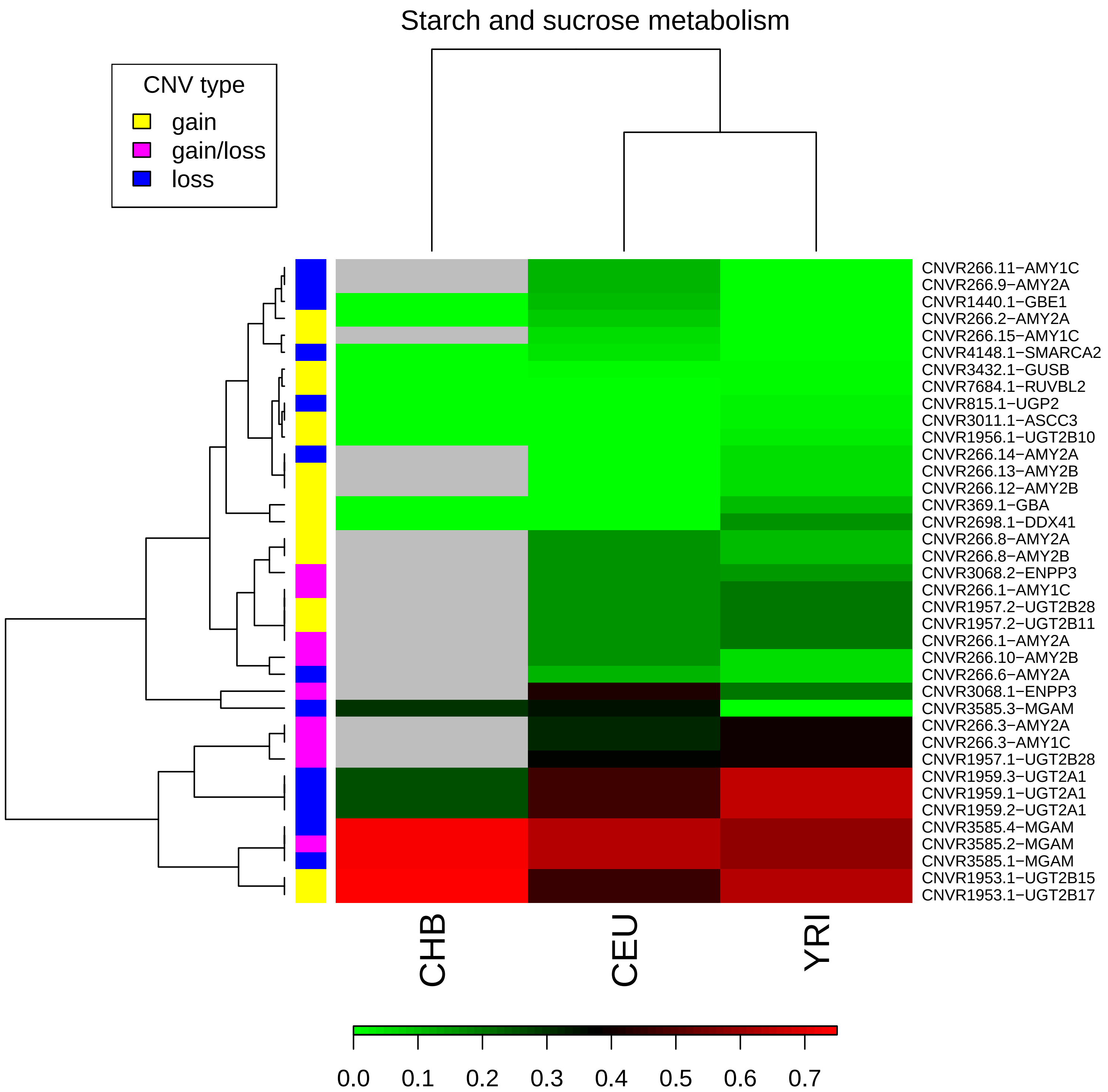
Starch and sucrose metabolism
CNV type
gain
gain/loss
loss
CNVR266.11−AMY1C
CNVR266.9−AMY2A
CNVR1440.1−GBE1
CNVR266.2−AMY2A
CNVR266.15−AMY1C
CNVR4148.1−SMARCA2
CNVR3432.1−GUSB
CNVR7684.1−RUVBL2
CNVR815.1−UGP2
CNVR3011.1−ASCC3
CNVR1956.1−UGT2B10
CNVR266.14−AMY2A
CNVR266.13−AMY2B
CNVR266.12−AMY2B
CNVR369.1−GBA
CNVR2698.1−DDX41
CNVR266.8−AMY2A
CNVR266.8−AMY2B
CNVR3068.2−ENPP3
CNVR266.1−AMY1C
CNVR1957.2−UGT2B28
CNVR1957.2−UGT2B11
CNVR266.1−AMY2A
CNVR266.10−AMY2B
CNVR266.6−AMY2A
CNVR3068.1−ENPP3
CNVR3585.3−MGAM
CNVR266.3−AMY2A
CNVR266.3−AMY1C
CNVR1957.1−UGT2B28
CNVR1959.3−UGT2A1
CNVR1959.1−UGT2A1
CNVR1959.2−UGT2A1
CNVR3585.4−MGAM
CNVR3585.2−MGAM
CNVR3585.1−MGAM
CNVR1953.1−UGT2B15
CNVR1953.1−UGT2B17
CHB
CEU
YRI
0.0
0.1
0.2
0.3
0.4
0.5
0.6
0.7

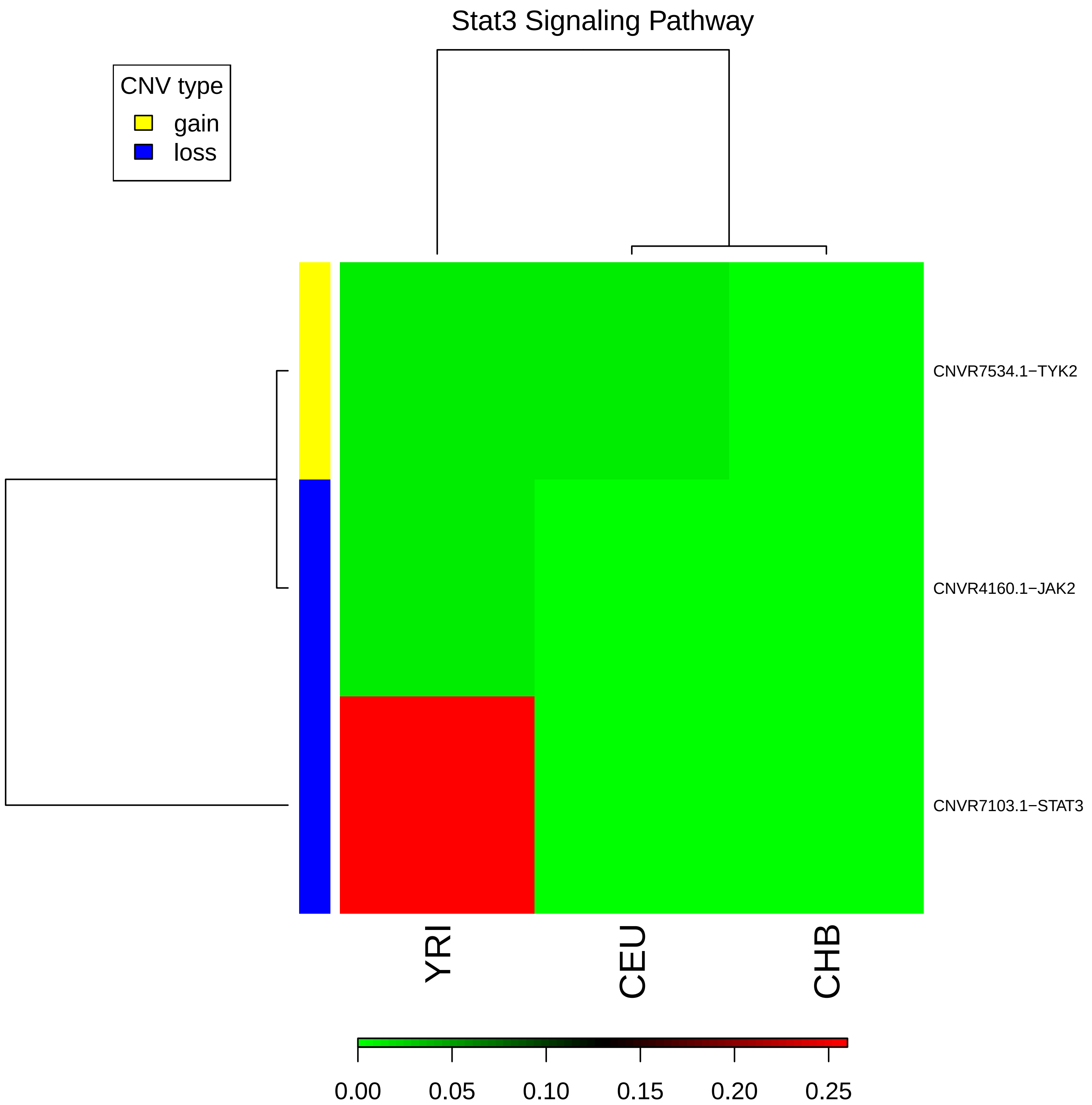
Stat3 Signaling Pathway
CNV type
gain
loss
CNVR7534.1−TYK2
CNVR4160.1−JAK2
CNVR7103.1−STAT3
YRI
CEU
CHB
0.00
0.05
0.10
0.15
0.20
0.25

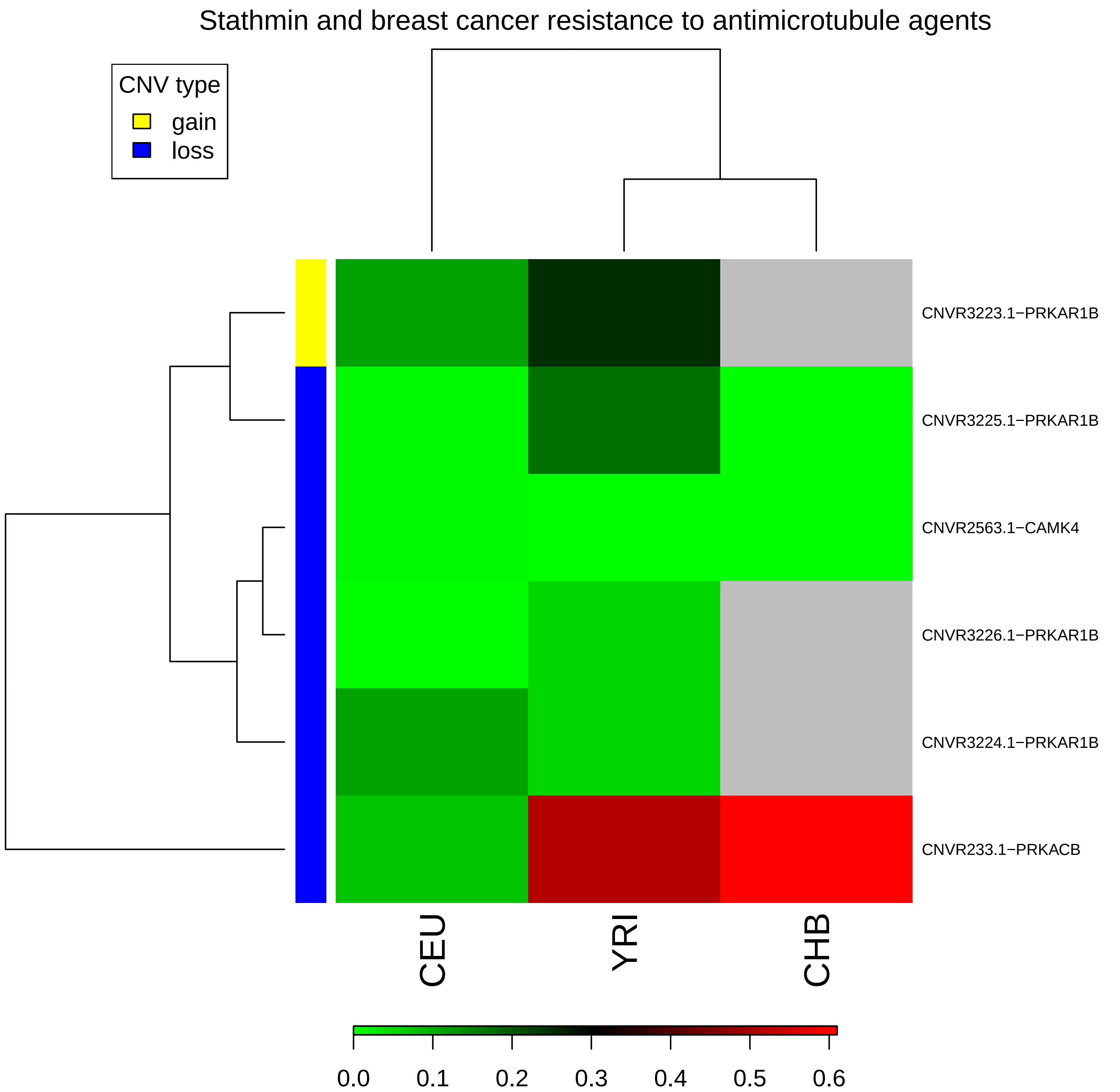
Stathmin and breast cancer resistance to antimicrotubule agents
CNV type
gain
loss
CNVR3223.1−PRKAR1B
CNVR3225.1−PRKAR1B
CNVR2563.1−CAMK4
CNVR3226.1−PRKAR1B
CNVR3224.1−PRKAR1B
CNVR233.1−PRKACB
CEU
YRI
CHB
0.0
0.1
0.2
0.3
0.4
0.5
0.6

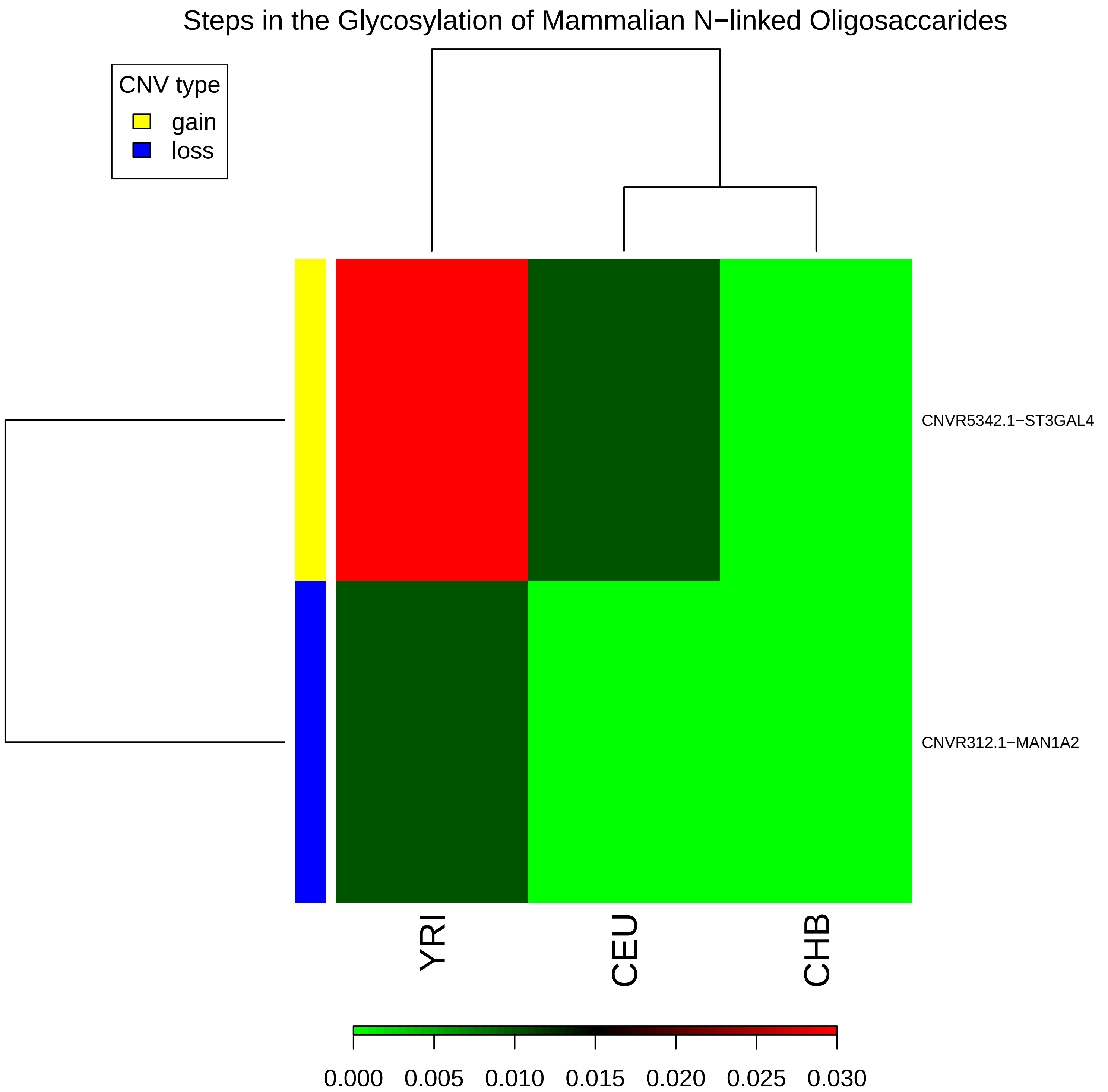
Steps in the Glycosylation of Mammalian N−linked Oligosaccarides
CNV type
gain
loss
CNVR5342.1−ST3GAL4
CNVR312.1−MAN1A2
YRI
CEU
CHB
0.000
0.005
0.010
0.015
0.020
0.025
0.030

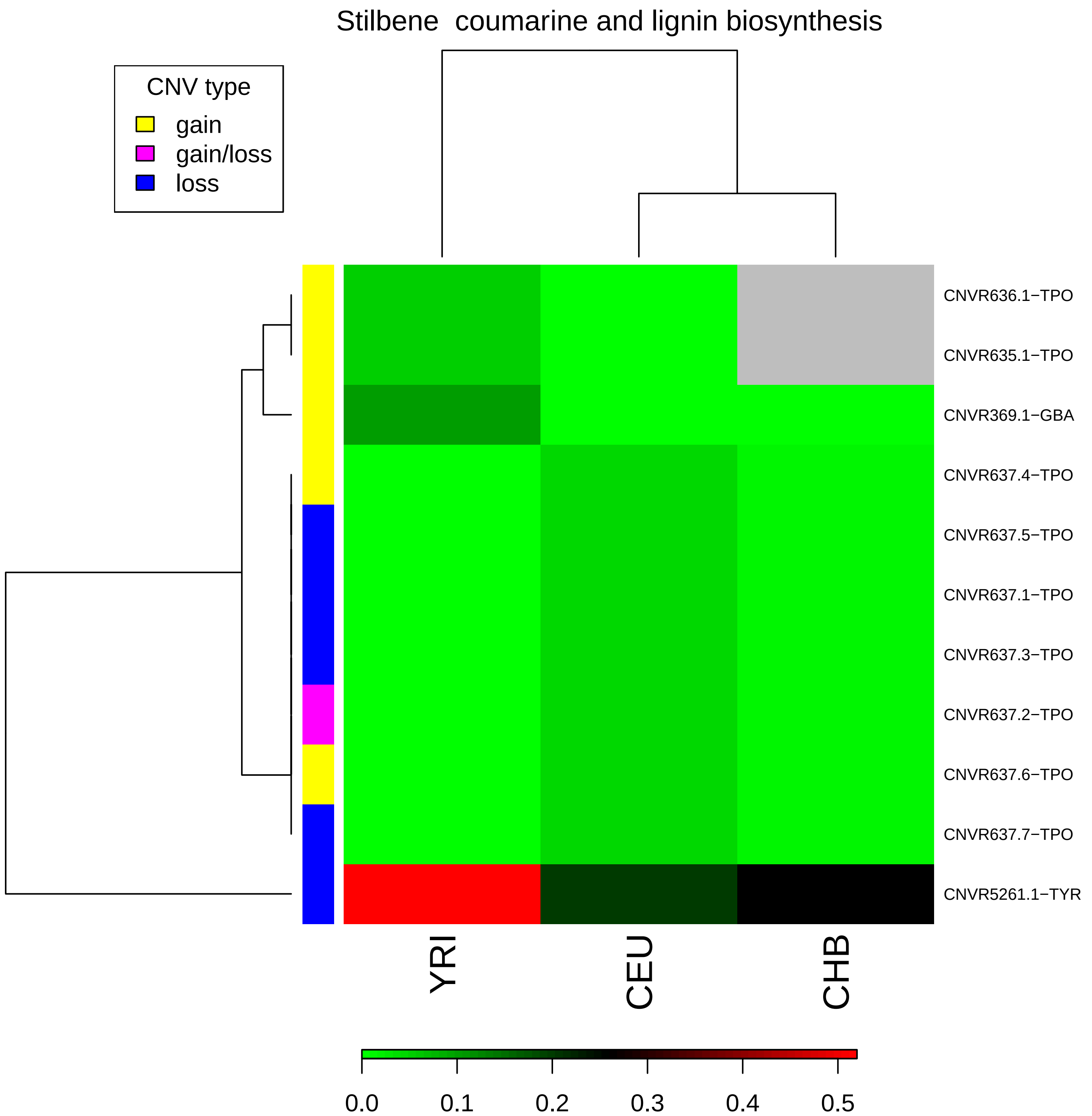

Stilbene  coumarine and lignin biosynthesis
CNV type
gain
gain/loss
loss
CNVR636.1−TPO
CNVR635.1−TPO
CNVR369.1−GBA
CNVR637.4−TPO
CNVR637.5−TPO
CNVR637.1−TPO
CNVR637.3−TPO
CNVR637.2−TPO
CNVR637.6−TPO
CNVR637.7−TPO
CNVR5261.1−TYR
YRI
CEU
CHB
0.0
0.1
0.2
0.3
0.4
0.5

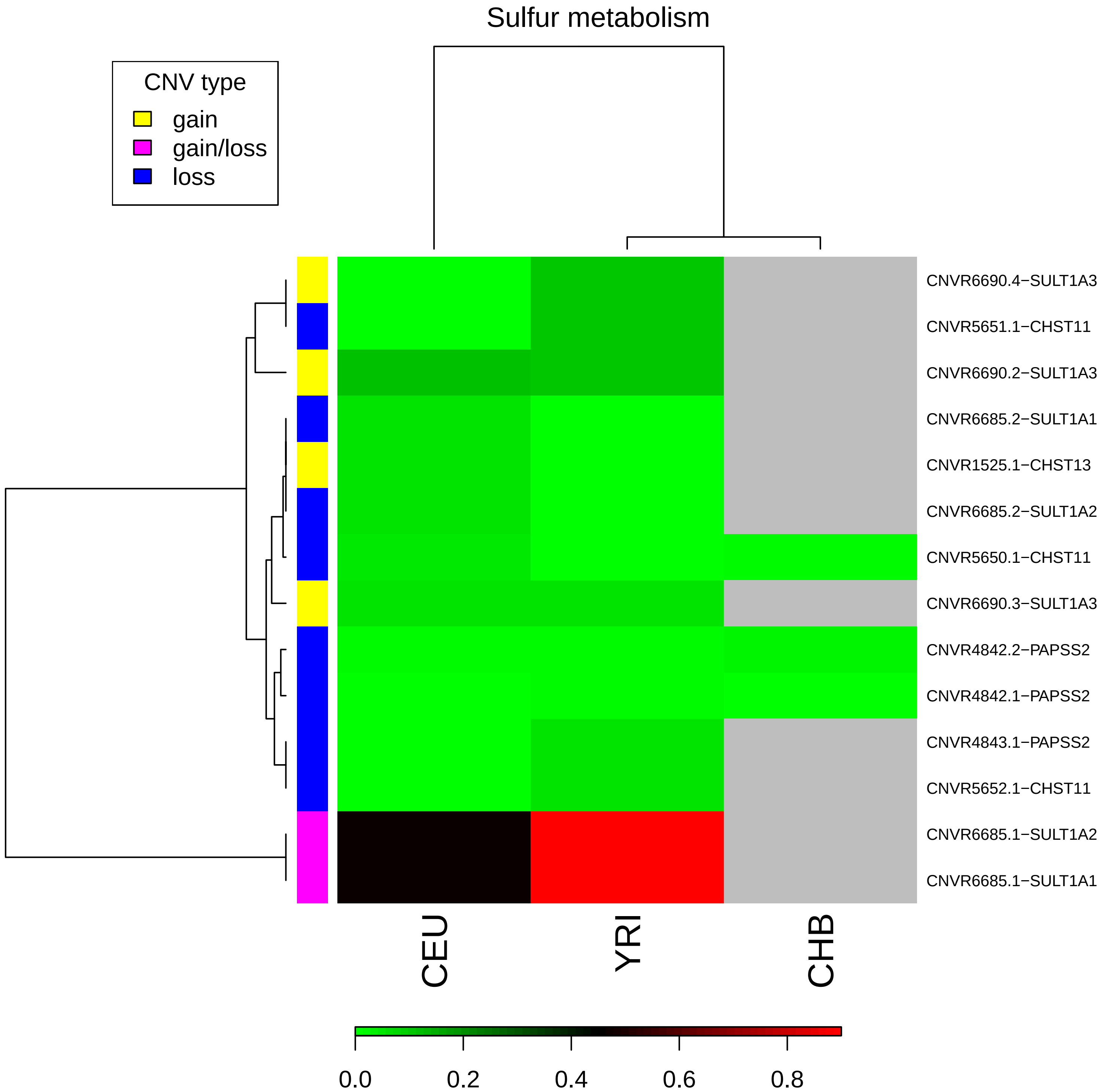
Sulfur metabolism
CNV type
gain
gain/loss
loss
CNVR6690.4−SULT1A3
CNVR5651.1−CHST11
CNVR6690.2−SULT1A3
CNVR6685.2−SULT1A1
CNVR1525.1−CHST13
CNVR6685.2−SULT1A2
CNVR5650.1−CHST11
CNVR6690.3−SULT1A3
CNVR4842.2−PAPSS2
CNVR4842.1−PAPSS2
CNVR4843.1−PAPSS2
CNVR5652.1−CHST11
CNVR6685.1−SULT1A2
CNVR6685.1−SULT1A1
CEU
YRI
CHB
0.0
0.2
0.4
0.6
0.8

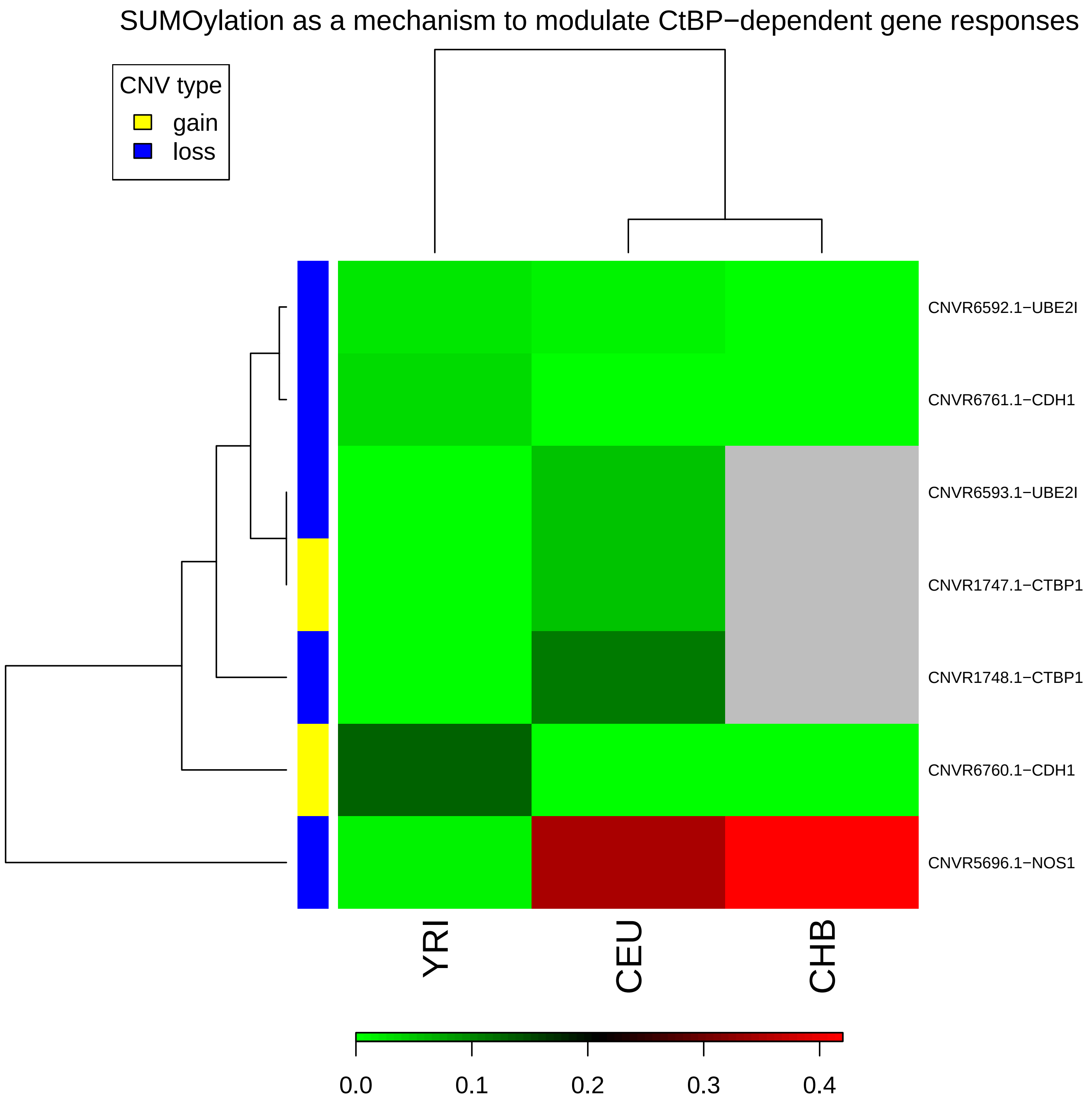
SUMOylation as a mechanism to modulate CtBP−dependent gene responses
CNV type
gain
loss
CNVR6592.1−UBE2I
CNVR6761.1−CDH1
CNVR6593.1−UBE2I
CNVR1747.1−CTBP1
CNVR1748.1−CTBP1
CNVR6760.1−CDH1
CNVR5696.1−NOS1
YRI
CEU
CHB
0.0
0.1
0.2
0.3
0.4

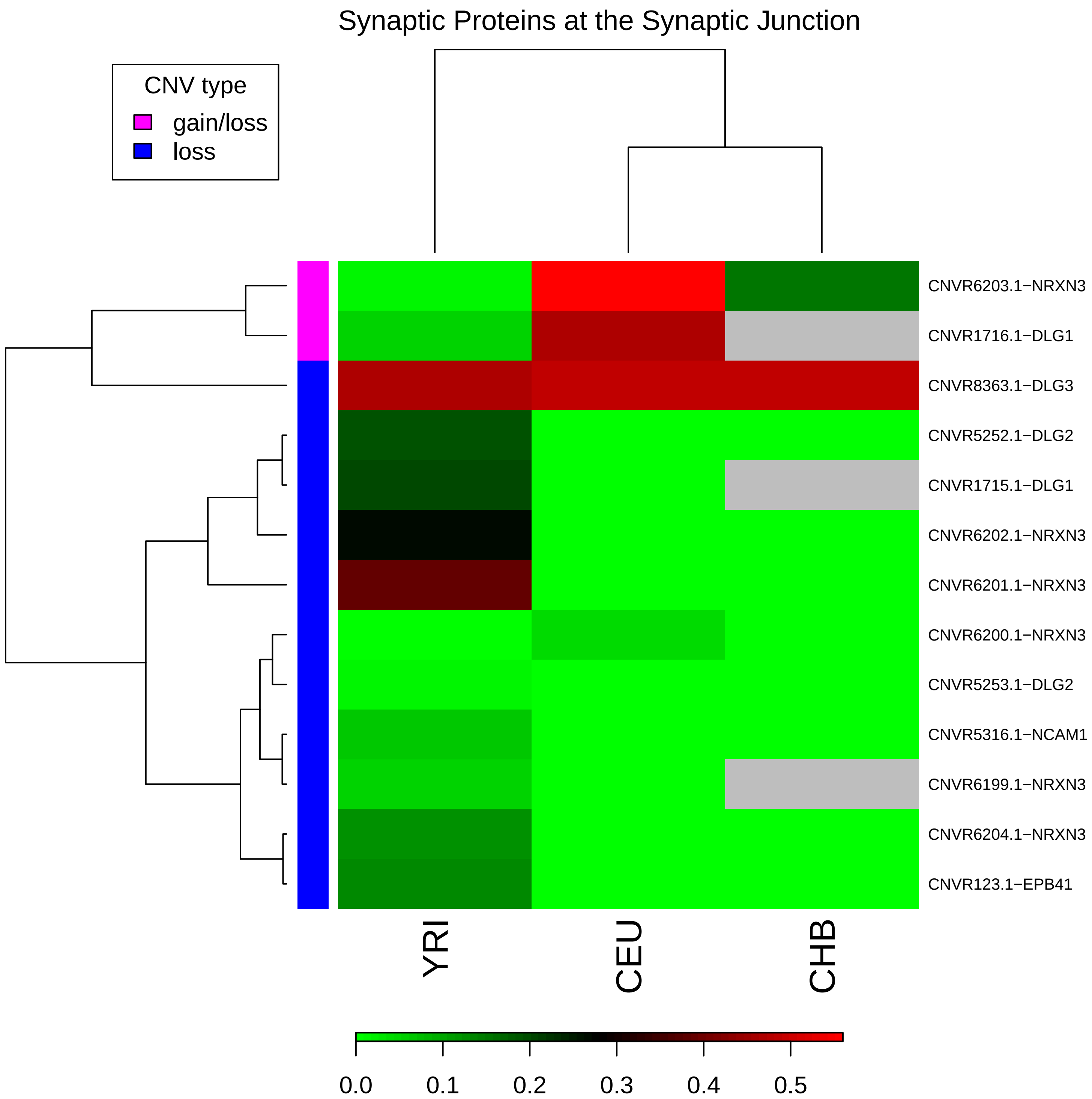

Synaptic Proteins at the Synaptic Junction
CNV type
gain/loss
loss
CNVR6203.1−NRXN3
CNVR1716.1−DLG1
CNVR8363.1−DLG3
CNVR5252.1−DLG2
CNVR1715.1−DLG1
CNVR6202.1−NRXN3
CNVR6201.1−NRXN3
CNVR6200.1−NRXN3
CNVR5253.1−DLG2
CNVR5316.1−NCAM1
CNVR6199.1−NRXN3
CNVR6204.1−NRXN3
CNVR123.1−EPB41
YRI
CEU
CHB
0.0
0.1
0.2
0.3
0.4
0.5

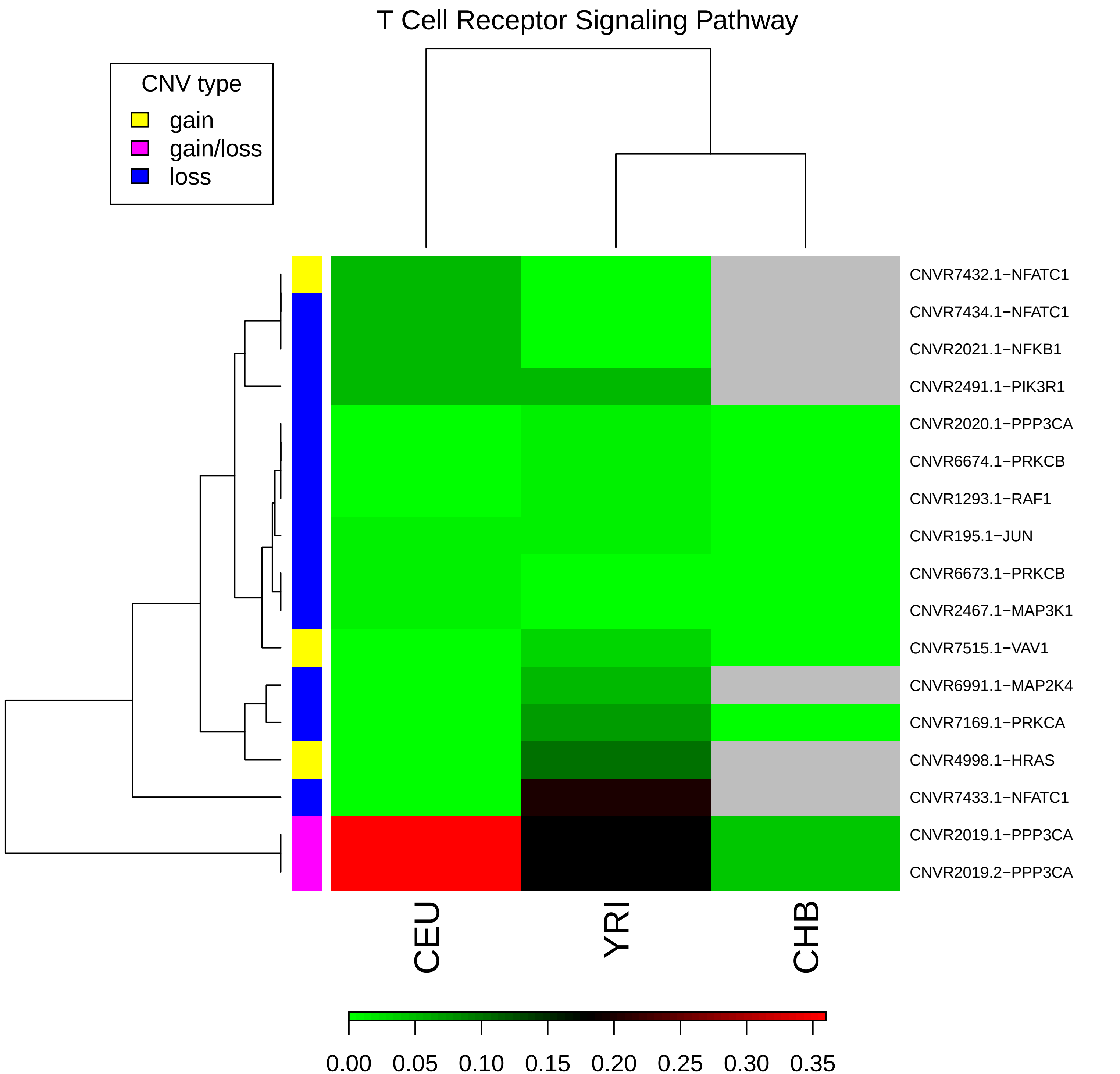
T Cell Receptor Signaling Pathway
CNV type
gain
gain/loss
loss
CNVR7432.1−NFATC1
CNVR7434.1−NFATC1
CNVR2021.1−NFKB1
CNVR2491.1−PIK3R1
CNVR2020.1−PPP3CA
CNVR6674.1−PRKCB
CNVR1293.1−RAF1
CNVR195.1−JUN
CNVR6673.1−PRKCB
CNVR2467.1−MAP3K1
CNVR7515.1−VAV1
CNVR6991.1−MAP2K4
CNVR7169.1−PRKCA
CNVR4998.1−HRAS
CNVR7433.1−NFATC1
CNVR2019.1−PPP3CA
CNVR2019.2−PPP3CA
CEU
YRI
CHB
0.00
0.05
0.10
0.15
0.20
0.25
0.30
0.35

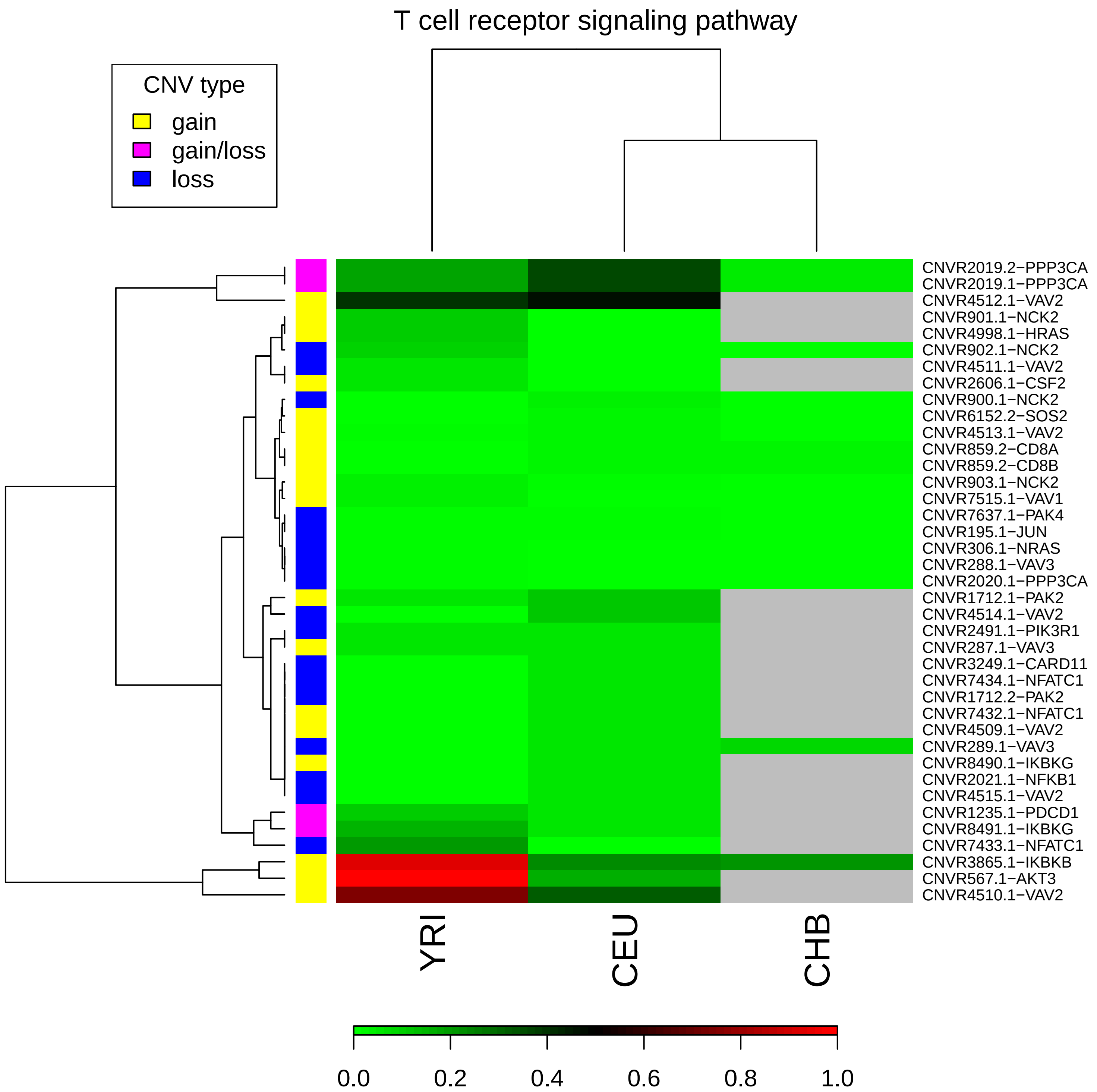
T cell receptor signaling pathway
CNV type
gain
gain/loss
loss
CNVR2019.2−PPP3CA
CNVR2019.1−PPP3CA
CNVR4512.1−VAV2
CNVR901.1−NCK2
CNVR4998.1−HRAS
CNVR902.1−NCK2
CNVR4511.1−VAV2
CNVR2606.1−CSF2
CNVR900.1−NCK2
CNVR6152.2−SOS2
CNVR4513.1−VAV2
CNVR859.2−CD8A
CNVR859.2−CD8B
CNVR903.1−NCK2
CNVR7515.1−VAV1
CNVR7637.1−PAK4
CNVR195.1−JUN
CNVR306.1−NRAS
CNVR288.1−VAV3
CNVR2020.1−PPP3CA
CNVR1712.1−PAK2
CNVR4514.1−VAV2
CNVR2491.1−PIK3R1
CNVR287.1−VAV3
CNVR3249.1−CARD11
CNVR7434.1−NFATC1
CNVR1712.2−PAK2
CNVR7432.1−NFATC1
CNVR4509.1−VAV2
CNVR289.1−VAV3
CNVR8490.1−IKBKG
CNVR2021.1−NFKB1
CNVR4515.1−VAV2
CNVR1235.1−PDCD1
CNVR8491.1−IKBKG
CNVR7433.1−NFATC1
CNVR3865.1−IKBKB
CNVR567.1−AKT3
CNVR4510.1−VAV2
YRI
CEU
CHB
0.0
0.2
0.4
0.6
0.8
1.0

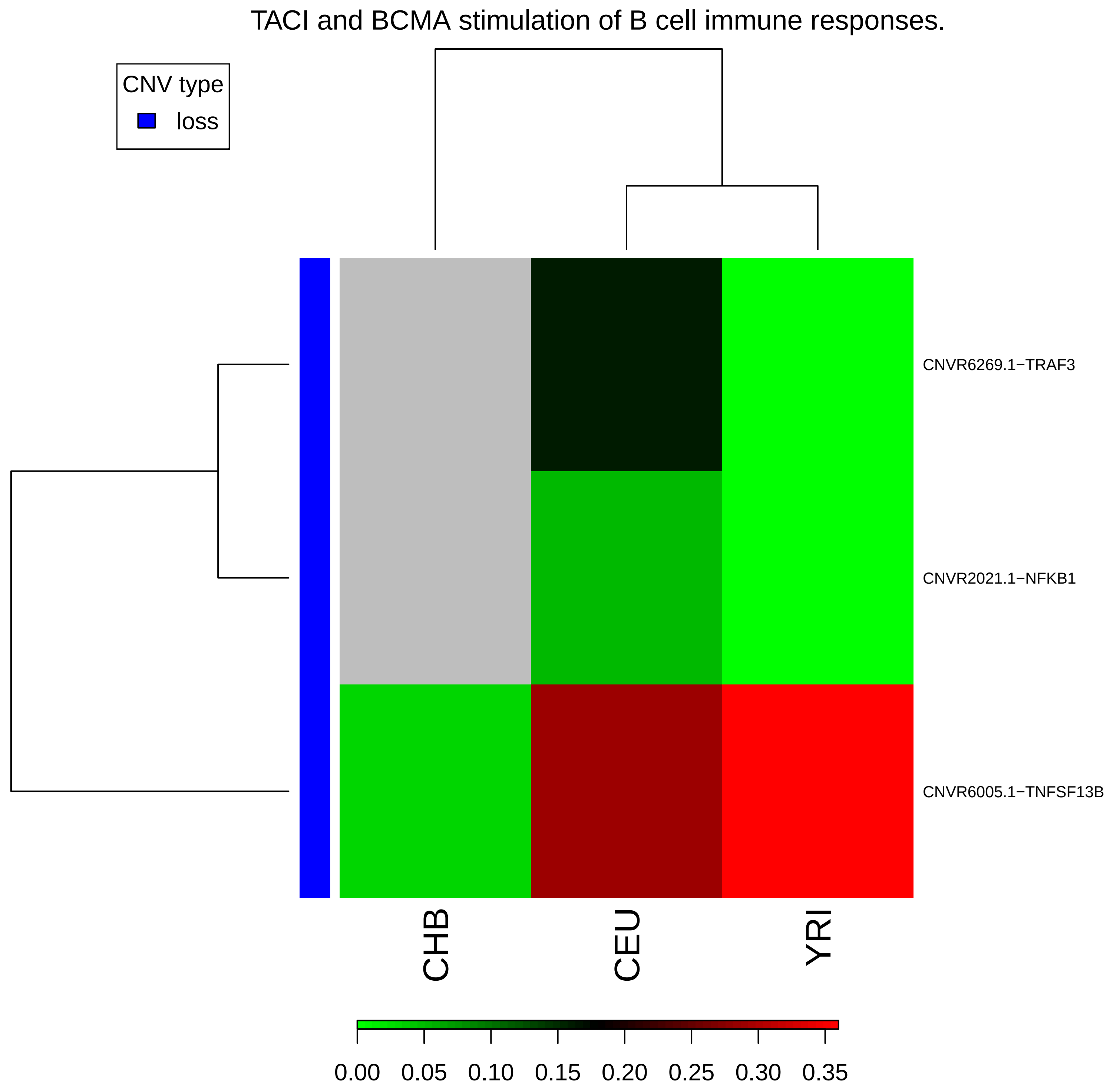

TACI and BCMA stimulation of B cell immune responses.
CNV type
loss
CNVR6269.1−TRAF3
CNVR2021.1−NFKB1
CNVR6005.1−TNFSF13B
CHB
CEU
YRI
0.00
0.05
0.10
0.15
0.20
0.25
0.30
0.35

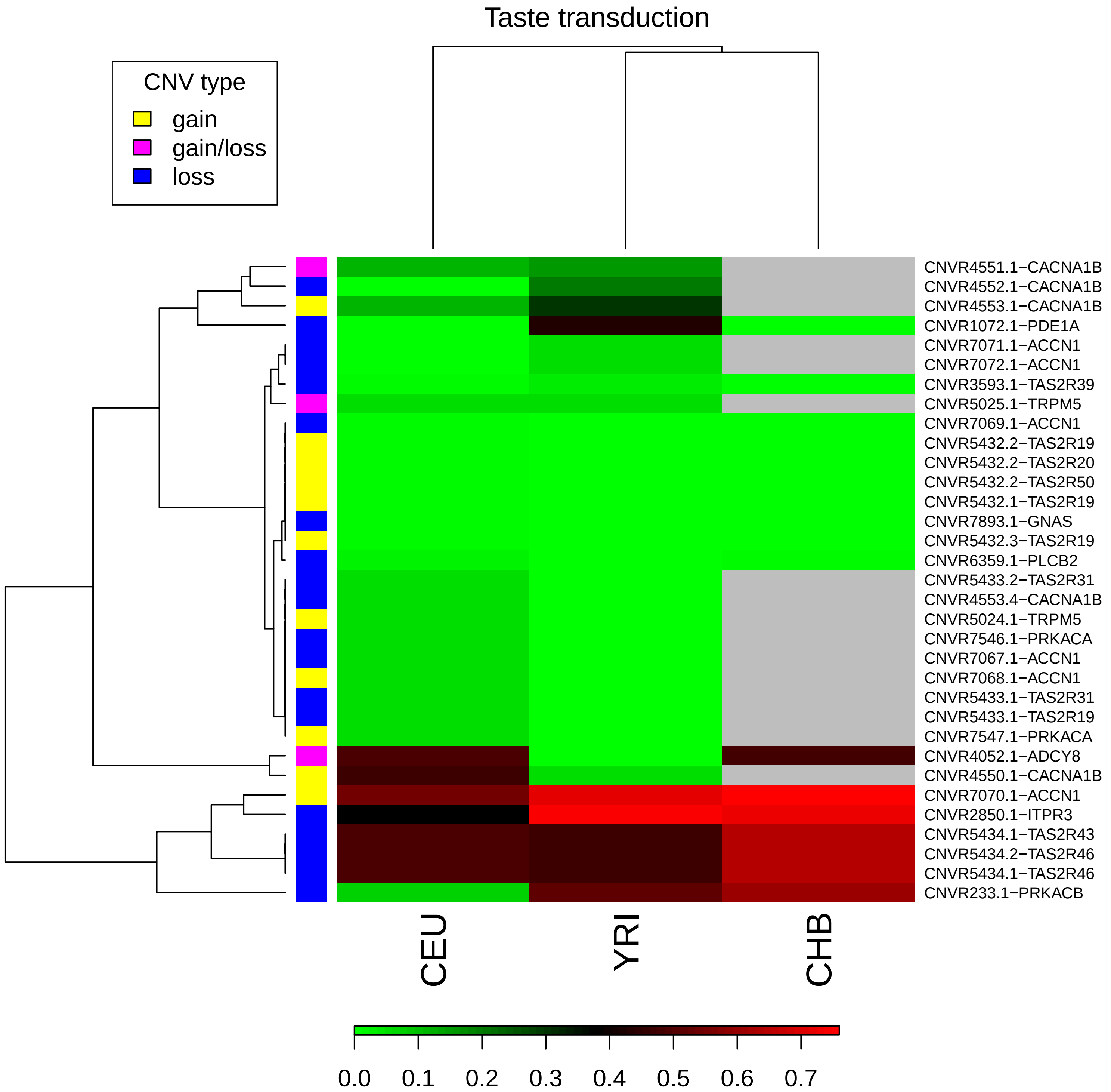

Taste transduction
CNV type
gain
gain/loss
loss
CNVR4551.1−CACNA1B
CNVR4552.1−CACNA1B
CNVR4553.1−CACNA1B
CNVR1072.1−PDE1A
CNVR7071.1−ACCN1
CNVR7072.1−ACCN1
CNVR3593.1−TAS2R39
CNVR5025.1−TRPM5
CNVR7069.1−ACCN1
CNVR5432.2−TAS2R19
CNVR5432.2−TAS2R20
CNVR5432.2−TAS2R50
CNVR5432.1−TAS2R19
CNVR7893.1−GNAS
CNVR5432.3−TAS2R19
CNVR6359.1−PLCB2
CNVR5433.2−TAS2R31
CNVR4553.4−CACNA1B
CNVR5024.1−TRPM5
CNVR7546.1−PRKACA
CNVR7067.1−ACCN1
CNVR7068.1−ACCN1
CNVR5433.1−TAS2R31
CNVR5433.1−TAS2R19
CNVR7547.1−PRKACA
CNVR4052.1−ADCY8
CNVR4550.1−CACNA1B
CNVR7070.1−ACCN1
CNVR2850.1−ITPR3
CNVR5434.1−TAS2R43
CNVR5434.2−TAS2R46
CNVR5434.1−TAS2R46
CNVR233.1−PRKACB
CEU
YRI
CHB
0.0
0.1
0.2
0.3
0.4
0.5
0.6
0.7

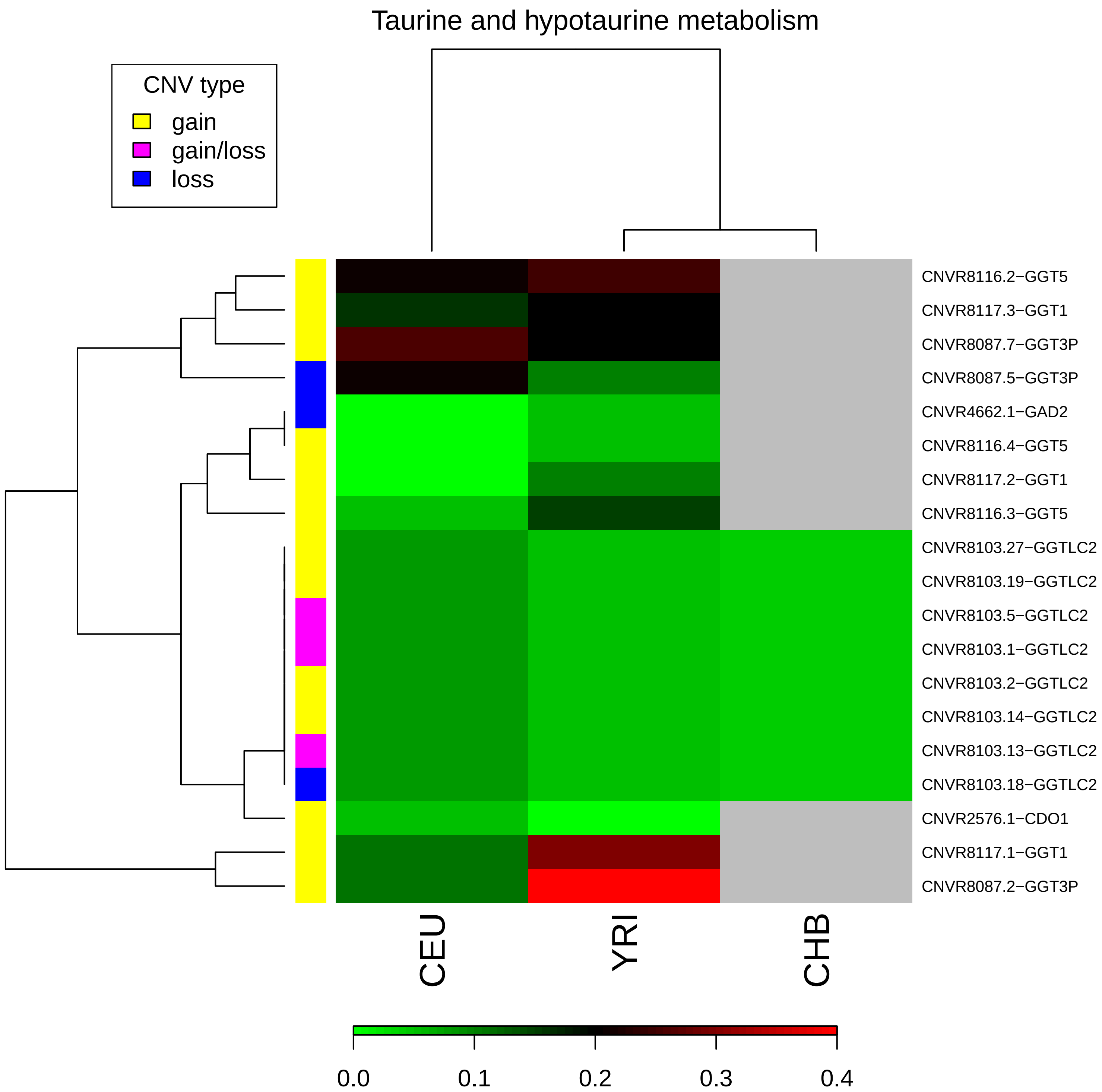

Taurine and hypotaurine metabolism
CNV type
gain
gain/loss
loss
CNVR8116.2−GGT5
CNVR8117.3−GGT1
CNVR8087.7−GGT3P
CNVR8087.5−GGT3P
CNVR4662.1−GAD2
CNVR8116.4−GGT5
CNVR8117.2−GGT1
CNVR8116.3−GGT5
CNVR8103.27−GGTLC2
CNVR8103.19−GGTLC2
CNVR8103.5−GGTLC2
CNVR8103.1−GGTLC2
CNVR8103.2−GGTLC2
CNVR8103.14−GGTLC2
CNVR8103.13−GGTLC2
CNVR8103.18−GGTLC2
CNVR2576.1−CDO1
CNVR8117.1−GGT1
CNVR8087.2−GGT3P
CEU
YRI
CHB
0.0
0.1
0.2
0.3
0.4

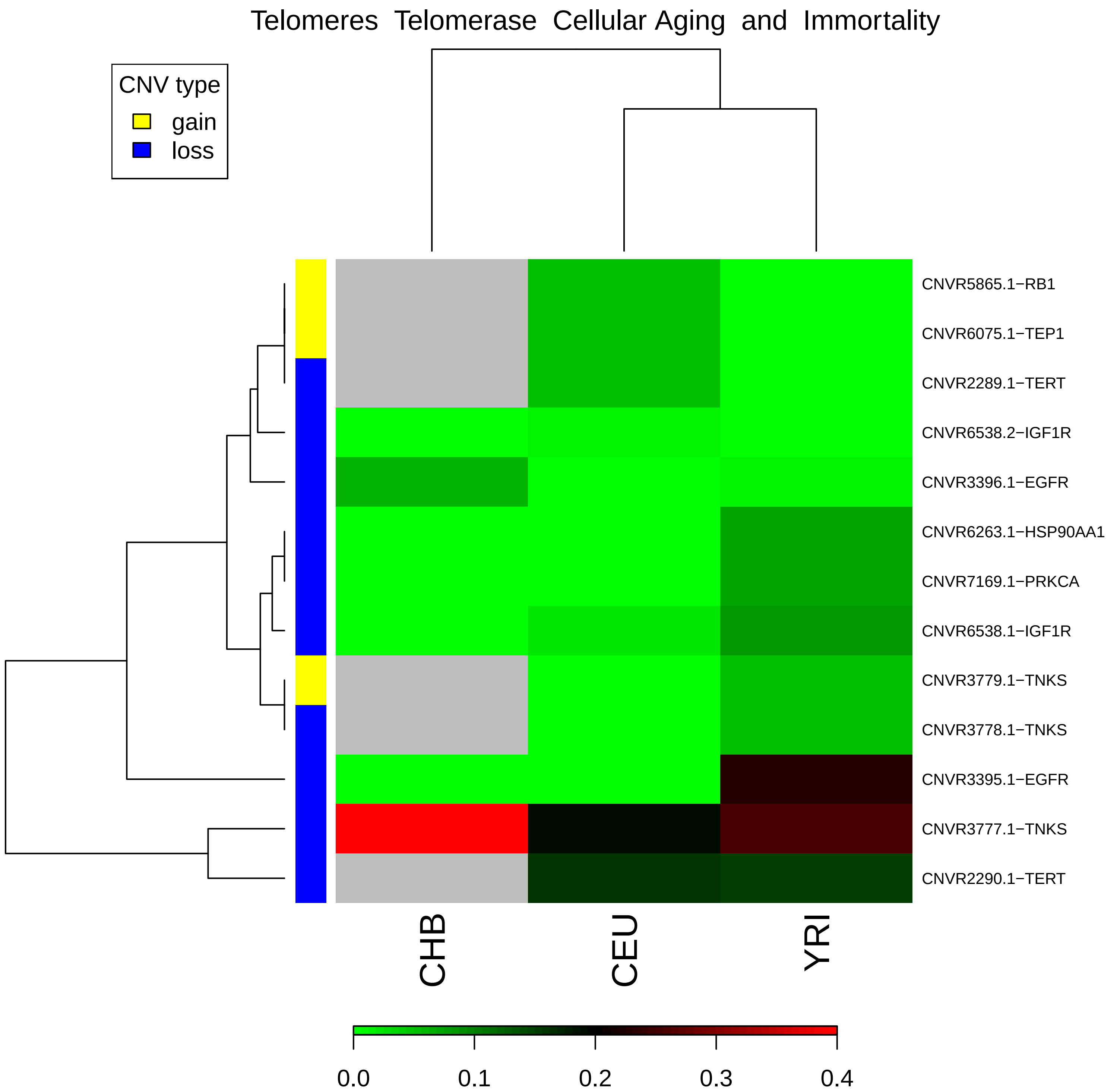
Telomeres Telomerase Cellular Aging and Immortality
CNV type
gain
loss
CNVR5865.1−RB1
CNVR6075.1−TEP1
CNVR2289.1−TERT
CNVR6538.2−IGF1R
CNVR3396.1−EGFR
CNVR6263.1−HSP90AA1
CNVR7169.1−PRKCA
CNVR6538.1−IGF1R
CNVR3779.1−TNKS
CNVR3778.1−TNKS
CNVR3395.1−EGFR
CNVR3777.1−TNKS
CNVR2290.1−TERT
CHB
CEU
YRI
0.0
0.1
0.2
0.3
0.4

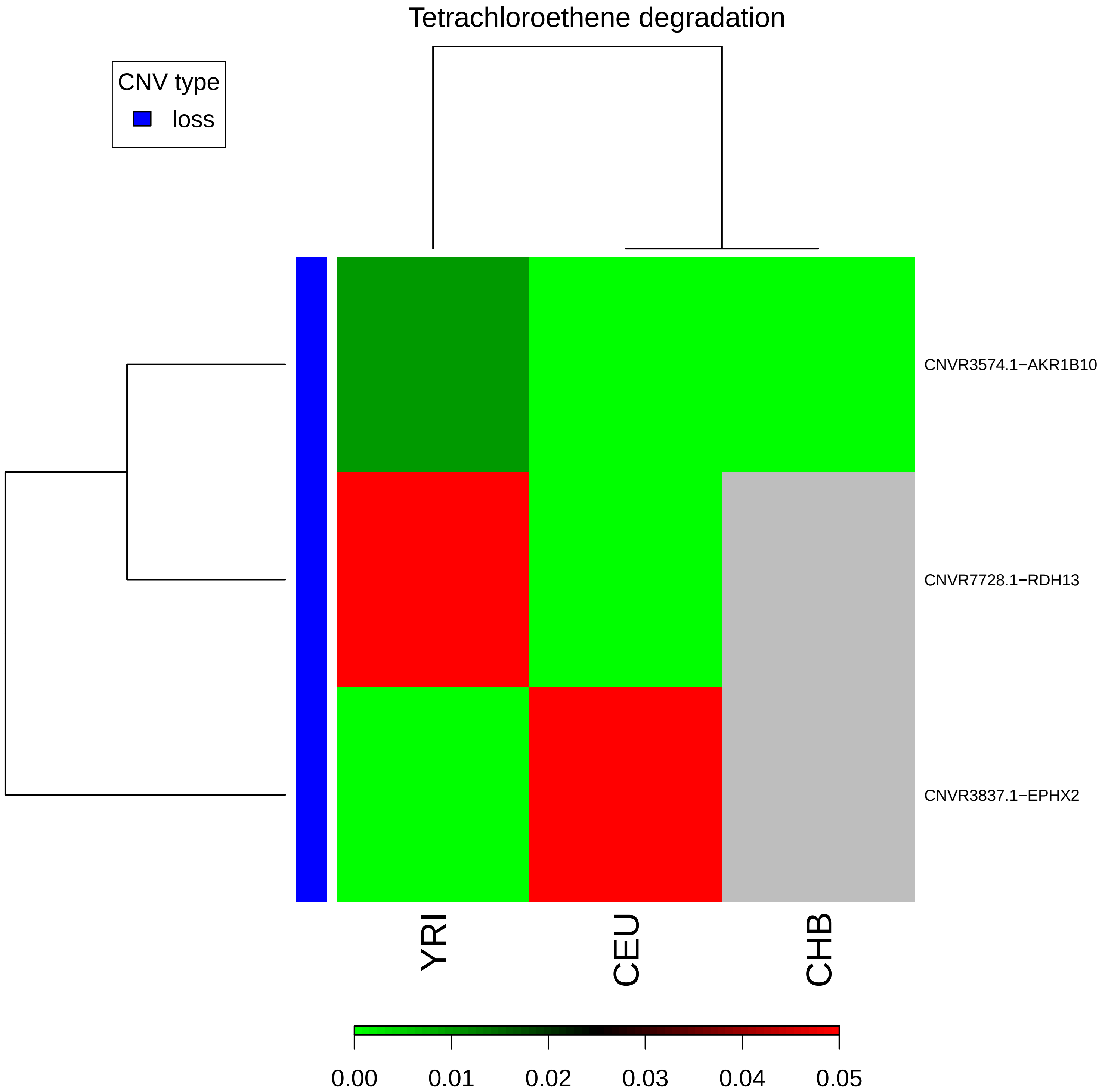

Tetrachloroethene degradation
CNV type
loss
CNVR3574.1−AKR1B10
CNVR7728.1−RDH13
CNVR3837.1−EPHX2
YRI
CEU
CHB
0.00
0.01
0.02
0.03
0.04
0.05

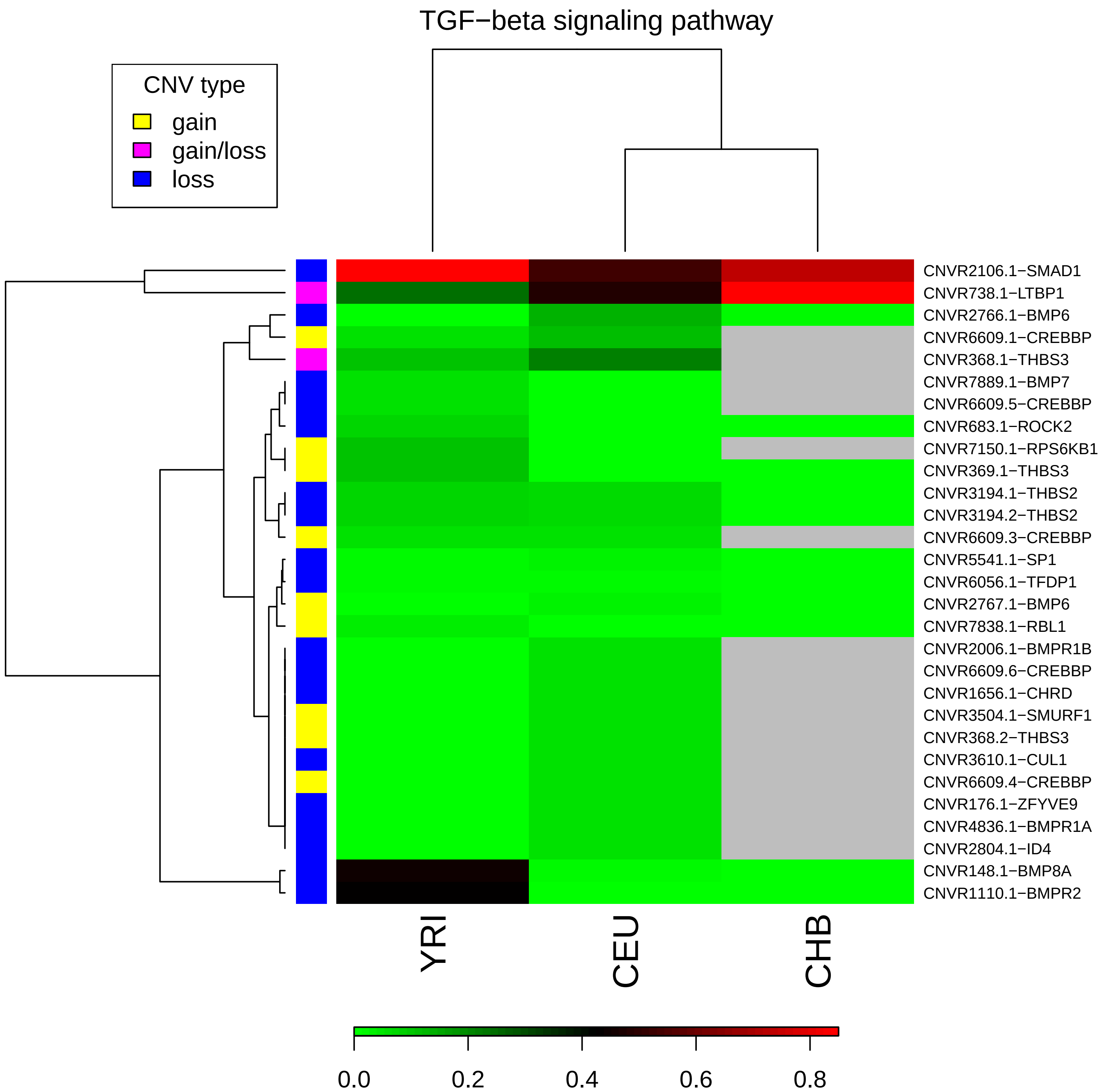
TGF−beta signaling pathway
CNV type
gain
gain/loss
loss
CNVR2106.1−SMAD1
CNVR738.1−LTBP1
CNVR2766.1−BMP6
CNVR6609.1−CREBBP
CNVR368.1−THBS3
CNVR7889.1−BMP7
CNVR6609.5−CREBBP
CNVR683.1−ROCK2
CNVR7150.1−RPS6KB1
CNVR369.1−THBS3
CNVR3194.1−THBS2
CNVR3194.2−THBS2
CNVR6609.3−CREBBP
CNVR5541.1−SP1
CNVR6056.1−TFDP1
CNVR2767.1−BMP6
CNVR7838.1−RBL1
CNVR2006.1−BMPR1B
CNVR6609.6−CREBBP
CNVR1656.1−CHRD
CNVR3504.1−SMURF1
CNVR368.2−THBS3
CNVR3610.1−CUL1
CNVR6609.4−CREBBP
CNVR176.1−ZFYVE9
CNVR4836.1−BMPR1A
CNVR2804.1−ID4
CNVR148.1−BMP8A
CNVR1110.1−BMPR2
YRI
CEU
CHB
0.0
0.2
0.4
0.6
0.8

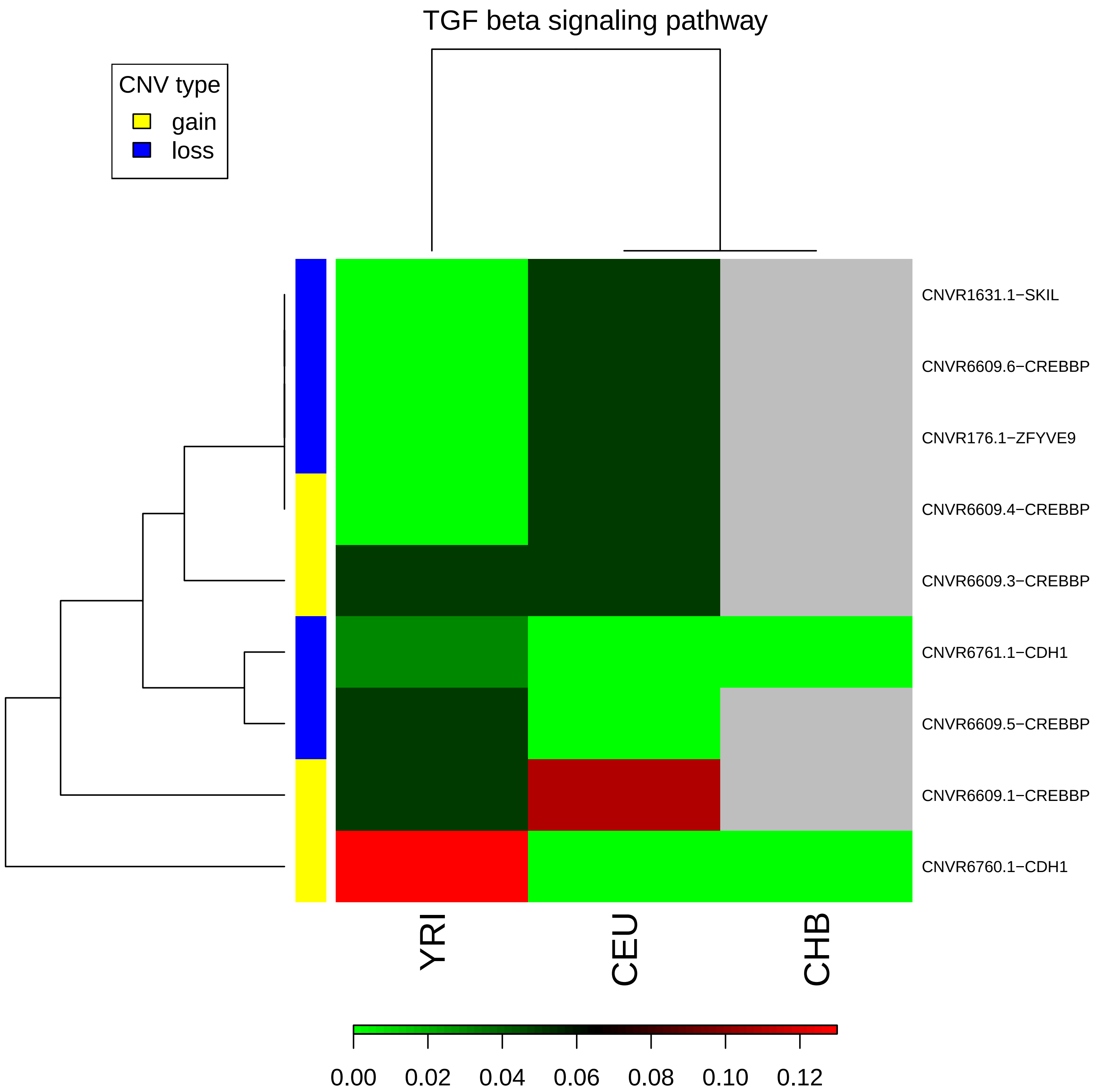

TGF beta signaling pathway
CNV type
gain
loss
CNVR1631.1−SKIL
CNVR6609.6−CREBBP
CNVR176.1−ZFYVE9
CNVR6609.4−CREBBP
CNVR6609.3−CREBBP
CNVR6761.1−CDH1
CNVR6609.5−CREBBP
CNVR6609.1−CREBBP
CNVR6760.1−CDH1
YRI
CEU
CHB
0.00
0.02
0.04
0.06
0.08
0.10
0.12

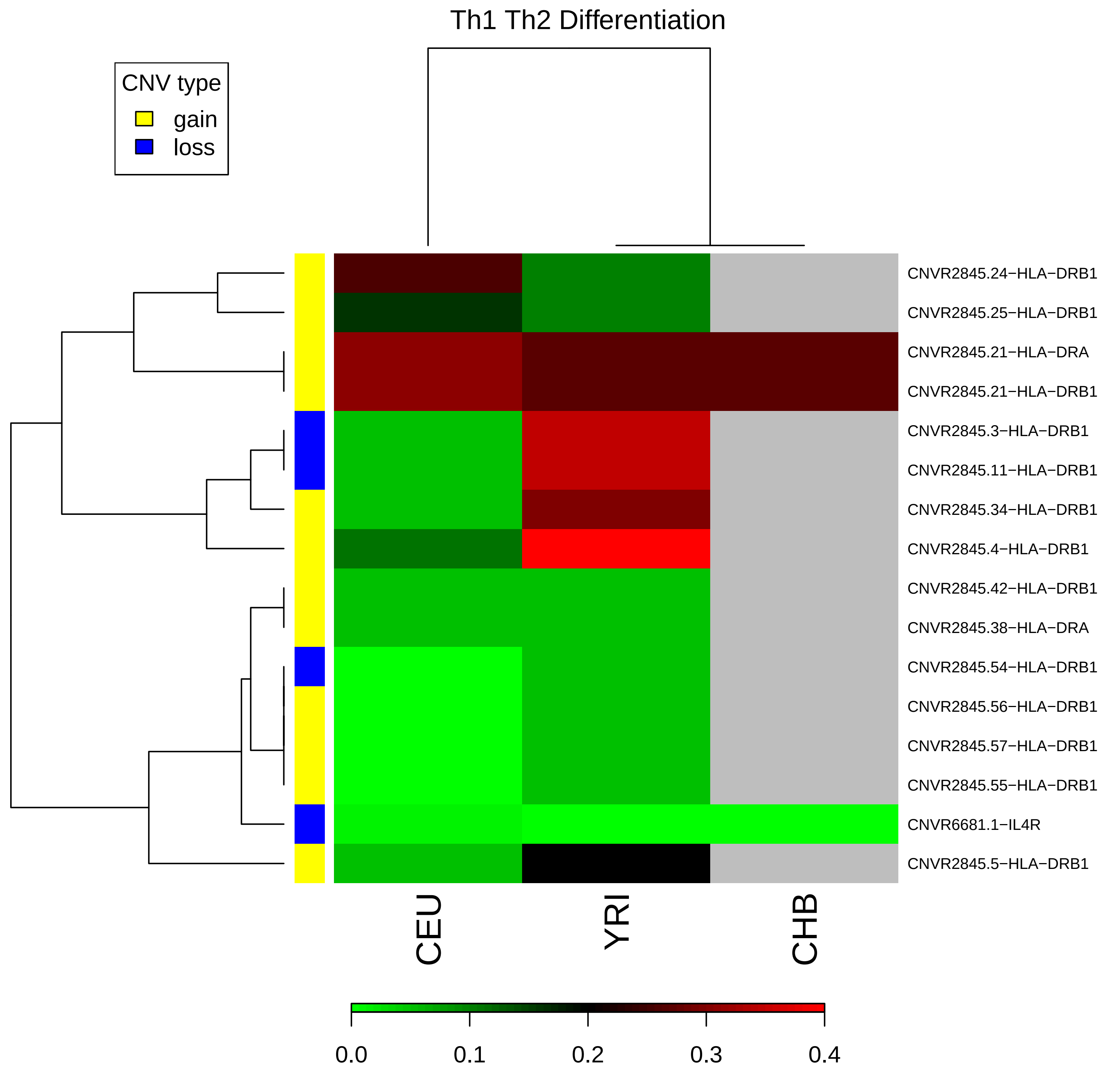

Th1 Th2 Differentiation
CNV type
gain
loss
CNVR2845.24−HLA−DRB1
CNVR2845.25−HLA−DRB1
CNVR2845.21−HLA−DRA
CNVR2845.21−HLA−DRB1
CNVR2845.3−HLA−DRB1
CNVR2845.11−HLA−DRB1
CNVR2845.34−HLA−DRB1
CNVR2845.4−HLA−DRB1
CNVR2845.42−HLA−DRB1
CNVR2845.38−HLA−DRA
CNVR2845.54−HLA−DRB1
CNVR2845.56−HLA−DRB1
CNVR2845.57−HLA−DRB1
CNVR2845.55−HLA−DRB1
CNVR6681.1−IL4R
CNVR2845.5−HLA−DRB1
CEU
YRI
CHB
0.0
0.1
0.2
0.3
0.4

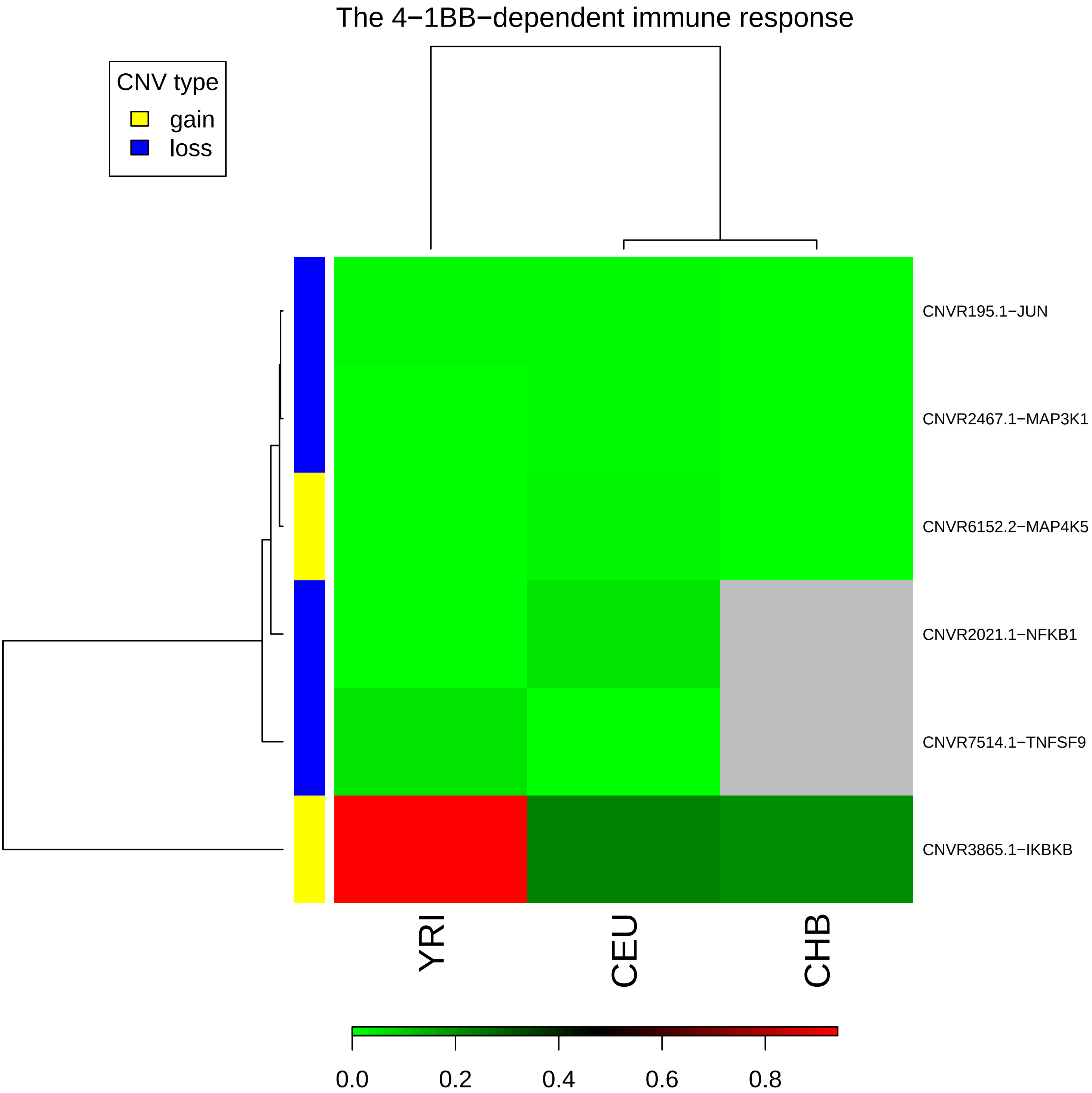

The 4−1BB−dependent immune response
CNV type
gain
loss
CNVR195.1−JUN
CNVR2467.1−MAP3K1
CNVR6152.2−MAP4K5
CNVR2021.1−NFKB1
CNVR7514.1−TNFSF9
CNVR3865.1−IKBKB
YRI
CEU
CHB
0.0
0.2
0.4
0.6
0.8

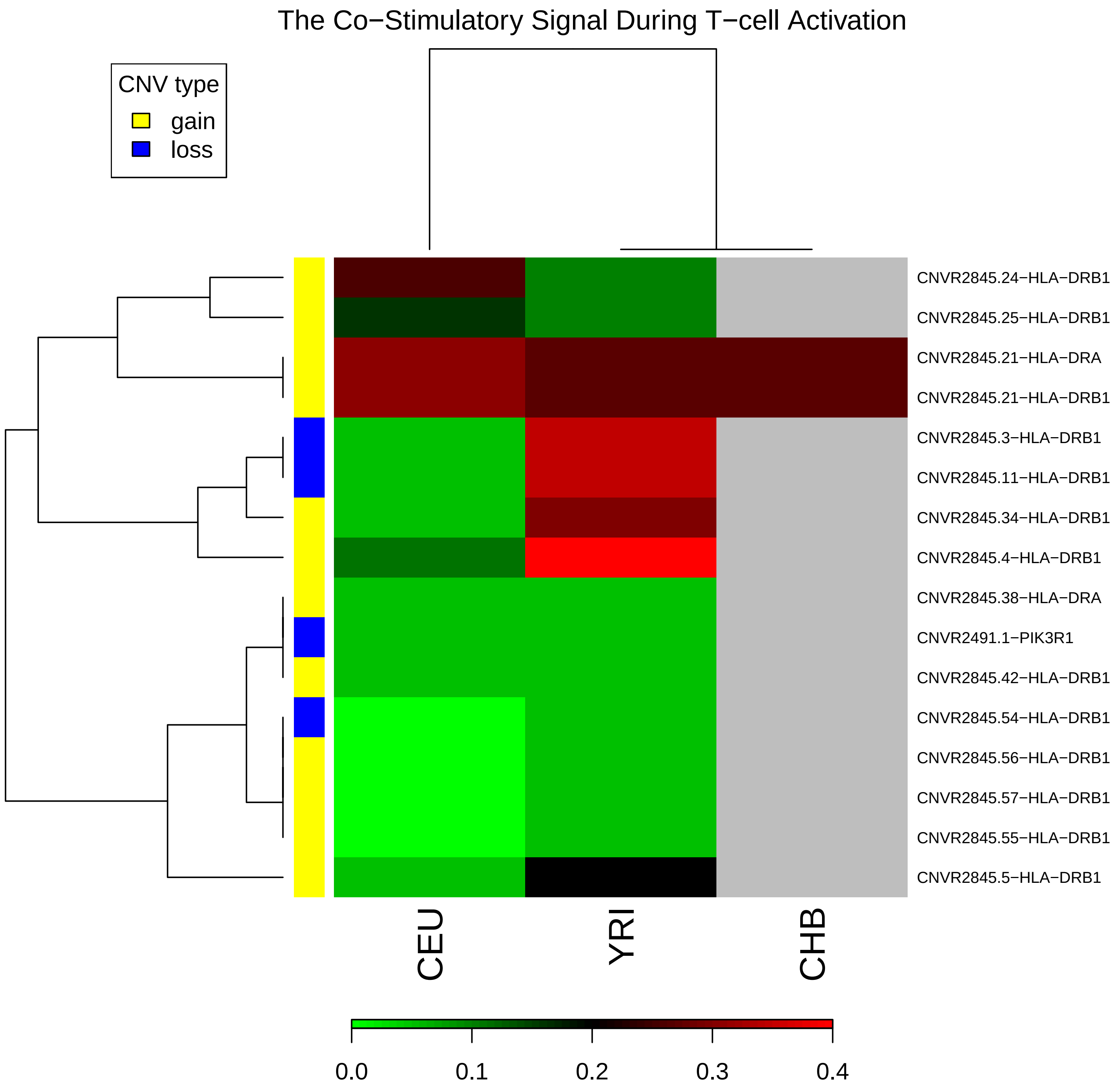

The Co−Stimulatory Signal During T−cell Activation
CNV type
gain
loss
CNVR2845.24−HLA−DRB1
CNVR2845.25−HLA−DRB1
CNVR2845.21−HLA−DRA
CNVR2845.21−HLA−DRB1
CNVR2845.3−HLA−DRB1
CNVR2845.11−HLA−DRB1
CNVR2845.34−HLA−DRB1
CNVR2845.4−HLA−DRB1
CNVR2845.38−HLA−DRA
CNVR2491.1−PIK3R1
CNVR2845.42−HLA−DRB1
CNVR2845.54−HLA−DRB1
CNVR2845.56−HLA−DRB1
CNVR2845.57−HLA−DRB1
CNVR2845.55−HLA−DRB1
CNVR2845.5−HLA−DRB1
CEU
YRI
CHB
0.0
0.1
0.2
0.3
0.4

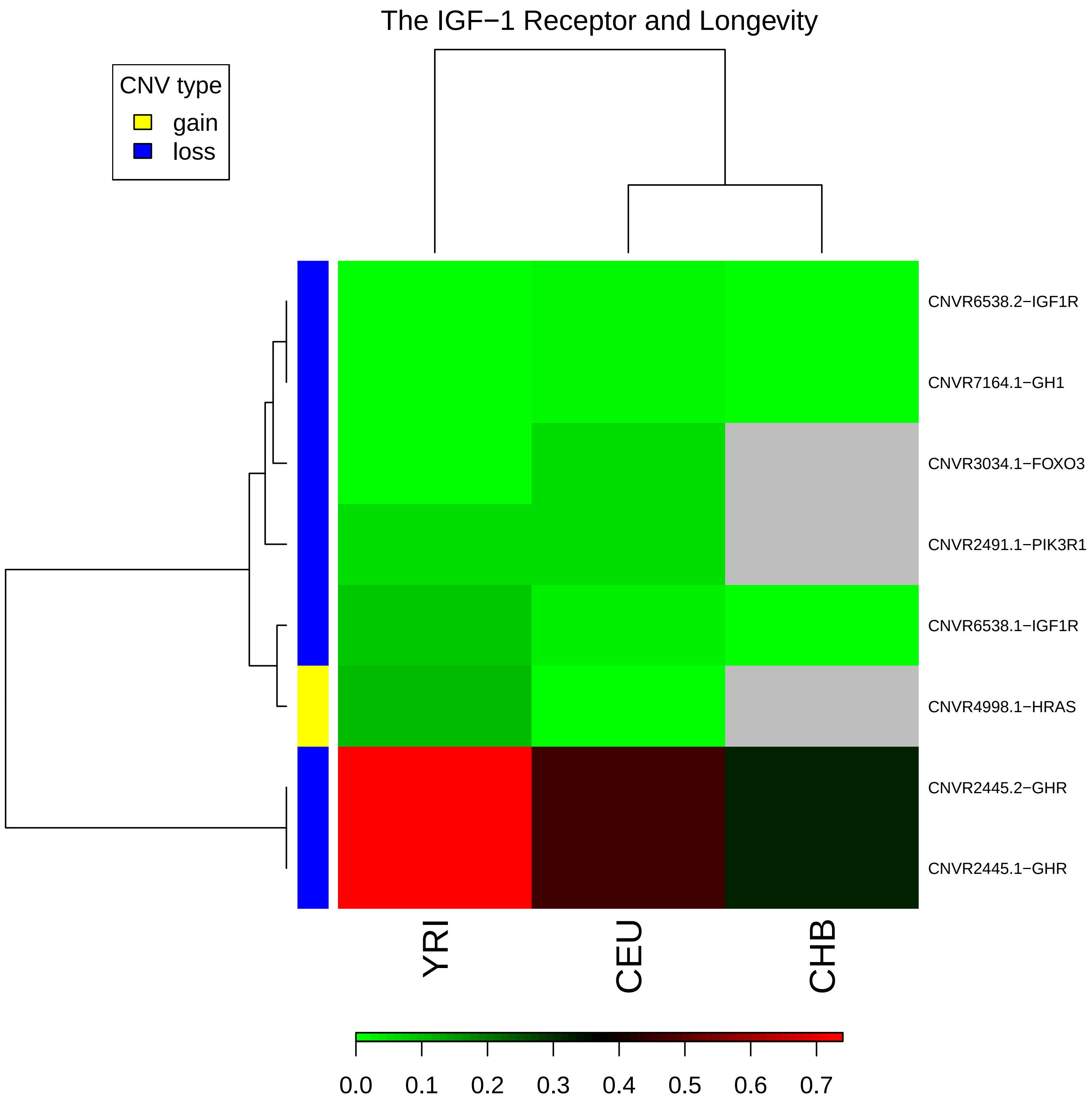
The IGF−1 Receptor and Longevity
CNV type
gain
loss
CNVR6538.2−IGF1R
CNVR7164.1−GH1
CNVR3034.1−FOXO3
CNVR2491.1−PIK3R1
CNVR6538.1−IGF1R
CNVR4998.1−HRAS
CNVR2445.2−GHR
CNVR2445.1−GHR
YRI
CEU
CHB
0.0
0.1
0.2
0.3
0.4
0.5
0.6
0.7

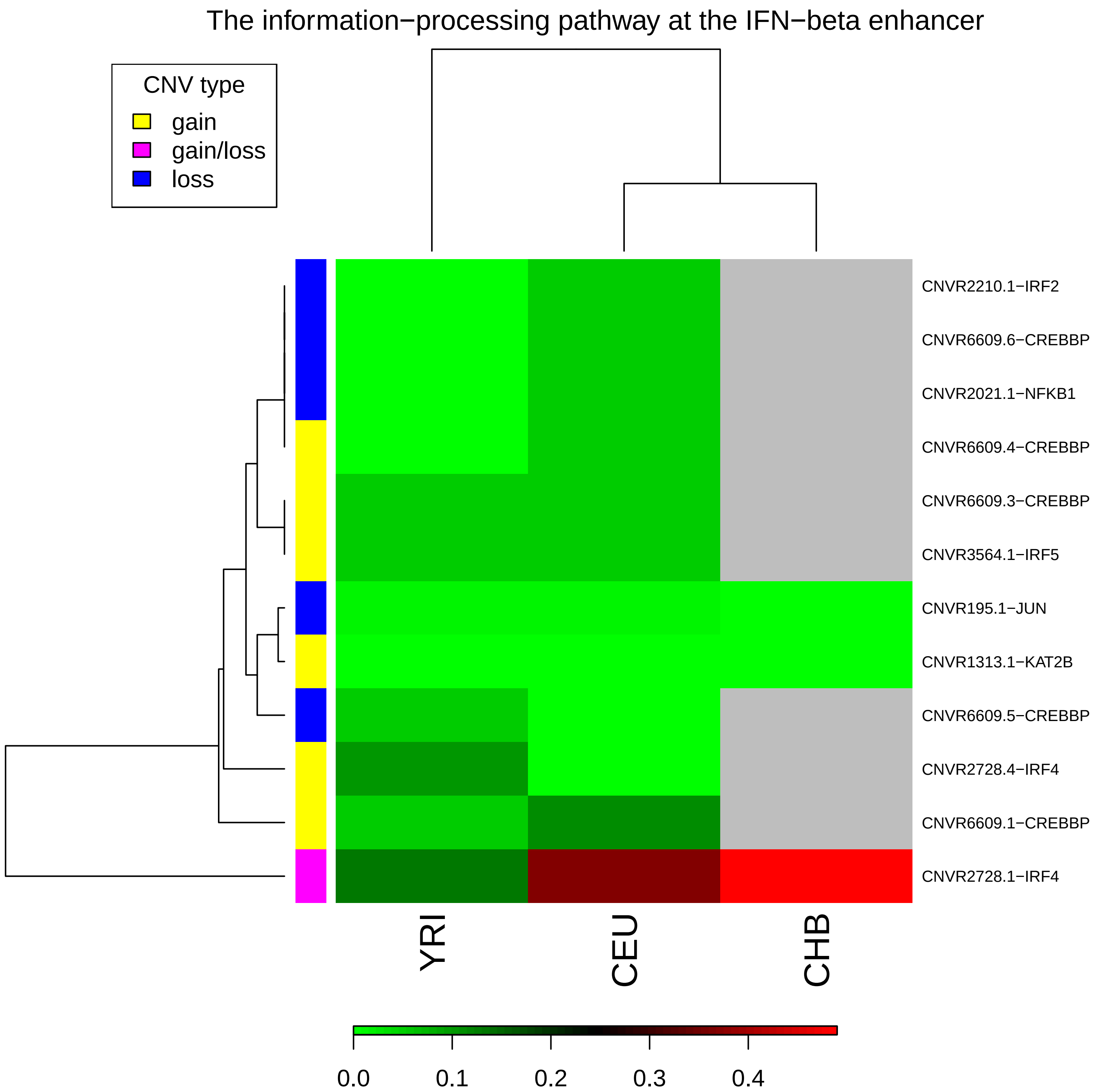

The information−processing pathway at the IFN−beta enhancer
CNV type
gain
gain/loss
loss
CNVR2210.1−IRF2
CNVR6609.6−CREBBP
CNVR2021.1−NFKB1
CNVR6609.4−CREBBP
CNVR6609.3−CREBBP
CNVR3564.1−IRF5
CNVR195.1−JUN
CNVR1313.1−KAT2B
CNVR6609.5−CREBBP
CNVR2728.4−IRF4
CNVR6609.1−CREBBP
CNVR2728.1−IRF4
YRI
CEU
CHB
0.0
0.1
0.2
0.3
0.4

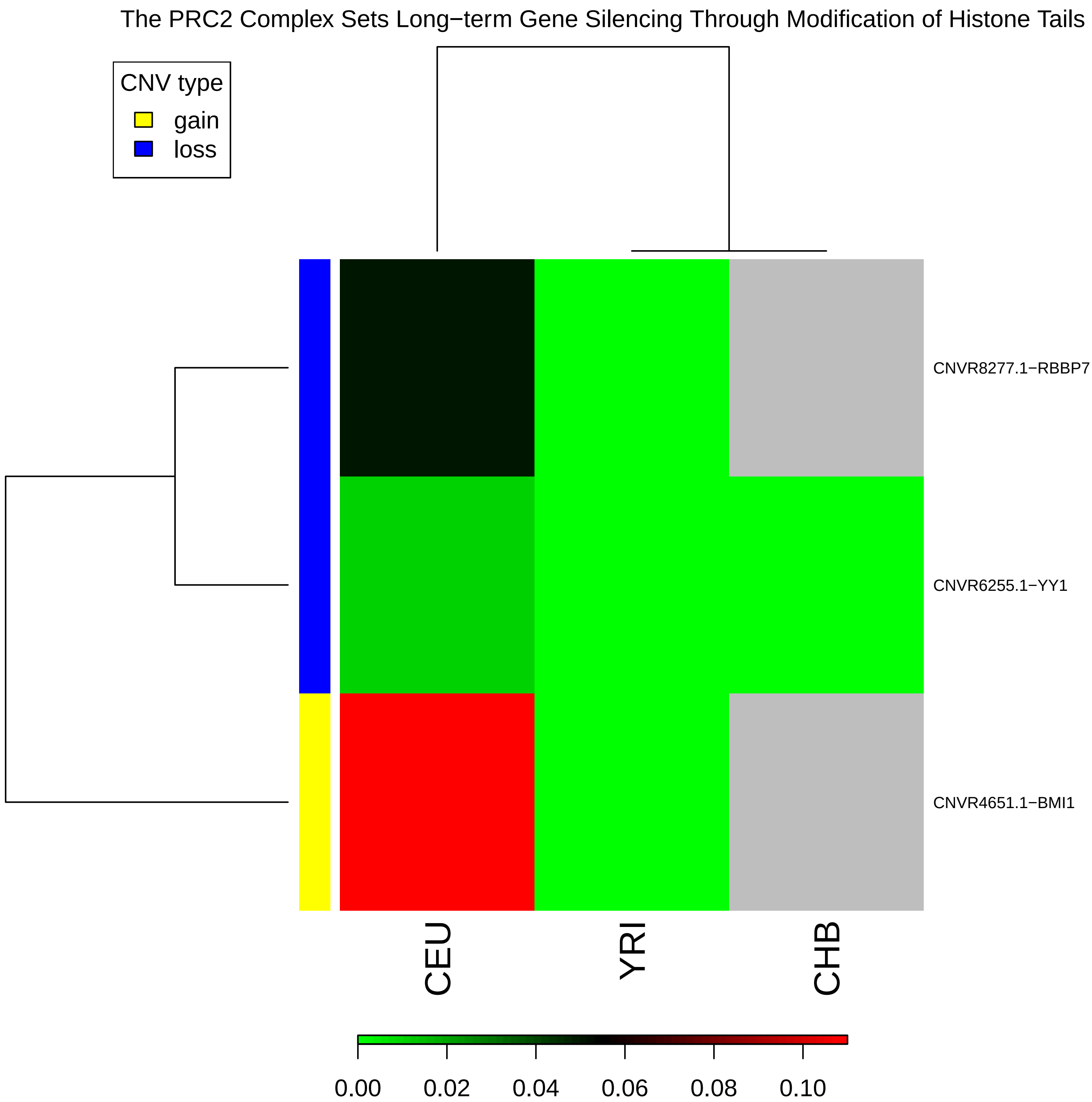
The PRC2 Complex Sets Long−term Gene Silencing Through Modification of Histone Tails
CNV type
gain
loss
CNVR8277.1−RBBP7
CNVR6255.1−YY1
CNVR4651.1−BMI1
CEU
YRI
CHB
0.00
0.02
0.04
0.06
0.08
0.10

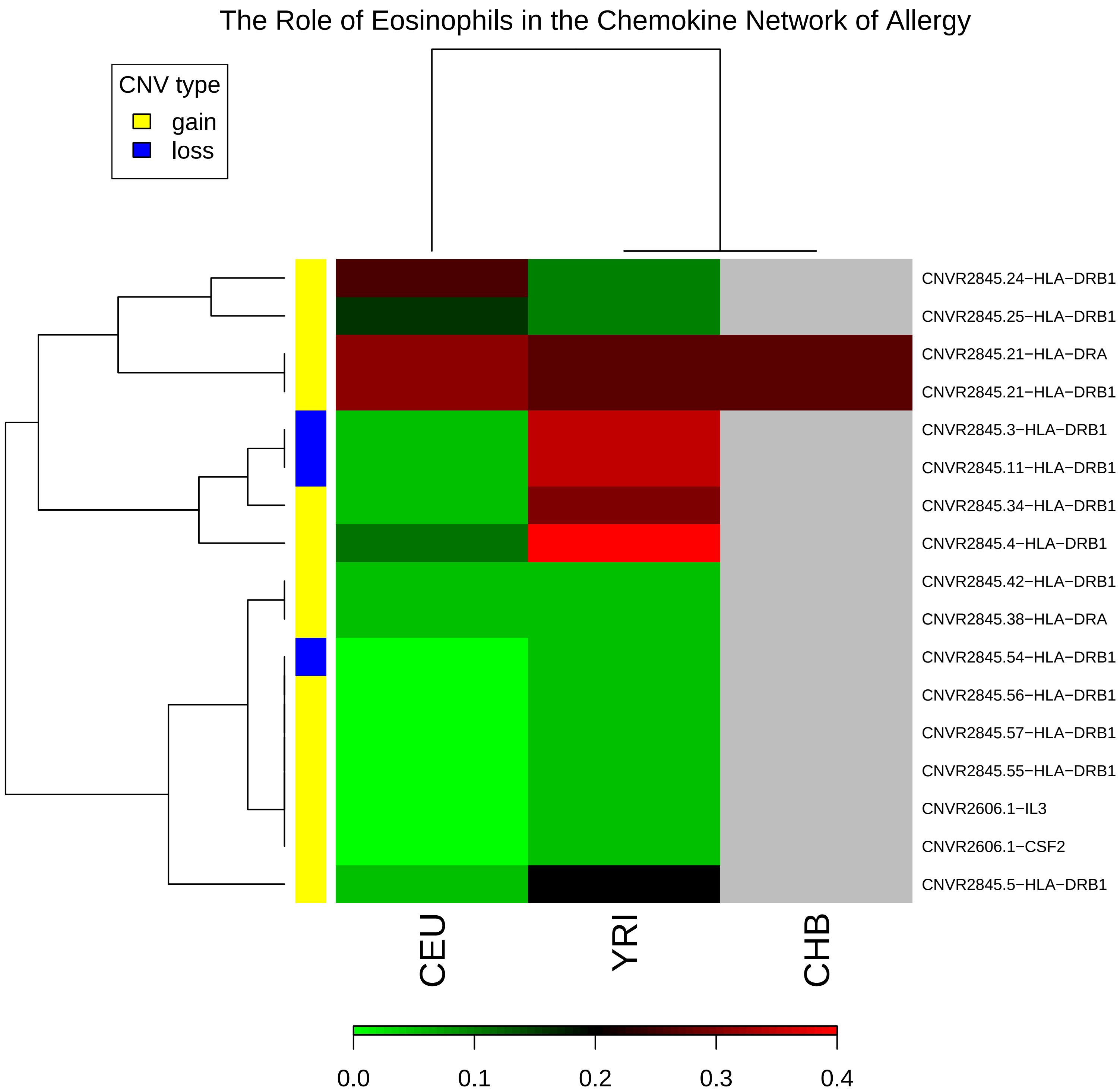
The Role of Eosinophils in the Chemokine Network of Allergy
CNV type
gain
loss
CNVR2845.24−HLA−DRB1
CNVR2845.25−HLA−DRB1
CNVR2845.21−HLA−DRA
CNVR2845.21−HLA−DRB1
CNVR2845.3−HLA−DRB1
CNVR2845.11−HLA−DRB1
CNVR2845.34−HLA−DRB1
CNVR2845.4−HLA−DRB1
CNVR2845.42−HLA−DRB1
CNVR2845.38−HLA−DRA
CNVR2845.54−HLA−DRB1
CNVR2845.56−HLA−DRB1
CNVR2845.57−HLA−DRB1
CNVR2845.55−HLA−DRB1
CNVR2606.1−IL3
CNVR2606.1−CSF2
CNVR2845.5−HLA−DRB1
CEU
YRI
CHB
0.0
0.1
0.2
0.3
0.4

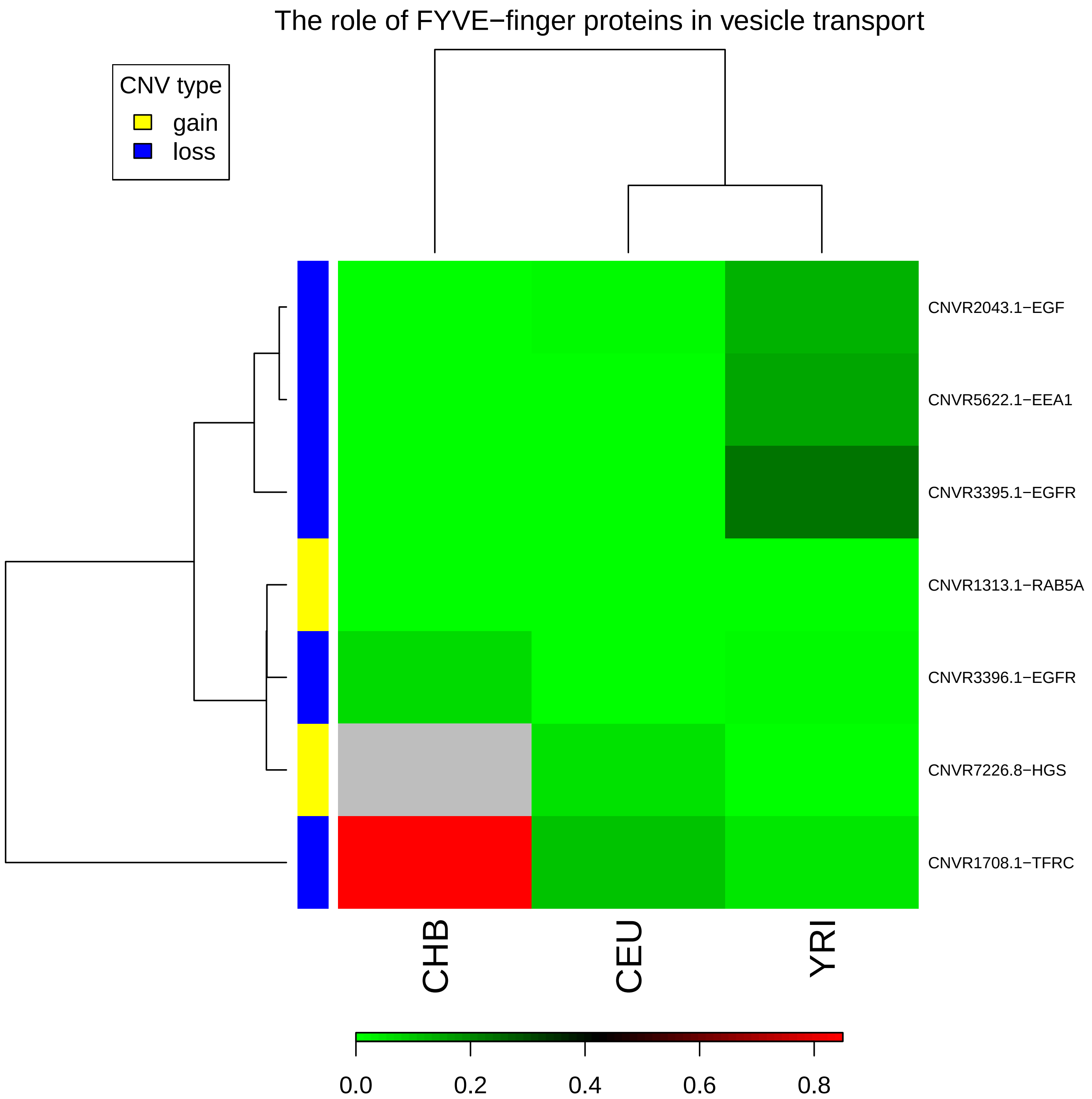

The role of FYVE−finger proteins in vesicle transport
CNV type
gain
loss
CNVR2043.1−EGF
CNVR5622.1−EEA1
CNVR3395.1−EGFR
CNVR1313.1−RAB5A
CNVR3396.1−EGFR
CNVR7226.8−HGS
CNVR1708.1−TFRC
CHB
CEU
YRI
0.0
0.2
0.4
0.6
0.8

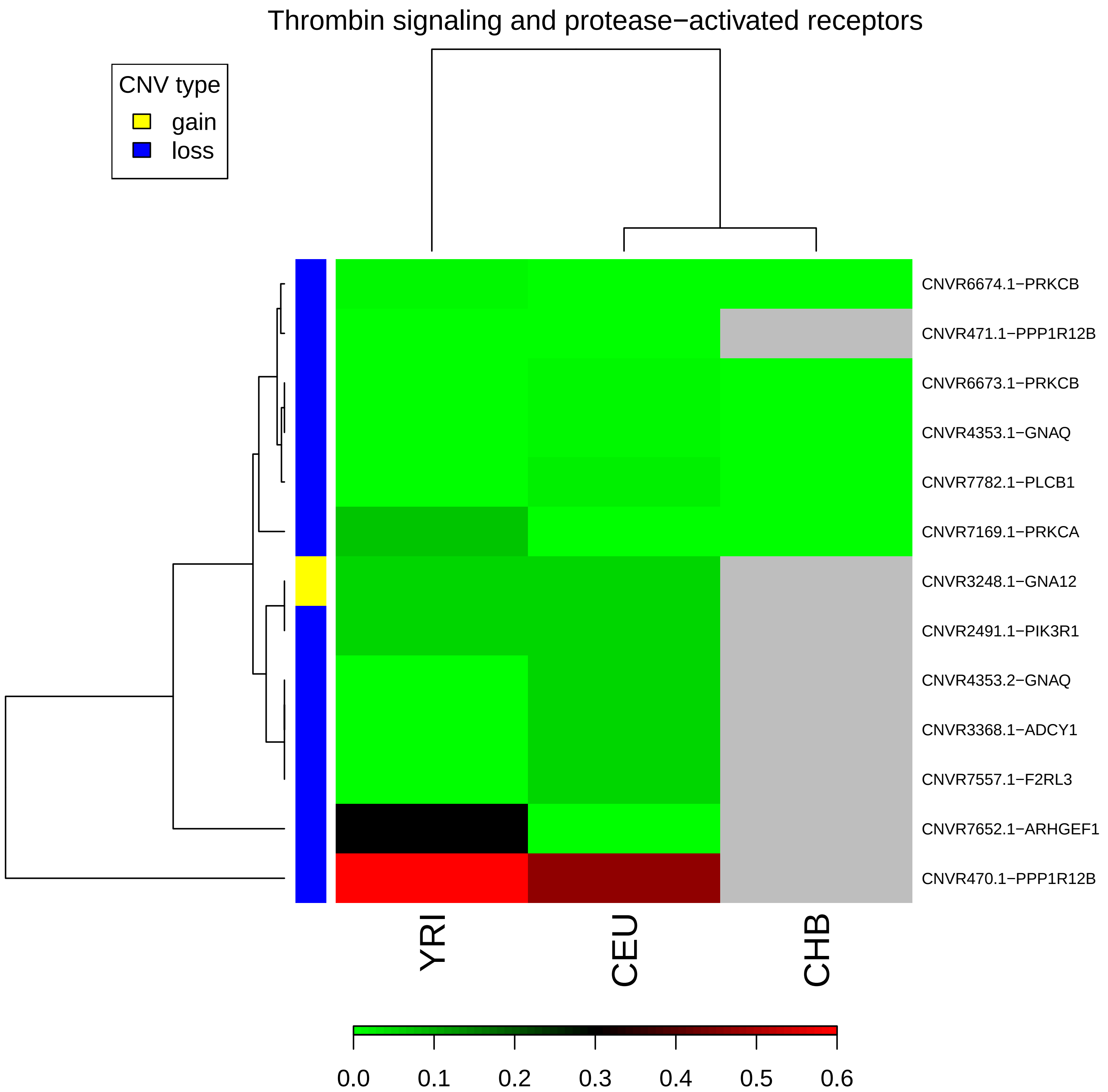

Thrombin signaling and protease−activated receptors
CNV type
gain
loss
CNVR6674.1−PRKCB
CNVR471.1−PPP1R12B
CNVR6673.1−PRKCB
CNVR4353.1−GNAQ
CNVR7782.1−PLCB1
CNVR7169.1−PRKCA
CNVR3248.1−GNA12
CNVR2491.1−PIK3R1
CNVR4353.2−GNAQ
CNVR3368.1−ADCY1
CNVR7557.1−F2RL3
CNVR7652.1−ARHGEF1
CNVR470.1−PPP1R12B
YRI
CEU
CHB
0.0
0.1
0.2
0.3
0.4
0.5
0.6

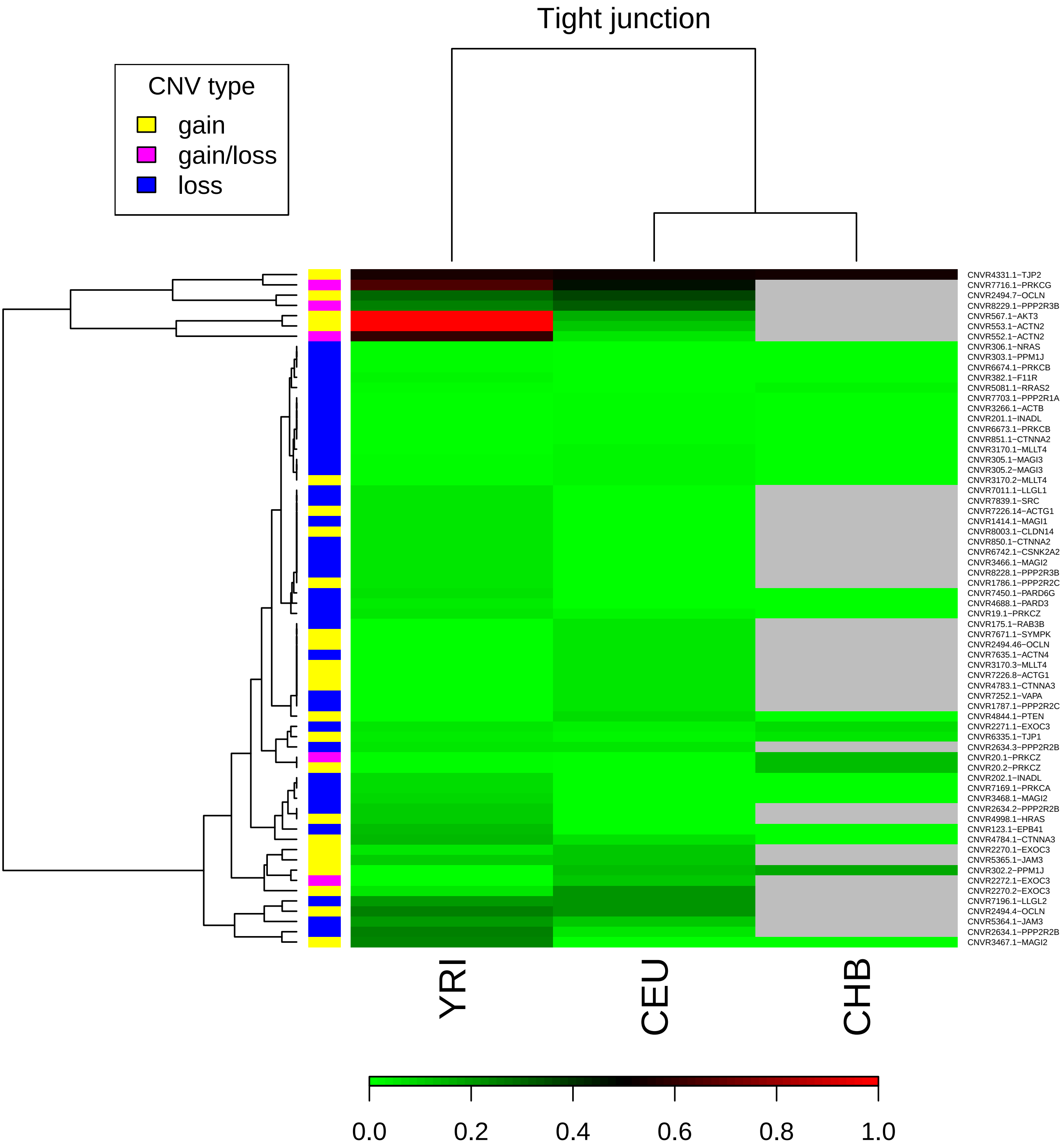
Tight junction
CNV type
gain
gain/loss
loss
CNVR4331.1−TJP2
CNVR7716.1−PRKCG
CNVR2494.7−OCLN
CNVR8229.1−PPP2R3B
CNVR567.1−AKT3
CNVR553.1−ACTN2
CNVR552.1−ACTN2
CNVR306.1−NRAS
CNVR303.1−PPM1J
CNVR6674.1−PRKCB
CNVR382.1−F11R
CNVR5081.1−RRAS2
CNVR7703.1−PPP2R1A
CNVR3266.1−ACTB
CNVR201.1−INADL
CNVR6673.1−PRKCB
CNVR851.1−CTNNA2
CNVR3170.1−MLLT4
CNVR305.1−MAGI3
CNVR305.2−MAGI3
CNVR3170.2−MLLT4
CNVR7011.1−LLGL1
CNVR7839.1−SRC
CNVR7226.14−ACTG1
CNVR1414.1−MAGI1
CNVR8003.1−CLDN14
CNVR850.1−CTNNA2
CNVR6742.1−CSNK2A2
CNVR3466.1−MAGI2
CNVR8228.1−PPP2R3B
CNVR1786.1−PPP2R2C
CNVR7450.1−PARD6G
CNVR4688.1−PARD3
CNVR19.1−PRKCZ
CNVR175.1−RAB3B
CNVR7671.1−SYMPK
CNVR2494.46−OCLN
CNVR7635.1−ACTN4
CNVR3170.3−MLLT4
CNVR7226.8−ACTG1
CNVR4783.1−CTNNA3
CNVR7252.1−VAPA
CNVR1787.1−PPP2R2C
CNVR4844.1−PTEN
CNVR2271.1−EXOC3
CNVR6335.1−TJP1
CNVR2634.3−PPP2R2B
CNVR20.1−PRKCZ
CNVR20.2−PRKCZ
CNVR202.1−INADL
CNVR7169.1−PRKCA
CNVR3468.1−MAGI2
CNVR2634.2−PPP2R2B
CNVR4998.1−HRAS
CNVR123.1−EPB41
CNVR4784.1−CTNNA3
CNVR2270.1−EXOC3
CNVR5365.1−JAM3
CNVR302.2−PPM1J
CNVR2272.1−EXOC3
CNVR2270.2−EXOC3
CNVR7196.1−LLGL2
CNVR2494.4−OCLN
CNVR5364.1−JAM3
CNVR2634.1−PPP2R2B
CNVR3467.1−MAGI2
YRI
CEU
CHB
0.0
0.2
0.4
0.6
0.8
1.0

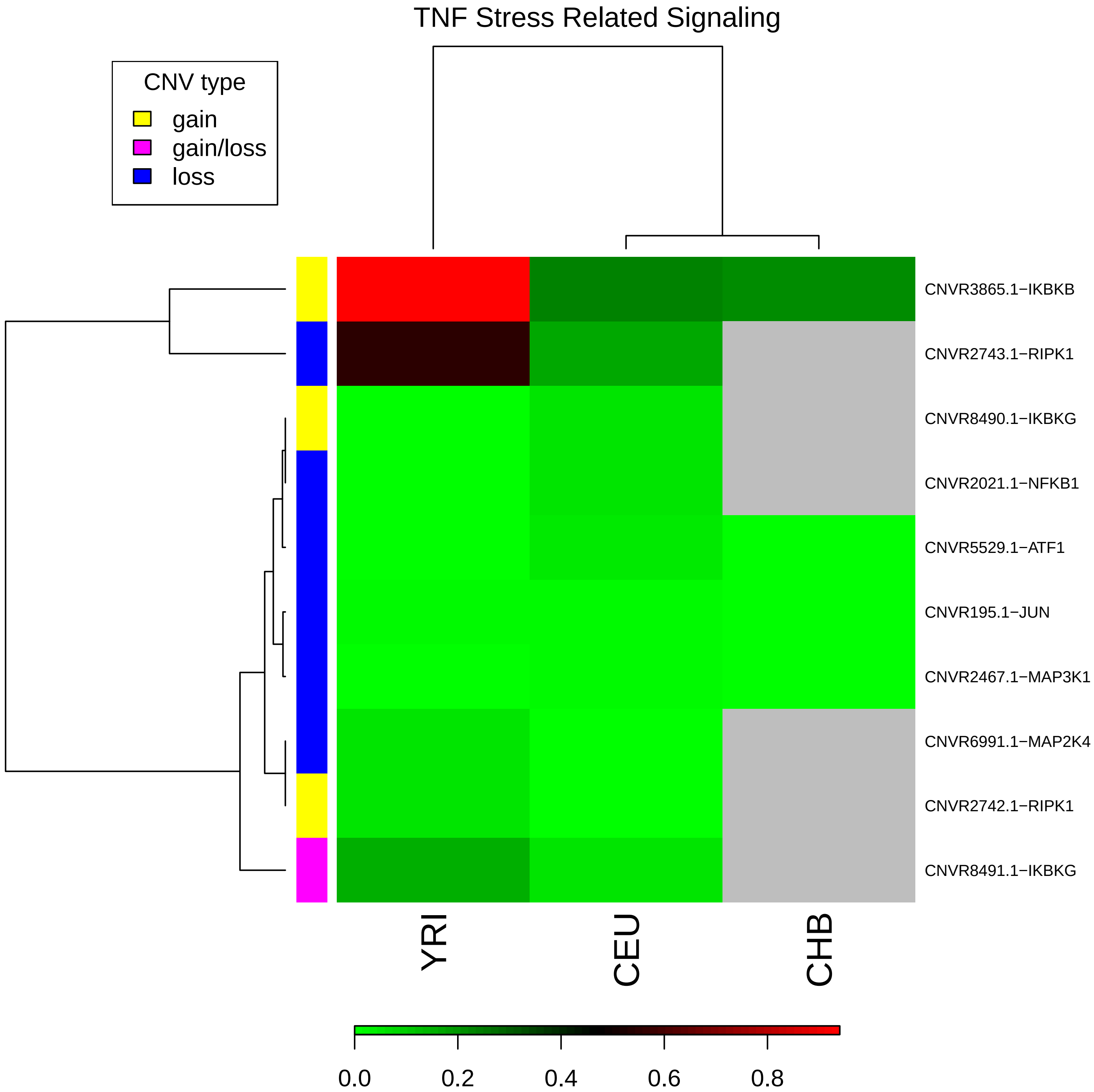
TNF Stress Related Signaling
CNV type
gain
gain/loss
loss
CNVR3865.1−IKBKB
CNVR2743.1−RIPK1
CNVR8490.1−IKBKG
CNVR2021.1−NFKB1
CNVR5529.1−ATF1
CNVR195.1−JUN
CNVR2467.1−MAP3K1
CNVR6991.1−MAP2K4
CNVR2742.1−RIPK1
CNVR8491.1−IKBKG
YRI
CEU
CHB
0.0
0.2
0.4
0.6
0.8

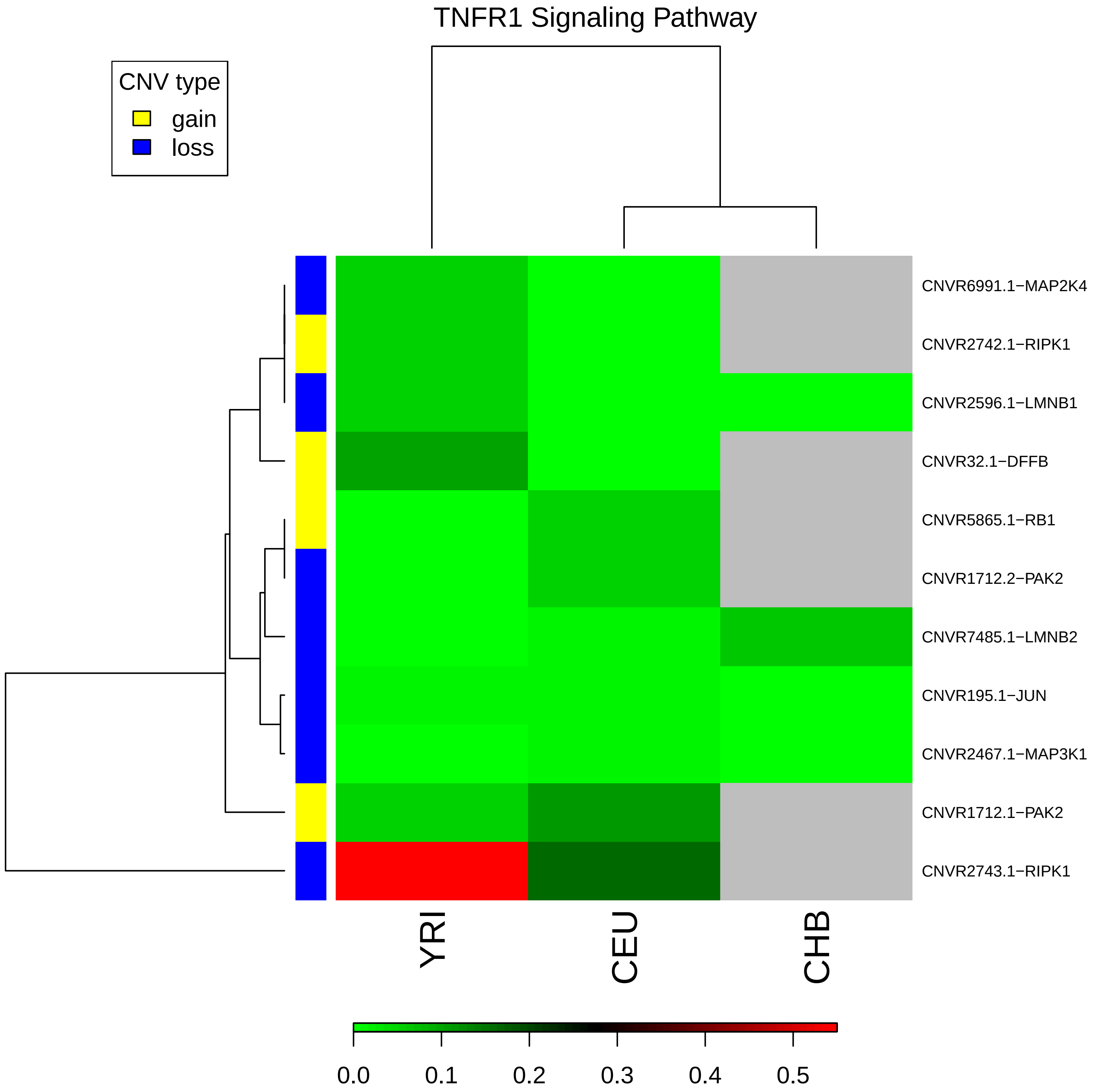

TNFR1 Signaling Pathway
CNV type
gain
loss
CNVR6991.1−MAP2K4
CNVR2742.1−RIPK1
CNVR2596.1−LMNB1
CNVR32.1−DFFB
CNVR5865.1−RB1
CNVR1712.2−PAK2
CNVR7485.1−LMNB2
CNVR195.1−JUN
CNVR2467.1−MAP3K1
CNVR1712.1−PAK2
CNVR2743.1−RIPK1
YRI
CEU
CHB
0.0
0.1
0.2
0.3
0.4
0.5

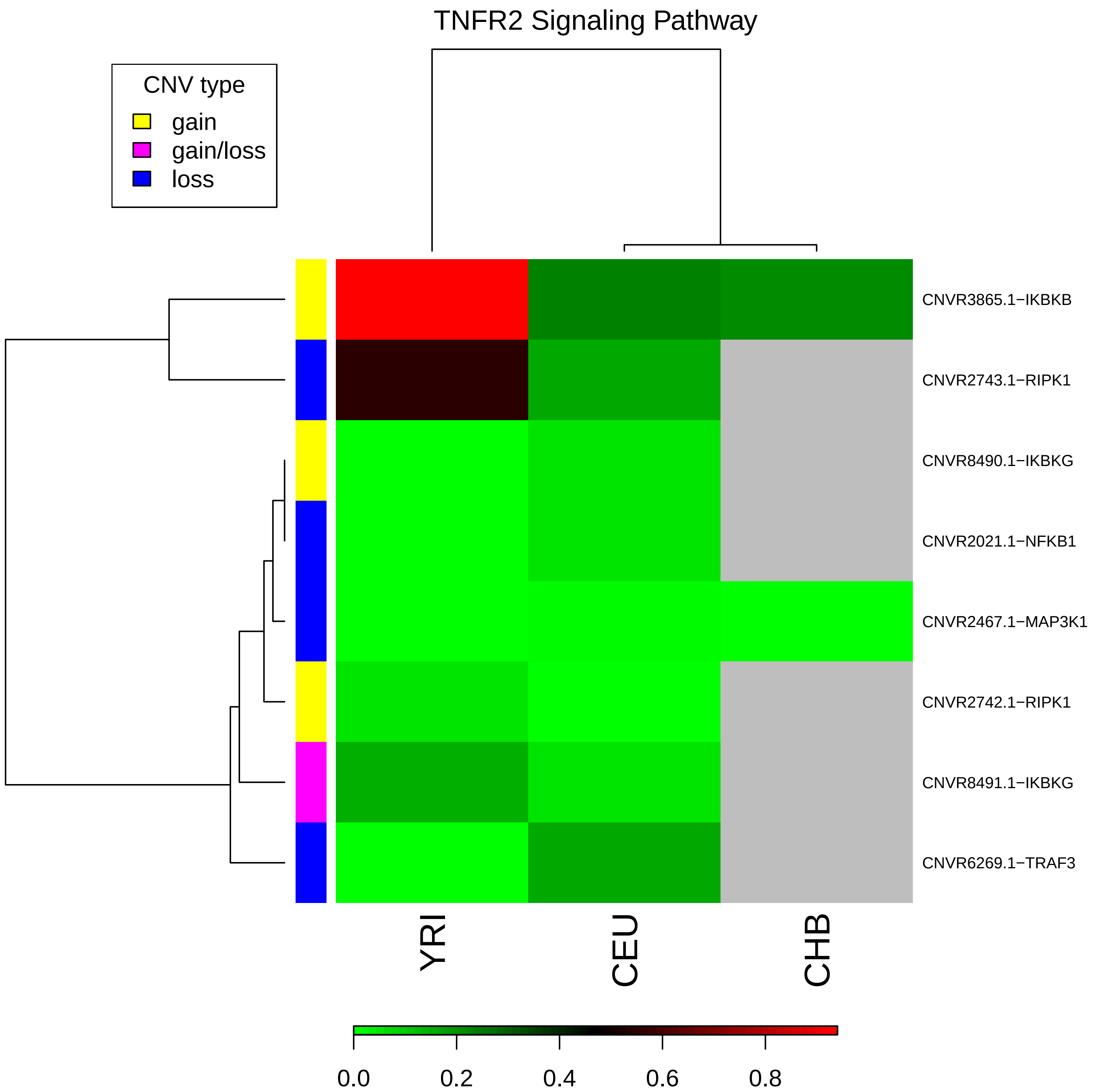
TNFR2 Signaling Pathway
CNV type
gain
gain/loss
loss
CNVR3865.1−IKBKB
CNVR2743.1−RIPK1
CNVR8490.1−IKBKG
CNVR2021.1−NFKB1
CNVR2467.1−MAP3K1
CNVR2742.1−RIPK1
CNVR8491.1−IKBKG
CNVR6269.1−TRAF3
YRI
CEU
CHB
0.0
0.2
0.4
0.6
0.8

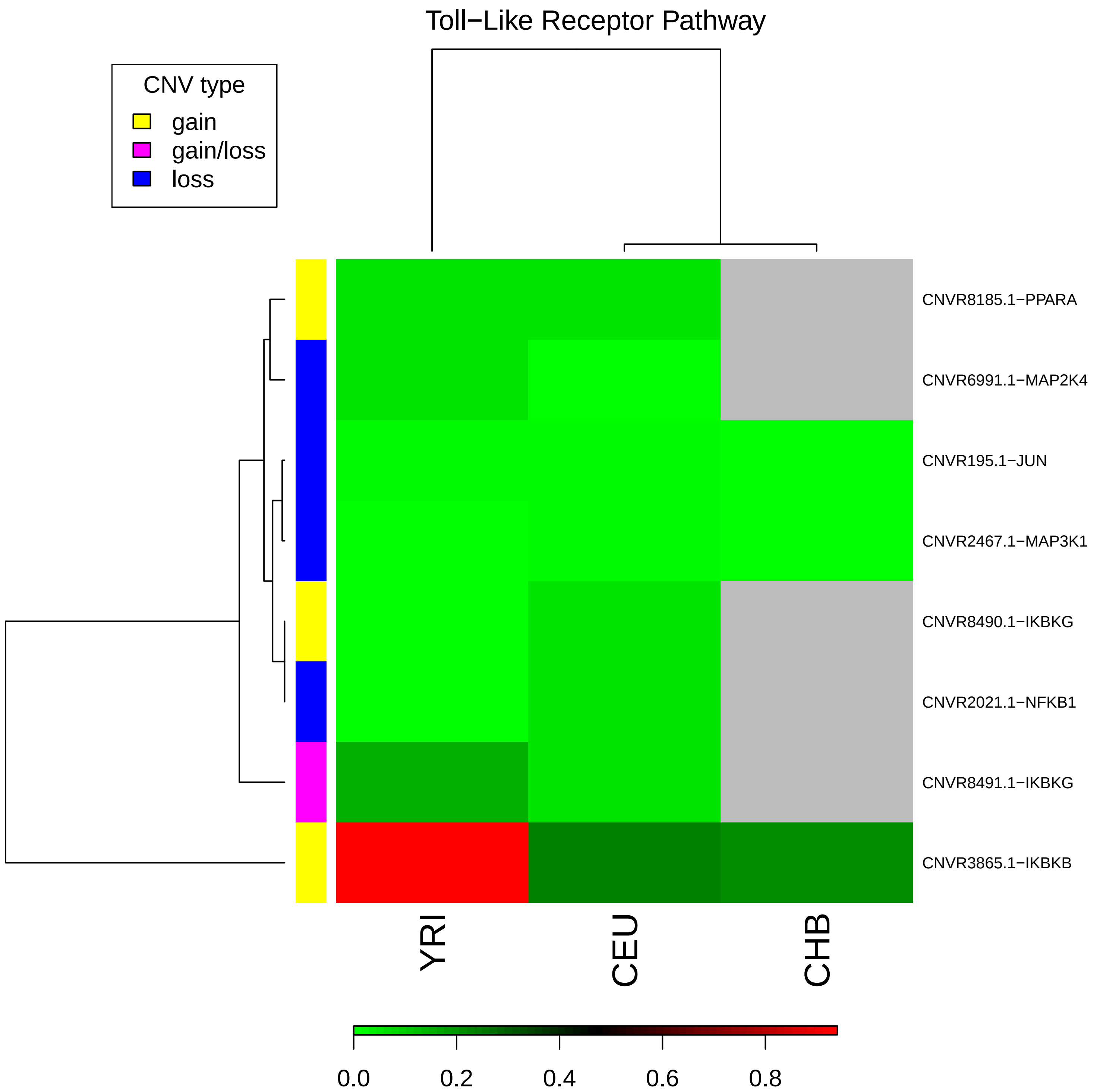
Toll−Like Receptor Pathway
CNV type
gain
gain/loss
loss
CNVR8185.1−PPARA
CNVR6991.1−MAP2K4
CNVR195.1−JUN
CNVR2467.1−MAP3K1
CNVR8490.1−IKBKG
CNVR2021.1−NFKB1
CNVR8491.1−IKBKG
CNVR3865.1−IKBKB
YRI
CEU
CHB
0.0
0.2
0.4
0.6
0.8

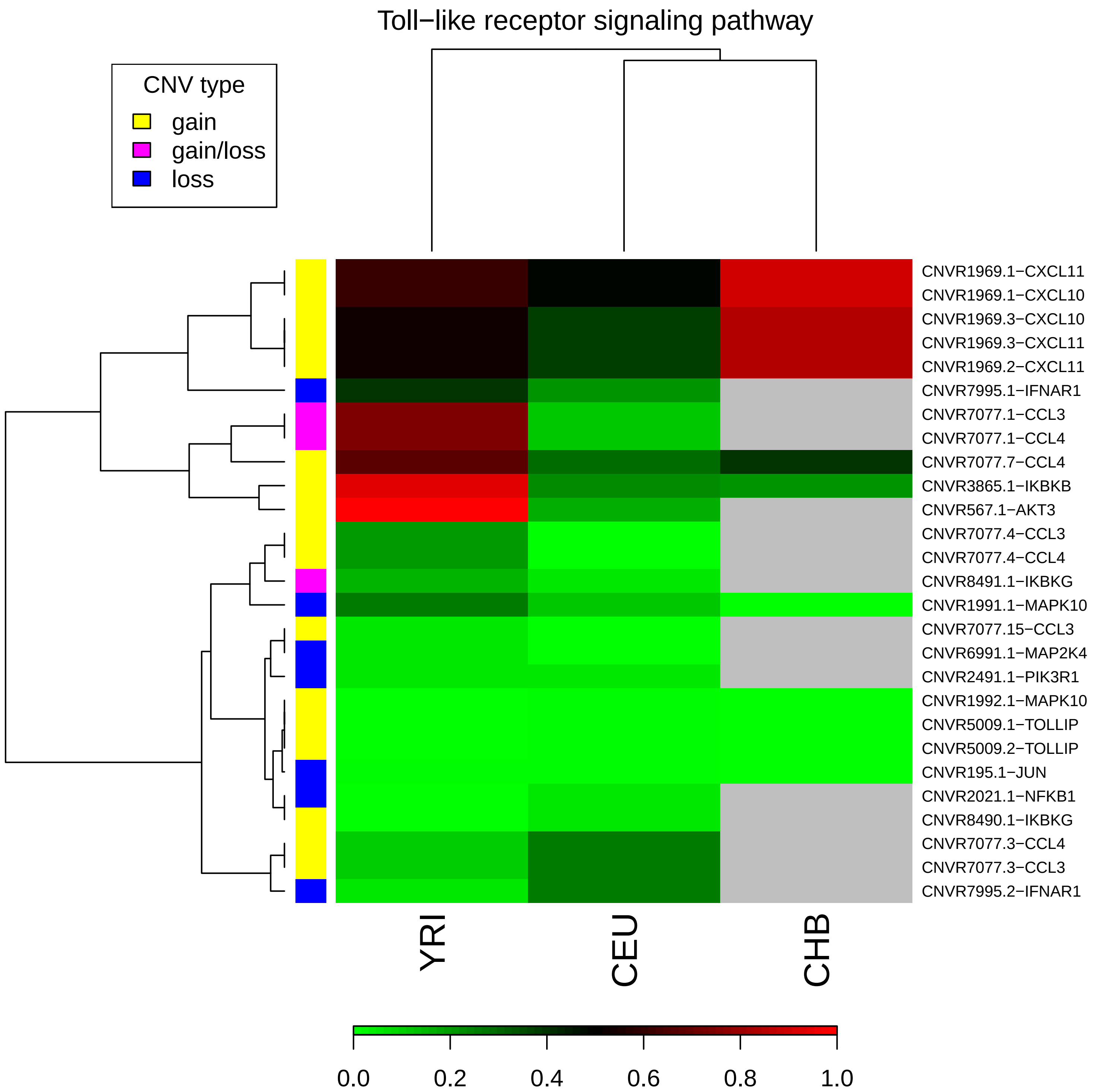
Toll−like receptor signaling pathway
CNV type
gain
gain/loss
loss
CNVR1969.1−CXCL11
CNVR1969.1−CXCL10
CNVR1969.3−CXCL10
CNVR1969.3−CXCL11
CNVR1969.2−CXCL11
CNVR7995.1−IFNAR1
CNVR7077.1−CCL3
CNVR7077.1−CCL4
CNVR7077.7−CCL4
CNVR3865.1−IKBKB
CNVR567.1−AKT3
CNVR7077.4−CCL3
CNVR7077.4−CCL4
CNVR8491.1−IKBKG
CNVR1991.1−MAPK10
CNVR7077.15−CCL3
CNVR6991.1−MAP2K4
CNVR2491.1−PIK3R1
CNVR1992.1−MAPK10
CNVR5009.1−TOLLIP
CNVR5009.2−TOLLIP
CNVR195.1−JUN
CNVR2021.1−NFKB1
CNVR8490.1−IKBKG
CNVR7077.3−CCL4
CNVR7077.3−CCL3
CNVR7995.2−IFNAR1
YRI
CEU
CHB
0.0
0.2
0.4
0.6
0.8
1.0

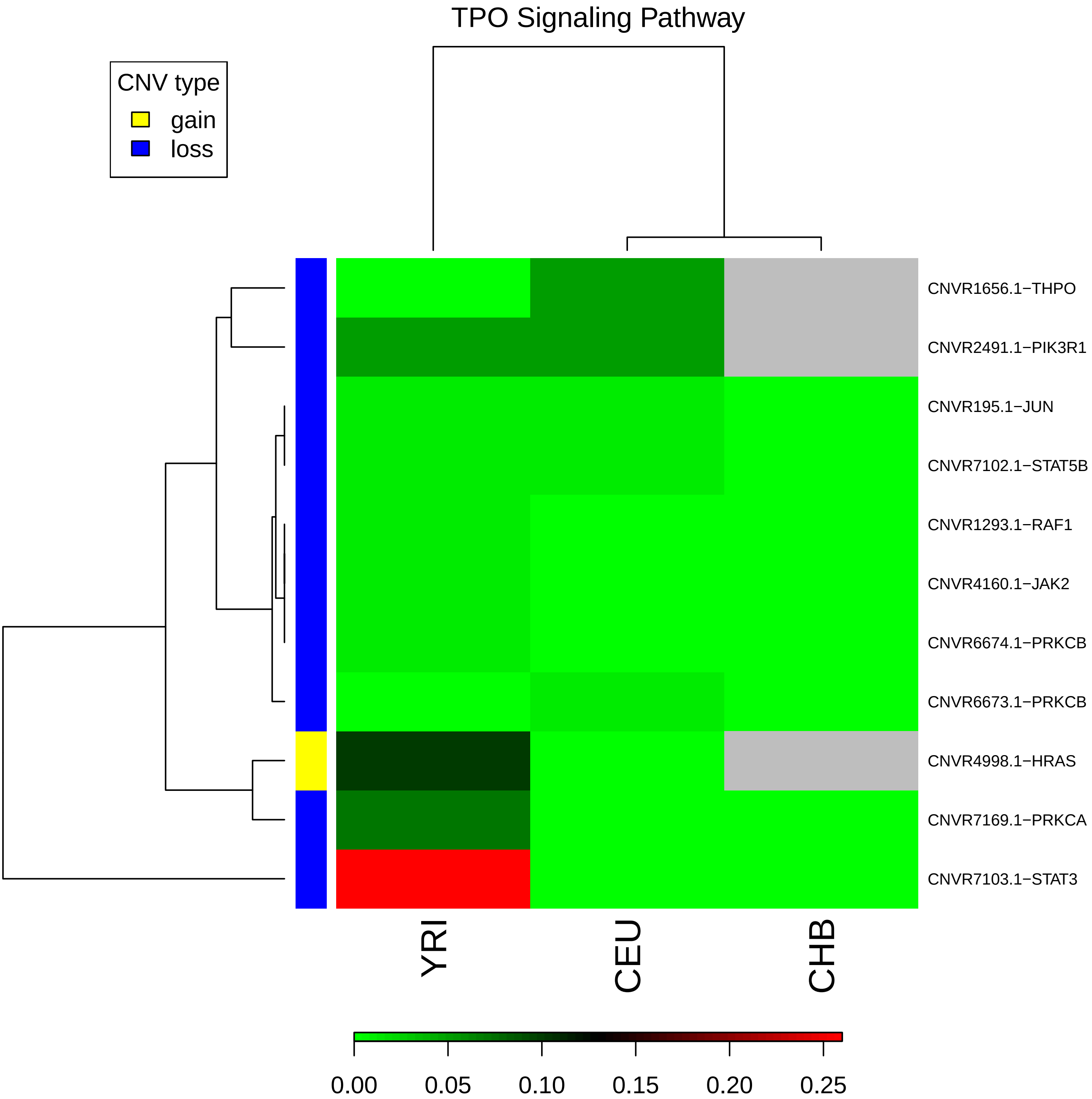
TPO Signaling Pathway
CNV type
gain
loss
CNVR1656.1−THPO
CNVR2491.1−PIK3R1
CNVR195.1−JUN
CNVR7102.1−STAT5B
CNVR1293.1−RAF1
CNVR4160.1−JAK2
CNVR6674.1−PRKCB
CNVR6673.1−PRKCB
CNVR4998.1−HRAS
CNVR7169.1−PRKCA
CNVR7103.1−STAT3
YRI
CEU
CHB
0.00
0.05
0.10
0.15
0.20
0.25

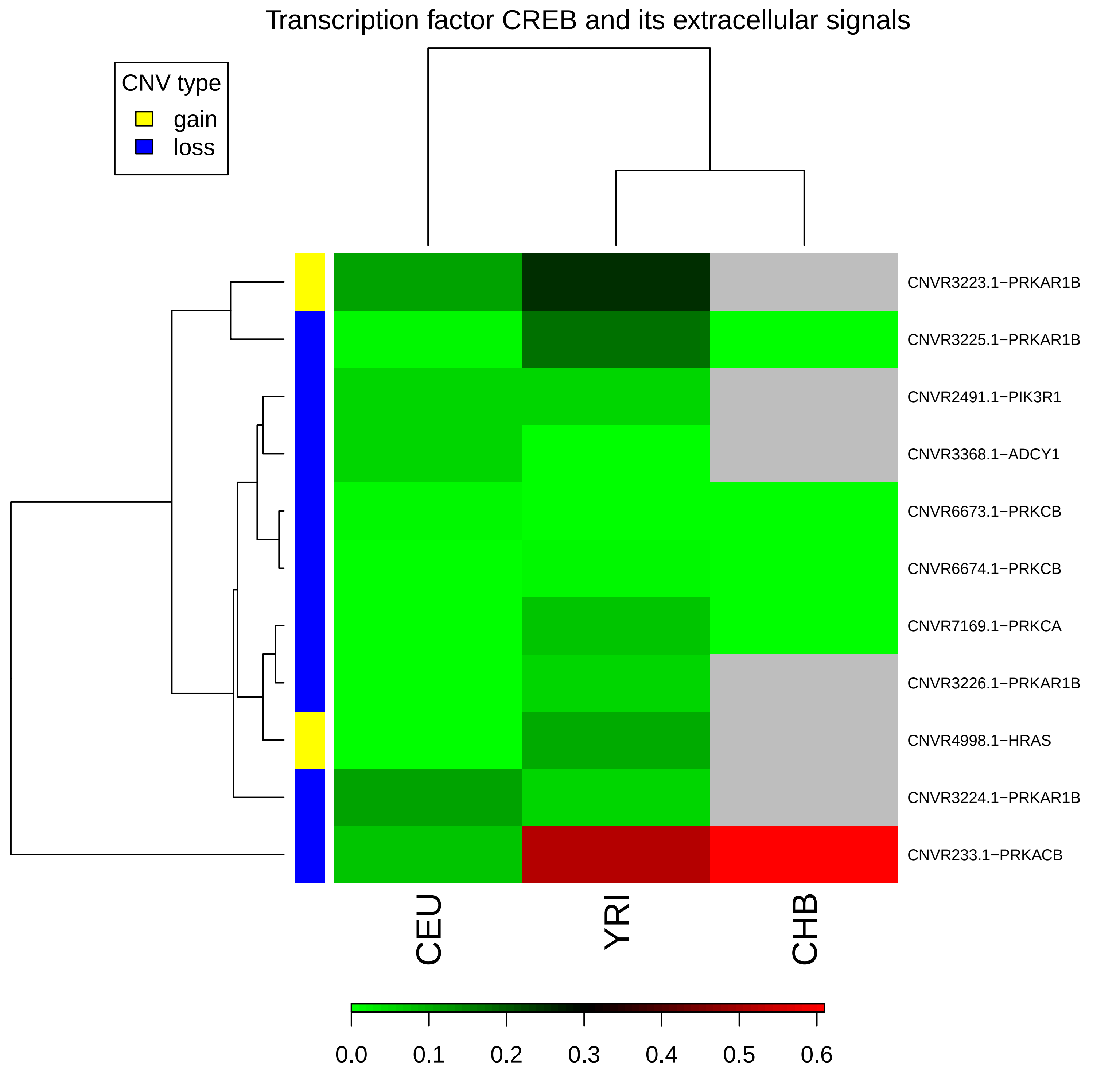
Transcription factor CREB and its extracellular signals
CNV type
gain
loss
CNVR3223.1−PRKAR1B
CNVR3225.1−PRKAR1B
CNVR2491.1−PIK3R1
CNVR3368.1−ADCY1
CNVR6673.1−PRKCB
CNVR6674.1−PRKCB
CNVR7169.1−PRKCA
CNVR3226.1−PRKAR1B
CNVR4998.1−HRAS
CNVR3224.1−PRKAR1B
CNVR233.1−PRKACB
CEU
YRI
CHB
0.0
0.1
0.2
0.3
0.4
0.5
0.6

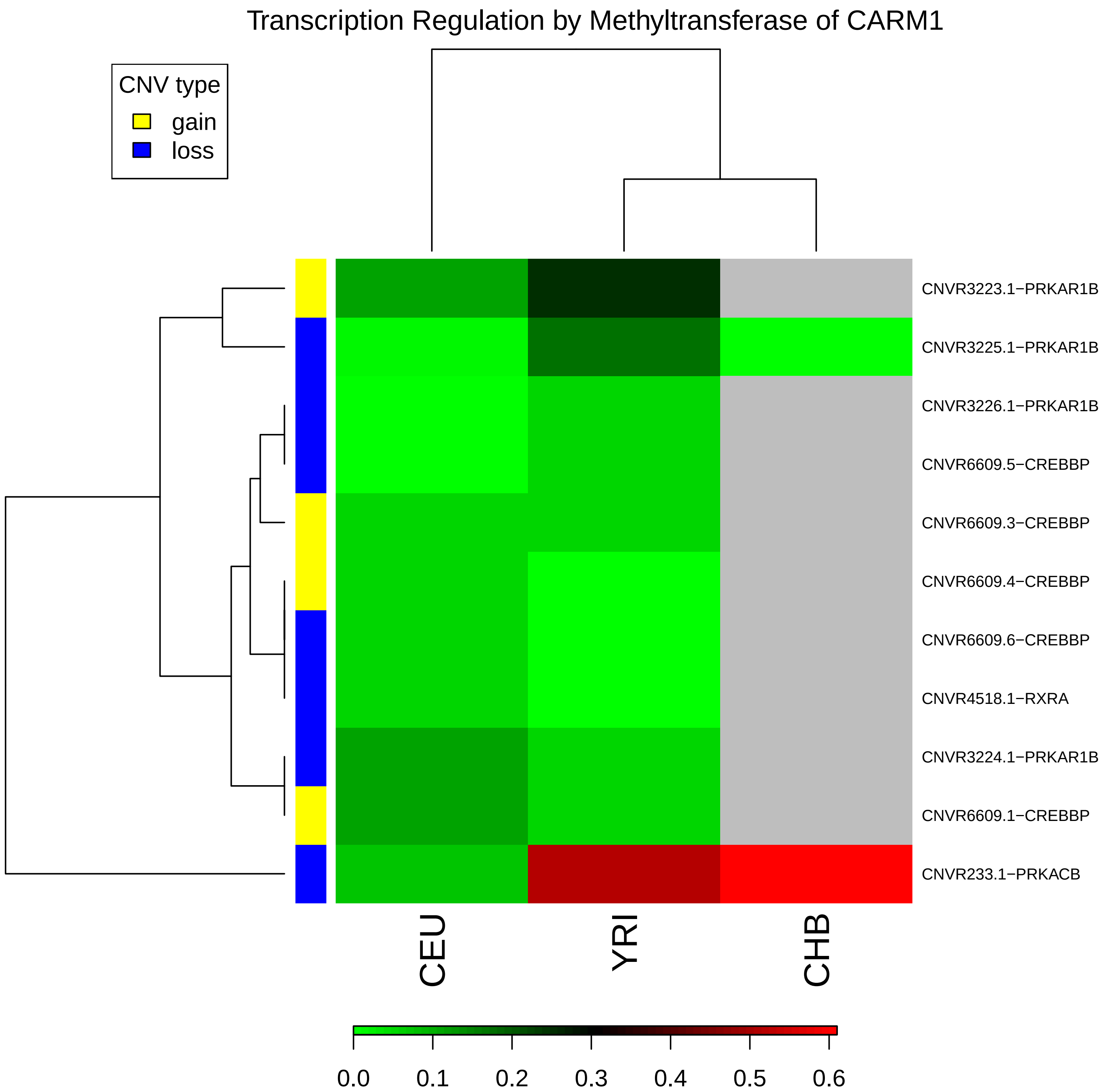

Transcription Regulation by Methyltransferase of CARM1
CNV type
gain
loss
CNVR3223.1−PRKAR1B
CNVR3225.1−PRKAR1B
CNVR3226.1−PRKAR1B
CNVR6609.5−CREBBP
CNVR6609.3−CREBBP
CNVR6609.4−CREBBP
CNVR6609.6−CREBBP
CNVR4518.1−RXRA
CNVR3224.1−PRKAR1B
CNVR6609.1−CREBBP
CNVR233.1−PRKACB
CEU
YRI
CHB
0.0
0.1
0.2
0.3
0.4
0.5
0.6

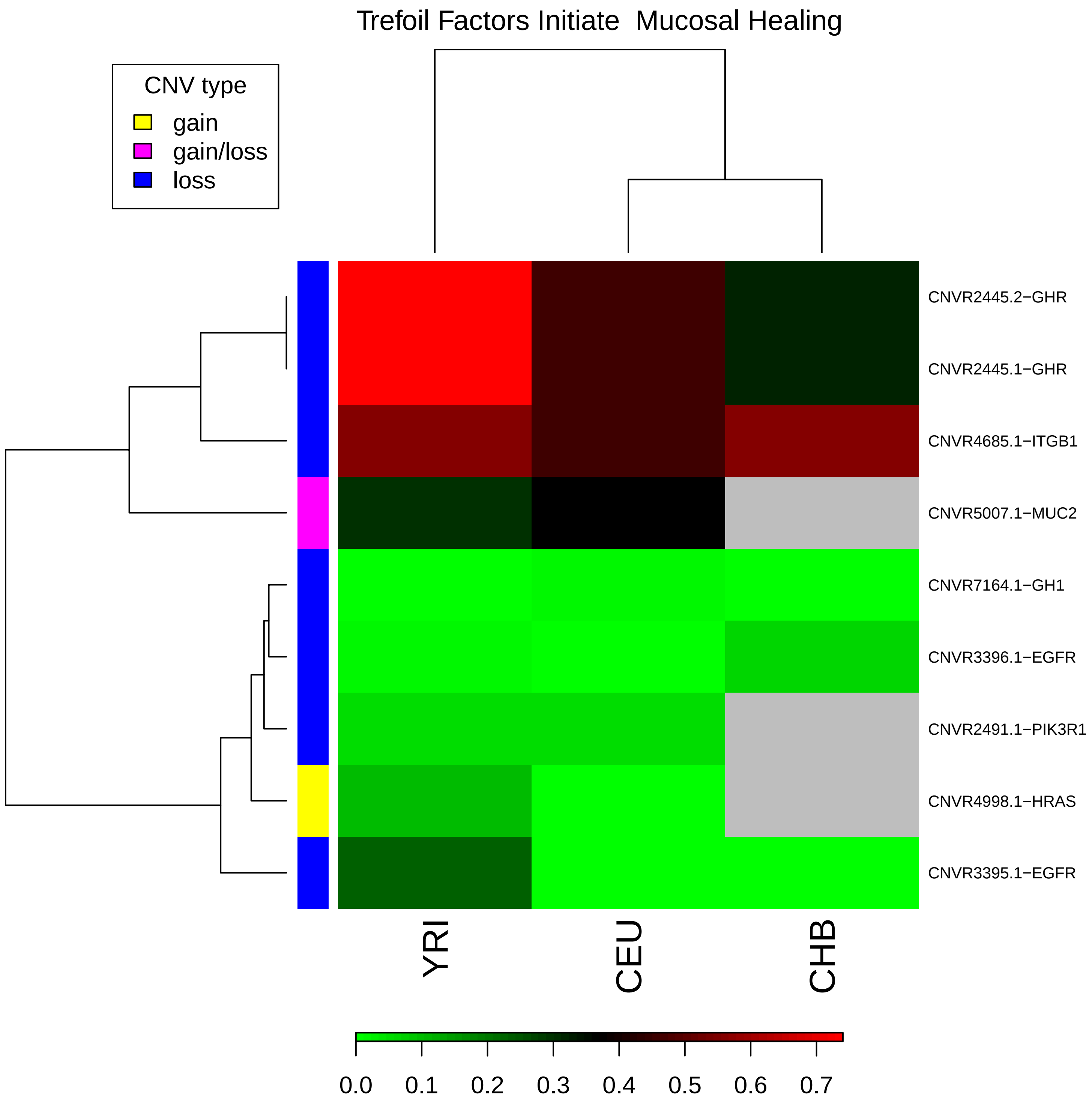
Trefoil Factors Initiate Mucosal Healing
CNV type
gain
gain/loss
loss
CNVR2445.2−GHR
CNVR2445.1−GHR
CNVR4685.1−ITGB1
CNVR5007.1−MUC2
CNVR7164.1−GH1
CNVR3396.1−EGFR
CNVR2491.1−PIK3R1
CNVR4998.1−HRAS
CNVR3395.1−EGFR
YRI
CEU
CHB
0.0
0.1
0.2
0.3
0.4
0.5
0.6
0.7

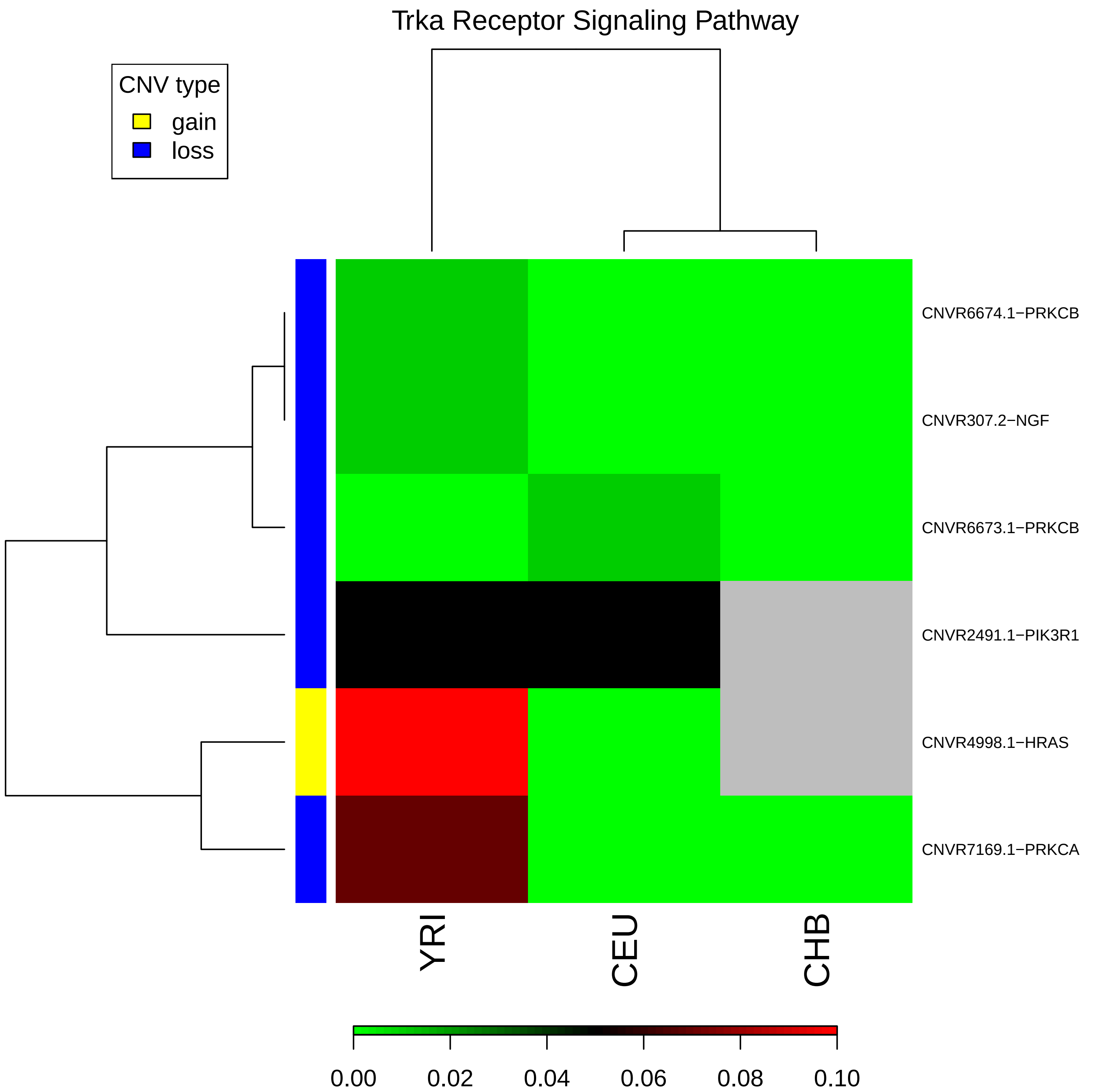
Trka Receptor Signaling Pathway
CNV type
gain
loss
CNVR6674.1−PRKCB
CNVR307.2−NGF
CNVR6673.1−PRKCB
CNVR2491.1−PIK3R1
CNVR4998.1−HRAS
CNVR7169.1−PRKCA
YRI
CEU
CHB
0.00
0.02
0.04
0.06
0.08
0.10

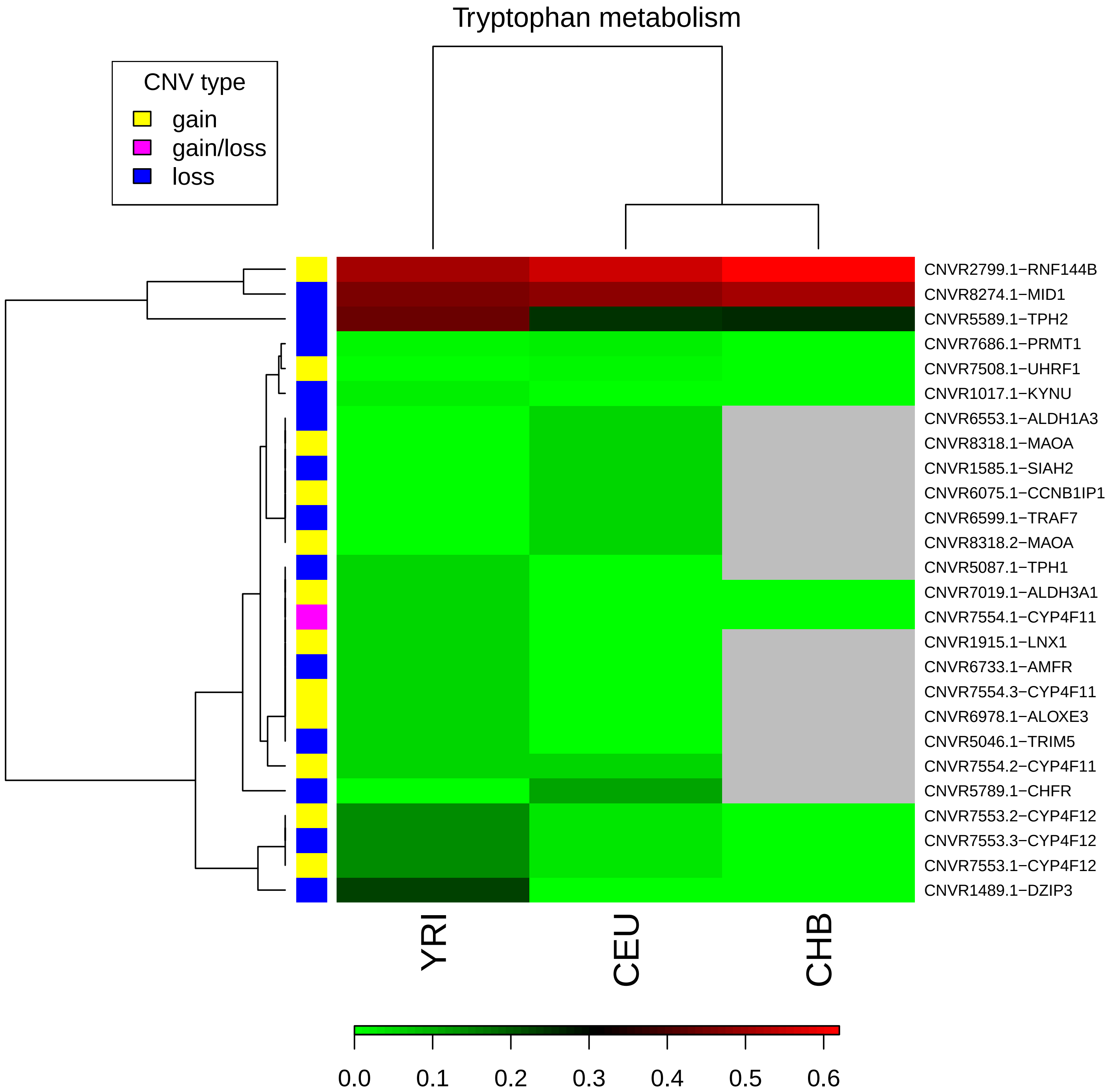
Tryptophan metabolism
CNV type
gain
gain/loss
loss
CNVR2799.1−RNF144B
CNVR8274.1−MID1
CNVR5589.1−TPH2
CNVR7686.1−PRMT1
CNVR7508.1−UHRF1
CNVR1017.1−KYNU
CNVR6553.1−ALDH1A3
CNVR8318.1−MAOA
CNVR1585.1−SIAH2
CNVR6075.1−CCNB1IP1
CNVR6599.1−TRAF7
CNVR8318.2−MAOA
CNVR5087.1−TPH1
CNVR7019.1−ALDH3A1
CNVR7554.1−CYP4F11
CNVR1915.1−LNX1
CNVR6733.1−AMFR
CNVR7554.3−CYP4F11
CNVR6978.1−ALOXE3
CNVR5046.1−TRIM5
CNVR7554.2−CYP4F11
CNVR5789.1−CHFR
CNVR7553.2−CYP4F12
CNVR7553.3−CYP4F12
CNVR7553.1−CYP4F12
CNVR1489.1−DZIP3
YRI
CEU
CHB
0.0
0.1
0.2
0.3
0.4
0.5
0.6

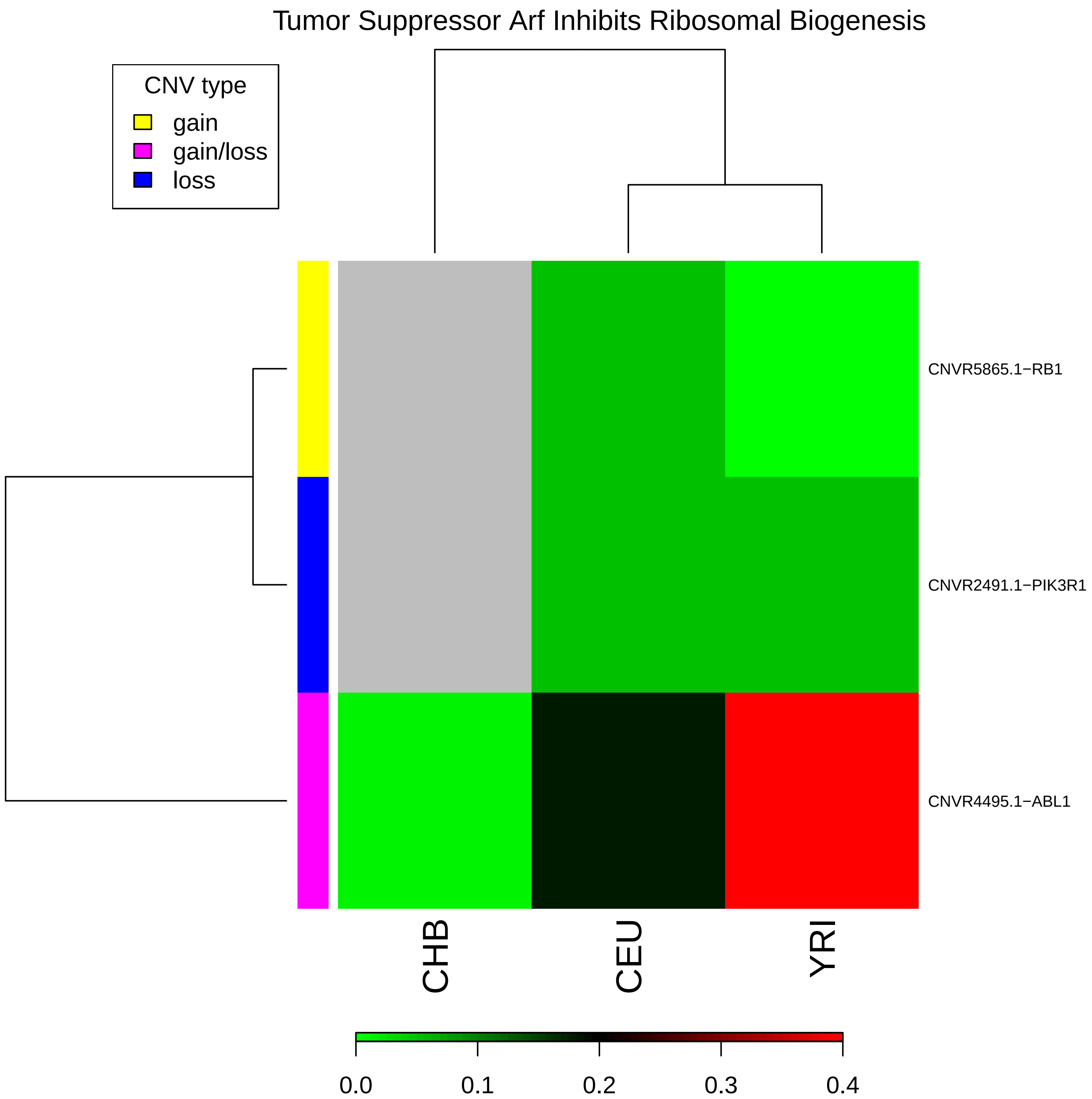
Tumor Suppressor Arf Inhibits Ribosomal Biogenesis
CNV type
gain
gain/loss
loss
CNVR5865.1−RB1
CNVR2491.1−PIK3R1
CNVR4495.1−ABL1
CHB
CEU
YRI
0.0
0.1
0.2
0.3
0.4

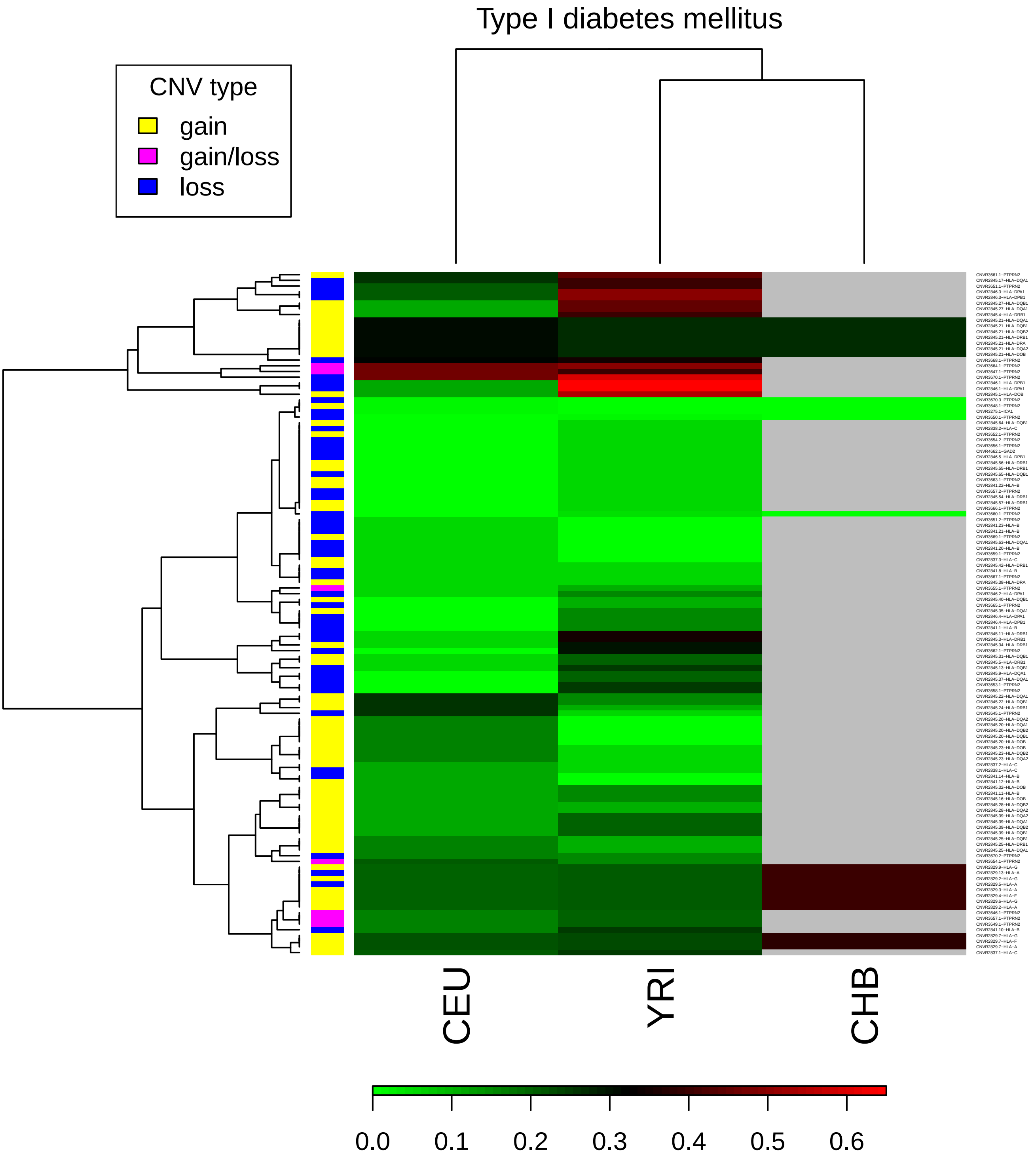

Type I diabetes mellitus
CNV type
gain
gain/loss
loss
CNVR3661.1−PTPRN2
CNVR2845.17−HLA−DQA1
CNVR3651.1−PTPRN2
CNVR2846.3−HLA−DPA1
CNVR2846.3−HLA−DPB1
CNVR2845.27−HLA−DQB1
CNVR2845.27−HLA−DQA1
CNVR2845.4−HLA−DRB1
CNVR2845.21−HLA−DQA1
CNVR2845.21−HLA−DQB1
CNVR2845.21−HLA−DQB2
CNVR2845.21−HLA−DRB1
CNVR2845.21−HLA−DRA
CNVR2845.21−HLA−DQA2
CNVR2845.21−HLA−DOB
CNVR3668.1−PTPRN2
CNVR3664.1−PTPRN2
CNVR3647.1−PTPRN2
CNVR3670.1−PTPRN2
CNVR2846.1−HLA−DPB1
CNVR2846.1−HLA−DPA1
CNVR2845.1−HLA−DOB
CNVR3670.3−PTPRN2
CNVR3648.1−PTPRN2
CNVR3275.1−ICA1
CNVR3650.1−PTPRN2
CNVR2845.64−HLA−DQB1
CNVR2838.2−HLA−C
CNVR3652.1−PTPRN2
CNVR3654.2−PTPRN2
CNVR3656.1−PTPRN2
CNVR4662.1−GAD2
CNVR2846.5−HLA−DPB1
CNVR2845.56−HLA−DRB1
CNVR2845.55−HLA−DRB1
CNVR2845.65−HLA−DQB1
CNVR3663.1−PTPRN2
CNVR2841.22−HLA−B
CNVR3657.2−PTPRN2
CNVR2845.54−HLA−DRB1
CNVR2845.57−HLA−DRB1
CNVR3666.1−PTPRN2
CNVR3660.1−PTPRN2
CNVR3651.2−PTPRN2
CNVR2841.23−HLA−B
CNVR2841.21−HLA−B
CNVR3669.1−PTPRN2
CNVR2845.63−HLA−DQA1
CNVR2841.20−HLA−B
CNVR3659.1−PTPRN2
CNVR2837.3−HLA−C
CNVR2845.42−HLA−DRB1
CNVR2841.8−HLA−B
CNVR3667.1−PTPRN2
CNVR2845.38−HLA−DRA
CNVR3655.1−PTPRN2
CNVR2846.2−HLA−DPA1
CNVR2845.40−HLA−DQB1
CNVR3665.1−PTPRN2
CNVR2845.35−HLA−DQA1
CNVR2846.4−HLA−DPA1
CNVR2846.4−HLA−DPB1
CNVR2841.1−HLA−B
CNVR2845.11−HLA−DRB1
CNVR2845.3−HLA−DRB1
CNVR2845.34−HLA−DRB1
CNVR3662.1−PTPRN2
CNVR2845.31−HLA−DQB1
CNVR2845.5−HLA−DRB1
CNVR2845.13−HLA−DQB1
CNVR2845.9−HLA−DQA1
CNVR2845.37−HLA−DQA1
CNVR3653.1−PTPRN2
CNVR3658.1−PTPRN2
CNVR2845.22−HLA−DQA1
CNVR2845.22−HLA−DQB1
CNVR2845.24−HLA−DRB1
CNVR3645.1−PTPRN2
CNVR2845.20−HLA−DQA2
CNVR2845.20−HLA−DQA1
CNVR2845.20−HLA−DQB2
CNVR2845.20−HLA−DQB1
CNVR2845.20−HLA−DOB
CNVR2845.23−HLA−DOB
CNVR2845.23−HLA−DQB2
CNVR2845.23−HLA−DQA2
CNVR2837.2−HLA−C
CNVR2838.1−HLA−C
CNVR2841.14−HLA−B
CNVR2841.12−HLA−B
CNVR2845.32−HLA−DOB
CNVR2841.11−HLA−B
CNVR2845.16−HLA−DOB
CNVR2845.28−HLA−DQB2
CNVR2845.28−HLA−DQA2
CNVR2845.39−HLA−DQA2
CNVR2845.39−HLA−DQA1
CNVR2845.39−HLA−DQB2
CNVR2845.39−HLA−DQB1
CNVR2845.25−HLA−DQB1
CNVR2845.25−HLA−DRB1
CNVR2845.25−HLA−DQA1
CNVR3670.2−PTPRN2
CNVR3654.1−PTPRN2
CNVR2829.9−HLA−G
CNVR2829.13−HLA−A
CNVR2829.2−HLA−G
CNVR2829.5−HLA−A
CNVR2829.3−HLA−A
CNVR2829.4−HLA−F
CNVR2829.6−HLA−G
CNVR2829.2−HLA−A
CNVR3646.1−PTPRN2
CNVR3657.1−PTPRN2
CNVR3649.1−PTPRN2
CNVR2841.10−HLA−B
CNVR2829.7−HLA−G
CNVR2829.7−HLA−F
CNVR2829.7−HLA−A
CNVR2837.1−HLA−C
CEU
YRI
CHB
0.0
0.1
0.2
0.3
0.4
0.5
0.6

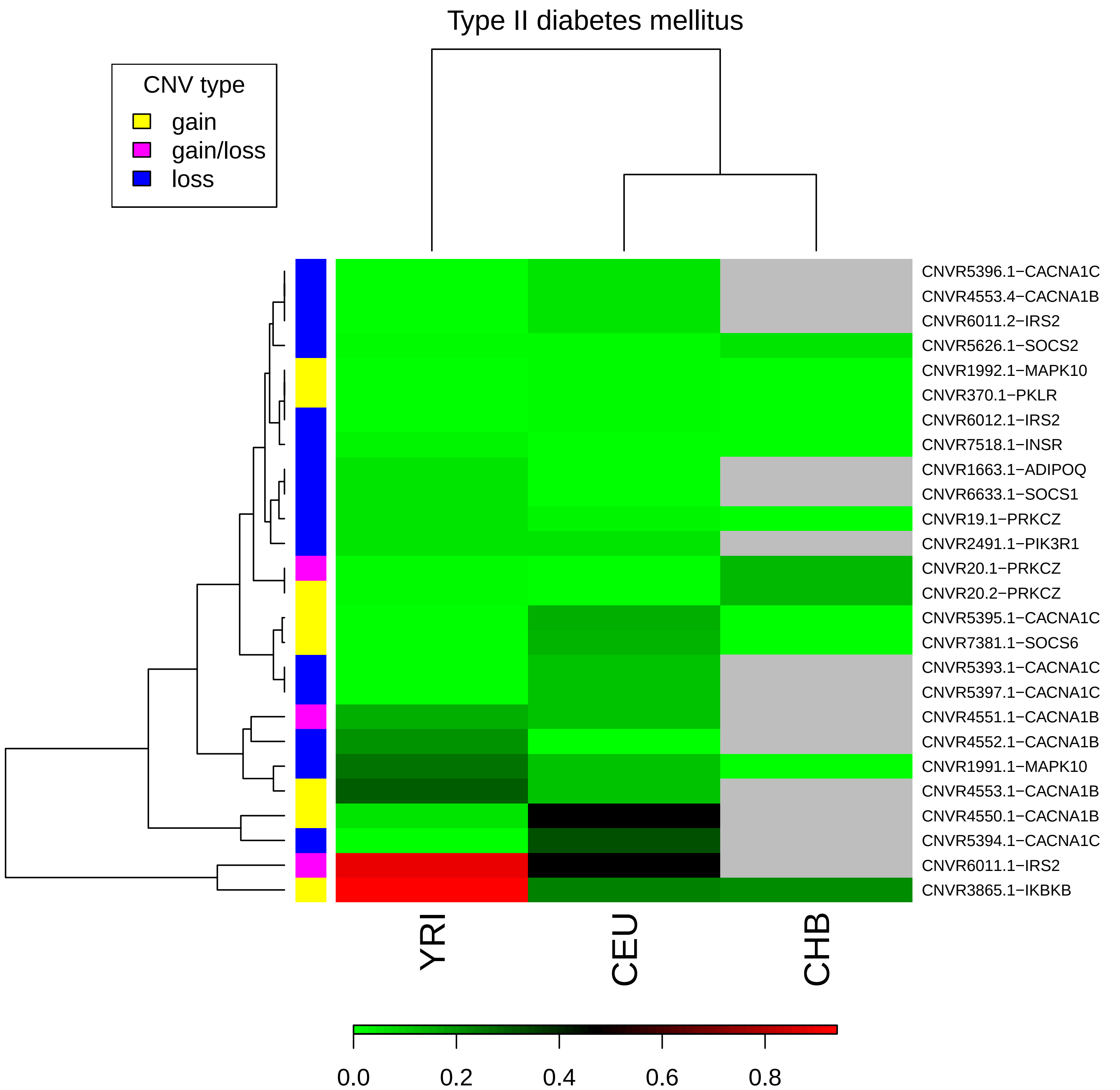

Type II diabetes mellitus
CNV type
gain
gain/loss
loss
CNVR5396.1−CACNA1C
CNVR4553.4−CACNA1B
CNVR6011.2−IRS2
CNVR5626.1−SOCS2
CNVR1992.1−MAPK10
CNVR370.1−PKLR
CNVR6012.1−IRS2
CNVR7518.1−INSR
CNVR1663.1−ADIPOQ
CNVR6633.1−SOCS1
CNVR19.1−PRKCZ
CNVR2491.1−PIK3R1
CNVR20.1−PRKCZ
CNVR20.2−PRKCZ
CNVR5395.1−CACNA1C
CNVR7381.1−SOCS6
CNVR5393.1−CACNA1C
CNVR5397.1−CACNA1C
CNVR4551.1−CACNA1B
CNVR4552.1−CACNA1B
CNVR1991.1−MAPK10
CNVR4553.1−CACNA1B
CNVR4550.1−CACNA1B
CNVR5394.1−CACNA1C
CNVR6011.1−IRS2
CNVR3865.1−IKBKB
YRI
CEU
CHB
0.0
0.2
0.4
0.6
0.8

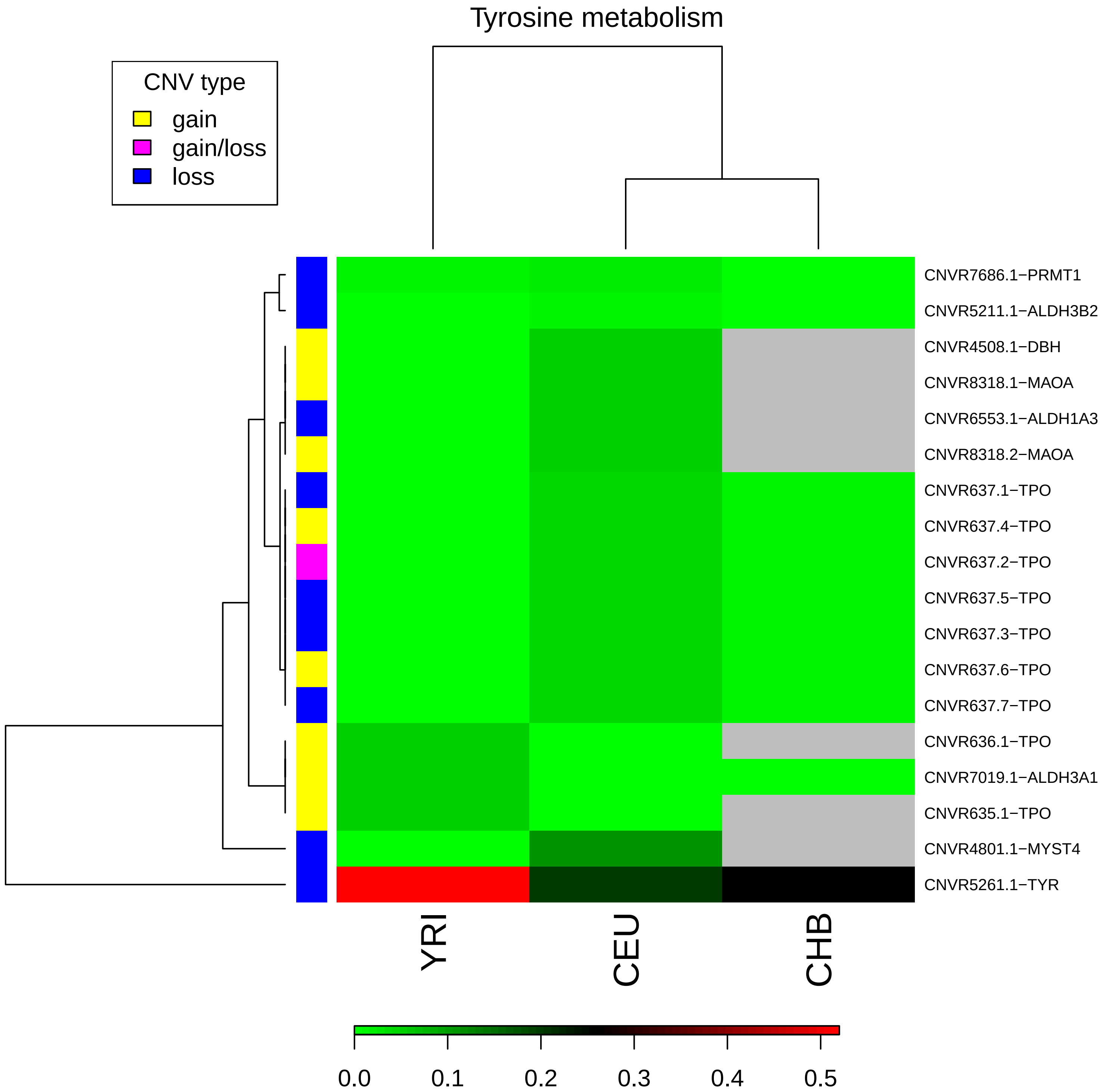

Tyrosine metabolism
CNV type
gain
gain/loss
loss
CNVR7686.1−PRMT1
CNVR5211.1−ALDH3B2
CNVR4508.1−DBH
CNVR8318.1−MAOA
CNVR6553.1−ALDH1A3
CNVR8318.2−MAOA
CNVR637.1−TPO
CNVR637.4−TPO
CNVR637.2−TPO
CNVR637.5−TPO
CNVR637.3−TPO
CNVR637.6−TPO
CNVR637.7−TPO
CNVR636.1−TPO
CNVR7019.1−ALDH3A1
CNVR635.1−TPO
CNVR4801.1−MYST4
CNVR5261.1−TYR
YRI
CEU
CHB
0.0
0.1
0.2
0.3
0.4
0.5

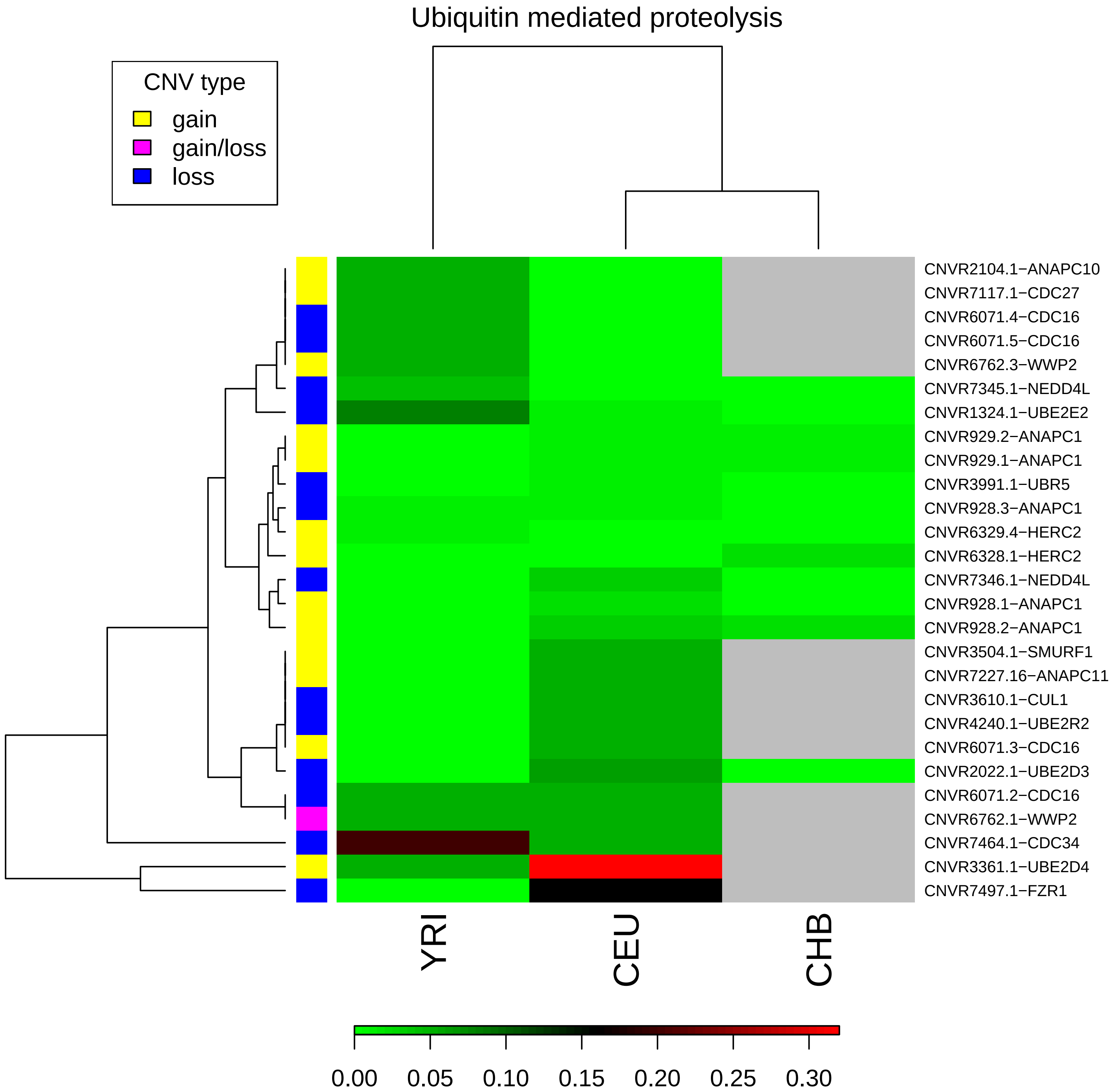

Ubiquitin mediated proteolysis
CNV type
gain
gain/loss
loss
CNVR2104.1−ANAPC10
CNVR7117.1−CDC27
CNVR6071.4−CDC16
CNVR6071.5−CDC16
CNVR6762.3−WWP2
CNVR7345.1−NEDD4L
CNVR1324.1−UBE2E2
CNVR929.2−ANAPC1
CNVR929.1−ANAPC1
CNVR3991.1−UBR5
CNVR928.3−ANAPC1
CNVR6329.4−HERC2
CNVR6328.1−HERC2
CNVR7346.1−NEDD4L
CNVR928.1−ANAPC1
CNVR928.2−ANAPC1
CNVR3504.1−SMURF1
CNVR7227.16−ANAPC11
CNVR3610.1−CUL1
CNVR4240.1−UBE2R2
CNVR6071.3−CDC16
CNVR2022.1−UBE2D3
CNVR6071.2−CDC16
CNVR6762.1−WWP2
CNVR7464.1−CDC34
CNVR3361.1−UBE2D4
CNVR7497.1−FZR1
YRI
CEU
CHB
0.00
0.05
0.10
0.15
0.20
0.25
0.30

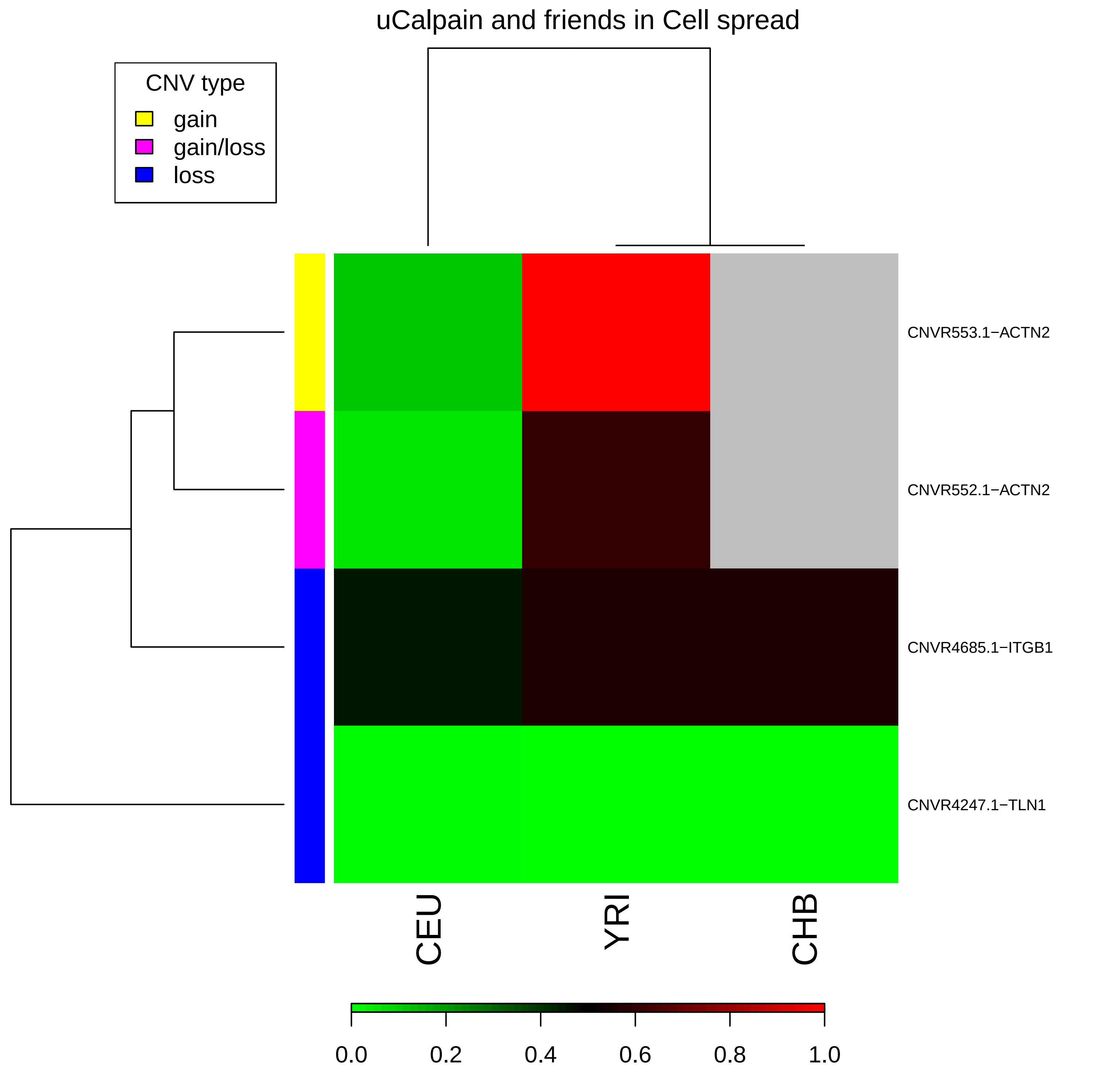
uCalpain and friends in Cell spread
CNV type
gain
gain/loss
loss
CNVR553.1−ACTN2
CNVR552.1−ACTN2
CNVR4685.1−ITGB1
CNVR4247.1−TLN1
CEU
YRI
CHB
0.0
0.2
0.4
0.6
0.8
1.0

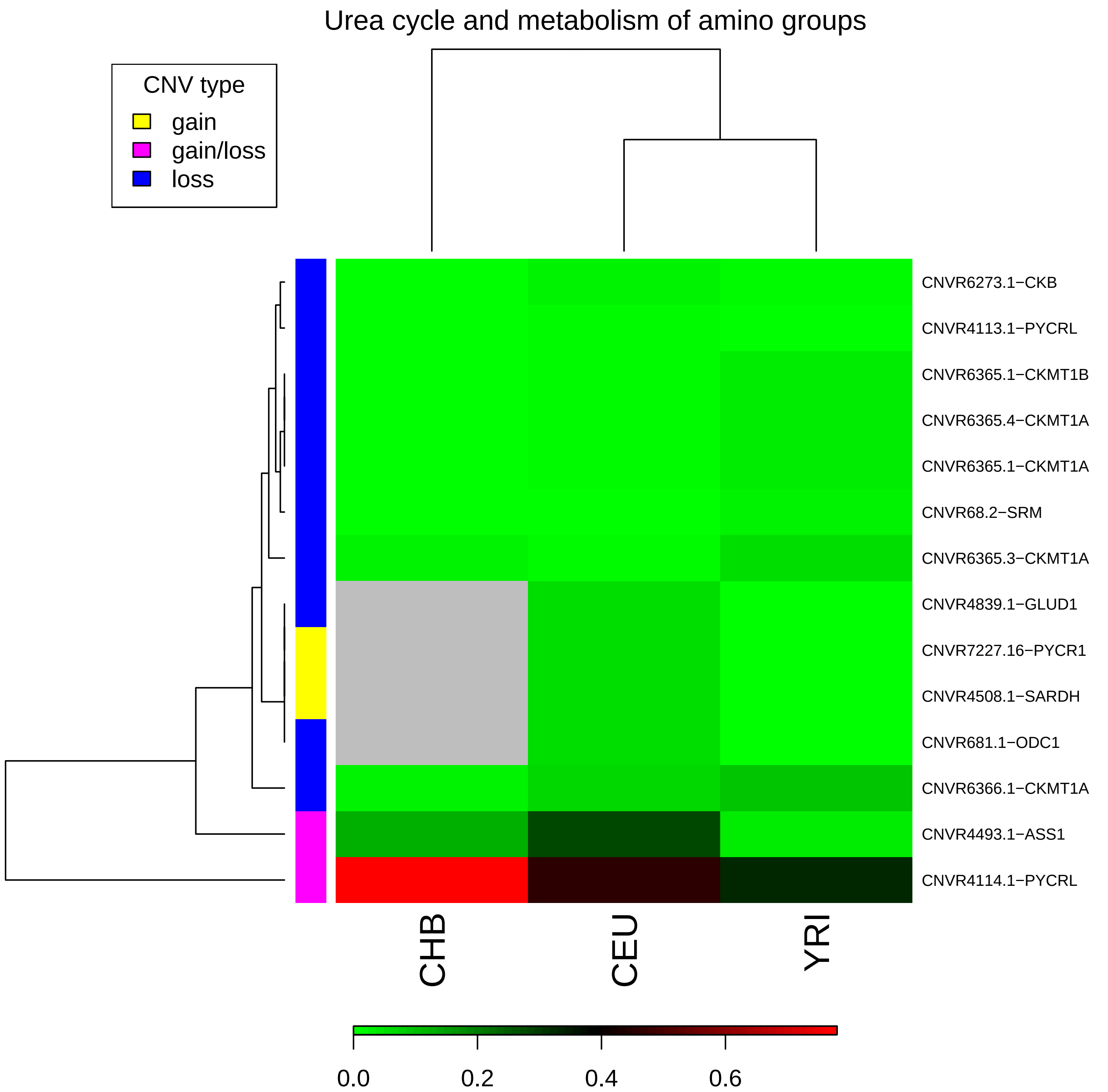

Urea cycle and metabolism of amino groups
CNV type
gain
gain/loss
loss
CNVR6273.1−CKB
CNVR4113.1−PYCRL
CNVR6365.1−CKMT1B
CNVR6365.4−CKMT1A
CNVR6365.1−CKMT1A
CNVR68.2−SRM
CNVR6365.3−CKMT1A
CNVR4839.1−GLUD1
CNVR7227.16−PYCR1
CNVR4508.1−SARDH
CNVR681.1−ODC1
CNVR6366.1−CKMT1A
CNVR4493.1−ASS1
CNVR4114.1−PYCRL
CHB
CEU
YRI
0.0
0.2
0.4
0.6

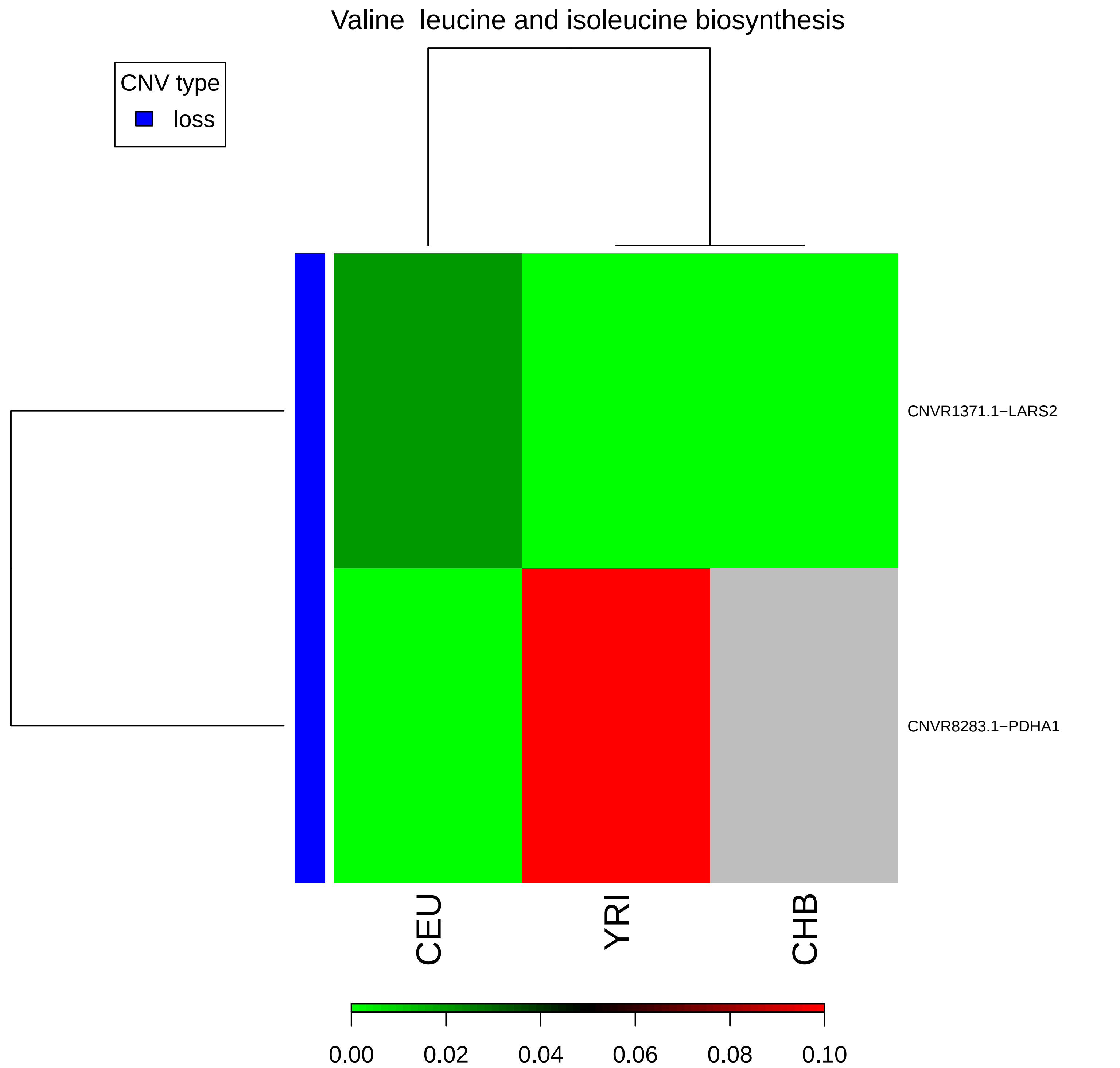
Valine  leucine and isoleucine biosynthesis
CNV type
loss
CNVR1371.1−LARS2
CNVR8283.1−PDHA1
CEU
YRI
CHB
0.00
0.02
0.04
0.06
0.08
0.10

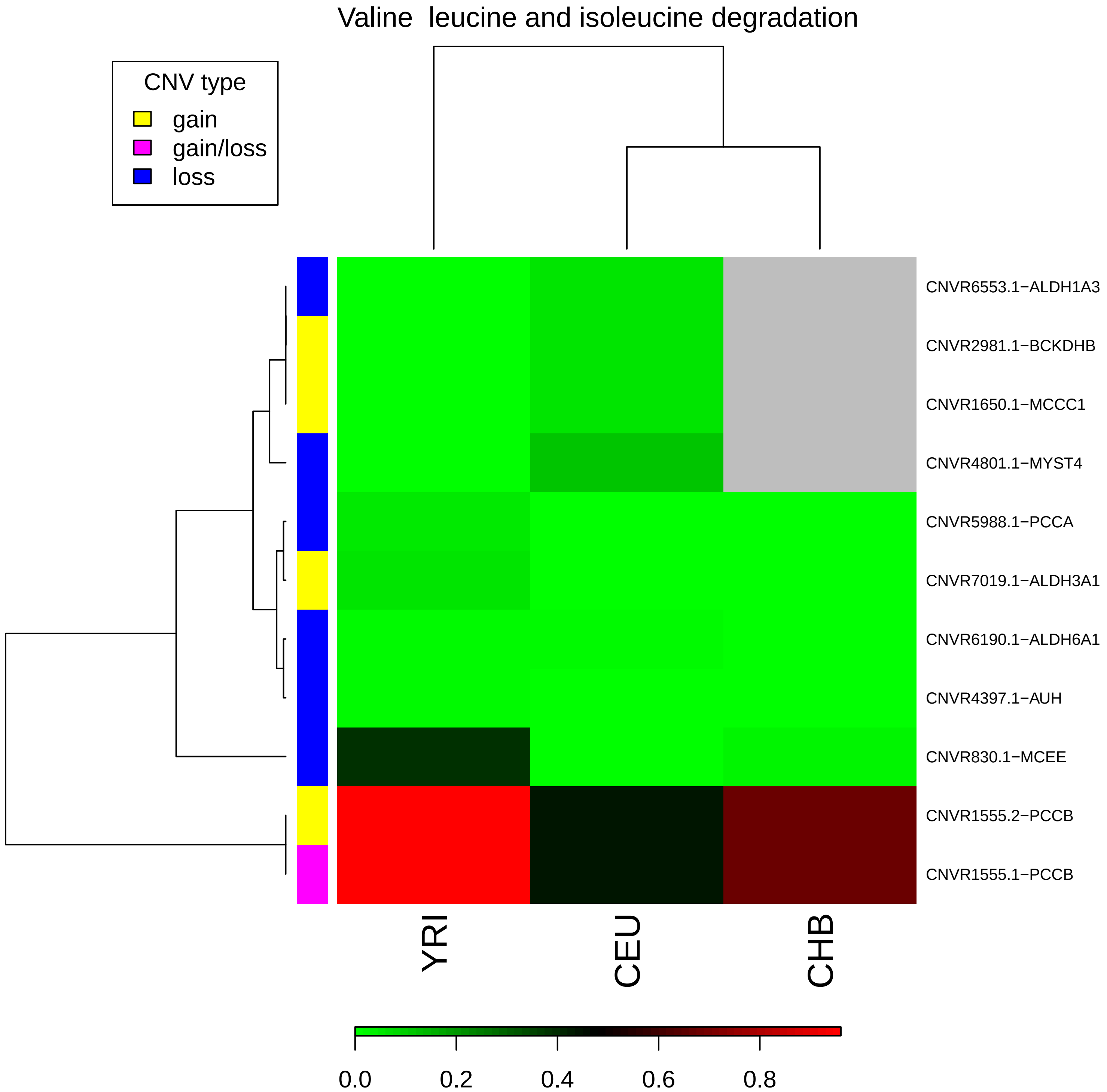
Valine leucine and isoleucine degradation
CNV type
gain
gain/loss
loss
CNVR6553.1−ALDH1A3
CNVR2981.1−BCKDHB
CNVR1650.1−MCCC1
CNVR4801.1−MYST4
CNVR5988.1−PCCA
CNVR7019.1−ALDH3A1
CNVR6190.1−ALDH6A1
CNVR4397.1−AUH
CNVR830.1−MCEE
CNVR1555.2−PCCB
CNVR1555.1−PCCB
YRI
CEU
CHB
0.0
0.2
0.4
0.6
0.8

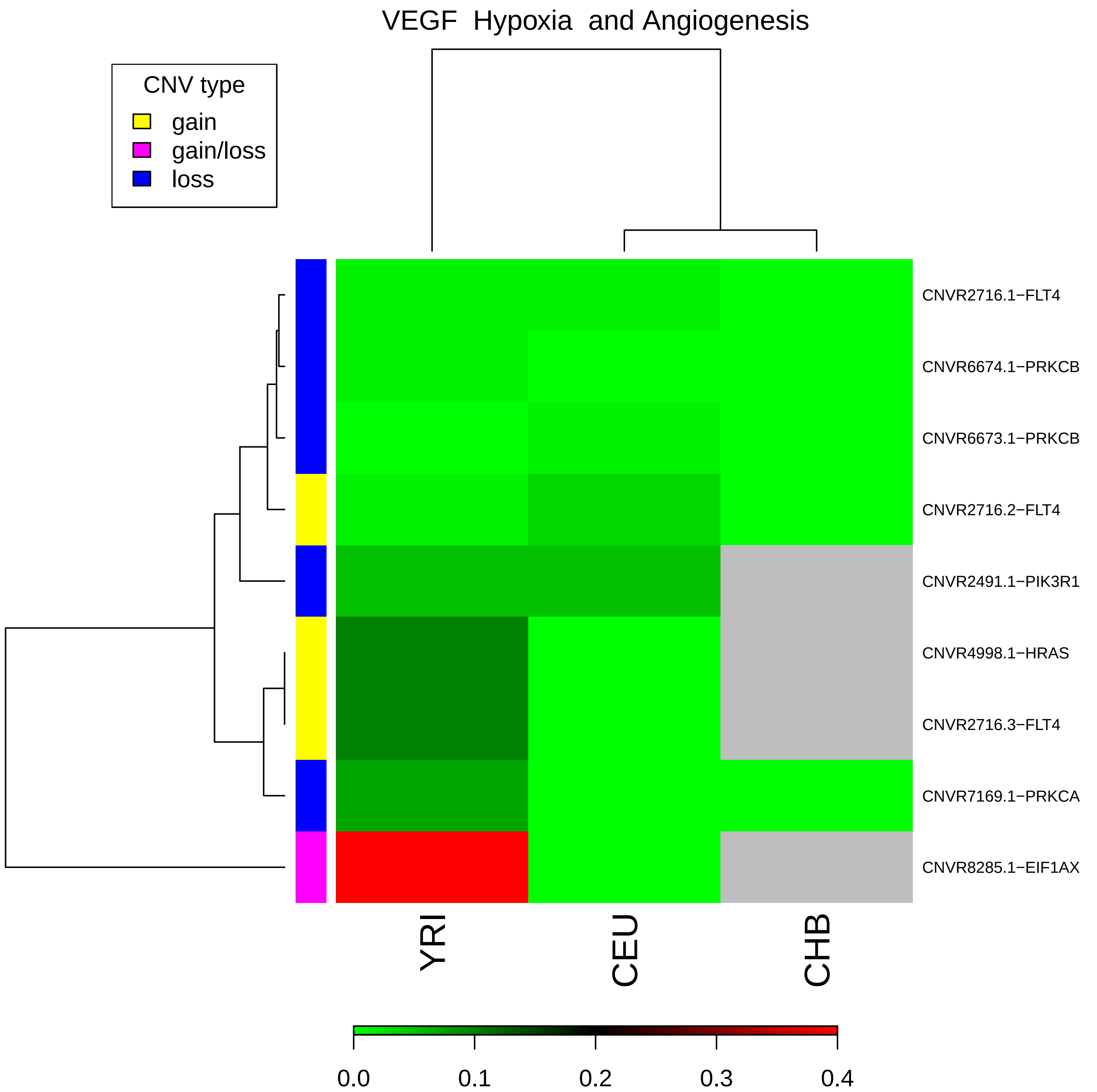

VEGF Hypoxia and Angiogenesis
CNV type
gain
gain/loss
loss
CNVR2716.1−FLT4
CNVR6674.1−PRKCB
CNVR6673.1−PRKCB
CNVR2716.2−FLT4
CNVR2491.1−PIK3R1
CNVR4998.1−HRAS
CNVR2716.3−FLT4
CNVR7169.1−PRKCA
CNVR8285.1−EIF1AX
YRI
CEU
CHB
0.0
0.1
0.2
0.3
0.4

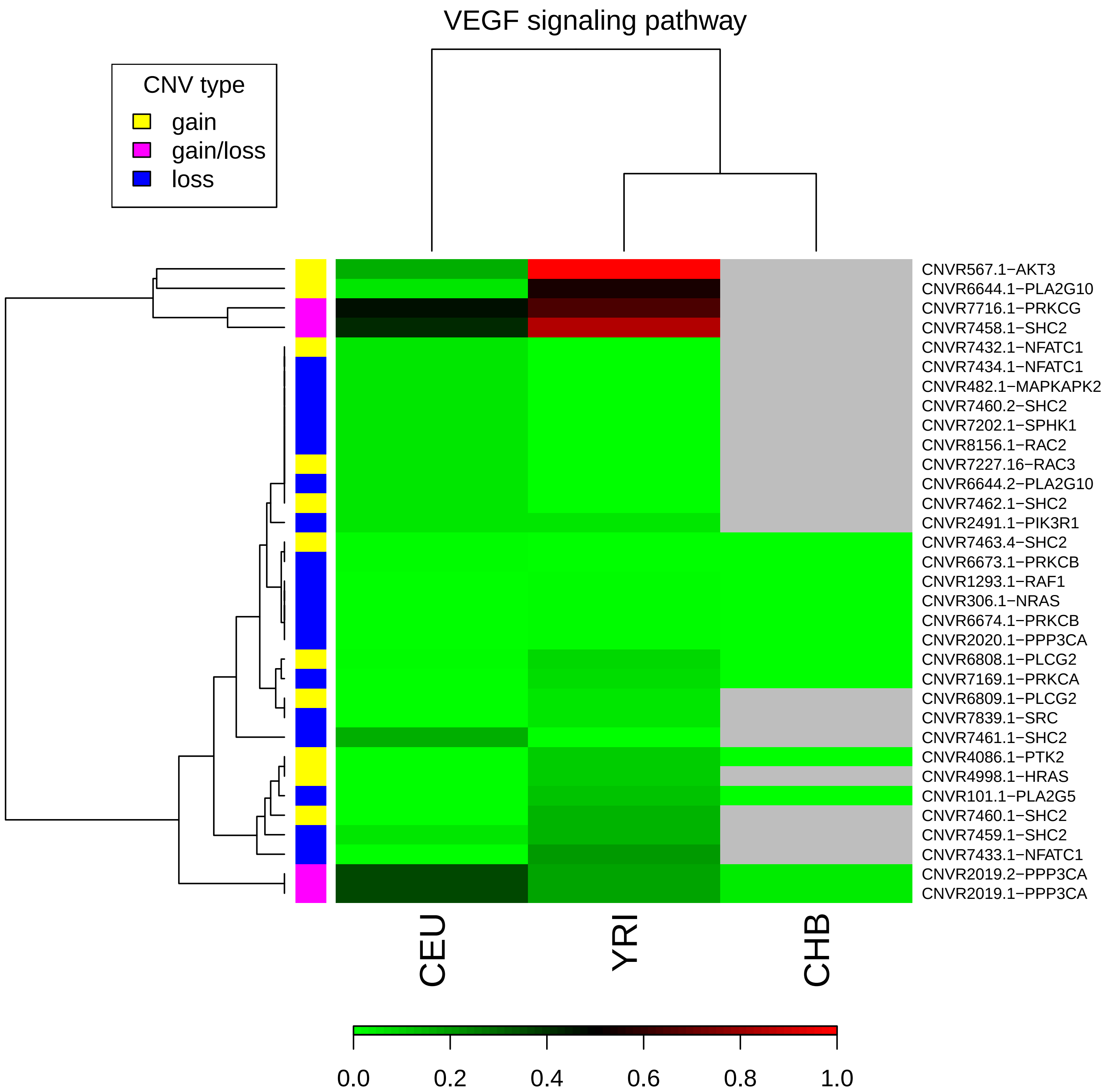

VEGF signaling pathway
CNV type
gain
gain/loss
loss
CNVR567.1−AKT3
CNVR6644.1−PLA2G10
CNVR7716.1−PRKCG
CNVR7458.1−SHC2
CNVR7432.1−NFATC1
CNVR7434.1−NFATC1
CNVR482.1−MAPKAPK2
CNVR7460.2−SHC2
CNVR7202.1−SPHK1
CNVR8156.1−RAC2
CNVR7227.16−RAC3
CNVR6644.2−PLA2G10
CNVR7462.1−SHC2
CNVR2491.1−PIK3R1
CNVR7463.4−SHC2
CNVR6673.1−PRKCB
CNVR1293.1−RAF1
CNVR306.1−NRAS
CNVR6674.1−PRKCB
CNVR2020.1−PPP3CA
CNVR6808.1−PLCG2
CNVR7169.1−PRKCA
CNVR6809.1−PLCG2
CNVR7839.1−SRC
CNVR7461.1−SHC2
CNVR4086.1−PTK2
CNVR4998.1−HRAS
CNVR101.1−PLA2G5
CNVR7460.1−SHC2
CNVR7459.1−SHC2
CNVR7433.1−NFATC1
CNVR2019.2−PPP3CA
CNVR2019.1−PPP3CA
CEU
YRI
CHB
0.0
0.2
0.4
0.6
0.8
1.0

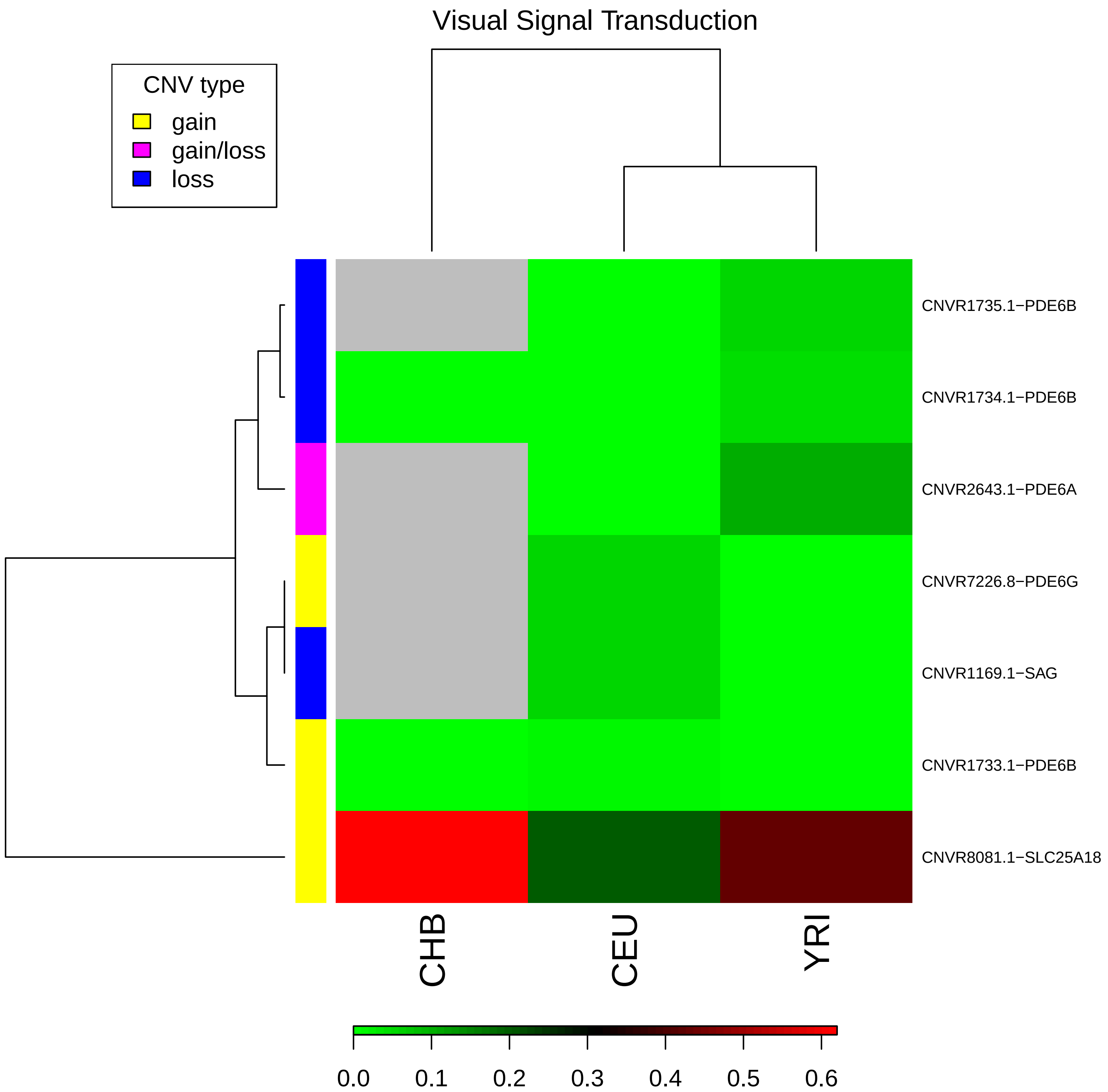

Visual Signal Transduction
CNV type
gain
gain/loss
loss
CNVR1735.1−PDE6B
CNVR1734.1−PDE6B
CNVR2643.1−PDE6A
CNVR7226.8−PDE6G
CNVR1169.1−SAG
CNVR1733.1−PDE6B
CNVR8081.1−SLC25A18
CHB
CEU
YRI
0.0
0.1
0.2
0.3
0.4
0.5
0.6

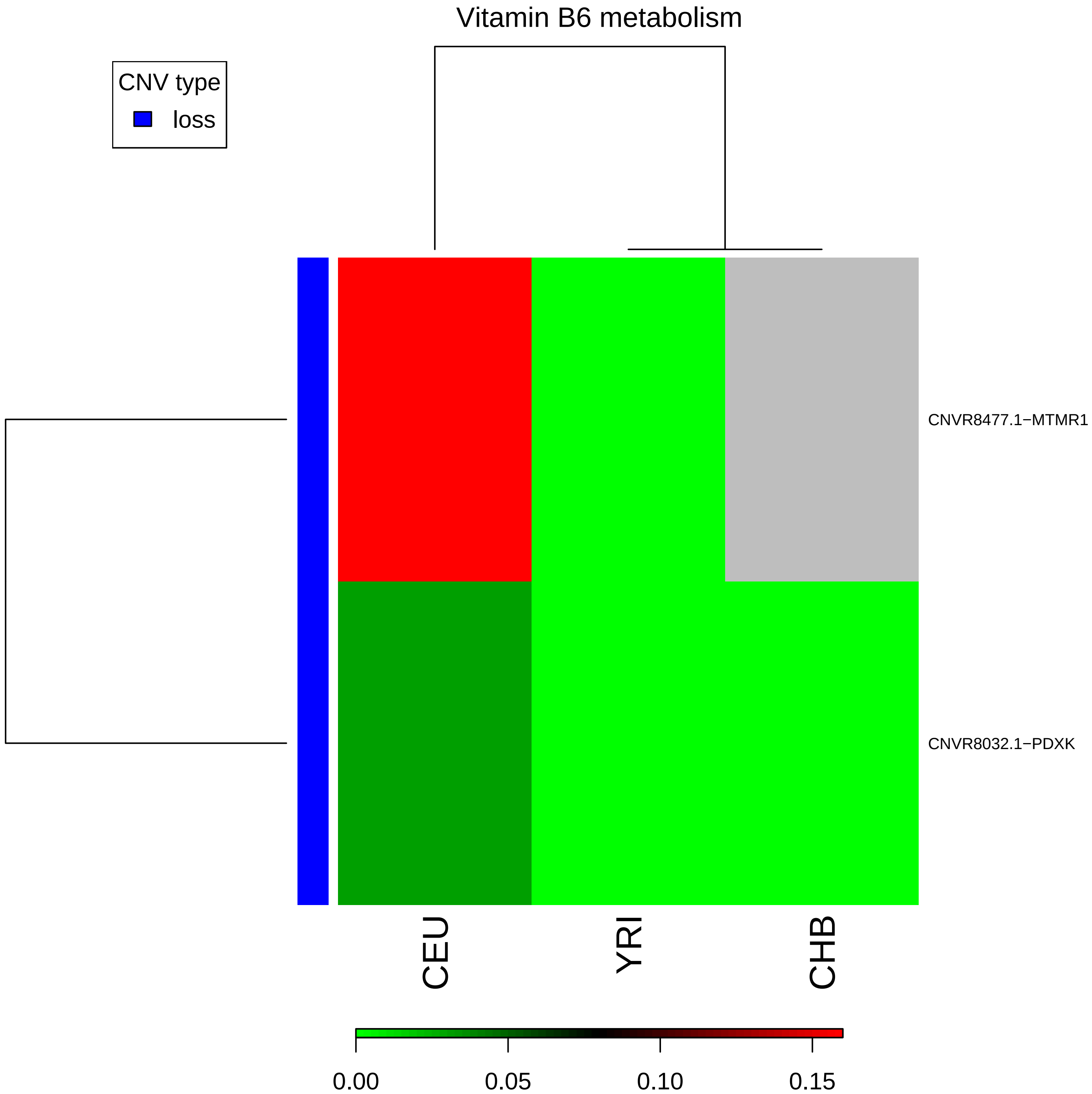
Vitamin B6 metabolism
CNV type
loss
CNVR8477.1−MTMR1
CNVR8032.1−PDXK
CEU
YRI
CHB
0.00
0.05
0.10
0.15

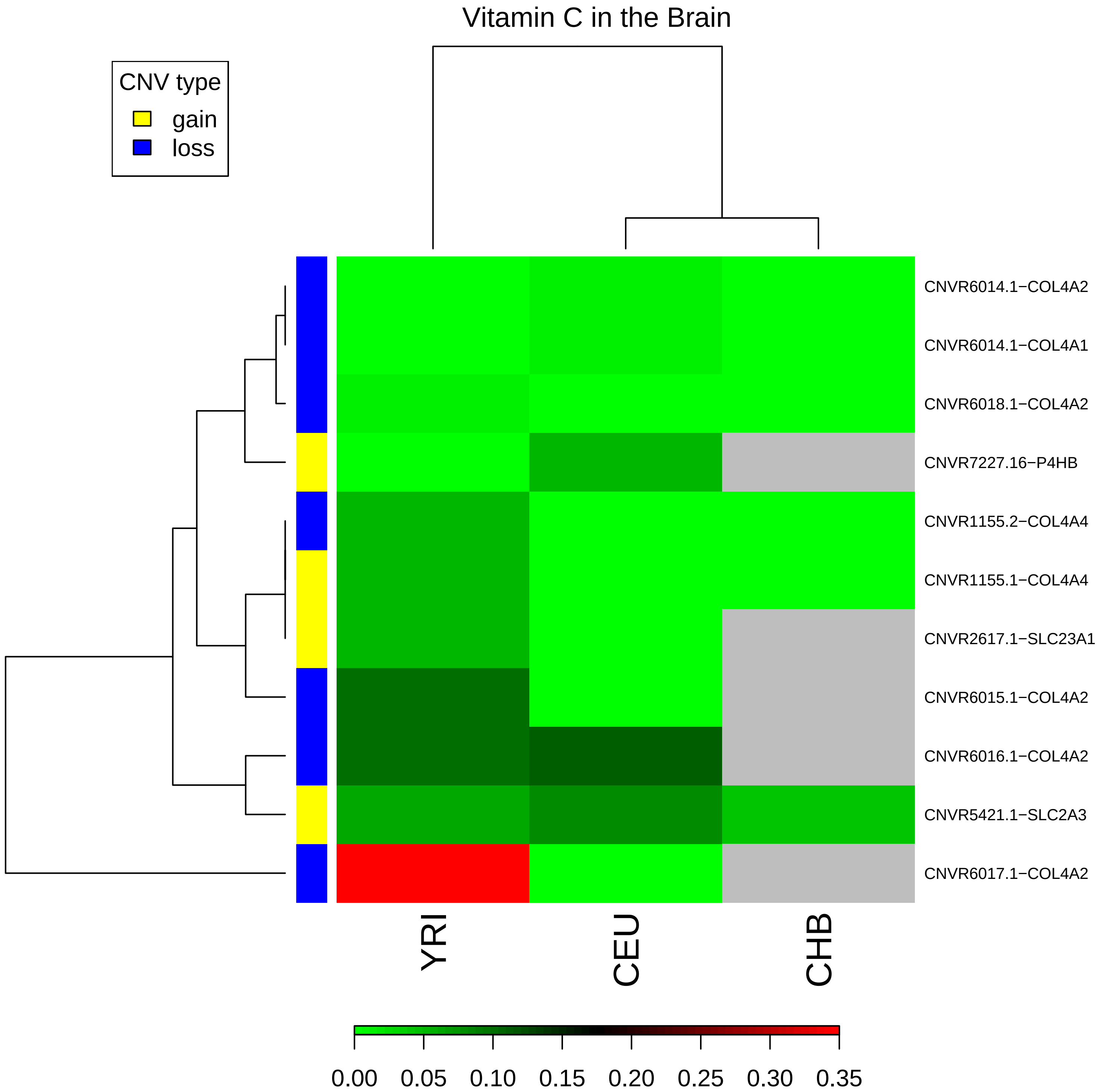

Vitamin C in the Brain
CNV type
gain
loss
CNVR6014.1−COL4A2
CNVR6014.1−COL4A1
CNVR6018.1−COL4A2
CNVR7227.16−P4HB
CNVR1155.2−COL4A4
CNVR1155.1−COL4A4
CNVR2617.1−SLC23A1
CNVR6015.1−COL4A2
CNVR6016.1−COL4A2
CNVR5421.1−SLC2A3
CNVR6017.1−COL4A2
YRI
CEU
CHB
0.00
0.05
0.10
0.15
0.20
0.25
0.30
0.35

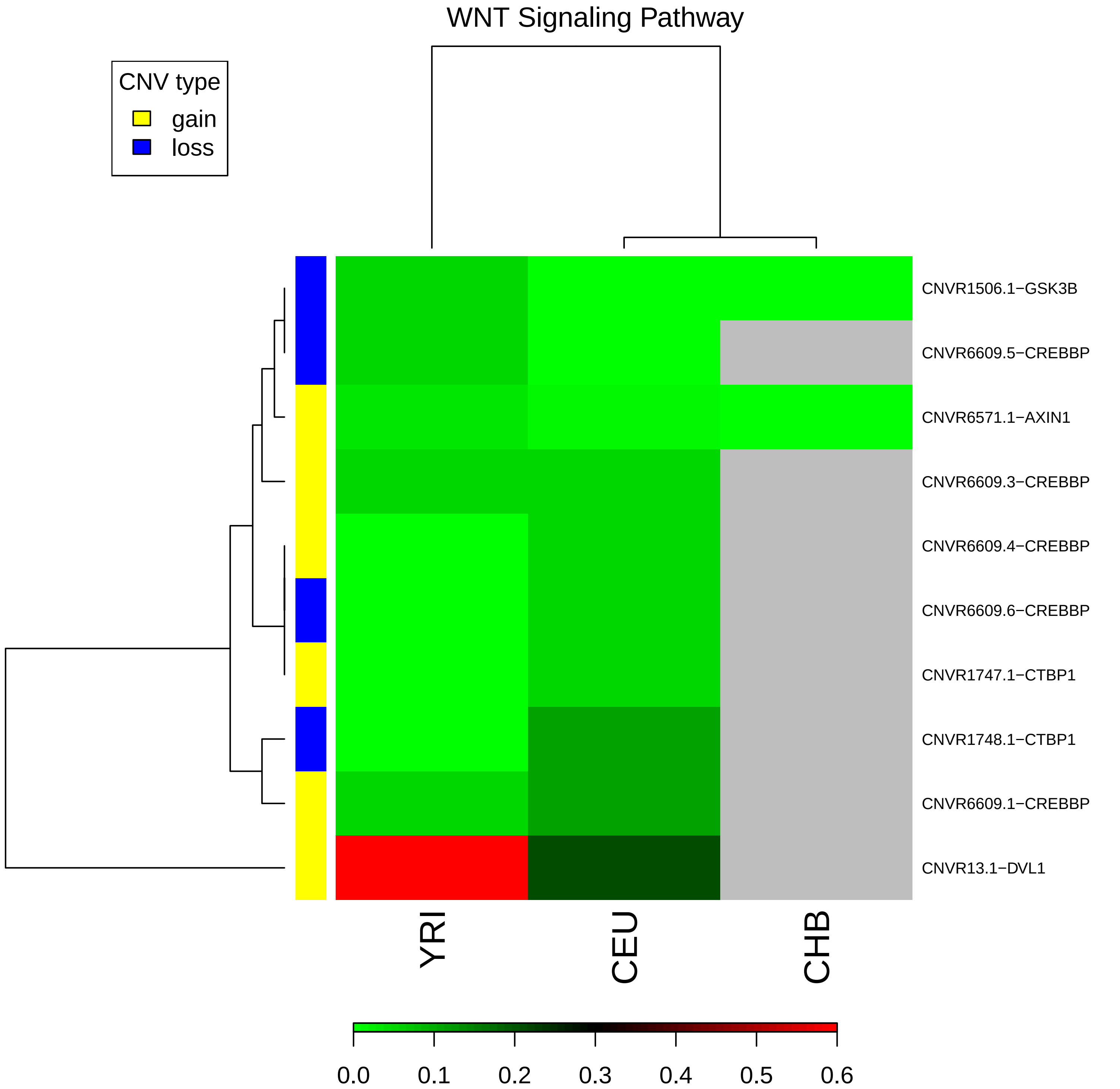
WNT Signaling Pathway
CNV type
gain
loss
CNVR1506.1−GSK3B
CNVR6609.5−CREBBP
CNVR6571.1−AXIN1
CNVR6609.3−CREBBP
CNVR6609.4−CREBBP
CNVR6609.6−CREBBP
CNVR1747.1−CTBP1
CNVR1748.1−CTBP1
CNVR6609.1−CREBBP
CNVR13.1−DVL1
YRI
CEU
CHB
0.0
0.1
0.2
0.3
0.4
0.5
0.6

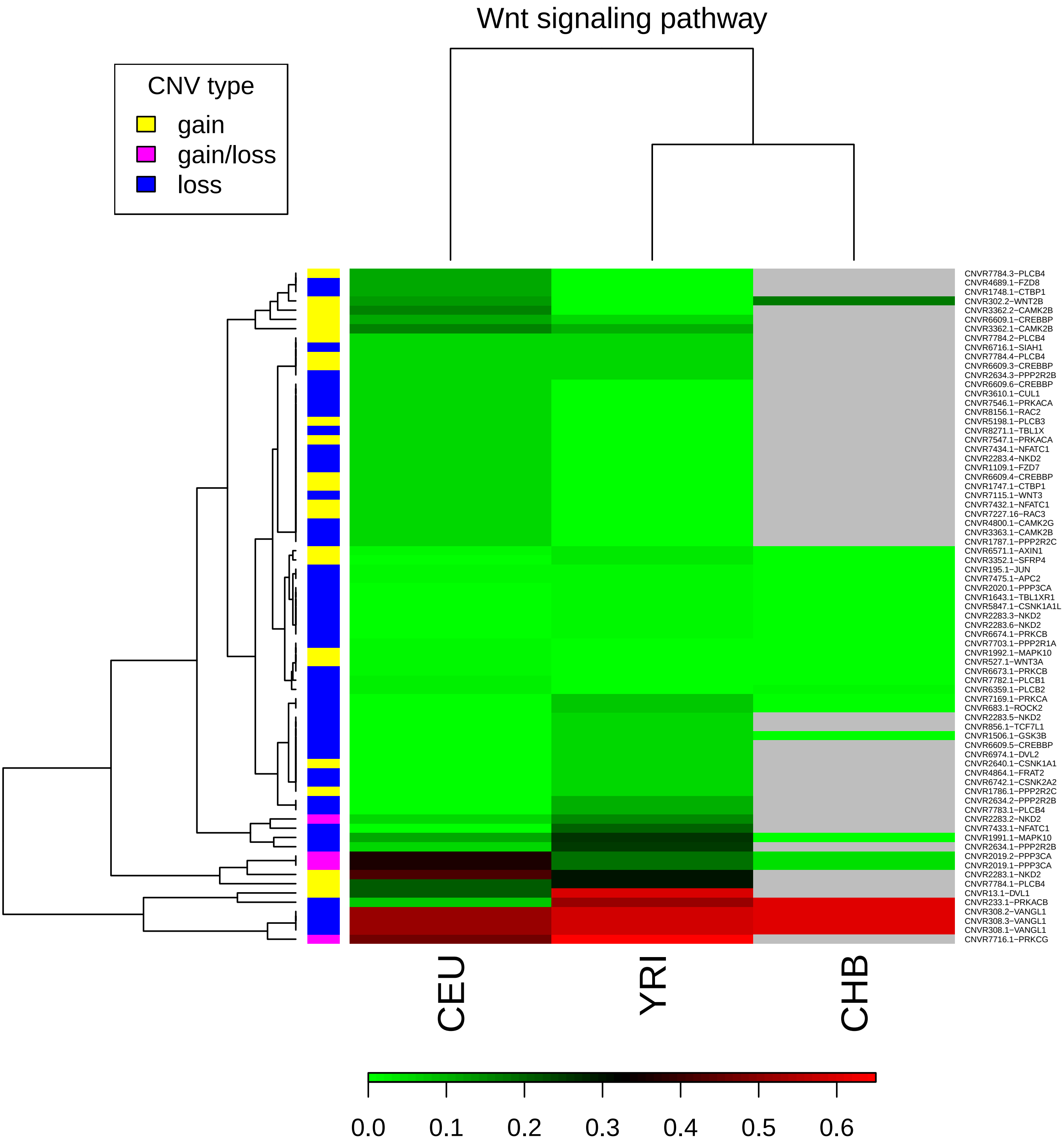
Wnt signaling pathway
CNV type
gain
gain/loss
loss
CNVR7784.3−PLCB4
CNVR4689.1−FZD8
CNVR1748.1−CTBP1
CNVR302.2−WNT2B
CNVR3362.2−CAMK2B
CNVR6609.1−CREBBP
CNVR3362.1−CAMK2B
CNVR7784.2−PLCB4
CNVR6716.1−SIAH1
CNVR7784.4−PLCB4
CNVR6609.3−CREBBP
CNVR2634.3−PPP2R2B
CNVR6609.6−CREBBP
CNVR3610.1−CUL1
CNVR7546.1−PRKACA
CNVR8156.1−RAC2
CNVR5198.1−PLCB3
CNVR8271.1−TBL1X
CNVR7547.1−PRKACA
CNVR7434.1−NFATC1
CNVR2283.4−NKD2
CNVR1109.1−FZD7
CNVR6609.4−CREBBP
CNVR1747.1−CTBP1
CNVR7115.1−WNT3
CNVR7432.1−NFATC1
CNVR7227.16−RAC3
CNVR4800.1−CAMK2G
CNVR3363.1−CAMK2B
CNVR1787.1−PPP2R2C
CNVR6571.1−AXIN1
CNVR3352.1−SFRP4
CNVR195.1−JUN
CNVR7475.1−APC2
CNVR2020.1−PPP3CA
CNVR1643.1−TBL1XR1
CNVR5847.1−CSNK1A1L
CNVR2283.3−NKD2
CNVR2283.6−NKD2
CNVR6674.1−PRKCB
CNVR7703.1−PPP2R1A
CNVR1992.1−MAPK10
CNVR527.1−WNT3A
CNVR6673.1−PRKCB
CNVR7782.1−PLCB1
CNVR6359.1−PLCB2
CNVR7169.1−PRKCA
CNVR683.1−ROCK2
CNVR2283.5−NKD2
CNVR856.1−TCF7L1
CNVR1506.1−GSK3B
CNVR6609.5−CREBBP
CNVR6974.1−DVL2
CNVR2640.1−CSNK1A1
CNVR4864.1−FRAT2
CNVR6742.1−CSNK2A2
CNVR1786.1−PPP2R2C
CNVR2634.2−PPP2R2B
CNVR7783.1−PLCB4
CNVR2283.2−NKD2
CNVR7433.1−NFATC1
CNVR1991.1−MAPK10
CNVR2634.1−PPP2R2B
CNVR2019.2−PPP3CA
CNVR2019.1−PPP3CA
CNVR2283.1−NKD2
CNVR7784.1−PLCB4
CNVR13.1−DVL1
CNVR233.1−PRKACB
CNVR308.2−VANGL1
CNVR308.3−VANGL1
CNVR308.1−VANGL1
CNVR7716.1−PRKCG
CEU
YRI
CHB
0.0
0.1
0.2
0.3
0.4
0.5
0.6

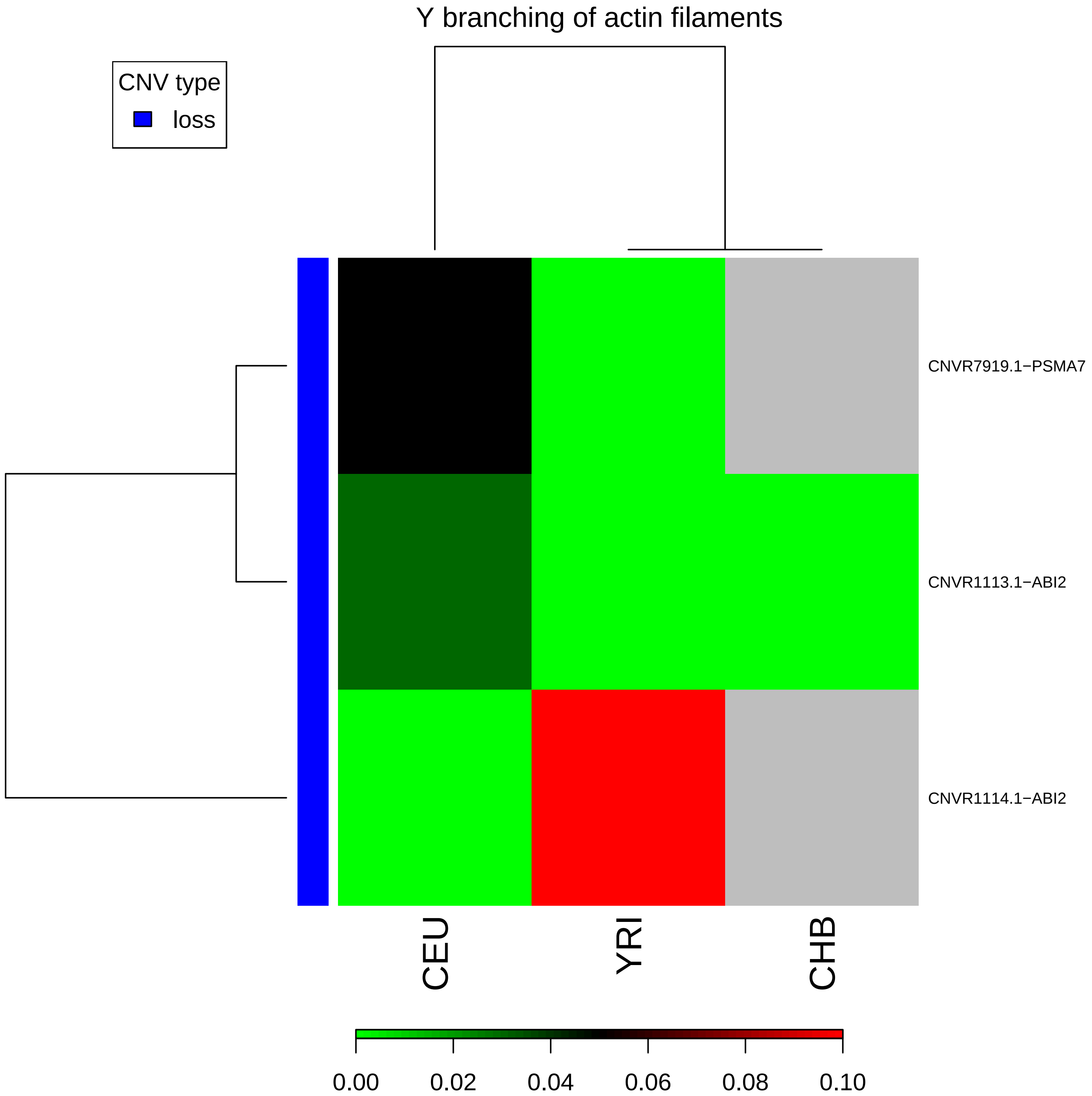

Y branching of actin filaments
CNV type
loss
CNVR7919.1−PSMA7
CNVR1113.1−ABI2
CNVR1114.1−ABI2
CEU
YRI
CHB
0.00
0.02
0.04
0.06
0.08
0.10

**Supplementary Figure 4. Rearrangements around *SORD* gene area in human and chimpanzee.**

Figure shows Mauve block alignment of four homologous regions in human and chimpanzee. First two regions are extracted from human reference genome (build hg18): chr15:43,080,000-43,163,000 and chr15:42,917,000-43,079,000, and the second two regions are taken from chimpanzee genomes (build panTro2): chr15:42,173,000-42,250,000 and chr15:41,950,000-42,030,000. The region of ~ 80kB that includes the gene *SORD* underwent inverse duplication before the split of human and chimpanzee. The active copy of the gene *SORD* is encoded on the plus strand and is shown in the orange color. The copy of the gene *SORD* on the minus strand, that is most likely became a pseudogene, is shown in the light orange. CNV resulted from a loss of a region in human genome from the inverted copy of the gene *SORD* (see empty box at the second alignment row). Analysis of the RepeatMasker annotation revealed that in the chimpanzee, the L1 element (L1PA3) is located right next to the CNV boundary. In the corresponding region in human we see that the same L1PA3 element was truncated from 50% of length to 15%, and the Alu element (AluJb) was inserted just at the location of CNV. However only 62% of Alu length remained in the sequence. The transposable elements activity can also be seen in the promoter area of the gene *SORD*. The remnants of retrovirus (HERV9, 50% of length) are present in the promoter region of three copies of *SORD* except the active human copy (first alignment row). Also, full length L1 element (L1PA6) that was most likely inserted in the retrovirus, is observed upstream the active copy of the gene *SORD*, and truncated copy of this element (65% of length) remained upstream the *SORD* pseudogene copy.

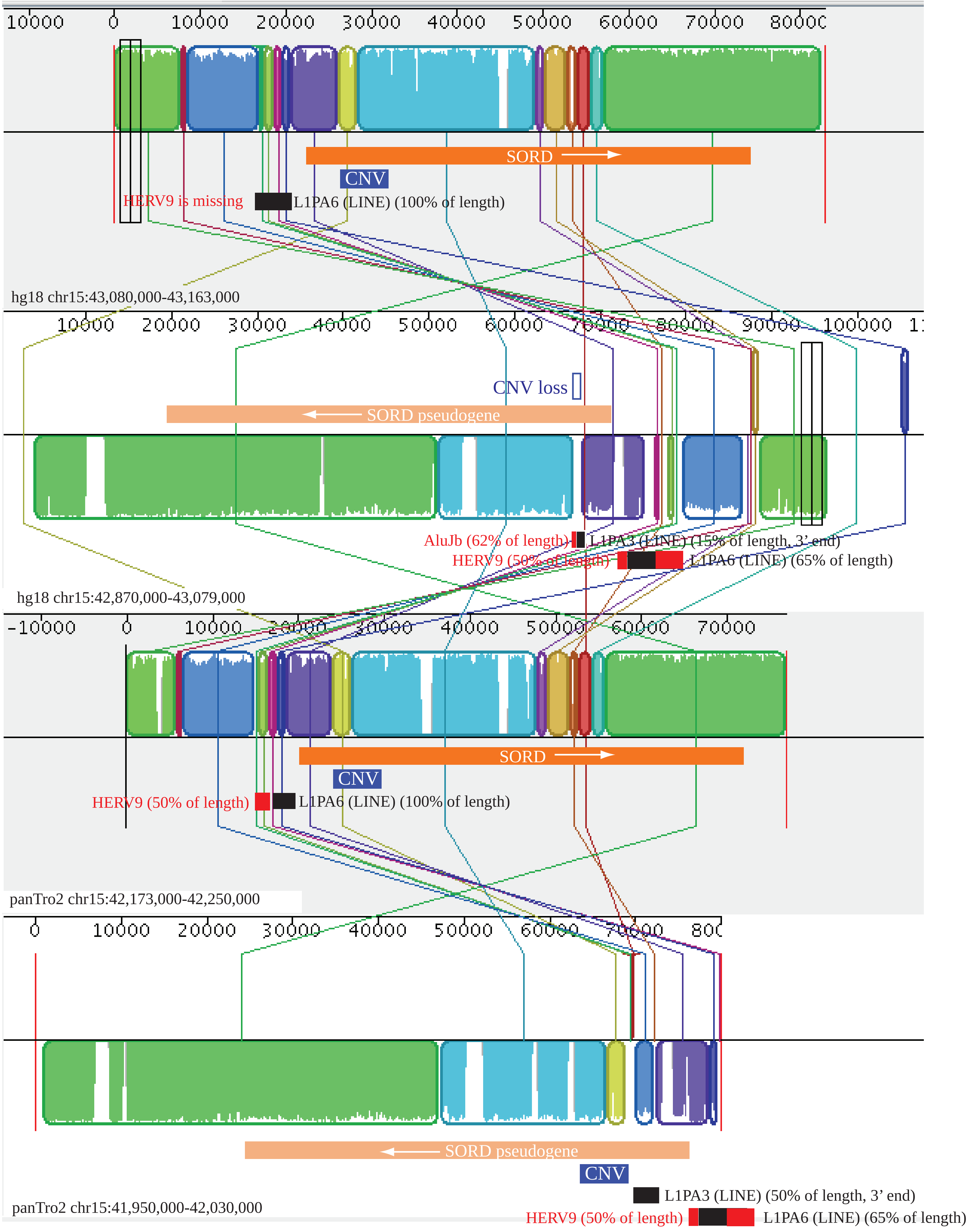

SORD
CNV
HERV9 is missing
L1PA6 (LINE) (100% of length)
hg18 chr15:43,080,000-43,163,000
CNV loss
SORD pseudogene
AluJb (62% of length)
L1PA3 (LINE) (15% of length, 3' end)
HERV9 (50% of length)
L1PA6 (LINE) (65% of length)
hg18 chr15:42,870,000-43,079,000
SORD
CNV
HERV9 (50% of length)
L1PA6 (LINE) (100% of length)
panTro2 chr15:42,173,000-42,250,000
SORD pseudogene
CNV
L1PA3 (LINE) (50% of length, 3' end)
panTro2 chr15:41,950,000-42,030,000
HERV9 (50% of length)
L1PA6 (LINE) (65% of length)

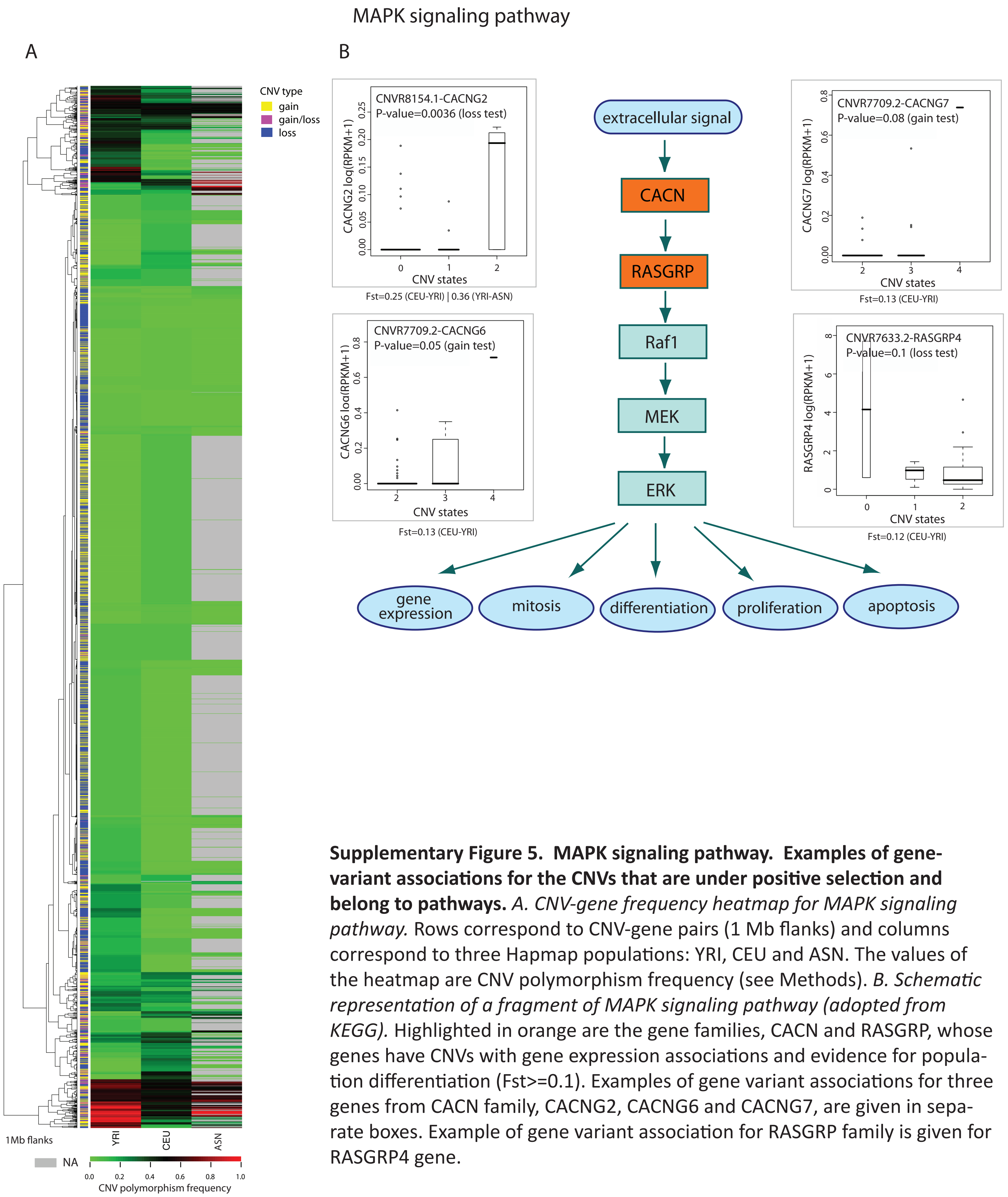


**Supplementary Figure 5. MAPK signaling pathway. Examples of gene-variant associations for the CNVs that are under positive selection and belong to pathways.** *A. CNV-gene frequency heatmap for MAPK signaling pathway.* Rows correspond to CNV-gene pairs (1 Mb flanks) and columns correspond to three Hapmap populations: YRI, CEU and ASN. The values of the heatmap are CNV polymorphism frequency (see Methods). *B. Schematic representation of a fragment of MAPK signaling pathway (adopted from KEGG).* Highlighted in orange are the gene families, CACN and RASGRP, whose genes have CNVs with gene expression associations and evidence for population differentiation (Fst>=0.1). Examples of gene variant associations for three genes from CACN family, CACNG2, CACNG6 and CACNG7, are given in separate boxes. Example of gene variant association for RASGRP family is given for RASGRP4 gene.

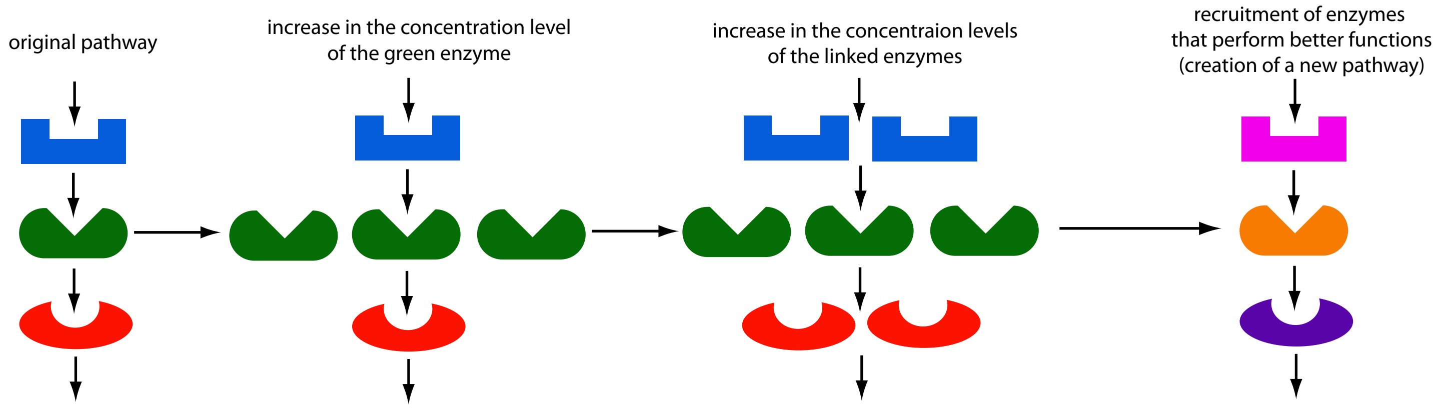


**Supplementary Figure 6. Tuning Effect of Pathway Evolution**. Different color and shape correspond to different enzymes. Increase in the concentration level of one enzyme (here green) can induce changes in the concentration levels of the linked enzymes (here blue and red). In the process of evolution, it can lead to the recruitment of enzymes that perform better functions, and as a result, create a new pathway.
